# Dipeptide-Derived Alkynes as Potent and Selective Irreversible Inhibitors of Cysteine Cathepsins

**DOI:** 10.1021/acs.jmedchem.2c01360

**Published:** 2023-03-03

**Authors:** Lydia Behring, Gloria Ruiz-Gómez, Christian Trapp, Maryann Morales, Robert Wodtke, Martin Köckerling, Klaus Kopka, M. Teresa Pisabarro, Jens Pietzsch, Reik Löser

**Affiliations:** † Helmholtz-Zentrum Dresden-Rossendorf, Institute of Radiopharmaceutical Cancer Research, Bautzner Landstraße 400, 01328 Dresden, Germany; ‡ BIOTEC, Technische Universität Dresden, Tatzberg 47-51, 01307 Dresden, Germany; § Institute of Chemistry, University of Rostock, Albert-Einstein-Straße 3a, 18059 Rostock, Germany; ∥ Technische Universität Dresden, School of Science, Faculty of Chemistry and Food Chemistry, Mommsenstraße 4, 01069 Dresden, Germany

## Abstract

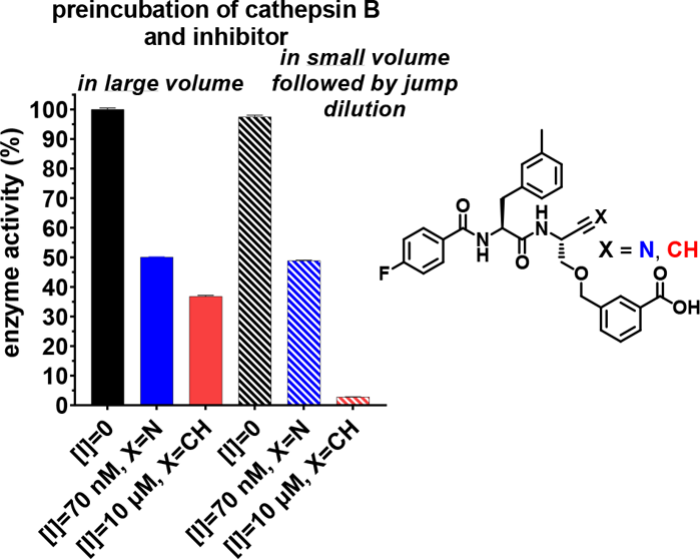

The potential of designing irreversible alkyne-based inhibitors of cysteine cathepsins by isoelectronic replacement in reversibly acting potent peptide nitriles was explored. The synthesis of the dipeptide alkynes was developed with special emphasis on stereochemically homogeneous products obtained in the Gilbert–Seyferth homologation for C≡C bond formation. Twenty-three dipeptide alkynes and 12 analogous nitriles were synthesized and investigated for their inhibition of cathepsins B, L, S, and K. Numerous combinations of residues at positions P1 and P2 as well as terminal acyl groups allowed for the derivation of extensive structure–activity relationships, which were rationalized by computational covalent docking for selected examples. The determined inactivation constants of the alkynes at the target enzymes span a range of >3 orders of magnitude (3–10 133 M^–1^ s^–1^). Notably, the selectivity profiles of alkynes do not necessarily reflect those of the nitriles. Inhibitory activity at the cellular level was demonstrated for selected compounds.

## Introduction

Tumor-associated proteolysis is considered a key driver of tumor invasion and metastasis. It involves proteases of all four major catalytic classes, which are represented by the serine, cysteine, and aspartic proteases and metalloproteases.^1−4^ A pivotal function of these protein-degrading enzymes in tumor progression is believed to originate from the cleavage of extracellular proteins, such as constituents of the extracellular matrix, precursors of growth factors, and cell adhesion proteins.^5,6^ However, the functional interplay among proteolytic enzymes, protein substrates, and endogenous inhibitors seems to be complex as intracellular proteolysis is also important for cancer initiation and progression,^7−9^ and even tumor-attenuating functions of proteases were identified.^10^ Nevertheless, the increased proteolytic activity of neoplastic tissues is increasingly harnessed for cancer diagnosis and therapy. In particular, an increased level of expression of certain proteases is of prognostic value for disease development^11,12^ and targeting of proteases either directly by inhibition with small molecules^13−16^ or by using substrate-based linkers as cleavage elements for prodrug activation of cytotoxic agents appears to be a promising strategy for cancer treatment.^17−19^

An important role among the tumor-associated proteases is played by the lysosomal papain-like cysteine proteases, which are part of the C1 family within clan CA according to the MEROPS classification^20,21^ and are termed cysteine cathepsins.^22^ The 11 human cysteine cathepsins (B, C, F, H, K, L, O, S, and V–X) share a high degree of sequence and structural homology with the plant enzyme papain.^23^ Furthermore, they are structurally and biochemically well-characterized enzymes, except for cathepsins O and W; their crystal structures have not been reported, and no catalytic activity was demonstrated for the latter. Most of the cysteine cathepsins are expressed ubiquitously, while cathepsins K, S, F, V, X, and W show a more cell and tissue specific distribution, as their expression is mainly restricted to osteoclasts (cathepsin K) and immune cells (cathepsins S, F, V, X, and W).^24^ Due to their lysosomal localization and broad specificity, cathepsins were long thought to be mainly involved in unspecific protein turnover as so-called housekeeping enzymes.^25,26^ While this picture is still correct to a certain extent, specific tasks of individual cathepsins were identified more recently. For example, cathepsin S is involved in MHC II invariant chain cleavage in professional antigen-presenting cells;^27,28^ cathepsin X takes part in insulin processing and T-cell signaling;^22^ cathepsin K secreted by osteoclasts plays an important role in bone remodeling via cleavage of collagen types I and II;^27^ and cathepsin L is involved in the proteolytic processing of neuropeptide precursors.^29^ By far the most extensively studied human cysteine protease is cathepsin B with regard to both its structural and biochemical properties and its pathophysiological functions. While cathepsin B is mainly localized in lysosomes under physiological conditions, its increased level of expression and secretion into the extracellular space is associated with different pathologies such as cancer, inflammatory respiratory syndrome, viral infections, rheumatoid arthritis, osteoarthritis, and pancreatitis.^26,30−32^

The cathepsins represent a heterogeneous group of proteases, which, in addition to the cysteine cathepsins, also includes the serine proteases cathepsins A and G as well as the aspartic proteases cathepsins D and E.^33^ Even though their involvement in tumor progression was supposed nearly a century ago,^34^ their particular functions in this context were mainly unraveled in the past three decades. Virtually all 11 human cysteine cathepsins were found to be associated with tumor progression to some extent, while the most compelling evidence was obtained for cathepsins B, L, S, and K and, furthermore, for cathepsin X, which—in contrast to the former enzymes—acts exclusively as a carboxymonopeptidase.^31,35−41^ Similar to other proteases, cathepsins are involved in tumor development and growth as well as associated processes such as angiogenesis, invasion, and metastasis.^42,43^ The overexpression of cysteine cathepsins B, L, S, and K is described for a plethora of tumors, and expression levels were correlated with poor prognosis.^40,44−49^ In the extracellular space, the cysteine cathepsins can degrade proteinaceous components of the extracellular matrix (ECM) such as collagen, elastin, fibronectin, and various proteoglycans and activate proteolytic cascades.^24,42,50−54^ Degradation of the ECM releases embedded growth factors, cytokines, and chemokines and thereby induces tumor growth, cell migration, and angiogenesis.^42^ Cleavage of the cell adhesion protein E-cadherin enhances metastasis.^55^ In addition to their predominantly extracellular effects associated with cancer, intracellular proteolytic processes mediated by cysteine cathepsins have also been linked to tumor progression.^56,57^ With regard to the particular function of cathepsin B in neoplastic diseases, the secretion of this cathepsin seems to be regulated by tumor-associated processes. Accordingly, the lower pH in the tumor microenvironment induces the relocation of cathepsin B-containing vesicles to the cell surface and the release of their content into the extracellular space.^25,58^ Additionally, the enzyme is secreted by tumor-associated benign cells such as macrophages, fibroblasts, osteoclasts, T-lymphocytes, and endothelial cells.^59,60^ The correlation between cathepsin B activity and the metastatic potential in B16 melanoma cells was demonstrated 40 years ago.^61^

Due to their unequivocal involvement in tumor progression, cysteine cathepsins are a vital target for cancer diagnosis and therapy.^42,62,63^ Therefore, inhibitors of various compound classes have been developed over the past three decades. In this context, the functionalization of substrate-derived peptidic moieties with electrophilic warheads proved to be a very fruitful approach toward the development of cysteine protease inhibitors.^23,64^ In particular, peptidic nitriles represent an attractive chemotype of inhibitors, because the softly electrophilic cyano group interacts preferably with active-site thiol groups, while serine proteases are generally much less prone to interact with nitriles.^65−67^ However, in the case of the prolyl oligopeptidases (family S9 of serine proteases), highly potent inhibition by peptidic nitriles has been observed.^68^ However, the inherent reactivity of the cyano group can result in indiscriminate thioimidate formation with other biogenic thiols such as glutathione, even though this is strongly dependent on the chemical surrounding of the C≡N bond.^69^ This is particularly important for the development of inhibitor-derived imaging probes, as any off-target reactivity will compromise the image contrast, as encountered during the development of azadipeptide nitriles as cysteine cathepsin-targeting PET tracers.^70,71^ An approach for obtaining lower-reactivity inhibitors is the isoelectronic replacement of the cyano nitrogen atom by methylidyne leading to peptide-derived alkynes, which, however, often abolishes inhibitory activity completely as a consequence of the greatly reduced electrophilicity.^72^ Alkynes, nevertheless, represent potentially electrophilic species,^162^ and nucleophiles including thiols can react with the C≡C bond under the formation of the corresponding vinylated products.^73,74^ However, in the case of thiols, this usually requires specialized conditions such as the presence of free radical initiators, catalytic bases at high temperature and pressure, or strongly basic conditions at higher temperatures^75^ and, furthermore, catalysis by π-electrophilic transition metals.^76,77^ Considering the extraordinary nucleophilicity of the active-site thiol in cysteine proteases and related enzymes, it does not appear to be too surprising that extended peptides functionalized with C-terminal ethynyl groups derived from efficient substrates, which confer multiple contacts on the enzyme, act as irreversible inhibitors, in contrast to their reversibly acting nitrile analogues. This was initially observed for deubiquitinating enzymes with ubiquitin-derived propargylamide.^78,79^ Furthermore, it was also demonstrated that other cysteine proteases such as caspase-1 can be covalently targeted by peptide-derived alkynes.^79^ Potent and selective irreversible inhibitors are attractive from multiple biomedical perspectives.^80^ Compared to reversible inhibitors, for which an equilibrium is attained between the inhibited and free enzyme, binding of irreversible inhibitors leads to permanent suppression of the target enzyme, which can be restored only by resynthesis of the protein. From a pharmacological point of view, this means that irreversible inhibition will remain even after elimination of the drug from the circulation, and thus, pharmacodynamic action can be partially decoupled from pharmacokinetics.^81,82^ In terms of designing molecular probes for detection and imaging of target enzymes, irreversible inhibitors offer the development of activity-based probes by equipping the inhibitor molecule with reporter groups. If radionuclides are used for the latter, radiotracers will be obtained, which can detect the target protein in a robust and quantitative manner in complex biological sample material.^83^ Provided that suitable radionuclides such as fluorine-18 and iodine-123 are used, such probes can also be employed for noninvasive imaging *in vivo* by employing PET and SPECT as imaging modalities, respectively. For the *in vivo* application of radiotracers based on irreversible inhibitors, the aspect of decoupling pharmacodynamics from pharmacokinetics for therapeutic treatment could potentially translate to increased image contrast for the radiotracer–protein complex after clearance of the unbound radiotracer. However, this remains to be proven experimentally.

Given the diagnostic, prognostic, and therapeutic potential of targeting the tumor-associated cysteine cathepsins, highly specific PET and SPECT probes are highly desirable, which could be potentially obtained in targeted radiolabeled peptide-derived alkynes. Considering the similar tubular shape of the electron cloud of the C≡C and C≡N bonds, starting from potent irreversibly inhibiting nitriles seems to be promising for obtaining potent alkyne-based irreversible inhibitors. Given its central role in tumor progression, the design of alkynes was first carried out for cathepsin B starting from potent dipeptide nitriles. In addition to the focus on cathepsin B, the potency and selectivity of the designed inhibitors toward cysteine cathepsins of oncological relevance, such as the cathepsins L, S, and K, were also taken into consideration.

## Results and Discussion

### Compound Design and Synthesis

**Figure 1:**
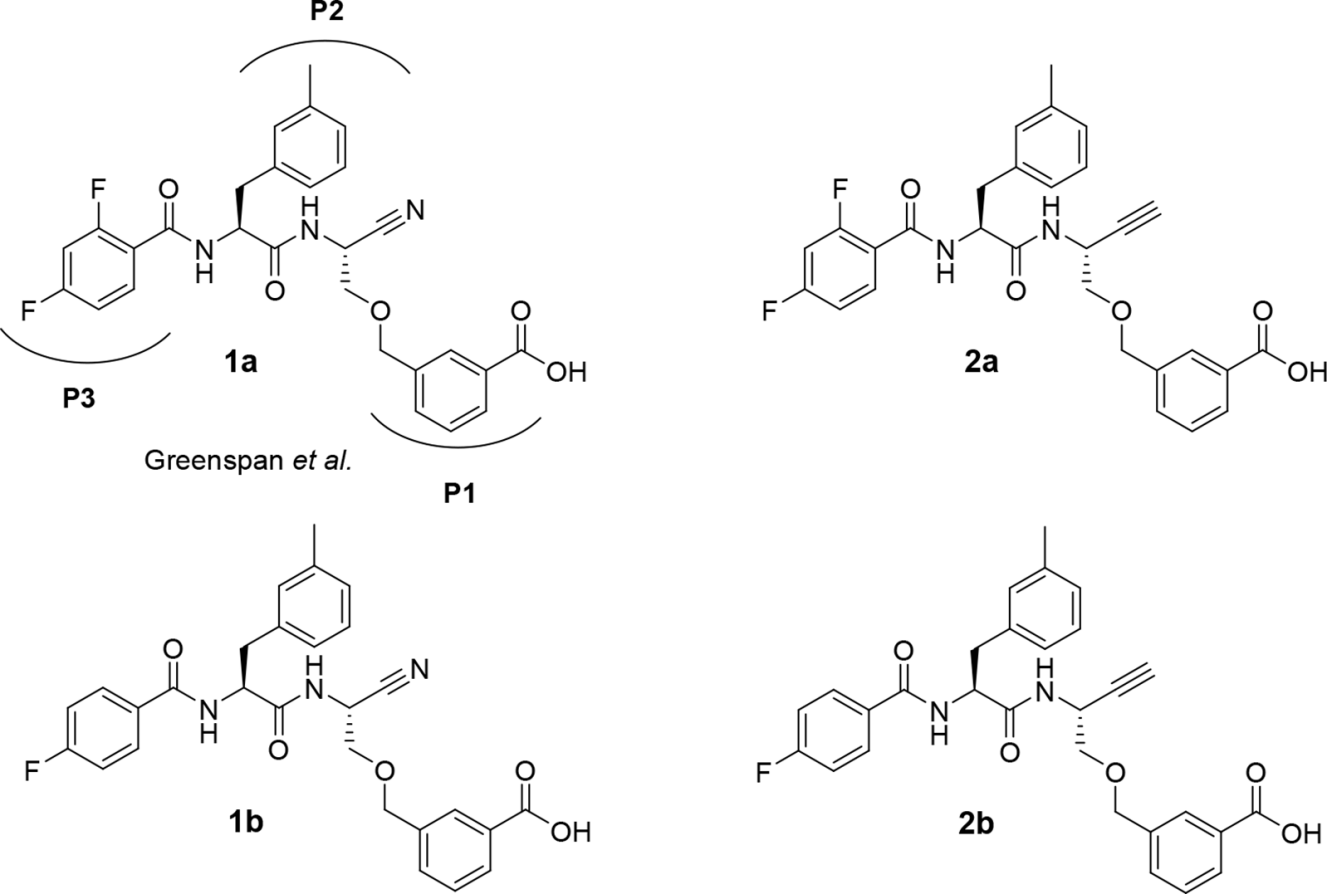
Cathepsin B-inhibitory dipeptide nitriles **1a** (reported by Greenspan et al.^86^) and **1b** as lead compounds for the design of dipeptide-derived alkynes **2a** and **2b**, respectively, as potentially irreversible inhibitors of cathepsin B. Protease subsite-targeting moieties P1–P3 are highlighted for nitrile **1a**.

Considering the oncological importance of cathepsin B, this cysteine protease became the focus of inhibitor development. This enzyme is unique among the papain-like proteases in the presence of the occluding loop, which is a flexible 20mer insertion conferring carboxydipeptidase activity to cathepsin B in addition to its function as endopeptidase, because two histidine residues, His 110 and His 111, can act as hydrogen bond/salt bridge interaction partners toward the terminal carboxylate of peptidic substrates.^84,85^ Potent dipeptide-derived nitriles targeting this enzyme were reported by Greenspan et al.^86^ The high inhibitory potency of these compounds is conferred by a *m*-carboxybenzylserine residue at P1 (nomenclature for proteases subsites according to Schechter and Berger^87^), whose carboxylic group was proposed to engage in salt bridge-like interactions with one histidine (His 110) of the occluding loop, although direct structural X-ray crystallography evidence for such contacts was not obtained. As the identification of radiotracer candidates for PET and SPECT imaging was a major goal of our work, the 2,4-di- and 4-monofluorobenzoylated dipeptide nitriles, **1a** and **1b**, respectively, were selected as lead compounds (Figure 1), which would enable prospective radiolabeling with the PET nuclide fluorine-18.

### Synthesis of Dipeptide Nitriles with a *m*-Carboxybenzylserine Residue at P1 (compounds **1a**–**e**)

**Scheme 1:**
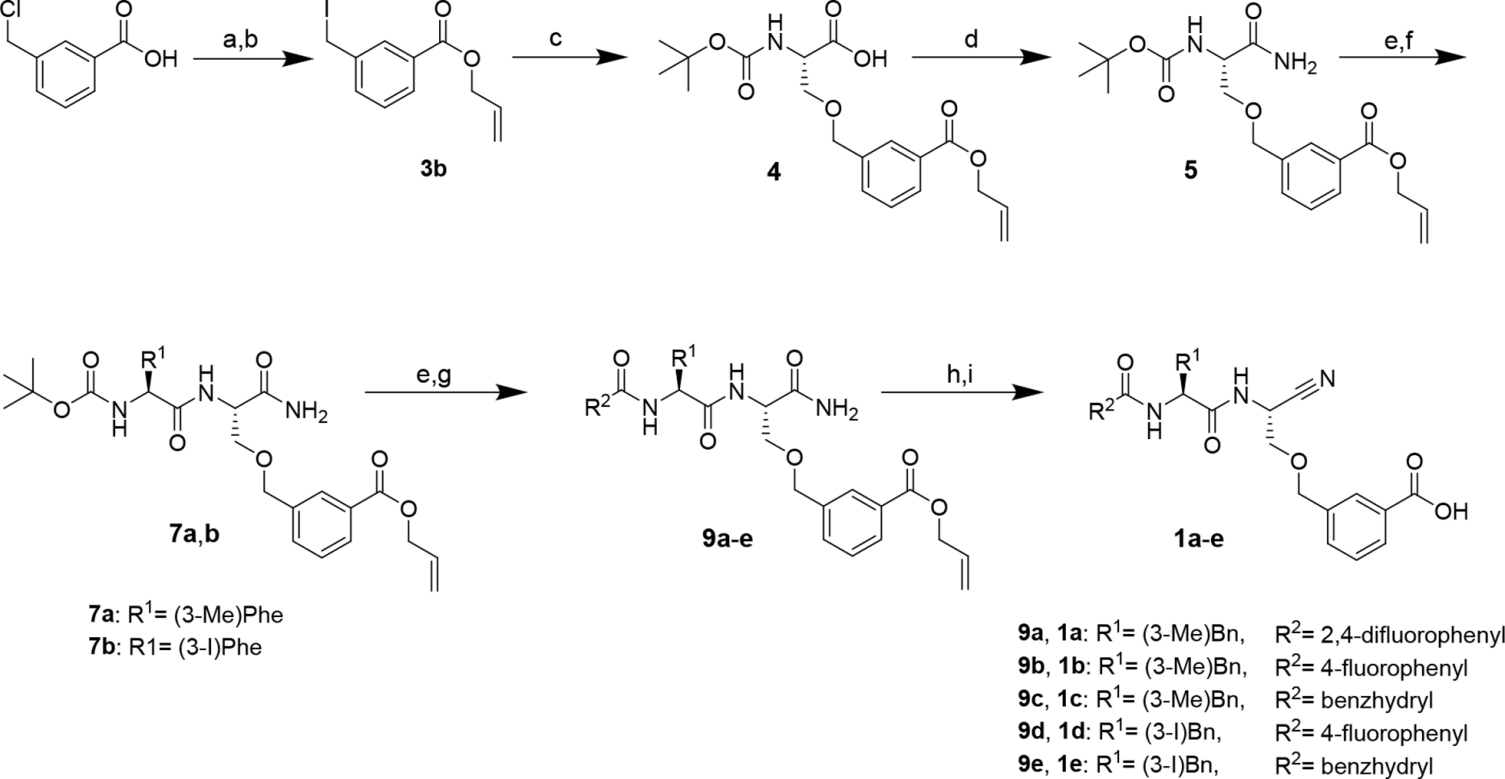
Synthesis of Dipeptide Nitriles **1a–e** Reagents and conditions: (a) allyl bromide, K_2_CO_3_, acetone, reflux, 2 h; (b) NaI, acetone, 5 h; (c) Boc-L-serine, NaH, DMF, 0 °C, 15 min, rt, 30 min; (d) iBCF, NMM, NH_3_, THF, −10 °C, 10 min, rt, 30 min; (e) TFA/CH_2_Cl_2_ (1:1), 2 h; (f) *N*-Boc-amino acid, DIPEA, PyBOP, THF, 3 h; (g) acyl chloride, TEA, CH_2_Cl_2_, 2 h, or carboxylic acid, DIPEA, PyBOP, THF, 3 h; (h) cyanuric chloride, DMF, 2 h; (i) Pd(PPh_3_)_4_, morpholine, CH_2_Cl_2_, 30 min.

The synthesis of these nitrile-derived inhibitors was performed following the published procedure (Scheme 1).^86^ The route started with 3-chloromethylbenzoic acid, which was converted into allyl 3-(iodomethyl)benzoate (**3b**). Boc-serine was side-chain O-alkylated with **3b** to obtain compound **4**. This serine derivative was transformed into the corresponding primary amide by *in situ* generation of the mixed anhydride with isobutyl chloroformate (iBCF) and subsequent reaction with aqueous ammonia. In contrast to Greenspan et al., who introduced the cyano group in the following step, we decided to perform this at a later stage, because dipeptide nitriles are potentially unstable under the strongly acidic conditions required for Boc removal.^88^ Therefore, in the next steps, the Boc protecting group of amide **6** was removed with TFA in CH_2_Cl_2_, and the resulting free amino acid amide was coupled to Boc-(3-methyl)phenylalanine as a P2-amino acid using PyBOP in the presence of diisopropylethylamine (DIPEA) as the base. After removal of the Boc protecting group, the N-terminal capping group was introduced as a moiety for potential targeting of the S3 binding region. To obtain **1a**, ammonium trifluoroacetate **8a** was reacted with 2,4-difluorobenzoyl chloride in the presence of *N*-methylmorpholine (NMM) as the base. Dehydration of the resulting primary dipeptide amide into the corresponding nitrile was achieved with cyanuric chloride in dry DMF. In the final reaction step, the carboxylic group was deprotected by the Pd(0)-catalyzed cleavage of the allyl ester to afford **1a** or **1b**. In the synthesis of **1a**, phenylsilane was used as an allyl scavenger,^89^ but the corresponding carboxylic acid was obtained in an only yield of 15%. Employing morpholine as an alternative allyl acceptor for the synthesis of **1b** resulted in a significantly higher deprotection yield of 55%. The dipeptide nitriles were obtained in overall yields of 1% and 5% for **1a** and **1b**, respectively. The latter yield is in the range of the procedure published by Greenspan et al.^86^ Dipeptide nitriles **1c**–**e** were synthesized using a procedure analogous to that described above. Detailed procedures and analytical data are given in the Synthesis section of the Supporting Information.

### Synthesis of the Dipeptide Alkyne Analogue of Nitrile **1a** as a Diastereomeric Mixture (compound **18**)

**Scheme 2:**
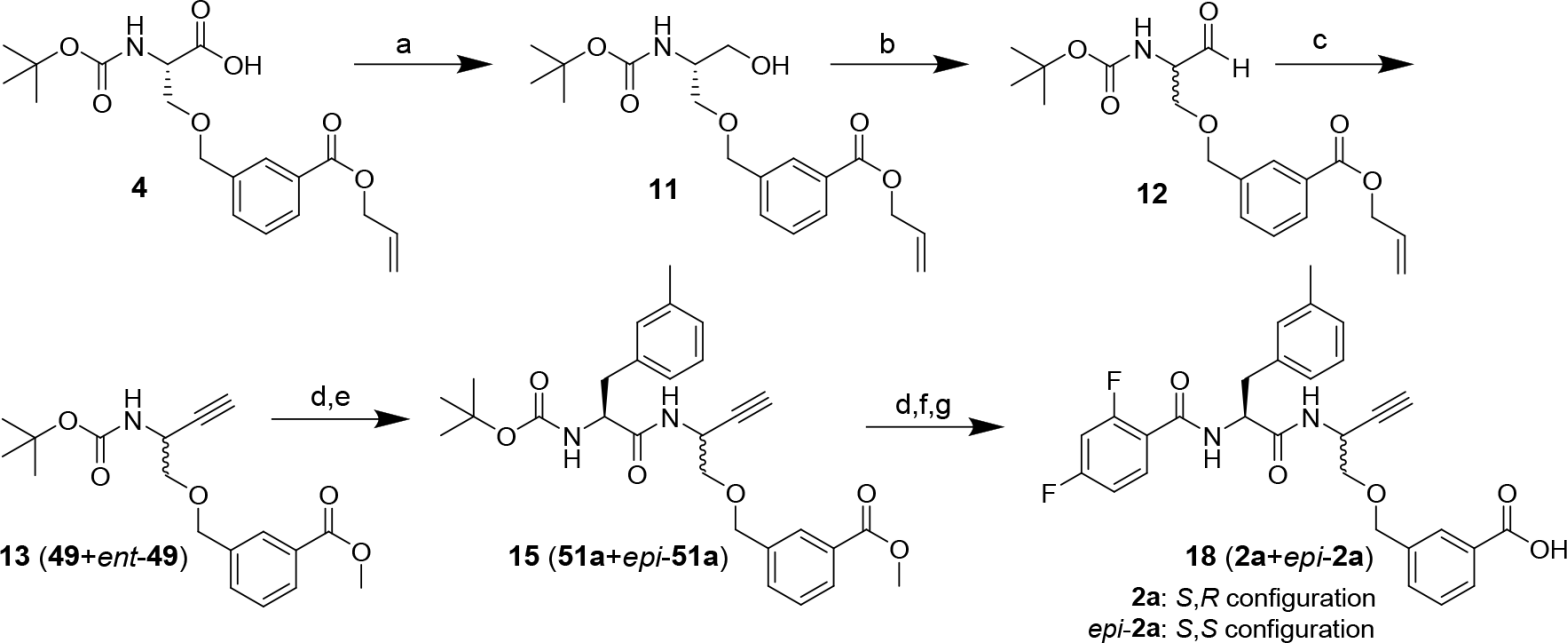
Synthesis of Dipeptide Alkyne **18** as a Diastereomeric Mixture See Scheme 6 for the structural definition of stereochemically pure compounds. Reagents and conditions: (a) PyBOP, DIPEA, NaBH_4_, THF, 1 h; (b) Dess-Martin periodinane, CH_2_Cl_2_, 4 h; (c) dimethyl (1-diazo-2-oxopropyl)phosphonate, K_2_CO_3_, MeOH, 0 °C, 2 h, rt, overnight; (d) TFA/CH_2_Cl_2_ (1:1), 2 h; (e) *N*-Boc-3-methyl-l-phenylalanine, PyBOP, DIPEA, THF, 3 h; (f) 2,4-difluorobenzoyl chloride, NMM, CH_2_Cl_2_, 2.5 h; (g) NaOH, THF/MeOH (3:1), overnight.

**Figure 2:**
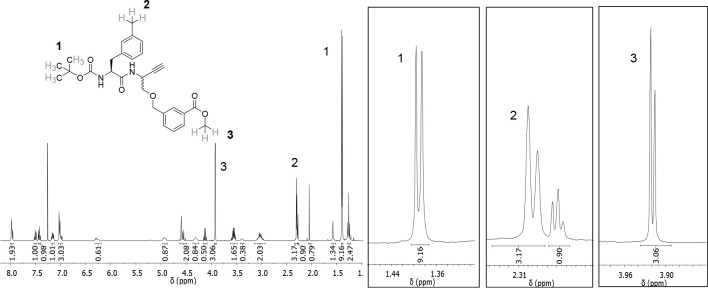
^1^H NMR spectrum of dipeptide alkyne **15** in CDCl_3_ and detailed view of relevant signals (right), whose doubling indicates the presence of a diastereomeric mixture.

Initially, the synthesis of dipeptide alkyne **18** was performed like that of dipeptide nitrile **1a**. Therefore, the ethynyl group was introduced by C–C bond formation at the α-carboxylic group of building block **4** containing the allyl-protected P1 targeting moiety (Scheme 2). To this end, **4** was transformed into the corresponding aldehyde via alcohol **11**, which was obtained by reduction of the in situ-generated **4**-derived HOBt ester with sodium borohydride.^90^ This step was followed by oxidation to aldehyde **12** using Dess-Martin periodinane. Subsequently, **12** was subjected to a Gilbert–Seyferth homologation by treatment with the Bestmann–Ohira reagent^91,92^ to obtain alkyne **13**. Due to the basic reaction conditions and the long reaction time, a transesterification of the allyl into the methyl ester occurred. Because the methyl group introduced in the process continued to serve as a protecting group that can be removed by hydrolysis in the final reaction step, the transesterification did not affect the subsequent steps of the synthetic pathway. Following homologation, the Boc protecting group was removed from **13** and the resulting 2-aminoalkyne was coupled to 3-methylphenylalanine as the P2 residue to obtain dipeptide alkyne **15**. However, the ^1^H NMR spectrum of purified compound **15** revealed additional signals (Figure 2). As impurities other than isomeric compounds were excluded on the basis of LC-MS, we concluded that partial epimerization occurred, most likely under the basic conditions of the homologation. Hence, compound **15**, the following intermediates, and final product **18** were obtained as diastereomeric mixtures (4:5 ratio for stereochemically impure **18** in favor of desired diastereomer **2a**).

### Synthesis of Dipeptide Nitriles and Dipeptide Alkynes with Carboxy-Functionalized 1,2,3-Triazolyl Residues at P1 (compounds **28** and **35a**–**c**)

**Figure 3:**
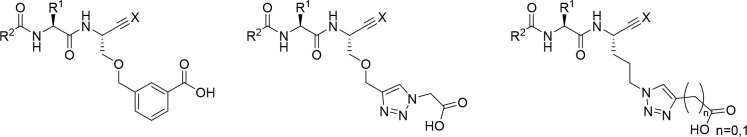
Carboxy-functionalized P1 residues intended as occluding loop-targeting moieties in the dipeptide-derived cathepsin B inhibitor. Structure of the P1 residue described by Greenspan et al. (left; X = N) and Schmitz et al. (middle; X = N) and derived alternative P1 residue (right) for the design of cathepsin B-directed dipeptide alkynes (X = CH).

As epimerization/racemization to a greater extent was apparently not noted for other α-amino and peptide aldehydes according to the literature,^79,93,94^ the partially lost stereochemical integrity during the homologation reaction to **15** was attributed to the −*I* effect of the ether oxygen atom. Therefore, an alternative residue at P1 was considered. As Greenspan et al. described the carboxylic group at P1 as an important moiety for conveying potent cathepsin B inhibition,^86^ this group had to be maintained. Schmitz et al. published a selective, nitrile-based cathepsin B inhibitor with a carboxy-functionalized 1,2,3-triazolyl moiety at P1 as the occluding loop binding element [when R^1^ = 3-bromobenzyl and R^2^ = 4-biphenyl, *K*_i_ = 41.3 nM (Figure 3)].^95^ As the oxygen at the γ-position contained in the inhibitors described by Schmitz and Greenspan probably promotes racemization by favoring proton abstraction at the C_α_-H group, a pure hydrocarbon-based side-chain linker was envisaged at that position. Therefore, the corresponding dipeptide nitrile/alkyne pair shown in Figure 3 was synthesized. An N-terminal capping group 4-fluorobenzoyl was chosen to enable perspective labeling with fluorine-18.

**Scheme 3:**
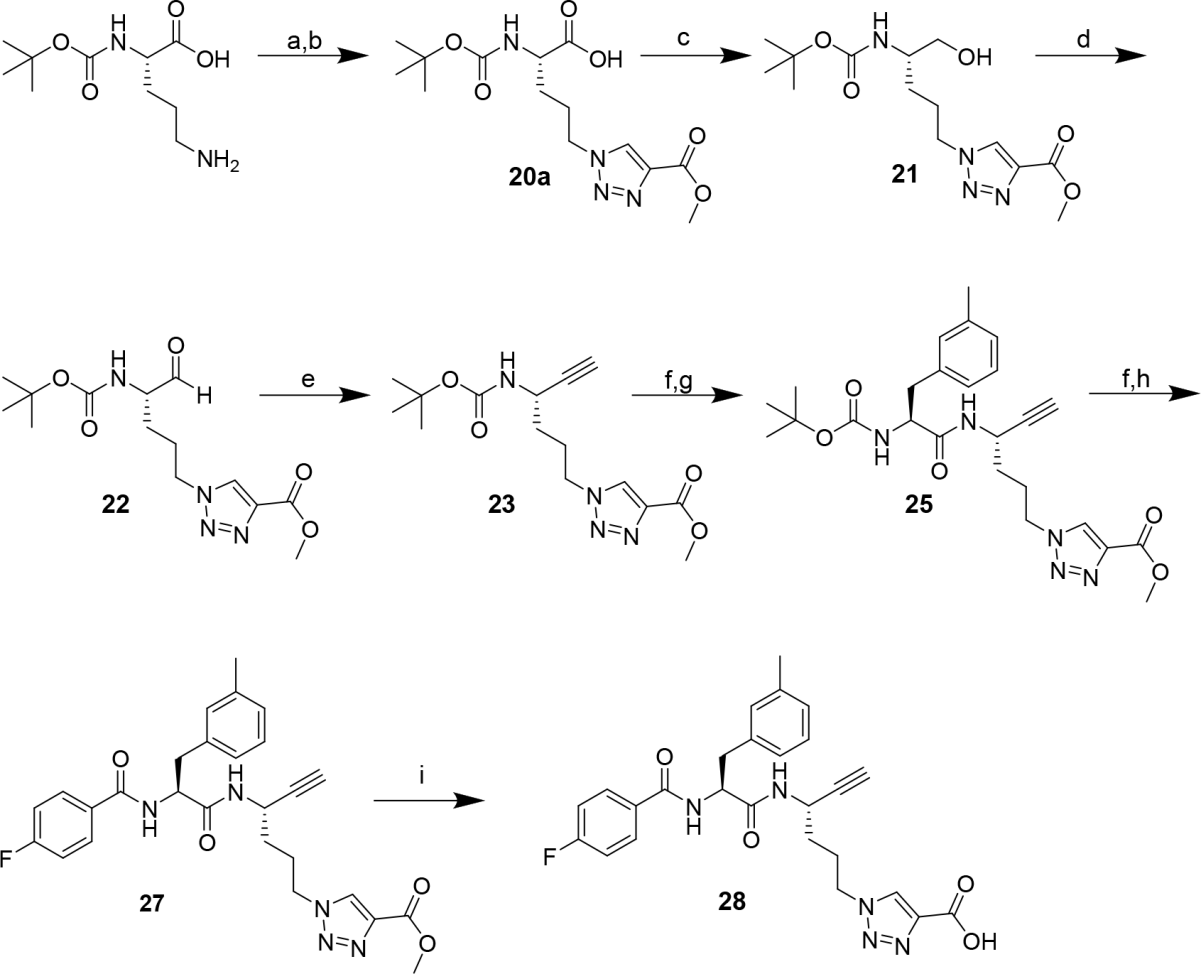
Synthesis of Dipeptide Alkyne **28** Containing a Carboxy-Functionalized 1,2,3-Triazolyl Moiety at P1 Reagents and conditions: (a) triflyl azide, CuSO_4_·5H_2_O, K_2_CO_3_, MeOH/H_2_O (3:1), overnight; (b) methyl propiolate, CuSO_4_·5H_2_O, sodium ascorbate, DMSO/H_2_O (2:1), overnight; (c) DIPEA, NaBH_4_, PyBOP, THF, 1 h; (d) Dess-Martin periodinane, CH_2_Cl_2_, 1 h; (e) dimethyl (1-diazo-2-oxopropyl)phosphonate, K_2_CO_3_, MeOH, 0 °C, 2 h, rt, 3 h; (f) TFA/CH_2_Cl_2_ (1:1), 2 h; (g) Boc-3-methyl-l-phenylalanine, PyBOP, DIPEA, THF, 3 h; (h) 4-fluorobenzoyl chloride, NMM, CH_2_Cl_2_, 2 h; (i) NaOH, THF/MeOH (3:1), overnight.

For the construction of the P1 moiety, Boc-l-ornithine was transformed into δ-azidonorvaline by diazo transfer with triflyl azide.^96^ Subsequently, the 1,2,3-triazolyl moiety was formed in a copper(I)-catalyzed 1,3-dipolar azide–alkyne cycloaddition in a manner like the procedure described by Schmitz et al. (Scheme 3, steps a and b)^95^ The synthesis of dipeptide alkyne **28** was performed as described above for the synthesis of **18** starting from the P1 amino acid containing the 1,2,3-triazolyl moiety [**20a** (Scheme 3)].

**Figure 4:**
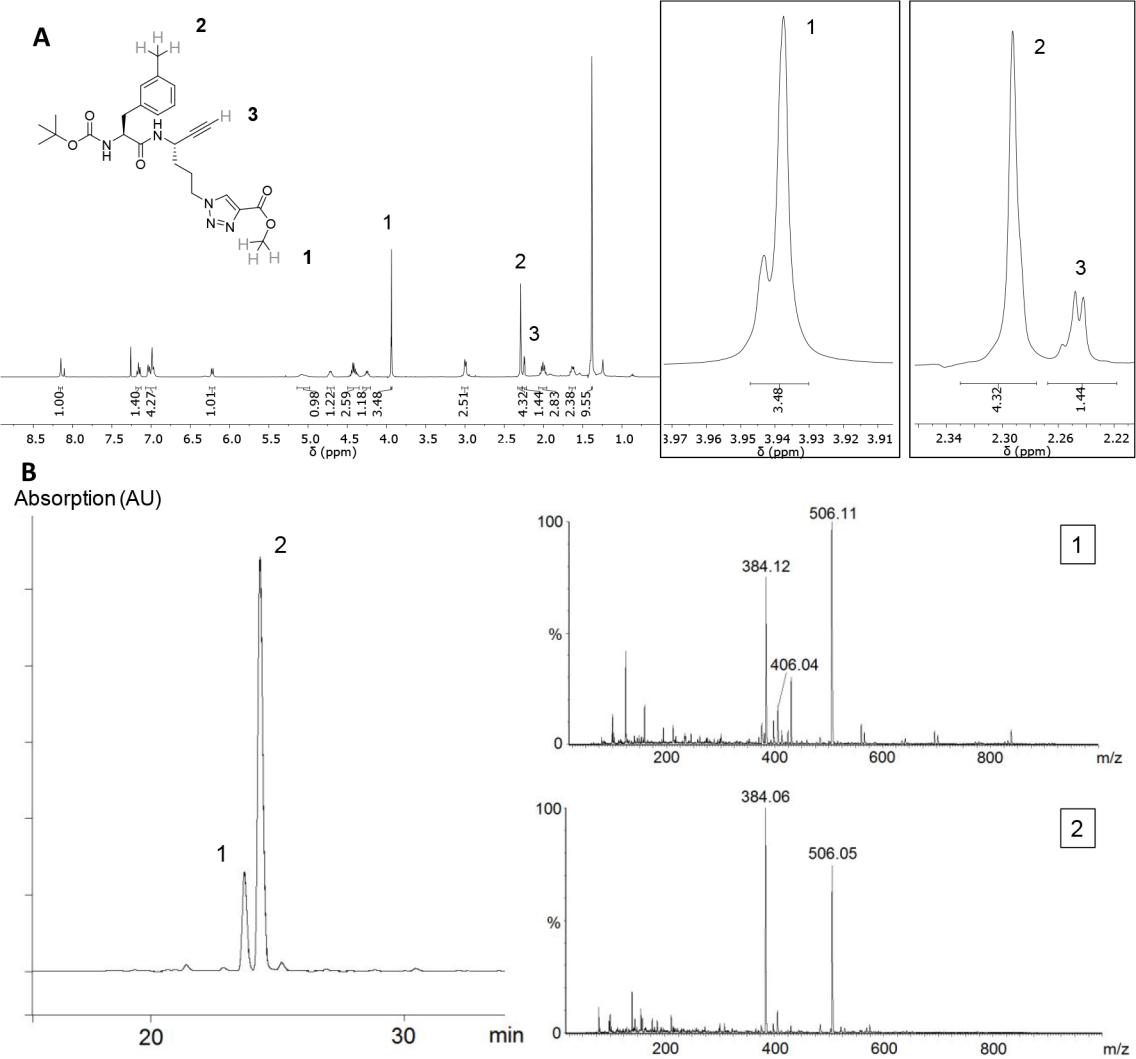
(A) ^1^H NMR spectrum of dipeptide alkyne **25** in CD_3_Cl and detailed view of selected signals (right). (B) HPLC chromatogram of **25** (left) and ESI-MS spectra corresponding to the peaks (right).

The reaction progress of the reduction, oxidation, and homologation of **20a**, **21**, and **22**, respectively, was closely monitored via mass spectrometry. The Gilbert–Seyferth homologation of **22** was stopped after 3 h to keep the exposure to the potentially detrimental basic conditions as short as possible. The stereochemical purity was checked via NMR spectroscopy after coupling of 3-methyl-l-phenylalanine at P2 [compound **25** (Figure 4A)]. Even though the signals of the two diastereomers overlap, the signals of the methyl hydrogens at the ester moiety appear at distinct chemical shifts [*1* (Figure 4A)]. On this basis, the ratio of both diastereomers was determined to be 1:3 in favor of the desired isomer. Hence, the level of epimerization was higher than expected but lower than in the case of dipeptide alkyne **15**. This finding supports the assumption that the presence of oxygen at the γ-position promotes epimerization at P1 C_α_ under basic conditions. Unlike **15**, the obtained diastereomers could be separated via HPLC (Figure 4B). In the following reaction steps, the residues at P2 and P3 were coupled and the methyl group was removed as described above. The crude product was purified via semipreparative HPLC, and the diastereomeric purity verified via ^1^H NMR (>99%). Dipeptide alkyne **28** with 4-(carboxy-1*H*-1,2,3-triazol-1-yl)-l-norvaline at P1 was obtained with an overall yield of 1% over 10 steps. This is comparable to the synthesis of the mixture of diastereomeric dipeptide alkynes **2a** and *epi*-**2a** (**18**) with *O*-(3-carboxybenzyl)serine at P1 (>1% over 11 steps).

Because dipeptide alkyne **28** showed no signs of irreversible inhibition of cathepsin B (see Figure S45), the synthesis of further dipeptide alkynes with carboxy-functionalized 1,2,3-triazolyl residues at P1 was not pursued.

**Scheme 4:**
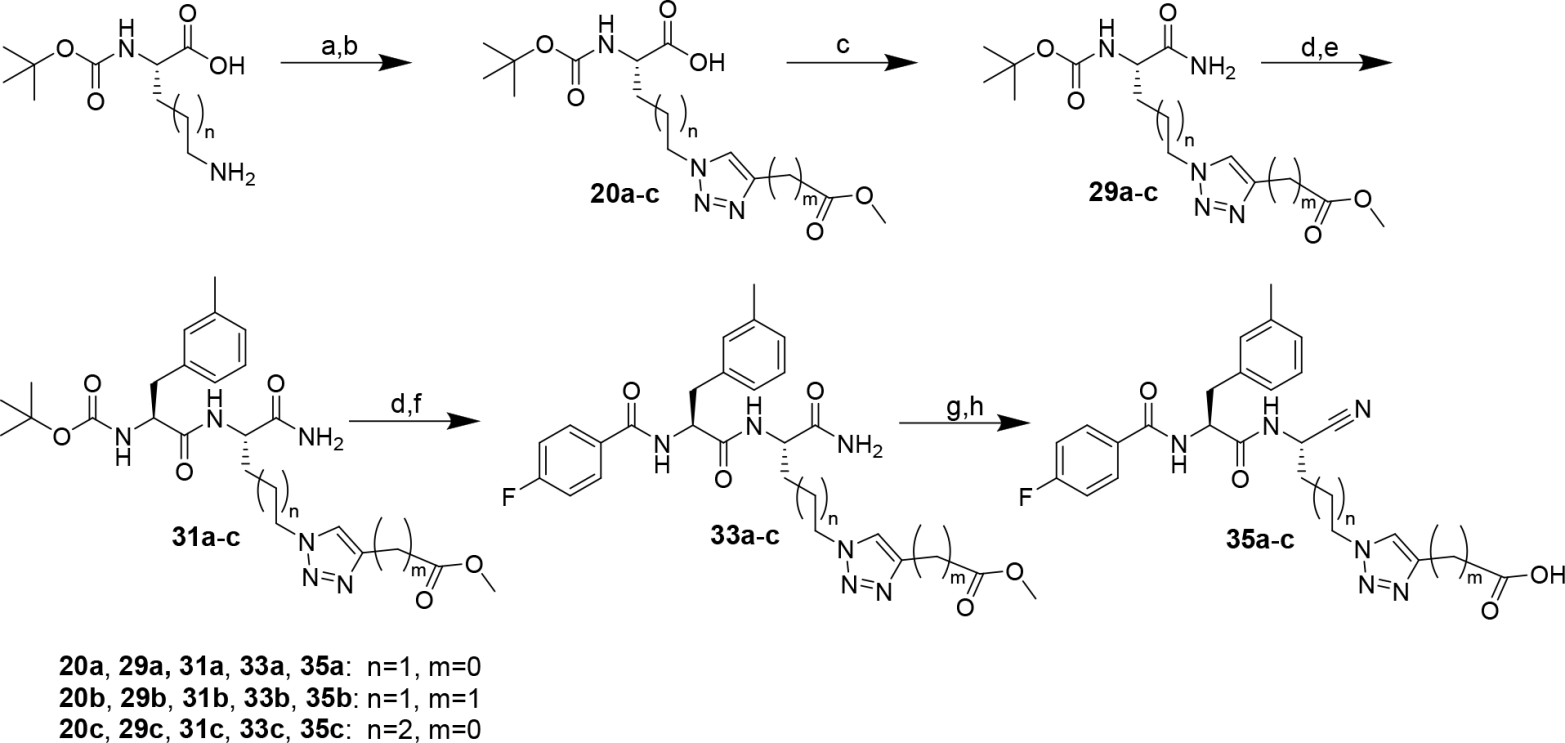
Synthesis of Dipeptide Nitriles **35a**–**c** with a Carboxy-Functionalized 1,2,3-Triazolyl Residue at P1 Reagents and conditions: (a) triflyl azide, CuSO_4_·5H_2_O, K_2_CO_3_, MeOH/H_2_O (3:1), overnight; (b) methyl propiolate or methyl butynoate, CuSO_4_·5H_2_O, sodium ascorbate, DMSO/H_2_O (2:1), overnight; (c) iBCF, NMM, NH_3_, THF, −15 °C, 10 min, rt, 30 min; (d) TFA/CH_2_Cl_2_ (1:1), 2 h; (e) Boc-3-methyl-l-phenylalanine, DIPEA, PyBOP, THF, 3 h; (f) 4-fluorobenzoyl chloride, TEA, CH_2_Cl_2_, 2 h; (g) cyanuric chloride, DMF, 3 h; (h) NaOH, THF/MeOH (3:1), overnight or pig liver esterase, KH_2_PO_4_ buffer (0.2 M, pH 7.0)/acetone (10:1), 6–10 days.

**Scheme 5:**
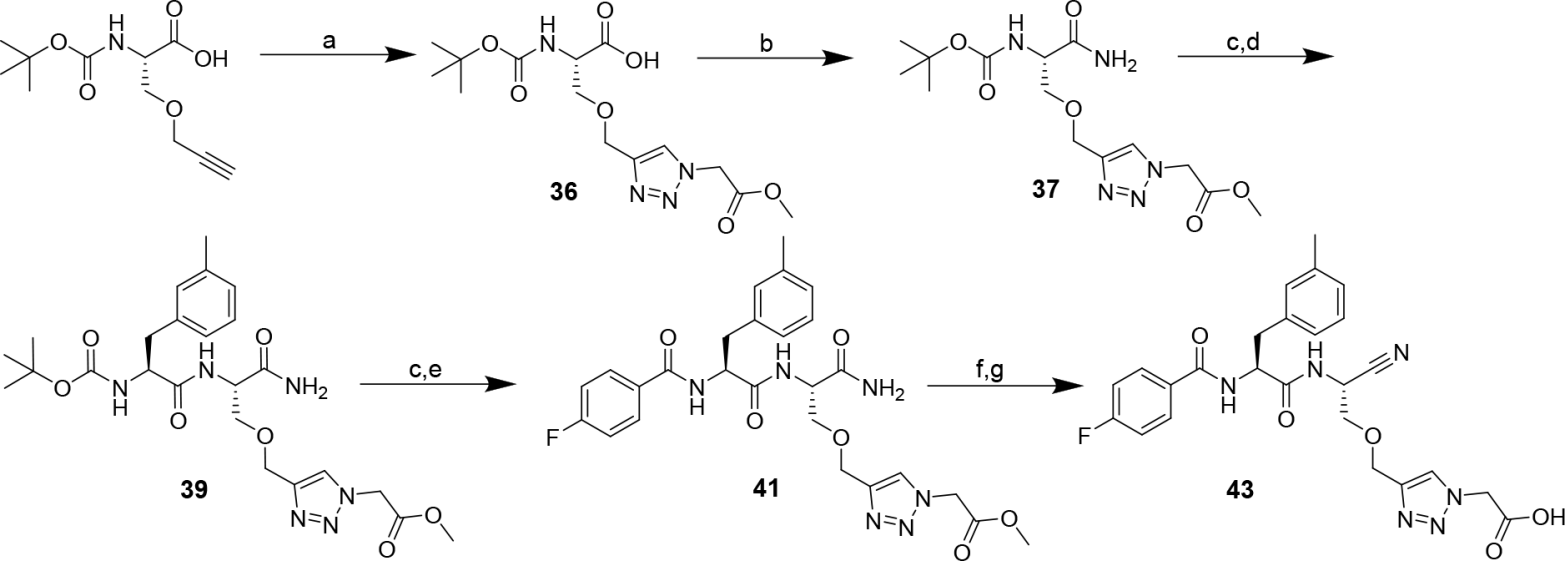
Synthesis of Serine-Based Dipeptide Nitrile **43** with a Carboxy-Functionalized 1,2,3-Triazolyl Residue at P1 Reagents and conditions: (a) methyl-2-azidoacetate, CuSO_4_·5H_2_O, sodium ascorbate, DMSO/H_2_O (1:2), 0 °C, 1 h, rt, overnight; (b) iBCF, NMM, NH_3_, THF, −15 °C, 10 min, rt, 30 min; (c) TFA/CH_2_Cl_2_ (1:1), 2 h; (d) Boc-3-methyl-l-phenylalanine, PyBOP, DIPEA, THF, 3 h; (e) 4-fluorobenzoyl chloride, TEA, CH_2_Cl_2_, 2 h; (f) cyanuric chloride, DMF, 2 h; (g) LiOH, THF/H_2_O (5:1), 0 °C, 5 min; procedure following ref (95).

To obtain further insights into the recognition of carboxytriazole-functionalized side chains at P1 by the cysteine cathepsins, nitrile analogue **35a** of alkyne **28** and additional derivatives containing a carboxymethyl group in place of the directly attached carboxylic group and extended side-chain linker, compounds **35b** and **35c**, respectively, were synthesized according to Scheme 4 (complete procedure described in the Supporting Information). Ester hydrolysis in the final step by saponification with sodium hydroxide resulted in a slightly diminished yield for nitrile **35a** compared to that of the analogous alkyne **28**, with dipeptide nitrile **35a** being obtained with an overall yield of 3% over nine steps. More favorable yields were achieved for **35b** and **35c**, for which the corresponding methyl esters were cleaved by pig liver esterase-catalyzed hydrolysis. Furthermore, the related nitrile containing an “inverted” triazole ring attached to a serine-derived side chain (compound **43**) was prepared from commercially available *O*-propargylserine according to Scheme 5. Basic hydrolysis for transforming methyl ester **42** into carboxylic acid **43** was problematic due to substantial β-elimination resulting in the formation of an α,β-dehydroalanine-derived aminonitrile moiety when sodium hydroxide was used as the base with a longer reaction time. Therefore, ester hydrolysis was carried out by employing lithium hydroxide in an equimolar amount and restricting the reaction time and temperature to 5 min and 4 °C, respectively, which preserved the structural integrity of the P1 side chain.

### Stereoconservative Synthesis of Dipeptide Alkynes via Garner’s Aldehyde (compounds **2a**–**m**)

**Scheme 6:**
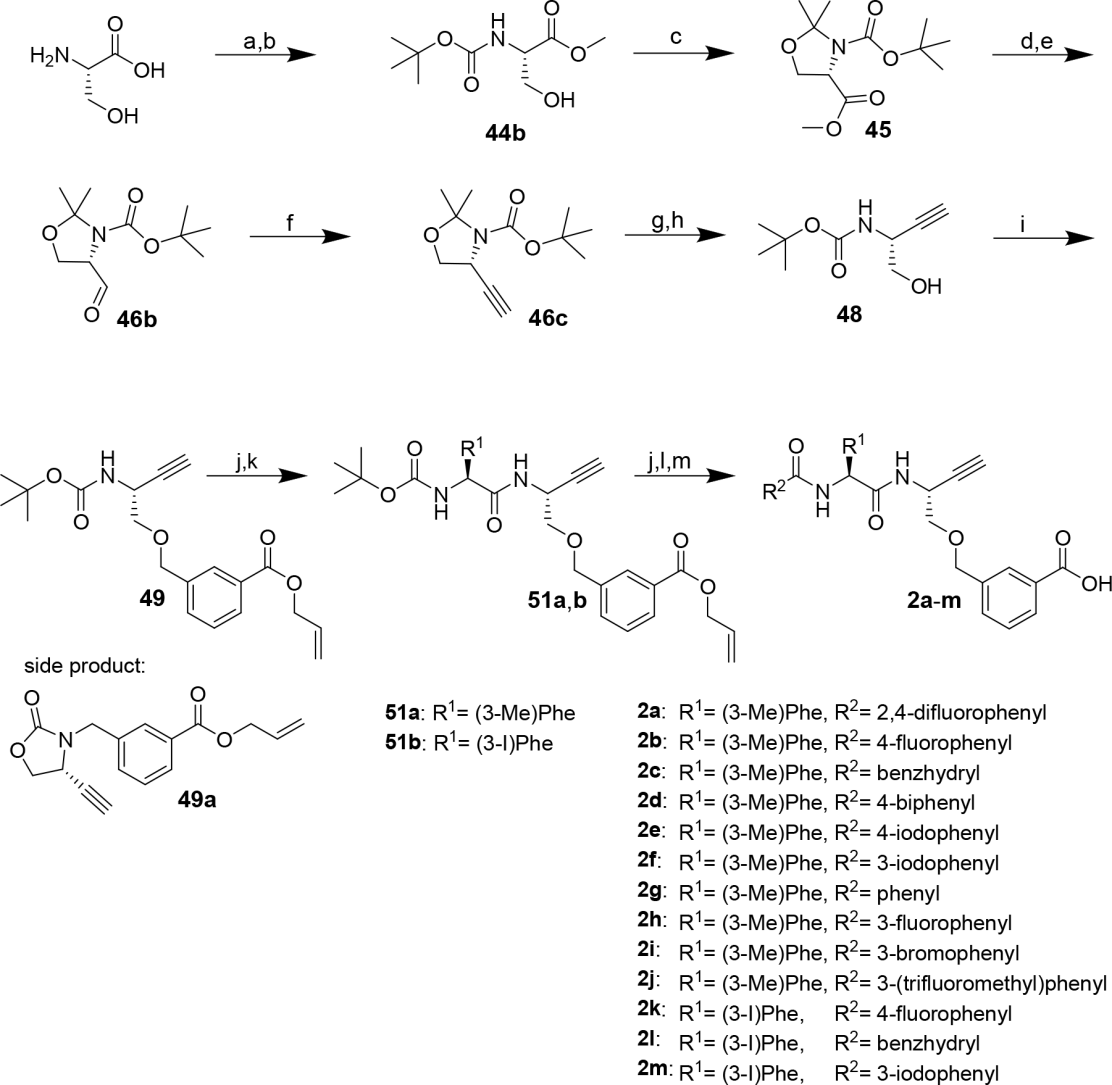
Synthesis of Dipeptide Alkynes **2a**–**m** Reagents and conditions: (a) acetyl chloride, MeOH, reflux, 2 h; (b) Boc_2_O, TEA, THF, 0 °C, 45 min, rt, overnight; (c) 2,2-dimethoxypropane, BF_3_·OEt_2_, acetone, 3 h; (d) LiAlH_4_, THF, 45 min; (e) oxalyl chloride, DIPEA, DMSO, CH_2_Cl_2_, −78 °C, 80 min, 0 °C, 10 min; (f) dimethyl (1-diazo-2-oxopropyl)phosphonate, K_2_CO_3_, MeOH, 0 °C, 4 h; (g) HCl (4 M)/MeOH (3:5), reflux, 1 h; (h) Boc_2_O, TEA, THF, 0 °C, 45 min, rt, overnight; (i) NaH, DMF, 0 °C, 5 min, rt, 1.5 h; (j) TFA/CH_2_Cl_2_ (1:1), 2 h; (k) *N*-Boc-amino acid, DIPEA, PyBOP, THF, 3 h; (l) acyl chloride, TEA, CH_2_Cl_2_, 2 h, or carboxylic acid, DIPEA, PyBOP, THF, 3 h; (m) Pd(PPh_3_)_4_, morpholine, CH_2_Cl_2_, 30 min.

As the tri- and tetramethylene linkers at P1 were revealed to be detrimental for the inhibitory activity, we aimed to synthesize stereochemically pure lead dipeptide alkynes **2a** and **2b** as shown in Figure 1. For this purpose, the synthesis route via Garner’s aldehyde, which represents an established synthon for the stereoconservative synthesis of chiral compounds, was implemented (Scheme 6).^97−99^

The route started from l-serine, which was transformed into Boc-Ser-OMe (**44b**). Compound **44b** was reacted with 2,2-dimethoxypropane to obtain oxazolidine derivative **45**. Similar to the synthesis of the diastereomeric mixture of dipeptide alkynes **2a** and **18**, the ethinyl moiety was introduced via stepwise reduction and partial reoxidation of the methyl carboxylate followed by the homologation reaction. For this purpose, **45** was reduced to primary alcohol **46a** using LiAlH_4_ and then subjected to Swern oxidation to obtain Garner’s aldehyde **46b**. The use of the sterically demanding Hünig’s base (DIPEA) accounts for the suppression of racemization under the conditions of the oxidation step.^98^ To prove the stereochemical integrity of **46b**, a sample was reduced to the corresponding primary alcohol with sodium borohydride. The resulting primary alcohol was acylated with (*R*)-Mosher’s acid-derived chloride [(*S*)-3,3,3-trifluoro-2-methoxy-2-phenylpropanoyl chloride]. ^1^H and ^19^F NMR analysis did not reveal signals of the undesired diastereomer. This result was controlled by subjecting racemic Garner’s aldehyde (obtained from racemic serine) to the identical synthetic transformation and by esterifying Garner’s alcohol as obtained along the route to *S*-configured Garner’s aldehyde with (*R*)-Mosher’s acid. The corresponding NMR spectra and a detailed discussion are included in the Supporting Information.

For the conversion of **46b** with the Bestmann–Ohira reagent, the reaction time was reduced to 3 h compared to that for the synthesis of the mixture of epimeric dipeptide alkynes **2a** and **18** (12 h). An optical rotation that matched the reported values indicated the stereochemical purity of the obtained cyclic alkyne **46c** was determined to verify stereochemical purity. Even though the electronic situation of the serine-derived C atom in Garner’s aldehyde is similar to that in open-chain **12**, racemization by deprotonation is disfavored by the ring strain of the corresponding cyclic enolate. As the acidic reaction conditions applied for cleavage of the oxazolidine ring cause concomitant cleavage of the Boc group, the protecting group had to be reintroduced to obtain Boc-protected serine-derived alkyne **48**, which was obtained in form of single crystals suitable for structural analysis by X-ray diffraction.

**Figure 5:**
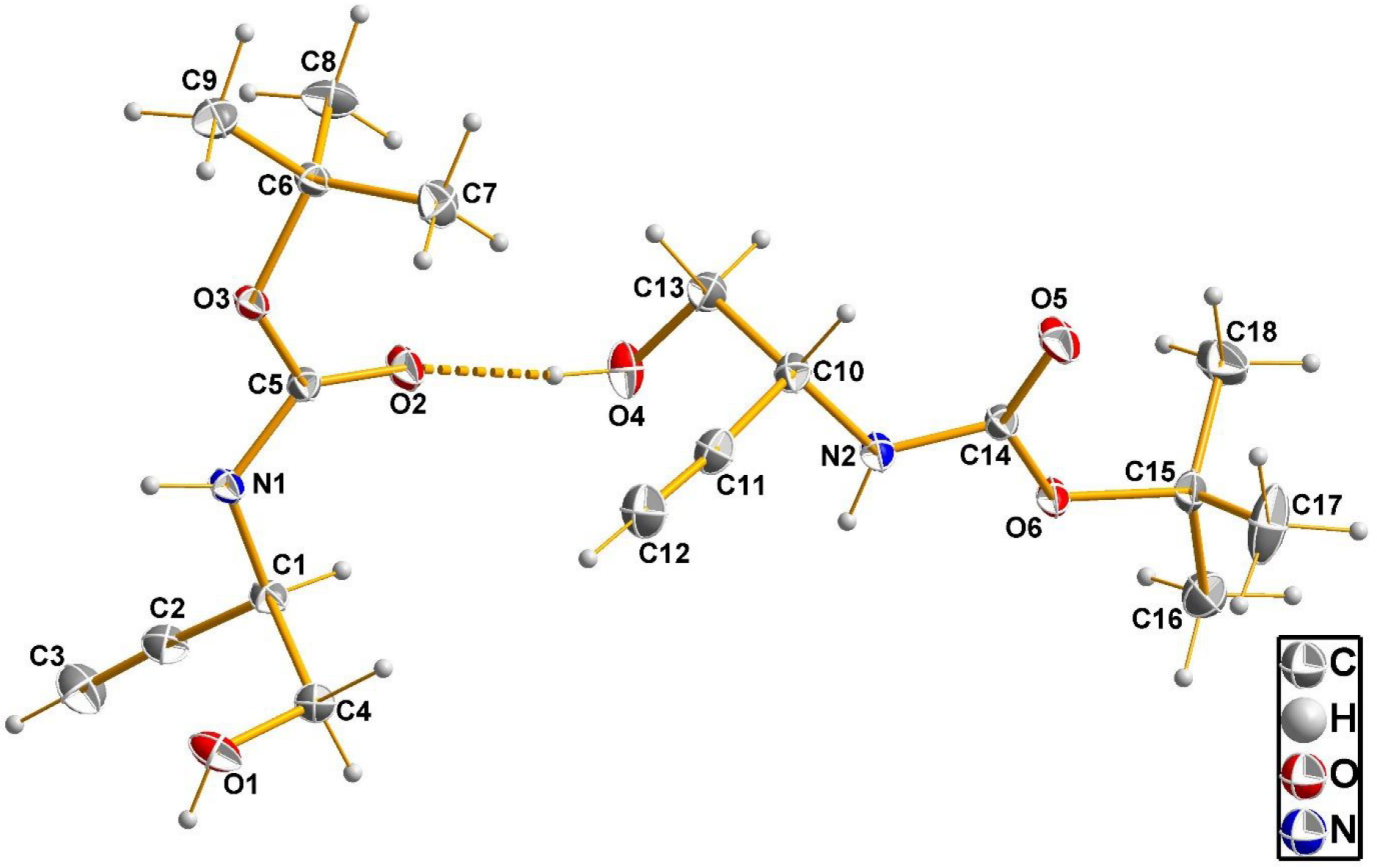
Structure of the two symmetry-independent molecules of compound **48** as observed in the crystalline state by X-ray diffraction analysis with the atom labeling scheme. Thermal displacement ellipsoids are shown at the 50% probability level. The hydrogen bond between the two molecules is shown as a dashed line.

Compound **48** crystallizes in the monoclinic crystal system with acentric space group *P*2_1_. The asymmetric unit contains two symmetry-independent molecules whose structures are shown in Figure 5. Both chiral C atoms, C1 and C10, have the *R* configuration, proving that crystals of **48** are enantiomerically pure and thereby their configurational homogeneity. This result unambiguously confirms the conservation of configuration during the homologation step. In the crystal, the molecules are interconnected by hydrogen bonds. The shortest one, O4–H···O2′ (′: −*x* + 1, *y* – ^1^/_2_, −*z* + 1), with a donor–acceptor (D···A) distance of 2.753(1) Å is shown in Figure 5. Further intermolecular contacts, which also concern the (alkyne)C–H bond,^100^ are highlighted and discussed in Figure S64.

When **48** was subjected to O-alkylation with compound **3b**, adding the alkylating agent immediately after NaH proved to be critical for obtaining the desired product **49** (see the Supporting Information for details).

After the removal of the Boc group of **49**, 3-methylphenylalanine was introduced at P2 as described above followed by N-terminal deprotection and (2,)4-(di)fluorobenzoylation. Pd(0)-catalyzed allyl ester cleavage as described and subsequent HPLC purification furnished the final dipeptide-derived alkynes **2a** and **2b**, in overall yields of 4% and 8%, respectively. The diastereomeric purities of the final compounds were >99% (**2a**) and >94% (**2b**) as determined on the basis of their ^1^H NMR spectra. Notwithstanding the additional reaction steps, the overall yield of **2a**, 4% over 14 steps, was significantly higher than that achieved for the epimerized dipeptide-derived alkyne **18** (>1% over 11 steps). Moreover, the yield achieved via Garner’s aldehyde was also higher than the yield of dipeptide alkyne **28** containing (4-carboxy-1*H*-1,2,3-triazol-1-yl)-l-norvaline at P1, which was 1% over 10 steps, as mentioned above. Therefore, the synthetic route via Garner’s aldehyde was used to synthesize dipeptide-derived alkynes **2c**–**m**, which were obtained in diastereomeric purities in the range of 94–99%. Yields for alkynes containing 3-iodophenylalanine at P2 were diminished compared to those of compounds with 3-methylphenylalanine (52% over the last three steps for **2b** compared to 7% for **2k**), which is attributed to Pd-mediated side reactions at the iodophenyl moiety during allyl ester cleavage. Detailed procedures and analytical data of these compounds are included in the Supporting Information.

### Synthesis of Dipeptide Nitriles and Alkynes with Glycine at P1 (compounds **56a**–**f** and **62**)

**Scheme 7:**
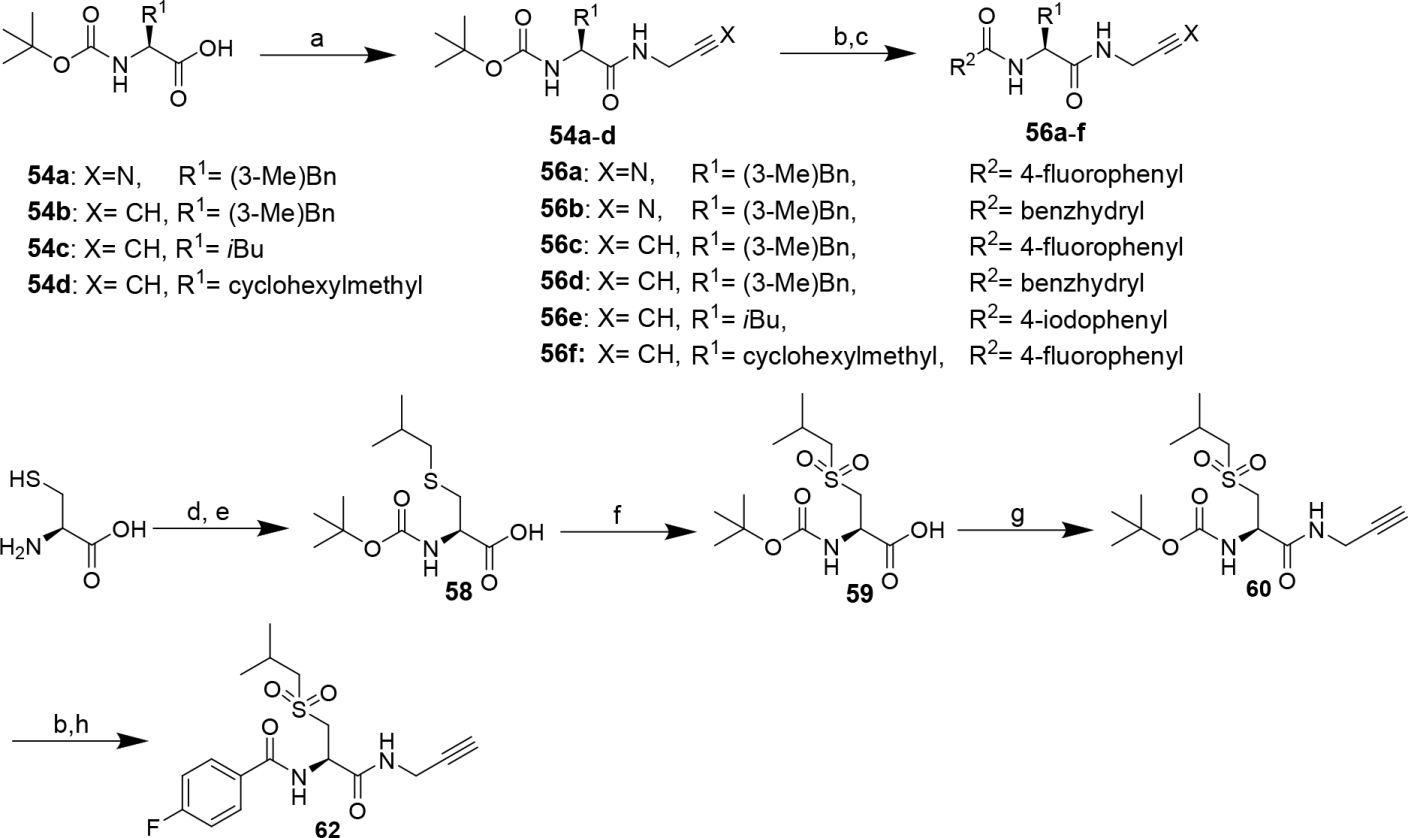
Synthesis of Nitrile- and Alkyne-Based Dipeptide Alkynes **56a**–**f** and **62**, respectively Reagents and conditions: (a) propargylamine or aminoacetonitrile (in THF/H_2_O/NaOH), iBCF, NMM, THF, −25 °C, 10 min, rt, 30 min; (b) TFA/CH_2_Cl_2_ (1:1), 2 h; (c) acidic chloride, TEA, CH_2_Cl_2_, 2 h, or carboxylic acid, DIPEA, PyBOP, THF, 2 h; procedure following^102^ (d) isobutyl bromide, tetrabutylammonium iodide, EtOH/3 M NaOH (1:1), 3 days; (e) Boc_2_O, 1 day; (f) KMnO_4_, acetic acid, 2.5 h; (g) propargylamine, iBCF, NMM, THF, −25 °C, 10 min, rt, 30 min; (h) 4-fluorobenzoyl chloride, TEA, CH_2_Cl_2_, 2 h.

Facing the complexity of the synthesis of amino acid-derived amino alkynes reported above, we envisioned the preparation of selected amino acid *N*-propargylamides as dipeptide alkynes bearing Gly at P1 on the basis of potent Gly-containing dipeptide nitriles. To accomplish this, the particular P2 amino acids were subjected to amide bond coupling with aminoacetonitrile^95^ or propargylamine via the mixed anhydride method^101^ to obtain the corresponding Boc-protected dipeptide-derived nitriles and alkynes, respectively (Scheme 7). For dipeptide alkyne **62**, Boc-protected isobutyl sulfonylalanine (**59**) was prepared prior to amide bond formation.^102^ Functionalization with the N-terminal P3 capping group by deprotection and acylation furnished the final inhibitor compounds. As mentioned above, Boc removal at dipeptide nitriles was complicated by the acid-induced formation of side products. Dipeptide nitriles **56a** and **56b** were obtained in overall yields of 26–88%. The yields of dipeptide alkynes **56c**–**f** were in the range of 17–88% over three steps. Detailed procedures for synthesis and analytical data of compounds are given in the Supporting Information.

### Kinetic Characterization of Dipeptide Nitrile/Alkyne Pairs **1a**/**2a** and **1b**/**2b**

#### Fluorimetric Assay and Elaboration of Inhibition Mode

To determine the inhibitory potency of the synthesized inhibitors against cathepsin B, a fluorimetric enzyme assay based on the procedures and conditions described by Schmitz et al. was implemented.^95^ As species-dependent differences between murine and human cathepsins can lead to varying inhibition constants,^103,104^ human cathepsin B was used. The enzyme stability was verified using the Selwyn test,^105^ and proof of inhibitor solubility was obtained by recording ultraviolet (UV)–visible absorption up to 600 nm for all used inhibitor concentrations (detailed description in the Supporting Information). The fluorogenic standard substrate Z-RR-AMC, which releases fluorescent 7-amino-4-methylcoumarin (AMC) upon enzyme-catalyzed hydrolysis,^106^ was used,^107,108^ and the *K*_m_ value determined prior to the inhibition experiments [*K*_m,Z-RR-AMC pH 6.0_ = 302 μM (detailed description in the Supporting Information)]. With respect to the intended application of targeting tumor-associated cathepsins, inhibitory potencies of the synthesized dipeptide nitriles and alkynes were determined at pH 6.0, which resembles the slightly acidic tumor microenvironment.^109^

#### Inhibition Kinetics of Dipeptide Nitriles **1a** and **1b** with Respect to Cathepsin B

First, the inhibitory activity of nitrile-based lead compounds **1a** and **1b** was investigated. Therefore, the cathepsin B-catalyzed hydrolysis of Z-RR-AMC was monitored in the presence of six different concentrations of **1a**.

**Figure 6:**
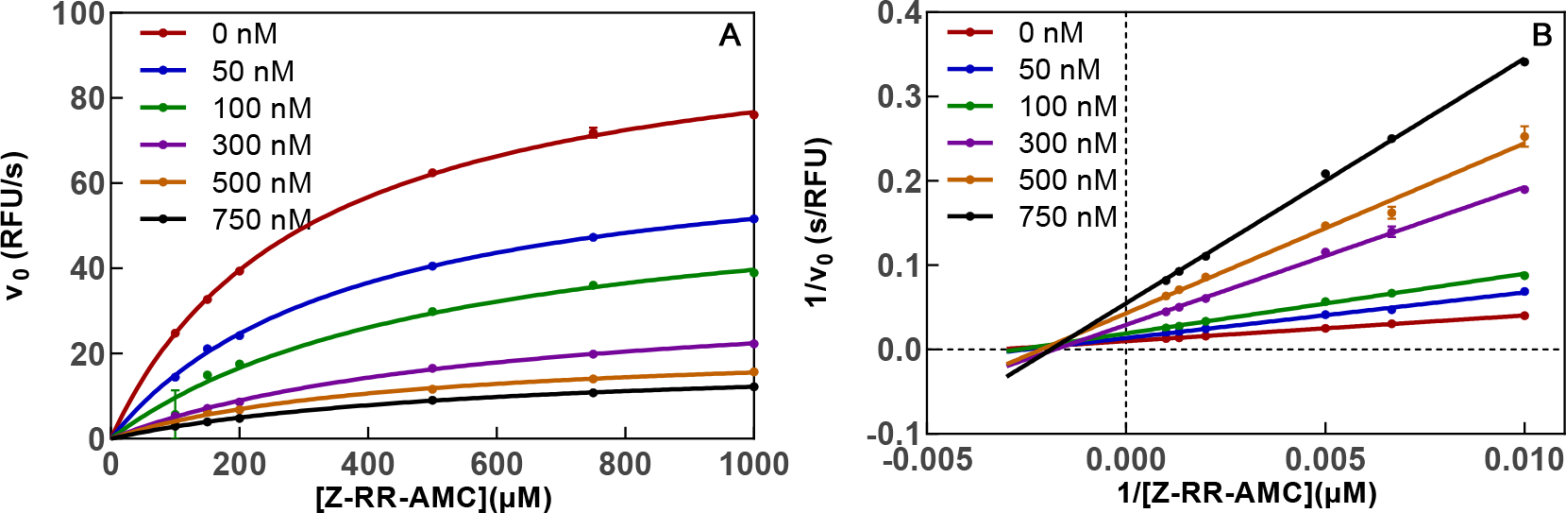
Determination of the type of inhibition of cathepsin B by dipeptide nitrile **1a**. (A) Initial rates of substrate turnover as a function of substrate concentration (*x* axis) and inhibitor concentration (legend). (B) Double-reciprocal plot of 1/*v*_i_ vs 1/[S] for different inhibitor concentrations (transformed data set identical to that of panel A). The lines intersect in the fourth quadrant, which allows the characterization of **1a** as a noncompetitive inhibitor with an α value of >1. Measurements were performed as duplicate determinations in assay buffer (pH 6.0) containing 200 μM Z-RR-AMC, 25 ng/mL cathepsin B, and 1.5% DMSO. Measured values ± SEM are shown.

Substrate conversion by cathepsin B in the presence of **1a** resulted in linear substrate conversion curves at all inhibitor concentrations (see Figure S41A). This observation matches the expectations, because peptidic nitriles commonly exhibit covalent-reversible inhibition in fast binding equilibria.^72,86^ Reversibility was furthermore verified in a jump-dilution experiment (described below). The IC_50_ value obtained by analyzing the plots of initial substrate conversion velocities, *v*_i_, against inhibitor concentrations, [I], was transformed into the dissociation constant of the enzyme–inhibitor complex, *K*_i_, under the assumption of competitive inhibition using the Cheng–Prusoff equation (see the Experimental Section).^110^ In particular, an IC_50_ value of 0.18 μM was determined for nitrile **1a**, from which a *K*_i_ of 0.11 μM was calculated. Greenspan et al. reported an IC_50_ of 6.8 nM,^86^ which, however, cannot be directly compared because different assay conditions were used and the particular *K*_m_ was not stated. Therefore, the *K*_i_ of **1a** was determined at varying substrate concentrations without bias toward the inhibition mechanism (Figure 6). The location of the intersection of the set of lines in the Lineweaver–Burk replot in the fourth quadrant clearly above the *x* axis indicates mixed-type inhibition. Analysis by global nonlinear regression according to eq IX of the Supporting Information has revealed a true *K*_i_ of 64 nM and an α value of 2.5, which indicates that the binding of the inhibitor does not occur exclusively in the substrate binding site but also to the enzyme–substrate complex with 2.5-fold reduced affinity compared to that for the free enzyme. Considering the presence of the thiol-reactive nitrile warhead, this result was unexpected. The inhibition constant in the double-digit nanomolar range confirms potent inhibition of cathepsin B by dipeptide nitrile **1a**, even though its inhibitory activity is lower than suggested by the IC_50_ value reported by Greenspan et al. Replacement of the difluorinated benzoyl moiety in **1a** with the corresponding *p*-monofluorinated residue as realized in nitrile **1b** even increased the inhibitory activity against cathepsin B slightly (Table 1), which represents a promising result in terms of labeling with fluorine-18. The confirmed inhibitory activity of dipeptide-derived nitriles **1a** and **1b** with respect to cathepsin B in the low nanomolar range encouraged their transformation into alkyne-based inhibitors **2a** and **2b**, respectively.

**Table 1:**
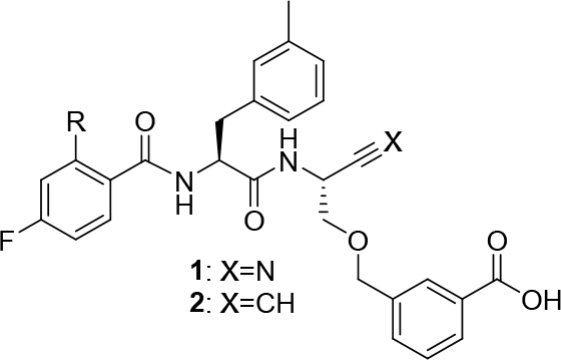
Kinetic Parameters of Dipeptide Nitriles **1a** and **1b** and Dipeptide Alkynes **2a** and **2b** with Respect to Cathepsins B, S, L, and K^a^

compound	R	CatB	CatS	CatL	CatK
*K*_i_ (μM)					
**1a**	F	0.109 ± 0.023	0.406 ± 0.040	0.661 ± 0.011	25.94 ± 0.58
**1b**	H	0.042 ± 0.003	0.046 ± 0.004	0.220 ± 0.004	8.81 ± 0.17
*k*_inact_**/***K*_I_ (M^–1^ s^–1^)					
**2a**	F	22 ± 2	58 ± 1	30 ± 3	3 ± 0,1
**2b**	H	85 ± 3	682 ± 85	281 ± 30	48 ± 5

a Data shown are mean values ± SEM of three experiments, each performed in duplicate.

#### Inhibition Kinetics of Dipeptide Alkynes **2a** and **2b** with Respect to Cathepsin B

**Figure 7:**
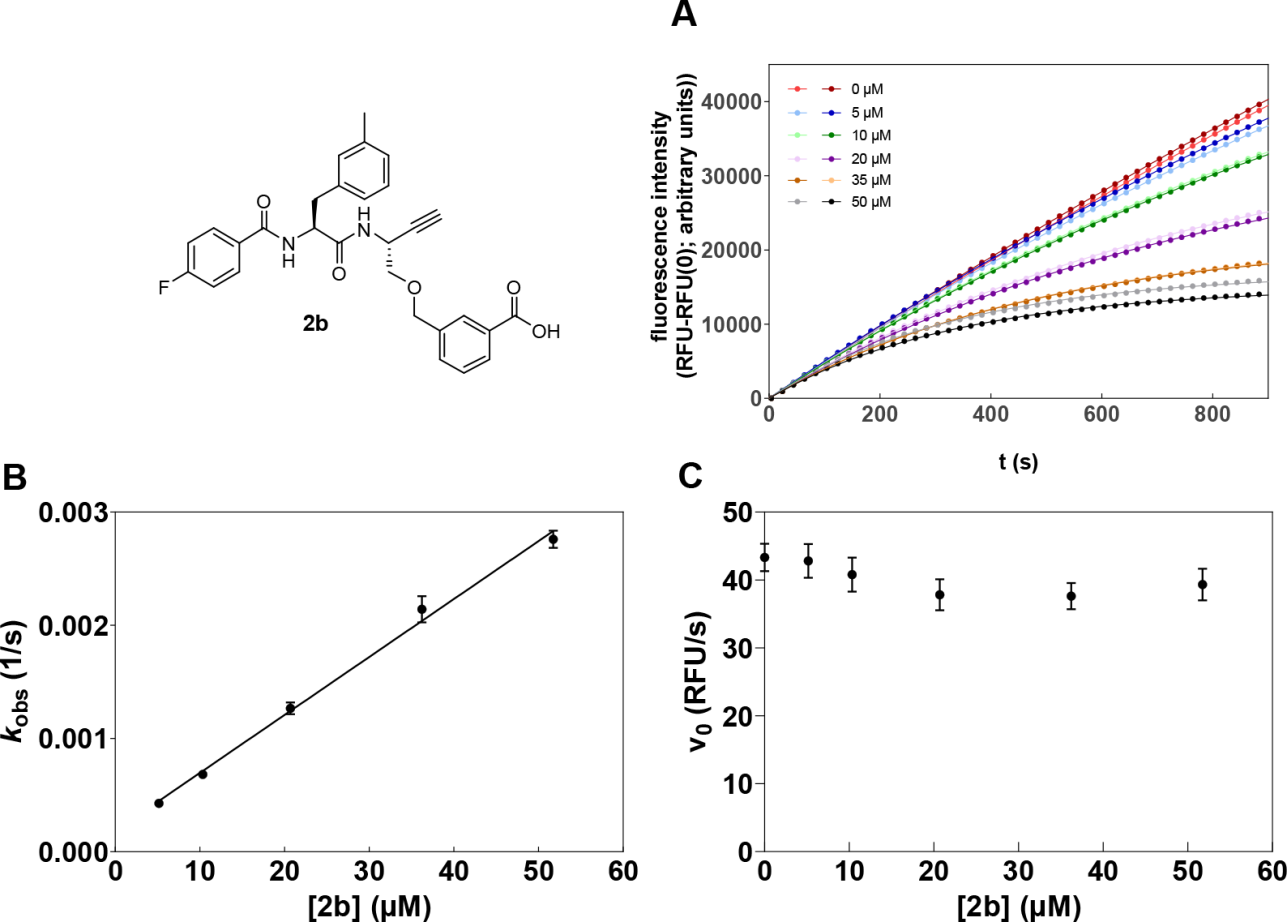
Inhibition of cathepsin B by dipeptide alkyne **2b**. (A) Turnover of Z-RR-AMC by cathepsin B in the presence of increasing concentrations of dipeptide alkyne **2b**. (B and C) Replots of pseudo-first-order rate constants, *k*_obs_, and initial velocities, *v*_0_, respectively, vs inhibitor concentration.

Investigation of the inhibitory activity of alkyne **2a** by monitoring the cathepsin B-catalyzed substrate conversion revealed convex progression curves of increasing curvature with higher inhibitor concentrations, which provides strong evidence of time-dependent irreversible inhibition (Figure 7). Determining the *k*_obs_ values from the product-release curves (eq IV in the Experimental Section) and replotting these values against the inhibitor concentration allowed for the calculation of the second-order rate constant for enzyme inactivation, *k*_inact_/*K*_I_ (eqs V–VII in the Experimental Section), which is the most meaningful parameter for reporting the potency of irreversible inhibitors. Higher values of *k*_inact_/*K*_I_ indicate higher inhibitory potency.^82,111^ In this way, a second-order inactivation constant of 22 M^–1^ s^–1^ for dipeptide alkyne **2a** could be calculated from the determined *k*_obs_ values. In accordance with the slightly higher inhibitory potency of nitrile **1b**, a higher inactivation constant of 85 M^–1^ s^–1^ was obtained for alkyne **2b**.

**Figure 8:**
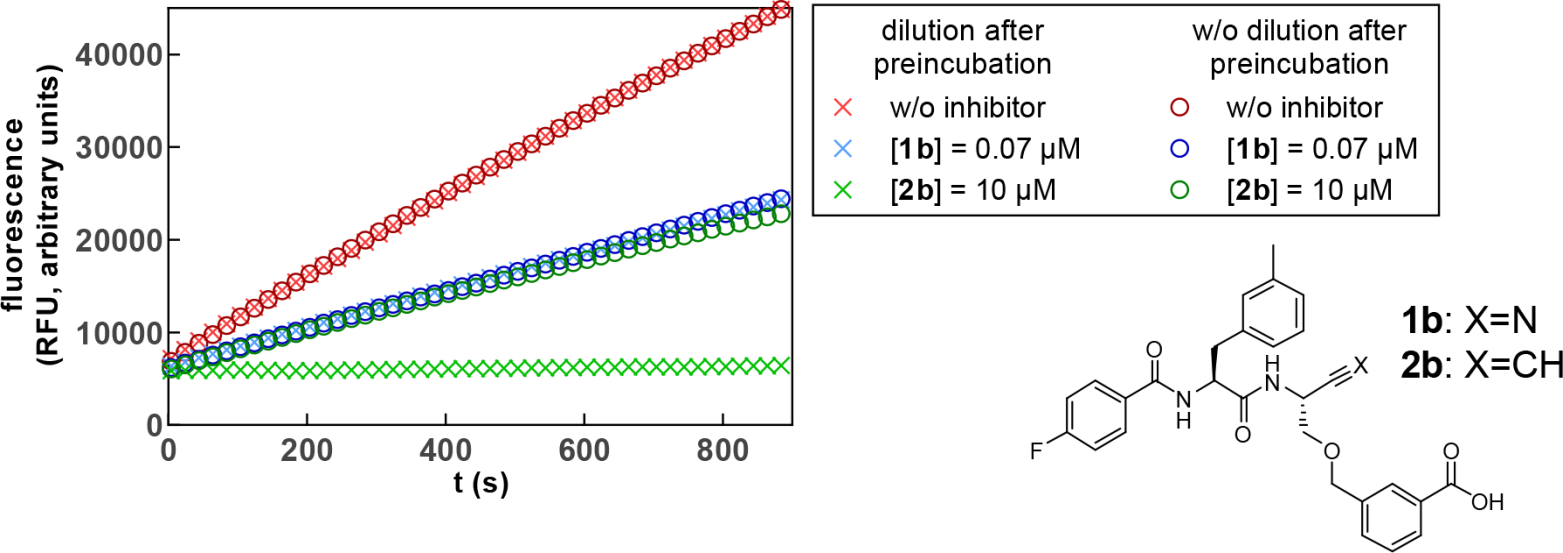
Jump-dilution inhibition experiment for nitrile **1b** and alkyne **2b** with cathepsin B to prove the irreversible inhibition by **2b**. The inhibitors were preincubated in a volume of 30 or 180 μL in the presence of enzyme as schematically shown in Figure S46. The highly concentrated solution was diluted to 180 μL immediately before measurement. Subsequently, the reaction was started by substrate addition so that equal enzyme and inhibitor concentrations were achieved at the start of each measurement. The measurement was performed as a duplicate determination in assay buffer (pH 6.0) containing 200 μM Z-RR-AMC, 25 ng/mL cathepsin B, and 1.5% DMSO. The concentrations indicated refer to the final concentrations during the measurement after dilution.

To verify the reversibility of the nitrile-based inhibitors and the irreversible inhibition mode of the dipeptide alkynes, a jump-dilution experiment^112^ was exemplarily performed for dipeptide nitrile **1b** and alkyne **2b** with cathepsin B. For this purpose, the enzyme was incubated for 30 min in the presence of the inhibitor at a given concentration or in a small volume and a higher concentration followed by dilution before substrate addition. For fast-reversible inhibitors, the new equilibrium forms directly after dilution, while an irreversible inhibitor remains at the active site, resulting in less substrate turnover.^111^ The jump-dilution experiment for **1b** (0.07 μM) and **2b** (10 μM) is shown in Figure 8. As the diverse inhibition modes lead to virtually different inhibition efficiencies, inhibitor concentrations resulting in comparable substrate conversion rates for the undiluted sample were chosen for better comparability of the observed effects. Nitrile **1b** (data colored blue) shows instantaneous and complete readjustment of the equilibrium after dilution resulting in overlapping curves. This proves the fast-reversible nature of the inhibition mechanism. Dipeptide alkyne **2b** (data colored green) shows inhibition efficiency comparable to that of the nitrile for incubation at the lower inhibitor concentration. For incubation in the presence of a higher concentration followed by dilution, complete inhibition is observed. No recovery of enzyme activity could be observed over a time course of 15 min. This result unequivocally demonstrates the irreversibility of the inhibition of cysteine cathepsins by peptidic alkynes. Verification of irreversibility toward other cathepsins was exemplarily performed for the inhibition of cathepsin S by alkyne **2b** (Figure S47).

#### Selectivity of **1a**, **1b**, **2a**, and **2b** for Cathepsin B

Sufficient selectivity should be achieved for the specific detection of the different cysteine cathepsins. For this purpose, compounds **1a**, **1b**, **2a**, and **2b** were tested for their inhibitory effect against the oncologically relevant cathepsins S, L, and K.^42^ The fluorimetric assays were established on the basis of the procedures published by Schmitz et al.^95^ Again, the stability of these cysteine cathepsins under assay conditions was tested using the Selwyn test^105^ (detailed information in the Supporting Information). Analysis of assay data was performed as described above for cathepsin B.

The calculated inhibition parameters for compounds **1a**, **1b**, **2a**, and **2b** are listed in Table 1. It is worth noting that the inhibitory activity toward all four human cysteine cathepsins could be detected both for the nitrile and for the alkyne derivatives. This demonstrates that dipeptide alkynes can inhibit various cysteine cathepsins, which is known from their nitrile-based counterparts.^113,114^ Remarkably, replacement of the difluorobenzoyl capping group with 4-fluorobenzoyl improves the inhibitory potency toward all four considered cathepsins.

**Figure 9:**
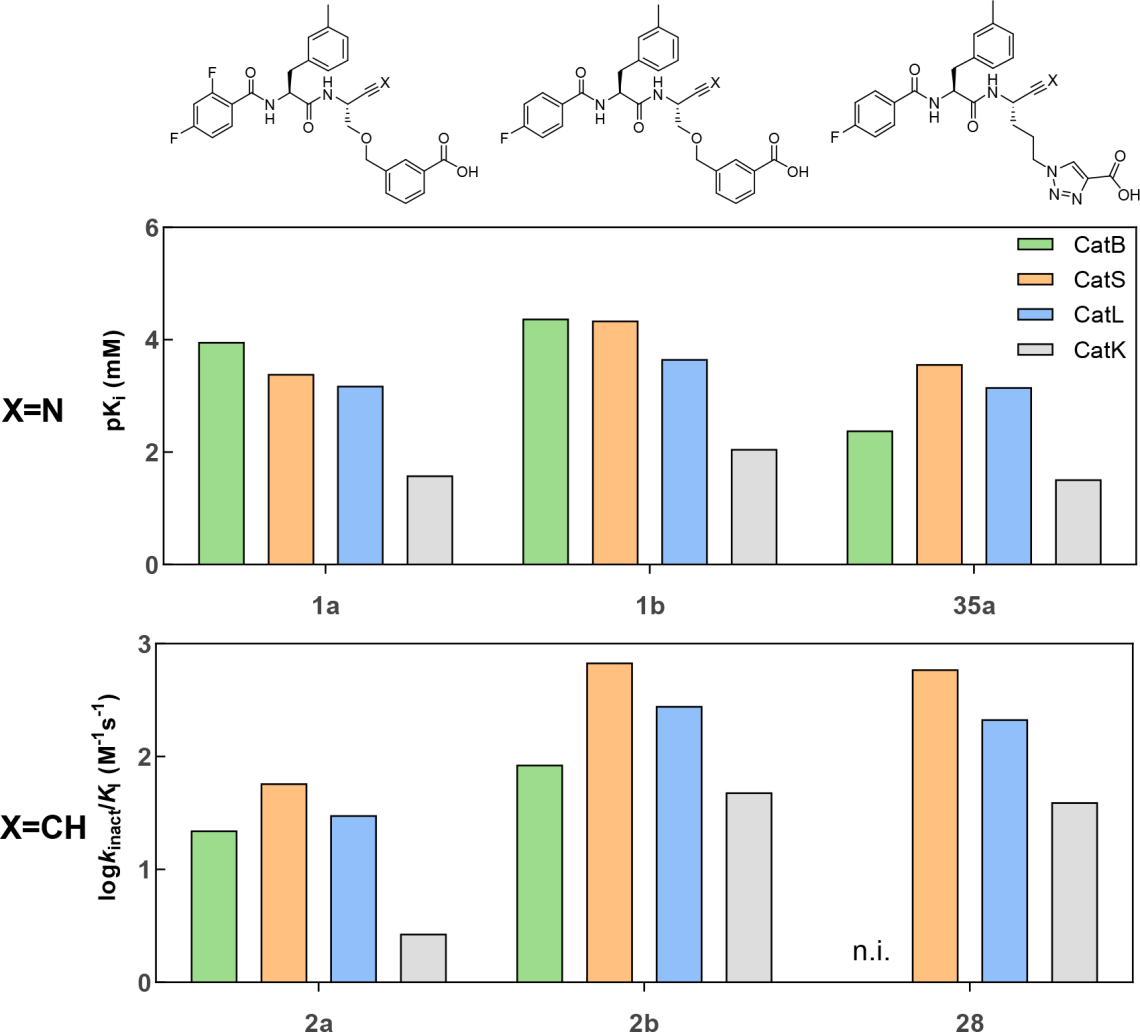
Selectivity profile of stereochemically pure alkyne-based inhibitors **2a**, **2b**, and **28** (bottom) and corresponding nitriles (top). Shown are the negative decadic logarithms of the *K*_i_ values and the decadic logarithm of the inactivation constants *k*_inact_/*K*_I_. Thus, larger values are equivalent to higher inhibition potentials of the compounds. The measurement was performed in three independent experiments (each as a duplicate determination) in assay buffer (pH 6.0) with 1.5% DMSO. n.i. = no inhibition; i.e., no evidence of irreversible inhibition was discernible within the considered time and concentration ranges.

Inhibition was judged selective when the ratio between the equilibrium *K*_i_ values or the second-order inactivation constant *k*_inact_/*K*_I_ toward different cathepsins is >10. Differences by a factor between 5 and 10 are considered as moderate selectivity. Even though the lowest *K*_i_ of **1a** was determined for cathepsin B, 11 nM (Table 1), its selectivity for cathepsin B over cathepsins L and S with a factor of >100 as reported by Greenspan et al. could not be confirmed.^86^ Furthermore, **1b** does not exhibit selectivity for cathepsin B over L and S either, but both compounds were considerably less potent inhibitors for cathepsin K. Dipeptide alkynes **2a** and **2b** preferably inhibit cathepsin S, even if selectivity is not achieved. Considering the identical orbital hybridization for the electrophilic carbon atom and number of non-hydrogen atoms in the warhead, similar selectivity profiles were expected for the dipeptide nitriles and the corresponding alkynes. However, the inhibition profiles of alkynes **2a** and **2b** toward the distinct cysteine cathepsins do not directly reflect that of the corresponding nitriles (Figure 9). Surprisingly, even the weakest observed inhibition of cathepsin K by nitrile **1a** with a *K*_i_ value in the two-digit micromolar range translates into weak, yet detectable, irreversible inhibition of cathepsin K by the analogous alkyne **2a** with a *k*_inact_/*K*_I_ of 3 M^–1^ s^–1^.

### Structure–Activity Relationships

The compelling capability of dipeptide-derived alkynes **2a** and **2b** for irreversible inhibition of cysteine cathepsins encouraged the exploration of further structural variations to increase inhibitory potency and selectivity. The kinetic parameters for all inhibitor compounds with respect to cathepsins B, S, L, and K obtained in the fluorimetric assays are included in Tables S1 and S2.

#### Influence of P1 Substituents

The beneficial influence of *m*-carboxybenzylserine at P1 and derived moieties bearing a free carboxylic group on inhibitory potency against cathepsin B was previously reported for peptidic nitriles, which was attributed to interactions with the two adjacent His residues in the unique occluding loop of this cathepsin.^86,95^ Accordingly, irreversible inhibition was observed for dipeptide alkyne **2b** with respect to cathepsin B. However, inactivation of cathepsins S and L by this alkyne was even faster and inhibitory potency was only slightly lower with cathepsin K. Surprisingly, replacement of the ether linker and phenyl moiety in the P1 side chain of alkyne **2b** with propylene and 1,2,3-triazole, respectively, as realized in compound **28**, abolished the inhibitory activity toward cathepsin B, while this alkyne was still capable of inactivating cathepsins S, L, and K to an extent similar to that of **2b** (Figure 9) with the highest potency against cathepsin S (*k*_inact_/*K*_I_ = 595 M^–1^ s^–1^). In line with these results, the analogous nitrile **35a** exhibits a drastically diminished *K*_i_ value for cathepsin B, while the inhibition of the other three cathepsins was less affected and declined in the following order: S > L > K (Table 2). Given that p*K*_i_(CatB) > p*K*_i_(CatK) for **35a**, the fact that the corresponding alkyne **28** is capable of inactivating cathepsin K in the absence of irreversible inhibition of cathepsin B appears surprising. Such discrepancies were also observed for other dipeptide nitrile/alkyne pairs [IC_50_ > 50 μM (Figure S48)]. Consequently, the binding affinity of nitriles does not directly translate into the kinetics of irreversible inhibition by analogous alkynes, particularly with regard to selectivity profiles. This finding may indicate subtle differences in the structure of the catalytic sites between human cysteine cathepsins, which thereby deal differently with the stabilization of covalent adducts formed by the nucleophilic attack of the active-site thiolate on the C≡N and C≡C bonds. Further studies are required to explore the reason for this phenomenon.

**Table 2:** Inhibition Constants of Dipeptide Nitriles with Varying P1 Side Chains for Cathepsins B, S, L, and K^a^

	*K*_i_ (μM)						
	**56a**	**1b**	**35a**	**35b**	**35c**	**42**	**43**
CatB	1.19(4)	0.042(3)	4.1(1)	4.6(5)	2.34(8)	0.86(3)	1.61(4)
CatS	0.055(5)	0.044(4)	0.279(7)	0.32(2)	0.21(1)	0.110(9)	0.191(2)
CatL	0.049(3)	0.220(4)	0.69(2)	0.54(3)	0.47(2)	0.049(2)	0.52(2)
CatK	2.1(2)	8.8(2)	30(5)	25(2)	26(2)	4.0(1)	19.1(6)

a Data shown are mean values ± SEM of three experiments, each performed in duplicate.

Even though irreversible inhibition of cathepsin B by **28** does not occur, the alkyne interacts weakly reversibly with this enzyme (see Figure S45). The fact that nitrile **35a**, whose inhibition constant for cathepsin B is in the single-digit micromolar range, does not exhibit irreversible inhibition for the analogous alkyne **28** with the same enzyme indicates that only peptidic ligands with sufficient binding affinity translate into irreversible inhibitors upon functionalization with the weakly electrophilic C≡C bond. This reflects the finding that covalent targeting of caspase-1 required long interleukin 1β-derived peptidic recognition sequences of 16–26 amino acids with a C-terminally ethynylated aspartic acid residue. In contrast, the conversion of tetrapeptidic aldehyde Ac-YVAD-ψ[CHO] as a potent reversible inhibitor into the corresponding alkyne did not result in significant inhibition of this cysteine protease.^79^ However, the relation between affinity for reversible binding and strength of irreversible inhibition seems to be complex, as indicated by the inhibitory activities of the nitrile-alkyne pair **1a**/**2a** against cathepsin K discussed above, where irreversible inhibition was detectable for alkyne **2a** despite the even lower binding affinity of nitrile **1a**.

**Figure 10:**
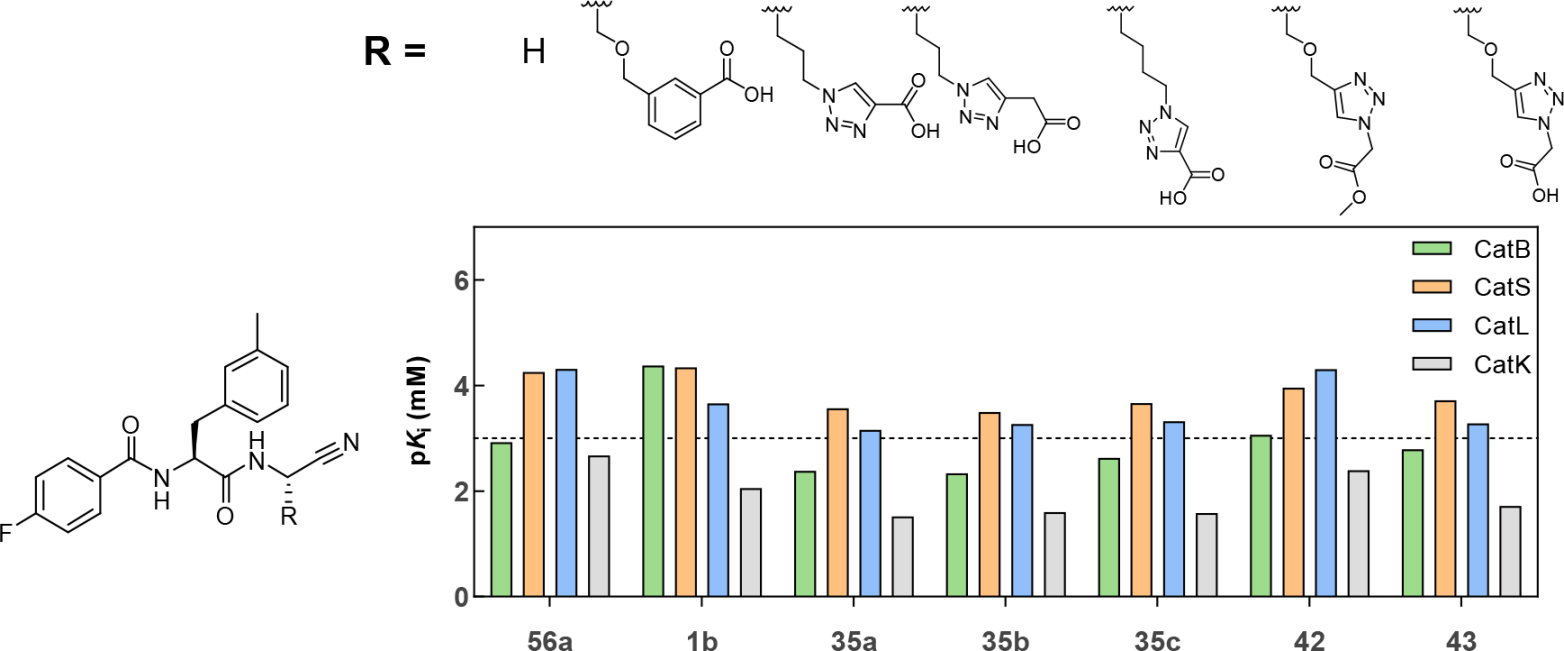
Influence of the side chain at P1 in depicted dipeptide nitriles on their inhibitory selectivity for cathepsins B, S, L, and K. The measurement was performed in three independent experiments (each as a duplicate determination) in assay buffer (pH 6.0) containing 1.5% DMSO.

To obtain more insights into the influence of related P1 moieties on cysteine cathepsin inhibition and to improve the interaction with the enzyme, the linker between the peptidic backbone and the carboxy-functionalized hetarene was modified. As no cathepsin B inhibition was observed for alkyne **28**, modifications were performed on the basis of nitrile **35a**. The *K*_i_ values determined for these compounds for cathepsins B, S, L, and K are included in Table 2, and their structures and selectivity profiles are shown in Figure 10.

All tested dipeptide nitriles showed inhibition of all four cathepsins. The strongest potency toward cathepsin B was obtained for **1b** with *m*-carboxybenzylserine at P1. Despite the similar P1 side-chain architecture, **35a** was most potent against cathepsin S (*K*_i_ = 270 nM) without selectivity over cathepsin L, but the compound was somewhat selective over cathepsin B (15-fold) and clearly selective over cathepsin K (112-fold). Replacement of the benzene ring and the ether side-chain linker in P1 with a 1,2,3-triazole ring and propylene chain, respectively, as realized in dipeptide nitrile **35a**, resulted in reduced inhibitory potency toward all four cathepsins, irrespective of the direct ring attachment of the carboxylic group or spacing of a methylene group between these structural elements (**35a** and **35b** vs **1b**). Extension of the linker or introduction of oxygen (**35c** and **43**) led to slightly increased potency, but the obtained *K*_i_ values were still higher than without a residue at P1 (**56a**). Interestingly, the cathepsin B inhibitory potency of methyl ester **42** was higher than that of the corresponding free acid **43**. Therefore, the interaction of the triazole-bound carboxylic groups in dipeptide nitriles **35a**–**c** and **43** with the His residues in the occluding loop appears unlikely, which can be also concluded from the decreased potency compared to that of **1b**.

Surprisingly, cathepsin S preferred *m*-carboxybenzylserine at P1 similar to cathepsin B despite the absence of the occluding loop in this enzyme. In contrast, a higher inhibitory potency was observed in the absence of a free carboxylic group in the case of cathepsin L (**56a** and **42**). The preference of cathepsin K for Gly at P1, as reported previously,^115^ was reproduced, as the lowest *K*_i_ toward cathepsin K was exhibited by **56a**. Exclusive selectivity toward one of the four cysteine cathepsins was not observed within this series of inhibitors.

In conclusion, the *m*-carboxybenzylserine-derived moiety at P1 was identified to be favorable and critical for inhibition of cathepsin B, in accordance with recent findings.^86,95^ However, exclusive targeting of this enzyme among other cysteine is not conferred by this residue, which applies for both dipeptide nitriles and analogous alkynes.

#### Variation of P3 Substituents

**Figure 11:**
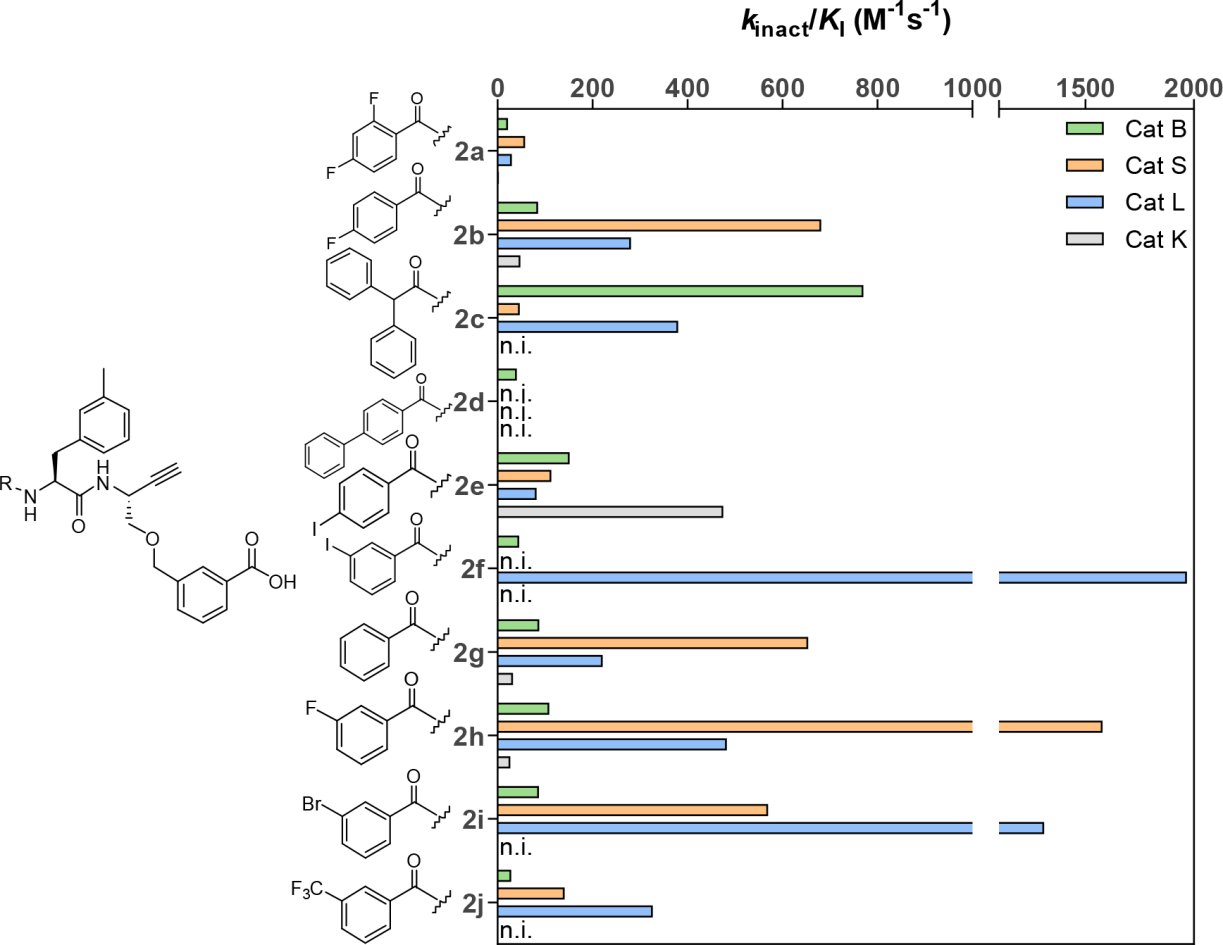
Influence of the P3 substituents in depicted dipeptide alkynes on their selectivity for cathepsins B, S, L, and K. n.i. = no inhibition; i.e., no evidence of irreversible inhibition was discernible within the considered time and concentration ranges.

Due to structural differences between the S3 binding areas of the cathepsins, structural modifications at P3 can be expedient for achieving selectivity.^27,116^ As the inhibition profiles among the investigated cysteine cathepsins did not exactly match for the hitherto investigated nitrile/alkyne pairs, these structural variations were exclusively introduced for dipeptide alkynes with *m*-methylphenylalanine and *m*-carboxybenzylserine at P2 and P1, respectively. The diastereomeric purity was >92% for all compounds. Their *k*_inact_/*K*_I_ values are included in Table 3, and their structures and selectivity profiles are shown in Figure 11.

**Table 3:**
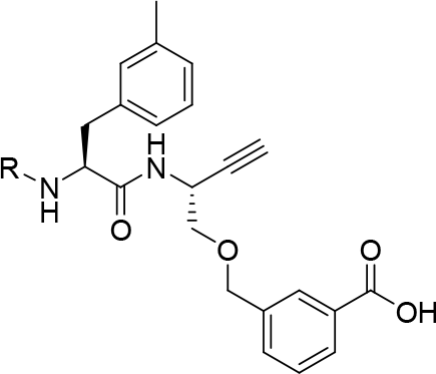
Kinetic Characterization of Dipeptide Alkynes with Variation at P3^a^

		*k*_inact_/*K*_I_ (M^–1^ s^–1^)			
compound	R	CatB	CatS	CatL	CatK
**2a**	2,4-difluorobenzoyl	22(2)	58(1)	30(3)	3(0.1)
**2b**	4-fluorobenzoyl	85(3)	682(85)	281(30)	48(5)
**2c**	diphenylacetyl	771(17)	47(11)	381(43)	n.i.
**2d**	4-phenylbenzoyl	41(1)	n.i.	n.i.	n.i.
**2e**	4-iodobenzoyl	152(2)	113(10)	82(6)	476(65)
**2f**	3-iodobenzoyl	45(1)	n.i.	1968(153)	n.i.
**2g**	benzoyl	88(8)	654(48)	222(6)	33(6)
**2h**	3-fluorobenzoyl	109(5)	1579(114)	483(54)	27(2)
**2i**	3-bromobenzoyl	87(29)	570(47)	1309(12)	n.i.
**2j**	3-trifluoromethylbenzoyl	29(1)	141(18)	327(17)	n.i.

a The measurement was performed in three independent experiments (each as a duplicate determination) in assay buffer (pH 6.0) containing 1.5% DMSO. n.i. = no inhibition; i.e., no evidence of irreversible inhibition was discernible within the considered time and concentration ranges. Data shown are mean values ± SEM of three experiments, each performed in duplicate.

Introduction of diphenylacetyl at P3 (**2c**) led to an increased inhibitory potency against cathepsin B and L compared to that of 4-fluorobenzoyl. In the presence of cathepsin L, the plot of *k*_obs_ versus [I] showed a hyperbolic rather than linear character. This indicates a two-step inhibition mechanism, which means in the case of irreversible inhibition that covalent bond formation occurs in a noncovalent complex that is sufficiently stable to accumulate.^35^ With a value of 771 M^–1^ s^–1^, the strongest cathepsin B inactivation within this series of compounds was observed for diphenylacetyl at P3 (**2c**). Diphenylacetyl was also described by Greenspan et al. as the preferred residue at P3.^86^ However, **2c** did not selectively inhibit cathepsin B over cathepsin L but showed selectivity over cathepsin S and K. Inhibitor **2d** with a 4-phenylbenzoyl residue contained in the cathepsin B selective dipeptide nitrile described by Schmitz et al.^95^ showed weak but selective inhibition of cathepsin B. This compound did not exhibit irreversible inhibition of cathepsin S but behaved like a reversible inhibitor toward this enzyme with a *K*_i_ of 8.21 μM.

Various pharmaceutically relevant radioisotopes of iodine exist such as iodine-123 and iodine-124, which are suitable SPECT and PET nuclides, respectively. Therefore, compounds **2e** and **2f** bearing iodinated benzoyl residues at P3 were synthesized and analyzed for their inhibitory activity. *m*-Iodobenzoylated dipeptide alkyne **2f** was less potent against cathepsin B than its *p*-substituted counterpart **2e**, which is in agreement with the report of Ren et al., who found for a series of azadipeptide nitriles that cathepsin B does not tolerate larger substituents at the *meta* or *ortho* position of the benzoyl residue.^117^ However, iodine as a substituent at the *para* position was preferred over fluorine and phenyl by cathepsin B. For cathepsin S, a high inhibitory potency was observed for the unsubstituted benzoyl residue at P3 (**2g**) within this series of compounds, which, however, was slightly lower than that of 4-fluorobenzoylated **2b**. Cathepsin K preferred 4-iodobenzoyl at P3 (**2e**) with an inactivation constant of 476 M^–1^ s^–1^, which represents the highest value determined for cathepsin K within this study. Neither **2e** nor **2g** was a selective inhibitor for any of the tested cathepsins.

Larger substituents at the *para* position, as contained in compounds **2d** and **2e**, led to a significant decrease in cathepsin L-inhibitory activity. However, relocating the iodine from the *para* to *meta* position (**2f**) resulted in a large increase in inhibitory activity from no detectable inhibition to a *k*_inact_/*K*_I_ of 1968 M^–1^ s^–1^. Notably, compound **2f** was a selective cathepsin L inhibitor with a selectivity factor of >40 over cathepsin B. The compound showed weak and reversible inhibition of cathepsin S (*K*_i_ = 5.64 μM) and was virtually devoid of any influence on cathepsin K activity in the tested concentration range.

Hardegger et al. published a series of dipeptide nitriles with varying residues at P3 and substituted proline residues at P2 and observed strong inhibition for compounds with *para*-substituted phenyl residues. The inhibitory potency increased in the following order: F < Cl < Br < I. Analysis of X-ray co-crystal structures revealed halogen bonding between the halogen σ-hole and the backbone C=O group of Gly61 located in the S3 pocket.^118^ The σ-hole is more pronounced for the heavier halogens, leading to the strongest interaction being that of iodine.^119^ The formation of such halogen bonding could be the reason for the pronounced cathepsin L inactivation by **2f**. Even though Hardegger et al. described *para*-substituted aryl residues, the substituents there should correspond to *meta* substitution in **2f** as the proline residue in these dipeptide nitriles gives them a more bent shape compared to the unrestricted peptidic backbone in the dipeptide alkynes considered herein.

**Figure 12:**
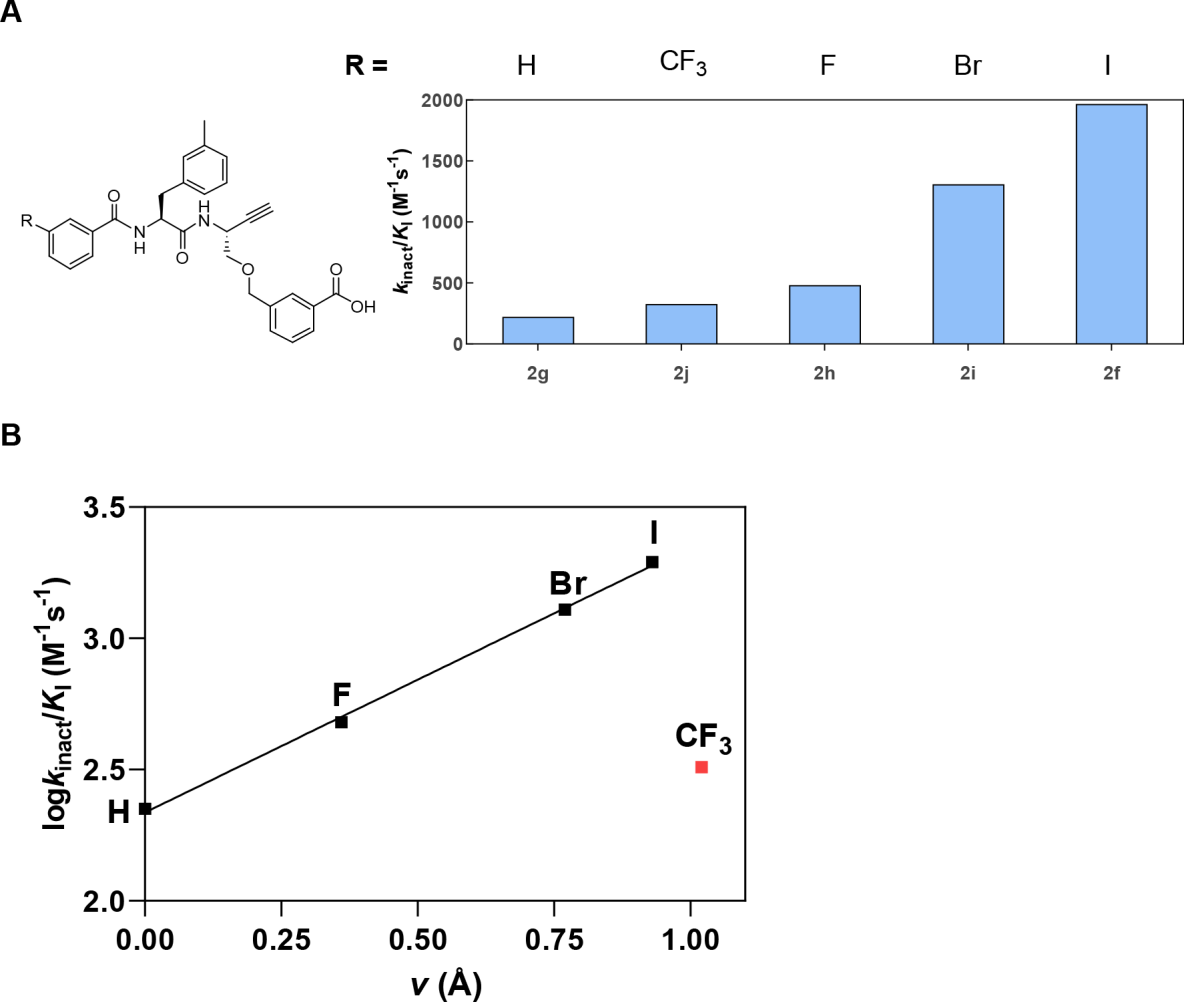
(A) Influence of the substituent at the *meta* position of the benzoyl residue at P3 on the inhibition of cathepsin L. The measurement was performed in three independent experiments (each as a duplicate determination) in assay buffer (pH 6.0) containing 10 μM Z-FR-AMC, 25 ng/mL cathepsin L, and 1.5% DMSO. (B) Relationship between the van der Waals radii of the substituents at the *meta* position of the P3 benzoyl residue and the cathepsin L inactivation constant. The data point for the CF_3_ substituent was excluded from calculating the regression line.

To support the assumed contribution of halogen bonding to inhibition of cathepsin L by compound **2f**, a series of derived dipeptide alkynes with varying substituents at the *meta* position, including other halogens and carbon-based residues, were synthesized and characterized (see the Supporting Information). The results for cathepsin L are shown in Figure 12A. Furthermore, the obtained cathepsin L inactivation constants for this enzyme were plotted against the van der Waals radii of the substituents at the *meta* position to investigate their relationship (Figure 12B).

The determined inactivation constants clearly increase with the radii of the halogen substituents. The logarithmically transformed inactivation constants show a fairly linear correlation with the van der Waals radii. Nevertheless, in spite of a van der Waals radius similar to that of iodine, trifluoromethyl at the *meta* position leads to a significantly lower inhibitory potency. This indicates distinct electrostatic interactions for these two substituents of approximately equal steric demand (**2f** and **2j**). A similar correlation was found by Hardegger et al.^118^ for the dipeptide nitriles mentioned above, for which halogen bonding with the carbonyl oxygen of Gly61 was confirmed by a single crystal structure. The result can be interpreted in favor of halogen bonding, as attractive electrostatic interactions with the positive σ-hole of iodine would not be possible with the trifluoromethyl group, which instead exclusively displays negative partial charge at its surface.

With regard to cathepsins B, S, and K, no evidence of halogen bonding could be discerned from the observed structure–activity relationships within the series of *meta*-substituted benzoyl compounds (see Figure S49). As mentioned above, substituents at the *meta* position are not well tolerated by cathepsin B. This finding is further supported by the fact that the increasing van der Waals radii of the substituent lead to a decreased inhibitory potency. For cathepsin S, introduction of fluorine at the *para* (**2b**; 682 M^–1^ s^–1^) or *meta* position (**2h**; 1579 M^–1^ s^–1^) leads to an increase in inhibitory activity compared to that of benzoyl (**2g**; 654 M^–1^ s^–1^), but larger substituents are not well tolerated. This is in accordance with its rather small S3 binding pocket.^120^ None of the tested components significantly reduced cathepsin K activity. Only **2f** showed selectivity for one of the tested cathepsins.

Upon variation of the residue at P3, the inhibitory potency of the dipeptide alkynes against cathepsin B was significantly improved compared to those of the lead compounds [22 M^–1^ s^–1^ for **2a** and 5 M^–1^ s^–1^ for **2b**, compared to 771 M^–1^ s^–1^ for diphenylacetyl at P3 (**2c**)]. None of the variations led to a cathepsin B-selective inhibitor. However, **2f** constituted a selective cathepsin L inhibitor with a remarkable second-order inactivation constant of 1968 M^–1^ s^–1^, which exceeds the value of 1650 M^–1^ s^–1^ reported by Mons et al. for the inhibition of cathepsin K by the alkyne analogue of Odanacatib.^121^ Compound **2h**, which shows some preference for cathepsin S, though its *k*_inact_/*K*_I_ with respect to cathepsin L is only ∼3-fold lower, might constitute an interesting basis for further modifications toward a selective cathepsin S inhibitor.

#### Combined Variation of P2 and P3 Substituents

Encouraged by the observed beneficial influence of various P3 residues on selectivity, we varied the P2 residue in combination with selected P3 acyl moieties.

Substrate specificity studies showed a preference of cathepsin B for aromatic residues at P2.^122−124^ In diazoketones, E-64 derivatives, and peptidic vinylsulfones, introduction of diiodotyrosine resulted in an enhanced inhibitory potency compared to that with phenylalanine at the corresponding position.^125−128^ Moreover, structurally related monohalogenated phenylalanines such as 3-bromophenylalanine were found to be beneficial for cathepsin B inhibition.^95^ Therefore and because *o*-iodophenyl moieties are prone to biotransformative deiodination,^129^ 3-iodophenylalanine was introduced at P2. With respect to the aspired radiolabeling, 4-fluorobenzoyl was chosen at P3 (**2k**). Additionally, dipeptide alkynes containing diphenylacetyl and 3-iodophenyl at P3 were tested. The inactivation constants determined in the fluorimetric assays are listed in Table 4.

**Table 4:**
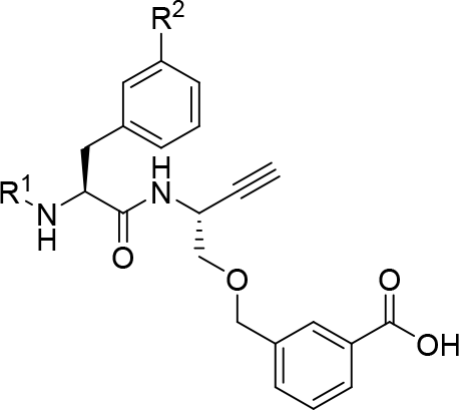
Kinetic Characterization of the Inhibition of Cathepsins B, S, L, and K by Dipeptide Alkynes with 3-Methylphenylalanine or 3-Iodophenylalanine^a^

			*k*_inact_/*K*_I_ (M^–1^ s^–1^)			
compound	R^1^	R^2^	CatB	CatS	CatL	CatK
**2b**	4-fluorobenzoyl	Me	85(3)	682(85)	281(30)	48(5)
**2k**		I	1179(222)	10133(842)	2128(230)	121(5)
**2c**	diphenylacetyl	Me	771(17)	47(11)	381(43)	n.i.
**2l**		I	301(25)	859(83)	552(58)	76(5)
**2f**	3-iodobenzoyl	Me	45(1)	n.i.	1968(153)	n.i.
**2m**		I	225(24)	4368(235)	2876(252)	n.i.

a The measurement was performed in three independent experiments (each as a duplicate determination) in assay buffer (pH 6.0) containing 1.5% DMSO. n.i. = no inhibition; i.e., no evidence of irreversible inhibition was discernible within the considered time and concentration ranges. Data shown are mean values ± SEM of three experiments, each performed in duplicate.

For halogen-substituted benzoyl residues, the introduction of 3-iodophenyl at P2 resulted in a significant increase in cathepsin B inhibitory activity compared to that with 3-methylphenylalanine at P2. The *k*_inact_/*K*_I_ of 1179 M^–1^ s^–1^ determined for dipeptide alkyne **2k** represented the highest cathepsin B inactivation constant determined in this study. Interestingly, this tendency was not observed with diphenylacetyl at P2. For all inhibitors listed in Table 4, cathepsins S and L significantly preferred 3-iodophenylalanine over 3-methylphenylalanine, with the effect being more pronounced for cathepsin S. This leads to the loss of the cathepsin L selectivity of **2f** after introduction of 3-iodophenylalanine at P2 regardless of the increased cathepsin L inhibitory potency (**2f** compared to **2m**). For lead compound **2b**, replacement of the P2 residue with 3-iodophenylalanine (**2k**) led to increases in inhibitory potency of 8- and 15-fold for cathepsins L and S, respectively. Considering the low electrophilicity of the C≡C bond discussed in the Introduction, the obtained cathepsin S inactivation constant of 10133 M^–1^ s^–1^ is in the range of that reported by Giordano et al. for inactivation of cathepsin B by an intrinsically more reactive epoxysuccinyl peptide E-64-c analogue (12300 M^–1^ s^–1^)^127^ and only 1 order of magnitude below the cathepsin B inactivation constant of the broad-band cysteine protease-inhibiting epoxysuccinyl peptide E-64 (118333 M^–1^ s^–1^).^121^ Dipeptide alkyne **2k** showed moderate selectivity for cathepsin S over cathepsin L (5-fold) and cathepsin B (9-fold) and high selectivity over cathepsin K (84-fold). This demonstrated the high potential of the dipeptide alkynes as irreversible cathepsin inhibitors.

#### Inhibitors with Gly at P1

**Figure 13:**
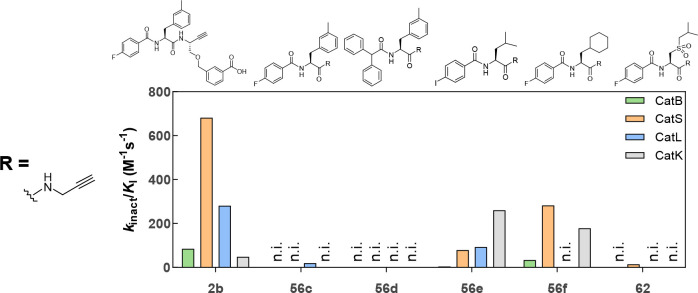
Selectivity profile of dipeptide alkynes with a glycine-derived moiety at P1. The lead compound **2b** is included for comparison. The measurement was performed in three independent experiments (each as a duplicate determination) in assay buffer (pH 6.0) containing 1.5% DMSO. n.i. = no inhibition; i.e., no evidence of irreversible inhibition was discernible within the considered time and concentration ranges.

Several potent, nitrile-based cathepsin inhibitors with a glycine-derived residue at P1 were reported,^72,130^ and their alkyne analogues can be synthesized in fewer steps, compared to the route via Garner’s aldehyde. Table 2 compares dipeptide nitriles with different residues at P1. For cathepsins S, L, and K, the highest inhibitory potency was observed for glycine at P1. Therefore, different dipeptide alkynes with glycine-derived propargylamine at P1 were synthesized. The determined inactivation constants of these compounds are listed in Table 5, and their structures and selectivity profiles are shown in Figure 13.

**Table 5:** Inhibitory Activity of Dipeptide Alkynes Containing a Glycine-Derived P1 Moiety with Respect to Cathepsins B, L, S, and K^a^

	*k*_inact_/*K*_I_ (M^–1^ s^–1^)					
	**2b**	**56c**	**56d**	**56e**	**56f**	**62**
CatB	85(3)	n.i.	n.i.	4(2)	34(3)	n.i.
CatS	682(85)	n.i.	n.i.	n.i.	293(36)	14(1)
CatL	281(30)	19(3)	n.i.	93(11)	n.i.	n.i.
CatK	48(5)	n.i.	n.i.	260(29)	179(4)	n.i.

a n.i. = no inhibition; i.e., no evidence of irreversible inhibition was discernible within the considered time and concentration ranges. Data shown are mean values ± SEM of three experiments, each performed in duplicate.

Contrary to the dipeptide nitriles with a large P1 residue listed in Table 2, replacement of *m*-carboxybenzylserine with a glycine-derived alkyne moiety at P1 led to a drastically reduced inhibitory activity (**2b** vs **56c** or **2c** vs **56d**) for all investigated cathepsins. Nevertheless, further variations of P2 and P3 have revealed compounds with inhibitory activity such as **56e** and **56f**.

Dipeptide alkyne **56e** was synthesized with the aim of obtaining a cathepsin K-selective inhibitor with 3-iodobenzoyl being the preferred residue at P3 according to this study and leucine being preferred at P2, as reported for nitrile-based inhibitors.^72^ Indeed, the inactivation constants determined for **56e** were highest for cathepsin K, despite its low selectivity.

According to literature reports, cathepsin S prefers β-cyclohexylalanyl at P2 over phenylalanine.^115,123,131^ Correspondingly, **56f** showed a significantly increased cathepsin S inhibitory potency in comparison to that of **56c** with moderate selectivity over cathepsin B and L (>8-fold). Due to the preference of cathepsin K for aliphatic residues at P2, no selectivity over cathepsin K was achieved. Additionally, the cathepsin S inactivation constant of 283 M^–1^ s^–1^ for **56f** was far below the constant determined for **2k** (10133 M^–1^ s^–1^).

Inspired by the nanomolar nitrile-based inhibitor reported by Frizler et al. (*K*_i_ = 33 nM),^102^ dipeptide alkyne **62** was synthesized. This compound showed selective inactivation of cathepsin S with no inhibition of cathepsins B, L, or K observed in the tested concentration range. However, given the low second-order inactivation constant of 14 M^–1^ s^–1^, the compound’s applicability for biomedical purposes might be hampered, particularly with regard to translation for molecular imaging *in vivo*.

Compounds **56e** and **56f** demonstrate the inhibitory potential of easily accessible propargylamine-based dipeptides with respect to cysteine cathepsins and underline the importance of a suitable moiety at P1 for increasing inhibitor efficiency.

#### Influence of the Free Carboxylic Group at P1 on the Inhibition Efficiency of Selected Dipeptide Alkynes

In general, charged residues generally impair membrane permeability, whereas esterification of free carboxylic groups can potentially enhance it.^132^ This was demonstrated for the cathepsin B-selective epoxysuccinyl peptide-based inhibitor CA-074 and its methyl ester CA-074Me, which undergoes intracellular hydrolysis.^133^ To investigate the influence of the carboxylic group at P1 on the inhibitory potency, methyl esters of dipeptide alkynes **2b** and **2f** were synthesized via reaction with diazomethane (details are given in the Supporting Information). Methyl esters **63** and **64** as well as allyl-protected precursor **53k** of dipeptide alkyne **2k** were characterized using the fluorimetric enzyme assay. Compound **53k** represents the nonradioactive intermediate in the aspired radiosynthesis of [^18^F]**2k**. The determined inactivation constants are included in Table 6.

**Table 6:**
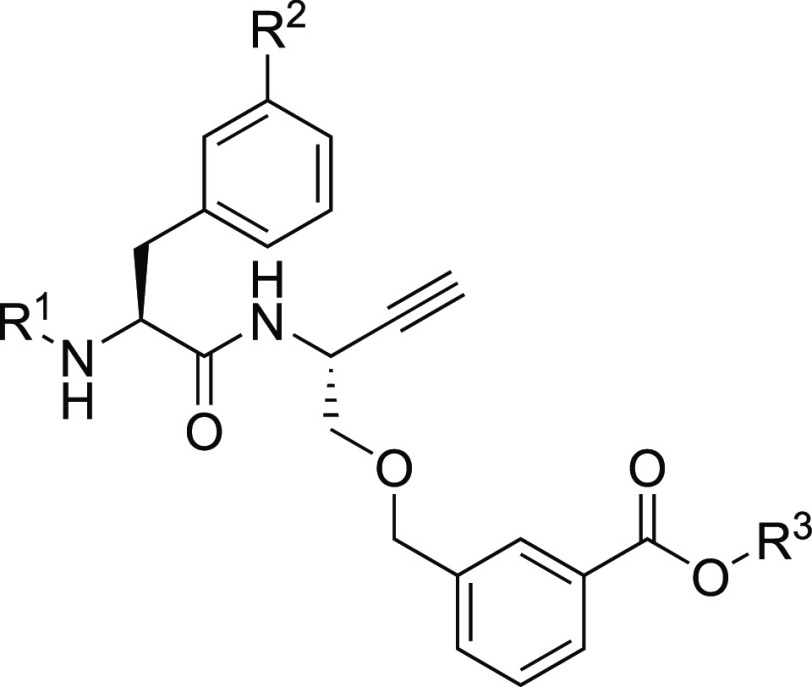
Inhibitory Activity of Dipeptide Alkynes with or without a Free Carboxylic Group at P1 with Respect to Cathepsins B, L, S, and K^a^

				*k*_inact_/*K*_I_ (M^–1^ s^–1^)			
compound	R^1^	R^2^	R^3^	CatB	CatS	CatL	CatK
**2b**	4-fluorobenzoyl	Me	H	85(3)	682(85)	281(30)	48(5)
**63**			Me	n.i.	12(2)	79(13)	n.i.
**2f**	3-iodobenzoyl	Me	H	45(1)	n.i.	1968(153)	n.i.
**64**			Me	15(2)	137(9)	537(18)	n.i.
**2k**	4-fluorobenzoyl	I	H	1179(222)	10133(842)	2128(230)	121(5)
**53k**			allyl	141(19)	3589(79)	1246(116)	n.i.

a The measurement was performed in three independent experiments (each as a duplicate determination) in assay buffer (pH 6.0) with 1.5% DMSO. n.i. = no inhibition; i.e., no evidence of irreversible inhibition was discernible within the considered time and concentration ranges. Data shown are mean values ± SEM of three experiments, each performed in duplicate.

As mentioned above, the carboxylic group presumably interacts with the histidine imidazole rings of the occluding loop in cathepsin B. This correlates well with the observed significantly reduced cathepsin B inhibitory potency after methylation or allylation of the carboxylic group. Similar structure–activity relationships for dipeptide nitriles were observed by Greenspan et al.^86^ Consistent with the results obtained in the molecular docking studies described in the following section, a reduction in the inhibitory potency upon esterification was observed for all tested cathepsins, despite cathepsins S, L, and K lacking a structural element corresponding to the occluding loop. In addition to polar contacts formed by the carboxylic groups discovered during covalent docking as reported below, unfavored solvation of the methyl ester compared to the free carboxylate might contribute to the attenuated inhibitory activity, as the S1 binding pocket in general is poorly defined in papain-like cysteine proteases and larger P1 residues are mainly solvent-exposed.^116^ The cathepsin L selectivity of **2f** is lost after introduction of the methyl group, even if the resulting methyl ester **64** preferably inhibits cathepsin L with an inactivation constant of 537 M^–1^ s^–1^ and a selectivity factor of 3.9 versus cathepsin S. The kinetic characteristics render the potentially membrane-permeable methyl ester **64** an interesting candidate as an activity-based probe for targeting intracellular cysteine cathepsins. This can be expected on the basis of the results of Mons et al., who reported inactivation of cathepsin K by an alkynylated Odanacatib derivative in an osteoclast–bone resorption model with an inactivation constant of 833 M^–1^ s^–1^.^121^

The cathepsin S inactivation constant of **2k** (10133 M^–1^ s^–1^) decreased to 3589 M^–1^ s^–1^ for the corresponding allyl ester **53k**. While the selectivity toward cathepsin B (9- and 25-fold for **2k** and **53k**, respectively) and cathepsin K (>80-fold) is maintained, the selectivity factor decreases upon esterification from 5 to 3. Nevertheless, the selectivity profile of **53k** is largely similar to that of **2k**. Therefore, hydrolysis of **53k** inside cells would not significantly change the ratio of inhibitory potency toward the four cathepsins. On the contrary, the epoxysuccinyl peptide CA-074 and its methyl ester show distinct selectivity profiles, as at pH 5.5 CA-074 is selectively inactivating cathepsin B while CA-074Me, at the identical pH, inhibits preferably cathepsin S over cathepsin B with a selectivity factor of 2.5.^134^ Furthermore, inactivation of cathepsin L by CA-074Me at higher inhibitor concentrations was reported.^135,136^ On this basis and considering the option of a facile radiofluorination by taking advantage of its 4-fluorobenzoyl group, **53k** can be furthermore selected as a candidate for labeling with fluorine-18. This would also allow radiosynthetic access to [^18^F]**2k** by subjecting [^18^F]**53k** to either hydrolytic or Pd(0)-catalyzed allyl transfer-based ester cleavage. Therefore, potential radiotracers for the detection of intra- and extracellular cathepsin could be obtained.

#### Modeling of Covalent Enzyme–Inhibitor Complexes

**Figure 14:**
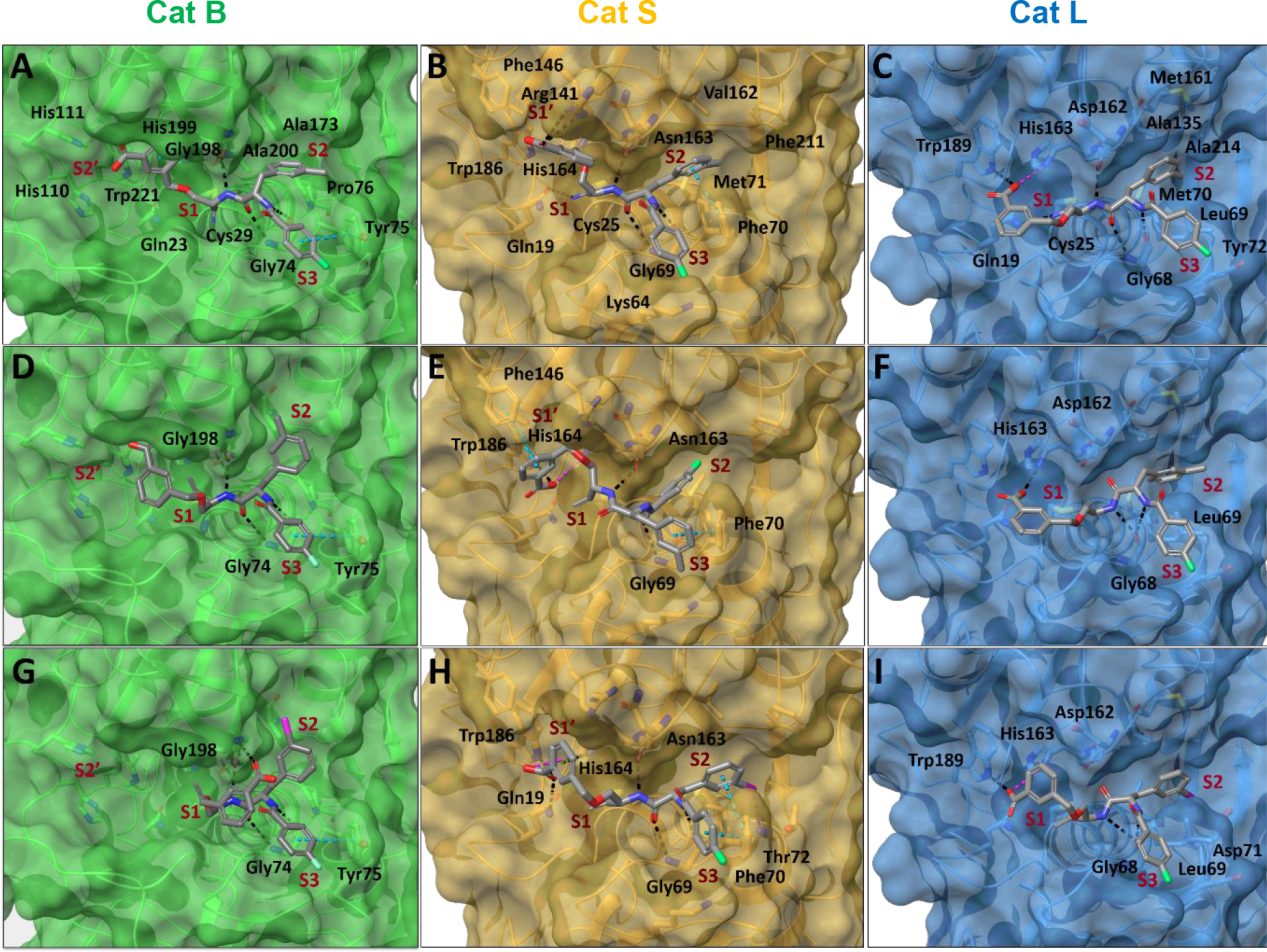
Molecular models for covalent enzyme–inhibitor complexes predicted *in silico*. Cathepsins B, S, and L in cartoon and transparent surface representations are colored green, orange, and cyan, respectively. Interacting protein residues are shown as sticks, colored by atom type and labeled. Inhibitors (A–C) **1b**, (D–F) **2b**, and (G–I) **2k** are shown as gray sticks and colored by atom type. Pocket binding sites S1–S3 are indicated by red labels. Intermolecular hydrogen bonds, salt bridges, π–π and halogen hydrogen bond interactions are depicted as black, magenta, cyan, and purple dashed lines, respectively. Figure generated in Maestro (Schrödinger).

Covalent molecular docking was performed to gain further insights into the molecular basis governing potency and selectivity in the recognition of the obtained inhibitors. Nitriles **1b** and **35a** and alkynes **2b**, **2c**, **2f**, **2k**, and **53k** were selected for docking at cathepsins B, S, and L. In the case of cathepsin L, the covalent docking studies were extended to inhibitors **2e** and **2h**–**j** (Figure 14 and Figures S50–S52).

All of the obtained models of the covalent cathepsin B–inhibitor complexes, except the complex with compound **53k**, displayed hydrogen bonds involving the main-chain amide groups, which are contacted by Gly74 (Gly-NH···CO-P2 and Gly-CO···NH-P2) and Gly198 (Gly-CO···NH-P1). This recognition mode is consistent with that observed in the crystal structure of cathepsin B–inhibitor complexes.^86^ With the exception of compound **53k**, the investigated inhibitors were predicted to be disposed at the tripartite cavity comprising pockets S1–S3. Shallow pocket S1 was occupied by the thioimidate or vinylthioether groups formed through the thiol–nitrile or thiol–alkyne reactions, respectively, and the C_β_ methylene group of the P1 moiety. For compounds **1b** and **35a**, the computational predictions suggest that hydrogen bonding to the imine group does not occur (Figure 14A and Figure S50A). Interestingly, a conformational preference at residue P1 was observed for compounds **1b**, **2b**, **2c**, and **2k**, as in those complexes the C_Ar_–C–O–C dihedral angle adopted values between 60° and 80° or approximately −80°, which could be favored by a stereoelectronic effect. Furthermore, the oxygen of the ether moiety did not participate in hydrogen bonds with cathepsin B. The carboxylate group at P1 of **2k** participated in hydrogen bonding with Gly198 (Figure 14G), while in compounds **1b** and **35a**, it was oriented toward His111 at the occluding loop defining pocket S2′, although no close contact was obvious (Figure 14 D and Figure S50A). The P1 phenyl ring of **1b** engaged in van der Waals contacts with Val176, His199, and Trp221, and in the case of **2f** with His110 and Trp221, while the triazolyl moiety of **35a** was oriented toward the methylene group of Gly198 (Figure S50A). Notably, upon comparison of the predicted complexes of the analogous nitriles **1b** and **35a**, it became obvious that the interaction of the active-site His199 with the carboxyphenyl residue in **1b**, which seems to be of π–π character, was not observed for the corresponding 4-carboxytriazolyl residue, which is probably a result of the lower electron density of the triazole ring. This finding could explain the different inhibitory potencies of the two compounds despite their close structural relationship. The P2 entities participated in van der Waals interactions with Tyr75, Pro76, Ala173, Gly198, and Ala200, which delineate hydrophobic pocket S2. Except for **2f** and **53k**, residue Tyr75 was also involved in the recognition of the N-terminal acyl caps by π–π interactions of the edge-to-face type within the S3 pocket. A substitution at the *meta* position of the phenyl ring occupying pocket S3 was predicted to be less favorable as this is slightly displacing the P3 aromatic ring of Tyr75. A different recognition mode was obtained for the best-ranked binding pose of **53k** (Figure S50B). Here, the large residue at P1 was accommodated by pocket S3, pocket S2 was not occupied, the P2 side chain was located in pocket S1 making π–π interactions with His199, and the terminal 4-fluorobenzoyl group was predicted to establish van der Waals contacts with Gly121 and Glu122. This binding mode was further supported by a hydrogen bond with the main-chain amide hydrogen of Cys29 and another with the side-chain amide hydrogen of Gln23 each to the P2 carbonyl oxygen as hydrogen bond acceptor. In summary, the molecular recognition of the investigated compounds by cathepsin B is mainly mediated by the formation of main-chain hydrogen bonds with Gly74 and Gly198 as well as π–π interactions of the P3 aromatic moiety with Tyr75, with the exception of compound **53k**.

For cathepsin S, the predicted covalent complexes indicated a recognition mode similar to that observed in a previously reported crystal structure of the enzyme complexed with a dipeptide nitrile-derived inhibitor (Figure 14 and Figure S51).^131^ Analogously to cathepsin B, the recognition of the inhibitors by the enzyme exhibited two main-chain hydrogen bonds with Gly69 (Gly-NH···CO-P2 and Gly-CO···NH-P2) and one with Asn163 (Asn-αCO···NH-P1). In the case of **2c**, the Gly^69^-NH···CO-P2 contact is missing, whereas **2f** is additionally lacking the Asn^163^-αCO···NH-P1 hydrogen bond, although a new contact involving Gly^165^-NH···CO-P2 was predicted. The lacking hydrogen bonds reflect their low (**2c**) and absent (**2f**) inhibitory activities with respect to cathepsin S. As expected, substituents P1–P3 of the investigated inhibitors were accommodated by the corresponding pockets S1–S3. Similar to cathepsin B, the imine and alkene groups were positioned at pocket S1. The imine group of **1b** was hydrogen bonded to Gln19, stabilizing the oxyanion hole. In the case of **35a**, hydrogen bonds to the imine were predicted to involve the main chain of Asn163 and the side chain of active-site His164. Interestingly, the oxygen of the ether moiety in **1b** formed one hydrogen bond with the side chain of His164. In contrast to cathepsin B, the carboxylate group at P1 is engaging in hydrogen bonds. For these contacts, the side chains of Arg141 at site S1′ proximal to the active site or His164 were predicted to act as hydrogen bond donors. Nevertheless, additional hydrogen bond interactions were predicted for **35a** and **2k** with Gln19, and in the case of **2k** also with Trp186 in the proximal S1′ subsite (Figure 14H). This finding is consistent with the previously reported crystal structure of cathepsin S in complex with a dipeptide nitrile containing *O*-benzylserine at P1, where this residue was oriented toward the S1′ pocket.^131^ In the case of compound **2b** (best-ranked binding pose), the aromatic ring at P1 was predicted to participate in π–π interactions of the edge-to-face type with Phe146 in the distal region of pocket S1′, whereas no evidence of such contacts was obtained for the other inhibitors (Figure 14E). Interestingly, the 3-allyloxycarbonylphenyl moiety of **53k** forms multiple van der Waals interactions with the side chains of Tyr18 and Trp186 and, furthermore, with the backbone of the Gly20-Ser21-Cys22-Gly23 section. The P2 moieties of all investigated inhibitors, except for best-ranked binding pose of **2b**, were located in pocket S2 and participated in π–π interactions with Phe70. In addition, van der Waals contacts with Trp26, Met71, Gly137, Gly165, Val162, and Phe211 were predicted. It is noteworthy that the iodine atom of the 3-iodo-phenyl alanine side-chain group present in **2k** and **53k** was predicted to act as a hydrogen bond acceptor for the NH group of Thr72 for **2k** and **53k**, and also the NH group of Met71 for **53k** [predicted donor–acceptor distances of 4.2 and 3.7 Å (Figure 14H and Figure S51B)]. This interaction might strongly contribute to the compounds’ high inhibitory potency and selectivity toward cathepsin S. In general, it has been found that halogen atoms attached to aromatic rings tend to form such hydrogen bonds in protein–ligand complexes, in addition to the formation of halogen bonds.^137^ The P3 acyl groups, except for that of compound **2b**, were predicted to be accommodated in the S3 pocket and packed between Phe70 and Gly69. For the inhibitors bearing a 4-fluorobenzoyl group, the fluorine atom was positioned toward Lys64. In the particular case of the best-ranked compound **2b**, residue P2 was disposed in pocket S3 being able to establish π–π interactions with Phe70, while residue P3 occupied pocket S2. Interestingly, the second-best ranked binding pose of **2b** [according to docking score (see Experimental Section)] showed the expected recognition mode for residues P2 and P3 establishing π–π interactions with Phe70 (Figure S51A). In summary, the predicted recognition modes of the selected inhibitors of cathepsin S reveal that their recognition pattern is characterized by the formation of three main-chain hydrogen bonds with Gly69 and Asn163, the establishment of additional interactions with active-site residues, and, to a lesser extent, the ability to occupy the oxyanion hole. In addition, favorable π–π interactions in pockets S2 and S1′ seem to be crucial for modulating the inhibitory potency toward cathepsin S. These findings are in accordance with activity data of peptidic cathepsin S substrates, for which cooperative targeting of the S2 and S1′ subsites was found to be beneficial for selectivity.^22,138,139^ The strongest inhibitory capacity of compounds **2k** and **53k** can be attributed to the presence of hydrogen bonds in pocket S2 mediated by their iodine atom.

The results of covalent docking calculations for cathepsin L are largely in agreement with previously reported crystal structures of cathepsin L–nitrile complexes (Figure 14 and Figure S52). Similar to cathepsins B and S, the obtained models of the enzyme–inhibitor complexes indicated the presence of main-chain hydrogen bond interactions: two with Gly68 (Gly-NH···CO-P2 and Gly-CO···NH-P2) and one with Asp162 (Asp-αCO···NH-P1). Interestingly, the complexes formed by compounds **2h**, **2i**, **2k**, and **53k** lost one of these interactions. In the case of nitriles **1b** and **35a**, the resulting imine group occupies the oxyanion hole by hydrogen bonding to the main chain of Cys25 and the side chain of Gln19 (Figure 14C), as predicted for cathepsin S. The tripartite cavity was occupied by residues P1–P3 of the inhibitors, except for compound **53k**. In general, the carboxylate group at P1 was involved in hydrogen bonding to His163, Trp189, or both (Figure 14H and Figure S52A). Hydrophobic pocket S2 was occupied by residue P2 establishing van der Waals contacts with Leu69, Met70, Ala135, Met161, and Ala214. Interestingly, the iodine at the *meta* position of the benzoyl group of inhibitor **2k** formed a hydrogen bond with the main chain of Asp71 (Figure 14I), which is similar to the case of the complex of **2k** with cathepsin S. A similar scenario was predicted for the best-ranked pose according to the Prime energy (see Experimental Section) obtained for **2i**, with residues P2 and P3 being recognized by pockets S3 and S2, respectively. In particular, the bromine atom of the *m*-bromobenzoyl residue was located in pocket S2 forming a hydrogen bond to the backbone NH group of Met70 (Figure S52B). Nevertheless, considering the best-ranked binding pose of **2i** according to docking score (see Experimental Section), the expected recognition mode for residues P2 and P3 was obtained (Figure S52C). In the particular case of **53k**, an inverted binding mode was predicted in which the P1 moiety binds in the S2 pocket with the ester carbonyl oxygen acting as a hydrogen bond acceptor for the amide hydrogen of Met70 (Figure S52D). Apart from the special binding pose of **53k**, the P3 aryl moieties were found to be commonly involved in van der Waals interactions with Leu69 and, to a different extent, with Tyr72 or Glu63 in pocket S3. It is noteworthy that the iodine atom of the 3-iodobenzoyl group of **2f** established a halogen bond with Gly61, whose carbonyl oxygen acts as an iodine acceptor (predicted I···O distance and C–I···O angle of 3.4 Å and 167°, respectively). This confirms the function of the iodine atom proposed on the basis of the obtained structure–activity relationships discussed above in combination with previously published crystal structure.^118^ The predicted models of the enzyme–inhibitor complexes suggest that the inhibitory potency of the considered compounds toward cathepsin L is related to the establishment of hydrogen bonds with the main chains of Gly68 and Asp162, as well as interactions at the active site with His163 and Trp189. The inhibitory effect of the most potent compounds appears to be further related to the formation of halogen-mediated interactions in pockets S2 and/or S3.

Summarizing the results obtained from the computational docking studies, we find the *m*-carboxybenzylserine moiety at position P1 is not capable of conferring selectivity for cathepsin B, because the aryl moiety is prone to interact with the active-site histidine residue and the carboxylate finds even more polar contacts in the case of cathepsins S and L. This conclusion is supported by the experimentally obtained inhibitory activities. Therefore, the perception of Greenspan et al. that the *m*-carboxylate can interact with His110 and His111 of the occluding loop of cathepsin B cannot be maintained.^86^ In the context of selectivity across the *in silico* investigated enzymes, recognition in pocket S1′ as well as hydrogen bonding to halogen atoms in pocket S2 seem to be responsible for the highest inhibitory capacity of the investigated inhibitors against cathepsin S in comparison to cathepsins B and L. In the case of compound **2f**, its selectivity toward cathepsin L can be rationalized by the formation of one halogen bond through residue P3 in pocket S3.

Despite the valuable insights gained from the molecular docking described above, the different behavior of alkynes and analogous nitriles in terms of SAR and selectivity profiles cannot be explained on this basis. To this end, perspective QM/MM studies could be informative for understanding the differences in covalent adduct formation between the active-site cysteine and the C≡C and C≡N bonds.

#### Nonadditivity Analysis of SARs for Matched Molecular Pairs of Selected Dipeptide Nitriles and Alkynes

The investigation of structure–activity relationships for nonadditive effects by arithmetical comparison of the binding affinities in matched molecular pairs of protein ligands can provide hints about cooperative effects between the binding of distinct ligand moieties or alternative binding modes associated with structural changes or indicate altered conformational flexibility within the ligand molecule. Such investigations were previously applied to SAR data of reversible cysteine protease inhibitors.^140^ Specifically, the differences in binding affinities or inhibitory activities of four compounds that are related to each other by two structural variations in a double-transformation cycle need to be considered.^141,142^ For this purpose, the differences in logarithmically transformed inhibition and second-order inactivation constants [Δp*K*_i_ and Δlog(*k*_inact_/*K*_I_), respectively] were calculated followed by calculation of the secondary differences [ΔΔp*K*_i_ and ΔΔlog(*k*_inact_/*K*_I_), respectively]. Values different from zero indicate nonadditive effects. However, in reality, considering the experimental error of the assay method, true nonadditivity is evidenced by absolute values of >1.^142^ In those cases, in which irreversible inhibition of alkynes was not detectable, second-order inactivation constants were consequently assumed to be equal to zero and log(*k*_inact_/*K*_I_) values of −∞ were included in the formal calculation.

**Figure 15:**
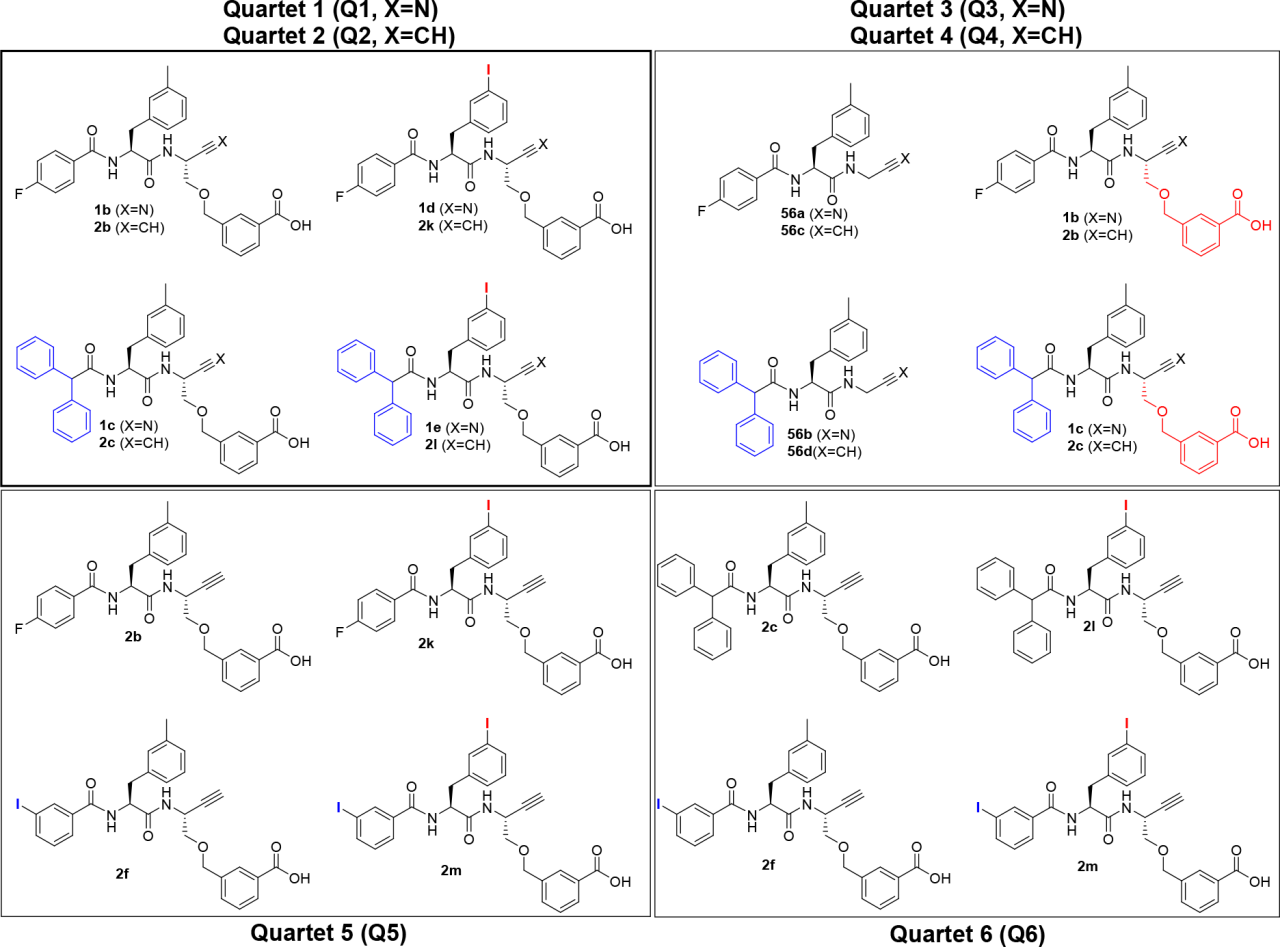
Quartets of matched molecular pairs of dipeptide nitriles and alkynes, which are structurally related to each other by double-transformation cycles.

**Figure 16:**
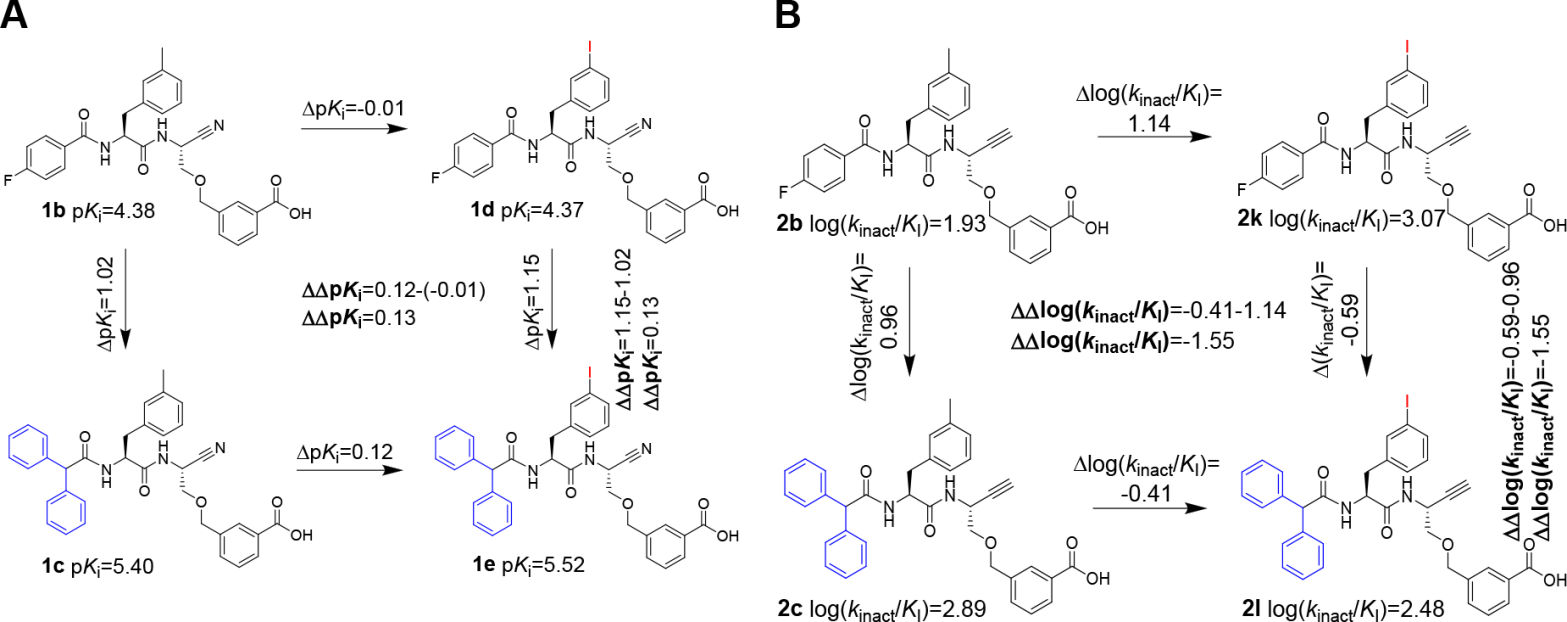
Nonadditivity analysis in double-transformation cycles (horizontal arrows for *m*-tolyl → *m*-iodophenyl and vertical arrows for *p*-fluorobenzoyl → diphenylacetyl) for (A) dipeptide nitrile and (B) dipeptide alkyne quartets **Q1** and **Q2**, respectively, as exemplified for the inhibition of cathepsin B.

In particular, the compound quartets shown in Figure 15 were assigned among the investigated dipeptide nitriles and alkynes and subjected to nonadditivity analysis, with regard to their inhibitory activities toward cathepsins B, L, S, and K. The calculations for the nitrile and alkyne quartets are shown exemplarily for quartets **Q1** and **Q2** for cathepsin B in Figure 16.

The obtained results are listed in Table 7. In the case of the dipeptide nitriles, the SAR should be judged largely additive, with the exception of cathepsin K inhibition within compound quartet **Q3**. In contrast, nonadditivities of >1 are predominant within the quartets of dipeptide alkynes in the case of all four cysteine cathepsins. In some cases, in which subtle structural changes transform virtually absent inhibition into potent irreversible inactivation, nonadditivities even reach infinity, which indicates extreme nonadditive effects. The contrasting results obtained for the matched nitrile/alkyne quartets **Q1**/**Q2** and **Q3**/**Q4**, each with identical structural variations at the dipeptidic scaffold, reflect the steep SAR observed for alkynes as reported above and can be interpreted as an indication of strong cooperativity between covalent bond formation with the C≡C bond in the active site and noncovalent interactions in the side-chain binding pockets.

**Table 7:** Additivity of Inhibitory Activities toward Cysteine Cathepsins within Compound Quartets defined in Figure 15

quartet		CatB	CatS	CatL	CatK
**Q1**	ΔΔp*K*_i_	0.14	0.21	0.11	0.24
**Q2**	ΔΔlog(*k*_inact_/*K*_I_)	–1.55	0.09	–0.72	**∞**
**Q3**	ΔΔp*K*_i_	–0.12	0.12	0.12	1.26
**Q4**	ΔΔlog(*k*_inact_/*K*_I_)	0.96	–1.16	–1.17	–∞
**Q5**	ΔΔlog(*k*_inact_/*K*_I_)	–0.44	**∞**	–0.71	–0.40
**Q6**	ΔΔlog(*k*_inact_/*K*_I_)	1.11	**∞**	0	–∞

### Inhibitory Activity in the Cellular Environment

Contrary to assays that use isolated enzymes, the tissue distribution, the presence of endogenous inhibitors and physiological substrates, and the localization of the target enzyme affect the kinetics of enzyme–inhibitor complex formation *in vivo*. Therefore, because the inhibitors were developed as potential candidates for radiotracers, the inhibitory activity of dipeptide alkynes should be proven in a cellular environment. To obtain information about potential nonspecific adsorption of the inhibitors, which is a frequent cause of the failure of radiotracer candidates,^143^ the chromatographic hydrophobicity indices at an immobilized artificial membrane (CHI IAM) were determined for all tested inhibitors. The obtained values were in the ranges of 22.8–39.5 for the dipeptide nitriles and 24.6–46.2 for the dipeptide alkynes and indicate no propensity for a high level of nonspecific binding (see Table S3).

**Figure 17:**
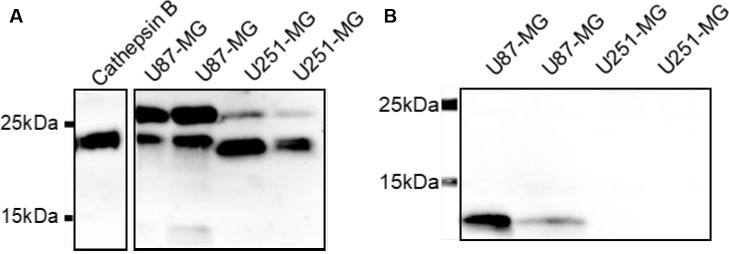
Analysis of expression of (A) cathepsin B and (B) cystatin B in the total cell lysate by Western blotting.

**Figure 18:**
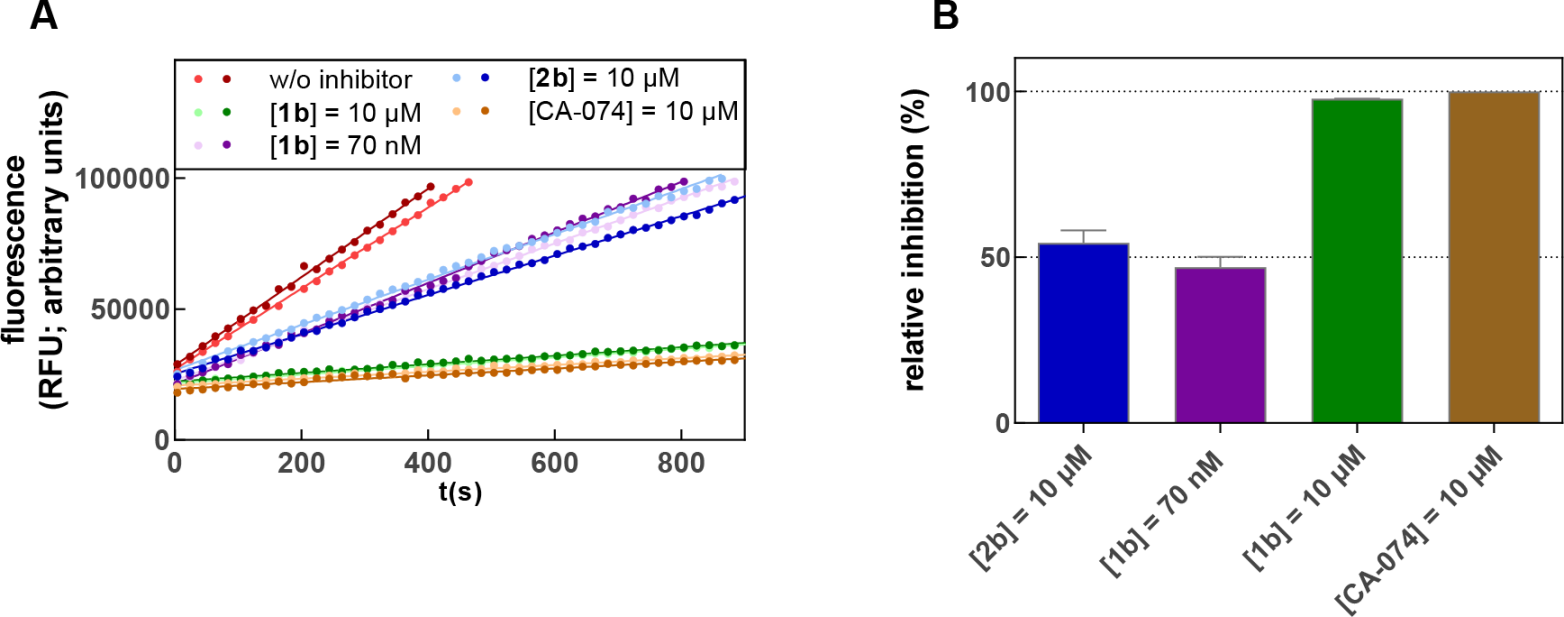
Dipeptide nitrile **1b** and dipeptide alkyne **2b** compared with the literature inhibitor CA-074 in the U251-MG cell lysate. (A) Turnover of internally quenched substrate Abz-GIVR↓AK(Dnp)-NH_2_ (↓ indicates the cleavage site) as measured by increasing fluorescence intensities after preincubation for 30 min with the respective inhibitor. (B) Relative inhibition normalized to the inhibitory effect of CA-074 (mean ± SEM). The measurement was performed as a duplicate determination in assay buffer (pH 6.0) containing 0.5 mg/mL protein, 100 μM Abz-GIVRAK(Dnp)-NH_2_, and 1% DMSO.

**Figure 19:**
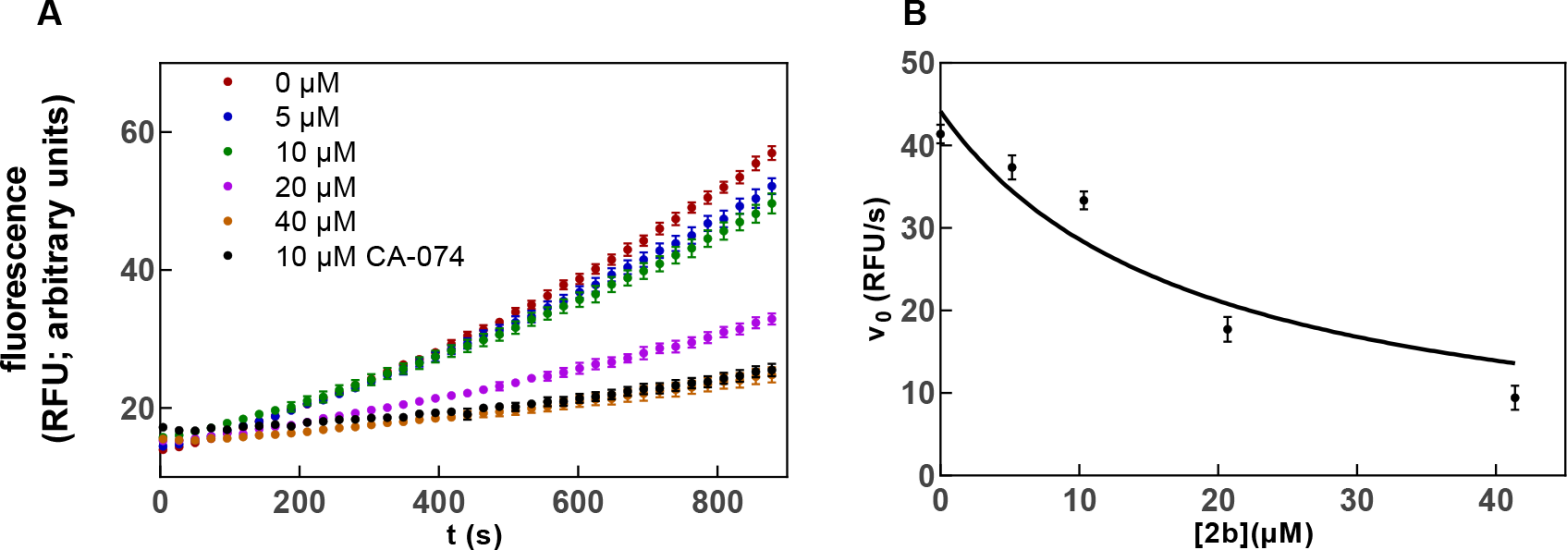
(A) Increasing fluorescence intensity originating from the turnover of internally quenched substrate Abz-GIVRAK(Dnp)-NH_2_ shown for an examplary measurement as a duplicate determination and (B) initial velocities averaged over three measurements of *v*_0_ = *f*([**2b**]) for inhibitor **2b** on viable U87-MG cells. In panel A, the primary curves show an upward curvature presumably due to the continuously secreted enzyme. Initial velocities were determined from substrate turnover curves via linear regression over the first 600 s. For panel B, analysis of *v*_0_ = *f*([**2b**]) was performed according to eq III. Measurements were performed in three independent experiments (each as duplicate determinations) in assay buffer (pH 6.0) containing 100 μM Abz-GIVRAK(Dnp)-NH_2_, 25 ng/mL cathepsin B, and 1.5% DMSO. Shown are mean values ± SEM.

To identify suitable model cell lines, various tumor cell lines were investigated with regard to intra- and extracellular cathepsin B activity using the hexapeptide substrate Abz-GIVRAK(Dnp)-NH_2_ and the cathepsin B specific inhibitor CA-074 (for detailed information, see the Supporting Information). Both glioblastoma cell lines U87-MG and U251-MG showed high protein levels in the Western blot analysis and are often described in the literature as cathepsin B-overexpressing cell lines (Figure 17).^144^ However, while U251-MG showed high cathepsin B activity in the cell lysate and lower activity on living cells, U87-MG showed considerably higher cathepsin B activity on living cells and almost no cathepsin B activity in the cell lysate despite high cathepsin B protein levels that were detected in the Western blot. The presence of high levels of the endogenous cathepsin B inhibitor cystatin B in U87-MG cell lysates, as detected by Western blot analysis, was identified as a likely explanation for this observation. Therefore, the inhibitory activity of selected compounds was tested in cell lysates using U251-MG cell lysates (Figure 18) and activity on living cells was investigated using U87-MG cells (Figure 19), each in comparison to CA-074 as a well-characterized irreversible cathepsin B inhibitor.^134^

#### Inhibition of Cathepsin B in Cell Lysates

The inhibition assay with the U251-MG cell lysate was performed under conditions selected in orientation to the assay using the purified enzyme. The quenched hexapeptide Abz-GIVRAK(Dnp)-NH_2_ was used to be a fluorogenic substrate.^145^ As the experiments were performed parallel to inhibitor structure optimization, lead compounds **1b** and **2b** were chosen for testing. The substrate conversion graph is shown in Figure 18A.

After preincubation for 30 min with the cell lysate at an inhibitor concentration of 10 μM, dipeptide nitrile **1b** revealed an inhibitory activity that was almost equal to that of CA-074 under identical conditions. Inhibition by dipeptide alkyne **2b** was also significant and reached >50% in relation to the inhibitory effect of CA-074. Hence, dipeptide alkyne-based cathepsin B inhibition in the complex biological matrix of the U251-MG cell lysate was successfully demonstrated. The stronger inhibitory effect of nitrile **1b** compared to that of alkyne **2b** reflects the different electrophilicity and thus the differential inhibition kinetics conferred by the two warheads, each containing sp-hybridized carbon atoms.

#### Inhibition of Cathepsin B on Living Cells

As high extracellular levels of cathepsin B are associated with tumor progression and certain other diseases such as fibrosis,^146^ the inhibitors were designed to address extracellular cysteine cathepsins. Extracellular cathepsin B is partially associated with the membrane-bound annexin II tetramer, which modulates the enzyme’s activity in the context of tumor invasion and metastasis.^147^ Consequently, the tumor targeting by cathepsin B inhibitors should relate to their binding to the cell surface-bound enzyme fraction.

A more complex experimental setup was required to investigate inhibitor activity on living cells. To this end, U87-MG cells were cultivated in polylysine-coated clear-bottom 96-well plates to 95% confluency. Prior to the addition of the assay buffer and inhibitor, the cells were carefully washed with phosphate-buffered saline (PBS). Cell integrity in assay buffer over the duration of the experiment was validated using an LDH activity-based assay kit and visual monitoring under a microscope. Preincubation was performed in an incubator under standard cell culturing conditions for 30 min. The measurement was started by adding the substrate Abz-GIVRAK(Dnp)-NH_2_. The obtained substrate conversion curves in the presence of different concentrations of dipeptide alkyne **2b** are shown in Figure 19.

A significant inhibition was observed with increasing concentrations of **2b**, with a concentration of 40 μM being required for 100% inhibition compared to 10 μM CA-074 on living U87-MG cells. The control curve shows an increase in reaction velocity over the observation time. This probably reflected increasing amounts of extracellular cathepsin B due to continuous secretion of the enzyme during the course of the experiment. Consequently, it was not possible to determine *k*_inact_/*K*_I_ because the decrease in reaction velocity typical for irreversible enzyme inhibition was not observed because enzyme inactivation is obviously overcompensated by continuous secretion of cathepsin B. Analysis of the data by linear regression was restricted to the initial 600 s, and plotting the resulting velocities against the inhibitor concentrations allowed for the determination of an IC_50_ value of 15.8 μM, compared to a value of 5.9 μM obtained for preincubation (30 min) of **2b** with isolated cathepsin B (see Table S3).

## Conclusion

Cysteine cathepsins play an important part in tumor progression, with the most evidence existing for cathepsin B. Therefore, these enzymes represent highly promising targets for the development of radiolabeled activity-based probes, which would allow for their quantitative detection at the cellular level and potentially also for imaging *in vivo* by PET and SPECT. Inspired by recent reports on the ability of peptidic alkynes to covalently bond to cysteine proteases,^78,79^ alkynes were designed as irreversible inhibitors directed toward the oncologically relevant cysteine cathepsins B, L, S, and K starting from potent dipeptide nitriles.

Partial epimerization was encountered when amino aldehydes were subjected to the Gilbert–Seyferth homologation with the Bestmann–-Ohira reagent. Therefore, a stereoconservative synthesis via Garner’s aldehyde had to be established for dipeptide alkynes with serine-derived side chains in P1, which furnished stereochemically homogeneous products in yields of 4–8% over 14 steps. By specifically varying the residues at positions P1–P3, we were able to generate dipeptide alkynes as effective irreversible inhibitors for each of the four cysteine cathepsins. The irreversibility of inactivation was verified exemplarily for cathepsins B and S in a jump-dilution experiment. This confirms the ability of terminal alkynes to react with various cysteine proteases when the C≡C bond is brought close to the thiol at the active site of the enzyme–ligand complex supported by additional noncovalent interactions.

Despite the identification of a cathepsin L-selective irreversible inhibitor in dipeptide alkyne **2f**, obtaining selective alkynes remains challenging due to the overlapping substrate specificity of the different members of the cysteine cathepsin family. Accordingly, no dipeptide alkyne showed exclusive selectivity toward cathepsin B. Further exploration of SARs will potentially result in selective inhibitors for each of the tested cathepsins. Despite the virtual chemical inertness of alkynes under physiological conditions, inactivation constants as high as 10133 M^–1^ s^–1^ were determined for inhibition of cathepsin S by compound **2k**. This clearly demonstrates the potential of terminal alkynes as suitable warheads for the development of irreversible cysteine protease inhibitors. For selected examples, experimental results were rationalized by computational covalent docking, which has revealed that the *m*-carboxybenzylserine-derived moiety at P1 is prone to interact with subsites S1 and S1′ of cathepsins B, S, and L. Therefore, this residue should be judged unsuitable for conferring selectivity against cathepsin B, even though it is beneficial for improving inhibitory potency and aqueous solubility.

Comparing the selectivity profiles obtained for the dipeptide alkynes with the corresponding dipeptide nitriles, we observed a deviation from the expected congruence. Despite the fact that the inhibitor structure is largely preserved by isoelectronic exchange of nitrogen alone, the relative inhibitory activities of the dipeptide alkynes and nitriles differ with respect to the various cathepsins. Thus, the inhibitory potency of reversibly inhibiting nitriles does not necessarily translate into potent irreversible inhibition by alkynes. Therefore, the inhibitory potency of peptidic alkynes toward cysteine cathepsins can be only vaguely predicted on the basis of inhibition data of their nitrile counterparts.

Overall, peptide-derived alkynes constitute a promising new class of generic irreversible cysteine cathepsin inhibitors. High inactivation constants in combination with a low nonspecific thiol reactivity render dipeptide alkynes eligible for application as radiolabeled activity-based probes for the specific and quantitative detection of cysteine cathepsins *in vitro* and by molecular imaging *in vivo*. Their suitability for this purpose is currently being evaluated. For future studies, **53k** was identified as a highly interesting radiotracer candidate with promising inhibitory potency and sufficient selectivity toward cathepsin S.

## Experimental Section

### Synthesis

#### Reagents and Analytical Instrumentation

All commercial reagents and solvents were used without further purification, except for THF, which was freshly distilled prior to use over sodium using benzophenone as a water indicator.

Thin layer chromatography was performed on Merck TLC plates (silica gel 60 F_254_ on aluminum) using suitable solvent mixtures for development. Typically, mixtures of *n*-hexane and ethyl acetate or CH_2_Cl_2_ and MeOH were used as eluents. Visualization was performed under a UV lamp at 254 nm/366 nm or by staining with a solution of ninhydrin (0.1% m/V) in ethanol.

Analytical and preparative HPLC was performed as specified in the Supporting Information.

NMR spectra were recorded on a 400 MR NMR or 600 MR NMR spectrometer from Agilent Technolgies. Samples were dissolved in CDCl_3_, CD_3_CN, or DMSO, and recordings were carried out at 400 or 600 MHz (^1^H NMR), 376 or 564 MHz (^19^F NMR), and 101 or 151 MHz (^13^C NMR) at 25 °C. The spectra were analyzed using MestreNova (version 12.0.0-20080). Chemical shifts were calibrated on the basis of the solvent signal.

Mass spectra were recorded on a Xevo TQ-S spectrometer from Waters. The substances were ionized by electrospray ionization (ESI). Mass Lynx (version 4.1) was used to evaluate the spectra.

High-resolution mass spectra were recorded on an Accurate-Mass Q-TOF mass spectrometer from Agilent with the Agilent 1260 Infinity II coupled HPLC system.

Optical rotations were determined on a model 341 LC polarimeter from PerkinElmer Inc. The exact weight of the respective sample was dissolved in the specified solvent in a volumetric flask, and the mixture transferred to a glass cuvette with a path length of 10 cm. The measurements were taken at 25 °C. The specific angle of rotation was calculated from the optical angle of rotation using the following formula:
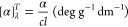
Iwhere *T* is the temperature in degrees Celsius, λ is the wavelength of the polarized light in nanometers, α is the measured angle of rotation in degrees, *c* is the concentration of the sample in grams per milliliter, and *l* is the path length of the cuvette in decimeters.

Crystallographic data for compound **48** were collected with a Bruker-Nonius Apex-II CCD diffractometer (Bruker, Madison, WI) with Mo Kα radiation (λ = 0.71073 Å) at 123 K. The structure was determined by direct methods and refined against *F*^2^ on all data by full-matrix least-squares refinements using the 2014 version of the program suites from G. M. Sheldrick.^148,149^ All non-hydrogen atoms were refined anisotropically; all hydrogen atoms bonded to C or N atoms were placed on geometrically calculated positions and refined using riding models. The absolute structure was determined on the basis of Flack’s *x* parameter.^150,151^ CCDC 2184827 contains the supplementary crystallographic data of compound **48**. These data can be obtained free of charge via http://www.ccdc.cam.ac.uk.

#### General Procedures for Inhibitor Synthesis

##### GP I. General Procedure for the Synthesis of Primary Amides

One equivalent of the amino acid derivative and 3 equiv of *N*-methylmorpholine (NMM) were dissolved in dry THF, and the solution was cooled to −15 °C. After the addition of 1.1 equiv of iBCF and the formation of a white precipitate, 5 equiv of NH_3_ (aqueous solution, 25%) was added. The solution was stirred for 10 min at −15 °C and then for 30 min at room temperature. The pH was adjusted to 4. Then the solvent was removed *in vacuo*, and the resulting residue dissolved in CH_2_Cl_2_ (20 mL) and washed with saturated NaHCO_3_ (3 × 10 mL) and brine (20 mL). The organic phase was dried over Na_2_SO_4_, and the solvent removed *in vacuo*.

##### GP II. General Procedure for Boc Removal

One equivalent of the Boc-protected compound was dissolved in CH_2_Cl_2_ (5 mL/1 mmol), followed by addition of TFA (5 mL/1 mmol), and the solution was stirred for 2 h at room temperature. The volatile components were removed in a N_2_ stream. The obtained residue was dissolved in a 2:1 H_2_O/CH_3_CN solvent, and the solution was lyophilized.

##### GP III. General Procedure for Amino Acid Coupling

One equivalent of the amine, 1.5 equiv of carboxylic acid, 4 equiv of DIPEA, and 1.5 equiv of PyBOP were dissolved in THF, and the mixture was stirred for 3 h. The solvent was removed *in vacuo*, and the obtained residue was dissolved in CH_2_Cl_2_ (10 mL). The organic phase was washed with saturated NaHCO_3_ (10 mL) and brine (10 mL), dried over Na_2_SO_4_, and evaporated.

##### GP IV. General Procedure for Acylation with Acyl Chlorides

One equivalent of acyl chloride was added to a solution of 1 equiv of amine and 3 equiv of TEA in dry CH_2_Cl_2_, and the resulting solution was stirred for 2 h. Subsequently, the solution was washed with 1 M HCl (10 mL), saturated NaHCO_3_ (10 mL), and brine (10 mL) and dried over Na_2_SO_4_, and the solvent was evaporated.

Occasionally, the formation of the terminally trifluoroacetylated dipeptide amide was observed during this step, which likely arises from the activation of trifluoroacetate as difluorobenzoic acid-derived mixed anhydride.^152^ Minimizing the content of trifluoroacetic acid in the starting material by repeated lyophilization prior to coupling of the P3 capping group accounted for an improved yield of this acylation step.^153^ Moreover, the use of triethylamine (TEA) as a base instead of NMM seems to improve the outcome of the acylation reaction, as shown by the higher yield of **9b** (84%) compared to that of **9a** (65%).

##### GP VII. General Procedure for Copper-Catalyzed Azide–Alkyne Click Reaction

One equivalent of the azido-functionalized amino acid derivative, 1 equiv of alkyne, 1 equiv of sodium ascorbate, and 0.5 equiv of CuSO_4_·5H_2_O were dissolved in ice-cold DMSO/H_2_O (2:1, 60 mL/mmol), and the solution was stirred overnight. Subsequently, the solution was acidified with 2 M HCl and extracted with ethyl acetate (4 × 30 mL). The combined organic layers were washed with brine (4 × 40 mL) and dried over Na_2_SO_4_, and the solvent was evaporated.

##### GP VIII. General Procedure for Amidation with Propargylamine

The synthesis was performed following the procedure described by Schmitz et al.^95^ One equivalent of the amino acid derivative and 1 equiv of NMM were dissolved in dry THF, and the solution was cooled to −25 °C. One equivalent of iBCF was added dropwise, and then a white precipitate formed. Subsequently, 2 equiv of propargylamine was added, and the solution was stirred for 10 min at −25 °C and 30 min at room temperature. The reaction progress was monitored via thin layer chromatography. The solvent was evaporated, and the residue was dissolved in ethyl acetate (25 mL). The solution was washed with HCl (2 × 10 mL, 1 M), saturated NaHCO_3_ (10 mL), and brine (10 mL) and dried over Na_2_SO_4_, and the solvent was evaporated.

All inhibitor compounds were determined to be >95% pure by HPLC analysis.

### Fluorimetric Assay with the Isolated Enzyme

For the kinetic characterization of the inhibitors, a stock solution in DMSO at a concentration of 10 mM was prepared for each inhibitor compound. Subsequently, this was diluted with assay buffer [100 mM sodium phosphate buffer (pH 6.0), 100 mM NaCl, 5 mM EDTA, and 0.01% Brij] containing 10% DMSO to obtain the respective desired intermediate dilutions at a concentration that was 20-fold higher than the highest, final inhibitor concentration in the assay. For inhibitor characterization, three separate experiments and six different concentrations for each compound (including a control in the absence of an inhibitor, for which neat DMSO was used instead of the inhibitor solution) were used.

The respective substrate and enzyme intermediate dilutions were prepared as listed below. In a black 96-well plate with a flat, transparent bottom, 10 μL of inhibitor intermediate dilution and 20 μL of substrate intermediate dilution were placed in 160 μL of assay buffer, and the mixture was incubated for 20 min at 37 °C. The enzyme working solution was preactivated for 5 min at 37 °C in a water bath, and then the reaction was started by adding 10 μL of this enzyme solution to the assay mixture containing the substrate and inhibitor. Substrate turnover was monitored over 15 min by detecting the increase in fluorescence in the Synergy 4 Hybrid Multi-Mode Microplate Reader from Biotek (15 min, 37 °C, excitation at 360 nm/40 nm, emission at 410 nm/40 nm, bottom-read). The sensitivity was set to 45 for cathepsins B, S, and K and 60 for cathepsin L. Three independent experiments were performed for each inhibitor and enzyme in duplicate. Analysis was performed in Prism version 5.02 (GraphPad Software, Inc.). The graphical representation was done as mean values ± SEM.

The determination of the *K*_m_ values of the different substrates at distinct enzymes, which is necessary for the calculation of the inhibition constants, is described in the Supporting Information. The following substrate and enzyme concentrations were adjusted for each cathepsin. For cathepsin B, the intermediate substrate dilution was obtained by diluting a 20 mM stock solution of Z-RR-AMC (*K*_m,Z-RR-AMC_ = 302.0 μM) in DMSO in a 1:10 ratio with assay buffer to 2 mM. For the enzyme intermediate dilution, the cathepsin B stock solution was first diluted to 54.44 μg/mL with cathepsin B enzyme buffer and then diluted to 0.5 μg/mL with assay buffer containing 10 mM DTT. The final enzyme concentration in the assay well was 25 ng/mL at a substrate concentration of 200 μM. For cathepsin S, the substrate intermediate dilution was obtained by diluting a 10 mM stock solution of Z-VVR-AMC (*K*_m,Z-VVR-AMC_ = 19.16 μM) in DMSO in a 1:25 ratio with assay buffer to 0.4 mM. For the enzyme working solution, the cathepsin S stock solution (0.1 mg/mL) was first diluted to 1 μg/mL with cathepsin S enzyme buffer and then diluted to 0.05 μg/mL with assay buffer containing 10 mM DTT. The final enzyme concentration in the assay well was 2.5 ng/mL at a substrate concentration of 40 μM. For cathepsin L, the substrate intermediate dilution was obtained by diluting a 20 mM stock solution of Z-FR-AMC (*K*_m,Z-FR-AMC_ = 3.05 μM) in DMSO first in a 1:20 ratio with DMSO and then in a 1:10 ratio with 10% DMSO in assay buffer to 0.1 mM. For the enzyme intermediate dilution, the cathepsin L stock solution was first diluted to 55.25 μg/mL with cathepsin L enzyme buffer and then diluted to 0.5 μg/mL with assay buffer containing 10 mM DTT. The final enzyme concentration was 25 ng/mL at a substrate concentration of 10 μM. For cathepsin K, the substrate intermediate dilution was obtained by diluting a 20 mM stock solution of Z-LR-AMC (*K*_m,Z-LR-AMC_ = 2.37 μM) in DMSO first in a 1:40 ratio with DMSO and then in a 1:10 ratio with 10% DMSO in assay buffer to 0.05 mM. For the enzyme intermediate dilution, the cathepsin K stock solution was first diluted to 1 μg/mL with cathepsin K enzyme buffer and then diluted to 0.1 μg/mL with assay buffer containing 10 mM DTT. The final enzyme concentration was 5 ng/mL at a substrate concentration of 5 μM.

In the case of reversibly inhibiting dipeptide nitriles and reversibly acting dipeptide alkynes, the recorded time courses of the type RFU – RFU_fl_ = *f*(*t*) were analyzed by linear regression. The determined rates, which were obtained as line slopes from linear regression, were reanalyzed by nonlinear regression according to eq II:
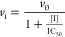
IIwhere *v*_0_ and *v*_i_ are the rates in the absence and presence of the inhibitor, respectively, [I] is the inhibitor concentration, and IC_50_ is the parameter to be fitted.

To obtain equilibrium dissociation constant *K*_i_, the determined IC_50_ values were transformed by applying the Cheng–Prusoff equation (eq III):
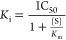
IIIwhere [S] is the employed concentration of the substrate and *K*_m_ is its Michaelis constant.

In the case of irreversibly inhibiting dipeptide alkynes, the recorded time courses of the type RFU – RFU(0) = *f*(*t*) were analyzed by nonlinear regression according to eq IV:
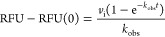
IVwhere *v*_i_ is the initial velocity and *k*_obs_ is the pseudo-first-order rate constant for reaching the final inhibited state; both represent parameters to be fitted.

In general, the obtained *k*_obs_ values were reanalyzed by nonlinear regression according to eq V:
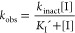
Vwhere *k*_inact_ is the first-order inactivation constant and *K*_I_′ = *K*_I_(1 + [S]/*K*_m_), the apparent kinetic inhibition constant, which equals the inhibitor concentration at which enzyme inaction proceeds at half of the maximum velocity. In most cases, for which the *k*_obs_ values did not reach saturation, reanalysis was performed by linear regression according to eq VI:
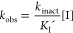
VI

The obtained slopes that equal the apparent second-order inactivation constants *k*_inact_/*K*_I_′ (equal to *k*_obs_/[I]) were transformed into the true values by applying eq VII:
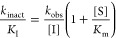
VII

If the determined initial velocities *v*_i_ determined by fitting of eq IV declined systematically with an increasing inhibitor concentration, analysis according to eqs II and III (note the different meanings of *v*_i_ in eqs II and IV) was performed to obtain kinetic dissociation constants *K*_i_.

### Fluorimetric Assay with Viable Cells

Methods for cell culture are specified in the Supporting Information. For the determination of cathepsin B activity on viable cells, an appropriate number of cells (3 × 10^5^ and 2 × 10^5^ cells/mL; which was automatically determined by a CASY cell counter from Innovatis) was added to a 96-well plate and cultured for 24 h in the usual culture medium in an incubator. Subsequently, the cells were carefully washed twice with 200 μL of PBS directly before the measurement.

A 10 mM substrate stock solution of Abz-GIVRAK(Dnp)-NH_2_ in DMSO was diluted with a mixture of 10% DMSO in assay buffer first to 1 mM and then to the desired concentrations of the intermediate dilution (100, 200, 400, 600, 800, and 1000 μM). Cells were incubated with each 160 μL of assay buffer (pretempered to 37 °C), 10 μL of a solution of DTT in assay buffer (10 mM), and 10 μL of a solution of CA-074 in assay buffer (0.2 mM) or 10 μL of assay buffer (control) for each 30 min at 37 °C in an incubator. The reaction was then started by adding 20 μL of substrate intermediate dilution using a multichannel pipet with a dispenser function.

Substrate turnover was monitored by the increase in fluorescence in a Biotek Synergy 4 Hybrid Multi-Mode Microplate Reader (15 min, 37 °C, excitation at 325 nm, emission at 410 nm, Sens100, top-read). All measurement points were recorded as duplicates within three independent experiments.

For the determination of the total protein amount of the cells grown in the 96-well plate per well, 70 μL of the lysis solution (1% SDS in 0.1 M NaOH) was added to each of four wells, and the plate was incubated for 30 min at room temperature on the shaker. Subsequently, the obtained lysates were processed as described in the Supporting Information. Protein determination could be performed only in untreated wells, as cell adhesion in assay buffer decreases significantly over the duration of the assay. As a result, a variable proportion of the cell material is removed with the transfer of the assay buffer.

For inhibitor characterization on viable cells, cells were incubated in a 96-well black plate with 160 μL of assay buffer (pretempered to 37 °C), 10 μL of DTT in assay buffer (10 mM), and 10 μL of a CA-074 solution (0.2 mM in assay buffer) or 10 μL of an inhibitor stock solution in assay buffer containing 10% DMSO for 30 min at 37 °C. Subsequently, the measurement was performed as described above.

### Determination of Chromatographic Hydrophobicity Indices on an Artificial Immobilized Membrane (CHI IAM)

CHI IAM indices were determined as described by Wodtke et al.^153^ (following the procedure published by Valko et al.^154^). An analytical HPLC system from Agilent (1100 Series, Santa Clara, CA) was used employing a Regis IAM PC DD2 column (10 cm × 4.6 cm) as the stationary phase. The mobile phase components were 50 mM ammonium acetate (pH 7.4; A) and acetonitrile (B). Elution was performed in gradient mode (from 0 to 9 min 100% A to 100% B, from 9 to 9.5 min 100% B, and from 9.5 to 10.5 min 100% A). Detection was performed at 254 nm.

### Western Blot Analysis

Cells were washed with ice-cold PBS and lysed in lysis buffer [50 mM Tris-HCl (pH 8.0), 150 mM NaCl, 1% Nonidet P-40, 0.5% sodium desoxycholate, 0.1% SDS, 1 mM PMSF, 5 mM NaF, 1 mM Na_3_VO_4_, and 1 mM DTT]. Samples were then sonicated twice for 7 s with ultrasound (20%, pulsed) and cooled on ice for 5 min between lysis cycles. After centrifugation (15 min at 4 °C and 16000*g*), the clear supernatant was transferred to a new Eppendorf tube and stored on ice or at −70 °C until further use. Protein concentrations in supernatants were determined using the Pierce BCA Protein Assay Kit (Thermo Fisher Scientific) as described in the Supporting Information. Prior to Western blot analysis, equal protein amounts (50 μg) were separated by sodium dodecyl sulfate–polyacrylamide gel electrophoresis (SDS–PAGE) on a 12.5% SDS–polyacrylamide gel and subsequently transferred to a polyvinylidene difluoride (PVDF) membrane (Merck KGaA). For each gel, the PageRuler Plus Prestained Protein Ladder (Thermo Fisher Scientific) was used as the molecular weight ladder standard. For Western blot analysis, PVDF membranes were incubated overnight in Tris-buffered saline with 0.05% Tween 20 with primary anti-cathepsin B (Abcam, 1:500 in 2% BSA), anti-cathepsin K (Abcam, 1:5000 in 5% nonfat dry milk powder), anti-cathepsin L (Abcam, 1:2000 in 1% BSA), anti-cathepsin S (Abcam, 1:5000 in 5% BSA), anti-cystatin S (Santa Cruz Biotechnologies, 1:600 in 2% BSA), or anti-cystatin C (Abcam, 1:1000 in 5% nonfat dry milk powder) antibodies. As secondary antibodies, anti-mouse IgG-POD (Sigma-Aldrich, 1:10000 in 5% nonfat dry milk powder), anti-rabbit IgG-POD (Sigma-Aldrich, 1:5000 in 5% nonfat dry milk powder), or anti-goat IgG-POD (Sigma-Aldrich, 1:5000 in 5% nonfat dry milk powder) antibodies were used. Protein detection was performed with the SuperSignal West Pico Chemiluminescent Substrate or SuperSignal West Femto Maximum Sensitivity Substrate or SuperSignal West Dura Extended Duration Substrate (Thermo Fisher Scientific) using the Bio-Imaging-System MF ChemiBIS 3.2 (Biostep).

### Molecular Modeling

The three-dimensional crystal structures of cathepsin B [Protein Data Bank (PDB) entry 1GMY, 1.9 Å],^86^ cathepsin S (PDB entry 1MS6, 1.9 Å),^131^ and cathepsin L (PDB entry 2YJC, 1.1 Å)^118^ used for our calculations were prepared in Maestro version 13.3^155^ with Protein Preparation Wizard,^156^ including an optimization step of hydrogen bond assignments using ProtAssign from Schrödinger. Compounds **1b**, **2b**, **2c**, **2e**, **2f**, **2h**–**k**, **35a**, and **53k** were prepared with LigPrep.^157^ Epik was used to generate the ionization state at pH 7.0 ± 2.0.^158,159^ The OPLS4 force field was employed.^160^

Covalent docking of selected compounds to cathepsins B, S, and L was performed with CovDock version 1.3^161^ in standard precision mode using the OPLS4 force field. Grid boxes were centered for cathepsin B (*x*, 35.0; *y*, 32.2; *z*, 33.0), cathepsin S (*x*, 48.1; *y*, 29.4; *z*, 60.2), and cathepsin L (*x*, 8.9; *y*, 36.1; *z*, 19.4) with inner and outer boxes of 10 Å × 10 Å × 10 Å and 30 Å × 30 Å × 30 Å, respectively. The sulfur atom of catalytic residue Cys29 in cathepsin B and Cys25 in cathepsins L and S was defined as the reaction site for the formation of a covalent bond with either the nitrile or propargyl group of the selected inhibitor. A refinement of binding poses was carried out with a minimization radius of 3.0 Å. Docking results were ranked according to their Prime energy and docking score, and they were visualized in Maestro version 13.3 (Schrödinger).

## Supporting Information Available

Details of the enzymatic assays and solubility of inhibitor compounds in aqueous medium; additional results and details of the kinetic characterization and inhibition type of dipeptide nitrile **1a**; schematic representation of the jump-dilution experiment; comparison of selectivity profiles between nitriles and alkynes; additional docking poses for selected inhibitors with cathepsins B, L, and S; overview of the determined kinetic parameters and CHI IAM for inhibitor compounds; methods for HPLC analysis and purification; proof of the enantiomeric purity of Garner’s aldehyde by Mosher analysis; additional aspects of the X-ray crystal structure of compound **48**; methods of cell culture and Western blot expression analysis for different tumor cell lines; experimental procedures and analytical data for synthesized compounds; and NMR spectra and HPLC chromatograms of inhibitor compounds (PDF)
Inhibitor structures in SMILES string notation with tabulated inhibition data (CSV)

## Dedication

Dedicated to Prof. Dr. Michael Gütschow on the occasion of his 65th birthday.

## Abbreviations

Abz: 2-aminobenzoyl
AMC: 7-amino-4-methylcoumarin
CA-074: [(2*S*,3*S*)-3-(propylcarbamoyl)oxirane-2-carbonyl]-l-isoleucyl-l-proline
CHI: chromatographic hydrophobicity index
DIPEA: diisopropylethylamine
Dnp: 2,4-dinitrophenyl
E-64: [(2*S*,3*S*)-3-(carboxy)oxirane-2-carbonyl]-l-leucine-(4-guanidinobutyl)amide
ECM: extracellular matrix
HOBt: 7-hydroxybenzotriazole
IAM: immobilized artificial membrane
iBCF: isobutylchloroformate
MM: molecular mechanics
n.i.: no inhibition
NMM: *N*-methylmorpholine
PET: positron emission tomography
PMSF: phenylmethanesulfonyl fluoride
POD: peroxidase
PVFDF: polyvinylidene difluoride
PyBOP: benzotriazol-1-yloxytripyrrolidinophosphonium hexafluorophosphate
QM: quantum mechanics
RFU: relative fluorescence units
SEM: standard error of the mean
SPECT: single-photon-computed tomography
TEA: triethylamine
