# Supplementary material for: Dipeptide-Derived Alkynes as Potent and Selective Irreversible Inhibitors of Cysteine Cathepsins
Source: J Med Chem. 2023 Mar 3;66(6):3818–51. doi: 10.1021/acs.jmedchem.2c01360 (PMC10041539; doi:10.1021/acs.jmedchem.2c01360)
Supplement: Supplementary file 1 — jm2c01360_si_001.pdf [file jm2c01360_si_001.pdf]

Supporting Information

Dipeptide-derived alkynes as potent and selective irreversible inhibitors of cysteine cathepsins

*Lydia Behring[a,d], Gloria Ruiz-Gómez[b], Christian Trapp[a], Maryann Morales[a], Robert Wodtke[a], Martin Köckerling[c], Klaus Kopka[a,d], M. Teresa Pisabarro[b], Jens Pietzsch[a,d], and Reik Löser[a,d]**

[a] Helmholtz-Zentrum Dresden-Rossendorf, Institute of Radiopharmaceutical Cancer Research, Bautzner Landstraße 400, 01328 Dresden, Germany

[b] BIOTEC, Technische Universität Dresden, Tatzberg 47-51, 01307 Dresden, Germany

[c] Institute of Chemistry, University of Rostock, Albert-Einstein-Straße 3a, 18059 Rostock, Germany

[d] Technische Universität Dresden, Faculty of Chemistry and Food Chemistry, Mommsenstraße 4, 01069 Dresden, Germany

*Corresponding author, email: r.loeser@hzdr.de

Content

Purity of the employed enzymes 3

Proof of enzyme stability 3

K_m determination of the used substrates 8

Solubility of the inhibitor compounds in aqueous media 9

Inhibition of cathepsin B by dipeptide nitrile **1a** 19

Determination of inhibition type for dipeptide nitrile **1a** at cathepsin B 20

Weak and reversible inhibition of cathepsin B by dipeptide alkyne **28** 24

Proof of irreversible inhibition by jump dilution 25

Comparison of selectivity profiles of dipeptide nitriles and the corresponding alkynes 26

Influence of varying *meta* substituents at *N*-benzoyl dipeptide alkynes on inhibition of cathepsins B, S, L and K 27

Molecular models of cathepsin (B/S/L)-inhibitor complexes for selected compounds obtained by covalent docking *in silico* 28

Overview of determined kinetic parameters for inhibitor compounds 30

CHI IAM values of inhibitor compounds 34

Methods for cell culture 35

Western Blots with corresponding loading controls... 35

Investigation of intra- and extracellular Cathepsin B activity in different tumor cell lines 38

Methods for HPLC analysis and purification 40

Proof of enantiomeric purity of Garner's aldehyde by Mosher analysis.............. 42

Xray crystal structure of Boc-serine-derived alkyne **48** 46

Experimental procedures and analytical data for synthesized compounds 47

NMR spectra of inhibitor compounds.. 142

Purities and chromatograms of inhibitor compounds 177

References for Supporting Information 193

Purity of the employed enzymes

The enzyme solution may contain different enzyme forms, such as different post-translational modification forms or partially digested products, which can show different catalytic efficiency and thus affect the kinetic parameters. Therefore, the purity of the enzyme solutions used was checked by Western blot (Figure S1). The procedure Western Blot analysis is described in the Experimental Section in the main text.

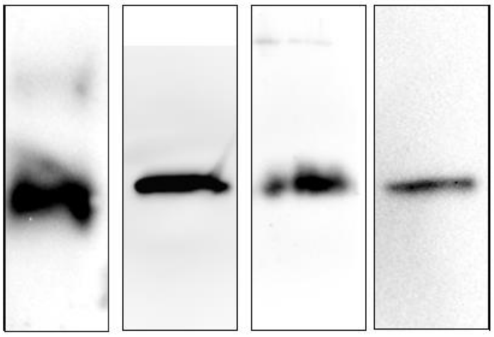

Figure S1: Western blot for the enzymes used in the activity assay.

Proof of enzyme stability

As the enzyme concentration can decline during the time of the assay due denaturation or -in the case of proteases – autocatalytic degradation, all four cathepsins were subjected to the Selwyn test, to prove their stability during assay conditions. Therefore, the assay is performed under identical conditions with different enzyme concentration. Under these conditions, the formation of the product is proportional to the enzyme concentration used ($[P] = f([E] \times t)$). If the latter is constant over the duration of the assay, the conversion curves will coincide when plotting the measured fluorescence intensities against $[E] \times t$, respectively. If the enzyme concentration changes, [E] becomes a time-dependent function and separate curves are obtained.[1]

Cathepsin B

For testing the stability of cathepsin B under assay conditions, a 5 mM Abz-GIVRAK(Dnp)-NH_2 stock solution in DMSO was diluted 1:10 with assay buffer (100 mM sodium phosphate buffer [pH 6.0], 100 mM NaCl, 5 mM EDTA, 0.01% Brij) containing 10% DMSO. In a black 96-well plate, 20 µL of substrateintermediate-dilution and in 170 µL of assay buffer were placed and incubated for 15 min at 37°C. The cathepsin B stock solution was diluted with enzyme buffer (20 mM sodium acetate [pH 5.0], 1 mM EDTA, 10 mM DTT) containing 5 mM DTT first to 0.054 mg/mL and then to the desired intermediate dilutions and pre-activated for 5 min at room temperature. (The DTT concentration as well as the pre-activation temperature were increased for the final assay conditions, as this significantly improved the reproducibility of the readings).

The reaction was started by adding 10 µL of enzyme solution to the substrate solution. The final enzyme concentrations were 100 to 250 ng/mL.

Substrate turnover was monitored by the increase in fluorescence in the Synergy 4 Hybrid Multi-Mode Microplate Reader from BIOTECH (15 min, 37°C, Exc325, Em410, Sens80, top-read). All measurement points were recorded as duplicates. Analysis was performed in Prism (GraphPad Software, Inc., version 5.02).

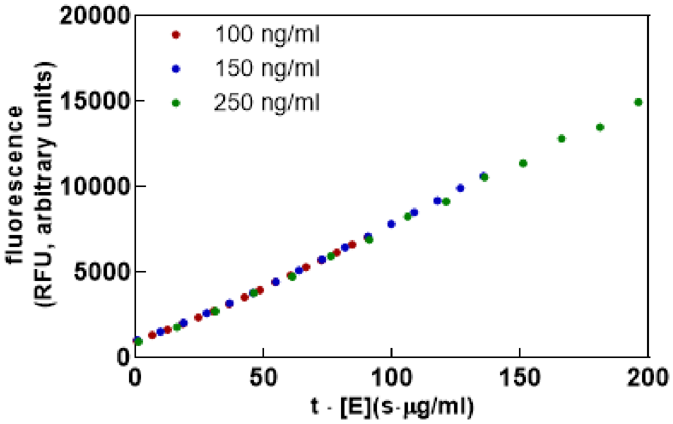

Figure S2: Selwyn test for the determination of cathepsin B stability under assay conditions. The determination was performed with 50 µM Abz-GIVRAK(Dnp)-NH2, 1% DMSO, and 0.25 mM DTT at 37°C.

As the turnover curves coincide (Figure S2), the enzyme can be judged as stable for the investigated period and under the applied conditions. For further experiments, pre-incubation of cathepsin B was carried at 37°C instead of room temperature.

Cathepsin S

For testing the stability of cathepsin S under assay conditions, a 10 mM Z-VVR-AMC stock solution in DMSO was diluted 1:25 with assay buffer containing 10% DMSO and warmed to 37°C. In a black 96-well plate with a transparent bottom, 10 µL of assay buffer containing 10% DMSO and in 160 µL of assay buffer were placed and tempered at 37°C for 10 min. The cathepsin S stock solution was diluted with enzyme buffer (0.05 mM sodium phosphate [pH 6.5], 50 mM NaCl, 2 mM EDTA, 0.01% Triton X-100, 5 mM DTT) containing 5 mM DTT first to 0.01 mg/mL and then to the desired intermediate dilutions and pre-activated for 5 min at 37°C. Then, 10 µL of enzymeintermediate-dilution was added to each buffer. The reaction was started by adding 20 µL of substrate solution. The final enzyme concentrations were 10 to 250 ng/mL.

Substrate turnover was monitored by the increase in fluorescence using the BIOTEK Synergy 4 Hybrid Multi-Mode Microplate Reader (15 min, 37°C, Exc360/40, Em460/40, sensitivity 45, top-read). All measurement points were recorded as duplicates. Analysis was performed in Prism (GraphPad Software, Inc., version 5.02).

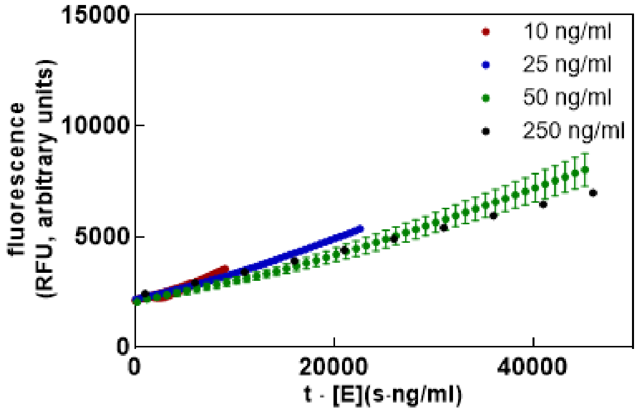

Figure S3: Selwyn test for the determination of cathepsin S stability under assay conditions. The determination was performed with 40 µM Z-VVR AMC, 1.5% DMSO and 0.25 mM DTT at 37°C.

The curves do not exactly coincide (Figure S3), which is probably due to the time delay in substrate addition and protracted mixing. The slight increase in the conversion could indicate incomplete enzyme activation prior to measurement. Deactivation of the enzyme, on the other hand, would lead to a decrease in the turnover rate. Since Schmitz *et al.* described a pre-incubation of 60 min at 37°C for the cathepsin S activity assay,[2] the enzyme activity was investigated as a function of the pre-incubation time.

For this purpose, 20 µL of substrate intermediate-dilution and 170 µL of assay buffer were placed in a 96-well plate and incubated for 20 min at 37°C. The cathepsin S stock solution was diluted to 0.5 µg/mL with enzyme buffer containing 5 mM DTT and the enzyme solution was pre-activated under various conditions. The reaction was then started by adding 10 µL of enzyme solution. The substrate conversion was measured as described above.

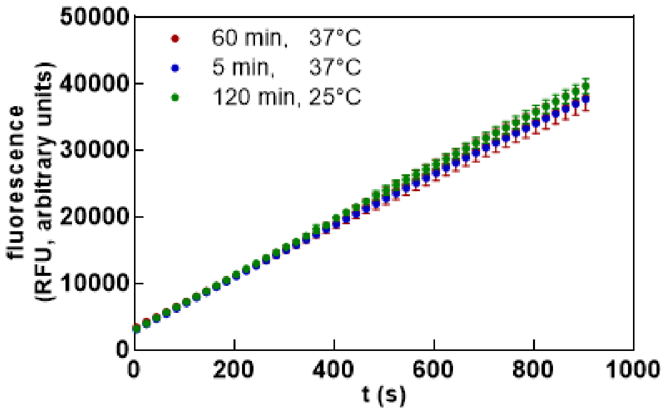

Figure S4: Cathepsin S activity as a function of pre-incubation time and temperature. The determination was performed with 40 µM Z-VVR-AMC, 1% DMSO and 0.25 mM DTT at 37°C.

As the enzyme activity was neither higher nor lower after 60 min of preincubation than after 5 min at 37°C (Figure S4), the enzyme can be considered fully activated after 5 min and is also stable for 60 min at 37°C. The curve for 120 min shows a minimally larger increase, although this deviation is probably due to pipetting inaccuracy rather than higher activity.

Cathepsin L

For testing the stability of cathepsin L under assay conditions, a 20 mM Z-FR-AMC stock solution in DMSO was diluted 1:20 with assay buffer containing 10% DMSO. In a black 96-well plate, 20 µL of substrate intermediate-dilution and 10 µL of assay buffer containing 10% DMSO were added to 160 µL of assay buffer and incubated for 15 min at 37°C. The cathepsin L stock solution was diluted with enzyme buffer (20 mM sodium malonate [pH 5.5], 400 mM NaCl, 1 mM EDTA) first to 0.052 mg/mL and then with assay buffer containing 10 mM DTT to the desired intermediate-dilutions. The enzyme was pre-activated for 5 min at 37°C. The reaction was started by adding 10 µL of enzyme solution to the substrate solution. The final enzyme concentrations were 25 to 100 ng/mL.

Substrate turnover was monitored by the increase in fluorescence in the BIOTEK Synergy 4 Hybrid Multi-Mode Microplate Reader (15 min, 37°C, Exc360/40, Em460/40, sensitivity 45, bottom-read). All measurement points were recorded as duplicates. Analysis was performed in Prism (GraphPad Software, Inc., version 5.02).

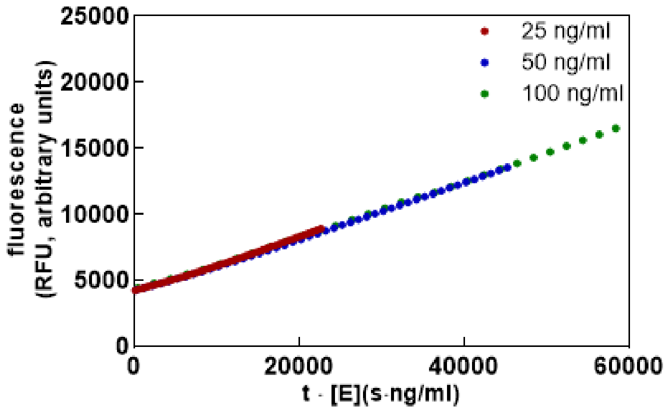

Figure S5: Selwyn test for the determination of cathepsin L stability under assay conditions. The determination was performed with 100 µM Z-FR-AMC, 1.5% DMSO, and 0.5 mM DTT at 37°C.

As the substrate conversion curves completely coincide, the enzyme can be judged stable under the chosen conditions.

Cathepsin K

For testing the stability of cathepsin K under assay conditions, a 20 mM Z-LR-AMC stock solution in DMSO was diluted 1:50 with assay buffer containing 10% DMSO. In a black 96-well plate, 20 µL of substrate intermediate-dilution was added to 170 µL of assay buffer and incubated for 15 min at 37°C. The cathepsin K stock solution was diluted with enzyme buffer (50 mM sodium citrate [pH 5.0], 100 mM NaCl, 1 mM EDTA, 0.01% CHAPS, 5 mM DTT) first to 0.001 mg/mL and then with assay buffer containing 10 mM DTT to the desired intermediate dilutions. The enzyme was pre-activated for 5 min at 37°C. The reaction was started by adding 10 µL of enzyme solution to the substrate solution. The final enzyme concentrations were 1 to 20 ng/mL.

Substrate turnover was monitored by the increase in fluorescence in the BIOTEK Synergy 4 Hybrid Multi-Mode Microplate Reader (15 min, 37°C, Exc360/40, Em460/40, sensitivity 45, bottom-read). All measurement points were recorded as duplicates. The analysis was performed in Prism (GraphPad Software, Inc., version 5.02).

Initially, measurements were performed according to Schmitz *et al.* in cathepsin K assay buffer containing 100 mM sodium citrate (pH = 5.0), 100 mM NaCl, 1 mM EDTA, and 0.01% CHAPS and a DMSO concentration of 1%.

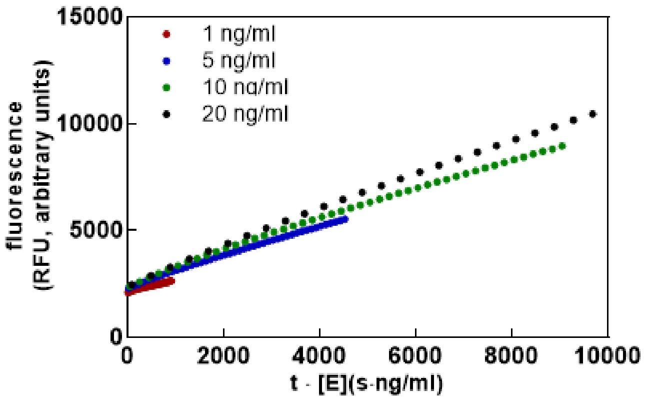

Figure S6: Selwyn test for the determination of cathepsin K stability in assay buffer according to Schmitz *et al*. The determination was performed with 40 µM Z-LR-AMC, 1% DMSO, and 0.5 mM DTT at 37°C in cathepsin K assay buffer according to SCHMITZ et al. (pH = 5.0).[2]

As the enzyme activity is obviously declining over time (Figure S6), the experiment was repeated in cathepsin B assay buffer containing 1.5% DMSO, in which cathepsin K revealed stable (Figure S7).

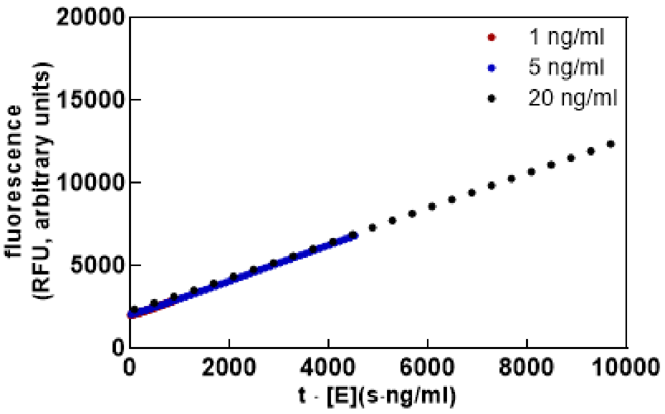

Figure S7: Selwyn assay for the determination of cathepsin K stability in standard assay buffer. The determination was performed with 40 µM Z-LR-AMC, 1.5% DMSO and 0.5 mM DTT at 37°C in the assay buffer as used for cathepsin B, S and L.

K_m determination of the used substrates

For K_m determination, the respective substrate stock solution in DMSO was diluted to the intermediate dilutions with assay buffer containing 10% DMSO. In a black 96-well plate with a transparent bottom 20 µL of substrate intermediate-dilution and 10 µL of assay buffer containing 10% DMSO were added to 160 µL of assay buffer and incubated for 20 min at 37°C. The reaction was started by adding 10 µL of enzyme solution to the substrate solution.

Substrate turnover was monitored by the increase in fluorescence in the BIOTEK Synergy 4 Hybrid Multi-Mode Microplate Reader (15 min, 37°C, Exc360/40, Em410/40, bottom-read). All measurement points were recorded as duplicates; each measurement was performed as a triplicate determination. Analysis was performed in Prism (GraphPad Software, Inc., version 5.02).

Cathepsin B

A 100 mM Z-RR-AMC stock solution in DMSO was diluted to 1 - 10 mM intermediate dilutions with assay buffer (100 mM sodium phosphate buffer [pH 6.0], 100 mM NaCl, 5 mM EDTA, 0.01% Brij) containing 10% DMSO. For the enzyme intermediate-dilution, the cathepsin B stock solution was first diluted to 54.44 µg/mL with cathepsin B enzyme buffer (20 mM sodium acetate [pH 5.0], 1 mM EDTA, 10 mM DTT) and then diluted to 0.5 µg/mL with assay buffer containing 10 mM DTT. The cathepsin B intermediate-dilution was pre-activated for 5 min at 37°C in a water bath. The final enzyme concentration was 25 ng/mL at a substrate concentration of 0 - 1000 µM. The measurement was recorded with a sensitivity of 45.

A Michaelis constant of K_m (Z-RR-AMC, 1.5% DMSO) = 302.0 ± 24.25 µM was determined.

Cathepsin S

A 10 mM Z-VVR-AMC stock solution in DMSO was diluted to 100 - 400 µM intermediate dilutions with assay buffer containing 10% DMSO. For the enzyme intermediate-dilution, the 0.1 mg/mL cathepsin S stock solution was first diluted to 1 µg/mL with cathepsin S enzyme buffer (0.05 mM sodium phosphate [pH 6.5], 50 mM NaCl, 2 mM EDTA, 0.01% Triton X-100, 5 mM DTT) and then diluted to 0.05 µg/mL with assay buffer containing 10 mM DTT. The cathepsin S intermediate-dilution was pre-activated for 5 min at 37°C in a water bath. The final enzyme concentration was 2.5 ng/mL at a substrate concentration of 10-40 µM. The measurement was recorded with a sensitivity of 45.

A Michaelis constant of K_m (Z-VVR-AMC, 1.5% DMSO) = 19.16 ± 1.23 µM was determined.

Cathepsin L

A 20 mM Z-FR-AMC stock solution in DMSO was diluted to 5 - 300 µM intermediate dilutions with assay buffer containing 10% DMSO. For the enzyme intermediate-dilution, the cathepsin L stock solution was first diluted to 55.25 µg/mL with cathepsin L enzyme buffer (20 mM sodium

malonate [pH 5.5], 400 mM NaCl, 1 mM EDTA) and then diluted to 1 µg/mL with assay buffer containing 10 mM DTT. The cathepsin L intermediate-dilution was pre-activated for 5 min at 37°C in a water bath. The final enzyme concentration was 25 ng/mL at a substrate concentration of 0.5-30 µM. The measurement was recorded with a sensitivity of 60.

A Michaelis constant of *K*m (Z-FR-AMC, 1.5% DMSO) = 3.05 ± 0.23 µM was determined.

Cathepsin K

A 20 mM Z-LR-AMC stock solution in DMSO was diluted to 20 - 600 µM intermediate dilutions with assay buffer containing 10% DMSO. For the enzyme intermediate-dilution, the cathepsin K stock solution was first diluted to 1 µg/mL with cathepsin K enzyme buffer (50 mM sodium citrate [pH 5.0], 100 mM NaCl, 1 mM EDTA, 0.01% CHAPS, 5 mM DTT) and then diluted to 0.1 µg/mL with assay buffer containing 10 mM DTT. The cathepsin K intermediate-dilution was pre-activated for 5 min at 37°C in a water bath and then the reaction was started by adding 10 µL of enzyme solution to the substrate inhibitor solution. The final enzyme concentration was 5 ng/mL at a substrate concentration of 2 - 60 µM. The measurement was recorded with a sensitivity of 45.

A Michaelis constant of K_m (Z-LR-AMC, 1.5% DMSO) = 2.37 ± 0.10 µM was determined.

Solubility of the inhibitor compounds in aqueous media

For solubility testing, a 10 mM inhibitor stock solution in DMSO was prepared, which was diluted with a 10% DMSO solution in assay buffer to the respective desired concentration as intermediate dilutions (20 times the final concentration in the assay in each case). In a flat-bottomed black 96-well plate, 10 µL of inhibitor intermediate-dilution and 20 µL of assay buffer containing 10% DMSO in 170 µL of assay buffer were added and incubated for 20 min at 37°C. Subsequently, the absorbance of the solutions was measured in a BIOTEK Synergy 4 Hybrid Multi-Mode Microplate Reader (300 - 700 nm). All measurement points were recorded as duplicates. The analysis was performed in Prism (GraphPad Software, Inc., version 5.02). For non- or incompletely dissolved substances, the absorbance in the wavelength range > 600 nm does not go back to zero due to scattering effects.

a) Scheme 1 compounds

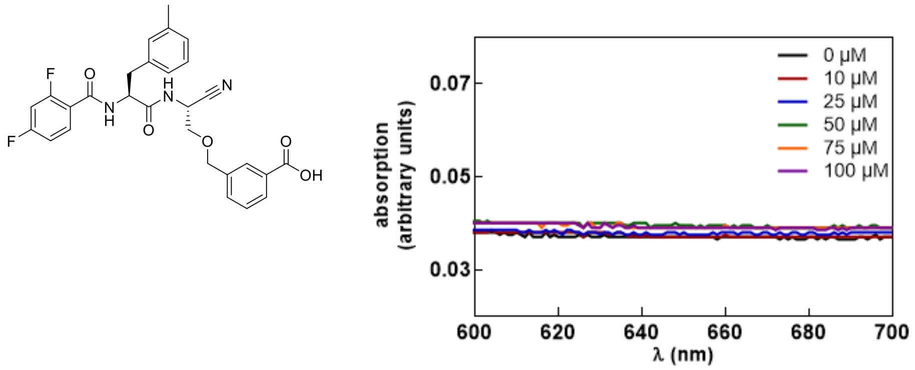

Figure S8: Solubility determination of compound **1a**

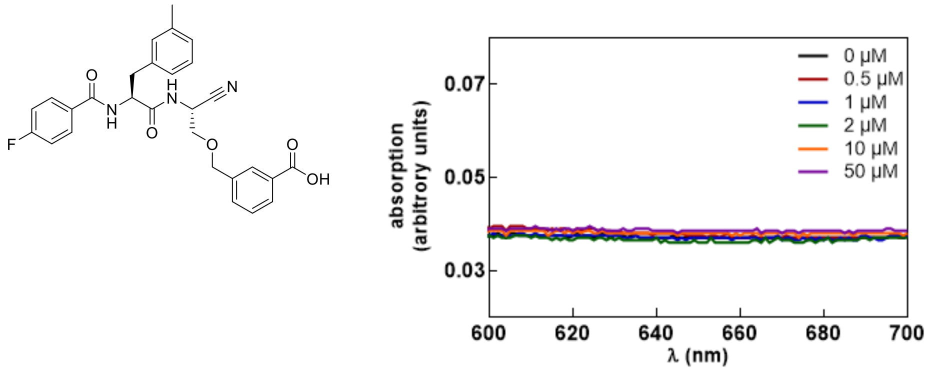

Figure S9: Solubility determination of compound **1b**

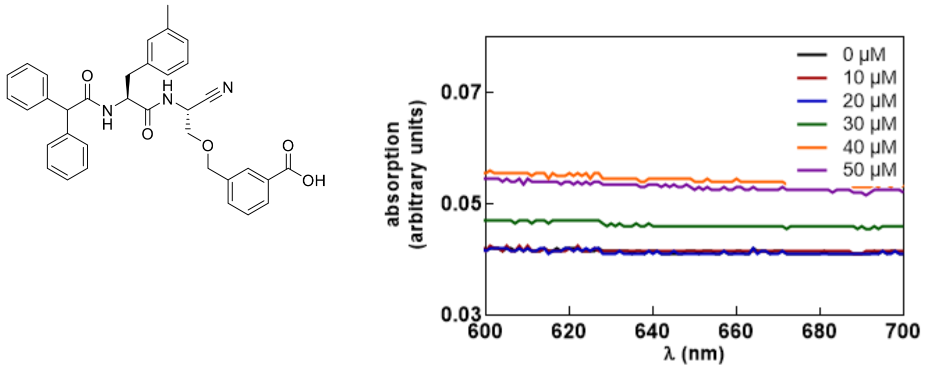

Figure S10: Solubility determination of compound **1c**

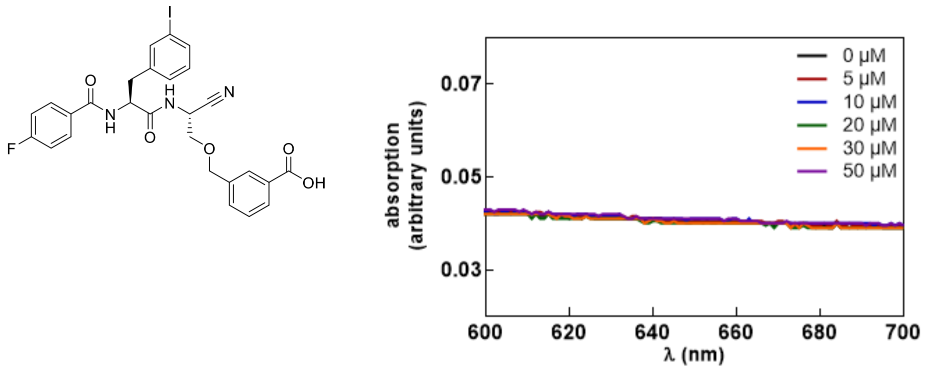

Figure S11: Solubility determination of compound **1d**

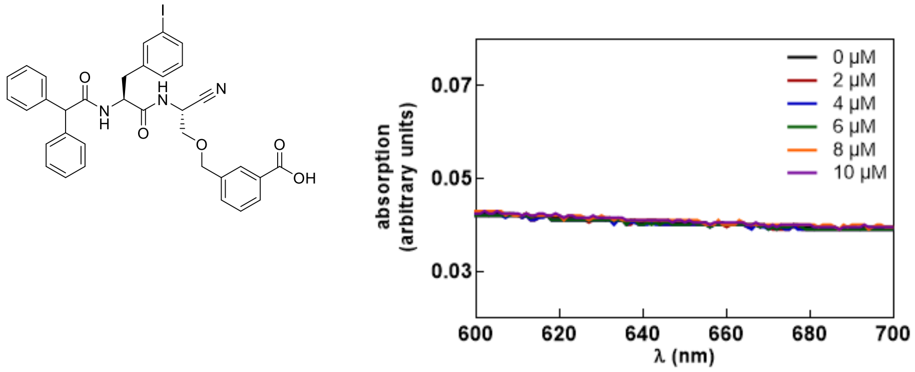

Figure S12: Solubility determination of compound **1e**

b) Scheme 2 compounds

The solubility of compound **18** obtained as diastereomeric mixture was not determined because its solubility behavior was expected to be similar compared to stereochemically pure compound **2a**.

c) Scheme 3 compounds

The solubility of compound **28** was not determined because its solubility behavior was expected to be similar compared to its alkyne analogue **35a**.

d) Scheme 4 compounds

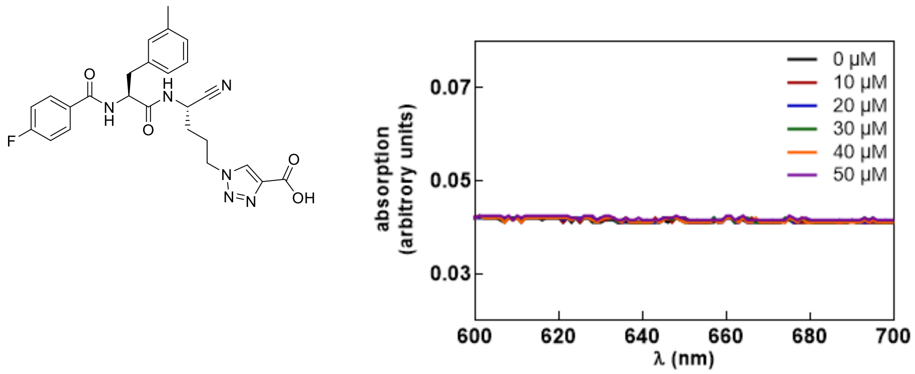

Figure S13: Solubility determination of compound **35a**

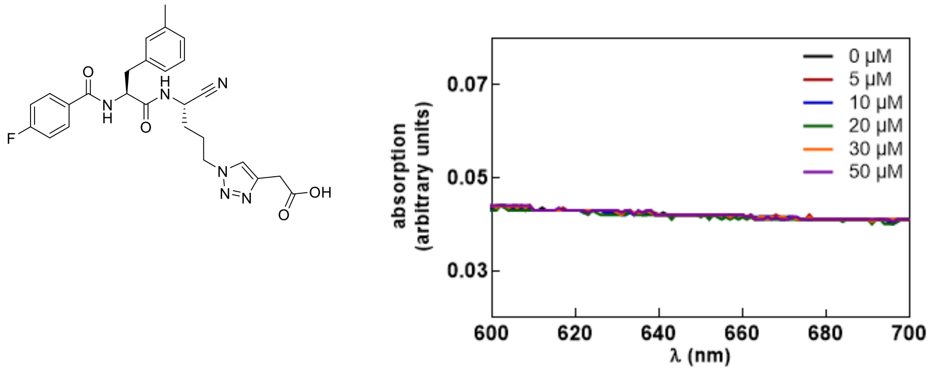

Figure S14: Solubility determination of compound **35b**

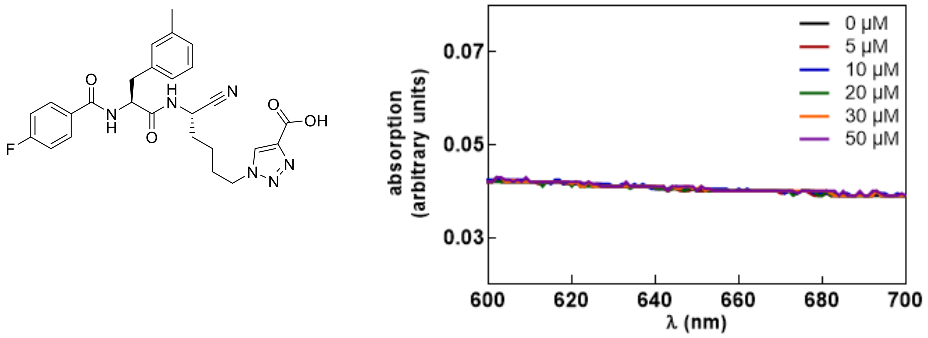

Figure S15: Solubility determination of compound **35c**

e) Scheme 5 compounds

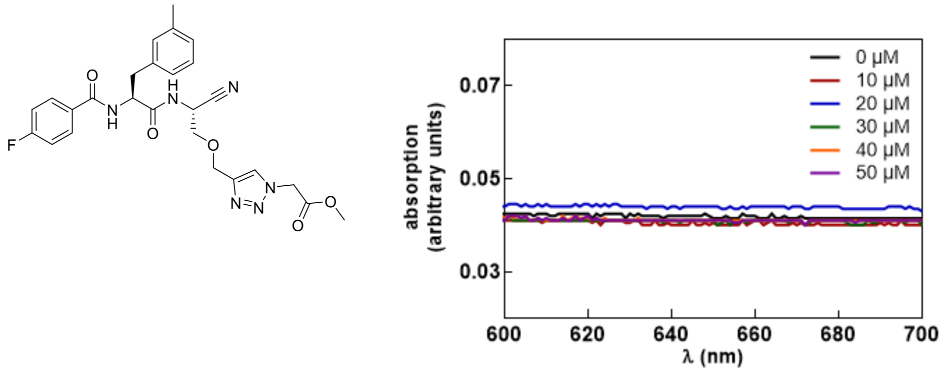

Figure S16: Solubility determination of compound **42**

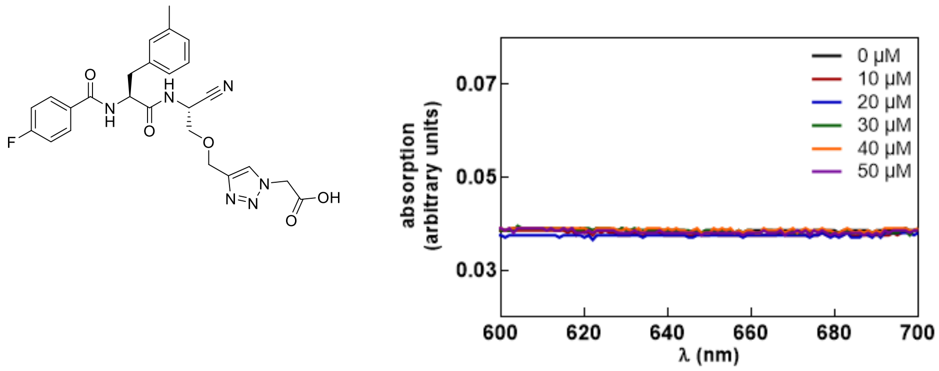

Figure S17: Solubility determination of compound **43**

f) Scheme 6 compounds

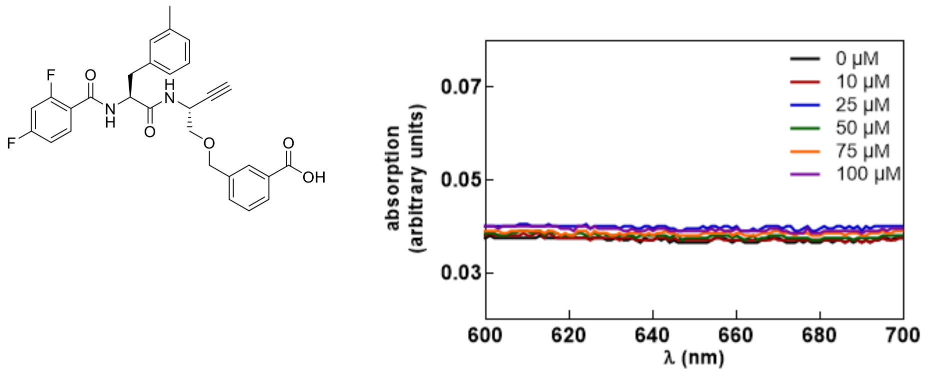

Figure S18: Solubility determination of compound **2a**

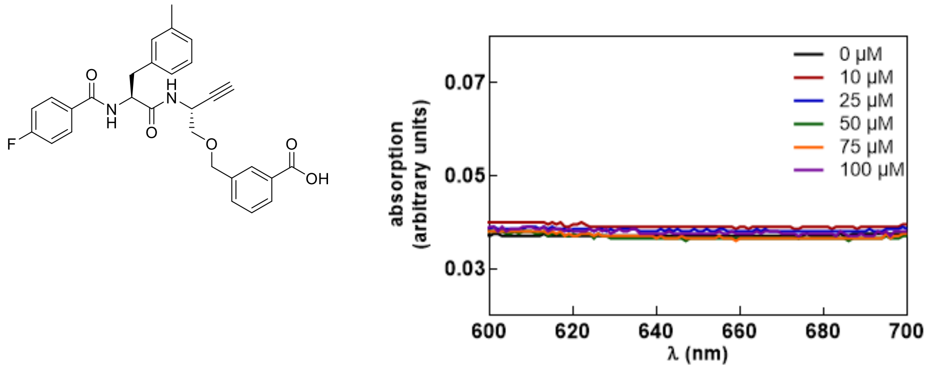

Figure S19: Solubility determination of compound **2b**

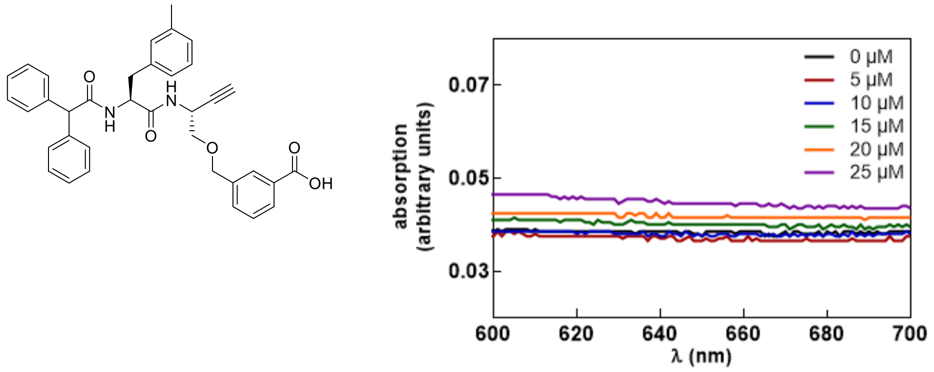

Figure S20: Solubility determination of compound **2c**

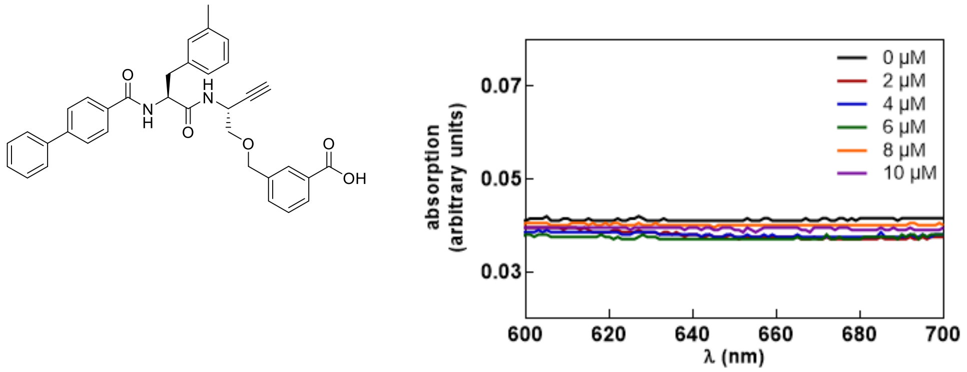

Figure S21: Solubility determination of compound **2d**

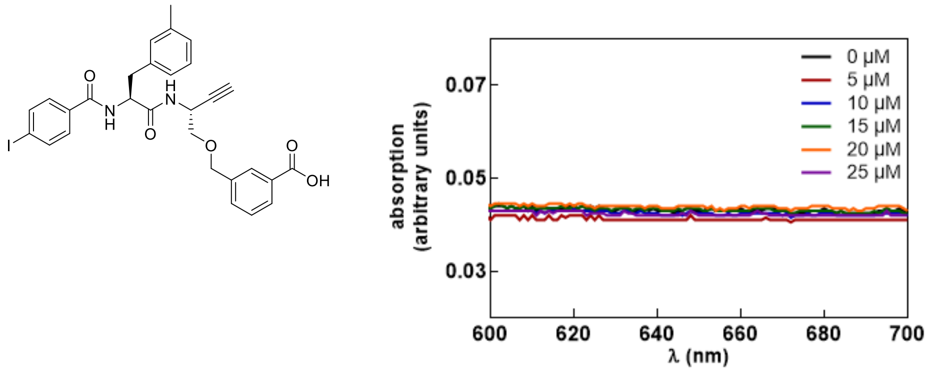

Figure S22: Solubility determination of compound **2e**

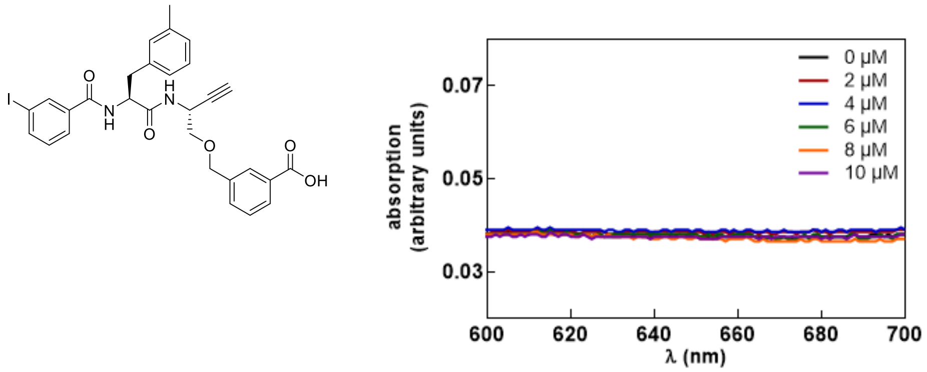

Figure S23: Solubility determination of compound **2f**

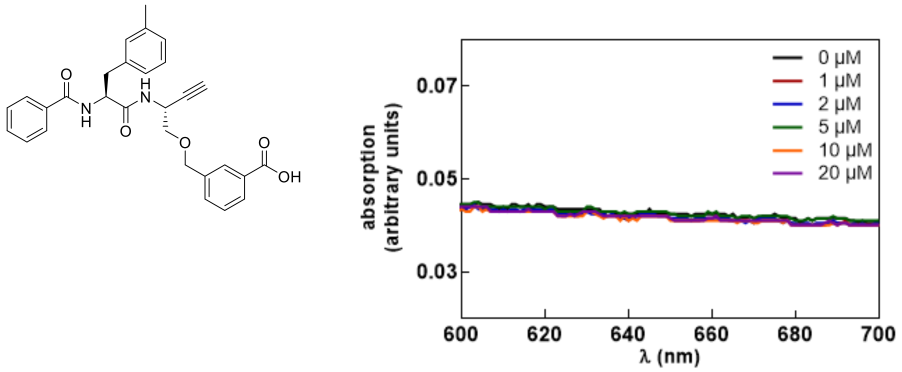

Figure S24: Solubility determination of compound **2g**

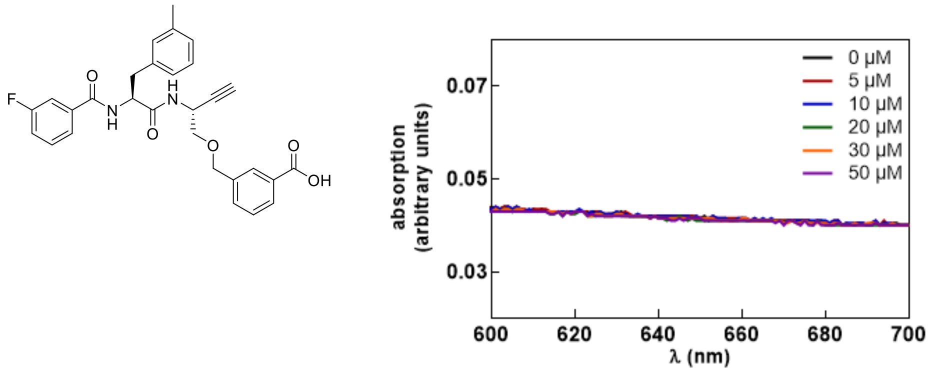

Figure S25: Solubility determination of compound **2h**

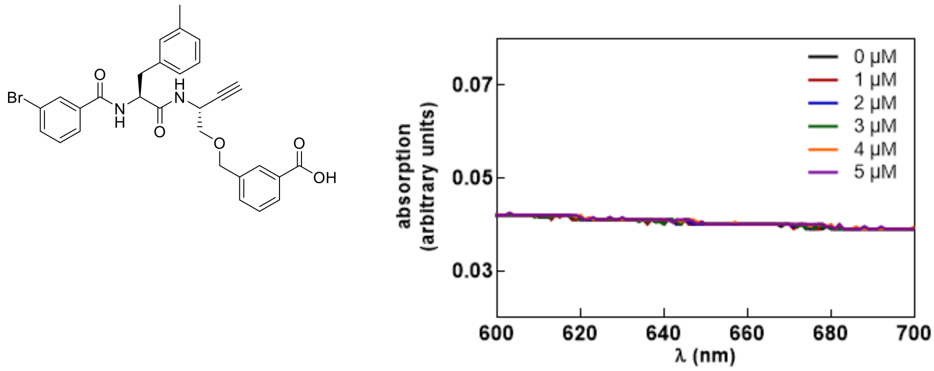

Figure S26: Solubility determination of compound **2i**

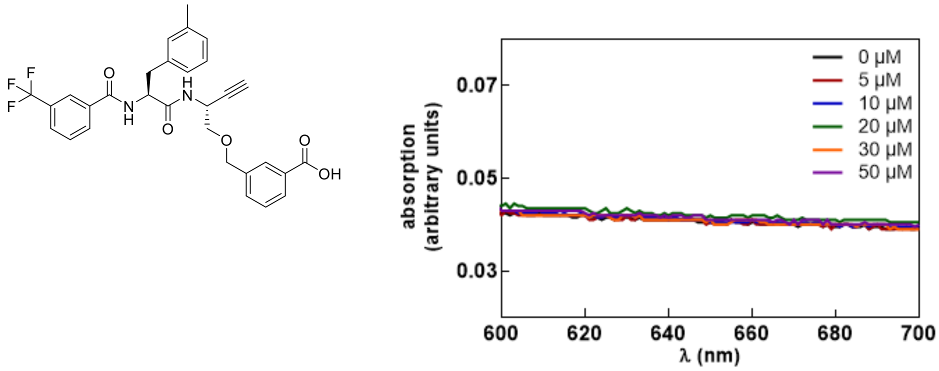

Figure S27: Solubility determination of compound **2j**

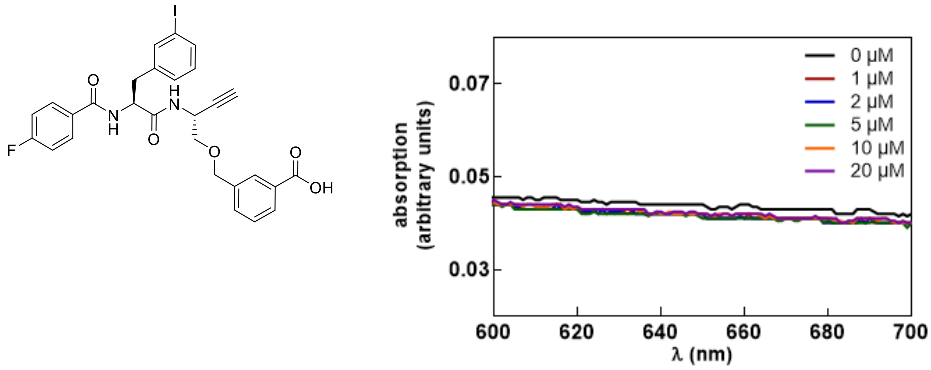

Figure S28: Solubility determination of compound **2k**

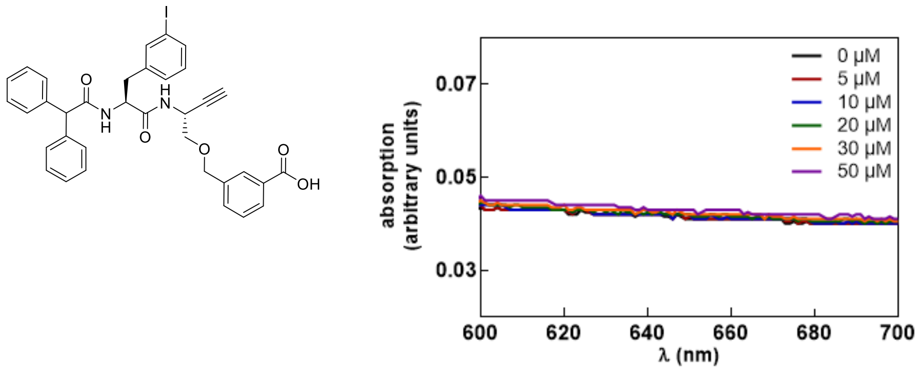

Figure S29: Solubility determination of compound **2l**

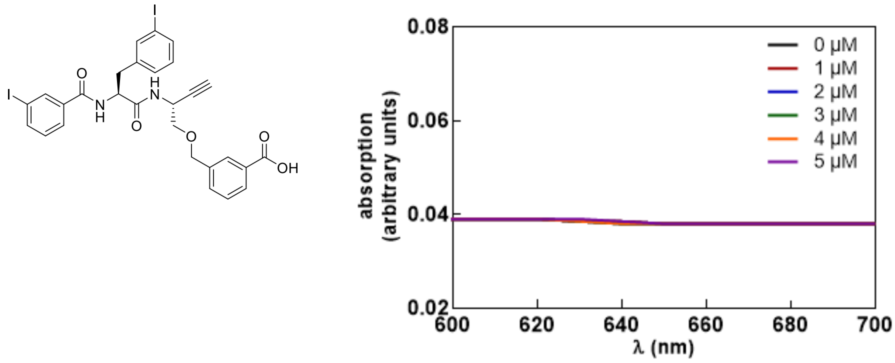

Figure S30: Solubility determination of compound **2m**

g) Scheme 7 compounds

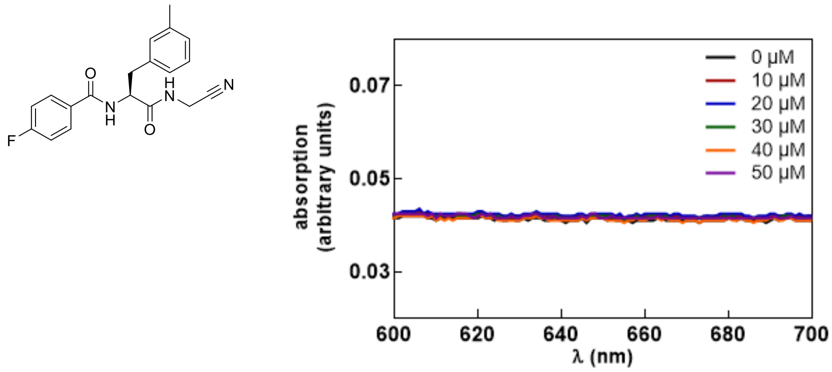

Figure S31: Solubility determination of compound **56a**

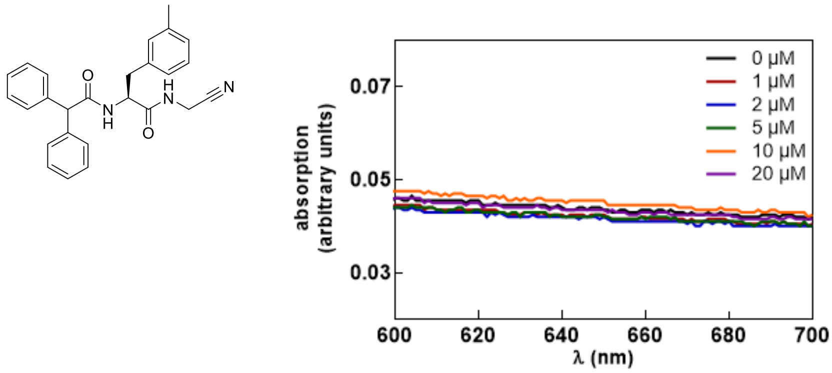

Figure S32: Solubility determination of compound **56b**

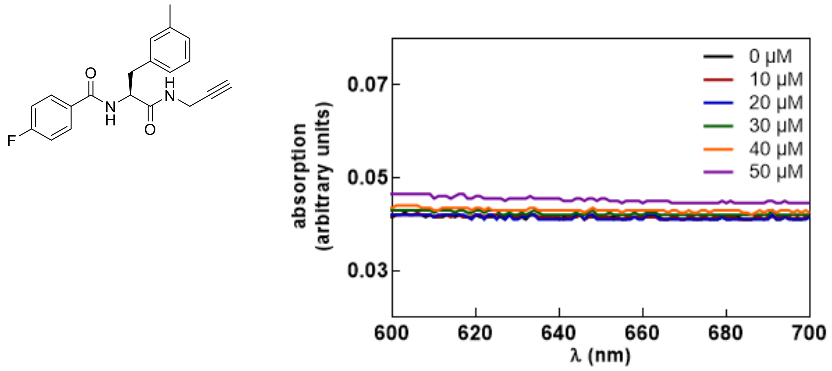

Figure S33: Solubility determination of compound **56c**

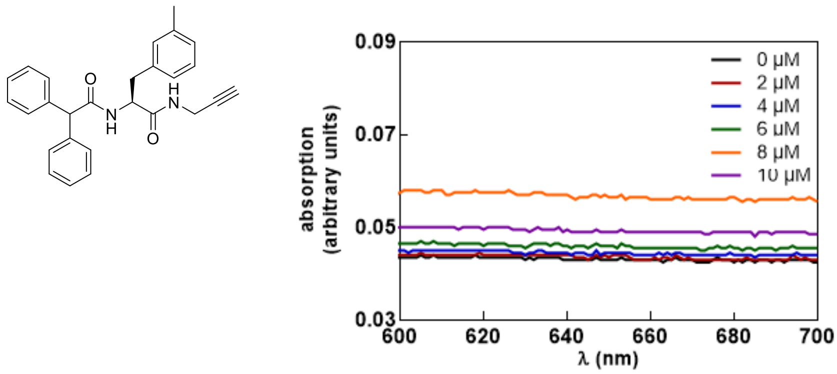

Figure S34: Solubility determination of compound **56d**

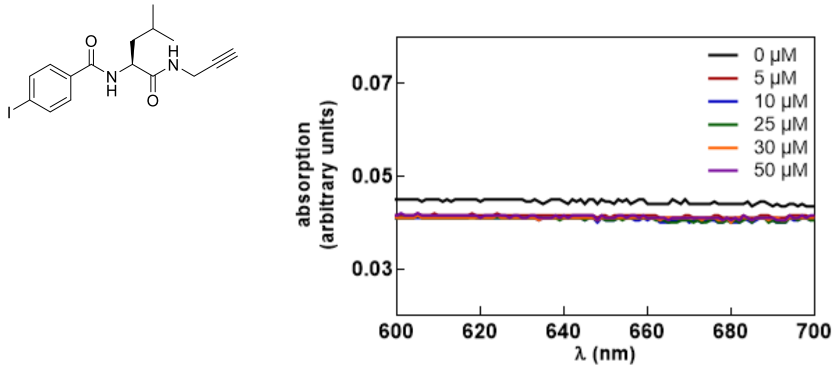

Figure S35: Solubility determination of compound **56e**

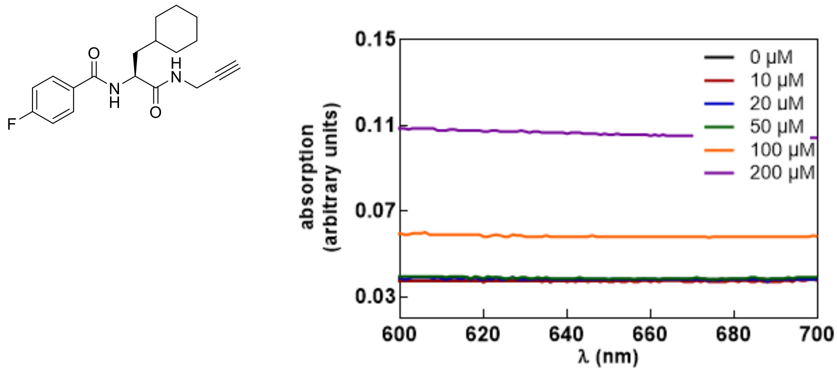

Figure S36: Solubility determination of compound **56f**

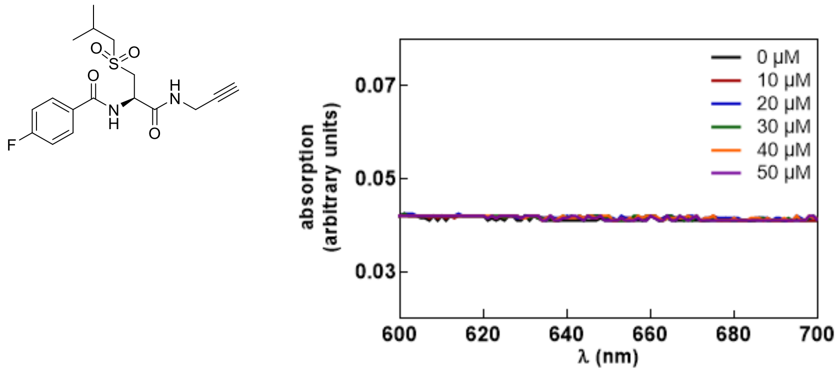

Figure S37: Solubility determination of compound **62**

h) Dipeptide alkynes 53k, 63 und 64 with esterified carboxylic group

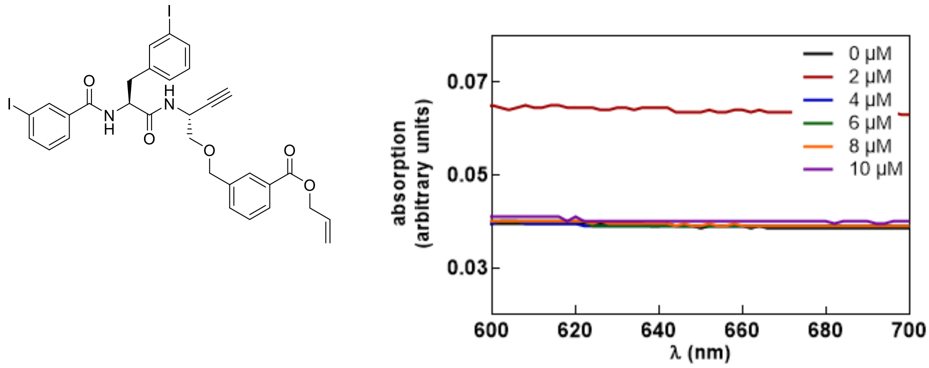

Figure S38: Solubility determination of compound **53k**

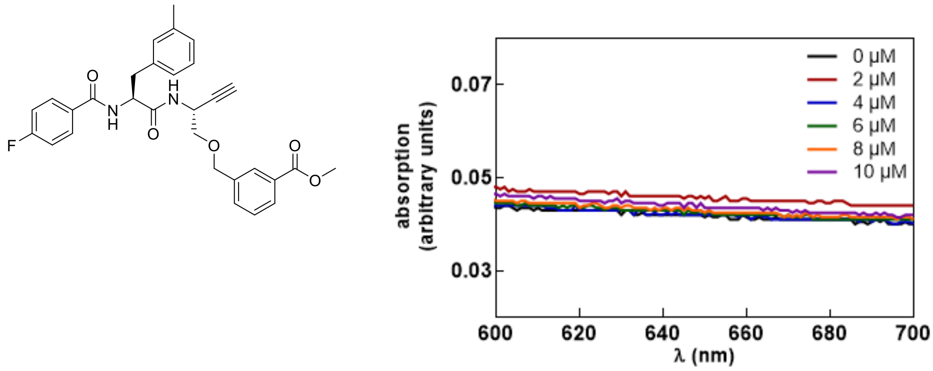

Figure S39: Solubility determination of compound **63**

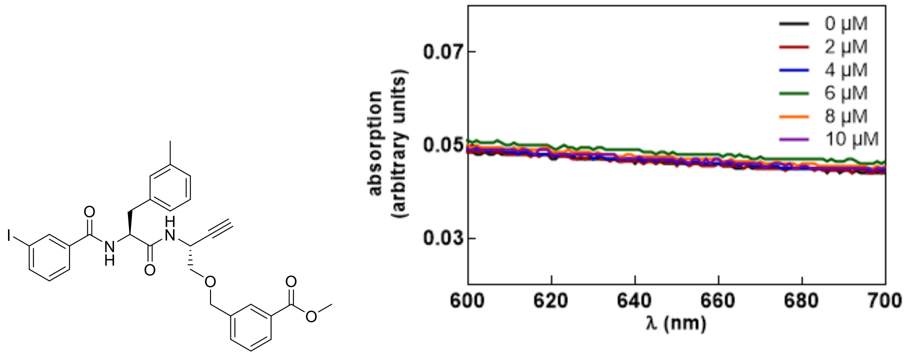

Figure S40: Solubility determination of compound **64**

Inhibition of cathepsin B by dipeptide nitrile 1a

Exemplarily for inhibition by dipeptide nitriles, substrate conversion curves and the secondary plot of the calculated rates against inhibitor concentration is shown for compound **1a** and cathepsin B in Figure S41.

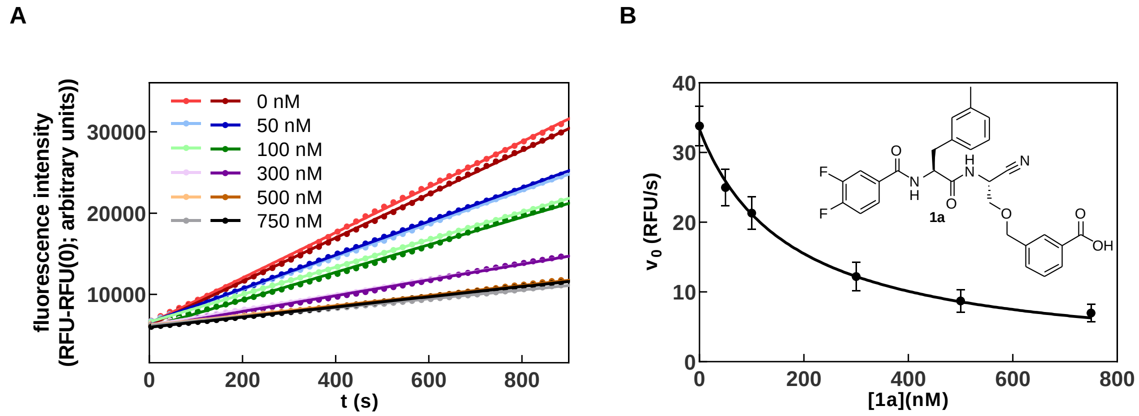

Figure S41: Substrate turnover curves of an example measurement as a double determination (**A**) and plot of the initial velocities v_i averaged over 4 measurements as v_i = f([**1a**]) (**B**) for the inhibition of cathepsin B by compound **1a**.

The linearity of substrate conversion during the observed time range is in agreement with fast reversible inhibition. The analysis of v_i = f([**1a**]) was performed according to equation II in the main text. The measurement was performed in four independent experiments (each as a duplicate determination) in assay buffer pH 6.0 containing 200 µM Z-RR-AMC, 25 ng/mL CatB, and 1.5% DMSO. Shown are the measured values ± SEM.

Determination of inhibition type for dipeptide nitrile 1a at cathepsin B

The K_i values of the dipeptide nitriles were determined by substituting the determined IC_{50} values into Equation III. This form of the Cheng-Prusoff equation applies to competitive inhibitors.[3] Since the dipeptide nitriles react covalently with the cysteine residue in the active site, a competitive inhibition mechanism was expected. To test this assumption, an inhibition type determination was performed for compound **1a**.

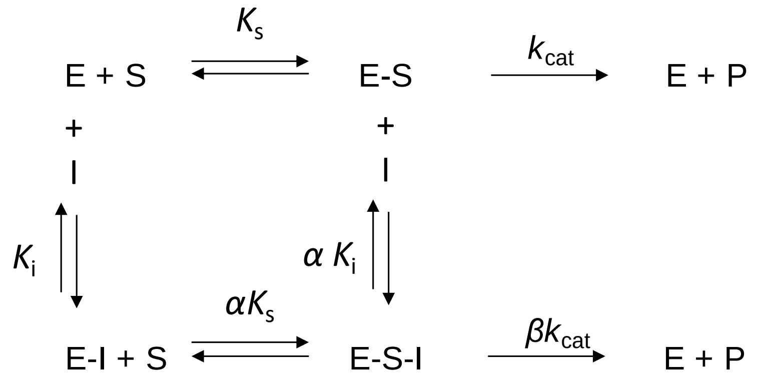

Figure S42: Kinetic model for substrate turnover in the presence of a reversible inhibitor

Figure S42 shows the possible interactions between an enzyme and an inhibitor in the presence of a substrate. Here, K_s and K_i are the dissociation constants of the enzyme-substrate/inhibitor complex and k_{cat} is the first-order rate constant of product release from the enzyme-substrate complex. The factor α defines the influence of the inhibitor on the binding of the substrate to the enzyme (and vice versa). The interference of the inhibitor with product formation is defined by β.

In the simplest case, the binding of the inhibitor prevents the binding of the substrate at the active site and α = 0(EI×S >> ESI) and β = 0. In this case, we speak of competitive inhibition. Substrate and inhibitor compete for the free enzyme. Thus, this inhibition can be overcome by increasing the substrate concentration. Thus, K_i' increases with substrate concentration.[4] More often, however, the inhibitor binds to both free enzyme and the enzyme-substrate complex. Thus, α ≠ 0 and β = 0. This case is described as non-competitive inhibition. Thus, V_{max} decreases with inhibitor concentration; K_m remains constant. Often, only the special case with α = 1 is described as non-competitive inhibition. For α ≠ 0; 1, the terminology "mixed-type inhibition" is used.[4] Another form of inhibition is uncompetitive inhibition, in which the inhibitor has much higher affinity toward the enzyme-substrate complex than toward free enzyme (α << 1). The last special case is partial inhibition, where enzyme-substrate-inhibitor complex can still release product (0 < β < 1). This means that complete inhibition of substrate turnover does not occur despite high inhibitor concentrations. However, this phenomenon can also be observed due to solubility phenomena (incomplete inhibition at maximum possible inhibitor concentration), so misinterpretation of the data must be carefully excluded.

The inhibition type of a compound can be determined graphically, most conveniently by the double reciprocal transformation of LINEWEAVER-BURK (plot of 1/v versus 1/[S]).[5] If substrate conversion is measured at varying inhibitor concentrations and the resulting array of

curves is superimposed, a characteristic pattern of lines of the particular inhibitor type is obtained, which indicates the inhibition type.

Since a competitive inhibitor does not affect V_{max}, the common point of intersection for the set of lines lies on the y-axis (because the point of intersection with the y-axis corresponds to $1/V_{max}$ in the double reciprocal plot). The slope ($K_{m,app}/V_{max,app}$) varies because $K_{m,app}$ depends on inhibitor concentration. From the plot of curves, K_i can be determined by two methods: If one plots the determined $K_{m,app}$ against [I], the obtained straight line intersects the x-axis at -K_i. The second method is to plot 1/v against [I] for different [S] (Dixon plot).[6] The straight lines intersect at -K_i.

For non-competitive inhibition ($\alpha \neq 0$), V_{max} varies and so does the intersection with the y-axis. When plotting 1/v versus 1/[S], the curves intersect in the third quadrant ($\alpha < 1$) or in the 4th quadrant ($\alpha > 1$). For the special case with $\alpha = 1$ (strictly non-competitive inhibition), the intersection point is located on the x-axis. From the Dixon plot of $1/V_{max}$ versus [I], -αK_i can be determined from the intersection of the obtained straight lines with the x-axis. In addition, the slopes of the straight lines from the double-reciprocal plot can be plotted against [I]. Again, a straight line intersecting the x-axis at -K_i is obtained. From both secondary plots, α and K_i can thus be determined.

For uncompetitive inhibitors, the slope of the straight line in the double-reciprocal plot is independent of the inhibitor concentration. Thus, parallel-shifted straight lines are obtained, with the intercept with the y-axis increasing with increasing inhibitor concentration. If [S] >> K_m holds (substrate saturation), - αK_i can be determined from the Dixon plot as described for non-competitive inhibition.

For the determination of the inhibition type of **1a**, six measurements were performed at different substrate concentrations analogous to the determination of the kinetic constants of the inhibitors:

A 1 mM stock solution of **1a** in DMSO was first diluted 1:10 with assay buffer containing 10% DMSO to 100 µM and then to the desired intermediate dilutions. A 10 mM Z-RR-AMC stock solution in DMSO was first diluted 1:10 with assay buffer containing 10% DMSO to 1 mM and then to the desired intermediate dilutions. For the enzyme intermediate-dilution, the cathepsin B stock solution was first diluted to 54.44 µg/mL with cathepsin B enzyme buffer and then diluted to 0.5 µg/mL with assay buffer containing 10 mM DTT. The final enzyme concentration in assay was 25 ng/mL.

In a black 96-well plate with a flat, transparent bottom, 10 µL of inhibitor intermediate-dilution and 20 µL of substrate intermediate-dilution were added to 160 µL of assay buffer and incubated for 20 min at 37°C. The enzyme intermediate-dilution was pre-activated for 5 min at 37°C in a water bath and then the reaction was started by adding 10 µL of enzyme solution to the substrate inhibitor solution.

Substrate turnover was monitored by the increase in fluorescence in a BIOTEK Synergy 4 Hybrid Multi-Mode Microplate Reader (15 min, 37°C, Exc360/40, Em410/40, Sens45, bottom-read). All measurement points were recorded as duplicates. Analysis was performed in Prism (GraphPad Software, Inc., version 5.02). Initial velocities were determined by linear regression of the obtained substrate turnover curves. Since some of the control curves were slightly curved, only the first 300 seconds of the measurement were evaluated. Figure S43 shows the resulting v_i = f([S];[I]) and the double reciprocal plot according to LINEWEAVER and BURK.

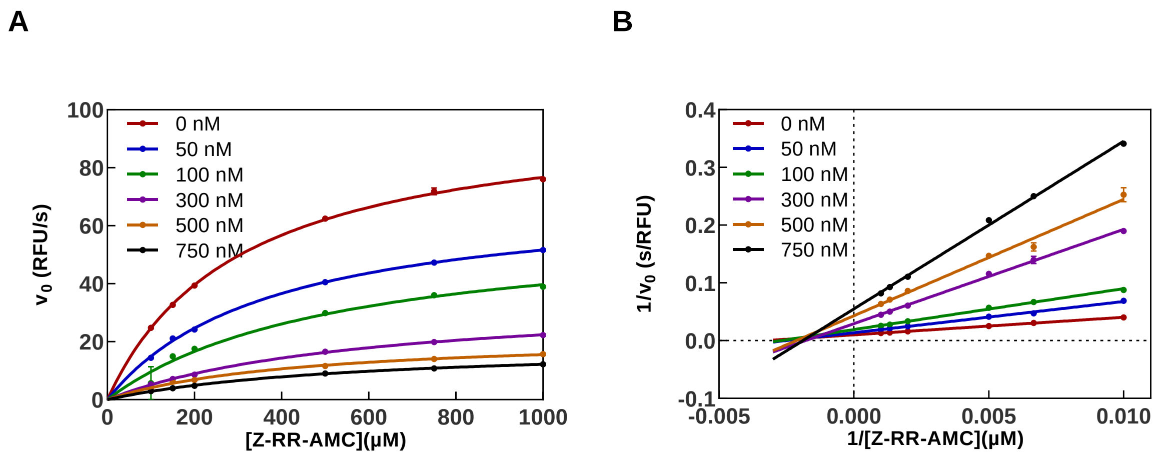

Figure S43: Determination of inhibition type for dipeptide nitrile **1a**. **A**) Initial rates of substrate turnover as a function of substrate concentration (x-axis) and inhibitor concentration (legend). **B**) Double-reciprocal plot of $1/v_i$ versus 1/[S] for different inhibitor concentrations. The set of lines intersects in the 4th quadrant, which allows for characterizing **1a** as a mixed-type, predominantly competitive inhibitor with α > 1. Measurements were performed as duplicate determinations in assay buffer pH 6.0 containing 200 µM Z RR AMC, 25 ng/mL CatB, and 1.5% DMSO. Mean values ± SEM are shown. Figure is identical to Figure 7

To avoid overweighting of the velocities measured at small substrate concentrations, COPELAND recommends evaluating v_0 = f([S]) according to the Michaelis-Menten equation. The obtained constants $K_{m,app}$ and $V_{max,app}$ in the presence of the inhibitor can then be substituted directly into the reciprocal equation VIII.[4]

$$\frac{1}{v} = \left(\frac{K_m}{V_{max}}\frac{1}{[S]}\right) + \frac{1}{V_{max}} \qquad \text{I}$$

Both the direct transformation of the values and the indirect procedure resulted in an intersection of the straight line chart in the 4th quadrant. Thus, **1a** is a non-competitive inhibitor according to COPELAND with α > 0. SEGEL [7] additionally distinguishes between pure non-competitive inhibition (α = 1) and mixed-type inhibition (α ≠ 0; 1), with **1a** belonging to the latter.[7] Here, α > 0 means that the affinity of the inhibitor towards the free enzyme is greater than towards the enzyme-substrate complex.

To determine the kinetic constants α and Ki, the different V_{max} were plotted in the Dixon plot as $1/V_{max}$ = f([I]). From the double-reciprocal plot, the slopes of the straight lines were determined and plotted as slope = f([I]). For partial inhibition, slope = f([I]) would be hyperbolic, and for β = 0 it would be a straight line. The intersection points with the x-axis correspond to $-\alpha K_i$ and $-K_i$, respectively (

A

B

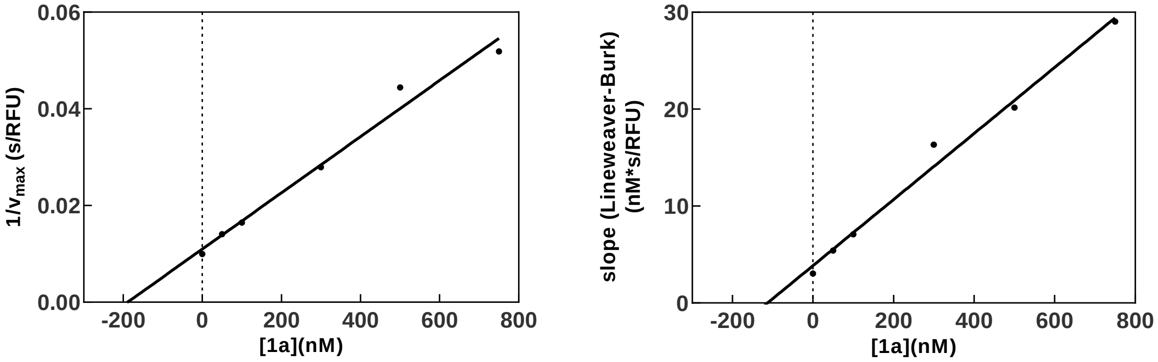

Figure S44).

A **B**

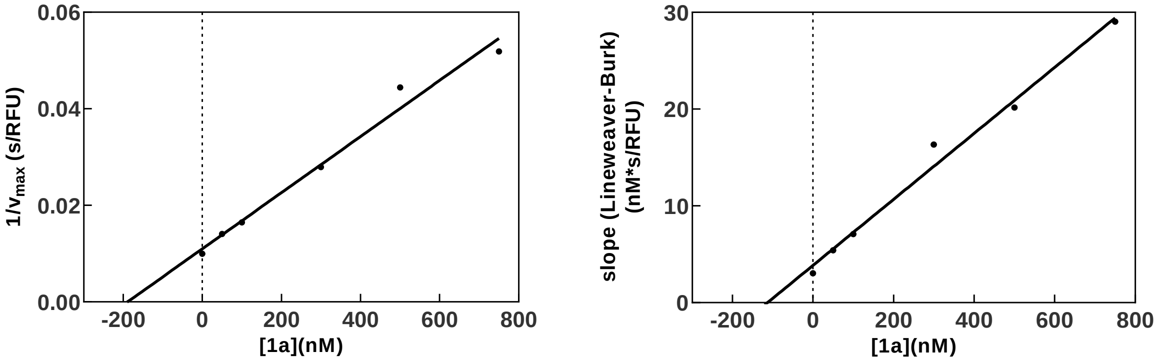

Figure S44: Secondary plots $1/V_{max}$ = f([I]) (**A**) and line slopes as obtained in the Lineweaver-Burk plot the double reciprocal plot = f([I]) (**B**). The intersection of $1/V_{max}$ = f([I]) with the x-axis corresponds to $-\alpha K_i$ and the plot of the slope against [I] corresponds to K_i. The linear progression of this plot also confirms complete inhibition ($\beta_{\mathbf{1a}}$ = 0).

From the secondary plots shown, $-\alpha K_i$ = -189.2 and $-K_i$ = -113.1, yielding the following kinetic constants for **1a**:

Noncompetitive/mixed inhibition, K_i = 113.1 nM, α = 1.67, β = 0.

A more accurate method is the global evaluation of v_0 = f([S]; [I]) according to Equation IX.

$$v_0 = \frac{V_{max}[S]}{[S]\left(1+\frac{[I]}{\alpha K_i}\right)+K_m\left(1+\frac{[I]}{K_i}\right)} \qquad \text{III}$$

Equation IX yields K_i = 63.8 nM and α = 2.48.

Therefore, dipeptide nitrile **1a** acts predominantly as a competitive inhibitor towards cathepsin B, even though its inhibition is accompanied by a weaker non-competitive component.

Weak and reversible inhibition of cathepsin B by dipeptide alkyne 28

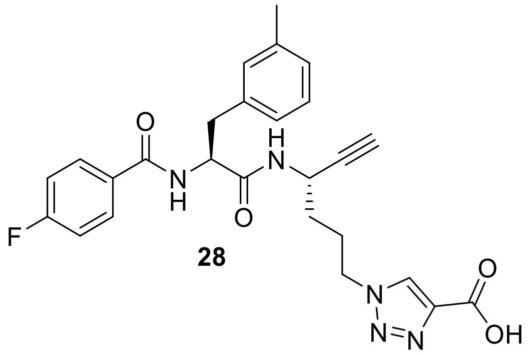

28

A

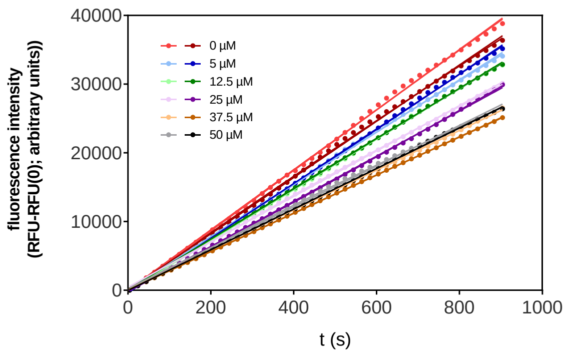

B

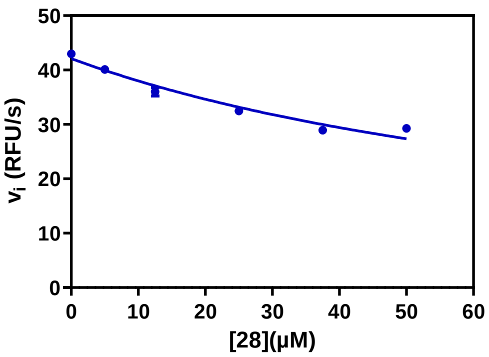

Figure S45: A) Turnover of Z-RR-AMC by cathepsin B in the presence of increasing concentrations of dipeptide alkyne **28. B)** Plot of initial velocities as v_i=f[**28**]

Proof of irreversible inhibition by jump dilution

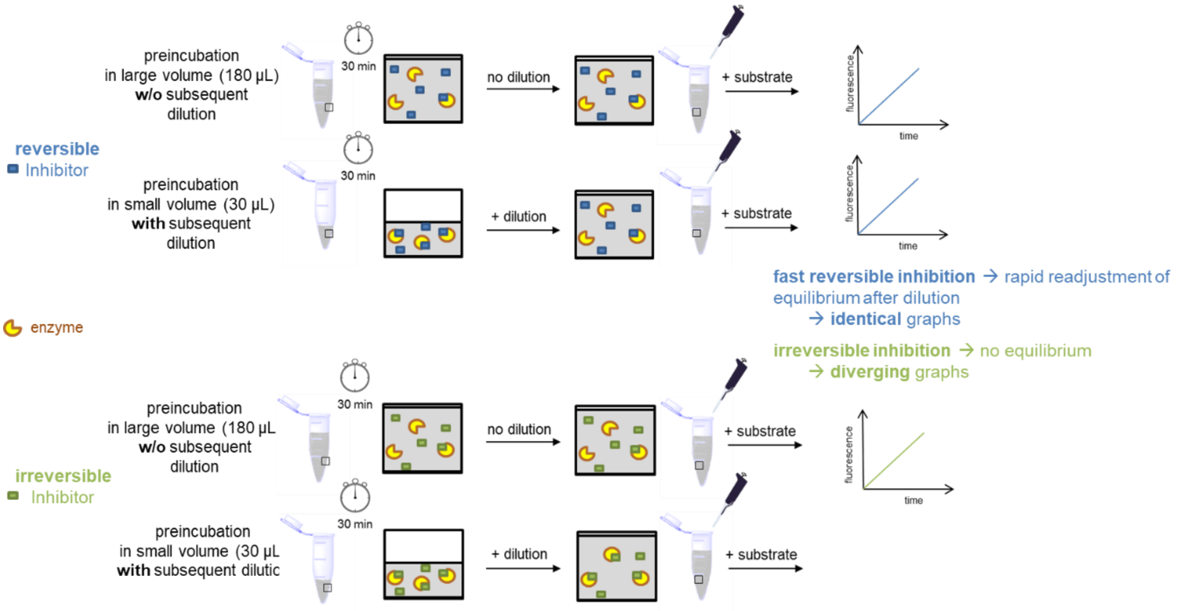

Figure S46: Schematic representation of the jump-dilution experiment for distinguishing between fast reversible, slow reversible (this case is not considered in the Scheme) and irreversible inhibition.

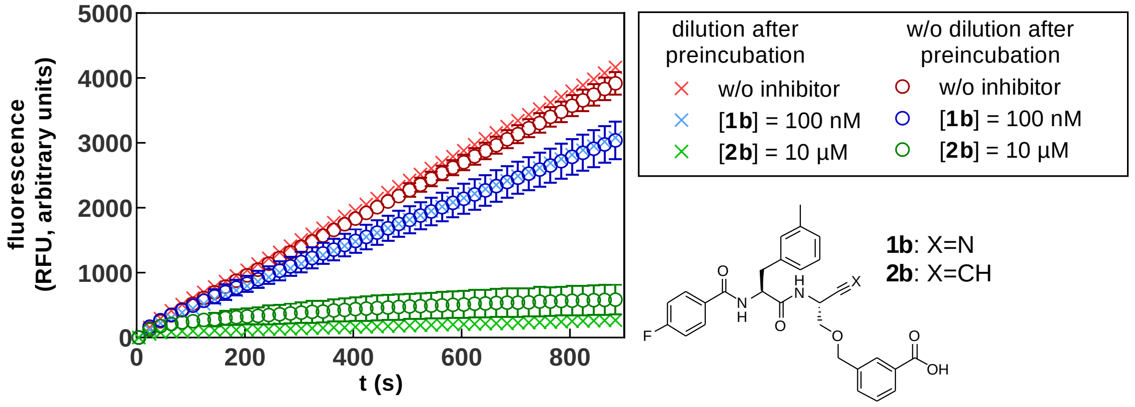

Figure S47: Jump-dilution inhibition experiment for nitrile **1b** and alkyne **2b** with cathepsin S to prove the irreversible inhibition by **2b**. The inhibitors were pre-incubated in a volume of 30 µL or 180 µL in the presence of enzyme as schematically shown in Figure S46. The highly concentrated solution was diluted to 180 µL immediately before measurement. Subsequently, the reaction was started by substrate addition so that equal enzyme and inhibitor concentrations were achieved at the start of each measurement. The measurement was performed as a duplicate determination in assay buffer pH 6.0 containing 200 µM Z-RR-AMC, 2,5 ng/mL CatS and 1.5% DMSO. The concentrations indicated refer to the final concentration during the measurement after dilution.

Comparison of selectivity profiles of dipeptide nitriles and the corresponding alkynes

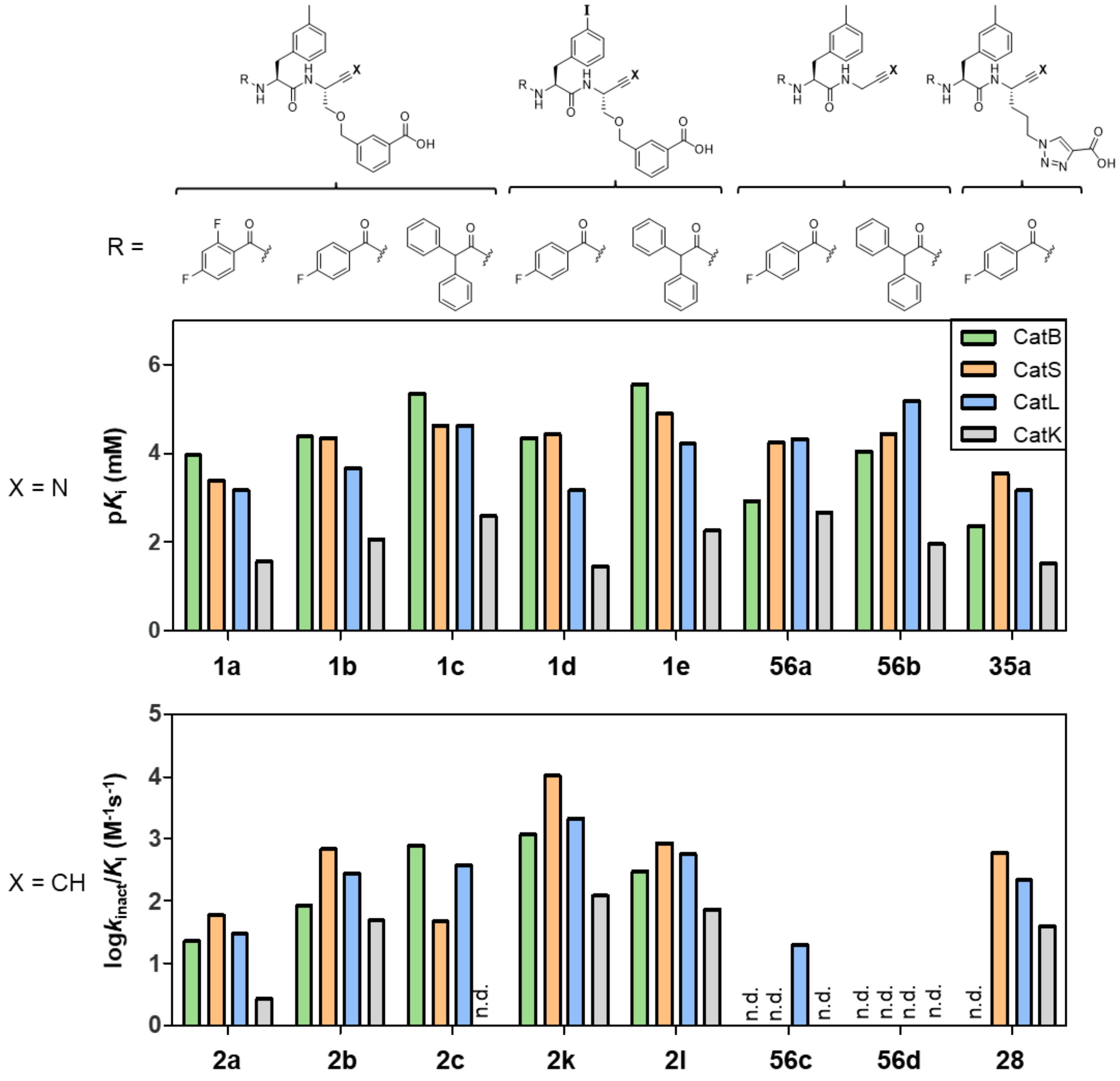

Figure S48: Selectivity profile of stereochemically pure alkyne-based inhibitors (bottom) and corresponding nitriles (top). Shown are the negative decadic logarithm of the K_i values and the decadic logarithm of the inactivation constants k_{inact}/K_I. Thus, larger values are equivalent to a higher inhibition potential of the compounds. The measurement was performed in 3 independent experiments (each as a duplicate determination) in assay buffer pH 6.0 with 1.5% DMSO. n.d. = not determinable (No inhibition constant was determinable/no inhibition was observed in the investigated concentration range).

n.d. – no inhibition

Influence of varying *meta* substituents at *N*-benzoyl dipeptide alkynes on inhibition of cathepsins B, S, L and K

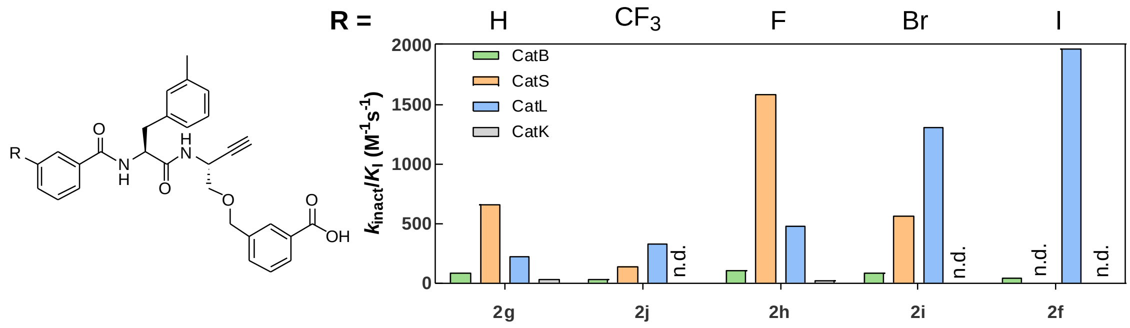

Figure S49: Influence of the substituent in *meta* position of the benzoyl residue in P3 on the inhibition of cathepsins B, L, S and K. Data points for cathepsin L are identical to those in Figure 13A in the main text.

n.d. - no inhibition

Molecular models of cathepsin (B/S/L)-inhibitor complexes for selected compounds obtained by covalent docking *in silico*

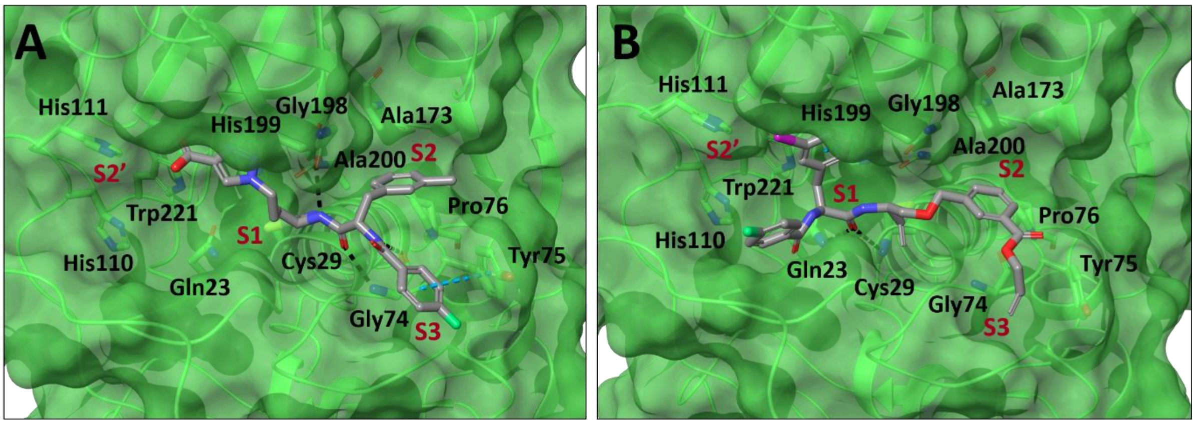

Figure S50: *In silico* predicted models for the interaction of cathepsin B with covalent inhibitors. Cathepsin B is shown in green cartoon and transparent surface representation. Interacting protein residues are shown in sticks, colored by atom type and labelled. Inhibitors A) **35a** and B) **53k** are shown in grey sticks and colored by atom type. Pocket binding sites S1-S3 are indicated by red labels. Intermolecular H-bonds and π-π interactions are depicted by black and cyan dashed lines, respectively. A) **35a** and B) **53k**. Figure generated in Maestro (Schrödinger).

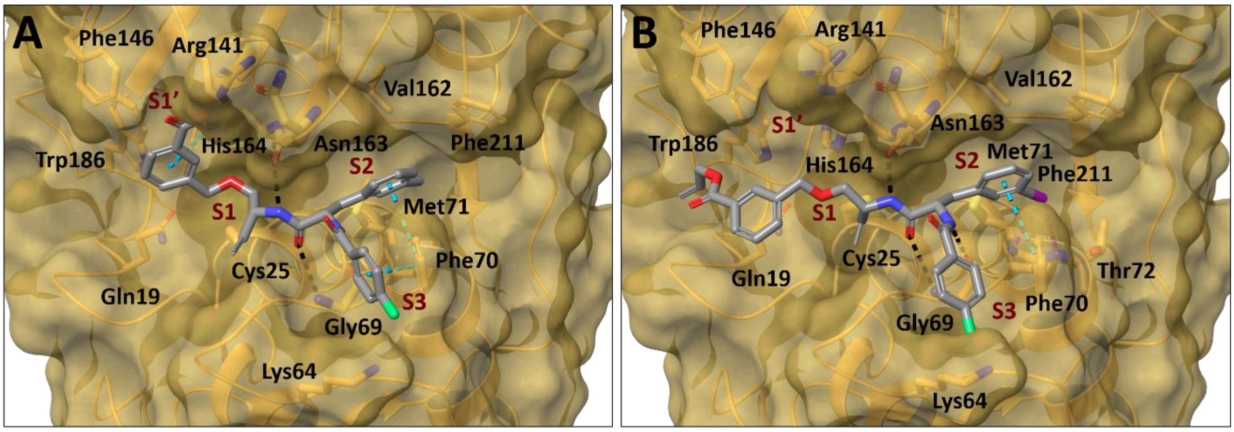

Figure S51: *In silico* predicted models for the interaction of cathepsin S with covalent inhibitors. Cathepsin S is shown in orange cartoon and transparent surface representation. Interacting protein residues are shown in sticks, colored by atom type and labelled. Inhibitors A) **2b** (second best docking score) and B) **53k** are shown in grey sticks and colored by atom type. Intermolecular H-bonds, π-π interactions and halogen H-bond interactions are depicted by black, cyan and purple dashed lines, respectively. Pocket binding sites S1-S3 are indicated by red labels. Figure generated in Maestro (Schrödinger).

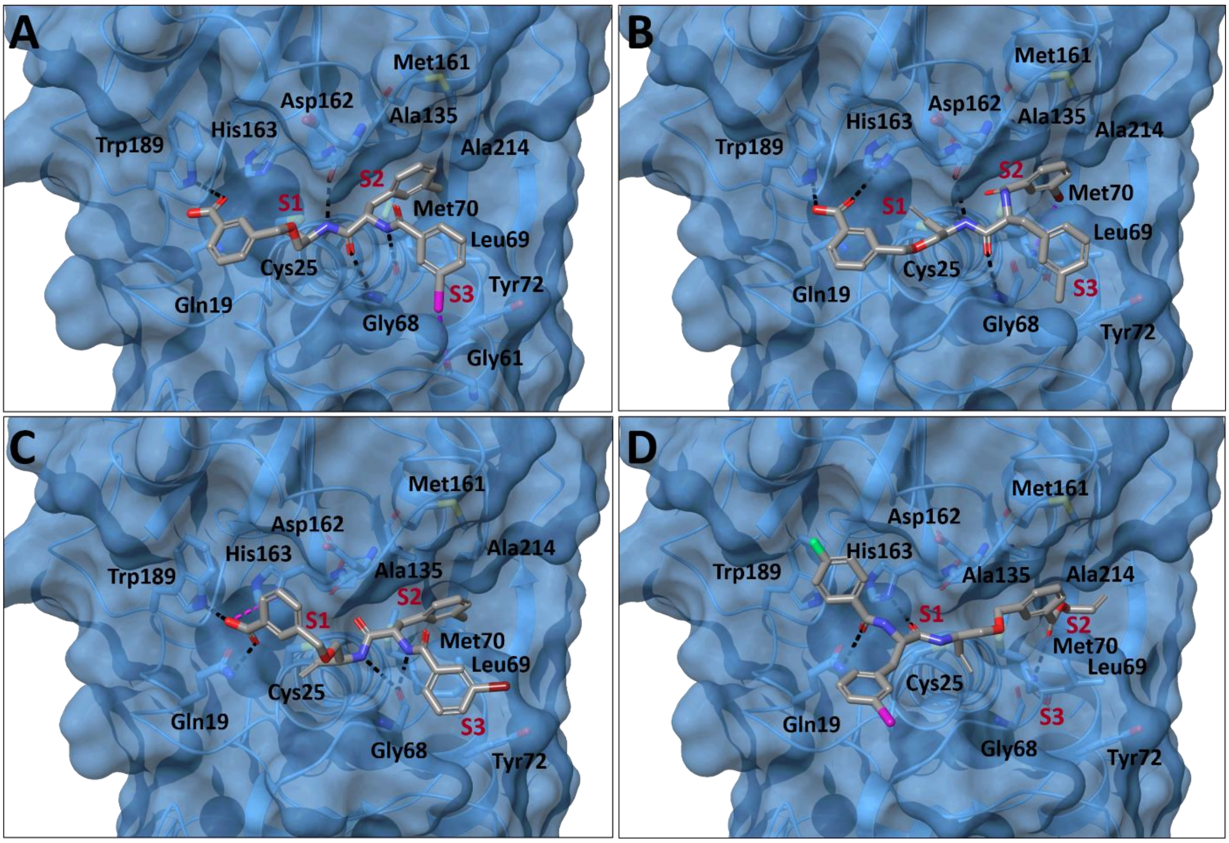

Figure S52: *In silico* predicted models for the interaction of cathepsin L with covalent inhibitors. Cathepsin L is shown in cyan cartoon and transparent surface representation. Interacting protein residues are shown in sticks, colored by atom type and labelled. Inhibitors A) **2f** B) **2i** (best Prime energy), C) **2i** (best docking score) and D) **53k** are shown in grey sticks and colored by atom type. Intermolecular H-bonds, salt bridges and halogen H-bond interactions are depicted by black, magenta and purple dashed lines, respectively. Figure generated in Maestro (Schrödinger).

Overview of determined kinetic parameters for inhibitor compounds

Table S1: Kinetic parameters for dipeptide nitriles

compound				cathepsin B		cathepsin S		cathepsin L		cathepsin K	
#	P3	P2	P1	IC_{50} (µM)	K_i (µM)	IC_{50} (µM)	K_i (µM)	IC_{50} (µM)	K_i (µM)	IC_{50} (µM)	K_i (µM)
**1a**	2,4-DiFBz	(3-Me)Phe	(BnCOOH)Ser	0.181	0.109	1.254	0.406	2.826	0.661	135.4	25.94
**1b**	4-FBz	(3-Me)Phe	(BnCOOH)Ser	0.071	0.042	0.141	0.046	3.882	0.220	45.96	8.807
**1c**	$(Ph)_2$CHCO	(3-Me)Phe	(BnCOOH)Ser	0.007	0.004	0.075	0.024	0.102	0.024	7.778	2.503
**1d**	4-FBz	(3-I)Phe	(BnCOOH)Ser	0.072	0.043	0.115	0.037	2.977	0.696	108.6	34.92
**1e**	$(Ph)_2$CHCO	(3-I)Phe	(BnCOOH)Ser	0.004	0.003	0.038	0.012	0.254	0.059	17.56	5.646
**35a**	4-FBz	(3-Me)Phe	Orn(TriazoleCOOH)	6.845	4.118	0.862	0.270	2.969	0.694	94.24	30.41
**35b**	4-FBz	(3-Me)Phe	Orn(TriazoleCH_2COOH)	7.614	4.580	0.986	0.319	2.296	0.537	78.40	25.21
**35c**	4-FBz	(3-Me)Phe	Lys(TriazoleCOOH)	3.889	2.343	0.663	0.215	2.026	0.474	80.81	25.99
**42**	4-FBz	(3-Me)Phe	Ser(CH_2TriazoleCOOMe)	1.428	0.859	0.341	0.110	0.209	0.049	12.58	4.043
**43**	4-FBz	(3-Me)Phe	Ser(CH_2TriazoleCOOH)	2.680	1.612	0.588	0.191	2.236	0.523	99.79	19.11
**56a**	4-FBz	(3-Me)Phe	Gly	1.979	1.187	0.171	0.055	0.209	0.049	6.595	2.121
**56b**	$(Ph)_2$CHCO	(3-Me)Phe	Gly	0.144	0.086	0.116	0.038	0.028	0.007	34.26	11.02

Table S2: Kinetic parameters for dipeptide alkynes

To be continued at the next page

compound				cathepsin B		cathepsin S		cathepsin L		cathepsin K	
**#**	**P3**	**P2**	**P1**	**k_{inact}/K_I ($M^{-1}s^{-1}$)**	**K_i (μM)[a]**	**k_{inact}/K_I ($M^{-1}s^{-1}$)**	**K_i (μM)[a]**	**k_{inact}/K_I ($M^{-1}s^{-1}$)**	**K_i (μM)[a]**	**k_{inact}/K_I ($M^{-1}s^{-1}$)**	**K_i (μM)[a]**
**2a**	2,4-DiFBz	(3-Me)Phe	(BnCOOH)Ser	22	335.6	58	—[b]	30	—	3	—
**2b**	4-FBz	(3-Me)Phe	(BnCOOH)Ser	85	—	682	12.86	281	25.31	48	247.9
**2c**	(Ph)₂CHCO	(3-Me)Phe	(BnCOOH)Ser	771	—	47	23.06	381	—	n.i.[c]	n.i.
**2d**	PhPhCO	(3-Me)Phe	(BnCOOH)Ser	41	—	n.i.	8.21	n.i.	n.i.	n.i.	n.i.
**2e**	4-IBz	(3-Me)Phe	(BnCOOH)Ser	152	—	113	17.75	82	—	476	12.50
**2f**	3-IBz	(3-Me)Phe	(BnCOOH)Ser	45	—	n.i.	5.64	1968	8.69	n.i.	n.i.
**2g**	Bz	(3-Me)Phe	(BnCOOH)Ser	88	95.67	654	7.90	222	25.06	32	n.i.
**2h**	3-FBz	(3-Me)Phe	(BnCOOH)Ser	109	82.79	1579	7.66	483	—	27	146.8
**2i**	3-BrBz	(3-Me)Phe	(BnCOOH)Ser	87	19.47	570	3.42	1309	9.17	n.i.	n.i.
**2j**	3-(F_3C)Bz	(3-Me)Phe	(BnCOOH)Ser	29	216.8	141	12.22	327	31.88	n.i.	n.i.
**2k**	4-FBz	(3-I)Phe	(BnCOOH)Ser	1179	5.92	10133	1.77	2128	7.00	121	—.
**2l**	(Ph)₂CHCO	(3-I)Phe	(BnCOOH)Ser	301	25.26	859	3.75	552	12.08	76	—
**2m**	3-IBz	(3-I)Phe	(BnCOOH)Ser	225	7.31	4368	1.02	2876	—	n.i.	n.i.
**18**	2,4-DiFBz	(3-Me)Phe	(BnCOOH)Ser	34	154.3	---[d]	---	---	---	---	---

	compound			cathepsin B		cathepsin S		cathepsin L		cathepsin K	
#	P3	P2	P1	k_{inact}/K_I ($M^{-1}s^{-1}$)	K_i (µM)[a]	k_{inact}/K_I ($M^{-1}s^{-1}$)	K_i (µM)[a]	k_{inact}/K_I ($M^{-1}s^{-1}$)	K_i (µM)[a]	k_{inact}/K_I ($M^{-1}s^{-1}$)	K_i (µM)[a]
**28**	4-FBz	(3-Me)Phe	Orn(TriazoleCOOH)	n.i.	55.95	594	7.88	214	28.92	40	—
**53k**	4-FBz	(3-I)Phe	(BnCOOAllyl)Ser	141	—	3589	—	1246	—	n.i.	n.i.
**56c**	4-FBz	(3-Me)Phe	Gly	n.i.	n.i.	n.i.	21.39	19	10.93	n.i.	n.i.
**56d**	$(Ph)_2CHCO$	(3-Me)Phe	Gly	n.i.	n.i.	n.i.	8.92	n.i.	n.i.	n.i.	n.i.
**56e**	4-IBz	Leu	Gly	4	73.56	79	11.68	93	—	260	6.22
**56f**	4-FBz	Cha	Gly	34	73.15	293	7.50	n.i.	3.70	179	73.42
**62**	4-FBz	$iBuSO_2Ala$	Gly	n.i.	n.i.	14	12.57	n.i.	n.i.	n.i.	n.i.
**63**	4-FBz	(3-Me)Phe	(BnCOOMe)Ser	n.i.	n.i.	12	39.65	79	—	n.i.	n.i.
**64**	3-IBz	(3-Me)Phe	(BnCOOMe)Ser	15	—	137	6.75	537	13.87	n.i.	n.i.

[a]refers to the dissociation constant of the eventually formed initial non-covalent complex
[b]— - for the case that no K_i value was stated in the presence of a k_{inact}/K_I value, no systematic decrease of the initial velocity with increasing inhibitor concentration was discernable
[c]n.i. - no (irreversible) inhibition was observed in the tested concentration range
[d]not determined

Table S3: IC_{50} values determined for selected dipeptide-derived alkynes for preincubation with cathepsin B for 30 min prior to substrate addition*

	compound			Cat B
#	P_3	P_2	P_1	$IC_{50,30\ min}$ (μM)
**2a**	2,4-DiFBz	(3-Me)Phe	(BnCOOH)Ser	16.9
**2b**	4-FBz	(3-Me)Phe	(BnCOOH)Ser	5.93
**2c**	$(Ph)_2CHCO$	(3-Me)Phe	(BnCOOH)Ser	0.52
**2d**	PhPhCO	(3-Me)Phe	(BnCOOH)Ser	7.5
**2e**	4-IBz	(3-Me)Phe	(BnCOOH)Ser	2.1
**2f**	3-IBz	(3-Me)Phe	(BnCOOH)Ser	7.1
**2g**	Bz	(3-Me)Phe	(BnCOOH)Ser	159
**2h**	3-FBz	(3-Me)Phe	(BnCOOH)Ser	138
**2i**	3-BrBz	(3-Me)Phe	(BnCOOH)Ser	32.4
**2j**	3-(F_3C)Bz	(3-Me)Phe	(BnCOOH)Ser	360
**2k**	4-FBz	(3-I)Phe	(BnCOOH)Ser	9.8
**2l**	$(Ph)_2CHCO$	(3-I)Phe	(BnCOOH)Ser	42.0
**2m**	3-IBz	(3-I)Phe	(BnCOOH)Ser	12.1
**18**	2,4-DiFBz	(3-Me)Phe	(BnCOOH)Ser	
**28**	4-FBz	(3-Me)Phe	Orn(TriazoleCOOH)	93
**53k**	4-FBz	(3-I)Phe	(BnCOOAllyl)Ser	30.6
**56c**	4-FBz	(3-Me)Phe	Gly	
**56d**	$(Ph)_2CHCO$	(3-Me)Phe	Gly	
**56e**	4-IBz	Leu	Gly	122
**56f**	4-FBz	Cha	Gly	122
**62**	4-FBz	$iBuSO_2Ala$	Gly	
**63**	4-FBz	(3-Me)Phe	(BnCOOMe)Ser	131
**64**	3-IBz	(3-Me)Phe	(BnCOOMe)Ser	141

*In analogy to the set-up for the control experiment, inhibitor and enzyme were preincubated for 30 min in 180 µL prior to addition of 20 µL of substrate solution

CHI IAM values of inhibitor compounds

Table S4: CHI IAM coefficients of dipeptide nitriles and alkynes

dipeptide nitrile	CHI-IAM	dipeptide alkyne	CHI-IAM
**1a**	28.6	**2a**	29.0
**1b**	27.7	**2b**	28.2
**1c**	34.5	**2c**	34.4
**1d**	32.6	**2d**	36.8
**1e**	38.3	**2e**	34.4
**35a**	23.2	**2f**	34,3
**35b**	22.8	**2g**	27.4
**35c**	24.6	**2h**	28.8
**42**	32.8	**2i**	32.8
**43**	23.0	**2j**	32.7
**56a**	33.1	**2k**	31.9
**56b**	39.5	**2l**	38.0
		**2m**	38.3
		**28**	24.6
		**56c**	34.3
		**56d**	40.6
		**56e**	37.4
		**56f**	37.4
		**62**	25.0
		**63**	41.8
		**64**	46.2

The determined coefficients are in good correlation with the structures of the compounds. Thus, as expected, the introduction of diphenylacetyl in P3 leads to significantly higher values than for fluorobenzoyl in P3 (for example, **1c**, **1e**, **2c**, and **2l** *versus* **1b**, **1d**, **2b**, and **2k**). Also, the introduction of iodine in place of methyl in both P2 (**1d** and **2k** *versus* **1b** and **2b**) and P3 (for example, **2e** and **2f** *versus* **2b**) leads to enhanced membrane interaction. The influence of the free carboxyl group is also readily apparent. The absence of the side chain in P1 (**56a** *versus* **1b** or **56c** compared with **2b**) or the esterification of the carboxyl group to the methyl ester (for example, **63** *versus* **2b**) leads to higher CHI IAM coefficients, as expected. Interestingly, no significant difference is apparent between the corresponding dipeptide nitriles and alkynes.

Auberson *et al*. determined the CHI IAM coefficients of various PET tracer candidates clinically successful for brain imaging and obtained values between 13.1 and 51.7, median 34.50 and mean 34.49. On this basis, the authors recommend CHI IAM values to be approximately in the range between 30 and 50.[8] In the case of higher values, non-specific binding of the radiotracer may detrimentally influence its pharmacokinetics. Lower values indicate insufficient blood-brain barrier permeation by passive diffusion, which, however, should be less problematic for tumor-related target proteins.[9] Therefore, the CHI IAM values for the selective dipeptide alkynes **2f** and **2k**, with coefficients of 34.3 and 31.9, respectively, are in the range of values determined for successful compounds.

Methods for cell culture

U87-MG cells (origin: human glioblastoma) and A431 (origin: human epidermioid carcinoma) were originally purchased from ATCC and obtained as gift from the clinics of neurosurgery at Dresden University hospital. U251-MG cells (origin: human glioblastoma/astrocytoma) were purchased from ECACC via SIGMA-ALDRICH. Mel-Juso (origin: human melanoma) were purchased from DSZM. The cell lines were culture in DMEM containing 10% FCS and 1% HEPES. SW403 (origin: human colorectal adenocarcinoma) were purchased by DSZM and cultivated in DMEM containing 10% FCS and 1% P/S. SW480 (origin: human colorectal adenocarcinoma) were purchased by DSZM and cultivated in RPMI containing 10% FCS and 1% P/S. SW620 (origin: human colorectal adenocarcinoma) were purchased by ATCC and cultivated in DMEM containing 10% FCS and 1% P/S. All cells were incubated in an incubator at 37°C and 5% CO2 partial pressure. Cells were observed every 2 - 3 days and passaged at >95% confluence, but at least after 5 days.

Cells cultured in 75 cm^2 cell culture flasks were detached with 0.05% trypsin/0.02% EDTA and the enzymatic reaction was stopped with 5 mL cell culture medium. Cells were transferred to an Eppendorf tube, centrifuged (5 min, 4°C, 300×g) in and the cell pellet was washed three times with PBS. Storage was performed at -70°C.

For lysis, cell pellets were resuspended in 100 µL of lysis buffer (50 mM Tris-HCl [pH 8.0], 150 mM NaCl, 1% Nonidet P-40, 0.5% sodium desoxycholate, 0.1% SDS, 1 mM PMSF, 5 mM NaF, 1 mM Na3VO4, 1 mM DTT) and incubated for 10 min at room temperature. Cells were disrupted twice for 7 s with ultrasound (20%, pulsed) and cooled on ice for 5 min between lysis cycles. After centrifugation (15 min, 4°C, 16000 × g), the clear supernatant was transferred to a new Eppendorf tube and stored on ice or at - 70°C until further use.

Protein concentration was determined using the Pierce BCA Protein Assay Kit from THERMO FISHER SCIENTIFIC according to the method published by Smith *et al*.[10] A dilution series was prepared from a BSA standard solution (2 mg/mL) in the concentration range of 0 to 2 mg/mL. The lysates to be tested were diluted 20-fold with distilled water. In a clear 96-well plate, 25 µL of each protein solution was mixed with 200 µL of an intermediate dilution consisting of 50 parts by volume of Pierce Protein Assay Reagent A with one part by volume of Pierce Protein Assay Reagent B. The intermediate dilution was incubated at 37°C. After 30 min of incubation at 37°C, absorbance was measured at 562 nm on the Synergy 4 Hybrid Multi-Mode Microplate Reader from BIOTEK. The measurement was performed as a duplicate determination, using the pure intermediate dilution as a reference.

From the values obtained for the dilution series of the albumin standard, a calibration line was constructed to determine the unknown concentrations.

Western Blots with corresponding loading controls

To identify a suitable tumor model, different tumor cell lines were investigated with regards to their expression of the considered cysteine cathepsins. In the following, the Western blots with the corresponding loading controls are listed, unless they have already been shown in the

results section. Cells were cultured and harvested as described above. Western Blot procedure is describe in the Experimental Section in the main text.

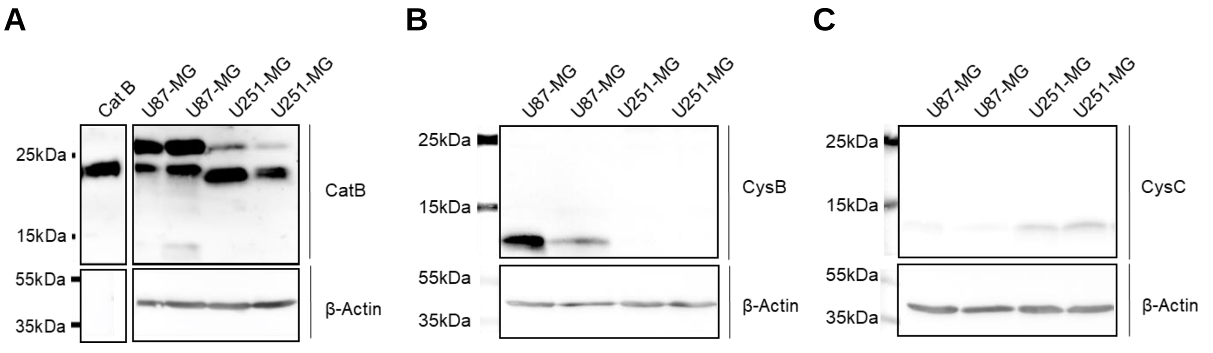

Figure S53: Cathepsin B (human, A) cystatin B (B) and cystatin C (C) protein levels in U87-MG and U251-MG tumor cell lysate and corresponding β-actin loading controls (complement to Figure 17 in the main text).

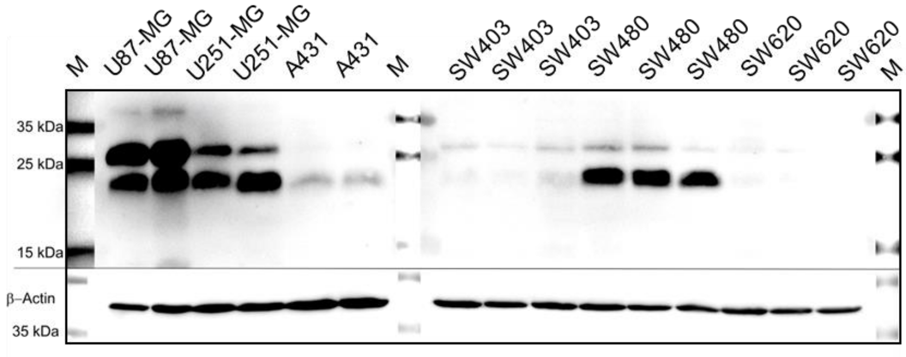

Figure S54: Cathepsin B synthesis in different tumor cell lysates and corresponding β-actin loading control.

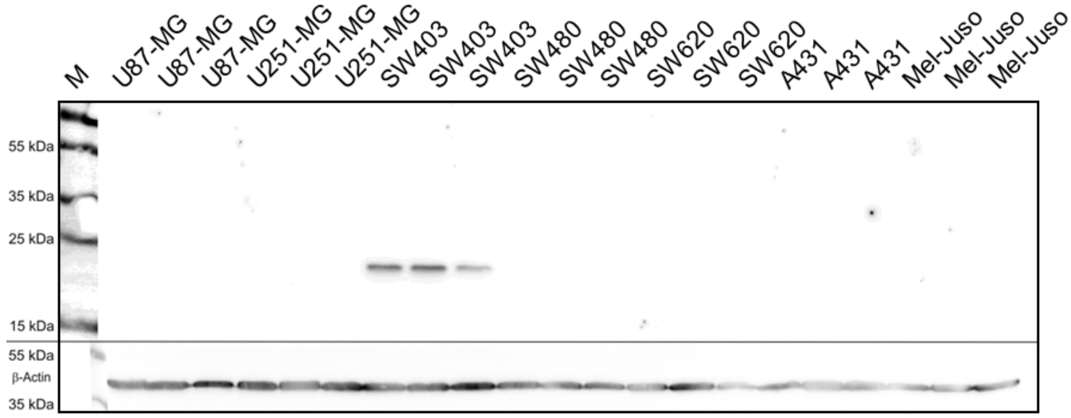

Figure S55: Cathepsin S synthesis in different tumor cell lysates and corresponding β-actin loading control.

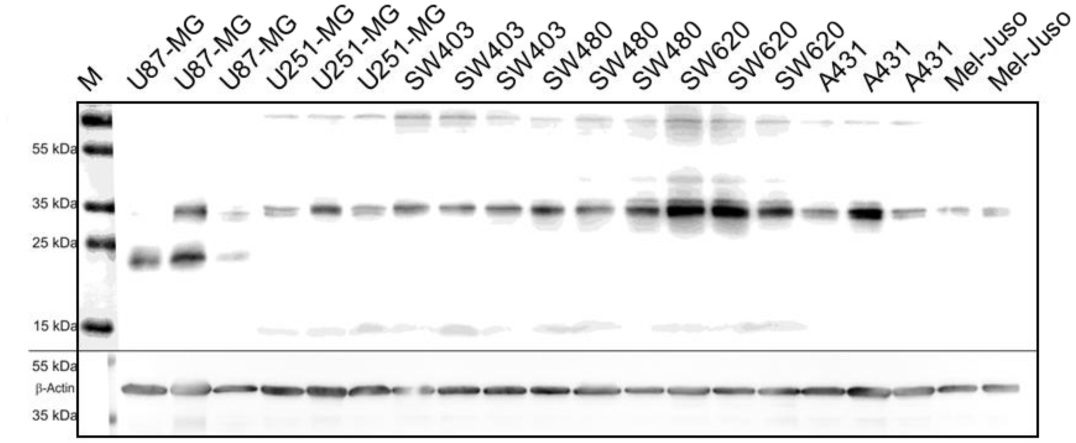

Figure S56: Cathepsin L synthesis in different tumor cell lysates and corresponding β-actin loading control.

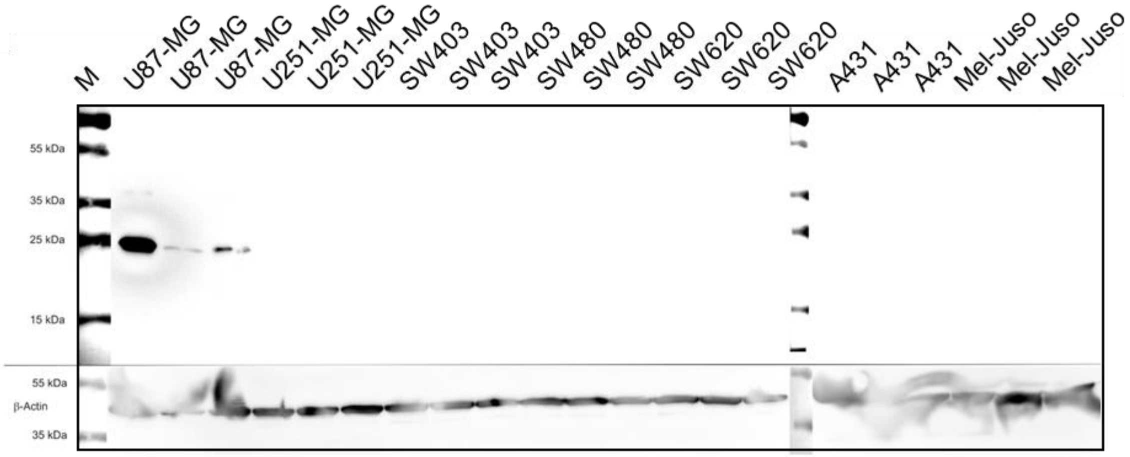

Figure S57: Cathepsin K synthesis in different tumor cell lysates and corresponding β-actin loading control.

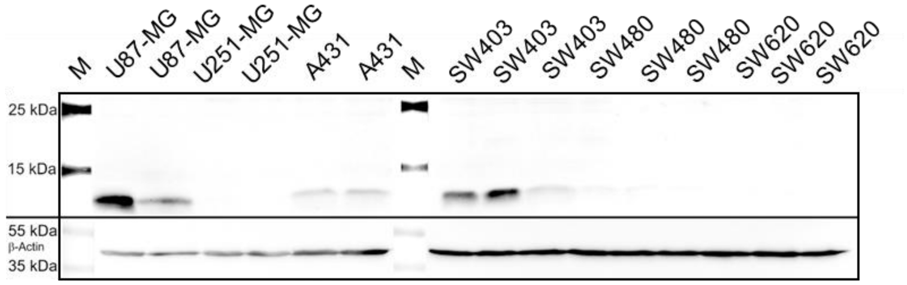

Figure S58: Cystatin B synthesis in different tumor cell lysates and corresponding β-actin loading control.

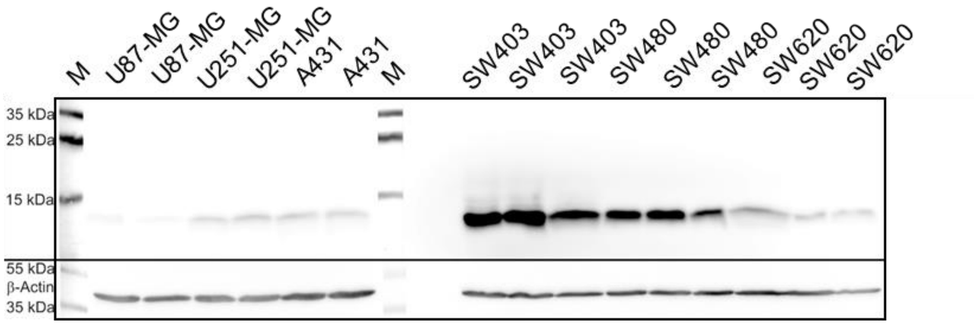

Figure S59: Cystatin C synthesis in different tumor cell lysates and corresponding β-actin loading control.

Investigation of intra- and extracellular Cathepsin B activity in different tumor cell lines

To implement a suitable experimental protocol for the investigation of inhibitor activity on living cells, different tumor cells were investigated with regards to their intra- and extracellular CatB activity using the hexapeptide substrate Abz-GIVRAK(Dnp)-NH2 and the CatB-specific inhibitor CA 074 as described below. The observed substrate conversion curves are shown in Figure S60. The Cat B protein levels as detected by Western Blot analysis are shown in Figure S54 for comparison.

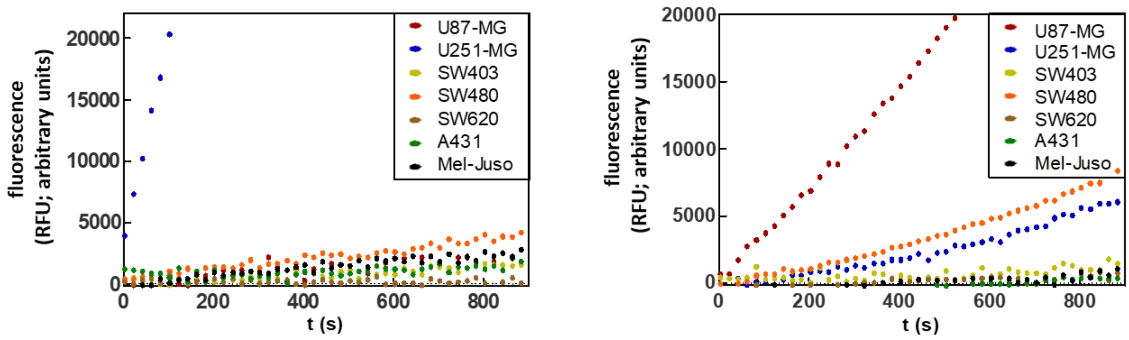

Figure S60: Cathepsin B-specific substrate turnover of Abz-GIVRAK(Dnp)-NH_2 in cell lysate (left) and on living cells (right).
Shown are the mean specific substrate turnover curves. To achieve these, for each individual measurement, an additional control with 10 μM CA-074 was performed to determine the nonspecific substrate turnover and then subtracted from the total substrate turnover. Measurements were performed in 3 independent experiments (each as a duplicate determination) in assay buffer pH 6.0 containing 0.5 mg protein/mL, 100 μM Abz-GIVRAK(Dnp)-NH_2, and 1% DMSO

Cell lysates

For cathepsin activity determination in lysed cells, lysates were first prepared as described above and then diluted with lysis buffer to a concentration of 10 mg/mL. A 10 mM stock solution

of the substrate Abz-GIVRAK(Dnp)-NH_2 in DMSO was diluted to 1 mM with 10% DMSO in assay buffer.

In a black 96 well plate, 150 µL of assay buffer, 10 µL of DTT in assay buffer (10 mM), 10 µL of lysate and 10 µL of CA-074 (0.2 mM in assay buffer) or 10 µL of assay buffer were incubated at 37°C for 30 min. The reaction was started by adding 20 µL of substrate intermediate-dilution. The final protein concentration was adjusted to 0.5 mg/mL and the substrate concentration was 100 µM.

Substrate turnover was monitored by detecting the increase in fluorescence in BIOTEK's Synergy 4 Hybrid Multi-Mode Microplate Reader (15 min, 37°C, Exc325, Em410, Sens120, top-read). All measurement points were recorded in duplicate for the cell lysates; each measurement was performed in triplicate.

For characterization of inhibitors in the cell lysate, 150 µL of assay buffer, 10 µL of DTT in assay buffer (10 mM), 10 µL of lysate and 10 µL of CA-074 solution (0.2 mM in assay buffer) or 10 µL of inhibitor stock solution in assay buffer containing 10% DMSO were incubated for 30 min at 37°C in a black 96-well plate. Subsequently, the measurement was carried out as described above.

Viable cells

For the determination of cathepsin B activity on viable cells, a suitable number of cells (determined with the CASY cell counter from INNOVATIS, Table S5) was placed in a 96-well plate and cultivated for 24 h in the usual culture medium in the incubator. Subsequently, the cells were carefully washed twice with 200 µL PBS directly in advance of the measurement.

Table S5: Cell counts suitable for performing the fluorimetric assay in a 96-well plate the following day.

The concentrations of the cell suspensions are given in cells/mL. 200 µL/well are introduced in each case.

	**U87-MG**	**U251-MG**	**SW403**	**SW480**	**SW620**	**A431**	**Mel-Juso**
**cells/mL**	3×10^5	2×10^5	5×10^5	7×10^5	3×10^5	3×10^5	2×10^5

A 10 mM stock solution of the substrate Abz-GIVRAK(Dnp)-NH_2 in DMSO was diluted with 10% DMSO in assay buffer first to 1 mM and then to the desired concentration of the intermediate dilution 100 - 200 - 400 - 600 - 800 - 1000 µM). The cells were incubated with 160 µL of assay buffer (pre-tempered to 37°C), 10 µL of DTT in assay buffer (10 mM) and 10 µL of CA-074 (0.2 mM in assay buffer) or 10 µL of assay buffer each for 30 min at 37°C in an incubator. The reaction was then started by adding 20 µL of substrate intermediate-dilution using a multichannel pipette with dispenser function.

Substrate turnover was monitored by the increase in fluorescence in BIOTEK's Synergy 4 Hybrid Multi-Mode Microplate Reader (15 min, 37°C, Exc325, Em410, Sens100, top-read). All

measurement points were recorded as duplicates; each measurement was performed in triplicate.

For the determination of the content of total protein of cells grown in the 96-well plate per well, 70 µL of lysis agent (1% SDS in 0.1 M NaOH) each was added to 4 wells and incubated for 30 min at room temperature on the shaker. Subsequently, the lysates obtained were processed as described in the Experimental Section in the main text. Protein determination was only possible in untreated wells, as cell adhesion in assay buffer declines strongly over the duration of the assay. As a result, a varying proportion of the cell material is removed as the assay buffer decreases.

For characterization of inhibitors on viable cells, cells were incubated in a black 96-well plate with 160 µL of assay buffer (pre-tempered to 37°C), 10 µL of DTT in assay buffer (10 mM) and 10 µL of CA-074 solution (0.2 mM in assay buffer) or 10 µL of inhibitor stock solution in assay buffer containing 10% DMSO for 30 min at 37°C. Subsequently, the measurement was carried out as described above.

Methods for HPLC analysis and purification

a) Analytical RP-HPLC

System **A**: AlphaCrom consisting of the following components

pump (2x):	Varian, PrepStar 218 Solvent Delivery Module
UV/VIS detector:	Varian, ProStar 325
wave length:	254 nm
Software:	Varian Galaxy Chromatography Workstation
stationary phase:	Varian, Dynamax 250 x 21.4 mm, Microsorb 60-8 C18
eluent:	A: 0.1% TFA in water
	B: 0.1% TFA in CH_3CN
elution mode:	Gradient (see Tables below)
flow rate:	1 mL/min

Table S6: Optimized gradient for system A (25 - 75% acetonitrile)

t (min)	% (B)
0-5	25
5-25	75
25-26	95
26-31	95
31-32	25
32-34	25

Table S7: Optimized gradient for system A (5 - 55% acetonitrile)

t (min)	% (B)
0-5	5
5-25	55
25-26	95
26-31	95
31-32	5
32-34	5

System **B**: Shimdazu LC-20A Prominence HPLC consisting of the following components

degasser unit:	DGU-20A5R
pump (2x):	LC-A20R
sample manager:	SIC-20ACHT
column oven:	CTO-20AC
PDA detector:	SPD-M20A
communication-bus module:	CBM-20A
stationary phase:	Aeris Peptide 5 µm XB-C18 columns (100 Å, 250x4.6 mm and 250x21.2 mm)
eluent:	A: 0.1% TFA in water<br>B: 0.1% TFA in CH_3CN
elution mode:	Gradient (see Table below)
flow rate:	1 mL/min

Table S8: Gradient for system B (35 - 85% acetonitrile)

t (min)	% (B)
0-3	35
3-17	85
17-18	95
18-21	95
21-22	35
22-25	35

System **C**: A Waters ACQUITY UPLC I class system including a ACQUITY UPLC PDA e λ detector coupled to a Xevo TQ-S mass spectrometer) was used. A ACQUITY UPLC BEH C18 column (1.7 µm, 130 Å, 100x2.1 mm, equipped with a ACQUITY UPLC BEH C18 VanGuard Pre-column, 1.7 µm, 130 Å, 5x2.1 mm) was used as stationary phase. A binary gradient system of 0.1% CH_3COOH/water (solvent A) and 0.1% CH_3COOH in CH_3CN/CH_3OH (1:1, v/v, solvent B) at a flow rate of 0.4 mL/min served as the eluent.

Table S9: Gradient for system C (25 - 75% acetonitrile)

t (min)	% (B)
0-0.5	25
0.5-5.5	75
5.5-6	95
6-7	95
7-8	25
8-8.5	25

b) Semi-preparative HPLC

Purification of the final products by semi-preparative RP-HPLC was carried out on System **A** using the following separation column:

Stationary phase: Varian, Dynamax 250 x 21.4 mm, Microsorb 60-8 C18

Flow rate: 10 mL/min

The separation method was adapted for each product. The solvent composition at which the desired product eluted was determined by analytical HPLC, and the gradient for semi-preparative HPLC was chosen so that the product eluted after two-thirds of the gradient time. The purified products were isolated form solvent by freeze-drying using the Alpha 2-4 LSC lyophilizer from CHRIST.

Proof of enantiomeric purity of Garner's aldehyde by Mosher analysis

Derivatization by acylation with enantiopure α-methoxy-α-trifluoromethylphenylacetic acid (Mosher's acid, Figure S61) represents a well-established method the assessing the enantiomeric purity and determining the absolute configuration of alcohols and amines on the basis of ^{1}H and ^{19}F NMR spectroscopy.[11]

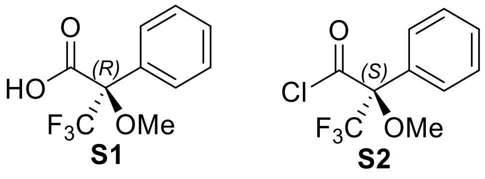

Figure S61: Chemical structures of (*R*)-Mosher's acid (**S1**, left) and the derived acyl chloride (MTPCL (**S2**), right).

To assay the enantiopurity of the obtained Garner's aldehyde **46b**, it was reduced back to Garner's alcohol by treatment with $NaBH_4$. The resulting hydroxy group was acylated with MTPA-Cl (Scheme S2).

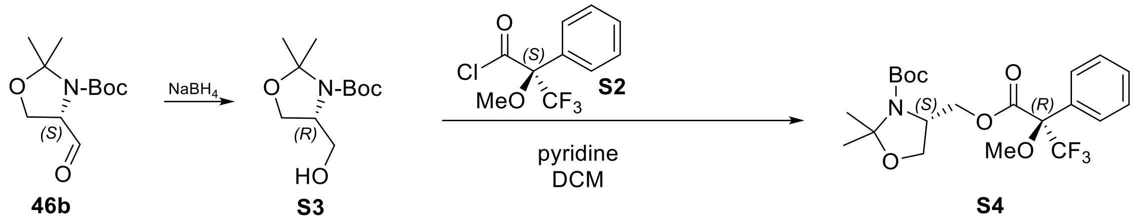

Scheme S1: Derivatization of the Garner alcohol with MTPA-Cl.

In addition to the Mosher ester derived from the back-reduced Garner aldehyde (compound **S4** in Scheme S1) two other reference compounds were synthesized. First, the Garner alcohol directly prepared from L-serine (without oxidation step) was subjected to acylation with **S2** (Scheme S2). As a second reference compound, the Garner alcohol was prepared as a racemic mixture, starting from racemic D-/L-serine and subjected to acylation to obtain Mosher ester S4'' (Scheme S2).

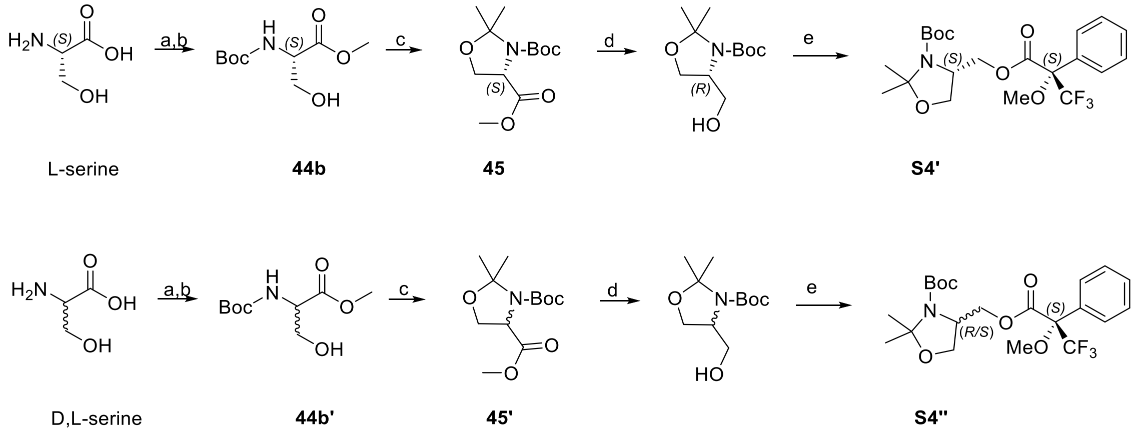

Scheme S2: Synthesis schemes for the reference compound of the stereochemically pure or the racemic Garner alcohol. Reagents and conditions: a) acetyl chloride, MeOH, reflux; b) Boc$_2$O, TEA, THF, RT (93% via two steps); c) DMP, BF$_3$-OEt$_2$, acetone, RT; (84%); d) LiAlH$_4$, THF, RT (94%); e) Mosher acid chloride; Pyridine, DCM (90%).

The ring methylene protons in the diastereomeric Mosher esters derived from the enantiomeric Garner alcohol resonate at distinct chemical shifts, which is mainly due to differential shielding by the phenyl moiety. In particular, the corresponding signals for the derivatized *S* enantiomer are high field-shifted compared to the *R* enantiomer,[12-13] which was confirmed by the ^{1}H NMR spectra of **S4'** and **S4''**.

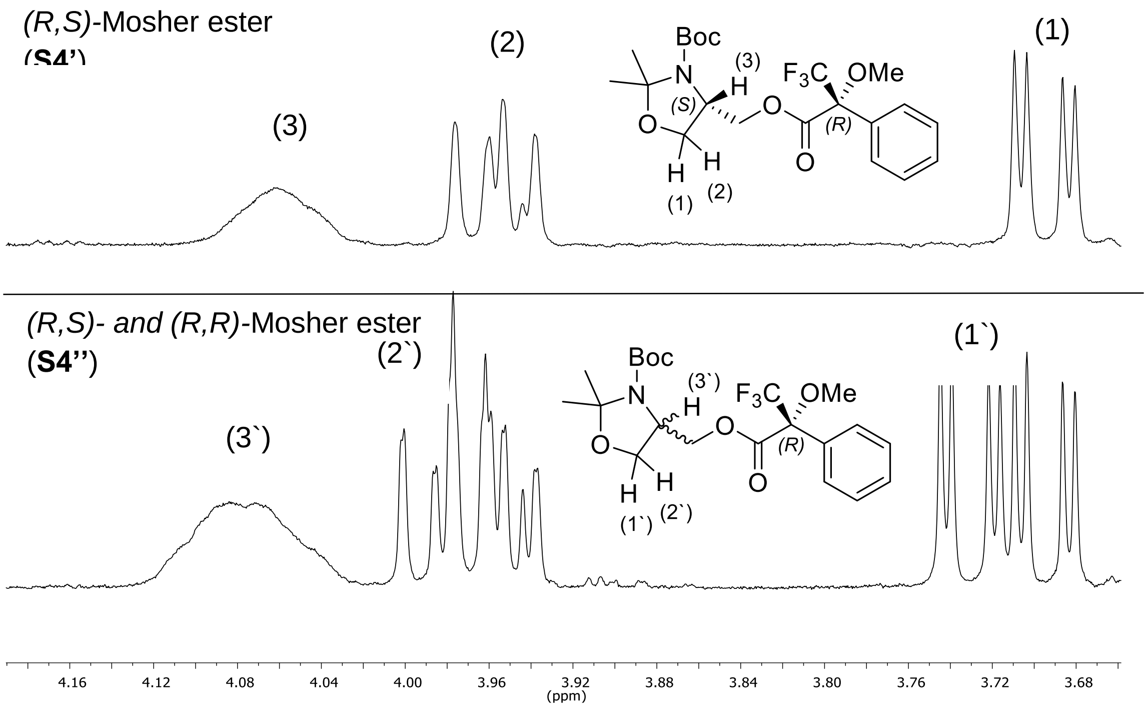

Figure S62: Section of the ^{1}H NMR spectra of the two derivatized Garner alcohols. Derivatized Garner alcohol **S4'** (top) and derivatized rac. Garner alcohol **S4''** (bottom). Due to the influence of the phenyl ring, there is a down-field shift of the (*R*,*R*)-Mosher ester.

Comparing the ^{1}H and ^{19}F NMR spectra of **S4**, **S4'** and **S4''** recorded at 80 °C (Figure S63; high temperature measurement was necessary to prevent peak doubling due to hindered rotation of the Boc carbamate C-N bond) indicates that the spectra of **S4'** and **S4** do not differ. Thus, the obtained Garner aldehyde can be judged enantiomerically pure, even though the detection limit for the undesired diastereomer was not assessed.

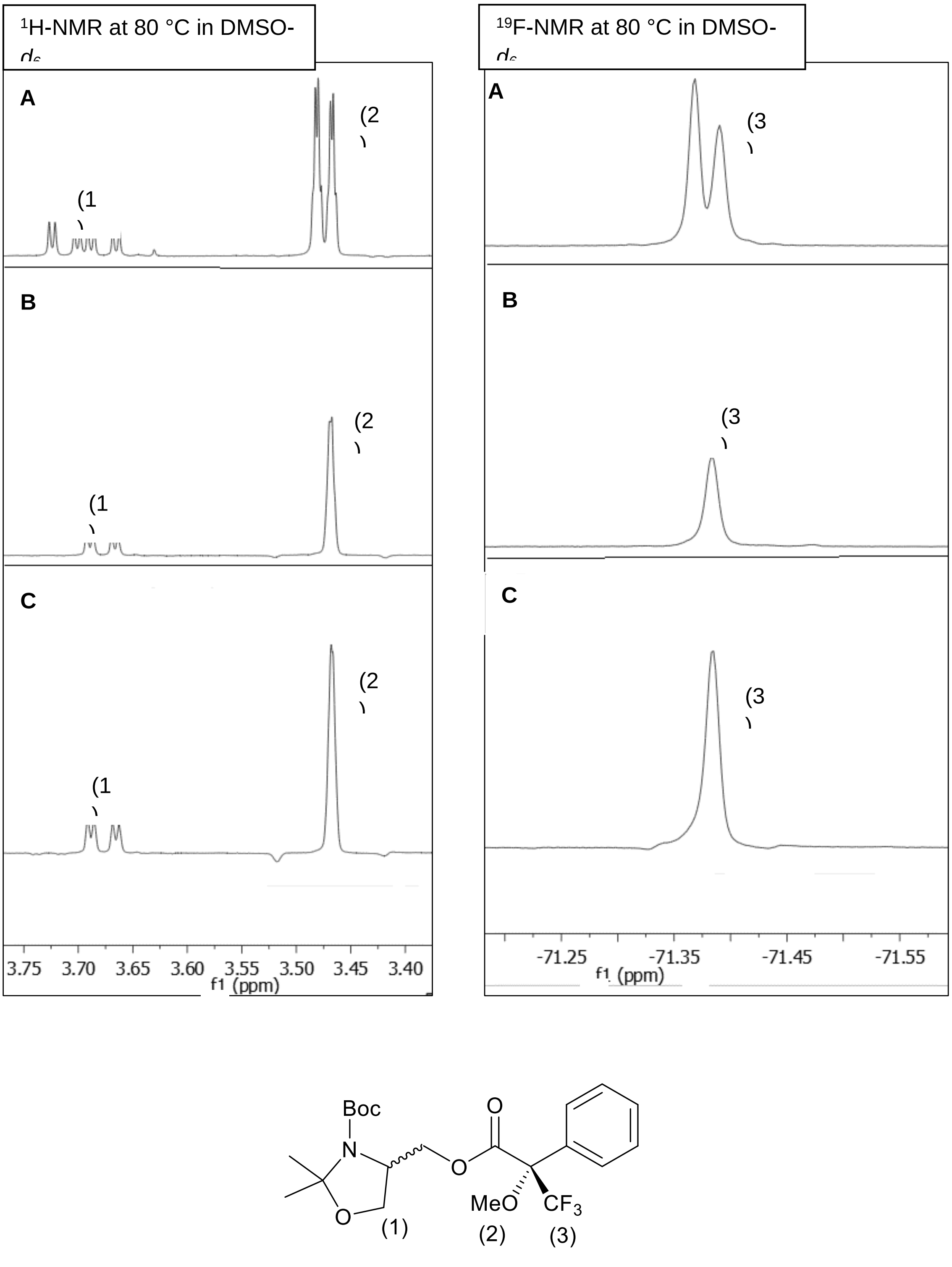

Figure S63: NMR spectra of Garner alcohol-derived Mosher esters of different stereochemical composition.
The figure shows the ^{1}H NMR (left) and ^{19}F NMR spectra (right) of the derivatized compounds **S4''** (racemic Garner alcohol), **S4** (back-reduced Garner aldehyde), and **S4'** (stereoisomerically pure Garner alcohol). Bottom: Structure for signal assignment by In each case, the spectra were measured in DMSO-d_6 at 80 °C. Signal assignment is by numbering the compound below.

Xray crystal structure of Boc-serine-derived alkyne 48

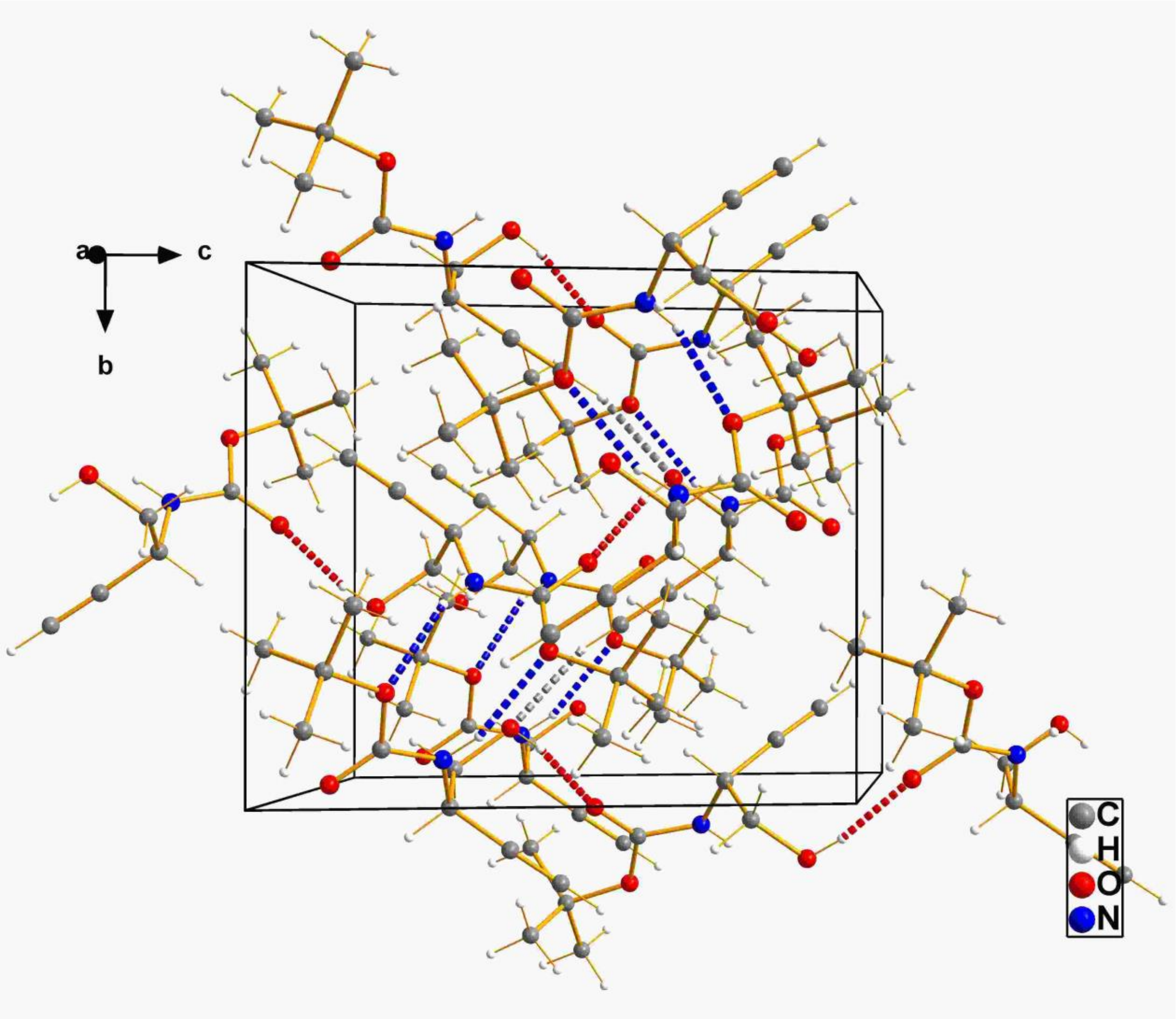

Figure S64: Packing and network of intermolecular contacts in the unit cell of compound **48**. Hydrogen bonds and other contacts below the distance of van-der-Waals radii are indicated by dotted lines. In addition to the shortest hydrogen bond O4−H···O2' mentioned in the main text, further H-bonds, in particular O1−H···O5, N1−H···O6, and N2−H···O3, interconnect the entities in the crystal and lead to a 3D hydrogen bonded network of molecules (as shown above). In detail, the D···A distances are 2.763(1) Å for O1−H1B···O5' (': x, y, z-1), 3.100(1) Å for N1−H1C···O6' (': −x+1, y+1/2, −z+1), and 3.079(1) Å for N2−H2A···O3' (': −x+1, y−1/2, −z+1). Furthermore, weaker hydrogen bond-like intermolecular interactions are present in the crystal between (alkyne)C−H and neighboring O atoms: D···A for C3−H3···O1' (': −x+1, y−1/2, −z) 3.139(1) Å and C12−H12···O4' (': −x+1, y+1/2, −z+1) 3.241(1) Å. Similar values are observed for polar contacts involving the methylidyne group and oxygen atoms in the crystalline state of other alkynes.[14] These crystallographic findings indicate the propensity of the alkyne C-H bond for hydrogen bond-like interactions, which might also contribute to molecular recognition in the active site of enzymes.

Experimental procedures and analytical data for synthesized compounds

1 Synthesis of Dipeptide Nitriles 1a-e (Scheme 1)

Allyl 3-(chloromethyl)benzoate (3a)

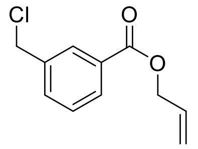

ESI (+):

m/z = 211.00 ([M+H]$^+$)

M ($C_{11}H_{12}ClO_2^+$, monoisotopic): 211.05

3-(Chloromethyl)benzoic acid (2.00 g, 11.72 mmol, 1 eq.) and K_2CO_3 (1.94 g, 14.04 mmol, 1.2 eq.) were dissolved in acetone (20 mL). Then allyl bromide (2.03 mL, 22.98 mmol, 2 eq.) was added slowly. The solution was heated at reflux for 2h and the reaction progress monitored *via* thin layer chromatography. After the solution cooled to room temperature, the mixture was filtered over Celite and the solvent evaporated. The resulting yellow oil was purified *via* preparative column chromatography (petroleum ether/ ethyl acetate = 1:0.02; to 1:0.05) to afford allyl 3-(chloromethyl)benzoate (**3a**) as a yellow oil (2.03 g). The purified product contained 1% allyl 3-(bromomethyl)benzoate, which did not interfere with the following reactions.

^{1}H NMR (400 MHz, $CDCl_3$) δ = 8.08 (t, ^4J = 1.5 Hz, 1H, H-2 aromat.), 8.04 – 8.01 (m, 1H, H-6 aromat.), 7.62 – 7.59 (m, 1H, H-4 aromat.), 7.46 (t, ^3J = 7.7 Hz, 1H, H-5 aromat.), 6.10 – 5.99 (m, 1H, C*H*=CH$_2$), 5.45 – 5.39 (m, 1H, CH=CHH_{trans}), 5.33 – 5.28 (m, 1H, CH=CH_{cis}H), 4.86 – 4.82 (m, 2H, CH_2OCO), 4.63 (s, 2H, CH_2Cl).

^{13}C-NMR (101 MHz, $CDCl_3$) δ = 165.87 (COO), 138.04, 133.23, 132.24, 130.88, 129.85, 129.07, 118.64, 65.91 (CH_2O), 45.67 (CH_2Cl).

^{1}H NMR data are in agreement to published data.[15]

Allyl 3-(iodomethyl)benzoate (3b)

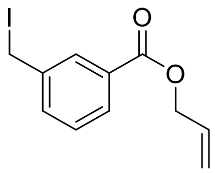

ESI (+):

m/z = 302.83 ([M+H]$^+$)

M ($C_{11}H_{12}IO_2^+$, monoisotopic): 302.99

The oily mixture of compound **3a** and allyl 3-(bromomethyl)benzoate (2.00 g) was taken up in acetone (20 mL), NaI (3.60 g, 24.02 mmol, 2 eq.) was added under stirring and the resulting mixture stirred for 5.5 h at room temperature. The progress of the reaction was monitored by RP-HPLC. After complete conversion of the starting material, the precipitate was filtered off and the solvent was removed *in vacuo*. Subsequently, the residue was taken up in diethyl ether (20 mL) and washed with water (1 × 10 mL), Na_2SO_3 solution (5%, 1 × 10 mL) and saturated NaCl solution (1 × 10 mL). After drying over Na_2SO_4, the solvent was removed in vacuo and the obtained residue was dried under very low pressure (oil pump) for 5 h to furnish allyl

3-(iodomethyl)benzoate (**3b**) as a white crystalline solid (2.59 g, 8.58 mmol, 72% over 2 steps).

^{1}H NMR (400 MHz, $CDCl_3$) δ = 8.06 (t, ^{4}J = 1.8 Hz, 1H, H-2 aromatic), 7.96 – 7.92 (m, 1H, H-6 aromatic), 7.59 – 7.54 (m, 1H, H-4 aromatic), 7.39 (t, ^{3}J = 7.7 Hz, 1H, H-5 aromatic), 6.10 – 5.98 (m, 1H, C*H*=CH_2), 5.44 – 5.38 (m, 1H, CH=CHH_{trans}), 5,32 – 5,27 (m, 1H, CH=CH_{cis}H), 4.84 – 4.79 (m, 2H, CH_2OCO), 4.47 (s, 2H, CH_2I).

^{13}C-NMR (101 MHz, $CDCl_3$) δ = 165.80 (COO), 139.95, 133.44, 132.23, 130.94, 129.84, 129.22, 129.17, 118.66, 65.91 (CH_2O), 4.29 (CH_2I).

^{1}H NMR data are in agreement to published data.[16]

***N*-(*tert*-Butyloxycarbonyl)-*O*-(3-(allyloxycarbonyl)benzyl)-L-serine (4)**

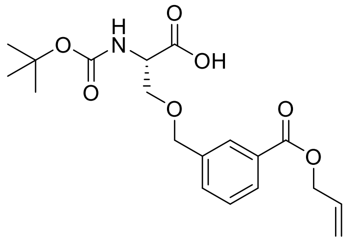

ESI (+):

m/z = 402.08 ([M+Na]$^{+}$)

M ($C_{19}H_{25}NO_7Na^{+}$, monoisotopic): 402.15

NaH in 60% mineral oil (0.92 g, 15.33 mmol, 2 eq.) was suspended in pentane (3 × 3 mL) under argon atmosphere. The supernatant was removed to remove the mineral oil. The cleansed NaH was suspended in dry DMF (15mL) and cooled to 0°C in an ice bath. Boc-L-serine (1.57 g, 7.62 mmol, 1 eq.) was added over 10 min while vigorously stirring and the solution stirred 30 min at room temperature (exposure of Boc-serine to NaH prior to addition of **3b** should be kept as short as possible, since longer deprotonation times resulted in the formation of multiple side products including the *O*,*O*'-dialkylated product). Then the solution was cooled to -10°C using an ice/sodium chloride cooling bath. Allyl 3-(iodomethyl)benzoate (**3b**, 2.30 g, 7.62 mmol, 1 eq.) in dry DMF (10 mL) was added dropwise over 15 min. Then the solution was stirred for further 10 min at 0°C and 30 min at room temperature. The reaction progress was monitored *via* thin layer chromatography and the solution acidified to pH 3 with 1 M HCl. The solution was extracted with ethyl acetate (4 × 50 mL), the combined organic layers washed with brine (40 mL) and dried over Na_2SO_4. The solvent was evaporated and the remaining solvent removed *in vacuo*. The crude product was purified via preparative column chromatography (1% acetic acid in petroleum ether/ethyl acetate (4:1) to 1% acetic acid in petroleum ether/ethyl acetate (1:1)). The solvent was evaporated and the product washed with toluol (4 × 30 mL). The product was dried *in vacuo* to yield *N*-(*tert*-Butyloxycarbonyl)-*O*-(3-(allyloxycarbonyl)benzyl)-L-serine (**4**) as clear oil (0.85 g, 2.24 mmol, 29%).

^{1}H NMR (400 MHz, $CDCl_3$) δ = 8.00 – 7.96 (m, 2H, H-2,4 aromatic), 7.51 (d, ^{3}J = 7.9 Hz, 1H, H-6 aromatic), 7.42 (t, ^{3}J = 7.6 Hz, 1H, H-5 aromatic), 6.10 – 5.98 (m, 1H, C*H*=CH_2), 5.46 – 5.37 (m, 2H, N_αH, CH=CHH_{trans}), 5.33 – 5.27 (m, 1H, CH=CH_{cis}H),

4.86 – 4.79 (m, 2H, CH_2OCO), 4.62 (d, ^2J = 12.3 Hz, 1H, Ar-CH*H*O), 4.53 (d, ^2J = 12.2 Hz, 1H, Ar-C*H*HO), 4.50 (broad s, 1H, C_αH), 3.94 (broad d, ^2J = 9.2 Hz, 1H, C_βH*H*), 3.73 (dd, ^2J = 9.4, ^3J = 3.7 Hz, 1H, C_β*H*H), 1.45 (s, 9H, CH_3).

^{13}C-NMR (101 MHz, $CDCl_3$) δ = 173.94 (COOH), 166.32 (COO-allyl), 155.95 (CO-Boc), 137.91, 132.37, 132.27, 130.49, 129.35, 128.94, 128.82, 118.60, 80.74 ($C_{quart.}$ Boc), 73.04, 69.96, 65.89, 53.83 (C_α Ser), 28.43 ($(CH_3)_3$).

N-(*tert*-Butyloxycarbonyl)-*O*-(3-(allyloxycarbonyl)benzyl)serinamide (5)

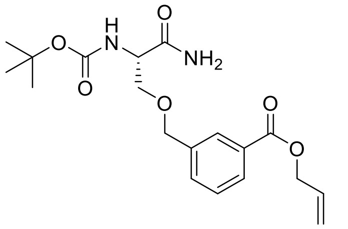

ESI (+):

m/z = 401.13 ($[M+Na]^+$), 279.16 ($[M\text{-Isobutylene-}CO_2+H]^+$)

M ($C_{19}H_{24}N_2O_6Na^+$, monoisotopic): 401.17

The synthesis was accomplished according to GP I. using 400 mg **4** (1.05 mmol, 1 eq.), 347 µL NMM (3.16 mmol, 3 eq.), 150 µL iBCF (1.16 mmol, 1.1 eq.) and 390 µL NH_3 (aqueous solution, 25%; 5.25 mmol, 5 eq.) in 20 mL dry THF to yield *N*-(*tert*-Butyloxycarbonyl)-*O*-(3-(allyloxycarbonyl)benzyl)serinamide (**5**) as a colorless oil (0.39 g, 1.02 mmol, 97%).

^{1}H-NMR (400 MHz CD_3CN) δ = 8.01 - 7.94 (m, 2H, H-2,4 aromatic), 7.61 - 7.57 (m, 1H), 7.49 (t, ^3J = 7.7 Hz, 1H, H-5 aromat), 6.45 (s, 1H, N*H*), 6.13 - 6.02 (m, 1H, C*H*=CH_2), 5.82 (s, 1H, CON*H*H), 5.63 (s, 1H, CONH*H*), 5.42 (dq, ^3J = 17.3, ^4J = 1.6 Hz, 1H, CH=CHH_{trans}), 5.28 (dq, ^3J = 10.5, ^4J = 1.4 Hz, 1H, CH=CH_{cis}H), 4.81 (dt, ^3J = 5.5, ^4J = 1.5 Hz, 2H, CH_2-Allyl), 4.59 - 4.57 (m, 2H, Ar-CH_2O), 4.22 - 4.14 (m, 1H, C_α*H*), 3.77 - 3.72 (m, 1H, C_βH*H*), 3.70 - 3.64 (m, 1H, C_β*H*H), 1.41 (s, 9H, $(CH_3)_3$).

^{13}C-NMR (101 MHz, CD_3CN) δ = 173.05 (*C*ONH$_2$), 166.71 (COO-allyl), 156.43 (CO-Boc), 139.93, 133.65, 133.31, 131.36, 129.67, 129.54, 129.39, 118.37 (C=*C*H$_2$), 80.07 (*C*(CH_3)$_3$), 73.07 (CH_2O), 71.13 (C_β Ser), 66.27 (CH_2 allyl), 55.21 (C_α Ser), 28.50 (C(*C*H$_3$)$_3$).

^{1}H NMR data are in agreement to published data.[16]

O-(3-(Allyloxycarbonyl)benzyl)serinamide (6)

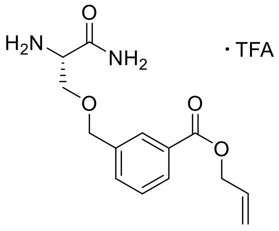

ESI (+):

m/z = 279.14 ($[M+H]^+$)

M ($C_{14}H_{19}N_2O_4^+$, monoisotopic): 279.13

The synthesis was accomplished according to GP II. using 0.40 g **5** (1.07 mmol) und 10 mL CH_2Cl_2/TFA (1:1). *O*-(3-(Allyloxycarbonyl)benzyl)-L-serinamide (**6**) was obtained as a yellow oil (0.50 g, quantitative yield, contained TFA).

^{1}H NMR (400 MHz, DMSO-*d₆*) δ = 8.17 (broad s, 3H, NH_3^+), 7.99 – 7.96 (m, 1H, H-2 aromatic), 7.94 (dt, ^3J = 7.7, ^4J = 1.4 Hz, 1H, H-4 aromatic), 7.85 (broad s, 1H, NH), 7.67 (d, ^3J = 7.8 Hz, 1H, H-6 aromatic), 7.63 (broad s, 1H, NH), 7.55 (t, ^3J = 7.7 Hz, 1H, H-5 aromatic), 6.11 – 6.00 (m, 1H, C*H*=CH_2), 5.44 – 5.37 (m, 1H, CH=CH_{trans}), 5.31 – 5.26 (m, 1H, CH=CH_{cis}H), 4.83 – 4.80 (m, 2H, CH_2 allyl), 4.67 – 4.56 (m, 2H, CH_2O), 4.04 – 3.92 (m, 1H, C_αH), 3.83 – 3.74 (m, 2H, C_βH).

^{13}C NMR (101 MHz, DMSO-*d₆*) δ = 167.98 (CO), 165.27 ($CONH_2$), 138.33, 132.69, 132.58, 129.64, 128.84, 128.52, 128.42, 118.02 (*C*H=CH2), 71.83 (CH_2O), 68.46 (C_β), 65.15 (CH_2 allyl), 52.39 (C_α).

N-(*tert*-Butyloxycabonyl)-3-methyl-L-phenylalanyl-*O*-(3-(allyloxycarbonyl)benzyl)-L-serinamide (7a)

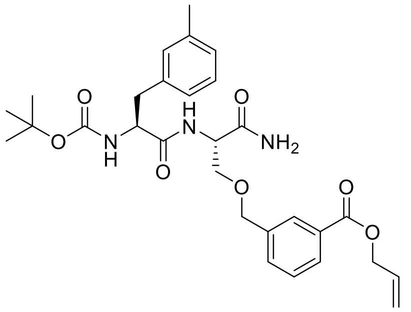

ESI (+):

m/z = 540.25 ([M+H]$^+$),

440.21 ([M-Isobutylene-CO_2+H]$^+$)

M ($C_{29}H_{38}N_3O_7^+$, monoisotopic): 540.27

The synthesis was accomplished according to GP III. using 0.10 g **6** (0.26 mmol, 1 eq.), 0.11 g Boc-(3-Me)Phe-OH (0.39 mmol, 1.5 eq.), 178 µL DiPEA (1.02 mmol, 4 eq.) and 0.18 g PyBOP (0.39 mmol, 1.5 eq.) in 10 mL THF. The crude product was purified *via* preparative column chromatography (2% methanol/CH_2Cl_2 to 3% methanol/CH_2Cl_2). *N*-(*N*-(*tert*-Butoxycabonyl)-3-methyl-L-phenylalanin)-*O*-((3-(allyloxycarbonyl)phenyl)methyl)-L-serinamide (**7a**) was obtained as white solid (0.08 g, 0.15 mmol, 57%).

^{1}H NMR (400 MHz, $CDCl_3$) δ = 8.00 (dt, ^3J = 7.3, ^4J = 1.7 Hz, 1H, H-4 aromatic), 7.96 (s, 1H, H-2 aromatic), 7.48 – 7.44 (m, 1H, H-6 aromatic), 7.42 (t, ^3J = 7.5 Hz, 1H, H-5 aromatic), 7.23 – 7.17 (m, 1H, H-5 aromatic Phe), 7.09 – 7.03 (m, 3H, H-2,4,6 aromatic Phe), 6.85 (d, ^3J = 8.0 Hz, 1H, N_αH Ser), 6.70 (s, 1H, CONH*H*), 6.11 – 5.99 (m, 1H, C*H*=CH_2), 5.45 – 5.38 (m, 1H, CH=CHH_{trans}), 5.33 – 5.25 (m, 2H, CON*H*H, CH=CH_{cis}H), 4.86 – 4.82 (m, 2H, CH_2 allyl), 4.61 (d, ^2J = 12.0 Hz, 1H, Ar-CH*H*O), 4.58 – 4.52 (m, 1H, C_αH Ser), 4.46 (d, ^2J = 11.9 Hz, 1H, Ar-C*H*HO), 4.31 – 4.25 (m, 1H, C_αH Phe), 4.10 (d, ^3J = 8.4 Hz, 1H, C_βH*H* Ser), 3.53 – 3.48 (m, 1H, C_β*H*H Ser), 3.17 – 3.09

(m, 1H, C_{β}H*H* Phe), 3.01 – 2.93 (m, 1H, C_{β}H Phe), 2.31 (s, 3H, CH_3), 1.30 (s, 9H, $(CH_3)_3$).

^{13}C NMR (101 MHz, $CDCl_3$) δ = 172.14, 171.40, 166.26 (COO allyl), 156.36 (OOCNH), 138.72, 138.27, 136.24, 132.42 (C-6 aromatic), 132.23 (*C*=CH_2), 130.61, 130.10 (C aromatic Phe), 129.27 (C-4 aromatic), 128.95, 128.91, 128.79, 128.16 (C aromatic Phe), 126.32 (C aromatic Phe), 118.71 (C=*C*H_2), 81.04 (C_{quart}), 72.91 (CH_2O), 69.56 (C_{β} Ser), 65.93 (CH_2 allyl), 57.02 (C_{α} Phe), 52.70 (C_{α} Ser), 37.64 (C_{β} Phe), 28.28 ($(CH_3)_3$), 21.51 (CH_3).

***N*-(*tert*-Butyloxycarbonyl)-3-iodphenylalanyl-*O*-(3-(allyloxycarbonyl)benzyl)-L-serinamide (7b)**

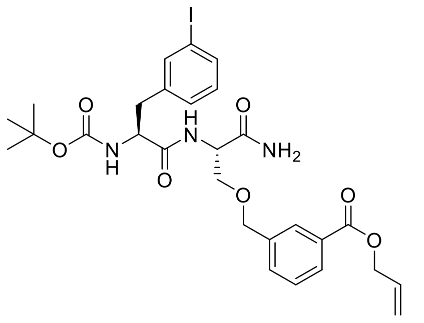

HR-MS ESI (+):

m/z = 674.1332 ($[M+Na]^+$)

M ($C_{28}H_{34}IN_3NaO_7^+$, monoisotopic): 674.1333

The synthesis was accomplished according to GP III. using 0.05 g **7b** (0.13 mmol, 1 eq.), 0.08 g Boc-(3-I)Phe-OH (0.19 mmol, 1.5 eq.), 90 µL DIPEA (0.52 mmol, 4 eq.) and 0.10 g PyBOP (0.19 mmol, 1.5 eq.) in 10 mL THF. The crude product was purified *via* semipreparative HPLC to yield *N*-(*tert*-Butyloxycarbonyl)-3-iodphenylalanyl-*O*-(3-(allyloxycarbonyl)benzyl)-L-serinamide (**7b**) as a white solid (0.04 g, 0.06 mmol, 50%).

^{1}H NMR (600 MHz, CD_3CN) δ = 7.98 (s, 1H, H-2 BnCOO), 7.96 (d, ^3J = 7.8 Hz, 1H, H-4 BnCOO), 7.65 (s, 1H, H-2 Phe), 7.61 – 7.57 (m, 2H, H-4 Phe, H-6 BnCOO), 7.49 (t, ^3J = 7.7 Hz, 1H, H-5 BnCOO), 7.26 (d, ^3J = 7.5 Hz, 1H, H-6 Phe), 7.12 (d, ^3J = 7.6 Hz, 1H, NH), 7.07 (t, ^3J = 7.8 Hz, 1H, H-5 Phe), 6.46 (s, 1H, CONH*H*), 6.12 – 6.02 (m, 1H, C*H*=CH_2), 5.83 (s, 1H, CON*H*H), 5.71 (s, 1H, NH), 5.47 – 5.36 (m, 1H, CH=CH_{trans}), 5.32 – 5.25 (m, 1H, CH=CH_{cis}), 4.81 (dt, ^3J = 5.5 Hz, ^4J = 1.5 Hz, 2H, CH_2 allyl), 4.58 (s, 2H, CH_2O), 4.42 (s, 1H, C_{α}H Ser), 4.28 – 4.22 (m, 1H, C_{α}H Phe), 3.87 – 3.81 (m, 1H, C_{β}*H*H Ser), 3.67 – 3.60 (m, 1H, C_{β}H*H* Ser), 3.12 – 3.05 (m, 1H, C_{β}*H*H Phe) , 2.85 – 2.76 (m, 1H, C_{β}H*H* Phe), 1.30 (s, 9H, CH_3).

^{13}C NMR (151 MHz, CD_3CN) δ = 172.39 (CO), 172.13 (CO), 166.83 (COO), 156.83 (CO), 141.20, 139.93, 139.24, 136.64, 133.60, 133.33, 131.35, 131.28, 129.81, 129.72, 129.55, 129.42, 118.26 (CD_3CN, C=*C*H_2), 94.71 (C-I), 80.48 (*C*$(CH_3)_3$), 73.11 (CH_2O), 70.72 (C_{β} Ser), 66.35 (CH_2 allyl), 56.95 (C_{α} Phe), 53.83 (C_{α} Ser), 37.49 (C_{β} Phe), 28.41 (CH_3).

3-Methyl-L-phenylalanyl-*O*-(3-(allyloxycarbonyl)benzyl)-L-serinamide (8a)

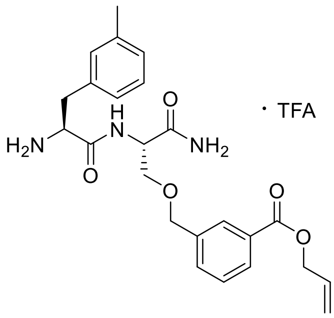

ESI (+):

m/z = 440.19 ([M+H]$^+$)

M ($C_{24}H_{30}N_3O_5^+$, monoisotopic): 440.22

The synthesis was accomplished according to GP II. using 0.08 g **7a** (0.14 mmol) and 10 mL TFA/CH_2Cl_2 (1:1). 3-Methyl-L-phenylalanyl-O-(3-(allyloxycarbonyl)benzyl)-L-serinamide (**8a**) was obtained as a white solid (0.08 g, quantitative yield, contained TFA).

^{1}H NMR (400 MHz, DMSO-*d$_6$*) δ = 8.78 (d, ^3J = 8.1 Hz, 1H, NH), 8.08 (s, 3H, NH_3^+), 7.96 – 7.90 (m, 2H, H-2,4 aromatic), 7.63 (d, ^3J = 7.8 Hz, 1H, H-6 aromatic), 7.53 (t, ^3J = 7.6 Hz, 1H, H-5 aromatic), 7.45 (s, 1H, CON*H*H), 7.24 (s, 1H, CONH*H*), 7.20 (t, ^3J = 7.5 Hz, 1H, H-5, aromatic Phe), 7.10 – 7.03 (m, 3H, H-2,4,6 aromatic Phe), 6.11 – 5.99 (m, 1H, C*H*=CH_2), 5.44 – 5.36 (m, 1H, CH=CHH_{trans}), 5.31 – 5.26 (m, 1H, CH=CH_{cis}H), 4.83 – 4.79 (m, 2H, CH_2 allyl), 4.60 (s, 2H, CH_2O), 4.57 – 4.51 (m, 1H, C_αH Ser), 4.17 – 4.08 (m, 1H, C_αH Phe), 3.71 – 3.60 (m, 2H, $C_\beta H_2$ Ser), 3.12 – 3.04 (m, 1H, C_β*H*H Phe), 2.93 – 2.85 (m, 1H, C_βH*H* Phe), 2.28 (s, 3H, CH_3).

^{13}C NMR (101 MHz, DMSO-*d$_6$*) δ = 170.49, 168.01, 165.30, 138.86, 137.58, 134.68, 132.60 (*C*=CH_2), 132.45 (C_6 aromatic), 130.15 (C aromatic Phe), 129.64, 128.84 (C_5 aromatic), 128.42 (C aromatic), 128.37 (C aromatic), 128.16 (C aromatic), 127.80 (C aromatic Phe), 126.59 (C aromatic Phe), 118.04 (C=*C*H_2), 99.68, 71.60 (CH_2O), 70.10 (C_β Ser), 65.16 (CH_2 allyl), 53.26 (C_α Phe), 52.68 (C_α Ser), 36.93 (C_β Phe), 21.02 (CH_3).

3-Iodo-L-phenylalanyl-*O*-(3-(allyloxycarbonyl)benzyl)-L-serinamide (8b)

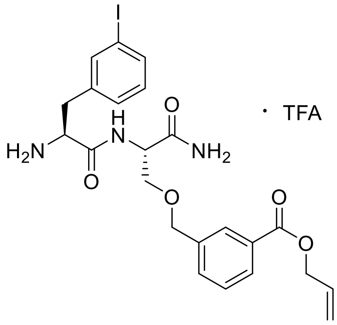

HR-MS ESI (+):

m/z = 574.0805 ([M+Na]$^+$)

M ($C_{23}H_{26}IN_3NaO_5^+$, monoisotopic): 574.0809

The synthesis was accomplished according to GP II. using 0.04 g **7b** (0.06 mmol) and 10 mL TFA/CH_2Cl_2 (1:1). 3-Iodo-L-phenylalanyl-*O*-(3-(allyloxycarbonyl)benzyl)-L-serinamide (**8b**) was obtained as a white solid (0.04 g, 92%, contained TFA).

^{1}H NMR (400 MHz, CD_3CN) δ = 7.98 (s, 1H, H-2 BnCOO), 7.97 – 7.92 (m, 1H, H-4 BnCOO), 7.74 – 7.70 (m, 1H, H-2 Phe), 7.68 – 7.64 (m, 1H, H-4 Phe), 7.61 – 7.53 (m, 2H, H-6 BnCOO, NH), 7.48 (t, ^3J = 7.6 Hz, 1H, H-5 BnCOO), 7.32 (d, ^3J = 7.8 Hz, 1H, H-6 Phe), 7.10 (t, ^3J = 7.8 Hz, 1H, H-5 Phe), 6.40 (s, 1H, CONH*H*), 6.13 – 6.01 (m, 1H, C*H*=CH_2), 5.92 (s, 1H, COON*H*H), 5.46 – 5.38 (m, 1H, CH=CH_{trans}), 5.32 – 5.25 (m, 1H, CH=CH_{cis}), 4.80 (dt, ^3J = 5.5, ^4J = 1.5 Hz, 2H, CH_2 allyl), 4.64 – 4.54 (m, 2H, CH_2O), 4.52 – 4.45 (m, 1H, C_αH Ser), 4.26 (t, ^3J = 6.8 Hz, 1H, C_αH Phe), 3.81 – 3.74 (m, 1H, C_β*H*H Ser), 3.70 – 3.64 (m, 1H, C_βH*H* Ser), 3.25 – 3.15 (m, 1H, C_β*H*H Phe), 3.13 – 3.04 (m, 1H, C_βH*H* Phe).

The NH_3^+ protons were not detectable in the chosen solvent.

^{13}C NMR (101 MHz, CD_3CN) δ = 171.57 (CO), 168.75 (CO), 166.85 (CO), 139.85, 139.59, 137.99, 137.62, 133.61, 133.29, 131.70, 131.31, 130.21, 129.69, 129.51, 129.33, 118.26 (CD_3CN, C=*C*H_2), 95.23 (CI), 73.09 (CH_2O), 70.58 (C_β Ser), 66.35 (CH_2 allyl), 55.28 (C_α Phe), 54.37 (C_α Ser), 37.13 (C_β Phe).

***N*-(2,4-Difluorobenzoyl)-3-methyl-L-phenylalanyl-*O*-(3-(allyloxycarbonyl)-benzyl)-L-serinamide (9a)**

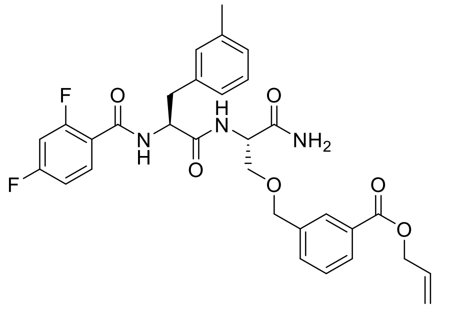

ESI (+):

m/z = 580.19 ([M+H]$^+$)

M ($C_{31}H_{32}F_2N_3O_6^+$, monoisotopic): 579.22

The synthesis was accomplished according to GP IV. using 0.06 g **8a** (0.10 mmol, 1 eq.), 13 µL 2,4-difluorobenzoyl chloride (0,10 mmol, 1 eq.), 33 µL NMM (instead of TEA; 0.20 mmol, 2 eq.) and 10 mL CH_2Cl_2. *N*-(2,4-Difluorobenzoyl)-3-methyl-L-phenylalanyl-*O*-(3-(allyloxycarbonyl)benzyl)serinamide (**9a**) was obtained as a white solid (0.05 g, 0.07 mmol, 65%; contained 22% in P3 trifluoroacetylated side product (**9a-NP**).)

^{1}H NMR (400 MHz, $CDCl_3$) δ = 8.03 – 7.88 (m, 3H, H-6 FBz, H-2,4 aromatic), 7.48 – 7.31 (m, 2H, H-5,6 aromatic), 7.23 – 7.17 (m, 1H, H-5 aromatic Phe), 7.11 – 6.99 (m, 3H, H-2,4,6 aromatic Phe), 6.97 – 6.91 (m, 1H, H-5 FBz), 6.85 – 6.78 (m, 1H, H-3 FBz), 6.68 (d, ^3J = 7.7 Hz, 1H, N_αH Ser), 6.52 (d, ^3J = 6.9 Hz, 1H, NH Phe), 6.37 (s, 1H, CON*H*H), 6.10 – 5.98 (m, 1H, C*H*=CH_2), 5.45 – 5.38 (m, 1H, CH=CHH_{trans}), 5.35 (s, 1H, CONH*H*), 5.32 – 5.27 (m, 1H, CH=CH_{cis}H), 4.83 (m, 2H, CH_2 allyl), 4.79 – 4.72 (m, 1H, C_αH Phe), 4.60 – 4.44 (m, 3H, C_αH Ser, CH_2O), 4.03 – 3.99 (m, 1H, C_βH*H* Ser), 3.54 – 3.47 (m, 1H, C_β*H*H Ser), 3.17 (d, ^3J = 7.2 Hz, 2H, C_βH Phe), 2.31 (s, 3H, CH_3).

^{13}C NMR (101 MHz, $CDCl_3$) δ = 171.79, 171.00, 170.63, 169.25, 166.26, 166.15, 163.34, 163.31, 138.92, 137.99, 135.98, 135.16, 132.25, 132.19, 130.49, 130.08,

129.45, 129.23, 129.06, 128.92, 128.79, 128.71, 128.35, 126.30, 118.64 (C=CH_2), 112.76 (C-5 FBz), 104.56 (C-3 FBz), 72.90 (CH_2O), 69.35 (C_β Ser), 65.87 (CH_2 Allyl), 56.35 (C_α Phe), 52.79 (C_α Ser), 37.85 (C_β Phe), 21.48 (CH_3).

Multiplicity of C-F signals was not considered.

^{19}F NMR (376 MHz, $CDCl_3$) δ = -102.60 (F aromatic), -108.49 (F aromatic).

N-(4-Fluorobenzoyl)-3-methyl-L-phenylalanyl-O-(3-(allyloxycarbonyl)benzyl)-L-serinamide (9b)

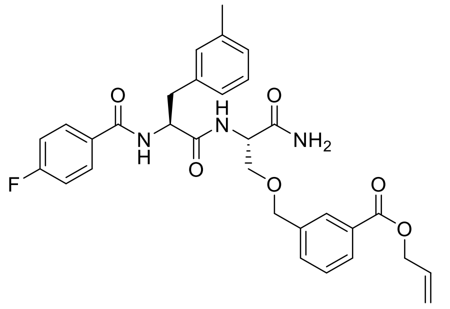

ESI (+):

m/z = 584.02 ([M+Na]$^+$)

M ($C_{31}H_{32}FN_3NaO_6^+$, monoisotopic): 584.22

The synthesis was accomplished according to GP IV. using 0.15 g **8a** (0.27 mmol, 1 eq.), 32 µL 4-fluorobenzoyl chloride (0.27 mmol, 1 eq.), 113 µL TEA (0.81 mmol, 3 eq.) and 25 mL CH_2Cl_2. *N*-(4-Fluorobenzoyl)-3-methyl-L-phenylalanyl-O-(3-(allyloxycarbonyl)benzyl)-L-serinamide (**9b**) was obtained as a white solid (0.13 g, 0.23 mmol, 84%; contained in P3 trifluoroacetylated side product (**9a-NP**, amount not quantified)).

^{1}H NMR (400 MHz, $CDCl_3$) δ = 8.21 – 8.14 (m, 2H, H-3,5 aromatic FBz), 7.98 – 7.94 (m, 1H, H-4 BnCOO), 7.91 – 7.89 (m, 1H, H-2 BnCOO), 7.71 – 7.66 (m, 1H, H-5 aromatic Phe), 7.47 – 7.40 (m, 1H, H-6 aromatic), 7.37 (t, ^3J = 7.5, 0.5 Hz, 1H, H-5 BnCOO), 7.24 – 7.18 (m, 2H, H-2,6 aromatic FBz), 7.13 – 7.00 (m, 3H, H-2,4,6 aromatic Phe), 6.82 (d, ^3J = 7.9 Hz, 1H, NH), 6.40 (s, 1H, CONH*H*), 6.10 – 5.98 (m, 1H, C*H*=CH_2), 5.46 – 5.35 (m, 2H, CH=CHH_{trans}, CON*H*H), 5.34 – 5.29 (m, 1H, CH=CH_{cis}H), 4.86 – 4.82 (m, 2H, CH_2 allyl), 4.79 – 4.74 (m, 1H, C_αH Phe), 4.62 – 4.44 (m, 3H, CH_2O, C_αH Ser), 4.01 (dd, ^3J = 9.2, ^2J = 3.4 Hz, 1H, C_βH*H* Ser), 3.55 – 3.47 (m, 1H, C_β*H*H Ser), 3.24 (dd, ^3J = 13.9, ^2J = 6.5 Hz, 1H, C_βH*H* Phe), 3.16 – 3.06 (m, 1H, C_β*H*H Phe), 2.31 (s, 3H, CH_3).

^{13}C NMR (101 MHz, $CDCl_3$) δ = 171.75, 170.88, 167.29, 166.47, 162.65 (d, $^1J_{C,F}$ = 258.3 Hz, CF), 138.90, 138.17, 136.42, 133.51, 133.41, 132.32, 132.13, 130.46, 130.02, 129.70, 129.61, 129.21, 129.02, 128.81, 128.55, 128.28, 126.22, 118.75 (CH=CH_2), 115.86 (d, $^2J_{C,F}$ = 22 Hz, C-3/5 FBz), 72.63 (CH_2O), 69.25 (C_β Ser), 65.98 (CH_2 allyl), 56.13 (C_α Phe), 52.74 (C_α Ser), 37.87 (C_β Phe), 21.51 (CH_3).

***N*-Diphenylacetyl-3-methyl-L-phenylalanyl-*O*-(3-(allyloxycarbonyl)benzyl)-L-serinamide (9c)**

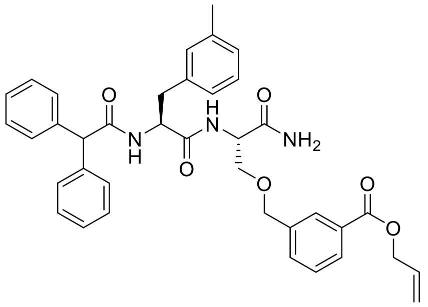

ESI (+):

m/z = 656.28 ([M+Na]$^+$)

M ($C_{38}H_{40}N_3O_6^+$, monoisotopic): 634.29

The synthesis was accomplished according to GP III. using 0.05 g **8a** (0.09 mmol, 1 eq.), 0.03 g diphenylacetic acid (0.14 mmol, 1.5 eq.), 62.9 µL DiPEA (0.36 mmol, 4 eq.), 0.07 g PyBOP (0.14 mmol, 1.5 eq.) and 5 mL THF. *N*-Diphenylacetyl-3-methyl-L-phenylalanyl-*O*-(3-(allyloxycarbonyl)benzyl)-L-serinamide (**9c**) was obtained as a white solid (0.09 g, quantitative yield).

^{1}H NMR (400 MHz, CD_3CN) δ = 7.99 – 7.90 (m, 2H, H-2,4 BnCOO), 7.59 – 7.52 (m, 1H, H-6 BnCOO), 7.52 – 7.43 (m, 1H, H-5 BnCOO), 7.30 – 6.88 (m, 16H, 10 H aromatic diphenyl, 4 H aromatic Phe, 2 NH), 6.29 (s, 1H, CON*H*H), 6.12 – 6.01 (m, 1H, C*H*=CH_2), 5.79 (s, 1H, CONH*H*), 5.46 – 5.37 (m, 1H, CH=CH_{trans}), 5.31 – 5.23 (m, 1H, CH=CH_{cis}), 4.92 (s, 1H, CH diphenyl), 4.83 – 4.76 (m, 2H, CH=CH_2), 4.67 – 4.56 (m, 1H, C_αH Ser), 4.51 (s, 2H, CH_2O), 4.45 – 4.31 (m, 1H, C_αH Phe), 3.80 – 3.70 (m, 1H, C_β*H*H Ser), 3.61 – 3.47 (m, 1H, C_βH*H* Ser), 3.13 – 3.02 (m, 1H, C_β*H*H Phe), 2.90 – 2.76 (m, 1H, C_βH*H* Phe), 2.24 (s, 3H, CH_3).

^{13}C NMR (101 MHz, CD_3CN) δ = 173.10 (CO), 172.30 ($CONH_2$), 171.80 (CO), 166.86 (COO), 140.64, 139.90, 138.96, 138.01, 133.61, 133.31, 131.28, 130.88, 129.69, 129.68, 129.57, 129.51, 129.43, 129.41, 129.35, 129.30, 128.33, 127.91, 127.19, 118.52 (CD_3CN, C=CH_2), 73.02 (CH_2O), 70.64 (C_β Ser), 66.34 (CH_2 allyl), 58.44 (CH diphenyl), 55.84 (C_α Ser), 53.84 (C_α Phe), 37.62 (C_β Phe), 21.42 (CH_3).

Due to overlapping two signals were not identified.

***N*-(4-Fluorobenzoyl)-3-iodo-L-phenylalanyl-*O*-(3-(allyloxycarbonyl)benzyl)-L-serinamide (9d)**

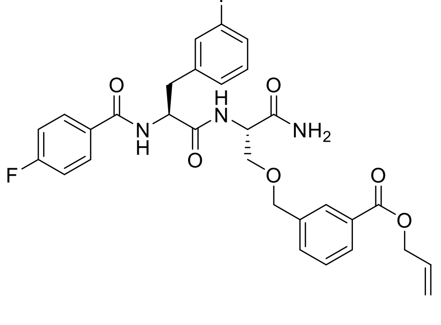

HR-MS ESI (+):

m/z = 696.0975 ([M+Na]$^+$)

M ($C_{30}H_{29}FIN_3NaO_6^+$, monoisotopic): 696.0977

The synthesis was accomplished according to GP IV. using 0.04 g **8b** (0.07 mmol, 1 eq.), 12 µL 4-fluorobenzoyl chloride (0.10 mmol, 1.4 eq.), 28 µL TEA (0.20 mmol, 3 eq.) and 10 mL CH_2Cl_2. *N*-(4-Fluorobenzoyl)-3-iodo-L-phenylalanyl-*O*-(3-(allyloxycarbonyl)benzyl)-L-serinamide (**9d**) was obtained as a white solid (0.04 g, 0.06 mmol, 90%; contained trifluoroacetylated side product, amount not quantified).

^{1}H NMR (400 MHz, CD_3CN) δ = 7.95 – 7.89 (m, 2H, H-2,4 BnCOO), 7.76 – 7.70 (m, 3H, H-2,6 FBz, H-2 Phe), 7.60 – 7.55 (m, 1H, H-4 Phe), 7.53 (d, ^3J = 7.8 Hz, 1H, H-6 BnCOO), 7.41 (t, ^3J = 7.6 Hz, 1H, H-5 BnCOO), 7.36 – 7.29 (m, 2H, NH, H-6 Phe), 7.26 (d, ^3J = 7.6 Hz, 1H, NH), 7.13 (t, $^3J_{H,H} \approx {}^3J_{H,F}$ = 8,8 Hz, 2H, H-3,5 FBz), 7.05 (t, ^3J = 7.8 Hz, 1H, H-5 Phe), 6.44 (s, 1H, CONH*H*), 6.13 – 5.99 (m, 1H, C*H*=CH_2), 5.85 (s, 1H, CON*H*H), 5.45 – 5.35 (m, 1H, CH=CH_{trans}), 5.30 – 5.22 (m, 1H, CH=CH_{cis}), 4.84 – 4.77 (m, 2H, CH_2 allyl), 4.78 – 4.69 (m, 1H, C_αH Phe), 4.57 – 4.53 (m, 2H, CH_2O), 4.50 – 4.41 (m, 1H, C_αH Ser), 3.85 – 3.77 (m, 1H, C_β*H*H Ser), 3.69 – 3.63 (m, 1H, C_βH*H* Ser), 3.26 – 3.18 (m, 1H, C_β*H*H Phe), 3.05 – 2.97 (m, 1H, C_βH*H* Phe).

^{13}C NMR (101 MHz, CD_3CN) δ = 172.33 ($CONH_2$), 171.72 (CO), 167.43 (CO), 166.81 (COO), 165.67 (d, $^1J_{C,F}$ = 250,48 Hz, CF), 141.38, 139.91, 139.30, 136.61, 133.60, 133.16, 131.31, 131.25, 130.82, 130.73, 129.76, 129.63, 129.44, 129.23, 118.26 (CD_3CN, C=CH_2), 116.29 (d, $^2J_{C,F}$ = 22.1 Hz, C-3/5 FBz), 94.66 (CI), 73.03 (CH_2O), 70.70 (C_β Ser), 66.33 (CH_2 allyl), 56.09 (C_α Phe), 54.01 (C_α Ser), 37.18 (C_β Phe).

N-Diphenylacetyl-3-iodo-L-phenylalanyl-*O*-(3-(allyloxycarbonyl)benzyl)-L-serinamide (9e)

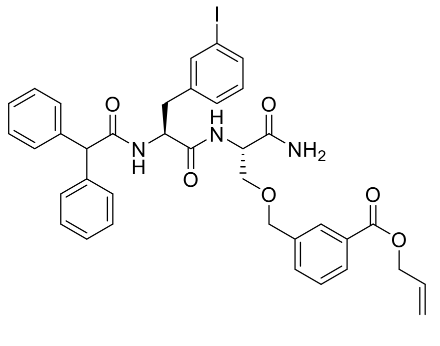

HR-MS ESI (+):

m/z = 768.1538 ([M+Na]$^+$)

M ($C_{37}H_{36}IN_3NaO_6^+$, monoisotopic): 768.1541

The synthesis was accomplished according to GP III. using 0.02 g **8b** (0.03 mmol, 1 eq.), 0.01 g diphenylacetic acid (0.04 mmol, 1.5 eq.), 9 µL DiPEA (0.05 mmol, 1.9 eq.), 0.02 g PyBOP (0.04 mmol, 1.5 eq.) and 10 mL THF. *N*-Diphenylacetyl-3-iodo-L-phenylalanyl-*O*-(3-(allyloxycarbonyl)benzyl)-L-serinamide (**9e**) was obtained as a yellow oil (0.03 g, quantitative yield).

^{1}H NMR (400 MHz, CD_3CN) δ = 7.97 – 7.93 (m, 2H, H-2,4 BnCOO), 7.62 – 7.53 (m, 3H, H-6 BnCOO, aromatic Phe), 7.49 – 7.44 (m, 1H, H-5 BnCOO), 7.30 – 7.07 (m, 12H, aromatic diphenyl, aromatic Phe, NH), 7.03 – 6.95 (m, 2H, H-2 Phe, NH), 6.34 (s, 1H, CONH*H*), 6.14 – 6.01 (m, 1H, C*H*=CH_2), 5.81 (s, 1H, CON*H*H), 5.45 – 5.38 (m, 1H, CH=CH_{trans}), 5.31 – 5.25 (m, 1H, CH=CH_{cis}), 4.90 (s, 1H, CH diphenyl), 4.81 (dt, ^3J = 5.5, ^4J = 1.5 Hz, 2H, CH_2 allyl), 4.70 – 4.61 (m, 1H, C_αH Ser), 4.51 (s, 2H, CH_2O),

4.43 – 4.38 (m, 1H, CαH Phe), 3.78 – 3.72 (m, 1H; Cβ*H*H Ser), 3.59 – 3.54 (m, 1H, CβH*H* Ser), 3.14 – 3.07 (m, 1H, Cβ*H*H Phe), 2.87 – 2.79 (m, 1H, CβH*H* Phe).

^{13}C NMR (101 MHz, CD_3CN) δ = 173.08, 172.19, 171.49, 166.90, 140.99, 140.57, 139.90, 139.18, 136.67, 133.61, 133.30, 131.29, 129.72, 129.68, 129.55, 129.48, 129.43, 129.32, 127.93, 118.26 (CH=*C*H_2, solvent), 101.05, 94.77, 73.01 (CH_2O), 70.67 (Cβ Ser), 66.39 (CH_2 allyl), 58.44 (CH diphenyl), 55.35 (Cα Ser), 53.83 (Cα Phe), 37.10 (Cβ Phe).

Due to overlapping four signals were not identified.

N-(2,4-Difluorobenzoyl)-3-methyl-L-phenylalanyl-O-(3-(allyloxycarbonyl)benzyl)-L-serine nitrile (10a)

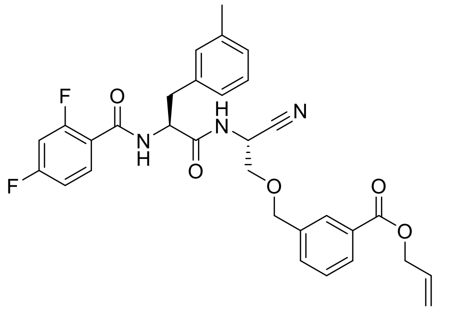

ESI (+):

m/z = 562.27 ($[M+H]^+$)

M ($C_{31}H_{30}F_2N_3O_5^+$, monoisotopic): 562.60

The synthesis was accomplished according to GP V. using 0.04 g **9a** (0.08 mmol, 1 eq.), 0.04 g cyanuric chloride (0.24 mmol, 3 eq.) and 5 mL DMF. The solution was stirred 5 h. *N*-(2,4-Difluorobenzoyl)-3-methyl-L-phenylalanyl-*O*-(3-(allyloxycarbonyl)benzyl)-L-serine nitrile (**10a**) was obtained as a wax-like white solid (38.6 mg, 0.07 mmol, 72%; contained 30% trifluoroacetylated side product).

^{1}H NMR (600 MHz, $CDCl_3$) δ = 8.06 – 7.91 (m, 3H, H-6 FBz, H-2,4 aromatic), 7.51 – 7.45 (m, 1H, H-6 aromatic), 7.42 (t, ^3J = 7.6 Hz, 1H, H-5 aromatic), 7.23 – 7.18 (m, 1H, H-5 aromatic Phe), 7.11 – 7.01 (m, 4H, H-2,4,6 aromatic Phe, NH), 6.98 – 6.92 (m, 1H, H-5 FBz), 6.88 – 6.81 (m, 1H, H-3 FBz), 6.54 (d, ^3J = 8.5 Hz, 1H, NH), 6.11 – 5.99 (m, 1H, C*H*=CH_2), 5.46 – 5.38 (m, 1H, CH=CHH_{trans}), 5.33 – 5.28 (m, 1H, CH=CH_{cis}H), 5.02 – 4.92 (m, 1H, CαH Ser), 4.86 – 4.77 (m, 3H, CH_2 allyl, CαH Phe), 4.56 (s, 2H, CH_2O), 3.71 – 3.52 (m, 2H, CβH_2 Ser), 3.25 – 3.04 (m, 2H, CβH_2 Phe), 2.31 (s, 3H, CH_3).

^{13}C NMR (151 MHz, $CDCl_3$) δ = 170.56 (CO), 166.19 (CF), 166.10 (COO), 164.36 (CO), 162.67 (CF), 162.06 (CF), 160.40 (CF), 139.00, 137.19, 135.85, 132.37, 132.24, 130.60, 130.24, 129.56, 129.10, 128.96, 128.94, 128.73, 128.34, 126.37, 118.76, 118.67, 116.64, 116.20, 112.66 (C-5 FBz), 104.59 (C-3 FBz), 73.15 (CH_2O), 68.94 (Cβ Ser), 65.92 (CH_2 Allyl), 55.41 (Cα Phe), 40.66, 38.17, 21.48 (CH_3).

Multiplicity of C-F signals was not considered.

^{19}F NMR (376 MHz, $CDCl_3$) δ = -102.91 (F aromatic), -108.30 (F aromatic).

N-(4-Fluorobenzoyl)-3-methyl-L-phenylalanyl-O-(3-(allyloxycarbonyl)benzyl)-L-serine nitrile (10b)

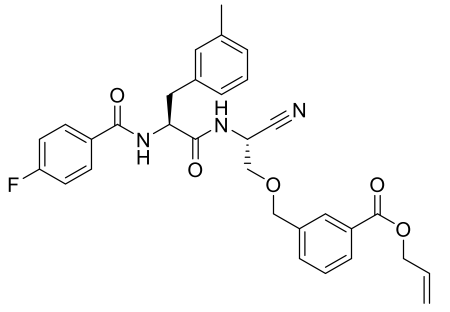

ESI (+):

m/z = 544.14 ($[M+H]^+$)

M ($C_{31}H_{31}FN_3O_5^+$, monoisotopic): 544.22

The synthesis was accomplished according to GP V. using 0.12 g **9b** (0.22 mmol, 1 eq.), 0.08 g cyanuric chloride (0.44 mmol, 2 eq.) and 10 mL DMF. *N*-(4-Fluorobenzoyl)-3-methyl-L-phenylalanyl-*O*-(3-(allyloxycarbonyl)benzyl)-L-serine nitrile (**10b**) was obtained as a yellow solid (0.13 g, quantitative yield; contained trifluoroacetylated side product, amount not quantified).

^{1}H-NMR (400 MHz, DMSO-*d6*) δ = 9.03 (d, ^3J = 7.7 Hz, 1H, NH), 8.67 (d, ^3J = 8.2 Hz, 1H, NH), 8.00 - 7.96 (m, 1H, H-4 BnCOO), 7.94 - 7.90 (m, 1H, H-2 BnCOO), 7.90 - 7.84 (m, 2H, H-2,6 FBz), 7.68 - 7.64 (m, 1H, aromatic H-6 BnCOO), 7.53 (t, ^3J = 7.7 Hz, 1H, H-5 BnCOO), 7.31 - 7.24 (m, 2H, H-3,5 FBz), 7.16 - 7.10 (m, 3H, aromatic Phe), 6.99 - 6.95 (m, 1H, aromatic Phe), 6.08 - 5.98 (m, 1H, C*H*=CH_2), 5.39 (dq, ^2J = 17.3, ^3J = 1.7 Hz, 1H, CH=CHH_{trans}), 5.26 (dq, ^2J = 10.5, ^3J = 1.4 Hz, 1H, CH=CHH_{cis}), 5.10 - 5.03 (m, 1H, C_αH Phe), 4.80 (dt, ^{3} = 5.4, ^4J = 1.5 Hz, 2H, CH_2 allyl), 4.73 - 4.63 (m, 3H, C_αH Ser, CH_2O), 3.77 - 3.67 (m, 2H, $C_\beta H_2$ Ser), 3.07 - 2.89 (m, 2H, C_βH*H* Phe), 2.23 (s, 3H, CH_3).

^{13}C-NMR (101 MHz, DMSO-*d6*) δ = 171.65 (CO), 165.23 (CO), 165.22 (CO), 163.91 (d, $^1J_{C,F}$ = 248.8 Hz, C-3/5 FBz), 138.44, 137.79, 137.02, 132.55, 130.30, 130.13, 130.04, 129.77, 129.67, 128.89, 128.51, 128.27, 127.93, 126.97, 126.22, 117.98 (CN), 117.96, 115.09 (d, $^2J_{C,F}$ = 21.7 Hz), 71.68 (CH_2O), 68.35 (C_β Ser), 65.12 (CH_2 Allyl), 54.76 (C_α Ser), 40.54 (C_α Phe), 36.84 (C_β Phe), 20.98 (CH_3).

^{19}F NMR (376 MHz, DMSO-*d6*) δ = - 109.19 (F aromatic).

N-Diphenylacetyl-3-methyl-L-phenylalanyl-O-(3-(allyloxycarbonyl)benzyl)-L-serine nitrile (10c)

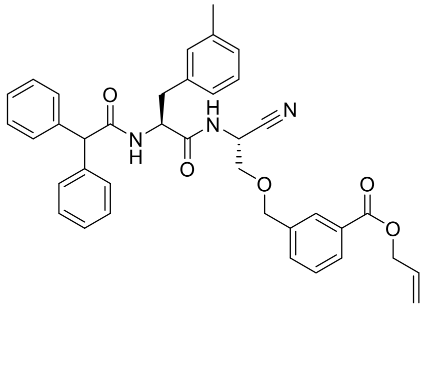

ESI (+):

m/z = 616.25 ($[M+H]^+$)

M ($C_{38}H_{38}N_3O_5^+$, monoisotopic): 616.28

The synthesis was accomplished according to GP V. using 0.07 g **9c** (0.12 mmol, 1 eq.), 0.04 g cyanuric chloride (0.24 mmol, 2 eq.) and 10 mL DMF. *N*-Diphenylacetyl-3-methyl-L-phenylalanyl-*O*-(3-(allyloxycarbonyl)benzyl)-L-serine nitrile (**10c**) was obtained as a yellow solid (0.08 g, quantitative yield).

^{1}H NMR (400 MHz, CD_3CN) δ = 8.01 – 7.95 (m, 2H, H-2,4 BnCOO), 7.60 (d, ^3J = 7.6 Hz, 1H, H-6 BnCOO), 7.49 (t, ^3J = 7.6 Hz, 1H, H-5 BnCOO), 7.33 (d, ^3J = 7.8 Hz, 1H, NH), 7.29 – 7.08 (m, 11H, 1 aromatic Phe, aromatic diphenyl), 7.02 (d, ^3J = 7.6 Hz, 1H, aromatic Phe), 6.97 – 6.88 (m, 3H, 2 aromatic Phe, NH), 6.12 – 6.01 (m, 1H, C*H*=CH_2), 5.44 – 5.38 (m, 1H, CH=CH_{trans}), 5.29 – 5.25 (m, 1H, CH=CH_{cis}), 4.95 – 4.89 (m, 2H, CH diphenyl, C_αH Ser), 4.80 (dt, ^3J = 5.5, ^4J = 1.5 Hz, 2H, CH_2 allyl), 4.66 – 4.61 (m, 1H, C_αH Phe), 4.60 (s, 2H, CH_2O), 3.74 – 3.69 (m, 1H, C_β*H*H Ser), 3.66 – 3.60 (m, 1H, C_βH*H* Ser), 3.09 – 3.02 (m, 1H, C_β*H*H Phe), 2.89 – 2.81 (m, 1H, C_βH*H* Phe), 2.23 (s, 3H, CH_3).

^{13}C NMR (101 MHz, CD_3CN) δ = 172.55 (CO), 171.87 (CO), 166.74 (COO), 140.77, 139.33, 138.94, 137.66, 133.60, 133.43, 131.39, 130.95, 129.78, 129.73, 129.69, 129.58, 129.48, 129.41, 129.40, 129.27, 128.35, 127.90, 127.27, 118.26, 73.29 (CH_2O), 69.62 (C_β Ser), 66.33 (CH_2 allyl), 58.37 (CH diphenyl), 55.20 (C_α Phe), 41.71 (C_α Ser), 37.89 (C_β Phe), 21.41 (CH_3).

Due to overlapping three signals were not identified.

***N-(*4-Fluorobenzoyl)-3-iodo-L-phenylalanyl-*O*-(3-(allyloxycarbonyl)benzyl)-L-serinamide (10d)**

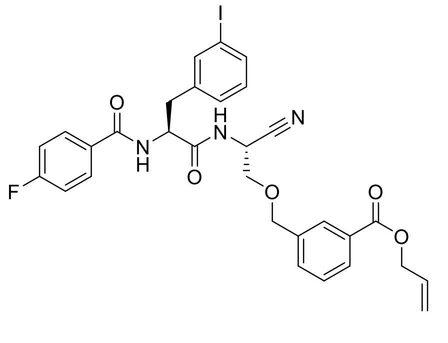

HR-MS ESI (+):

m/z = 656.1049 ($[M+H]^+$)

M ($C_{30}H_{28}FIN_3O_5^+$, monoisotopic): 656.1052

The synthesis was accomplished according to GP V. using 0.04 g **9d** (0.06 mmol, 1 eq.), 0.02 g cyanuric chloride (0.11 mmol, 1.9 eq.) and 10 mL DMF. *N-(*4-Fluorobenzoyl)-3-iodo-L-phenylalanyl-*O*-(3-(allyloxycarbonyl)benzyl)-L-serinamide (**10d**) was obtained as a yellowish solid (0.03 g, 0.05 mmol, 92%).

^{1}H NMR (400 MHz, CD_3CN) δ = 8.00 (s, 1H, H-2 BnCOO), 7.98 – 7.93 (m, 1H, H-4 BnCOO), 7.78 – 7.71 (m, 2H, H-2,6 FBz), 7.70 – 7.67 (m, 1H, H-2 Phe), 7.62 – 7.55 (m, 2H, H-6 BnCOO, H-4 Phe), 7.51 – 7.44 (m, 2H, NH, H-5 BnCOO), 7.29 (d, ^3J = 7.4 Hz, 2H, NH, H-6 Phe), 7.15 (t, $^3J_{H,H} \approx {}^3J_{H,F}$ = 8.9 Hz, 2H, H-3,5 FBz), 7.05 (t, ^3J = 7.8 Hz, 1H, H-5 Phe), 6.13 – 6.01 (m, 1H, C*H*=CH_2), 5.46 – 5.37 (m, 1H, CH=CH_{trans}), 5.30 – 5.25 (m, 1H, CH=CH_{cis}), 5.02 – 4.94 (m, 1H, C_αH Ser), 4.83 – 4.79 (m, 2H, CH_2 allyl),

4.78 – 4.71 (m, 1H, CαH Phe), 4.64 (s, 2H, CH_2O), 3.80 – 3.69 (m, 2H, $C_βH_2$ Ser), 3.25 – 3.18 (m, 1H, $C_β$H*H* Phe), 3.03 – 2.95 (m, 1H, $C_β$*H*H Phe).

^{13}C NMR (101 MHz, CD_3CN) δ = 171.81 (CO), 167.03 (CO), 166.73 (COO), 165.66 (d, $^1J_{C,F}$ = 249.5 Hz, CF), 141.14, 139.37, 139.30, 136.64, 133.60, 133.35, 131.37, 131.31, 131.18 (d, $^4J_{C,F}$ = 3.1 Hz, C-1 FBz), 130.81 (d, 3JC,F = 9.2 Hz, C-2/6 FBz), 129.78, 129.75, 129.70, 129.42, 118.26 (solvent, CH=*C*H_2), 116.26 (d, $^2J_{C,F}$ = 22.1 Hz, C-3/5 FBz), 94.65 (CI), 73.29 (CH_2O), 69.66 ($C_β$ Ser), 66.33 (CH_2 allyl), 55.56 ($C_α$ Phe), 41.77 ($C_α$ Ser), 37.28 ($C_β$ Phe).

Due to overlapping one signal was not identified.

***N*-Diphenylacetyl-3-iodo-L-phenylalanyl-*O*-(3-(allyloxycarbonyl)benzyl)-L-serine nitrile (10e)**

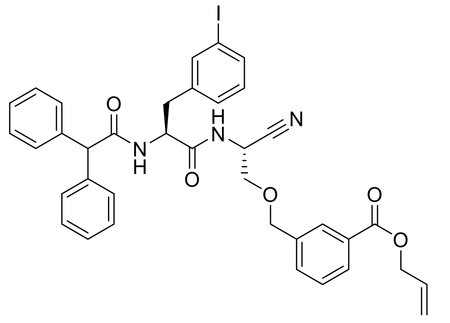

HR-MS ESI (+):

m/z = 728.1614 ($[M+H]^+$)

M ($C_{37}H_{35}IN_3O_5^+$, monoisotopic): 728.1616

The synthesis was accomplished according to GP V. using 0.02 g **9e** (0.03 mmol, 1 eq.), 0.01 g cyanuric chloride (0.05 mmol, 2 eq.) and 10 mL DMF. *N*-Diphenylacetyl-3-iodo-L-phenylalanyl-*O*-(3-(allyloxycarbonyl)benzyl)-L-serine nitrile (**10e**) was obtained as a yellowish solid (0.02 g, 0.02 mmol, 82%).

^{1}H NMR (400 MHz, CD_3CN) δ = 8.01 – 7.95 (m, 2H, H-2,4 BnCOO), 7.63 – 7.56 (m, 3H, H-2,4 Phe, H-6 BnCOO), 7.53 – 7.47 (m, 1H, H-5 BnCOO), 7.34 (d, ^3J = 7.9 Hz, 1H, NH), 7.30 – 7.08 (m, 11H, H-6 Phe, aromatic diphenyl), 7.03 – 6.93 (m, 2H, NH, H-5 Phe), 6.15 – 6.00 (m, 1H, C*H*=CH_2), 5.45 – 5.38 (m, 1H, CH=CH_{trans}), 5.30 – 5.25 (m, 1H, CH=CH_{cis}), 4.97 – 4.87 (m, 2H, CαH Phe, CH diphenyl), 4.83 – 4.78 (m, 2H, CH_2 allyl), 4.70 – 4.61 (m, 1H, CαH Ser), 4.60 (s, 2H, CH_2O), 3.74 – 3.68 (m, 1H, $C_β$*H*H Ser), 3.66 – 3.60 (m, 1H, $C_β$H*H* Ser), 3.12 – 3.03 (m, 1H, $C_β$*H*H Phe), 2.87 – 2.77 (m, 1H, $C_β$H*H* Phe).

^{13}C NMR (101 MHz, CD_3CN) δ = 172.66 (CO), 171.61 (CO), 166.77 (CO), 140.68, 139.34, 139.18, 136.74, 133.60, 133.43, 131.39, 131.30, 129.79, 129.77, 129.73, 129.69, 129.55, 129.47, 129.42, 127.93, 118.27 (CH_3CN, CH=*C*H_2) 94.76 (CI), 73.29 (CH_2O), 69.62 (CH_2 allyl), 66.36 ($C_β$ Ser), 58.35 ($C_α$ Phe), 54.84 (CH diphenyl), 41.74 ($C_α$ Ser), 37.30 ($C_β$ Phe).

Due to overlapping five signals were not identified.

***N*-(4-Fluorobenzoyl)-3-methyl-L-phenylalanyl-*O*-(3-carboxybenzyl)serine nitrile (1a)**

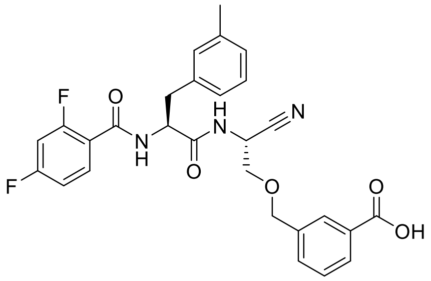

HR-MS ESI (+):

m/z = 526.1747 ($[M+Na]^+$)

M ($C_{28}H_{26}FN_3NaO_5^+$, monoisotopic): 526.1748

10a (0.04 g, 0.07 mmol, 1 eq.) was dissolved in dry CH_2Cl_2 (5 mL) under argon atmosphere. After adding phenylsilane (121 µL, 0.99 mmol, 15 eq.) and $Pd(PPh_3)_4$ (15 mg, 0.01 mmol, 0.2 eq.) the solution was stirred 2 h. Sodium diethyldithiocarbamate (0.5% in H_2O, 15 mL) was added and the solution stirred 10 min before diluting the solution with H_2O/ethyl acetate (1:1, 10 mL). The solution was extracted with ethyl acetate (3 × 10 mL) and the combined organic layers washed with brine (2 × 15 mL) and dried over Na_2SO_4. The solvent was evaporated. The crude product was purified via semipreparative HPLC to yield *N*-(4-fluorobenzoyl)-3-methyl-L-phenylalanyl-*O*-(3-carboxybenzyl)serine nitrile (**1a**) as a white solid (4.0 mg, 0.01 mmol, 15%).

^{1}H NMR (400 MHz, $CDCl_3$) δ = 8.08 – 7.97 (m, 2H, H-4 aromatic BnCOO, H-6 FBz), 7.93 (s, 1H, H-2 aromatic BnCOO), 7.49 (d, ^3J = 7.7 Hz, 1H, H-6 aromatic BnCOO), 7.42 (t, ^3J = 7.6 Hz, 1H, H-5 aromatic BnCOO), 7.38 – 7.31 (m, 1H, NH), 7.20 (t, ^3J = 7.5 Hz, 1H, H-5 aromatic Phe), 7.09 – 7.02 (m, 3H, H-2,4,6 aromatic Phe), 6.99 – 6.93 (m, 1H, H-5 FBz), 6.89 – 6.81 (m, 1H, H-2 FBz), 6.78 (d, ^3J = 8.4 Hz, 1H, NH), 5.05 – 4.99 (m, 1H, $C_αH$ Ser), 4.93 – 4.86 (m, 1H, $C_αH$ Phe), 4.57 (q, ^3J = 12.2 Hz, 2H, CH_2O), 3.70 (dd, ^3J = 9.8, ^2J = 3.8 Hz, 1H, $C_βH\mathit{H}$ Ser), 3.59 (dd, ^3J = 9.8, ^2J = 4.1 Hz, 1H, $C_β\mathit{H}H$ Ser), 3.21 (dd, ^3J = 13.6, ^2J = 6.3 Hz, 1H, $C_βH\mathit{H}$ Phe), 3.08 (dd, ^3J = 13.7, ^2J = 7.8 Hz, 1H, $C_β\mathit{H}H$ Phe), 2.30 (s, 3H, CH_3).

The carboxyl proton was not detectable in the chosen solvent.

^{13}C NMR (101 MHz, $CDCl_3$) δ = 170.60, 168.91, 168.69, 162.72, 162.69, 138.98, 137.32, 135.74, 132.74, 132.22, 130.26, 129.98, 129.87, 129.11, 129.07, 128.98, 128.32, 126.40, 116.63, 112.50, 104.65, 72.94 (CH_2O), 68.93 ($C_β$ Ser), 55.35 ($C_α$ Phe), 40.69 ($C_α$ Ser), 38.52 ($C_β$ Phe), 21.46 (CH_3).

Due to overlapping, one signal was not identified. Multiplicity of C-F signal was not considered.

N-(4-Fluorobenzoyl)-3-methyl-L-phenylalanyl-O-(3-carboxybenzyl)serine nitrile (1b)

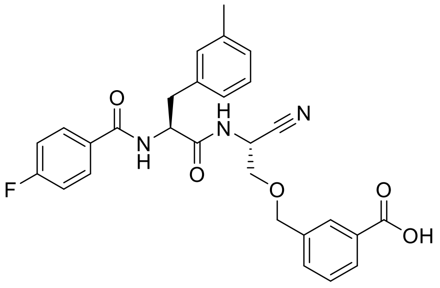

HR-MS ESI (+):

m/z = 504.1927 ([M+H]$^+$)

M ($C_{28}H_{27}FN_3O_5^+$, monoisotopic): 504.1929

The synthesis was accomplished according to GP VI. using 0.11 g **10b** (0.20 mmol), 174 µL morpholine (2.00 mmol), 0.05 g $Pd(PPh_3)_4$ (0.04 mmol) and 15 mL CH_2Cl_2. *N*-(4-Fluorobenzoyl)-3-methyl-L-phenylalanyl-*O*-(3-carboxybenzyl)serine nitrile (**1b**) was obtained as a white solid (55.5 mg, 0.11 mmol, 55%).

^{1}H NMR (400 MHz, DMSO-*d$_6$*) δ = 9.03 (d, ^3J = 7.7 Hz, 1H, NH), 8.68 (d, ^3J = 8.2 Hz, 1H, NH), 7.96 - 7.93 (m, 1H, aromatic), 7.90 - 7.84 (m, 3H, aromat), 7.64 - 7.60 (m, 1H, aromatic), 7.49 (t, ^2J = 7.8 Hz, 1H, aromatic), 7.28 (t, $^3J_{H,H}$ ≈ $^3J_{H,F}$ = 8,8 Hz, 2H, aromatic), 7.17 - 7.10 (m, 3H, aromatic), 7.00 - 6.94 (m, 1H, aromatic), 5.10 - 5.04 (m, 1H, $C_α$ H Phe), 4.73 - 4.65 (m, 3H, $C_α$H Ser, O-CH_2-Ar), 3.75 - 3.67 (m, 2H, $C_βH_2$ Ser), 3.07 - 2.91 (m, 2H, $C_βH_2$ Phe), 2.23 (s, 3H, CH_3).

The carboxyl proton was not detectable in the chosen solvent.

^{13}C-NMR (101 MHz, DMSO-*d$_6$*) δ = 171.65, 167.15, 165.25, 163.91 (d, $^1J_{C,F}$ = 248.7 Hz, CF), 138.13, 137.80, 137.03, 132.04, 130.84, 130.30 (d, $^4J_{C,F}$ = 2.8 Hz, C-1 FBz) 130.10 (d, $^3J_{C,F}$ = 9.1 Hz, C-2/6 FBz), 129.78, 128.64, 128.61, 128.47, 127.94, 126.97, 126.23, 118.00 (CN), 115.09 (d, $^2J_{C,F}$ = 21.8 Hz, C-3/5 FBz), 71.84 (O-CH_2-Ar), 68.34 ($C_βH_2$ Ser), 54.77 ($C_α$H Ser), 40.55 ($C_α$H Phe), 36.84 ($C_βH_2$ Phe), 20.98 (CH_3).

^{19}F NMR (376 MHz, DMSO-*d$_6$*) δ= - 109.19 (F aromatic).

N-Diphenylacetyl-3-methyl-L-phenylalanyl-O-(3-carboxybenzyl)-L-serine nitrile (1c)

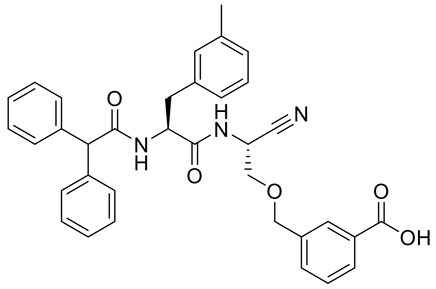

HR-MS ESI (+):

m/z = 576.2493 ([M+H]$^+$)

M ($C_{35}H_{34}N_3O_5^+$, monoisotopic): 576.2493

The synthesis was accomplished according to GP VI. using 0.04 g **10c** (0.07 mmol), 60 µL morpholine (0.68 mmol), 0.01 g $Pd(PPh_3)_4$ (0.01 mmol) and 10 mL CH_2Cl_2. *N*-

Diphenylacetyl-3-methyl-L-phenylalanyl-*O*-(3-carboxybenzyl)-L-serine nitrile (**1c**) was obtained as a white solid (9.3 mg, 0.02 mmol, 24%).

^{1}H NMR (400 MHz, CD_3CN) δ = 7.99 – 7.93 (m, 2H, H-2,4 BnCOO), 7.60 (d, ^3J = 7.8 Hz, 1H, H-6 BnCOO), 7.49 (t, ^3J = 7.7 Hz, 1H, H-5 BnCOO), 7.31 – 7.18 (m, 9H, H aromatic diphenylacetyl, NH), 7.18 – 7.08 (m, 3H, H aromatic diphenylacetyl, H aromatic Phe), 7.03 (d, ^3J = 7.7 Hz, 1H, H aromatic Phe), 6.97 – 6.86 (m, 3H, H aromatic Phe, NH), 4.95 – 4.88 (m, 2H, CH diphenylacetyl, $C_αH$ Ser), 4.70 – 4.61 (m, 1H, $C_αH$ Phe), 4.60 (s, 2H, CH_2O), 3.71 (dd, ^3J = 10.0, ^2J = 4.7 Hz, 1H, $C_β$*H*H Ser), 3.63 (dd, ^3J = 10.0, ^2J = 5.0 Hz, 1H, $C_β$H*H* Ser), 3.06 (dd, ^{3}J = 13.9, ^2J = 5.5 Hz, 1H, $C_β$*H*H Phe), 2.85 (dd, ^3J = 13.9, ^2J = 8.5 Hz, 1H, $C_β$H*H* Phe), 2.24 (s, 3H, CH_3).

The carboxyl proton was not detectable in the chosen solvent.

^{13}C NMR (101 MHz, CD_3CN) δ = 172.57 (C=O), 171.83 (C=O), 167.65 (COO), 140.74, 139.25, 138.95, 137.63, 133.42, 131.18, 130.96, 130.01, 129.74, 129.71, 129.68, 129.59, 129.42, 129.41, 129.28, 128.36, 127.92, 127.28 (CN), 73.30 (CH_2O), 69.60 ($C_β$ Phe), 58.39 (CH diphenylacetyl), 55.17 ($C_α$ Phe), 41.71 ($C_α$ Ser), 37.91 ($C_β$ Phe), 21.41 (CH_3).

Due to overlapping three signals were not identified.

_N-(_4-Fluorobenzoyl)-3-iodo-L-phenylalanyl-_O_-(3-carboxybenzyl)-L-serine nitrile (1d)

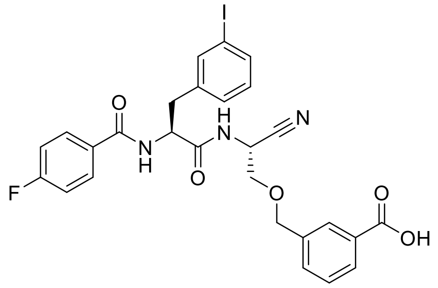

HR-MS ESI (+):

m/z = 638.0558 ([M+Na]$^+$)

M ($C_{27}H_{23}FIN_3NaO_5^+$, monoisotopic): 638.0558

The synthesis was accomplished according to GP VI. using 0.05 g **10d** (0.07 mmol, 1 eq.), 60 µL morpholine (0.68 mmol, 10 Äq.), 0.02 g $Pd(PPh_3)_4$ (0.02 mmol, 0.1 Äq.) and 10 mL CH_2Cl_2. *N*-(4-Fluorobenzoyl)-3-iodo-L-phenylalanyl-*O*-(3-carboxybenzyl)-L-serine nitrile (**1d**) was obtained as a white solid (11.4 mg, 0.02 mmol, 36%).

^{1}H NMR (400 MHz, CD_3CN) δ = 7.98 (s, 1H, H-2 BnCOO), 7.93 (d, ^3J = 7.7 Hz, 1H, H-4 BnCOO), 7.76 (dd, $^3J_{H,H}$ = 8.8, $^4J_{H,F}$ = 5.4 Hz, 2H, H-2,6 FBz), 7.68 (s, 1H, H-2 Phe), 7.60 – 7.55 (m, 2H, H-6 BnCOO, H-4 Phe), 7.53 (d, ^3J = 7.4 Hz, 1H, NH), 7.45 (t, ^3J = 7.7 Hz, 1H, H-5 BnCOO), 7.35 – 7.26 (m, 2H, NH, H-6 Phe), 7.15 (t, $^3J_{H,H} \approx {}^3J_{H,F}$ = 8.8 Hz, 1H), H-3,5 FBz), 7.05 (t, ^3J = 7.8 Hz, 1H, H-5 Phe), 5.02 – 4.94 (m, 1H, $C_αH$ Phe), 4.82 – 4.72 (m, 1H, $C_αH$ Ser), 4.63 (s, 2H, CH_2O), 3.79 – 3.68 (m, 2H, $C_βH_2$ Ser), 3.26 – 3.18 (m, 1H, $C_β$H*H* Phe), 3.05 – 2.96 (m, 1H, $C_β$*H*H Phe).

The carboxyl proton was not detectable in the chosen solvent.

^{13}C NMR (101 MHz, CD_3CN) δ = 172.89, 168.81, 141.15, 139.30, 136.64, 133.28, 131.30, 130.88, 130.79, 129.96, 129.81, 129.66, 125.87, 121.61 (CN), 116.27 (d, $^{2}J_{C,F}$ = 22.2 Hz, C-3/5 FBz) 105.88 (CI), 73.30 (CH_2O), 69.64 ($C_β$ Ser), 55.57 ($C_α$ Phe), 41.77 ($C_α$ Ser), 37.31 ($C_β$ Phe).

Due to low amounts of substance, four signals were not detectable.

***N*-Diphenylacetyl-3-iodo-L-phenylalanyl-*O*-(3-carboxybenzyl)-L-serine nitrile (1e)**

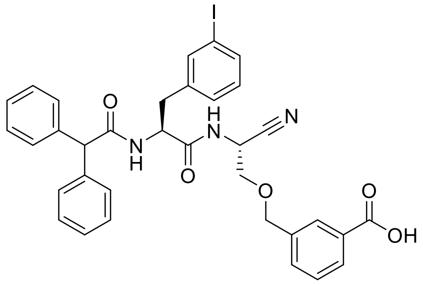

HR-MS ESI (+):

m/z = 710.1123 ($[M+Na]^+$)

M ($C_{34}H_{30}IN_3NaO_5^+$, monoisotopic): 710.1122

The synthesis was accomplished according to GP VI. using 0.02 g **10e** (0.02 mmol, 1 eq.), 19 µL morpholine (0.22 mmol, 10 eq.), 0.005 g $Pd(PPh_3)_4$ (0.004 mmol, 0.02 eq.) and 10 mL CH_2Cl_2. *N*-Diphenylacetyl-3-iodo-L-phenylalanyl-*O*-(3-carboxybenzyl)-L-serine nitrile (**1e**) was obtained as a white solid (4.4 mg, 0.01 mmol, 30%).

^{1}H NMR (400 MHz, CD_3CN) δ = 7.98 – 7.92 (m, 2H, H-2,4 BnCOO), 7.61 – 7.56 (m, 3H, H-6 BnCOO, aromatic Phe), 7.48 (t, ^{3}J = 7.6 Hz, 1H, H-5 BnCOO), 7.35 (d, ^{3}J = 7.9 Hz, 1H, NH Ser), 7.31 – 7.08 (m, 11H, aromatic Phe, diphenyl), 7.03 – 6.93 (m, 2H, H-2 Phe, NH Phe), 4.95 – 4.88 (m, 2H, CH diphenyl, $C_αH$ Ser), 4.71 – 4.63 (m, 1H, $C_αH$ Phe), 4.59 (s, 2H, CH_2O), 3.74 – 3.67 (m, 1H, $C_β\mathit{H}H$ Ser), 3.66 – 3.59 (m, 1H, $C_βH\mathit{H}$ Ser), 3.11 – 3.04 (m, 1H, $C_β\mathit{H}H$ Phe), 2.87 – 2.79 (m, 1H, $C_βH\mathit{H}$ Phe).

The carboxyl proton was not detectable in the chosen solvent.

^{13}C NMR (101 MHz, CD_3CN) δ = 172.69, 171.60, 167.77, 140.66, 140.63, 139.22, 139.16, 136.74, 133.39, 131.29, 130.01, 129.80, 129.74, 129.71, 129.68, 129.56, 129.47, 129.42, 127.93, 94.75, 73.31 (CH_2O), 69.59 ($C_β$ Ser), 58.35 (CH diphenyl), 54.83 ($C_α$ Phe), 41.74 ($C_α$ Ser), 37.35 ($C_β$ Phe).

Due to overlapping, four signals were not detectable.

2 Synthesis of Dipeptide Alkyne 18 (Scheme 2)

(R)-2-(*tert*-Butyloxycarbonylamino)-3-(3-(allyloxycarbonyl)phenyl)methoxypropan-1-ol (11)

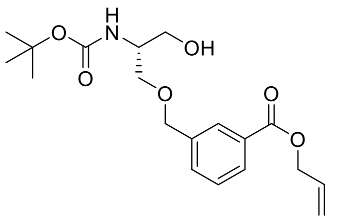

ESI (+):

m/z = 388.19 ([M+Na]$^+$)

M ($C_{19}H_{27}NO_6Na^+$, monoisotopic): 388.17

4 (0.50 g, 1.31 mmol, 1 eq.), PyBOP (0.76 g, 1.45 mmol, 1.1 eq.) and DiPEA (275.4 µL, 1.58 mmol, 1.2 eq.) were dissolved in dry THF (25 mL). After 5 min $NaBH_4$ (0.05 g, 1.32 mmol, 1 eq.) was added and the solution stirred. The solvent was removed *in vacuo* and the residue was dissolved in ethyl acetate (25 mL) and HCl (5%, 25 mL). The organic layer was washed with HCl (5%, 3 × 10 mL), saturated $NaHCO_3$ (3 × 20 mL) and brine (25 mL) and dried over Na_2SO_4. The solvent was evaporated. The crude product was purified via preparative column chromatography (petroleum ether/ethyl acetate (3:2)) to yield *(R)*-2-(*tert*-butyloxycarbonylamino)-3-(3-(allyloxy-carbonyl)phenyl)methoxy-propan-1-ol (**11**) as a yellow oil (0.29 g, 0.75 mmol, 60%).**^{1}H NMR** (400 MHz, $CDCl_3$) δ = 8.03 – 8.01 (m, 1H, H-2 aromatic), 8.01 – 7.98 (m, 1H, H-4 aromatic), 7.53 (m, 1H, H-6 aromatic), 7.47 – 7.42 (m, 1H, H-5 aromatic), 6.05 (m, 1H, C*H*=CH_2), 5.45 – 5.38 (m, 1H, CH=CHH_{trans}), 5.33 – 5.27 (m, 1H, CH=CH_{cis}H), 5.13 (broad s, 1H, N_αH), 4.85 – 4.82 (m, 2H, CH_2 allyl), 4.61 – 4.53 (m, 2H, Ar-CH_2O), 3.85 – 3.78 (m, 2H, C_αH, C_β*H*H), 3.75 – 3.59 (m, 3H, C_βH*H,* CH_2OH), 1.44 (s, 9H, $(CH_3)_3$).

The hydroxyl proton was not detectable in the chosen solvent.

^{13}C NMR (101 MHz, $CDCl_3$) δ = 166.19 (CO), 156.22 (CO), 138.26, 132.31 (C_6 aromatic, *C*=CH_2), 130.60, 129.33, 128.94, 128.84, 118.56 (C=*C*H_2), 79.93, 73.12 (Ar-CH_2), 70.96 (CH_2-OH), 65.84 (CH_2 allyl), 64.05 (C_β), 51.77 (C_α), 28.52 ($(CH_3)_3$).

2-(*tert*-Butyloxycarbonylamino)-3-(3-(allyloxycarbonyl)phenyl)methoxypropan-1-al (12)

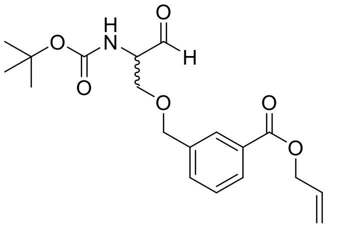

ESI (+):

m/z = 264.12 ([M-Isobutylene-CO_2+H]$^+$)

M ($C_{19}H_{26}NO_6^+$, monoisotopic): 364.18

11 (0.27 g, 0.74 mmol, 1 eq.) and Dess-Martin periodinane (0.62 g, 1.47 mmol, 2 eq.) were dissolved in CH_2Cl_2 (10 mL) and the solution was stirred 4 h. Sodium thiosulfate (10% in sat. $NaHCO_3$, 20 mL) was added and the solution stirred another 15 min. The solution was extracted with CH_2Cl_2 (3 × 10 mL) and the organic layers dried over Na_2SO_4. The solvent was removed *in vacuo* to yield 2-(*tert*-butyloxycarbonylamino)-3-(3-(allyloxycarbonyl)phenyl)-methoxypropan-1-al (**12**) as a yellow oil (0.25 g, quantitative yield).

^{1}H NMR (400 MHz, $CDCl_3$) δ = 9.65 (s, 1H, CHO), 8.01 (dt, ^3J = 7.5, ^4J = 1.5 Hz, 1H, H-4 aromatic), 7.96 (s, 1H, H-2 aromatic), 7.52 – 7.47 (m, 1H, H-6 aromatic), 7.44 (t, ^3J = 7.6 Hz, 1H, H-5 aromatic), 6.10 – 5.98 (m, 1H, C*H*=CH_2), 5.45 – 5.36 (m, 2H, CH=CH_{trans}, NH), 5.33 – 5.27 (m, 1H, CH=CH_{cis}H), 4.85 – 4.81 (m, 2H, CH_2 allyl), 4.60 – 4.51 (m, 2H, Ar-CH_2O), 4.34 (s, 1H, C_αH), 4.05 – 3.98 (m, 1H, C_β*H*H), 3.77 – 3.70 (m, 1H, C_βH*H*), 1.46 (s, 9H, $(CH_3)_3$).

^{13}C NMR (101 MHz, $CDCl_3$) δ = 199.09 (CHO), 166.13 (COO allyl), 155.73 (OOCNH), 137.88, 132.33 (*C*=CH_2), 132.30 (C_6 aromatic), 130.59, 129.42 (C_4 aromatic), 128.95 (C_2 aromatic), 128.85 (C_5 aromatic), 118.55 (C=*C*H_2),80.48 (*C*$(CH_3)_3$), 73.24 (Ar-CH_2O), 68.29 (C_β), 65.84 (CH_2 allyl), 60.18 (C_α), 28.44 ($(CH_3)_3$).

***N*-(*tert*-Butyloxycarbonyl)-*O*-(3-(methyloxycarbonyl)benzyl)serine alkyne (13)**

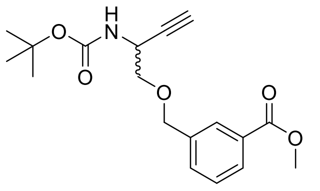

ESI (+):

m/z = 356.14 ([M+Na]$^+$)

M ($C_{18}H_{23}NO_5Na^+$, monoisotopic): 356.15

Dimethyl-(1-diazo-2-oxopropyl)phosphonate (330 µL, 1.38 mmol, 2 eq.) was dissolved in dry MeOH (10 mL). The solution was cooled to 0°C and K_2CO_3 (0.19 g, 1.38 mmol, 2 eq.) was added. The suspension was stirred 2 h at 0°C. Then **12** (0.25 g, 0.75 mmol, 1 eq.) was dissolved in dry MeOH (5 mL) and added dropwise over 20 min. The solution was stirred over night. Then H_2O/ethyl acetate (1:1, 20 mL) was added and the solution extracted with ether (3 × 10 mL). The combined organic layers were washed with brine (20 mL) and dried over Na_2SO_4. The solvent was evaporated. The crude product was purified *via* preparative column chromatography (petroleum ether/ethyl acetate (9:1) to petroleum ether (4:1)) to yield *N*-(*tert*-butyloxycarbonyl)-*O*-(3-(methyloxycarbonyl)-benzyl)serine alkyne (**13**) as a reddish oil (0.04 g, 0.13 mmol, 18% over two steps).

^{1}H NMR (400 MHz, $CDCl_3$) δ = 8.01 – 7.99 (m, 1H, H-2 aromatic), 7.96 (dt, ^3J = 7.7, ^4J = 1.4 Hz, 1H, H-4 aromatic), 7.56 (d, ^3J = 7.7 Hz, 1H, H-6 aromatic), 7.42 (t, ^3J = 7.7 Hz, 1H, H-5 aromatic), 4.95 (broad s, 1H, NH), 4.68 – 4.58 (m, 3H, Ar-CH_2O, C_αH), 3.91 (s, 3H, CH_3), 3.60 (d, ^3J = 4.8 Hz, 2H, $C_\beta H_2$), 2.29 (d, ^4J = 2.4 Hz, 1H, C≡CH), 1.43 (s, 9H, $(CH_3)_3$).

^{13}**C NMR** (101 MHz, $CDCl_3$) δ = 167.07 (*C*$OOCH_3$), 154.97 (OOCNH), 138.31, 132.29 (C_6 aromatic), 130.51, 129.19, 128.90 (C_4 aromatic), 128.73 ($C_{2,5}$ aromatic), 81.60, 80.35, 72.95 (Ar-CH_2O), 72.19 ($C_β$), 71.65, 52.30 (CH_3), 42.91 ($C_α$), 28.48 (C≡*C*H, $(CH_3)_3$).

***O*-(3-(Methyloxycarbonyl)benzyl)serine alkyne (14)**

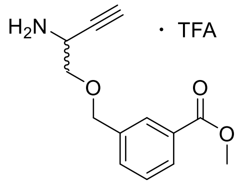

ESI (+):

m/z = 265.09 ($[M+Na]^+$)

M ($C_{13}H_{15}NO_3Na^+$, monoisotopic): 256.11

The synthesis was accomplished according to GP II. using 0.04 g **13** (0.13 mmol) and 10 mL TFA/CH_2Cl_2 (1:1). *O*-(3-(Methyloxycarbonyl)-benzyl)serine alkyne (**14**) was obtained as yellow oil (0.05 g, quantitative yield).

^{1}**H NMR** (400 MHz, DMSO-*d*$_6$) δ = 8.49 (s, 3H, NH_3^+), 7.99 (s, 1H, H-2 aromatic), 7.91 (d, ^3J = 7.8 Hz, 1H, H-4 aromatic), 7.68 (d, ^3J = 7.7 Hz, 1H, H-6 aromatic), 7.54 (t, ^3J = 7.7 Hz, 1H, H-5 aromatic), 4.67 (q, ^3J = 12.3 Hz, 2H, CH_2O), 4.41 (s, 1H, $C_αH$), 3.86 (s, 3H, CH_3), 3.75 (d, ^4J = 2.4 Hz, 1H, HC≡C), 3.74 – 3.53 (m, 2H, $C_βH$).

^{13}**C NMR** (101 MHz, DMSO-*d*$_6$) δ = 166.12 (COO), 138.22, 132.58 (C_6 aromatic), 129.71, 128.83 (C_5 aromatic), 128.51 (C_4 aromatic), 128.40 (C_2 aromatic), 78.71 (*C*≡CH), 77.44 (C≡*C*H), 71.75 (CH_2O), 69.34 ($C_β$), 52.18 (CH_3), 41.54 ($C_α$).

***N*-(*tert*-Butyloxycarbonyl)-3-methyl-L-phenylalanyl-*O*-(3-(methoxycarbonyl)-benzyl)serine alkyne (15)**

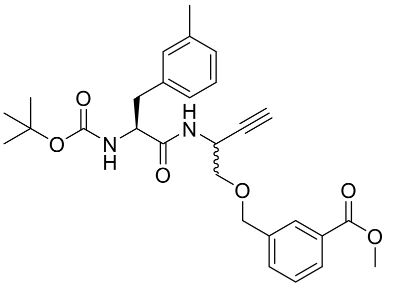

ESI (+):

m/z = 495.25 ($[M+H]^+$)

M ($C_{28}H_{35}N_2O_6^+$, monoisotopic): 495.60

The synthesis was accomplished according to GP III. using 0.05 g **14** (0.13 mmol, 1 eq.), 0.05 g Boc-(3-Me)Phe-OH (0.19 mmol, 1.5 eq.), 90 µL DiPEA (0.52 mmol, 4 eq.), 0.05 g PyBOP (0,19 mmol, 1.5 eq.) and 10 mL THF. The crude product was purified *via* preparative column chromatography (petroleum ether/ethyl acetate (3:1)) to yield *N*-(*tert*-butyloxycarbonyl)-3-methyl-L-phenylalanyl-*O*-(3-(methoxycarbonyl)benzyl)serine alkyne (**15**) as a yellow, wax-like solid (0.04 g, 0.08 mmol, 63%, contained 43% undesired diastereomer).

^{1}H NMR (400 MHz, $CDCl_3$) δ = 8.00 – 7.94 (m, 2H, H-2,4 aromatic), 7.54 – 7.48 (m, 1H, H-6 aromatic), 7.45 – 7.40 (m, 1H, H-5 aromatic), 7.19 – 7.13 (m, 1H, H-5 aromatic Phe), 7.05 – 6.96 (m, 3H, H-2,4,6 aromatic Phe), 6.33 – 6.21 (m, 2H, 2 NH), 4.93 (s, 1H, C_αH Ser), 4.63 – 4.50 (m, 2H, CH_2O), 4.31 (s, 1H, C_αH Phe), 3.92 (s, 3H, $COOCH_3$), 3.60 – 3.50 (m, 2H, $C_\beta H_2$ Ser), 3.12 – 2.94 (m, 2H, $C_\beta H_2$ Phe), 2.30 (s, 3H, CH_3), 2.27 – 2.26 (m, 1H; C≡CH), 1.40 (s, 9H, $C(CH_3)_3$).

^{13}C NMR (101 MHz, $CDCl_3$) δ = 170.73, 167.12, 155.58, 138.41, 138.25, 136.65, 132.17 (C_6 aromatic), 130.49, 130.33, 130.20 (C aromatic Phe), 129.15 (C aromatic), 128.83, 128.73, 127.90 (C aromatic Phe), 126.49 (C aromatic Phe), 80.92 (*C*≡CH), 80.67 (*C*$(CH_3)_3$), 72.71, 71.96, 71.68, 55.98 (C_α Phe), 52.34 (OCH_3), 41.22 (C_α Ser), 38.48 (C_β Phe), 28.38 ((*C*$H_3)_3$), 21.51 (CH_3).

3-Methyl-L-phenylalanyl-*O*-(3-(methoxycarbonyl)benzyl)serine alkyne (16)

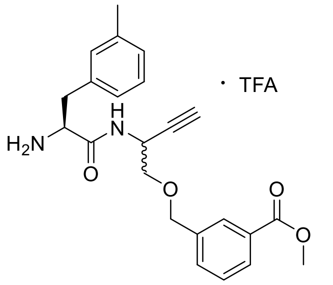

ESI (+):

m/z = 395.20 ($[M+H]^+$)

M ($C_{23}H_{27}N_2O_4^+$, monoisotopic): 359.20

The synthesis was accomplished according to GP II. using 0.03 g **15** (0.07 mmol) and 10 mL TFA/CH_2Cl_2 (1:1). 3-Methyl-L-phenylalanyl-*O*-(3-(methoxycarbonyl)benzyl)serine alkyne (**16**) was obtained as a white solid (0.04 g, quantitative yield; contained TFA and 41% undesired diastereomer).

^{1}H NMR (600 MHz, DMSO-*d*$_6$) δ = 9.01 (d, ^3J = 8.1 Hz, 1H, NH), 8.16 (broad s, 3H, NH_3^+), 7.96 (s, 1H, H-2 aromatic), 7.91 – 7.87 (m, 1H, H-4 aromatic), 7.65 – 7.57 (m, 1H, H-6 aromatic), 7.56 – 7.47 (m, 1H, H-5 aromatic), 7.21 (t, ^3J = 7.5 Hz, 1H, H-5 aromatic Phe), 7.11 – 6.95 (m, 3H, H-2,4,6 aromatic Phe), 4.84 – 4.76 (m, 1H, C_αH Ser), 4.68 – 4.60 (m, 2H, CH_2O), 3.99 (broad s, 1H, C_αH Phe), 3.86 (s, 3H, OCH_3), 3.62 – 3.52 (m, 1H, C≡CH), 3.45 – 3.32 (m, 2H, $C_\beta H_2$ Ser), 3.06 – 2.85 (m, 2H, $C_\beta H_2$ Phe), 2.29 (s, 3H, CH_3).

^{13}C NMR (151 MHz, DMSO-*d*$_6$) δ = 167.52 (CO), 166.30 (COO), 138.83, 137.72, 134.46, 132.48, 130.28, 129.82, 128.96, 128.49, 128.23, 128.22, 128.00, 126.78, 81.07 (*C*≡CH), 74.43, 71.64, 71.07, 53.44 (C_α Phe), 52.30 (OCH_3), 40.90 (C_α Ser), 36.85 (C_β Phe), 21.13 (CH_3).

N-(2,4-Difluorobenzoyl)-3-methyl-L-phenylalanyl-O-(3-methoxycarbonylbenzyl)serine alkyne (17)

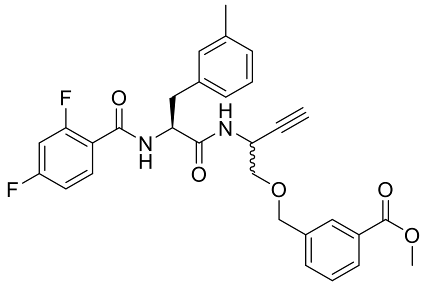

ESI (+):

m/z = 535.20 ($[M+H]^+$)

M ($C_{30}H_{29}F_2N_2O_5^+$, monoisotopic): 535.21

The synthesis was accomplished according to GP IV. using 0.03 g **16** (0.07 mmol, 1 eq.), 8 µL 2,4-difluorobenzoyl chloride (0.07 mmol, 1 eq.), 22 µL NMM (0.20 mmol, 2.9 eq.; instead of TEA) und 5 mL CH_2Cl_2. *N*-(2,4-Difluorobenzoyl)-3-methyl-L-phenylalanyl-*O*-(3-methoxycarbonylbenzyl)serine alkyne (**17**) was obtained as a white, wax-like solid (0.03 g, 0.05 mmol, 71%, contained undesired diastereomer at a ratio of 5 to 7 and 26% trifluoroacetylated side product).

^{1}H NMR (400 MHz, $CDCl_3$) δ = 8.14 – 7.89 (m, 3H, H-6 FBz, H-2,4 aromatic), 7.53 – 7.34 (m, 2H, H-5,6 aromatic), 7.22 – 7.13 (m, 1H, H-5 aromatic Phe), 7.12 – 7.00 (m, 3H, H-2,4,6 aromatic Phe), 6.99 – 6.90 (m, 1H, H-4 FBz), 6.88 – 6.79 (m, 1H, H-3 FBz), 6.20 (d, ^3J = 8.3 Hz, 1H, NH), 5.90 – 5.81 (m, 1H, NH), 4.96 – 4.86 (m, 1H, C_αH Ser), 4.85 – 4.75 (m, 1H, C_αH Phe), 4.56 – 4.52 (m, 2H, CH_2O), 3.92 – 3.90 (m, 3H, OCH_3), 3.57 – 3.31 (m, 2H, $C_\beta H_2$ Ser), 3.26 – 2.99 (m, 2H, $C_\beta H_2$ Phe), 2.32 – 2.27 (m, 4H, CH_3, C≡CH).

^{13}C NMR (101 MHz, $CDCl_3$) δ = 169.99 (CO), 168.44, 167.08, 165.99, 164.30, 162.48, 162.00, 160.48, 138.57, 138.12, 136.36, 132.12, 130.43, 130.22, 129.18, 129.11, 128.85, 128.77, 128.68, 128.05, 126.59, 126.49, 112.48, 104.50, 80.49 (*C*≡CH), 72.76, 72.02, 71.58, 55.45 (C_α Phe), 52.32 (OCH_3), 41.35 (C_α Ser), 38.41 (C_β Phe), 21.47 (CH_3).

Multiplicity of C-F signals was not considered.

^{19}F NMR (376 MHz, $CDCl_3$) δ = -103.53 (F aromatic), -108.32 (F aromatic).

N-(2,4-Difluorobenzoyl)-3-methyl-L-phenylalanyl-O-(3-carboxybenzyl)serine alkyne (18)

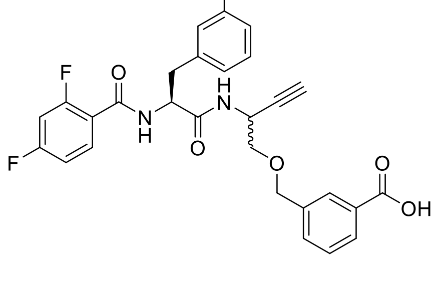

ESI (+):

m/z = 521.22 ($[M+H]^+$)

M ($C_{29}H_{27}F_2N_2O_5^+$, monoisotopic): 521.18

17 (0.02 g, 0.04 mmol, 1 eq.) was dissolved in THF/MeOH (3:1, 4 mL), NaOH (1 M, 98 µL, 0.10 mmol, 2.5 eq.) added and the solution stirred over night. The pH was adjusted to 7 with 1 M HCl and the solvent evaporated. The crude product was purified via semipreparative HPLC to yield *N*-(2,4-difluorobenzoyl)-3-methyl-L-phenylalanyl-*O*-(3-carboxybenzyl)serine alkyne (**18**) as a white solid (9.0 mg, 0.02 mmol, 44%; contained the undesired at a ratio of 4 to 6).

^{1}H NMR (400 MHz, $CDCl_3$) δ = 8.06 – 7.92 (m, 3H, H-2,4 BnCOO, H-6 FBz), 7.55 – 7.31 (m, 2H, H-5,6 BnCOO), 7.20 – 7.13 (m, 1H, H-5 Phe), 7.09 – 6.98 (m, 3H, H-2,4,6 Phe), 6.98 – 6.90 (m, 1H, aromatic FBz), 6.89 – 6.79 (m, 1H, aromatic FBz), 6.76 – 6.66 (m, 1H, NH), 6.50 – 6.43 (m, 1H, NH), 4.99 – 4.84 (m, 2H, C_αH Phe, C_αH Ser), 4.59 – 4.54 (m, 2H, CH_2O), 3.60 – 3.54 (m, 2H, $C_\beta H_2$ Ser), 3.25 – 3.16 (m, 1H, $C_\beta H\mathit{H}$ Phe), 3.12 – 3.03 (m, 1H, $C_\beta \mathit{H}H$ Phe), 2.30 (d, ^4J = 2.4 Hz, 1H, C≡CH), 2.29 (s, 3H, CH_3).

The carboxyl proton was not detectable in the chosen solvent.

^{13}C NMR (101 MHz, $CDCl_3$) δ = 170.23, 166.60, 162.81, 159.98, 138.61, 138.24, 135.98, 132.90, 130.39, 130.21, 129.77, 129.19, 128.87, 128.81, 128.13, 128.00, 126.56, 112.65, 112.44, 104.60, 99.58, 80.18, 72.59, 72.18, 71.48 (C_β Ser), 55.46 (C_α), 41.43 (C_α), 38.61 (C_β Phe), 21.46 (CH_3).

Multiplicity of C-F signals was not considered. The ratio of diastereomers was 6 to 4.

3 Synthesis of Dipeptide Alkyne 28 with Carboxy-functionalized 1,2,3-Triazolyl Residue in P2 (Scheme 3)

N-(*tert*-Butyloxycarbonyl)-5-azido-L-norvaline (19a)

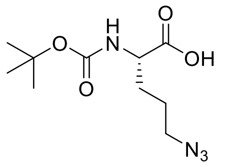

ESI (+):

m/z = 281.66 ([M+Na]$^+$)

M ($C_{10}H_{18}N_4O_4Na^+$, monoisotopic): 281.12

Prior to the synthesis, triflyl azide was freshly prepared. Therefore, sodium azide (1.27 g, 19.59 mmol, 9.1 eq.) was dissolved in H_2O/CH_2Cl_2 (2:1, 15 mL) cooled to 0°C before adding trifluoromethanesulfonic anhydride (651 µL, 3.87 mmol, 1.8 eq.). The solution was stirred 2 h at 0°C. The organic phase was separated and the aqueous phase carefully extracted with CH_2Cl_2 (2 × 5 mL). The combined organic layers were washed with sat. Na_2CO_3 (10 mL) and used without further purification. Due to the explosion risk, the solvent must not be evaporated.

Boc-L-ornithine (0.50 g, 2.15 mmol, 1 eq.), $CuSO_4 \times 5\ H_2O$ (0.01 g, 0.04 mmol, 0.02 eq.) and K_2CO_3 (0.32 g, 2.32 mmol, 1.08 eq.) were dissolved in $MeOH/H_2O$ (3:1, 40 mL). The previously prepared TfN_3 in CH_2Cl_2 was added slowly and the solution stirred over night. The reaction vessel was closed to prevent excessive evaporation of

solvent. CH_2Cl_2 (70 mL) was added and the organic phase extracted with H_2O (4 × 25 mL). The combined aqueous layers were acidified with HCl (3 M) to pH = 2, whereby a precipitate formed. The precipitate was redissolved by extraction with CH_2Cl_2 (6 × 20 mL). The organic phases were dried Na_2SO_4 and the solvent evaporated. *N*-(tert-Butyloxycarbonyl)-5-azido-L-norvaline (**19a**) was obtained as a yellowish solid (0.57 g, quantitative yield; contained residual solvent). PPh_3 (0.28 g, 1.08 mmol, 0.5 eq.) in ACN (20 mL) was slowly added to the combined washing solutions to inactivate excessive TfN_3.

^{1}H NMR (400 MHz, $CDCl_3$) δ = 5.04 (d, ^3J = 8.1 Hz, 1H, NH), 4.39 – 4.31 (m, 1H, $C_\alpha H$), 3.38 – 3.28 (m, 2H, $C_\delta H_2$), 2.03 – 1.90 (m, 1H, $C_\beta H$), 1.82 – 1.63 (m, 3H, $C_\beta \textit{H}H$, $C_\gamma H_2$), 1.46 (s, 9H, $(CH_3)_3$).

The carboxyl proton was not detectable in the chosen solvent.

^{13}C NMR (101 MHz, $CDCl_3$) δ = 176.49, 155.78, 80.70 ($C_{quart.}$), 52.97 (C_α), 51.00 (C_δ), 29.86 (C_β), 28.44 ($(CH_3)_3$), 25.07 (C_γ).

***N*-(*tert*-Butyloxycarbonyl)-(4-(methoxycarbonyl)-1*H*-1,2,3-triazol-1-yl)-L-norvaline (20a)**

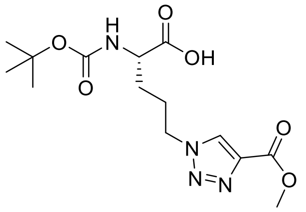

ESI (+):

m/z = 343.25 ($[M+H]^+$)

M ($C_{14}H_{23}N_4O_6^+$, monoisotopic): 343.16

The synthesis was accomplished according to GP VII. using 0.26 g **19a** (1.00 mmol, 1 eq.), 85 µL methyl propiolate (1.00 mmol, 1 eq.), 0.02 g sodium ascorbate (0.10 mmol, 0.1 eq.), 0.13 g $CuSO_4$×5 H_2O (0.50 mmol, 0.5 eq.) and 60 mL DMSO/H_2O. *N*-(*tert*-Butyloxycarbonyl)-(4-(methoxycarbonyl)-1*H*-1,2,3-triazol-1-yl)-L-norvaline (**20a**) was obtained as yellowish solid (0.31 g, 0.91 mmol, 69% over 2 steps; contained DMSO).

^{1}H NMR (400 MHz, $CDCl_3$) δ = 8.15 (s, 1H, CH aromatic), 5.20 (d, ^3J = 7.8 Hz, 1H, NH), 4.48 (t, ^3J = 7.1 Hz, 2H, $C_\delta H_2$), 4.40 – 4.35 (m, 1H, $C_\alpha H$), 3.95 (s, 3H, CH_3), 2.12 – 2.03 (m, 2H, $C_\gamma H_2$), 1.98 – 1.86 (m, 1H, $C_\beta H$), 1.77 – 1.64 (m, 1H, $C_\beta \textit{H}H$), 1.44 (s, 9H, $(CH_3)_3$).

The carboxyl proton was not detectable in the chosen solvent.

^{13}C NMR (101 MHz, $CDCl_3$) δ = 174.63, 161.31, 155.81, 140.10, 127.78, 80.64 ($C_{quart.}$), 52.51 (C_α), 52.40 (CH_3), 50.11 (C_δ), 29.69 (C_β), 28.44 ($(CH_3)_3$), 26.29 (C_γ).

(S)-2-(*tert*-Butyloxycarbonylamino)-5-(4-(methoxycarbonyl)-1*H*-1,2,3-triazol-1-yl)pentanol (21)

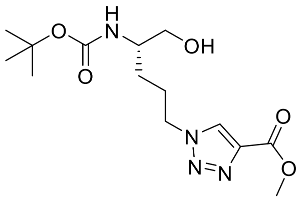

ESI (+):

m/z = 351.62 ($[M+Na]^+$)

M ($C_{14}H_{24}N_4NaO_5^+$, monoisotopic): 351.16

20a (0.99 g, 2.92 mmol, 1 eq.) and PyBOP (1.67 g, 3.21 mmol, 1.1 eq.) were dissolved in dry THF (50 mL). 5 min after adding DiPEA (612 µL, 3.51 mmol, 1.2 eq.) to the solution, $NaBH_4$ (0.11 g, 2.92 mmol, 1 eq.) was added and the solution stirred 1 h at room temperature. The solvent was evaporated and the residue dissolved in ethyl acetate/5% HCl (1:1, 30 mL). The organic phase was washed with 5% HCl (3 × 15 mL), saturated $NaHCO_3$ (3 × 15 mL) and brine (1 × 15 mL) and dried over Na_2SO_4. The solvent was evaporated. The crude product was purified *via* preparative column chromatography (petroleum ether/ethyl acetate (1:1) to ethyl acetate) to yield *(S)*-2-(*tert*-butyloxycarbonylamino)-5-(4-(methoxycarbonyl)-1*H*-1,2,3-triazol-1-yl)pentanol (**21**) as a white solid (0.31 g, 0.88 mmol, 31%).

^{1}H NMR (400 MHz, $CDCl_3$) δ = 8.13 (s, 1H, CH aromatic), 4.80 (d, ^3J = 8.6 Hz, 1H, NH), 4.47 (t, ^3J = 7.1 Hz, 2H, $C_\delta H_2$), 3.94 (s, 3H, CH_3) 3.74 – 3.49 (m, 3H, $C_\alpha H$, CH_2OH), 2.18 – 1.91 (m, 2H, $C_\gamma H_2$), 1.63 – 1.43 (m, 2H), 1.43 (s, 9H, $C(CH_3)_3$).

The hydroxyl proton was not detectable in the chosen solvent.

^{13}C NMR (101 MHz, $CDCl_3$) δ = 161.31, 156.38, 140.06, 127.68 (CH aromatic), 79.98, 65.26 (CH_2OH), 60.54, 52.35 (CH_3), 51.60 (C_α), 50.40 (C_δ), 28.50 (C_β, $(CH_3)_3$), 26.96 (C_γ).

(S)-2-(*tert*-Butyloxycarbonylamino)-5-(4-(methoxycarbonyl)-1*H*-1,2,3-triazol-1-yl)pentanal (22)

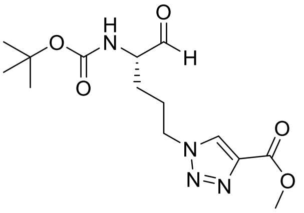

ESI (+):

m/z = 327.25 ($[M+H]^+$)

M ($C_{14}H_{23}N_4O_5^+$, monoisotopic: 327.17

21 (0.04 g, 0.12 mmol, 1 eq.) and Dess-Martin periodinane (0.10 g, 0.23 mmol, 2 eq.) was dissolved in dry CH_2Cl_2 (2 mL) and stirred 1 h at room temperature, after which time the oxidation was complete. 10% sodium thiosulfate in saturated $NaHCO_3$ (3 mL) was added and the solution stirred 15 min. Then the solution was extracted with CH_2Cl_2 (3 × 2 mL), the organic phases dried over $NaSO_4$ and the solvent evaporated. *(S)*-2-(*tert*-Butyloxycarbonylamino)-5-(4-(methoxycarbonyl)-1*H*-1,2,3-triazol-1-yl)pentanal

(**22**) was obtained as white solid (0.04 g, quantitative yield) and used without further purification and identification.

_N_-(_tert_-Butyloxycarbonyl)-(4-(methoxycarbonyl)-1_H_-1,2,3-triazol-1-yl)-L-norvaline alkyne (23)

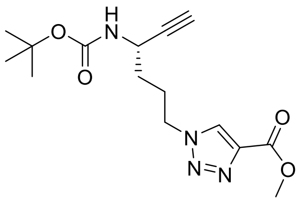

ESI (+):

m/z = 345.62 ($[M+Na]^+$)

M ($C_{15}H_{22}N_4O_4Na^+$, monoisotopic): 345.15

Dimethyl-(1-diazo-2-oxopropyl)phosphonate (56 µL, 0.23 mmol, 2 eq.) in MeOH (2 mL) was cooled to 0°C. K_2CO_3 (0.03 g, 0.23 mmol, 2 eq.) was added and stirred 2 h at 0°C. **22** (0.04 g, 0.12 mmol, 1 eq.) in MeOH (2 mL) was added dropwise, the solution allowed to warm up to room temperature and stirred for 3h (exposure to the potentially detrimental basic conditions should be kept as short as possible). Then H_2O/ethyl acetate (1:1, 10 mL) was added and the solution extracted with ethyl acetate (3 × 5 mL). The organic layer was washed with brine (2 × 5 mL), dried over Na_2SO_4 and the solvent evaporated. The crude product was purified *via* preparative column chromatography (petroleum ether/ethyl acetate (2:1) to petroleum ether/ethyl acetate (1:2)) to yield *N*-(*tert*-butyloxycarbonyl)-(4-(methoxycarbonyl)-1*H*-1,2,3-triazol-1-yl)-L-norvaline alkyne (**23**) as orange oil (0.01 g, 0.05 mmol, 39% over 2 steps) erhalten.**^{1}H NMR** (400 MHz, $CDCl_3$) δ = 8.12 (s, 1H, CH aromatic), 4.74 (s, 1H, NH), 4.54 – 4.38 (m, 3H, $C_\alpha H$, $C_\delta H_2$), 3.94 (s, 3H, CH_3), 2.30 (d, ^4J = 2.4 Hz, 1H, C≡CH), 2.14 – 2.03 (m, 2H, $C_\gamma H_2$), 1.75 – 1.63 (m, 2H, $C_\beta H_2$), 1.43 (s, 9H, $C(CH_3)_3$).

^{13}C NMR (101 MHz, $CDCl_3$) δ = 161.27, 154.91, 140.19, 127.57 (CH aromatic), 82.44, 80.51, 72.28, 52.33 (CH_3), 50.08 (C_γ), 42.14 (C_α), 33.09 (C_β), 28.45 ($(CH_3)_3$), 26.50 (C_γ).

(4-(Methoxycarbonyl)-1_H_-1,2,3-triazol-1-yl)-L-norvaline alkyne (24)

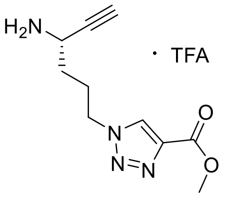

ESI (+):

m/z = 223.23 ($[M+H]^+$)

M ($C_{10}H_{15}N_4O_2^+$, monoisotopic): 223.12

The synthesis was accomplished according to GP II. using 0.02 g **23** (0.08 mmol) and 10 mL TFA/CH_2Cl_2 (1:1). (4-(Methoxycarbonyl)-1*H*-1,2,3-triazol-1-yl)-L-norvaline alkyne (**24**) was obtained as yellowish oil (0.03 g, quantitative yield; contained TFA).

^{1}H NMR (400 MHz, DMSO-d_6) δ = 8.83 (s, 1H, CH aromatic), 8.36 (s, 3H, NH_3^+), 4.50 (t, ^{3}J = 6.7 Hz, 2H, $C_\delta H_2$), 4.13 (s, 1H, C_αH), 3.84 (s, 3H, CH_3), 3.76 (d, ^{4}J = 2.3 Hz, 1H, C≡CH), 2.09 – 1.91 (m, 2H, $C_\gamma H_2$), 1.71 – 1.54 (m, 2H, $C_\beta H_2$).

^{13}C NMR (101 MHz, DMSO-d_6) δ = 160.71, 138.55, 129.20 (CH aromatic), 79.13, 78.60, 51.77 (CH_3), 48.89 (C_δ), 41.35 (C_α), 29.64 (C_β), 25.56 (C_γ).

***N*-(*tert*-Butyloxycarbonyl)-3-methyl-L-phenylalanyl-(4-(methoxycarbonyl)-1*H*-1,2,3-triazol-1-yl)-L-norvaline alkyne (25)**

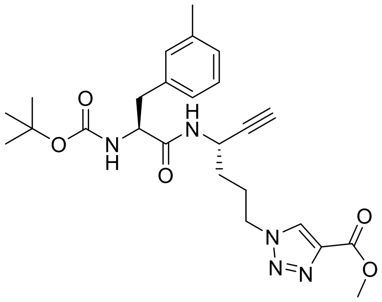

ESI (+):

m/z = 484.26 ([M+H]$^+$)

M ($C_{25}H_{34}N_5O_5^+$, monoisotopic): 484.26

The synthesis was accomplished according to GP III. using 0.03 g **24** (0.08 mmol, 1 eq.), 0.03 g Boc-(3-Me)Phe-OH (0.11 mmol, 1.4 eq.), 52 µL DiPEA (0.30 mmol, 3.8 eq.), 0.06 g PyBOP (0.11 mmol, 1.4 eq.) and 5 mL THF. The crude product was purified via preparative column chromatography (CH_2Cl_2 auf 3% MeOH/CH_2Cl_2) to yield *N*-(*tert*-butyloxycarbonyl)-3-methyl-L-phenylalanyl-(4-(methoxycarbonyl)-1*H*-1,2,3-triazol-1-yl)-L-norvaline alkyne (**25**) as yellow wax (0.02 g, 0.05 mmol, 64%, contained 22% undesired diastereomer). To minimize loss of product due to repeated purification steps, the separation of the diastereomers was performed at the stage of the final compound **28**.

^{1}H NMR (400 MHz, $CDCl_3$) δ = 8.15 (s, 1H, CH aromatic triazol), 7.19 – 7.12 (m, 1H, H-5 aromatic Phe), 7.05 – 6.94 (m, 3H, H-2,4,6 aromatic Phe), 6.22 (d, ^{3}J = 8.2 Hz, 1H, NH), 5.08 (broad s, 1H, NH), 4.76 – 4.68 (m, 1H, C_αH Phe), 4.49 – 4.35 (m, 2H, $C_\delta H_2$), 4.25 (m, 1H, C_αH Orn), 3.94 (s, 3H, CH_3O), 3.02 – 2.97 (m, 2H, $C_\beta H_2$ Phe), 2.29 (s, 3H, CH_3), 2.25 (d, ^{4}J = 2.4 Hz, 1H, C≡CH), 2.01 (p, ^{3}J = 7.3 Hz, 2H, $C_\gamma H_2$), 1.63 (m, 2H, $C_\beta H_2$ Orn), 1.38 (s, 9H, $(CH_3)_3$).

^{13}C NMR (101 MHz, $CDCl_3$) δ = 170.82, 161.28, 155.58, 140.12, 138.46, 136.37, 130.21 (CH aromatic), 128.77 (CH aromatic), 127.93 (CH aromatic), 127.68 (CH aromatic), 126.41 (CH aromatic), 81.68, 80.48, 72.42, 56.15 (C_α Orn), 52.31 (CH_3O), 49.97 (C_δ), 40.65 (C_α Phe), 38.28 (C_β Phe), 32.43 (C_β Orn), 28.35 ($(CH_3)_3$), 26.30 (C_γ), 21.49 (CH_3).

3-Methyl-L-phenylalanyl-(4-(methoxycarbonyl)-1*H*-1,2,3-triazol-1-yl)-L-norvaline alkyne (26)

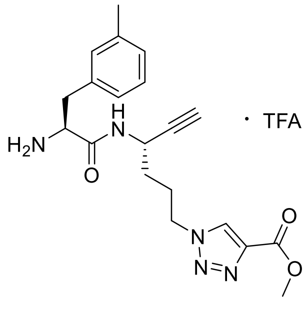

ESI (+):

m/z = 384.19 ([M+H]$^+$)

M ($C_{20}H_{26}N_5O^+$, monoisotopic): 384.20

The synthesis was accomplished according to GP II. using 0.02 g **25** (0.05 mmol) and 10 mL TFA/CH_2Cl_2 (1:1). 3-Methyl-L-phenylalanyl-(4-(methoxycarbonyl)-1*H*-1,2,3-triazol-1-yl)-L-norvaline alkyne (**26**) was obtained as white solid (0.03 g, quantitative yield; contained 16% undesired diastereomer).

^{1}H NMR (400 MHz, DMSO-d_6) δ = 8.80 (s, 1H, CH aromatic triazol), 8.13 (broad s, 3H, NH_3^+), 7.21 (t, ^3J = 7.5 Hz, 1H, H-5 aromatic Phe), 7.11 – 6.99 (m, 4H, H-2,4,6 aromatic Phe, NH), 4.60 – 4.53 (m, 1H, C_αH Phe), 4.47 (t, ^3J = 6.8 Hz, 2H, $C_\delta H_2$), 3.88 (s, 1H, C_αH Orn), 3.84 (s, 3H, CH_3O), 3.32 (d, ^4J = 2.3 Hz, 1H, C≡CH), 3.02 – 2.89 (m, 2H, $C_\beta H_2$ Phe), 2.28 (s, 3H, CH_3), 2.02 – 1.90 (m, 2H, $C_\gamma H_2$), 1.59 – 1.49 (m, 2H, $C_\beta H_2$ Orn).

^{13}C NMR (101 MHz, DMSO-d_6) δ = 167.06, 160.75, 138.56, 137.60, 134.45, 130.12 (CH aromatic Phe), 129.05 (CH triazol), 128.46 (C_5 aromatic Phe), 127.85 (CH aromatic Phe), 126.64 (CH aromatic Phe), 82.52, 74.09, 53.42 (C_α Orn), 51.80 (CH_3O), 49.18 (C_δ), 42.09, 36.75 (C_β Phe), 31.57 (C_β Orn), 25.94 (C_δ), 21.06 (CH_3).

***N*-(4-Fluorobenzoyl)-3-methyl-L-phenylalanyl-(4-(methoxycarbonyl)-1*H*-1,2,3-triazol-1-yl)-L-norvaline alkyne (27)**

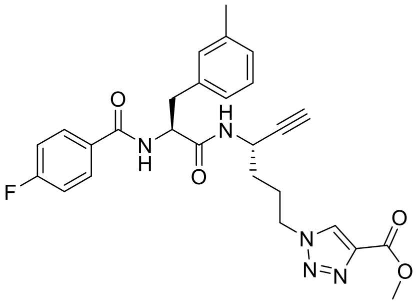

ESI (+):

m/z = 506.54 ([M+H]$^+$)

M ($C_{27}H_{29}FN_5O_4^+$, monoisotopic): 506.22

The synthesis was accomplished according to GP IV. using 0.02 g **26** (0.04 mmol, 1 eq.), 10 µL 4-fluorobenzoyl chloride (0.09 mmol, 2 eq.), 14 µL NMM (0,13 mmol, 3 eq., instead of TEA) and 5 mL CH_2Cl_2. *N*-(4-Fluorobenzoyl)-3-methyl-L-phenylalanyl-(4-(methoxycarbonyl)-1*H*-1,2,3-triazol-1-yl)-L-norvaline alkyne (**27**) was obtained as a white solid (0.02 g, 0.04 mmol, 43%, contained undesired diastereomer in a ratio of 3:7 and 52% trifluoroacetylated side product).

^{1}H NMR (400 MHz, $CDCl_3$) δ = 8.09 (s, 1H, NH triazol), 7.77 – 7.72 (m, 2H, H-2,6 FBz), 7.24 – 7.01 (m, 6H, H-3,5 FBz, aromatic Phe), 6.82 (d, ^3J = 7.2 Hz, 1H, NH), 6.03 (d, ^3J = 8.1 Hz, 1H, NH), 4.76 – 4.66 (m, 2H, C_αH Phe, C_αH Orn), 4.44 – 4.35 (m, 3H, $C_\delta H_2$), 3.94 (s, 3H, OCH_3), 3.24 – 3.16 (m, 1H, C_β*H*H Phe), 3.10 – 3.02 (m, 1H, C_βH*H* Phe), 2.32 (s, 3H, CH_3), 2.28 (d, ^4J = 2.4 Hz, 1H, C≡CH), 2.06 – 1.96 (m, 2H, $C_\gamma H_2$), 1.70 – 1.60 (m, 2H, $C_\beta H_2$ Orn).

^{13}C NMR (101 MHz, $CDCl_3$) δ = 170.33 (CO), 166.42 (CO), 161.25 (COO), 158.67 (d, $^1J_{C,F}$ = 247.1 Hz, CF), 138.77 (C_2 Phe), 136.25 (C-1 Phe), 132.98, 130.27, 129.65, 129.56, 129.00, 128.59, 128.22, 127.56, 126.46 (CH triazol), 115.93 (d, $^2J_{C,F}$ = 22.0 Hz, C-3/5 FBz), 81.41 (*C*≡CH), 72.62 (C≡*C*H), 55.38 (C_α Phe), 52.36 (OCH_3), 49.94 (C_δ), 40.82 (C_α Orn), 38.41 (C_β Phe), 32.34 (C_β Orn), 26.22 (C_γ), 21.53 (CH_3).

N-(4-Fluorobenzoyl)-3-methyl-L-phenylalanyl-(4-carboxy-1*H*-1,2,3-triazol-1-yl)-L-norvaline alkyne (28)

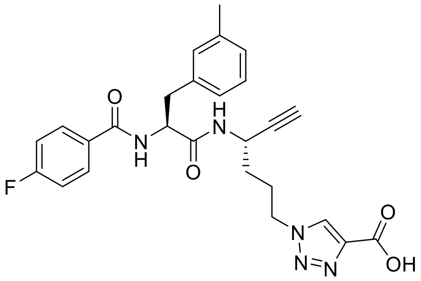

HR-MS ESI (+):

m/z = 514.186 ([M+Na]$^+$)

M ($C_{26}H_{25}FN_5NaO_4^+$, monoisotopic): 514.1861

27 (0.01 g, 0.03 mmol, 1 eq.) was dissolved in THF/MeOH (3:1, 4 mL) and 1 M NaOH (63 µL, 0.06 mmol, 3.5 eq.) added dropwise. The solution was stirred over night and then neutralized with 1 M HCl before evaporating the solvent. The crude product was purified *via* semipreparative HPLC to yield *N*-(4-fluorobenzoyl)-3-methyl-L-phenylalanyl-(4-carboxy-1*H*-1,2,3-triazol-1-yl)-L-norvaline alkyne (**28**) as a white solid (5.0 mg, 0.01 mmol, 32%).

^{1}H NMR (400 MHz, CD_3CN) δ = 8.28 (s, 1H, CH triazol), 7.79 – 7.70 (m, 2H, H-2,6 FBz), 7.22 – 6.98 (m, 7H, aromatic Phe, FBz, NH), 6.96 (d, ^3J = 8.4 Hz, 1H, NH), 4.73 – 4.58 (m, 2H, C_αH Phe, C_αH Ser), 4.41 (t, ^3J = 7.1 Hz, 2H, $C_\delta H_2$), 3.21 – 3.10 (m, 1H, C_βH*H* Phe), 3.02 – 2.91 (m, 1H, C_β*H*H Phe), 2.54 (d, ^4J = 2.4 Hz, 1H, C≡CH), 2.27 (s, 3H, CH_3), 2.02 – 1.96 (m, 2H, $C_\gamma H_2$), 1.71 – 1.59 (m, 2H, $C_\beta H_2$ Ser).

^{13}C NMR (101 MHz, CD_3CN) δ = 171.26, 166.86, 165.62 (d, $^1J_{C,F}$ = 249.4 Hz, CF), 161.79, 140.16, 138.94, 138.36, 131.40 (d, $^4J_{C,F}$ = 2.9 Hz, C-1 FBz) 131.04, 130.74 (d, $^3J_{C,F}$ = 9.1 Hz, C-2/6 FBz), 129.38, 129.26, 128.26, 127.30, 116.25 (d, $^2J_{C,F}$ = 22.2 Hz, C-3/5 FBz), 83.62, 72.38, 56.12 (C_α), 50.53 (C_δ), 41.05 (C_α), 38.11 (C_β Phe), 32.81 (C_β Orn), 26.95 (C_γ), 21.39 (CH_3).

Diastereomeric purity > 99%

4 Synthesis of Dipeptide Nitriles 35a – c with Carboxy-functionalized 1,2,3-Triazolyl Residue in P2 (Scheme 4)

N-(*tert*-Butyloxycarbonylamino)-6-azido-L-norleucine (19b)

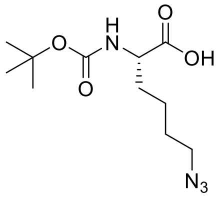

ESI (+):

m/z = 295.10 ([M+Na]$^+$)

M ($C_{11}H_{20}N_4NaO_4^+$, monoisotopic): 295.14

TfN_3 was prepared freshly prior to the synthesis. Therefore, NaN_3 (1.27 g, 19.59 mmol, 9.1 eq.) was dissolved in H_2O/CH_2Cl_2 (2:1, 15 mL) and the solution cooled to 0°C before adding trifluoromethanesulfonic anhydrid (651 µL, 3.87 mmol, 1.8 eq.). The solution was stirred 2 h at 0°C. Then the organic phase was separated and the aqueous phase extracted carefully with CH_2Cl_2 (2 × 5 mL). The combined organic layers were washed with saturated Na_2CO_3 (10 mL) and used without further purification. Due to the explosion risk the solvent must not be evaporated.

Boc-L-lysine (0.50 g, 2.03 mmol, 1 eq.), $CuSO_4$×5 H_2O (0.01 g, 0.04 mmol, 0.02 eq.) and K_2CO_3 (0.32 g, 2.32 mmol, 1.08 eq.) were dissolved in $MeOH/H_2O$ (3:1, 40 mL). The freshly prepared TfN_3 in CH_2Cl_2 was added slowly and the solution stirred over night. After adding CH_2Cl_2 (70 mL), the organic phase was extracted with H_2O (4 × 25 mL). The combined aqueous layers were acidified to pH = 2.0 with 3 M HCl. The formed precipitate was redissolved via extraction with CH_2Cl_2 (6 × 20 mL) and the organic phase dried over Na_2SO_4. The solvent was evaporated to yield *N*-(*tert*-butyloxycarbonyl)-5-azido-L-norvaline (**19b**) as a clear oil (0.28 g, 1.04 mmol, 51%). **19b** was used without further purification.

PPh_3 (0.28 g, 1.08 mmol, 0.5 eq.) in ACN (20 mL) was added to the combined washing solutions to deactivate remaining TfN_3.

N-(*tert*-Butyloxycarbonyl)-(4-(2-methoxy-2-oxoethyl)-1*H*-1,2,3-triazol-1-yl)-L-norvaline (20b)

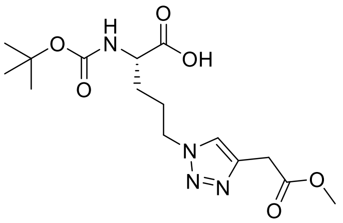

ESI (+):

m/z = 357.22 ([M+H]$^+$)

M ($C_{15}H_{25}N_4O_6^+$, monoisotopic): 357.18

The synthesis was accomplished according to GP VII. using 0.26 g **19a** (1.00 mmol, 1 eq.), 100 µL methyl butynoate (1.00 mmol, 1 eq.), 0.02 g sodium ascorbate (0.10 mmol, 0.1 eq.), 0.13 g $CuSO_4$×5 H_2O (0.50 mmol, 0.5 eq.) and 60 mL

DMSO/H_2O. The crude product was purified *via* preparative column chromatography (petroleum ether/ethyl acetate (1:2) with 2% acetic acid to petroleum ether/ethyl acetate (3:1) with 2% acetic acid). The organic phases were combined and the solvent evaporated. The residue was washed with toluol (4 × 20 mL) to remove remaining acetic acid and the product was lyophilized. *N*-(*tert*-Butyloxycarbonyl)-(4-(methoxycarbonyl)-1*H*-1,2,3-triazol-1-yl)-L-norvaline (**20b**) was obtained as a white solid (0.11 g, 0.32 mmol, 30% over 2 steps).

^{1}H NMR (400 MHz, $CDCl_3$) δ = 7.64 (s, 1H, H aromatic), 5.23 (d, ^3J = 7.2 Hz, 1H, NH), 4.43 – 4.30 (m, 3H, $C_{\delta}H_2$, $C_{\alpha}H$), 3.86 (s, 2H, CH_2COO), 3.74 (s, 3H, CH_3), 2.09 – 1.86 (m, 3H, $C_{\gamma}H_2$, $C_{\beta}H\mathit{H}$), 1.72 (m, 1H, $C_{\beta}\mathit{H}H$), 1.44 (s, 9H, $(CH_3)_3$).

The carboxyl proton was not detectable in the chosen solvent.

^{13}C NMR (101 MHz, $CDCl_3$) δ =174.27 (CO), 170.97 (CO), 155.81 (CO), 140.38 ($C_{quart.}$ triazol), 123.24 (CH aromatic), 80.50 ($\mathit{C}(CH_3)_3$), 52.74 (C_{α}), 52.54 (CH_3), 50.10 (C_{δ}), 31.47 ($\mathit{C}H_2COO$), 29.50 (C_{β}), 28.46 ($(CH_3)_3$), 26.10 (C_{γ}).

N-(*tert*-Butyloxycarbonyl)-(4-(methoxycarbonyl)-1*H*-1,2,3-triazol-1-yl)-L-norleucine (20c)

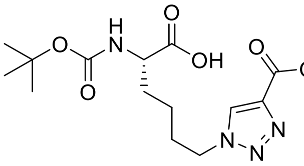

ESI (+):

m/z = 379.14 ([M+H]$^+$)

M ($C_{15}H_{24}N_4NaO_6^+$, monoisotopic): 379.16

The synthesis was accomplished according to GP VII. using 0.27 g **19b** (1.00 mmol, 1 eq.), 85 µL methyl propiolate (1.00 mmol, 1 eq.), 0.02 g sodium ascorbate (0.10 mmol, 0.1 eq.), 0.13 g $CuSO_4$×5 H_2O (0.50 mmol, 0.5 Eq.) and 60 mL DMSO/H_2O (2:1). *N*-(*tert*-Butyloxycarbonyl)-(4-(methoxycarbonyl)-1*H*-1,2,3-triazol-1-yl)-L-norleucine (**20c**) was obtained as orange oil (0.37 g, 1.05 mmol, 77%, contained residual solvent).

^{1}H NMR (400 MHz, $CDCl_3$) δ = 8.06 (s, 1H, CH aromatic), 5.05 (d, ^3J = 7.9 Hz, 1H, NH), 4.38 (t, ^3J = 7.1 Hz, 2H, $C_{\varepsilon}H_2$), 4.27 (broad s, 1H, $C_{\alpha}H$), 3.90 (s, 3H, CH_3), 1.98 – 1.90 (m, 2H, $C_{\gamma}H_2$), 1.90 – 1.82 (m, 1H, $C_{\beta}H\mathit{H}$), 1.74 – 1.66 (m, 1H, $C_{\beta}\mathit{H}H$), 1.39 (s, 11H, $C_{\delta}H_2$, $(CH_3)_3$).

The carboxyl proton was not detectable in the chosen solvent.

^{13}C NMR (101 MHz, $CDCl_3$) δ = 175.85 (COO), 161.32 ($\mathit{C}OOCH_3$), 155.71 (CONH), 140.08 ($C_{quart.}$ triazol), 127.57 (CH triazol), 80.43 ($C_{quart.}$), 52.96 (C_{α}), 52.37 (OCH_3), 50.50 (C_{ε}), 31.92 (C_{β}), 29.65 (CH_3), 28.43 (C_{δ}), 22.22 (C_{γ}).

_N_-(_tert_-Butyloxycarbonyl)-(4-(methoxycarbonyl)-1_H_-1,2,3-triazol-1-yl)-L-norvaline amide (29a)

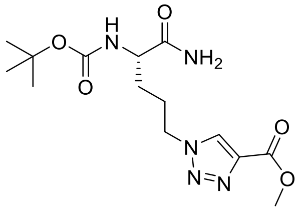

ESI (+):

m/z = 364.12 ([M+Na]$^+$)

M ($C_{14}H_{23}N_5NaO_5^+$, monoisotopic): 364.16

The synthesis was accomplished according to GP I. using 0.30 g **20a** (0.88 mmol, 1 eq.), 289 µL NMM (2.63 mmol, 2.7 eq.), 125 µL isobutyl chloroformate (0.96 mmol, 1.1 eq.), 682 µL NH_3 (25%, 4.38 mmol, 5 eq.) and 15 mL THF. *N*-(*tert*-Butyloxycarbonyl)-(4-(methoxycarbonyl)-1*H*-1,2,3-triazol-1-yl)-L-norvaline amide (**29a**) was obtained as a pinkish solid (0.10 g, 0.30 mmol, 36%).

^{1}H NMR (400 MHz, $CDCl_3$) δ = 8.12 (s, 1H, CH aromatic), 6.21 (broad s, 1H, CONH*H*), 5.46 (broad s, 1H, CON*H*H), 5.19 (d, ^3J = 8.1 Hz, 1H, NH), 4.63 – 4.40 (m, 2H, $C_\delta H_2$), 4.34 – 4.24 (m, 1H, $C_\alpha H$), 3.95 (s, 3H, CH_3), 2.05 (p, ^3J = 7.2 Hz, 2H, $C_\gamma H_2$), 1.86 – 1.76 (m, 1H, $C_\beta H$*H*), 1.68 – 1.57 (m, 1H, *$C_\beta H$*H), 1.44 (s, 9H, $(CH_3)_3$).

^{13}C NMR (101 MHz, $CDCl_3$) δ = 173.70 (CO), 161.21 (CO), 155.96 (CO), 140.22 ($C_{quart.}$ triazol), 127.76 (CH triazol), 80.63 ($C_{quart.}$), 52.68 (C_α), 52.39 (CH_3), 49.91 (C_δ), 29.74 (C_β), 28.45 ($(CH_3)_3$), 26.33 (C_γ).

_N_-(_tert_-Butyloxycarbonyl)-(4-(2-methoxy-2-oxoethyl)-1_H_-1,2,3-triazol-1-yl)-L-norvaline amide (29b)

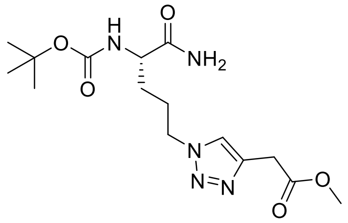

ESI (+):

m/z = 356.16 ([M+H]$^+$),

M ($C_{15}H_{26}N_5O_5^+$, monoisotopic): 356.19

The synthesis was accomplished according to GP I. using 0.15 g **20b** (0.43 mmol, 1 eq.), 141 µL NMM (1.28 mmol, 3 eq.), 55 µL isobutyl chloroformate (0.43 mmol, 1 eq.), 160 µL NH_3 (25%, 3.13 mmol, 7.3 Eq.) and 10 mL THF. *N*-(*tert*-Butyloxycarbonyl)-(4-(2-methoxy-2-oxoethyl)-1*H*-1,2,3-triazol-1-yl)-L-norvaline amide (**29b**) was obtained as a yellow oil (0.12 g, 0.34 mmol, 79%).

^{1}H NMR (400 MHz, $CDCl_3$) δ = 7.62 (s, 1H, CH aromatic), 6.21 (s, 1H, CONH*H*), 5.44 (s, 1H, CON*H*H), 5.20 (d, ^3J = 8.0 Hz, 1H, NH), 4.54 – 4.36 (m, 2H, $C_\delta H_2$), 4.23 (s, 1H, $C_\alpha H$), 3.84 (s, 2H, CH_2COO), 3.74 (s, 3H, CH_3), 2.01 (p, ^3J = 7.2 Hz, 2H, $C_\gamma H_2$), 1.86 – 1.55 (m, 2H, $C_\beta H_2$), 1.44 (s, 9H, $(CH_3)_3$).

^{13}C NMR (101 MHz, $CDCl_3$) δ = 173.82 (CO), 170.77 (CO), 155.95 (CO), 140.57 ($C_{quart.}$ triazol), 123.28 (CH triazol), 80.47 ($C_{quart.}$), 52.70 ($C_α$), 52.47 (CH_3), 49.68 ($C_δ$), 31.60 (CH_2COO), 29.83 ($C_β$), 28.46 ($(CH_3)_3$), 26.22 ($C_γ$).

_N_-(_tert_-Butyloxycarbonyl)-(4-(methoxycarbonyl)-1_H_-1,2,3-triazol-1-yl)-L-norleucine amide (29c)

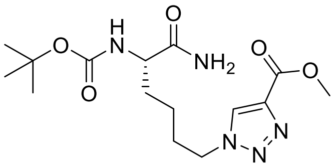

ESI (+):

m/z = 378.11 ($[M+Na]^+$)

M ($C_{15}H_{25}N_5NaO_5^+$, monoisotopic): 378.17

The synthesis was accomplished according to GP I. using 0.13 g **20c** (0.35 mmol, 1 eq.), 116 µL NMM (1.05 mmol, 3 eq.), 46 µL iBCF (0.35 mmol, 1 eq.), 132 µL NH_3 (25%, 1.76 mmol, 5 eq.) and 10 mL THF. *N*-(*tert*-Butyloxycarbonyl)-(4-(methoxycarbonyl)-1*H*-1,2,3-triazol-1-yl)-L-norleucine amide (**29c**) was obtained as an orange solid (0.10 g, 0.29 mmol, 83%). The product was used without further characterization.

(4-(Methoxycarbonyl)-1_H_-1,2,3-triazol-1-yl)-L-norvaline amide (30a)

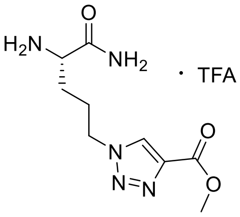

ESI (+):

m/z = 242.09 ($[M+H]^+$)

M ($C_9H_{16}N_5O_3^+$, monoisotopic): 242.12

The synthesis was accomplished according to GP II. using 0.10 g **29a** (0.29 mmol) and 10 mL TFA/CH_2Cl_2 (1:1). (4-(Methoxycarbonyl)-1*H*-1,2,3-triazol-1-yl)-L-norvaline amide (**30a**) was obtained as a white solid (0.12 g, quantitative yield; contained TFA).

^{1}H NMR (400 MHz, DMSO-d_6) δ = 8.81 (s, 1H, CH aromatic), 8.04 (d, ^3J = 5.0 Hz, 3H, NH_3^+), 7.84 (s, 1H, CONH*H*), 7.60 (s, 1H, CON*H*H), 4.46 (t, ^3J = 6.9 Hz, 2H, $C_δH_2$), 3.84 (s, 3H, CH_3), 3.77 – 3.64 (m, 1H, $C_αH$), 1.96 – 1.84 (m, 2H, $C_γH_2$), 1.69 – 1.60 (m, 2H, $C_βH_2$).

^{13}C NMR (101 MHz, DMSO-d_6) δ = 170.03 (CO), 160.76 (CO), 138.54 ($C_{quart.}$ triazol), 129.24 (CH triazol), 51.82 (CH_3), 51.65 ($C_α$), 49.14 ($C_δ$), 27.82 ($C_β$), 25.14 ($C_γ$).

(4-(2-Methoxy-2-oxoethyl)-1*H*-1,2,3-triazol-1-yl)-L-norvaline amide (30b)

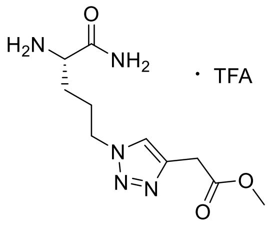

ESI (+):

m/z = 256.28 ([M+H]$^+$)

M ($C_{10}H_{18}N_5O_3^+$, monoisotopic): 256.14

The synthesis was accomplished according to GP II. using 0.02 g **29b** (0.05 mmol) and 10 mL TFA/CH_2Cl_2 (1:1). (4-(2-Methoxy-2-oxoethyl)-1*H*-1,2,3-triazol-1-yl)-L-norvaline amide (**30b**) was obtained as a clear oil (0.02 g, quantitative yield; contained TFA).

^{1}H NMR (400 MHz, DMSO-*d$_6$*) δ = 7.86 (s, 1H, CH triazol), 7.24 (s, 1H, CONH*H*), 6.94 (s, 1H, CON*H*H), 6.76 (d, ^3J = 8.3 Hz, 3H, NH_3^+), 4.30 (t, ^3J = 7.1 Hz, 2H, $C_δH_2$), 3.92 – 3.83 (m, 1H, $C_αH$), 3.63 (s, 2H, CH_2COO), 3.49 (s, 3H, OCH_3), 1.84 – 1.74 (m, 2H, $C_γH_2$), 1.51 – 1.42 (m, 2H, $C_βH_2$).

^{13}C NMR (101 MHz, DMSO-*d$_6$*) δ = 173.67 (CO), 172.16 (COO), 146.76 ($C_{quart.}$ triazol), 122.89 (CH triazol), 53.47 ($C_α$), 51.69 (CH_3), 48.81 ($C_δ$), 33.07 (*C*H_2COO), 29.02 ($C_β$), 26.48 ($C_γ$).

(4-(Methoxycarbonyl)-1*H*-1,2,3-triazol-1-yl)-L-norleucine amide (30c)

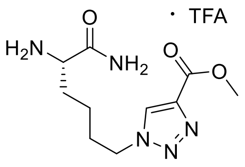

ESI (+):

m/z = 255.92 ([M+H]$^+$)

M ($C_{10}H_{18}N_5O_3^+$, monoisotopic): 256.14

The synthesis was accomplished according to GP II. using 0.10 g **29c** (0.27 mmol) and 10 mL TFA/CH_2Cl_2 (1:1). (4-(Methoxycarbonyl)-1*H*-1,2,3-triazol-1-yl)-L-norleucine amide (**30c**) was obtained as a clear oil (0.14 g, quantitative yield, contained TFA).

^{1}H NMR (400 MHz, DMSO-*d$_6$*) δ = 8.80 (s, 1H, CH triazol), 8.01 (broad s, 3H, NH_3^+), 7.80 (s, 1H, CONH*H*), 7.55 (s, 1H, CON*H*H), 4.43 (t, ^3J = 6.8 Hz, 2H, $C_εH_2$), 3.84 (s, 3H, CH_3), 3.71 – 3.60 (m, 1H, $C_αH$), 1.87 (p, ^3J = 7.4 Hz, 2H, $C_δH_2$), 1.78 – 1.63 (m, 2H, $C_βH_2$), 1.31 – 1.17 (m, 2H, $C_γH_2$).

^{13}C NMR (101 MHz, DMSO-*d$_6$*) δ = 170.28 (COO), 160.78 (CO), 138.53 ($C_{quart.}$ triazol), 129.02 (CH triazol), 51.94 ($C_α$), 51.74 (CH_3), 49.44 ($C_ε$), 30.28 ($C_β$), 28.89 ($C_δ$), 21.07 ($C_γ$).

N-(tert-Butyloxycarbonyl)-3-methyl-L-phenylalanyl-(4-(methoxycarbonyl)-1H-1,2,3-triazol-1-yl)-L-norvaline alkyne (31a)

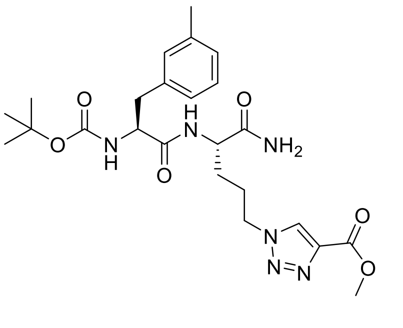

ESI (+):

m/z = 503.55 ([M+H]$^+$)

M ($C_{24}H_{35}N_6O_6^+$, monoisotopic): 503.26

The synthesis was accomplished according to GP III. using 0.11 g **30a** (0.30 mmol, 1 eq.), 0.13 g Boc-(3-Me)Phe-OH (0.45 mmol, 1.5 eq.), 210 µL DiPEA (1.21 mmol, 4 eq.), 0.24 g PyBOP (0.45 mmol, 1.5 eq.) and 25 mL THF. The crude product was purified via preparative column chromatography (CH_2Cl_2 to 3% MeOH/CH_2Cl_2). *N*-(*tert*-Butyloxycarbonyl)-3-methyl-L-phenylalanyl-(4-(methoxycarbonyl)-1*H*-1,2,3-triazol-1-yl)-L-norvaline alkyne (**31a**) was obtained as a white solid (0.13 g, 0.25 mmol, 83%).

^{1}H NMR (400 MHz, $CDCl_3$) δ = 8.11 (s, 1H, CH triazol), 7.18 (t, ^3J = 7.5 Hz, 1H, H-5 aromatic Phe), 7.08 – 6.97 (m, 3H, H-2,4,6 aromatic Phe), 6.60 (d, ^3J = 8.3 Hz, 1H, NH), 6.24 (broad s, 1H, CON*H*H), 5.31 (broad s, 1H, CONH*H*), 5.04 (d, ^3J = 6.5 Hz, 1H, NH), 4.57 – 4.46 (m, 2H, $C_{\delta}H_2$), 4.45 – 4.35 (m, 1H, $C_{\alpha}H$ Phe), 4.32 - 4.254 (m, 1H, $C_{\alpha}H$ Norvalin), 3.94 (s, 3H, CH_3O), 3.10 – 2.96 (m, 2H, $C_{\beta}H_2$ Phe), 2.31 (s, 3H, CH_3), 2.00 – 1.85 (m, 3H, $C_{\gamma}H_2$, $C_{\beta}H$*H* Norvalin), 1.63 – 1.52 (m, 2H, C_{β}*H*H Norvalin), 1.39 (s, 9H, $(CH_3)_3$).

^{13}C NMR (101 MHz, $CDCl_3$) δ = 172.90 (CO), 172.00 (CO), 161.28 (CO), 156.04 (CO), 140.22, 138.74, 136.25, 130.17 (CH aromatic), 129.00 (CH aromatic), 128.16 (CH aromatic), 127.79 (CH aromatic), 126.38 (CH triazol), 81.01 (*C*(CH_3)$_3$), 56.79 (C_{α} Phe), 52.39 (CH_3O), 51.73 (C_{α} norvaline) 50.03 (C_{δ}), 37.87 (C_{β} Phe), 28.99 (C_{β} norvaline), 28.35 ((CH_3)$_3$), 26.30 (C_{γ}), 21.49 (CH_3).

N-(tert-Butyloxycarbonyl)-3-methyl-L-phenylalanyl-(4-(2-methoxy-2-oxoethyl)-1H-1,2,3-triazol-1-yl)-L-norvaline amide (31b)

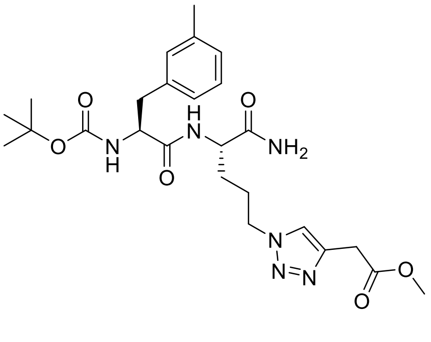

ESI (+):

m/z = 517.3 ([M+H]$^+$)

M ($C_{25}H_{37}N_6O_6^+$, monoisotopic): 517.28

The synthesis was accomplished according to GP III. using 0.02 g **30b** (0.05 mmol, 1 eq.), 0.02 g Boc-(3-Me)Phe-OH (0.08 mmol, 1.5 eq.), 38 µL DiPEA (0.22 mmol,

4 eq.), 0.04 g PyBOP (0.08 mmol, 1.5 eq.) and 5 mL THF. The crude product was purified *via* preparative column chromatography (CH_2Cl_2 to 5% MeOH/CH_2Cl_2). *N*-(*tert*-Butyloxycarbonyl)-3-methyl-L-phenylalanyl-(4-(2-methoxy-2-oxoethyl)-1*H*-1,2,3-triazol-1-yl)-L-norvaline amide (**31b**) was obtained as a white solid (0.01 g, 0.02 mmol, 58%).

^{1}H NMR (400 MHz, CD_3CN) δ = 7.69 (s, 1H, CH aromatic triazol), 7.17 (t, ^3J = 7.5 Hz, 1H, H-5 aromatic Phe), 7.08 – 6.98 (m, 3H, H-2,4,6 aromatic Phe), 6.91 (d, ^3J = 7.9 Hz, 1H, NH), 6.21 (broad s, 1H, CONH*H*), 5.69 (broad s, 1H, CON*H*H), 5.54 (broad s, 1H, NH), 4.33 (t, ^3J = 6.9 Hz, 2H, $C_δH_2$), 4.30 – 4.14 (m, 2H, $C_α$H Phe, $C_α$H norvaline), 3.74 (s, 2H, CH_2COO), 3.67 (s, 3H, OCH_3), 3.07 – 2.98 (m, 1H, $C_β$H*H* Phe), 2.87 – 2.76 (m, 1H, $C_β$*H*H Phe), 2.29 (s, 3H, CH_3), 1.88 – 1.73 (m, 3H, $C_γH_2$, $C_β$H*H* norvaline), 1.60 – 1.49 (m, 1H, $C_β$*H*H norvaline), 1.34 (s, 9H, $(CH_3)_3$).

^{13}C NMR (101 MHz, CD_3CN) δ = 173.97 (CO), 172.60 (CO), 171.77 (CO), 156.70 (CO), 141.33, 139.01, 138.31, 130.95, 129.30 (C_5 aromatic Phe), 128.32, 127.24, 123.98 (CH triazol), 80.29 (*C*$(CH_3)_3$), 57.27 ($C_α$ norvaline), 53.09 ($C_α$ Phe), 52.61 (OCH_3), 50.28 ($C_δ$), 38.12 ($C_β$ Phe), 32.17 (*C*H_2COO), 29.57 ($C_β$ norvaline), 28.46 ($(CH_3)_3$), 27.20 ($C_γ$), 21.40 (CH_3).

***N*-(*tert*-Butyloxycarbonyl)-3-methyl-L-phenylalanyl-(4-(methoxycarbonyl)-1*H*-1,2,3-triazol-1-yl)-L-norleucine amide (31c)**

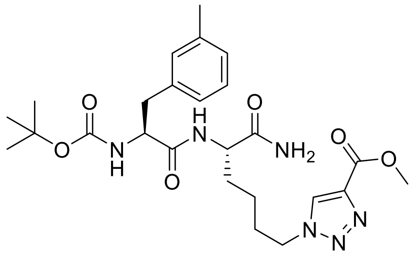

ESI (+):

m/z = 539.17 ([M+Na]$^+$)

M ($C_{25}H_{36}N_6NaO_6^+$, monoisotopic): 539.26

The synthesis was accomplished according to GP III. using 0.13 g **30c** (0.35 mmol, 1 eq.), 0.15 g Boc-(3-Me)Phe-OH (0.53 mmol, 1.5 eq.), 245 µL DiPEA (1.41 mmol, 4 eq.), 0.28 g PyBOP (0.53 mmol, 1.5 eq.) and 10 mL THF. The crude product was purified via preparative column chromatography (6% MeOH/CH_2Cl_2). *N*-(*tert*-Butyloxycarbonyl)-3-methyl-L-phenylalanyl-(4-(methoxy-carbonyl)-1*H*-1,2,3-triazol-1-yl)-L-norleucine amide (**31c**) was obtained as a white solid (0.15 g, 0.28 mmol, 55%; contained tris(pyrrolidin-1-yl)phosphine oxide, amount not quantified).

^{1}H NMR (400 MHz, CD_3CN) δ = 8.27 (s, 1H, CH aromatic triazol), 7.21 – 7.15 (m, 1H, H-5 aromatic Phe), 7.07 – 7.00 (m, 3H, H-2,4,6 aromatic Phe), 6.94 (d, ^3J = 7.7 Hz, 1H, NH), 6.30 (broad s, 1H, CONH*H*), 5.71 (broad s, 1H, CON*H*H), 5.60 (broad s, 1H, NH), 4.39 (t, ^3J = 7.1 Hz, 2H, $C_εH_2$), 4.19 (m, 2H, $C_α$H Phe, $C_α$H norleucine), 3.83 (s, 3H, OCH_3), 3.01 (m, 1H, $C_β$H*H* Phe), 2.81 – 2.71 (m, 1H, $C_β$*H*H Phe), 2.30 (s, 3H, CH_3), 1.97 – 1.76 (m, 3H, $C_β$H*H* Lys, $C_δH_2$), 1.67 – 1.54 (m, 1H, $C_β$*H*H norleucine), 1.34 (s, 9H, $(CH_3)_3$), 1.31 – 1.20 (m, 2H, $CγH_2$).

^{13}C NMR (101 MHz, CD_3CN) δ = 174.30 (CO), 172.56 (CO), 162.14 (CO), 156.64 (CO), 140.29, 138.98, 138.39, 130.95, 129.28, 129.10, 128.29, 127.23, 80.26 (*C*(CH_3)$_3$), 57.33 ($C_α$ norleucine), 53.40 ($C_α$ Phe), 52.41 (OCH_3), 50.92 ($C_ε$), 38.19 ($C_β$ Phe), 31.89 ($C_β$ norleucine), 30.11 ($C_δ$), 28.47 ((CH_3)$_3$), 23.01 ($C_γ$), 21.42 (CH_3).

3-Methyl-L-phenylalanyl-(4-(methoxycarbonyl)-1*H*-1,2,3-triazol-1-yl)-L-norvaline amide (32a)

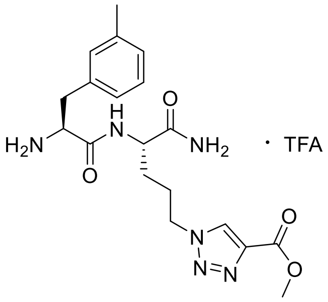

ESI (+):

m/z = 403.08 ($[M+H]^+$)

M ($C_{19}H_{27}N_6O_4^+$, monoisotopic): 403.21

The synthesis was accomplished according to GP II. using 0.13 g **31a** (0.26 mmol) and 10 mL TFA/CH_2Cl_2 (1:1). 3-Methyl-L-phenylalanyl-(4-(methoxycarbonyl)-1*H*-1,2,3-triazol-1-yl)-L-norvaline amide (**32a**) was obtained as a white solid (0.15 g, quantitative yield; contained TFA) without further identification.

3-Methyl-L-phenylalanyl-(4-(2-methoxy-2-oxoethyl)-1*H*-1,2,3-triazol-1-yl)-L-norvaline amide (32b)

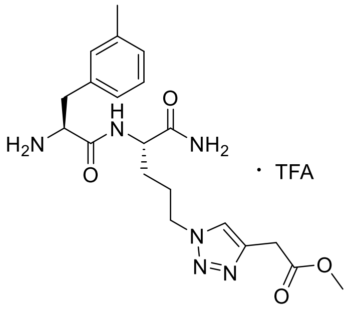

ESI (+):

m/z = 417.07 ($[M+H]^+$)

M ($C_{20}H_{29}N_6O_4^+$, monoisotopic): 417.22

The synthesis was accomplished according to GP II. using 0.05 g **31b** (0.10 mmol) and 10 mL TFA/CH_2Cl_2 (1:1). 3-Methyl-L-phenylalanyl-(4-(2-methoxy-2-oxoethyl)-1*H*-1,2,3-triazol-1-yl)-L-norvaline amide (**32b**) was obtained as a white solid (0.06 g, quantitative yield; contained TFA).

^{1}H NMR (400 MHz, DMSO-*d$_6$*) δ = 8.61 (d, ^3J = 8.2 Hz, 1H, NH), 8.07 (s, 3H, NH_3^+), 7.98 (s, 1H, CH triazol), 7.41 (s, 1H, CONH*H*), 7.22 – 7.14 (m, 2H, CON*H*H, H-5 Phe), 7.09 – 7.00 (m, 3H, aromatic Phe), 4.40 – 4.28 (m, 3H, $C_α$H Phe, $C_δH_2$), 4.11 – 4.03 (m, 1H, $C_α$H norvaline), 3.77 (s, 2H, CH_2COO), 3.63 (s, 3H, OCH_3), 3.07 – 2.97 (m, 1H, $C_β$H*H* Phe), 2.92 – 2.83 (m, 1H, $C_β$*H*H Phe), 2.28 (s, 3H, CH_3), 1.91 – 1.69 (m, 2H, $C_γH_2$), 1.69 – 1.47 (m, 2H, $C_βH_2$ norvaline).

^{13}C NMR (101 MHz, DMSO-d_6) δ = 172.31 (COONH$_2$), 170.53 (CO), 167.73 (COO), 139.71, 137.59, 134.65, 130.07, 128.42, 127.80, 126.52, 123.49, 53.23 (C$_\alpha$ norvaline), 51.87, 51.82, 48.97 (C$_\delta$H$_2$), 36.88 (C$_\beta$H$_2$ Phe), 31.05 (*C*H$_2$COO), 29.54 (C$_\beta$H$_2$ norvaline), 26.31 (C$_\gamma$H$_2$), 21.00 (CH$_3$).

3-Methyl-L-phenylalanyl-(4-(methoxycarbonyl)-1*H*-1,2,3-triazol-1-yl)-L-norleucine amide (32c)

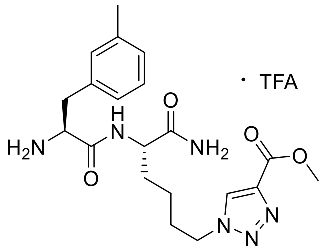

ESI (+):

m/z = 417.08 ([M+H]$^+$)

M (C$_{20}$H$_{29}$N$_6$O$_4$$^+$, monoisotopic): 417.22

The synthesis was accomplished according to GP II. using 0.14 g **31c** (0.27 mmol) and 10 mL TFA/CH$_2$Cl$_2$. 3-Methyl-L-phenylalanyl-(4-(methoxycarbonyl)-1*H*-1,2,3-triazol-1-yl)-L-norleucine amide (**32c**) was obtained as a yellow, wax-like solid (0.18 g, quantitative yield; contained TFA).

^{1}H NMR (400 MHz, DMSO-d_6) δ = 8.80 (s, 1H, NH triazol), 8.58 (d, ^3J = 8.2 Hz, 1H, NH), 8.07 (d, ^3J = 5.2 Hz, 3H, NH$_3^+$), 7.43 – 7.39 (m, 1H, CONH*H*), 7.24 – 7.17 (m, 1H, H-5 Phe), 7.14 – 7.01 (m, 4H, CON*H*H, aromatic Phe), 4.41 (t, ^3J = 7.1 Hz, 2H, C$_\varepsilon$H$_2$), 4.30 – 4.21 (m, 1H, C$_\alpha$H Phe), 4.09 – 4.01 (m, 1H, C$_\alpha$H norleucine), 3.83 (s, 3H, OCH$_3$), 3.14 – 3.05 (m, 1H, C$_\beta$H*H* Phe), 2.93 – 2.83 (m, 1H, C$_\beta$*H*H Phe), 2.28 (s, 3H, CH$_3$), 1.92 – 1.80 (m, 2H, C$_\delta$H$_2$), 1.70 – 1.52 (m, 2H, C$_\beta$H$_2$ norleucine), 1.31 – 1.20 (m, 2H, C$_\gamma$H$_2$).

^{13}C NMR (101 MHz, DMSO-d_6) δ = 172.54 (CONH$_2$), 167.69 (CO), 160.77 (COO), 138.55, 137.58, 134.67, 130.10, 128.94, 128.41, 127.79, 126.55, 53.24 (C$_\alpha$ norleucine), 52.12 (C$_\alpha$ Phe), 51.74 (OCH$_3$), 49.56 (C$_\varepsilon$), 36.89 (C$_\beta$ Phe), 31.72 (C$_\beta$ norleucine), 29.07 (C$_\delta$), 21.94 (C$_\gamma$), 21.01 (CH$_3$).

N-(4-Fluorobenzoyl)-3-methyl-L-phenylalanyl-(4-(methoxycarbonyl)-1*H*-1,2,3-triazol-1-yl)-L-norvaline amide (33a)

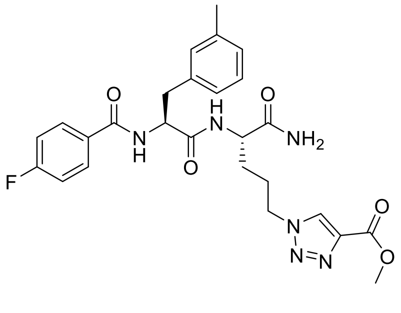

ESI (+):

m/z = 525.10 ([M+H]$^+$)

M (C$_{26}$H$_{30}$FN$_6$O$_5$$^+$, monoisotopic): 525.23

The synthesis was accomplished according to GP IV. using 0.06 g **32a** (0.12 mmol, 1 eq.), 14 µL 4-fluorobenzoyl chloride (0.12 mmol, 1 eq.), 49 µL TEA (0.35 mmol,

3 eq.) and 10 mL CH_2Cl_2. *N*-(4-Fluorobenzoyl)-3-methyl-L-phenylalanyl-(4-(methoxycarbonyl)-1*H*-1,2,3-triazol-1-yl)-L-norvaline amide (**33a**) was obtained as a white solid (0.05 g, 0.10 mmol, 87%).

^{1}H NMR (400 MHz, CD_3CN) δ = 8.27 (s, 1H, CH triazol), 7.84 – 7.72 (m, 2H, H-3,5 FBz), 7.25 (d, ^3J = 7.2 Hz, 1H, NH), 7.21 – 6.98 (m, 7H, H-2,6 FBz, H-2,4,5,6 aromatic Phe, NH), 6.20 (s, 1H, CONH*H*), 5.71 (s, 1H, CON*H*H), 4.67 – 4.61 (m, 1H, C_αH Phe), 4.38 (t, ^3J = 7.0 Hz, 2H, $C_\delta H_2$), 4.32 – 4.21 (m, 1H, C_αH norvaline), 3.85 (s, 3H, $COOCH_3$), 3.18 (dd, ^2J = 13.9, ^3J = 5.9 Hz, 1H, C_βH*H* Phe), 3.02 (dd, ^2J = 13.9, ^3J = 8.8 Hz, 1H, C_β*H*H Phe), 2.27 (s, 3H, CH_3), 1.91 – 1.75 (m, 3H, $C_\gamma H_2$, *C*$_\beta$*H*H norvaline), 1.62 – 1.49 (m, 1H, C_βH*H* norvaline).

^{13}C NMR (101 MHz, CD_3CN) δ = 173.93 ($CONH_2$), 172.15 (CO), 167.30 (CO), 165.66 (d, $^1J_{C,F}$ = 249.5 Hz, CF), 162.11 (COO), 140.27, 139.07, 138.41, 131.28 (d, $^4J_{C,F}$ = 3.1 Hz, C-1 FBz), 130.96, 130.80 (d, $^3J_{C,F}$ = 9.2 Hz, C-2/6 FBz), 129.34, 129.14, 128.34, 127.23, 116.28 (d, $^2J_{C,F}$ = 22.1 Hz, C-3/5 FBz), 56.67 (C_α Phe), 53.14 (C_α Orn), 52.43 (OCH_3), 50.68 (C_δ), 37.81 (C_β Phe), 29.36 (C_β Orn), 27.02 (C_γ), 21.37 (CH_3).

N-(4-Fluorobenzoyl)-3-methyl-L-phenylalanyl-(4-(2-methoxy-2-oxoethyl)-1*H*-1,2,3-triazol-1-yl)-L-norvaline amide (33b)

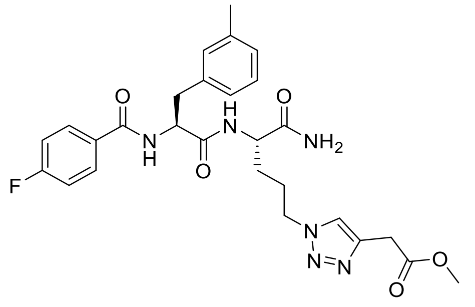

ESI (+):

m/z = 539.08 ($[M+H]^+$)

M ($C_{27}H_{32}FN_6O_5^+$, monoisotopic): 539.24

The synthesis was accomplished according to GP IV. using 0.05 g **32b** (0.09 mmol, 1 eq.), 11 µL 4-fluorobenzoyl chloride (0.09 mmol, 1 eq.), 39 µL TEA (0.28 mmol, 3 eq.) and 10 mL CH_2Cl_2. *N*-(4-Fluorobenzoyl)-3-methyl-L-phenylalanyl-(4-(2-methoxy-2-oxoethyl)-1*H*-1,2,3-triazol-1-yl)-L-norvaline amide (**33b**) was obtained as a white solid (0.03 g, 0.06 mmol, 67%).

^{1}H NMR (400 MHz, DMSO-*d6*) δ = 8.61 (d, ^3J = 8.1 Hz, 1H, NH Phe), 8.12 (d, ^3J = 8.2 Hz, 1H, NH norvaline), 7.95 (s, 1H, CH triazol), 7.84 (dd, $^3J_{H,H}$ = 8.9 Hz, $^4J_{H,F}$ = 5.5 Hz, 2H, H-2,6 FBz), 7.35 – 7.21 (m, 3H, H-3,5 FBz, H-5 Phe), 7.19 – 7.04 (m, 4H, H-2,6 FBz, $CONH_2$), 7.00 – 6.94 (m, 1H, H-4 Phe), 4.70 – 4.62 (m, 1H, C_αH Phe), 4.34 (t, ^3J = 7.1 Hz, 2H, $C_\delta H_2$), 4.30 – 4.21 (m, 1H, C_αH norvaline), 3.74 (s, 2H, CH_2COO), 3.62 (s, 3H, OCH_3), 3.16 – 2.82 (m, 2H, $C_\beta H_2$ Phe), 2.24 (s, 3H, CH_3), 1.87 – 1.62 (m, 3H, $C_\gamma H_2$, C_βH*H* norvaline), 1.59 – 1.47 (m, 1H, C_β*H*H norvaline).

^{13}C NMR (101 MHz, DMSO-*d6*) δ = 173.00 ($CONH_2$), 171.26 (CO), 170.47 (COO), 165.40 (CO), 163.90 (d, $^1J_{C,F}$ = 248.6 Hz, CF), 139.68, 138.21, 137.00, 130.48 (d, $^4J_{C,F}$ = 2.9 Hz, C-1 FBz), 130.02 (d, $^3J_{C,F}$ = 9.0 Hz, C-2/6 FBz), 129.79, 127.92, 126.88,

126.22, 123.34, 115.12 (d, $^{2}J_{C,F}$ = 21.8 Hz, C-3/5 FBz), 55.19 ($C_α$ Phe), 51.78 (OCH_3), 51.75 ($C_α$ norvaline), 48.99 ($C_δ$), 36.79 ($C_β$ Phe), 31.06 (CH_2CO), 29.15 ($C_β$ norvaline), 26.32 ($C_γ$), 21.00 (CH_3).

N-(4-Fluorobenzoyl)-3-methyl-L-phenylalanyl-(4-(methoxycarbonyl)-1H-1,2,3-triazol-1-yl)-L-norleucine amide (33c)

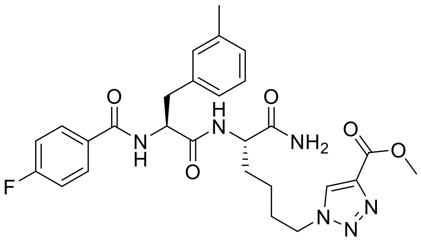

ESI (+):

m/z = 539.13 ($[M+H]^+$)

M ($C_{27}H_{32}FN_6O_5^+$, monoisotopic): 539.24

The synthesis was accomplished according to GP IV. using 0.13 g **32c** (0.11 mmol, 1 eq.), 16 µL 4-fluorobenzoyl chloride (0.16 mmol, 1.5 eq.), 45 µL TEA (0.32 mmol, 3 eq.) and 10 mL CH_2Cl_2. *N*-(4-Fluorobenzoyl)-3-methyl-L-phenylalanyl-(4-(methoxycarbonyl)-1*H*-1,2,3-triazol-1-yl)-L-norleucine amide (**33c**) was obtained as a yellowish solid (0.01 g, 0.15 mmol, 59%).

^{1}H NMR (400 MHz, DMSO-*d6*) δ = 8.77 (s, 1H, CH triazol), 8.59 (d, ^{3}J = 8.2 Hz, 1H, NH), 8.07 – 7.97 (m, 1H, NH), 7.89 – 7.81 (m, 2H, H-2,6 FBz), 7.38 – 7.25 (m, 3H, NH, H-3,5 FBz), 7.22 – 7.10 (m, 3H, aromatic Phe), 7.03 (s, 1H, NH), 6.99 – 6.93 (m, 1H, aromatic Phe), 4.70 – 4.58 (m, 1H, $C_αH$ Phe), 4.39 (t, ^{3}J = 7.1 Hz, 2H, $C_εH_2$), 4.26 – 4.16 (m, 1H, $C_αH$ norleucine), 3.79 (s, 3H, OCH_3), 3.12 – 3.04 (m, 1H, $C_β$*H*H Phe), 2.96 – 2.86 (m, 1H, $C_β$H*H* Phe), 2.24 (s, 3H, CH_3), 1.92 – 1.82 (m, 2H, $C_δH_2$), 1.72 – 1.67 (m, 1H, $C_β$H*H* norleucine), 1.65 – 1.53 (m, 1H, $C_β$*H*H norleucine), 1.32 – 1.21 (m, 2H, $C_γH_2$).

^{13}C NMR (101 MHz, DMSO-*d6*) δ = 173.24 ($CONH_2$), 171.15 (CO), 165.33 (CO), 163.89 (d, $^{1}J_{C,F}$ = 248.5 Hz, CF), 160.74 (COO), 138.50, 138.23, 136.98, 130.50 (d, $^{4}J_{C,F}$ = 2.8 Hz, C-1 FBz), 130.01 (d, $^{3}J_{C,F}$ = 9.0 Hz, C-2/6 FBz), 129.78, 128.89, 127.90, 126.86, 126.21, 115.12 (d, $^{2}J_{C,F}$ = 21.7 Hz, C-3/5 FBz), 55.21 ($C_α$ Phe), 51.99 ($C_α$ norleucine), 51.65 (OCH_3), 49.62 ($C_ε$), 36.85 ($C_β$ Phe), 31.43 ($C_β$ norleucine), 29.05 ($C_δ$), 22.02 ($C_γ$), 20.99 (CH_3).

N-(4-Fluorobenzoyl)-3-methyl-L-phenylalanyl-(4-(methoxycarbonyl)-1H-1,2,3-triazol-1-yl)-L-norvaline nitrile (34a)

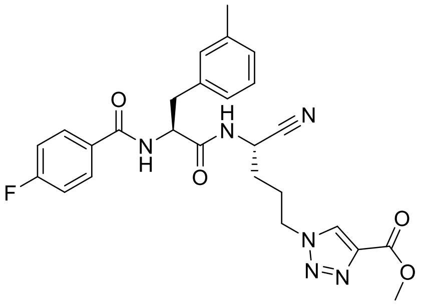

ESI (+):

m/z = 507.64 ($[M+H]^+$)

M ($C_{26}H_{28}FN_6O_4^+$, monoisotopic): 507.22

The synthesis was accomplished according to GP V. using 0.05 g **33a** (0.09 mmol, 1 eq.), 0.03 g cyanuric chloride (0.18 mmol, 2 eq.) and 10 mL DMF. *N*-(4-Fluorobenzoyl)-3-methyl-L-phenylalanyl-(4-(methoxycarbonyl)-1*H*-1,2,3-triazol-1-yl)-L-norvaline nitrile (**34a**) was obtained as a yellow oil (0.04 g, 0.08 mmol, 89%).

^{1}H NMR (400 MHz, CD_3CN) δ = 8.29 (s, 1H, CH triazol), 7.81 – 7.74 (m, 2H, H-3,5 FBz), 7.27 – 7.20 (m, 2H, 2 NH), 7.20 – 7.13 (m, 3H, H-2,6 FBz, H-5 aromatic Phe), 7.11 – 6.99 (m, 3H, H-2,4,6 aromatic Phe), 4.74 (m, 1H, $C_{\alpha}H$ norvaline), 4.70 – 4.59 (m, 1H, $C_{\alpha}H$ Phe), 4.41 (t, ^3J = 6.9 Hz, 2H, $C_{\delta}H_2$), 3.86 (s, 3H, $COOCH_3$), 3.19 (dd, ^2J = 13.8, ^3J = 5.9 Hz, 1H, $C_{\beta}HH$ Phe), 3.01 (dd, ^2J = 13.9, ^3J = 8.7 Hz, 1H, $C_{\beta}HH$ Phe), 2.27 (s, 3H, CH_3), 2.04 – 1.91 (m, 2H, $C_{\gamma}H_2$), 1.87 – 1.75 (m, 2H, $C_{\beta}H_2$ norvaline).

^{13}C NMR (101 MHz, CD_3CN) δ = 172.09 (CO), 166.94 (CO), 165.65 (d, $^1J_{C,F}$ = 249.6 Hz, CF), 162.08 (COO), 140.35, 139.04, 138.19, 131.27 (d, $^4J_{C,F}$ = 3.0 Hz, C-1 FBz), 130.98, 130.80 (d, $^3J_{C,F}$ = 9.1 Hz, C-2/6 FBz), 129.31, 129,25, 128.34, 127.24, 119.36 (CN), 116.26 (d, $^2J_{C,F}$ = 22.2 Hz, C-3/5 FBz), 56.21 (C_{α} Phe), 52.46 (OCH_3), 50.16 (C_{δ}), 40.70 (C_{α} norvaline), 37.87 (C_{β} Phe), 29.87 (C_{β} norvaline), 26.64 (C_{γ}), 21.37 (CH_3).

N-(4-Fluorobenzoyl)-3-methyl-L-phenylalanyl-(4-(2-methoxy-2-oxoethyl)-1H-1,2,3-triazol-1-yl)-L-norvaline nitrile (34b)

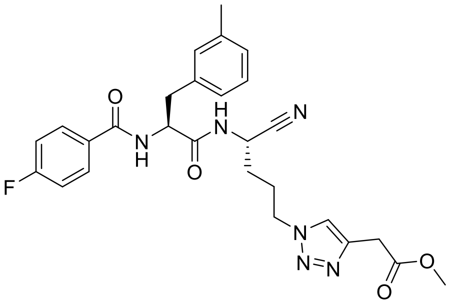

HR-MS ESI (+):

m/z = 521.2307 ($[M+H]^+$)

M ($C_{27}H_{30}FN_6O_4^+$, monoisotopic): 521.2303

The synthesis was accomplished according to GP V. using 0.03 g **33b** (0.06 mmol, 1 eq.), 0.04 g cyanuric chloride (0.22 mmol, 3.7 eq.) and 10 mL DMF. *N*-(4-Fluorobenzoyl)-3-methyl-L-phenylalanyl-(4-(2-methoxy-2-oxoethyl)-1*H*-1,2,3-triazol-1-yl)-L-norvaline nitrile (**34b**) was obtained as a yellow oil (0.03 g, quantitative yield; contained residual solvent).

^{1}H NMR (400 MHz, $CDCl_3$) δ = 7.74 – 7.68 (m, 2H, H-2,6 FBz), 7.59 (s, 1H, CH triazol), 7.23 – 6.92 (m, 8H, H-3,5 FBz, aromatic Phe, 2 NH), 4.85 – 4.76 (m, 2H, $C_αH$ Phe, $C_αH$ norvaline), 4.32 (t, ^3J = 6.8, 1.4 Hz, 2H, $C_δH_2$), 3.80 (s, 2H, CH_2COO), 3.73 (s, 3H, OCH_3), 3.17 – 3.12 (m, 2H, $C_βH_2$ Phe), 2.29 (s, 3H, CH_3), 2.05 – 1.94 (m, 2H, $C_βH_2$ norvaline), 1.87 – 1.70 (m, 2H, $C_γH_2$).

^{13}C NMR (101 MHz, $CDCl_3$) δ = 171.19 (CO), 171.03 (CO), 166.78 (CO), 165.22 (d, $^1J_{C,F}$ = 253.1 Hz, CF), 140.92, 138.85, 135.93, 130.22, 129.74, 129.65, 128.97, 128.29, 126.40, 122.99, 117.61, 115.94 (d, $^2J_{C,F}$ = 22.0 Hz, C-3/5 FBz), 55.15 ($C_α$), 52.49 (OCH_3), 49.05 ($C_δ$), 39.85 ($C_α$), 38.16 ($C_β$ Phe), 31.62 (*C*H_2CO), 29.65 ($C_γ$), 25.97 ($C_β$ norvaline), 21.47 (CH_3).

N-(4-Fluorobenzoyl)-3-methyl-L-phenylalanyl-(4-(methoxycarbonyl)-1*H*-1,2,3-triazol-1-yl)-L-norleucine nitrile (34c)

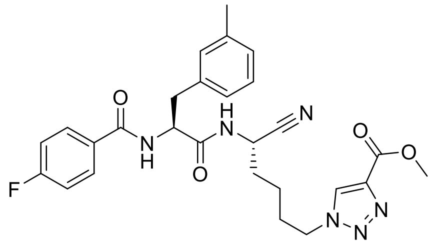

HR-MS ESI (+):

m/z = 543.2125 ($[M+Na]^+$)

M ($C_{27}H_{29}FN_6NaO_4^+$, monoisotopic): 543.2126

The synthesis was accomplished according to GP V. using 0.07 g **33c** (0.13 mmol, 1 eq.), 0.05 g cyanuric chloride (0.27 mmol, 2 eq.) and 10 mL DMF. *N*-(4-Fluorobenzoyl)-3-methyl-L-phenylalanyl-(4-(methoxycarbonyl)-1*H*-1,2,3-triazol-1-yl)-L-norleucine nitrile (**34c**) was obtained as a yellow oil (0.06 g, 0.11 mmol, 85%).

^{1}H NMR (400 MHz, CD_3CN) δ = 8.27 (s, 1H, CH triazol), 7.83 – 7.77 (m, 2H, H-2,6 FBz), 7.28 (d, ^3J = 8.0 Hz, 1H, NH), 7.23 – 7.02 (m, 7H, NH, H-3,5 FBz, aromatic Phe), 4.73 – 4.64 (m, 2H, $C_αH$ Phe, $C_αH$ norleucine), 4.39 (t, ^3J = 7.0 Hz, 2H, $C_εH_2$), 3.84 (s, 3H, OCH_3), 3.12 – 3.07 (m, 2H, $C_βH_2$ Phe), 2.27 (s, 3H, CH_3), 1.88 – 1.73 (m, 4H, $C_βH_2$ norleucine, $C_δH_2$), 1.43 – 1.33 (m, 2H, $C_γH_2$).

^{13}C NMR (101 MHz, CD_3CN) δ = 172.05 (CO), 166.89 (CO), 163.26 (d, $^1J_{C,F}$ = 231.0 Hz, CF), 163.23 (COO), 140.31, 139.02, 138.26, 131.32 (d, $^4J_{C,F}$ = 3.1 Hz, C-1 FBz) 131.00, 130.83 (d, $^3J_{C,F}$ = 9.1 Hz, C-2/6 FBz), 129.30, 129.16, 128.32, 127.25, 119.67, 116.25 (d, $^2J_{C,F}$ = 22.1 Hz, C-3/5 FBz), 56.15 ($C_α$), 52.42 (OCH_3), 50.75 ($C_ε$), 40.99 ($C_β$ Phe), 38.04 ($C_α$ norleucine), 32.10 ($C_β$ norleucine), 29.66 ($C_δ$), 22.81 ($C_γ$), 21.36 (CH_3).

N-(4-Fluorobenzoyl)-3-methyl-L-phenylalanyl-(4-carboxy)-1H-1,2,3-triazol-1-yl)-L-norvaline nitrile (35a)

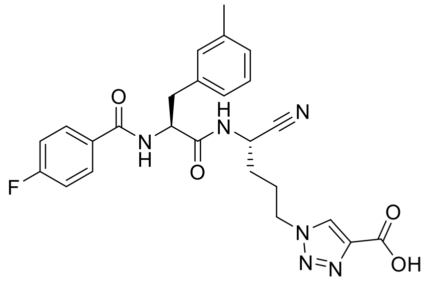

HR-MS ESI (+):

m/z = 515.1813 ($[M+Na]^+$)

M ($C_{25}H_{25}FN_6NaO_4^+$, monoisotopic): 515.181

34a (0.04 g, 0.08 mmol, 1 eq.) was dissolved in THF/MeOH (3:1, 8 mL) and 1M NaOH (234 µL, 0.24 mmol, 3 eq.) added dropwise. The solution was stirred over night and neutralized with 1M HCl. The solvent was evaporated and the crude product purified *via* semipreparative HPLC. *N*-(4-Fluorobenzoyl)-3-methyl-L-phenylalanyl-(4-carboxy)-1*H*-1,2,3-triazol-1-yl)-L-norvaline nitrile (**35a**) was obtained as a white solid (7 mg, 0.01 mmol, 17%).

^{1}H NMR (400 MHz, CD_3CN) δ = 8.30 (s, 1H, CH triazol), 7.80 – 7.71 (m, 2H, H-3,5 FBz), 7.23 – 7.14 (m, 5H, H-2,6 FBz, H-5 aromatic Phe, 2 NH), 7.12 – 6.99 (m, 3H, H-2,4,6 aromatic Phe), 4.79 – 4.71 (m, 1H, C_αH norvaline), 4.68 – 4.61 (m, 1H, C_αH Phe), 4.41 (t, ^3J = 6.9 Hz, 2H, $C_\delta H_2$), 3.19 (dd, ^2J = 13.8, ^3J = 5.9 Hz, 1H, C_βH*H* Phe), 3.01 (dd, ^2J = 13.8, ^3J = 8.7 Hz, 1H, C_β*H*H Phe), 2.27 (s, 3H, CH_3), 1.97 – 1.92 (m, 2H, $C_\gamma H_2$), 1.90 – 1.72 (m, 2H, $C_\beta H_2$ norvaline).

The carboxyl proton was not detectable in the chosen solvent.

^{13}C NMR (101 MHz, CD_3CN) δ = 172.08 (CO), 166.98 (CO), 165.66 (d, $^1J_{C,F}$ = 249.5 Hz, CF), 161.79 (COO), 140.23, 139.05, 138.17, 131.25 (d, $^4J_{C,F}$ = 3.0 Hz, C-1 FBz), 130.98, 130.81 (d, $^3J_{C,F}$ = 9.1 Hz, C-2/6 FBz), 129.48, 129.32, 128.34, 127.24, 119.35 (CN), 116.27 (d, $^2J_{C,F}$ = 22.1 Hz, C-3/5), 56.19 (C_α Phe), 50.15 (C_δ), 40.68 (C_α norvaline), 37.85 (C_β Phe), 29.86 (C_β norvaline), 26.61 (C_γ), 21.35 (CH_3).

N-(4-Fluorobenzoyl)-3-methyl-L-phenylalanyl-(4-carboxyethyl)-1H-1,2,3-triazol-1-yl)-L-norvaline nitrile (35b)

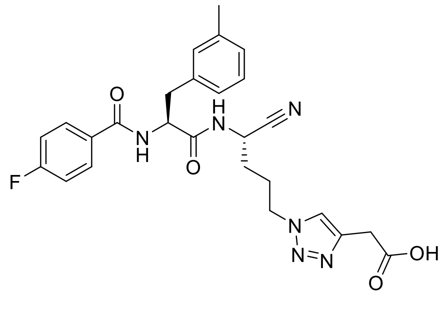

HR-MS ESI (+):

m/z = 507.5151 ($[M+H]^+$)

M ($C_{26}H_{28}FN_6O_4^+$, monoisotopic): 507.2151

34b (0.02 g, 0.03 mmol, 1 eq.) was dissolved in acetone (0.5 mL) and added swiftly to a solution of esterase from porcine liver (20 mg) in potassium dihydrogen phosphate

(0.2 M, pH 7.0, 5 mL). After 48 h, further 30 mg esterase from porcine liver were added. The reaction progress was monitored *via* HPLC. After the reaction was completed, the solution was acidified to pH 4.0 with 1 M HCl. A white precipitate formed. The suspension was diluted with H_2O (20 mL) and extracted with ethyl acetate (4 × 10 mL). The combined organic layers were washed with brine (5 mL) and dried over Na_2SO_4. The solvent was evaporated. The crude product was purified *via* semipreparative HPLC to yield *N*-(4-fluorobenzoyl)-3-methyl-L-phenylalanyl-(4-carboxyethyl)-1*H*-1,2,3-triazol-1-yl)-L-norvaline nitrile (**35b**) was obtained as a white solid (4 mg, 0.01 mmol, 29%).

^{1}H NMR (400 MHz, CD_3CN) δ = 7.82 – 7.74 (m, 2H, H-2,6 FBz), 7.73 (s, 1H, CH triazol), 7.25 – 7.13 (m, 5H, H-3,5 FBz, aromatic Phe, 2 NH), 7.10 – 7.00 (m, 3H, aromatic Phe), 4.78 – 4.70 (m, 1H, $C_\alpha H$ norvaline), 4.70 – 4.62 (m, 1H, $C_\alpha H$ Phe), 4.35 (t, ^3J = 6.8 Hz, 2H, $C_\delta H_2$ norvaline), 3.73 (s, 2H, CH_2COO), 3.23 – 3.16 (m, 1H, $C_\beta HH$ Phe), 3.05 – 2.97 (m, 1H, $C_\beta HH$ Phe), 2.27 (s, 3H, CH_3), 1.98 – 1.96 (m, 2H, $C_\beta H_2$), 1.83 – 1.75 (m, 2H, $C_\gamma H_2$).

The carboxyl proton was not detectable in the chosen solvent.

NMR (101 MHz, CD_3CN) δ = 172.07 (COO), 171.70 (CO), 167.03 (CO), 165.66 (d, $^1J_{C,F}$ = 249.5 Hz, CF), 141.47, 139.05, 138.15, 131.25 (d, $^4J_{C,F}$ = 2.9 Hz, C-1 FBz), 130.98, 130.82 (d, $^3J_{C,F}$ = 9.1 Hz, C-2/6 FBz), 129.32, 128.35, 127.24, 124.30 (CH triazol), 119.36, 116.27 (d, $^2J_{C,F}$ = 22.1 Hz, C-3/5), 56.15 (C_α Phe), 49.89 (C_δ), 40.77 (C_α norvaline), 37.91 (C_β Phe), 31.74 (*C*H_2COO), 29.95 (C_γ), 26.79 (C_β norvaline), 21.36 (CH_3).

_N_-(4-Fluorobenzoyl)-3-methyl-L-phenylalanyl-(4-carboxy-1_H_-1,2,3-triazol-1-yl)-L-norleucine nitrile (35c)

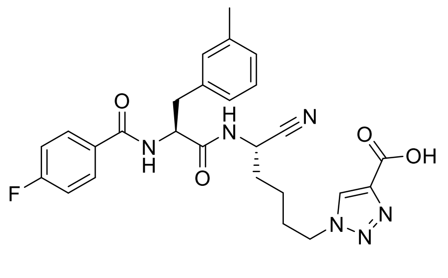

HR-MS ESI (+):

m/z = 507.2150 ([M+Na]$^+$)

M ($C_{26}H_{28}FN_6O_4^+$, monoisotopic): 507,.2151

34b (0.01 g, 0.02 mmol, 1 eq.) was dissolved in acetone (1 mL) and swiftly added to a solution of esterase from porcine liver (11 mg) in potassium dihydrogen phosphate (0.2 M, pH 7.0, 5 mL). Further esterase from porcine liver was added after 24 h (10 mg) and after 72 h (50 mg). The reaction progress was monitored *via* HPLC. After the reaction was completed, the solution was acidified to pH 4.0 using 1 M HCl. A white precipitate formed. The suspension was diluted with H_2O (10 mL) and extracted with ethyl acetate (4 × 10 mL). The combined organic layers were washed with brine (5 mL) and dried over Na_2SO_4. The solvent was evaporated. The crude product was purified via semipreparative HPLC to yield *N*-(4-fluorobenzoyl)-3-methyl-L-phenylalanyl-(4-

carboxy-1*H*-1,2,3-triazol-1-yl)-L-norleucine nitrile (**35c**) was obtained as a white solid (4 mg, 0.01 mmol, 33%).

^{1}H NMR (400 MHz, CD_3CN) δ = 8.27 (s, 1H, CH triazol), 7.81 – 7.75 (m, 2H, H-2,6 FBz), 7.26 – 7.14 (m, 5H, 2 NH, H-3,5 FBz, H-5 Phe), 7.10 – 7.00 (m, 3H, H-2,4,6 Phe), 4.73 – 4.63 (m, 2H, $C_\alpha H$ Phe, $C_\alpha H$ norleucine), 4.38 (t, ^3J = 7.0 Hz, 2H, $C_\varepsilon H_2$), 3.24 – 3.16 (m, 1H, $C_\beta H\textit{H}$ Phe), 3.02 – 2.93 (m, 1H, $C_\beta \textit{H}H$ Phe), 2.28 (s, 3H, CH_3), 1.93 – 1.78 (m, 4H, $C_\beta H_2$ norleucine, $C_\delta H_2$), 1.37 (p, ^3J = 7.9 Hz, 2H, $C_\gamma H_2$).

The carboxyl proton was not detectable in the chosen solvent.

^{13}C NMR (101 MHz, CD_3CN) δ = 172.00 (CO), 166.96 (CO), 165.64 (d, $^1J_{C,F}$ = 249.5 Hz, CF), 161.80 (COO), 140.13, 139.02, 138.19, 131.27 (d, $^4J_{C,F}$ = 3.0 Hz, C-1 FBz), 130.99, 130.81, (d, $^3J_{C,F}$ = 9.1 Hz, C-2/6 FBz), 129.39 (CH triazol), 129.30, 128.32, 127.24, 119.62 (CN), 116.26 (d, $^2J_{C,F}$ = 22.1 Hz, C-3/5), 56.08 (C_α), 50.73 (C_ε), 40.97 (C_α), 37.96 (C_β Phe), 32.04 (C_δ), 29.57 (C_β norleucine), 22.74 (C_γ), 21.35 (CH_3).

5 Synthesis of Serine-based Dipeptide Nitrile 43 with Carboxy-functionalized 1,2,3-Triazolyl Residue in P2 (Scheme 5)

***N*-(*tert*-Butyloxycarbonyl)-*O*-((1-(methylcarboxymethyl)-1*H*-1,2,3-triazol-4-yl)-methyl)-L-serine (36)**

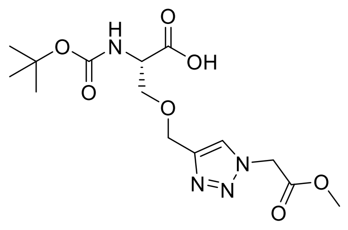

ESI (+):

m/z = 358.12 ($[M+H]^+$)

M ($C_{14}H_{23}N_4O_7^+$, monoisotopic): 358.15

Boc-L-Ser-(Propargyl)-OH (0.85 g; 0.21 mmol; 1 eq.), methyl-2-azidoacetate (25 mg; 0.21 mmol; 1 eq.), $CuSO_4$×5 H_2O (26 mg; 0.10 mmol; 0.5 eq.) and sodium ascorbate (41 mg; 0.21 mmol; 1 eq.) were dissolved in DMSO/ H_2O (1:2, 15 mL) at 0°C under argon atmosphere. The solution was stirred over night before adding 2 M HCl (20 mL). The product was extracted with ethyl acetate (4 × 10 mL). The combined organic layers were washed with brine (2 × 5 mL), dried over Na_2SO_4 and the solvent evaporated. *(S)*-*N*-(*tert*-Butyloxycarbonyl)-*O*-((1-(methylcarboxy-methyl)-1*H*-1,2,3-triazol-4-yl)-methyl)serine (**36**) was obtained as a clear oil (0.05 g, 0.13 mmol, 61%).

^{1}H NMR (600 MHz, $CDCl_3$) δ = 7.70 (s, 1H, CH triazol), 5.47 (s, 1H, NH), 5.27 - 5,13 (m, 2H, N-CH_2), 4.69 (dd, 2H, OCH_2), 4.42 (d, ^2J = 10.5 Hz, 1H, $C_\alpha H$), 4.00 - 3.94 (m, 1H, HC-CH*H*), 3.91 - 3.79 (m, 4H, HC-C*H*H, OCH_3), 1.44 (s, 9H, $(CH_3)_3$).

The carboxyl proton was not detectable in the chosen solvent.

^{13}C NMR (151 MHz, $CDCl_3$) δ = 173.30 (COOH), 167.20 (OCH_3), 155.91 (CO Boc), 145.10 (C-4 triazol), 124.44 (C-5 triazol), 80.47 (*C*$(CH_3)_3$), 70.06 (CH-*C*H_2-O), 64.59 (*C*H_2-C=C), 53.96 (C_α), 53.42 (N-CH_2), 50.94 (COO-*C*H_3), 28.45 ($(CH_3)_3$).

(S)-N-(*tert*-Butyloxycarbonyl)-O-((1-(methylcarboxymethyl)-1*H*-1,2,3-triazol-4-yl)-methyl)-L-serinamide (37)

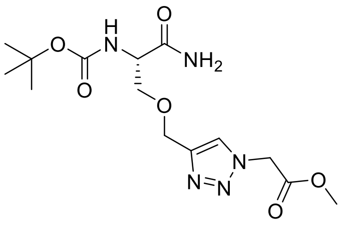

ESI (+):

m/z = 380.05 ($[M+H]^+$)

M ($C_{14}H_{23}N_5NaO_6^+$, monoisotopic): 380.15

The synthesis was accomplished according to GP I. using 0.18 g **36** (0.51 mmol, 1 eq.), 194 µL NMM (1.52 mmol, 3 eq.), 65 µL iBCF (0.56 mmol, 1.1 eq.), 189 µL NH_3 (25%, 2.55 mmol, 5 eq.) and 15 mL THF. *(S)-N*-(*tert*-Butyloxycarbonyl)-*O*-((1-(methylcarboxy-methyl)-1*H*-1,2,3-triazol-4-yl)-methyl)-L-serinamide (**37**) was obtained as a clear oil (0.13 g, 0.36 mmol, 64%).

^{1}H NMR (400 MHz, $CDCl_3$) δ = 7.70 (s, 1H, CH triazol), 6.53 (s, 1H, NH), 5.50 (s, 2H, $CONH_2$), 5.18 (s, 2H, N-CH_2), 4.73 (s, 2H, OCH_2), 4.28 (s, 1H, C_αH), 3.96 (dd, ^2J = 9.4, ^3J = 3.8 Hz, 1H, C_βH*H*), 3.82 (s, 3H, OCH_3), 3.63 (dd, ^2J = 9.4, ^3J = 6.7 Hz, 1H, C_β*H*H,), 1.44 (s, 9H, $(CH_3)_3$).

^{13}C NMR (101 MHz, $CDCl_3$) δ = 172.66 ($CONH_2$), 166.80 (*C*$OOCH_3$), 155.91 (CO-Boc), 144.94 (C-4 triazol), 125.66 (C-5 triazol), 80.46 (*C*$(CH_3)_3$), 70.13 (C_β Ser), 64.62 (OCH_2), 53.66 (C_α Ser), 53.27 (N-CH_2), 50.86 (COO-*C*H_3), 28.45 ((*C*$H_3)_3$).

O-((1-(Methylcarboxymethyl)-1*H*-1,2,3-triazol-4-yl)-methyl)-L-serinamide (38)

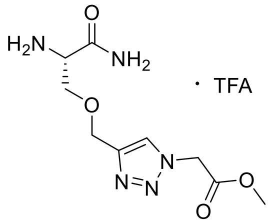

ESI (+):

m/z = 258.19 ($[M+H]^+$)

M ($C_9H_{16}N_5O_4^+$, monoisotopic): 258.12

The synthesis was accomplished according to GP II. using 0.17 g **37** (0.47 mmol) and 10 mL TFA/CH_2Cl_2 (1:1). *O*-((1-(Methylcarboxymethyl)-1*H*-1,2,3-triazol-4-yl)-methyl)-L-serinamide (**38**) was obtained as a greenish oil (0.18 g, quantitative yield; contained TFA).

^{1}H NMR (400 MHz, DMSO-d_6, 30°C) δ = 8.13 (broad s, 3H, NH_3^+), 8.11 (s, 1H, CH triazol), 7.82 (s, 1H, N*H*H), 7.58 (s, 1H, NH*H*), 5.28 (s, 2H, N-CH_2), 4.62 (s, 2H, OCH_2), 3.94 (s, 1H, C_αH), 3.84 - 3.72 (m, 2H, $C_\beta H_2$).

Due to overlapping with the solvent signal, the methyl proton signal was not detectable.

^{13}C NMR (101 MHz, DMSO-d_6, 30°C) δ = 168.40 ($CONH_2$), 167.83 ($COOCH_3$), 142.99 (C-4 triazol), 125.52 (C-5 triazol), 68.28 ($C_\beta H_2$), 63.85 (OCH_2), 52.66 (CH-$CONH_2$), 52.36 (N-CH_2), 50.42 (N-CH_2).

***N*-(*tert*-Butyloxycarbonyl)-3-methyl-L-phenylalanyl-*O*-((1-(methylcarboxymethyl)-1*H*-1,2,3-triazol-4-yl)-methyl)-L-serinamide (39)**

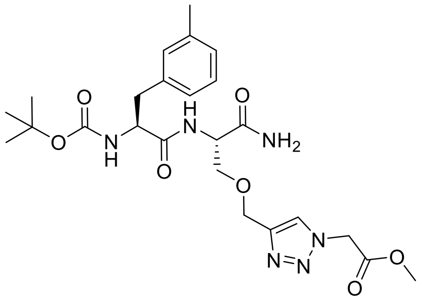

ESI (+):

m/z = 519.05 ($[M+H]^+$)

M ($C_{24}H_{35}N_6O_7^+$, monoisotopic): 519.26

The synthesis was accomplished according to GP III. using 0.17 g **38** (0.47 mmol, 1 eq.), 0.20 g Boc-(3-Me)Phe-OH (0.71 mmol, 1.5 eq.), 325 µL DiPEA (1.88 mmol, 4 eq.), 0.37 g PyBOP (0.71 mmol, 1.5 eq.) and 25 mL THF. *N*-(*tert*-Butyloxycarbonyl)-3-methyl-L-phenylalanyl-*O*-((1-(methylcarboxy-methyl)-1*H*-1,2,3-triazol-4-yl)-methyl)-L-serinemide (**39**) was obtained as a clear solid (0.22 g, 0.42 mmol, 89%).

^{1}H NMR (400 MHz, DMSO-d_6) δ = 8.08 (s, 1H, CH triazol), 7.94 (d, ^3J = 7.8 Hz, 1H, NH), 7.32 (s, 1H, NH), 7.18 - 7.11 (m, 1H, aromatic), 7.10 - 7.03 (m, 2H, aromatic), 7.03 - 6.94 (m, 3H, aromatic, $CONH_2$), 5.40 (s, 2H, CH_2COO), 4.58 (d, ^3J = 0.8 Hz, 2H, OCH_2), 4.46 - 4.38 (m, 1H, $C_\alpha H$ Ser), 4.21 - 4.13 (m, 1H, $C_\alpha H$ Phe), 3.70 (s, 3H, $COOCH_3$), 3.04 - 2.99 (m, 2H, $C_\beta H_2$ Ser), 2.98 - 2,92 (m, 1H, $C_\beta H$H Phe), 2.73 - 2.64 (m, 1H, $C_\beta HH$ Phe), 2.26 (s, 3H, Ar-CH_3), 1.33 - 1.26 (m, 9H, $(CH_3)_3$).

^{13}C NMR (101 MHz, DMSO-d_6) δ = 171.54, 171.11, 167.66, 155.27, 143.75 (C-4 triazol), 138.03, 136.87, 129.78, 127.86, 126.77, 125.36 (C-5 triazol), 78.16 ($C(CH_3)_3$), 69.94 (C_β Ser), 63.66 (OCH_2-triazol), 55.81 (C_α Ser), 52.48 (COO-CH_3), 52.43 (C_α Phe), 50.20 (CH_2COO) 45,28, 37.09 (C_β Phe), 28.09 ($(CH_3)_3$), 21.01 (CH_3).

3-Methyl-L-phenylalanyl-*O*-((1-(methylcarboxymethyl)-1*H*-1,2,3-triazol-4-yl)-methyl)-L-serinamide (40)

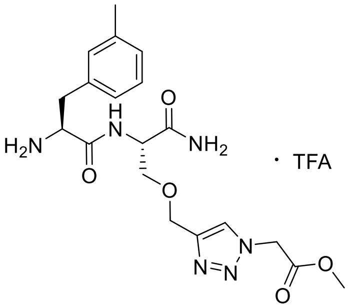

ESI (+):

m/z = 419.20 ($[M+H]^+$)

M ($C_{19}H_{27}N_6O_5^+$, monoisotopic): 419.20

The synthesis was accomplished according to GP II. using 0.21 g **39** (0.41 mmol) and 10 mL TFA/CH_2Cl_2. 3-Methyl-L-phenylalanyl-*O*-((1-(methylcarboxymethyl)-1*H*-1,2,3-triazol-4-yl)-methyl)-L-serinamide (**40**) was obtained as a yellow oil (0.25 g, quantitative yield; contained TFA).

^{1}H NMR (400 MHz, DMSO-d_6) δ = 8.11 - 8.03 (m, 4H, NH_3^+, CH triazol), 7.40 (s, 1H, NH), 7.23 - 7.16 (m, 2H, aromatic Phe), 7.13 - 7.03 (m, 4H, aromatic Phe, CONH$_2$), 5.27 (s, 2H, N-CH_2), 4.61 - 4.53 (m, 2H, OCH_2), 4.51 - 4.45 (m, 1H, C_αH Ser), 4.13 - 4.05 (m, 1H, C_αH Phe), 3.71 (s, 3H, $COOCH_3$), 3.16 - 3.05 (m, 3H, $C_\beta H_2$ Ser, C_β*H*H Phe), 2.91 - 2.83 (m, 1H, C_βH*H* Phe), 2.28 (s, 3H, CH_3).

^{13}C NMR (101 MHz, DMSO-d_6) δ = 170.46, 168.58, 168.00, 143.40 (C-4 triazol), 137.56, 134.68, 130.15, 128.40, 127.78, 126.59, 125.45, 69.99 (C_βH Ser), 63.61 (OCH_2), 53.56 (COOH*C*H$_3$), 53.25 (C_α Phe), 52.69 (C_α Ser), 50.45 (N-*C*H$_2$-COO), 36.91 (C_β Phe), 21.01 (CH_3).

N-(4-Fluorobenzoyl)-3-methyl-L-phenylalanyl-O-((1-(methylcarboxymethyl)-1H-1,2,3-triazol-4-yl)-methyl)-L-serinamide (41)

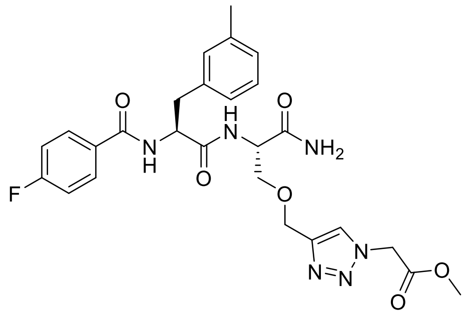

ESI (+):

m/z = 541.45 ([M+H]$^+$)

M ($C_{26}H_{30}FN_6O_6^+$, monoisotopic): 541.22

The synthesis was accomplished according to GP IV. using 0.24 g **40** (0.45 mmol, 1 eq.), 53µL 4-fluorobenzoyl chloride (0.45 mmol, 1 eq.), 186 µL TEA (1.35 mmol, 3 eq.) and 25 mL THF. *N-(*4-Fluorobenzoyl)-3-methyl-L-phenylalanyl-*O*-((1-(methylcarboxymethyl)-1*H*-1,2,3-triazol-4-yl)-methyl)-L-serinamide (**41**) was obtained as a yellowish solid (0.15 g, 0.27 mmol, 60%).

^{1}H NMR (600 MHz, CD_3CN) δ = 7.78 - 7.74 (m, 3H, H-2,6 aromatic FBz), 7.33 - 7.27 (m, 2H, CONH$_2$), 7.17 - 7.12 (m, 4H, aromatic), 7.10 - 7.01 (m, 2H, aromat), 6.33 (s, 1H, NH Ser), 5.79 (s, 1H, NH Phe), 5.17 (s, 2H, N-CH_2-COO), 4.73 - 4.68 (m, 1H, C_αH Phe), 4.61 - 4.55 (m, 2H, O-C*H*$_2$-triazol), 4.41 - 4.38 (m, 1H, C_αH Ser), 3.83 (dd, ^2J = 9.9, ^3J = 4.9 Hz, 1H C_β*H*H Ser), 3.72 (s, 3H, $COOCH_3$), 3.62 (dd, ^2J = 9.9, ^3J = 4.6 Hz, 1H, C_βH*H* Ser), 3.20 (dd, ^2J = 14.0, ^3J = 5.6 Hz, 1H, C_β*H*H Phe), 2.99 (dd, ^2J = 14.0, ^3J = 9.2 Hz, 1H, C_βH*H* Phe), 2.26 (s, 3H, Ar-CH_3).

^{13}C NMR (151 MHz, CD_3CN) δ = 172.37 (CO), 172.12 (CO), 168.53 (CO), 167.37 (CO), 165.66 (d, $^1J_{C,F}$ = 249.6 Hz, CF) , 145.46 (C-4 triazol), 139.06, 138.42, 130.96, 130.81, 130.75, 129.33, 128.32, 127.22, 125.75, 116.30 (d, $^2J_{C,F}$ = 22.1 Hz, C-3/5), 70.49 (C_β Ser), 64.94 (O-*C*H$_2$-triazol), 56.56 (C_α Phe), 53.99 (C_α Ser), 53.42 (COOH*C*H$_3$), 51.46, 37.86 (C_β Phe), 21.35 (CH_3).

^{19}F NMR (564 MHz, CD_3CN) δ = - 110.23 (FBz).

_N-(_4-Fluorobenzoyl)-3-methyl-L-phenylalanyl-_O_-((1-(methylcarboxymethyl)-1_H_-1,2,3-triazol-4-yl)-methyl)-L-serine nitrile (42)

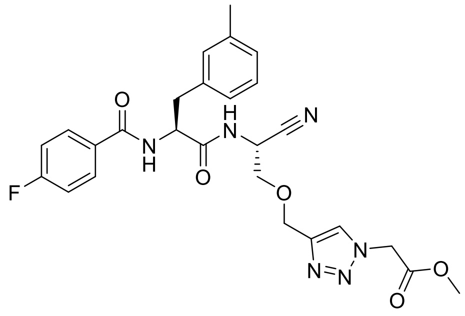

ESI (+):

m/z = 544.11 ($[M+Na]^+$)

M ($C_{26}H_{27}FN_6NaO_5^+$, monoisotopic): 545.19

The synthesis was accomplished according to GP V. using 0.12 g **41** (0.22 mmol, 1 eq.), 0.08 g cyanuric chloride (0.44 mmol, 2 eq.) and 10 mL DMF. *N-(*4-Fluorobenzoyl)-3-methyl-L-phenylalanyl-*O*-((1-(methylcarboxymethyl)-1*H*-1,2,3-triazol-4-yl)-methyl)-L-serine nitrile (**42**) was obtained as a yellow solid (0.13 g, quantitative yield).

^{1}H NMR (600 MHz, CD_3CN) δ = 7.95 (s, 1H, CH triazol), 7.92 - 7.85 (m, 2H, H-2,6 aromatic FBz), 7.62 (d, ^3J = 7.8 Hz, 1H, NH), 7.35 - 7.24 (m, 4H, H aromatic, NH), 7.24 - 7.12 (m, 3H, H aromatic), 5.32 (s, 2H, N-CH_2), 5.08 - 5.01 (m, 1H, C_αH Phe), 4.89 – 4.82 (m, 1H, C_αH Ser), 4.80 - 4.78 (m, 2H, OCH_2), 3.90 (dd, ^2J = 10.1, ^3J = 4.9 Hz, 1H, C_β*H*H Ser), 3.85 (s, 3H,$COOCH_3$), 3.83 (dd, ^2J = 10.1, ^3J = 5.2 Hz, 1H, C_βH*H* Ser), 3.31 (dd, ^2J = 13.9, ^3J = 5.6 Hz, 1H, C_β*H*H Phe), 3.10 (dd, ^2J = 13.9, ^3J = 8.9 Hz, 1H, C_βH*H* Phe), 2.38 (s, 3H, CH_3).

^{13}C NMR (151 MHz, CD_3CN) δ = 172.10 (CO), 168.50 (CO), 166.93 (CO), 165.63 (d, $^1J_{C,F}$ = 22.1 Hz, CF), 145.01 (C-4 triazol), 139.01, 138.14, 131.27 (d, $^4J_{C,F}$ = 3.1 Hz, C-1 FBz), 130.99, 130.77 (d, $^3J_{C,F}$ = 9.2 Hz, C-2/6 FBz), 129.29, 128.32, 127.25, 126.01, 116.26 (d, $^2J_{C,F}$ = 249.4 Hz, C-3/5), 69.50 (C_β Ser), 65.15 (OCH_2), 55.87 (C_α Phe), 53.42 (COOHCH_3), 51.49, 41.80 (C_α Ser), 37.95 (C_β Phe), 21.35 (CH_3).

Due to overlapping two signals were not identified.

_N_-(4-Fluorobenzoyl)-3-methyl-L-phenylalanyl-_O_-((1-(carboxymethyl)-1H-1,2,3-triazol-4-yl)-methyl)-L-serine nitrile (43)

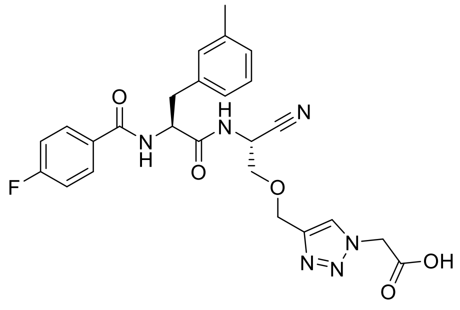

HR-MS ESI (+):

m/z = 531.1761 ($[M+Na]^+$)

M ($C_{25}H_{25}FN_6NaO_5^+$, monoisotopic): 531.1762

42 (0.01 g; 0.03 µmol; 1 eq.) was dissolved in THF/H_2O (5:1, 3 mL). The solution was cooled to 0°C before adding LiOH (1 M, 22.7 µL, 22.7 µmol, 0.85 eq.) and stirring the solution for 5 min at 0°C. Then, HCl (5 mL; 1 M) and ethyl acetate (10 mL) were added. The layers were separated and the aqueous phase was extracted with ethyl acetate (3 × 5 mL). The combined organic layers were washed with brine (5 mL), dried over Na_2SO_4 and the solvent evaporated. The crude product was purified via semipreparative HPLC. *N*-(4-Fluorobenzoyl)-3-methyl-L-phenylalanyl-*O*-((1-(carboxymethyl)-1H-1,2,3-triazol-4-yl)-methyl)-L-serine nitrile (**43**) was obtained was a white solid (6 mg, 0.01 mmol, 44%).

^{1}H NMR (400 MHz, DMSO-*d*$_6$) δ = 9.05 (d, ^3J = 7.7 Hz, 1H, NH Phe), 8.67 (d, ^3J = 8.3 Hz, 1H, NH Ser), 8.13 (s, 1H, H-5 triazol), 7.90 - 7.84 (m, 2H, H-2,6 aromatic FBz), 7.32 - 7.24 (m, 2H, aromatic), 7.17 - 7.09 (m, 3H, aromatic), 7.00 - 6.95 (m, 1H, aromatic), 5.28 (s, 2H, CH_2COO), 5.08 -4.99 (m, 1H, C_αH Phe), 4.73 - 4.65 (m, 3H, C_αH Ser, OCH_2), 3.72 (d, ^3J = 6.5 Hz, 2H, $C_\beta H_2$ Ser), 3.04 (dd, ^2J = 13.5 Hz, ^3J = 4.3 Hz, 1H, $C_\beta H\mathit{H}$ Phe), 2.95 (dd, ^2J = 13.6 Hz, ^3J = 10.3 Hz, 1H, $C_\beta \mathit{H}H$ Phe), 2.24 (s, 3H, CH_3).

The carboxyl proton was not detectable in the chosen solvent.

^{13}C NMR (101 MHz, DMSO-*d*$_6$) δ = 171.66 (CO), 168.55 (CO), 165.29 (CO), 163.91 (d, $^1J_{C,F}$ = 248.8 Hz, CF), 143.12, 137.82, 137.02,130.31 (d, $^4J_{C,F}$ = 2.8 Hz, C-1 FBz), 130.10 (d, $^3J_{C,F}$ = 9.1 Hz, C-2/6 FBz), 129.79, 127.94, 126.98, 126.25, 125.59, 117.99 (CN), 115.10 (d, $^2J_{C,F}$ = 21.8 Hz, C-3/5), 68.22 (C_β Ser), 63.78 (OCH_2), 54.76 (C_α Phe), 50.44, 40.51(C_α Ser), 36.85 (C_β Phe), 20.99 (CH_3).

^{19}F NMR (376 MHz, DMSO-*d*$_6$) δ = - 109.19 (FBz).

6 Synthesis of Dipeptide Alkynes 2a – m (Scheme 6)

L-Serine methyl ester (44a)

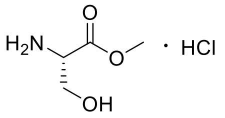

The reaction was performed under exclusion of moisture. Acetyl chloride (10 mL, 140 mmol, 3 eq.) was added dropwise to dry methanol (65 mL) over 5 min while cooling the solution in an ice bath. After stirring for 5 min, L-serine (5.00 g, 47.58 mmol, 1 eq.) was added. The solution was refluxed 2h before evaporating the solvent. The crude product was obtained as a clear, pungent-smelling solid and used without further purification.

N-(*tert*-Butyloxycarbonyl)-L-serine methyl ester (44b)

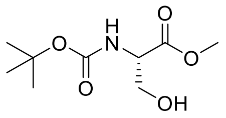

ESI (+):

m/z = 242.20 ($[M+Na]^+$)

M ($C_9H_{17}NNaO_5^+$, monoisotopic): 242.10

44a (7.40 g, 47.58 mmol, 1 eq.) and TEA (14.59 mL, 104.68 mmol, 2.2 eq.) were suspended in 200 mL dry THF. After a white precipitate formed, the solution was cooled to 0°C and Boc_2O (11.42 g, 52.34 mmol, 1.1 eq.) in dry THF (75 mL) added over 45 min. The reaction mixture was stirred over night at room temperature and 3 h at 50°C. The THF was evaporated and the residue dissolved in diethyl ether (150 mL) and sat. $NaHCO_3$ (200 mL). The aqueous phase was extracted with diethyl ether (3 × 100 mL). The combined organic layers were dried over Na_2SO_4 and the solvent evaporated. *N*-(*tert*-Butyloxycarbonyl)-L-serine methyl ester (**44b**) was obtained as a yellow oil (9.71 g, 40.09 mmol, 93% over two steps).

^{1}H NMR (400 MHz, $CDCl_3$) δ = 5.43 (s, 1H, NH), 4.39 (s, 1H, $C_\alpha H$), 4.01 - 3.86 (m, 2H, $C_\beta H_2$), 3.79 (s, 3H, COOC*H*$_3$), 1.99 (s, 1H, OH), 1.46 (s, 9H, (C*H*$_3$)$_3$).

^{13}C NMR (101 MHz, $CDCl_3$) δ = 171.36 (CO Ester), 155.86 (CO Boc), 80.53 (*C*$(CH_3)_3$), 63.82 ($C_\beta H_2$), 55.87 (C_αH), 52.81 (COO*C*H$_3$), 28.44 ((*C*H$_3$)$_3$).

^{1}H and ^{13}C NMR data are in agreement to published data.[17]

(S)-*N*-(*tert*-Butyloxycarbonyl)-4-methoxycarbonyl-2,2-dimethyl-1,3-oxazolidine (45)

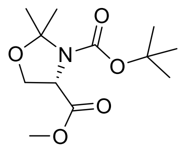

ESI (+):

m/z = 282.10 ($[M+Na]^+$)

M ($C_{12}H_{21}NNaO_5^+$, monoisotopic): 282.13

The reaction was performed under exclusion of moisture. **44b** (8.00 g, 36.49 mmol, 1 eq.) was dissolved in dry acetone (130 mL) and 2,2-dimethoxypropane (40 mL, 326.45 mmol, 9 eq.). Boron trifluoride diethyl etherate (280 µL, 2.21 mmol, 0.06 eq.) was added and the solution stirred 3 h. The reaction progress was monitored *via* thin layer chromatography. The reaction was quenched by adding TEA (720 µL) and the solvent evaporated. The residue was dissolved in diethyl ether (100 mL) and washed with sat. $NaHCO_3$-Lösung (150 mL). The washing solution was extracted with diethyl ether (3 × 25 mL) to ensure complete reextraction of product. The combined organic layers were dried over Na_2SO_4, the solvent evaporated and the product dried *in vacuo*. *(S)*-*N*-(*tert*-Butyloxycarbonyl)-4-methoxycarbonyl-2,2-dimethyl-1,3-oxazolidine (**45**) was obtained as a yellow oil (8.39 g, 32.36 mmol, 89%).

^{1}H NMR (400 MHz, $CDCl_3$) δ = 4.53 - 4.35 (m, 1H, CH_2-C*H*-N), 4.18 – 4.10 (m, 1H, O-CH*H*-CH), 4.08 – 4.00 (m, 1H, O-C*H*H-CH), 3.76 (s, 3H, $COOCH_3$), 1.67 (s, 3H, CH_3), 1.54 (s, 3H, CH_3), 1.42 (s, 9H, $C(CH_3)$).

Due to present rotamers additional signals were detected at 1.64 und 1.50.

^{13}C NMR (101 MHz, $CDCl_3$) δ = 171.86 (CO), 151.36 (CO), 95.23 (*C*-$(CH_3)_2$), 80.49 (*C*-$(CH_3)_3$), 66.43 (O-CH_2), 59.44 (N-CH), 52.45 (COO*C*H_3), 28.45 (C-(*C*H_3)$_3$), 25.12 (C(*C*H_3)(CH_3)), 24.55 (C(CH_3)(*C*H_3)).

Due to present rotamers additional signals were detected at 171.4, 152.27, 94.58, 81.07, 66.18, 59.37, 52.57, 28.52, 26.19 and 25.34.

Ratio of rotamers: 57:43

^{1}H and ^{13}C NMR data are in agreement to published data.[17]

(R)-N-(*tert*-Butoxycarbonyl)-4-hydroxymethyl-2,2-dimethyl-1,3-oxazolidine (46a)

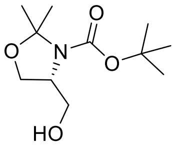

ESI (+):

m/z = 254.1 ([M+Na]$^+$)

M ($C_{11}H_{21}NNaO_4^+$, monoisotopic):254.14

The reaction was performed under argon atmosphere and exclusion of moisture. $LiAlH_4$ in THF (2.4 M, 19.28 mL, 46.28 mmol, 1.5 eq.) was diluted with dry THF (100 mL). **45** (8.00 g, 30.85 mmol, 1 eq.) in dry THF (45 mL) was added dropwise while stirring. The reaction progress was monitored *via* thin layer chromatography. After the reaction was completed (about 45 min), the reaction mixture was cooled to 0°C and aqueous KOH solution was added dropwise (16 mL, 10%ig (m/V)). The solution was stirred 1 h at room temperature. The solution was filtered to remove the precipitate and the precipitate washed three times with diethyl ether. The organic layer was washed with phosphate buffer (100 mL; pH = 7) and the aqueous layer extracted with diethyl ether (3 × 30 mL). All combined organic layers were dried over Na_2SO_4 and the solvent evaporated. The crude product was dried *in vacuo* and used without further purification. *(R)-N-*(*tert*-Butoxycarbonyl)-4-hydroxymethyl-2,2-dimethyl-1,3-oxazolidine (**46a**) was obtained as a clear solid (6.46 g, 28.1 mmol, 91%).

^{1}H NMR (600 MHz, $CDCl_3$) δ = 4.16 - 4.58 (m, 5H, O-CH_2, CH-N + CH-C*H*$_2$-OH), 2.47 (broad s, 1H, O*H*), 1.62 - 1.36 (m, 15H; (C*H*$_3$)$_3$, C(C*H*$_3$)$_2$).

^{13}C NMR (151 MHz, $CDCl_3$) δ = 155.53 (CO-Boc), 98.56 (*C*$(CH_3)_2$), 79.74 (*C*$(CH_3)_3$), 65.44 (O-CH_2), 64.17 (H_2C-OH), 59.74 (H*C*-CH_2-OH), 28.55 + 28.52 (C(*C*H_3)$_3$) + C(*C*H_3)$_2$).

Due to present rotamers, additional signals were detected at 156.47, 94.35, 80.04, 65.91, 64,09 and 28.62.

^{1}H and ^{13}C NMR data are in agreement to published data.[17]

***(S)-N-*(*tert*-Butyloxycarbonyl)-4-formyl-2,2-dimethyl-1,3-oxazolidine (46b; Garners aldehyde)**

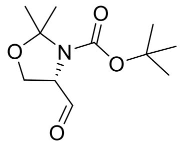

ESI (+):

m/z = 252.1 ([M+Na]$^{+}$)

M ($C_{11}H_{19}NNaO_4^{+}$, monoisotopic): 252.12

The reaction was performed under argon atmosphere and exclusion of moisture. Oxalyl chloride (3.50 mL, 40.86 mmol, 1.5 eq.) was dissolved in dry CH_2Cl_2 (70 mL) and cooled to - 78°C. DMSO (5.80 mL, 81.65 mmol, 3 eq.) in dry CH_2Cl_2 (8 mL) was added dropwise over 20 min while stirring. During the addition the temperature was raised to - 70°C. Then, the solution was stirred 20 min at - 60°C. **46a** (6.30 g, 27.24 mmol, 1 eq.) in dry CH_2Cl_2 (50 mL) was added dropwise over 40 min at – 60°C. Then, the solution was warmed to – 45°C and DiPEA (28.44 mL, 158 mmol, 5.8 eq.) in dry CH_2Cl_2 (5 mL) was added dropwise over 5 min. The solution was warmed to 0°C within 10 min. Ice-cold HCl (110 mL, 1 M) was added, the organic phase was separated and the aqueous layer was washed with CH_2Cl_2 (3 × 30 mL). The combined organic layers were washed with phosphate buffer (3 × 50 mL; pH = 7) and dried over Na_2SO_4. The solvent was evaporated and the crude product was purified *via* preparative column chromatography (petroleum ether/ethyl acetate (4:1)). *(S)-N-*(*tert*-Butyloxycarbonyl)-4-formyl-2,2-dimethyl-1,3-oxazolidine (**46b**, Garners aldehyde) was obtained as a yellow oil (5.06 g, 20.07 mmol, 81%).

$[\alpha]_D^{25}$ = –96.3 (c 1.03, $CHCl_3$), lit. $[\alpha]_D$ = –95.5 (c 0.78, $CHCl_3$).[17]

^{1}H NMR (600 MHz, $CDCl_3$) δ = 9.55 (d, ^{3}J = 2.6 Hz, 1H, C*H*O), 4.34 - 4.17 (m, 1H, H_2C-C*H*), 4.13 - 4.03 (m, 2H, O-CH_2), 1.65 (s, 3H, C(CH_3)$_2$), 1.56 (s, 3H, C(CH_3)$_2$), 1,43 (s, 9H, C(CH_3)$_3$).

Due to present rotamers additional signals were detected at 9.61, 1.60, 1.52 and 1.51.

^{13}C NMR (151 MHz, $CDCl_3$) δ = 153.96 (CO-Boc), 97.75 *C*(CH_3)$_2$), 83.74 (*C*(CH_3)$_3$), 67.34 (H*C*-CHO), 66.59 (O-*C*H_2-CH), 30.91 (C(*C*H_3)$_3$), 28.44, 26.45 (C(*C*H_3)$_2$).

The signal for the aldehyde carbonyl is not visible. Due to present rotamers additional signals were detected at 155.26, 96.98, 84.02, 67.41, 66.13, 33.56, 30.96, 29.35 and 27.34.

Ratio of rotamers: 57:43

^{1}H and ^{13}C NMR data are in agreement to published data.[17]

(R)-N-(*tert*-Butoxycarbonyl)-4-ethinyl-2,2-dimethyl-1,3-oxazolidine (46c)

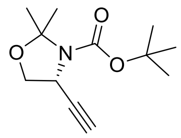

ESI (+):

m/z = 248.1 ([M+Na]$^+$)

M ($C_{12}H_{19}NNaO_3^+$, monoisotopic): 248.13

The reaction was performed under argon atmosphere and exclusion of moisture. Dimethyl (1-diazo-2-oxopropagyl)phosphate (4.75 mL, 19.62 mmol, 2 eq.) in dry MeOH (70 mL) was cooled to 0°C before adding potassium carbonate (2.70 g, 19.62 mmol, 2 eq.). The suspension was stirred 1h. Then, Garner´s aldehyde (**46b,** 2.25 g, 9.81 mmol, 1 eq.) in dry MeOH (80 mL) was added dropwise. The reaction progress was monitored *via* thin layer chromatography. After the reaction was completed (3 – 4 h), sat. NH_4Cl (150 mL) and petroleum ether (100 mL) were added. The aqueous phase was extracted with petroleum ether (2 × 50 mL). The combined organic layers were washed with brine (20 mL), dried over Na_2SO_4 and the solvent evaporated. The crude product was purified *via* preparative column chromatography (petroleum ether/ ethyl acetate (9:1)). *(R)-N-*(*tert*-Butoxycarbonyl)-4-ethinyl-2,2-dimethyl-1,3-oxazolidine (**46c**) was obtained as a yellowish oil (1.66 g, 7.26 mmol, 74%).

$[\alpha]_D^{25}$ = –96.3 (c 1.03, $CHCl_3$), lit. $[\alpha]_D^{20}$ = –95.2 (c 1.24, $CHCl_3$).[18]

^{1}H NMR (400 MHz, $CDCl_3$) δ = 4.56 (d, 1H, C*H*-C≡CH), 4.07 - 4.00 (m, 2H, O-C*H*$_2$), 2.27 (s, 1H, C≡C*H*), 1,64 (s, 3H, C(C*H*$_3$)(CH$_3$)), 1.50 (s, 12H, C(CH$_3$)(C*H*$_3$), C(C*H*$_3$)$_3$).

^{13}C NMR (101 MHz, $CDCl_3$) δ = 151.55 (CO-Boc), 94.61 (*C*(CH$_3$)$_2$), 82.95 (*C*≡CH), 80.57 (*C*(CH$_3$)$_3$), 70.25 (C≡C*H*), 68.89 (O-*C*H$_2$), 48.50 (N-*C*H), 28.57 (C(*C*H$_3$)$_3$), 26.03 (C(CH$_3$)(*C*H$_3$)), 24.52 (C(*C*H$_3$)(CH$_3$)).

Due to present rotamers additional signals were detected at 149.67, 94.18, 81.00, 70.65, 68,79, 27.02 and 25.32.

Ratio of rotamers: 57:43

^{1}H and ^{13}C NMR data are in agreement to published data.[19]

(R)-2-Aminobut-3-in-1-ol (47)

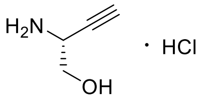

46c (1.56 g, 6.92 mmol 1 eq.) was dissolved in methanol (25 mL) bevor adding 4 M HCl (15 mL) while stirring. The solution was refluxed 1h. The reaction progress was monitored *via* thin layer chromatography. After the reaction was completed, the residue was dissolved in H_2O (10 mL) and filtered using a PTFE filter (0,2 µm) to remove insoluble residua. The clear solution was freezed and lyophilized over night. *(R)*-2-Aminobut-3-in-1-ol (**47**) was obtained as a clear solid (0.82 g, 6.71 mmol, 97%).

^{1}H NMR (400 MHz, DMSO-d_6) δ = 8.58 (s, 3H, NH_3^+), 5.70 (s, 1H, OH), 4.08 - 4.00 (m, 1H, C_αH), 3.72 - 3.66 (m, 1H, HO-CH*H*), 3.67 (d, ^2J = 2.3 Hz, 1H, C≡C*H*), 3.63 - 3.56 (m, 1H, HO-C*H*H).

^{13}C NMR (101 MHz, DMSO-d_6) δ = 78.18 (*C*≡CH), 77.77 (C≡*C*H), 61.31 (HO-*C*H_2), 43.64 (C_αH).

***(R)*-*N*-(*tert*-Butyloxycarbonyl)-2-aminobut-3-in-1-ol (48)**

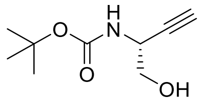

ESI (+):

m/z = 208.69 ([M+Na]$^+$)

M ($C_9H_{15}NNaO_3^+$, monoisotopic):208.09

47 (0.80 g, 6.60 mmol, 1 eq.) was suspended in dry THF (15 mL) and cooled to 0°C. The solution was stirred and TEA (2.04 mL, 14.52 mmol, 2.2 eq.) was added. Then, Di-*tert*-butyl dicarbonate (1.44 g, 6.60 mmol, 1 eq.) in dry THF (20 mL) was added dropwise. The solution was stirred over night (18 h). The solvent was evaporated, the residue was dissolved in sat. $NaHCO_3$ (40 mL) and diethyl ether (30 mL) and the phases separated. The aqueous phase was extracted with diethyl ether (3 × 10 mL). The combined organic phases were dried over Na_2SO_4 and the solvent evaporated. *(R)*-*N*-(*tert*-Butyloxycarbonyl)-2-aminobut-3-in-1-ol (**48**) was obtained as yellow solid (1.2 g, 6.47 mmol, 98%), which was sufficiently pure for further conversion. An aliquot of compound **48** was recrystallized from petroleum ether and ethyl acetate (minimum volume required for dissolution under heating) to obtain colorless needles (yield after recrystallisation: 85%; m.p.: 80-81°C, lit. 78-79°C[20]), which were suitable for structure determination by Xray diffraction analysis.

$[\alpha]_D^{25}$ = –39.6 (c 0.993, $CHCl_3$), lit. $[\alpha]_D^{20}$ = –37.9 (c 1.05, $CHCl_3$).[18]

^{1}H NMR (600 MHz, $CDCl_3$) δ = 5.01 (broad s, 1H, NH), 4.54 (broad s, 1H, C_αH), 3.79 - 3.67 (m, 2H, HO-C*H_2*), 2.34 (d, ^4J = 2.3 Hz, 1H, C≡CH), 1.99 (s, 1H, OH), 1.45 (s, 9H, $(CH_3)_3$).

^{13}C NMR (151 MHz, $CDCl_3$) δ = 155.64 (CO-Boc), 81.06 (*C*≡CH), 80.83 (*C*$(CH_3)_3$) , 72.92 (C≡*C*H), 66.04 (HO-CH_2), 45.58 (C_αH), 28.67 (C(*C*$H_3)_3$).

^{1}H and ^{13}C NMR data are in agreement to published data.[18]

***N*-(*tert*-Butyloxycarbonyl)-*O*-(3-(allyloxycarbonyl)benzyl)-L-serine alkyne (49)**

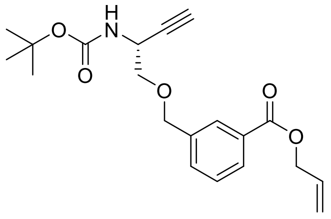

ESI (+):

m/z = 382.17 ($[M+Na]^+$)

M ($C_{20}H_{25}NNaO_5^+$, monoisotopic): 382.16

The reaction was performed under argon atmosphere and exclusion of moisture. **3b** (0.97 g, 3.22 mmol, 1 eq.) was dissolved in dry THF (3mL). In a separate flask, dry THF (27 mL) was cooled to 0°C and dry NaH (0.09 g, 3.74 mmol, 1.2 eq.) added while stirring vigorously. Increasing the amount of base leads to cleavage of the allyl group. The Alkyne **48** (0.60 g, 3.22 mmol, 1 eq.) was added at 0°C in one rush. After a deprotonation time of 1 – 2 min, **3b** was added swiftly. After gassing stopped, the solution was stirred 1.5 at room temperature. Reducing the reaction time to 30 min increases the yield (40% *vs* 30%). Unconverted **48** can be reisolated during the purification of the product.

To stop the reaction, KH_2PO_4 (aqueous solution, 50 mL, 5% (m/V)) and ethyl acetate (100 mL) were added and the organic layer separated. The aqueous phase was extracted with ethyl acetate (2 × 10 mL). The combined organic phases were washed with sat. $NaHCO_3$ (10 mL) and brine (10 mL). The solution was dried over Na_2SO_4 and the solvent evaporated. The crude product was purified *via* preparative column chromatography (petroleum ether to petroleum ether/ ethyl acetate (85:15)). *N*-(*tert*-Butyloxycarbonyl)-*O*-(3-(allyloxycarbonyl)benzyl)-L-serine alkyne (**49**) was obtained as a clear oil (0.47 g, 1.29 mmol, 40%).

^{1}H NMR (400 MHz, $CDCl_3$) δ = 8.05 - 7.97 (m, 2H, H-2,6 aromatic), 7.58 (d, ^3J = 7.7 Hz, 1H, H-4 aromatic), 7.44 (t, ^3J = 7.7 Hz, 1H, H-5 aromatic), 6.04 (m, 1H, C*H*=CH_2), 5.41 (dd, ^3J = 17.2, ^4J = 1.4 Hz, 1H, C=C*H*H_{trans}), 5.29 (dd, ^3J = 10.4, ^4J = 1.2 Hz, 1H, C=CHH_{cis}), 4.98 (s, 1H, NH), 4.83 (d, ^3J = 5.7 Hz, 2H, CH_2 allyl), 4.70 - 4.55 (m, 1H, C_αH), 4.65 (d, ^4J = 4.7 Hz, 2H, CH_2O), 3.65 (d, ^3J = 4.7 Hz, 2H, C_βH*H*), 2.30 (d, ^4J = 2.4 Hz, 1H, C≡C*H*), 1.44 (s, 9H, $(CH_3)_3$).

^{13}C-NMR (101 MHz, $CDCl_3$) δ = 166.23 (COO-allyl), 155.03 (CO Boc), 138.32, 132.36, 132.34, 130.51, 129.26, 128.94, 128.74, 118.49, 81.55, 80.41 (*C*≡CH), 72.16 (C≡*C*H), 71.68, 71.52, 65.79, 42.90 (C_αH), 28.47 ($C(CH_3)_3$).

Unexpectedly, initial attempts of subjecting **48** to *O*-alkylation with **3b** did not result in the desired product. Instead, **48** underwent cyclization and subsequent *N*-alkylation as revealed by NMR and MS analysis (Figure S65). The undesired side product **49a** was isolated as a colorless oil in a yield of 93% after stirring the serine alkyne **48** for 30 min in the presence of NaH before adding the alkylation agent **3b**. The unintended reaction course was attributed to the extended deprotonation time.

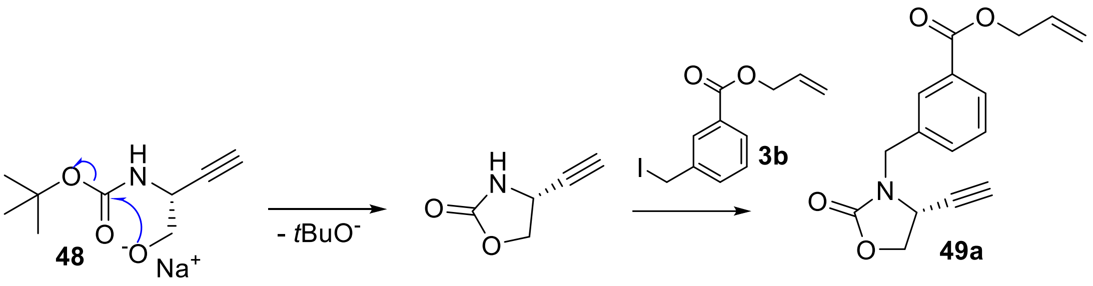

Figure S65: Intramolecular cyclization of Boc-serine alkyne (**48**) during preincubation with sodium hydride prior to the alkylation reaction with **3b**.

NMR data for allyl (*R*)-3-((4-ethynyl-2-oxooxazolidin-3-yl)methyl)benzoate (49a)

^{1}H NMR (400 MHz, $CDCl_3$) δ 8.05 – 8.00 (m, 2H, phenyl H-2, H-6), 7.58 – 7.54 (m, 1H, phenyl H-4), 7.48 – 7.43 (m, 1H, phenyl H-5), 6.10 – 5.99 (m, 1H, vinyl-CH), 5.42 (dq, ^3J = 17.2, ^4J = 1.5 Hz, 1H, vinyl-C*H*H), 5.31 (dq, ^3J = 10.4, ^4J = 1.3 Hz, 1H, vinyl-CH*H*), 4.91 (d, ^2J = 15.0 Hz, 1H, benzyl-C*H*H), 4.84 (dt, ^3J = 5.6, ^4J = 1.4 Hz, 2H, allyl-CH_2), 4.48 – 4.41 (m, 1H, 5-C*H*H), 4.32 – 4.23 (m, 3H, benzyl-CH*H*, 4-CH, 5-CH*H*), 2.55 (dd, ^4J = 2.0 Hz, 1H, CH alkyne).

^{13}C NMR (101 MHz, $CDCl_3$) δ 165.99 (CO ester), 157.20 (C-2), 135.86 (phenyl C-3), 133.29 (phenyl C-4), 132.22 (vinyl CH), 130.98 (phenyl C-1), 129.77, 129.61 (phenyl C-2, C-6, 129.22 (phenyl C-5), 118.61 (vinyl CH_2), 78.24, 76.05 (alkyne C, CH), 67.15 (C-5), 65.91 (allyl CH_2), 46.55 (C-4), 46.11 (benzyl CH_2).

***O*-(3-(Allyloxycarbonyl)benzyl)serine alkyne (50)**

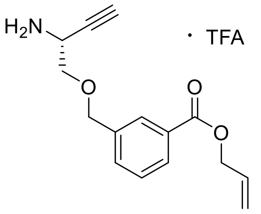

ESI (+):

m/z = 260.21 ($[M+H]^+$)

M ($C_{15}H_{18}NO_3^+$, monoisotopic): 260.13

The synthesis was accomplished according to GP II. using 0.14 g **49** (0.39 mmol) and 10 mL TFA/CH_2Cl_2 (1:1). *O*-(3-(Allyloxycarbonyl)benzyl)serine alkyne (**50**) was obtained as a white solid (0.14 g, quantitative yield).

^{1}H NMR (600 MHz, DMSO-d_6) δ = 8.51 (s, 3H, NH_3^+), 8.01 - 8.00 (m, 1H, H-2 aromatic), 7.96 - 7.93 (m, 1H, H-6 aromatic), 7.71 - 7.68 (m, 1H, H-4 aromatic), 7.56 (t, ^3J = 7.7 Hz, 1H, H-5 aromatic), 6.05 (m, 1H, C*H*=CH_2), 5.41 (dd, ^3J = 17.2, ^4J = 1.4 Hz, 1H, C=C*H*H_{trans}), 5.29 (dd, ^3J = 10.4, ^4J = 1.2 Hz, 1H, C=CHH_{cis}), 4.82 (dt, ^3J = 5.4, ^4J = 1.5 Hz, 2H, CH_2 allyl), 4.68 (m, 2H, CH_2O), 4.43 - 4.40 (m, 1H, $C_\alpha H$), 3.75 (d, ^4J = 2.4 Hz, 1H, C≡C*H*), 3.72 (dd, ^2J = 10.4, ^3J = 4.5 Hz, 1H, $C_\beta H$*H*), 3.67 (dd, ^2J = 10.4, ^3J = 6.8 Hz, 1H, C_β*H*H).

^{13}C NMR (151 MHz, DMSO-d_6) δ = 165.26 (CO), 138.28, 132.71, 132.58, 129.67, 128.88, 128.57, 128.43, 118.02 (C=CH_2), 78.68, 77.46 (C≡CH), 71.74 (CH_2O), 69.34 ($C_β$), 65.16 (CH_2 allyl), 41.53 ($C_α$H).

N-(*tert*-Butyloxycarbonyl)-3-methylphenylalanyl-*O*-(3-(allyloxycarbonyl)benzyl)-serine alkyne (51a)

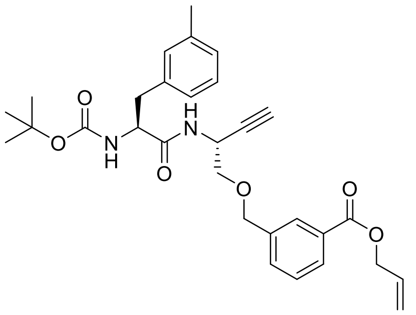

ESI (+):

m/z = 543.20 ($[M+Na]^+$)

M ($C_{30}H_{36}N_2NaO_6^+$, monoisotopic): 543.25

The synthesis was accomplished according to GP III. using 0.14 g **50** (0.36 mmol, 1 eq.), 0.15 g Boc-(3-Me)Phe-OH (0.53 mmol, 1.5 eq.), 245 µL DiPEA (1.41 mmol, 4 eq.), 0.28 g PyBOP (0.53 mmol, 1.5 eq.) and 10 mL THF. *N*-(*tert*-Butyloxycarbonyl)-3-methylphenylalanyl-*O*-(3-(allyloxycarbonyl)-benzyl)-serine alkyne (**51a**) was obtained as a clear solid (0.17 g, 0.32 mmol, 90%, contained 9% undesired diastereomer).

^{1}H NMR (600 MHz, $CDCl_3$) δ = 8.02 - 7.96 (m, 2H, H aromatic), 7.52 - 7,48 (m, 1H, H aromatic), 7.45 - 7.40 (m, 1H, H aromatic), 7.18 - 7.13 (m, 1H, H aromatic), 7.06 - 6.99 (m, 3H, aromatic), 6.26 (s, 1H, NH Ser), 6.08 - 5.99 (m, 1H, C*H*=CH_2), 5.44 - 5.38 (m, 1H, C*H*=C*H*H_{trans}), 5.31 - 5.27 (m, 1H, CH=C*H*H_{cis}), 5.21 (s, 1H, NH Ser), 4.94 (s, 1H, $C_α$H Phe), 4.84 - 4.80 (m, 2H CH_2 allyl), 4.63 - 4.51 (m, 2H, CH_2O), 4.30 (s, 1H, $C_α$H Ser), 3.57 (dd, ^2J = 9.7, ^3J = 4.8 Hz, 1H, $C_β$H*H*), 3.53 (dd, ^2J = 9.7, ^3J = 4.6 Hz, 1H, $C_β$*H*H), 3.07 - 2.92 (m, 2H, $C_βH_2$-Phe), 2.29 (s, 3H, Ar-CH_3), 2.27 (d, ^4J = 2.4 Hz, 1H, C≡C*H*), 1.38 (s, 9H, $(CH_3)_3$).

^{13}C NMR (151 MHz, $CDCl_3$) δ = 170.72 (CO Phe), 166.29 (COO-Allyl), 155.56 (CO Boc), 138.39, 138.27, 136.61, 132.31, 132.26, 130.49, 130.33, 130.20, 129.21, 128.88, 128.74, 127.84, 126.52, 118.57, 80.66 (*C*≡CH), 80.20, 72.70 (C≡*C*H), 71.97, 71.67, 65.85, 55.97 ($C_α$ Ser), 41.22 ($C_α$ Phe), 38.46, 28.37 (C(CH_3)$_3$), 21.52 (CH_3).

N-(tert-Butyloxycarbonyl)-3-iodo-L-phenylalanyl-O-(3-(allyloxycarbonyl)benzyl)-L-serine alkyne (51b)

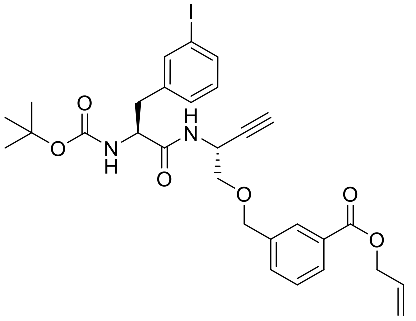

ESI (+):

m/z = 655.01 ([M+Na]+)

M ($C_{29}H_{34}IN_2O_6^+$, monoisotopic): 633.15

The synthesis was accomplished according to GP III. using 0.12 g **50** (0.32 mmol, 1 eq.), 0.19 g Boc-(3-I)Phe-OH (0.48 mmol, 1.5 eq.), 222 µL DiPEA (1.28 mmol, 4 eq.), 0.25 g PyBOP (0.48 mmol, 1.5 eq.) and 25 mL THF. The crude product was purified *via* semipreparative HPLC. *N*-(*tert*-Butyloxycarbonyl)-3-iodo-L-phenylalanyl-*O*-(3-(allyloxycarbonyl)benzyl)-L-serine alkyne (**51b**) was obtained as a white solid (0.09 g, 0.15 mmol, 64%, contained 2% undesired diastereomer).

^{1}H NMR (400 MHz, CD_3CN) δ = 8.03 – 8.00 (m, 1H, aromatic Phe), 7.98 – 7.94 (m, 1H, aromatic Phe), 7.64 – 7.57 (m, 3H, aromatic), 7.49 (t, ^3J = 7.6 Hz, 1H, aromatic BnCOO), 7.26 – 7.22 (m, 1H, aromatic Phe), 7.06 (t, ^3J = 7.8 Hz, 1H, aromatic Phe), 7.03 (d, ^3J = 9.4 Hz, 1H, NH), 6.13 – 6.02 (m, 1H, C*H*=CH_2), 5.55 (s, 1H, NH), 5.45 – 5.38 (m, 1H, CH=CH_{trans}), 5.31 – 5.25 (m, 1H, CH=CH_{cis}), 4.90 – 4.83 (m, 1H, C_αH Phe), 4.81 (dt, ^3J = 5.5 Hz, ^4J = 1.5 Hz, 2H, CH_2 allyl), 4.68 – 4.57 (m, 2H, CH_2O), 4.24 (s, 1H, C_αH Ser), 3.61 (d, ^3J = 5.3 Hz, 2H, $C_\beta H_2$ Ser), 3.06 (dd, ^3J = 14.0 Hz, ^2J = 5.2 Hz, 1H, C_βH*H* Phe), 2.76 (dd, ^3J = 13.9 Hz, ^2J = 9.0 Hz, 1H, C_β*H*H Phe), 2.55 (d, ^4J = 2.4 Hz, 1H; C≡CH), 1.31 (s, 9H, $(CH_3)_3$).

^{13}C NMR (101 MHz, CD_3CN) δ = 171.51 (CO), 166.81 (CO), 156.30 (CO), 141.26, 139.96, 139.27, 136.54, 133.62, 133.29, 131.33, 131.22 (C aromatic Phe), 129.90, 129.69, 129.53, 129.38, 118.26 (ACN, CH=*C*H_2), 94.65 (CI), 82.02 (*C*≡CH), 80.08 (*C*$(CH_3)_3$), 72.99, 72.72, 72.46, 66.33 (CH_2 allyl), 56.35 (C_α Ser), 41.79 (C_α Phe), 34.69 (C_β Phe), 28.43 (C(*C*H_3)$_3$).

3-Methyl-L-phenylalanyl-O-(3-(allyloxycarbonyl)benzyl)-L-serine alkyne (52a)

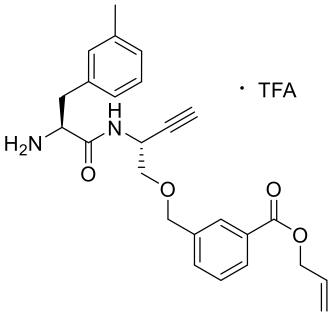

ESI (+):

m/z = 421.20 ([M+H]+)

M ($C_{25}H_{29}N2O_4^+$, monoisotopic): 421.21

The synthesis was accomplished according to GP II. using 0.14 g **51a** (0.24 mmol) and 10 mL TFA/CH_2Cl_2 (1:1). 3-Methyl-L-phenylalanyl-*O*-(3-(allyloxycarbonyl)benzyl)-L-serine alkyne (**52a**) was obtained as an orange oil (0.13 g, 0.24 mmol, quantitative yield, contained 15% undesired diastereomer).

^{1}H NMR (400 MHz, DMSO-d_6) δ = 9.01 (d, ^3J = 7.5 Hz, 1H, NH Ser), 8.16 (s, 3H, NH_3^+), 8.00 - 7.89 (m, 2H, aromatic), 7.66 - 7.63 (m, 1H, aromatic), 7.54 (t, ^3J = 7.7 Hz, 1H, aromatic), 7.21 (t, ^3J = 7.5 Hz, 1H, aromatic), 7.11 - 7.02 (m, 3H, aromatic), 6.10 - 5.99 (m, 1H, C*H*=CH_2), 5.44 - 5.36 (m, 1H, C=C*H*H_{trans}), 5.31 m 5.26 (m, 1H, C=CHH_{cis}), 4.85 m 4.74 (m, 3H, C_αH Phe, CH_2 allyl), 4.70 - 4.55 (m, 2H, CH_2O), 3.98 (s, 1H, C_αH Ser), 3.62 - 3.53 (m, 2H, $C_\beta H_2$ Ser), 3.32 (d, ^4J = 2.4 Hz, 1H, C≡C*H*), 3.06 - 2.89 (m, 2H, C_βH Phe), 2.28 (s, 3H, CH_3).

^{13}C NMR (101 MHz, DMSO-d_6) δ = 167.83 (CO Phe), 165.68 (COO-allyl), 139.20, 137.97, 134.82, 133.01, 132.86, 130.58, 130.08, 129.28, 128.83, 128.56, 128.25, 127.09, 118.43, 81.39 (*C*≡CH), 74.74 (C≡*C*H), 71.94, 71.41, 65.56 (CH_2 Allyl), 53.73 (C_α Phe), 41.20 (C_α Ser), 37.17 (C_β Phe), 21.44 (CH_3).

Due to overlapping two signals were not identified.

3-Iodo-L-phenylalanyl-*O*-(3-(allyloxycarbonyl)benzyl)-L-serine alkyne (52b)

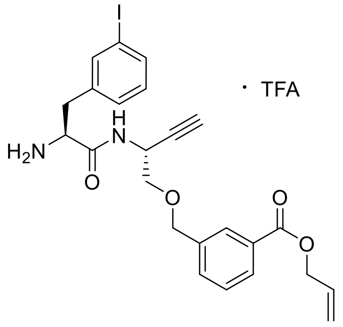

ESI (+):

m/z = 533.02 ([M+H]$^+$)

M ($C_{24}H_{26}IN_2O_4^+$, monoisotopic): 533.09

The synthesis was accomplished according to GP II. using 0.06 g **52b** (0.09 mmol) and 10 mL TFA/CH_2Cl_2 (1:1). 3-Iodo-L-phenylalanyl-*O*-(3-(allyloxycarbonyl)benzyl)-L-serine alkyne (**52b**) was obtained as a brownish solid (0.04 g, 0.06 mmol, 64%, contained TFA) and used without further characterization.

N-(2,4-Difluorobenzoyl)-3-methyl-L-phenylalanyl-O-(3-(allyloxycarbonyl)benzyl)-L-serine alkyne (53a)

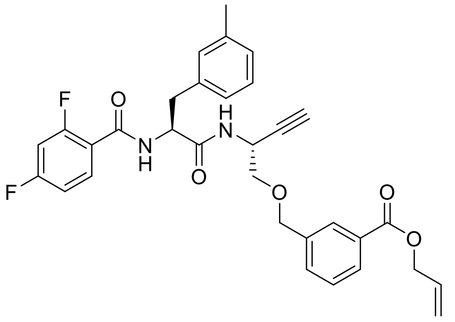

The synthesis was accomplished according to GP IV. using 0.03 g **52a** (0.06 mmol, 1 eq.), 7 µL 2,4-Difluorobenzoyl chloride (0.06 mmol, 1 eq.), 23 µL TEA (0.17 mmol, 2.8 eq.) and 10 mL CH_2Cl_2. *N-(2,*4-Difluorobenzoyl)-3-methyl-L-phenylalanyl-*O*-(3-(allyloxycarbonyl)benzyl)-L-serine alkyne (**53a**) was obtained as a white solid (7.5 mg, 0.04 mmol, 70%, contained 10% undesired diastereomer).

^{1}H NMR (400 MHz, CD_3CN) δ = 7.98 (s, 1H, H-2 BnCOO), 7.94 – 7.90 (m, 1H, H-4 BnCOO), 7.86 – 7.78 (m, 1H, H aromatic F_2Bz), 7.59 – 7.54 (m, 1H, H-6 BnCOO), 7.44 (t, ^3J = 7.7 Hz, 1H, H-5 BnCOO), 7.24 – 7.18 (m, 1H, NH), 7.18 – 7.12 (m, 1H, H aromatic), 7.09 – 6.97 (m, 6H, NH, H aromatic Phe, H aromatic F_2Bz), 6.11 – 5.98 (m, 1H, C*H*=CH_2), 5.43 – 5.35 (m, 1H, CH=CHH_{trans}), 5.30 – 5.23 (m, 1H, CH=CH_{cis}H), 4.93 – 4.84 (m, 1H, C_αH Ser), 4.80 – 4.68 (m, 3H, C_αH Phe, CH_2 allyl), 4.65 – 4.53 (m, 2H, CH_2O), 3.61 (d, ^3J = 5.6 Hz, 2H, $C_\beta H_2$ Ser), 3.19 – 3.11 (m, 1H, C_βH*H* Phe), 3.04 – 2.95 (m, 1H, C_β*H*H Phe), 2.57 (d, ^4J = 2.4 Hz, 1H, C≡CH), 2.27 (s, 3H, CH_3).

^{13}C NMR (101 MHz, CD_3CN) δ = 170.94 (CO), 166.74 (CO), 165.63 (d, $^1J_{C,F}$ = 252.2 Hz, C-4 F_2Bz), 163.06 (CO), 161.67 (d, $^1J_{C,F}$ = 250.8 Hz, C-2 F_2Bz), 139.92, 138.93, 137.78, 133.83, 133.62, 133.21, 131.27, 131.19, 129.62, 129.47, 129.29, 129.24, 128.38, 127.45, 119.20, 118.26 (solvent, C=CH_2), 113.00 (dd, $^2J_{C,F}$ = 21.7 Hz, $^4J_{C,F}$ = 3,5 Hz, C-5 F_2Bz), 105.30 (dd, $^2J_{C,F}$ = 28.2 ≈ 26.3 Hz, C-3 F_2Bz), 81.93, 73.00, 72.80, 72.47, 66.27 (CH_2 allyl), 55.77 (C_α Phe), 41.88 (C_α Ser), 38.38 (C_β Phe), 21.36 (CH_3).

N-(4-Fluorobenzoyl)-3-methyl-L-phenylalanyl-O-(3-(allyloxycarbonyl)benzyl)-L-serine alkyne (53b)

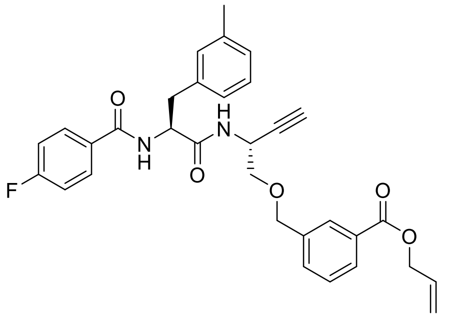

ESI (+):

m/z = 543.33 ($[M+H]^+$)

M ($C_{32}H_{32}FN_2O_5^+$, monoisotopic): 543.23

The synthesis was accomplished according to GP IV. using 0.11 g **52a** (0.20 mmol, 1 eq.), 23 µL 4-Fluorobenzoyl chloride (0.20 mmol, 1 eq.), 81 µL TEA (0.59 mmol, 3 eq.) and 15 mL CH_2Cl_2. *N-(*4-Fluorobenzoyl)-3-methyl-L-phenylalanyl-*O*-(3-(allyloxycarbonyl)benzyl)-L-serine alkyne (**53b**) was obtained as a yellowish solid (0.10 g, 0.18 mmol, 93%).

^{1}H NMR (400 MHz, CD_3CN) δ = 7.98 - 7.89 (m, 2H, aromatic), 7.76 - 7.69 (m, 2H, aromatic), 7.58 -7,53 (m, 1H, aromatic), 7.42 (t, ^3J = 7.6 Hz, 1H, aromatic), 7.22 - 7.00 (m, 8H, aromatic, NH Ser, NH Phe), 6.11 - 6.01 (m, 1H, C*H*=CH_2), 5.40 (dq, ^3J = 17.3, ^4J = 1.6 Hz, 1H, C=C*H*H_{trans}), 5.27 (dq, ^3J = 10.5, ^4J = 1.4 Hz, 1H, C=CHH_{cis}), 4.93 - 4.87 (m, 1H, C_αH Phe), 4.80 - 4.78 (m, 2H, CH_2 allyl), 4.76 - 4.69 (m, 1H, C_αH Ser), 4.59 (d, ^4J = 5.1 Hz, 2H, CH_2O), 3.62 (d, ^3J = 5.7 Hz, 2H, $C_\beta H_2$ Ser), 3.20 (dd, ^2J = 13.9, ^3J = 5.5 Hz, 1H, C_β*H*H Phe), 2,98 (dd, ^2J = 13,9, ^3J = 8,8 Hz, 1H, C_βH*H* Phe), 2.55 (d, ^4J = 2.4 Hz, 1H, C≡C*H*), 2.26 (s, 3H, CH_3).

^{13}C NMR (101 MHz, CD_3CN) δ = 171.40 (CO Phe), 166.83 (CO FBz), 166.80 (COO-Allyl), 165.85 (d, $^1J_{C,F}$ = 250.5 Hz, CF), 139.94, 138.91, 138.42, 133.61, 133.19, 131.39 (d, $^4J_{C,F}$ = 3.0 Hz, C-1 FBz), 131.25, 131.05, 130.68 (d, $^3J_{C,F}$ = 9.1 Hz, C-2/6 FBz), 129.62, 129.45, 129.26, 129.23, 128.24, 127.29, 118.26 (CD_3CN, C=*C*H_2) 116.22 (d, $^2J_{C,F}$ = 22.1 Hz, C-3/5 FBz), 82.02 (*C*≡CH), 72.98 (C≡*C*H), 72.73, 72.51, 66.30, 55.92 (C_α Ser), 41.85 (C_α Phe), 38.07 (*C*$_\beta$ Phe), 21.38 (CH_3).

^{19}F NMR (376 MHz, CD_3CN) δ = - 110.36 (FBz).

N-Diphenylacetyl-3-methyl-L-phenylalanyl-*O*-(3-(allyloxycarbonyl)benzyl)-L-serine alkyne (53c)

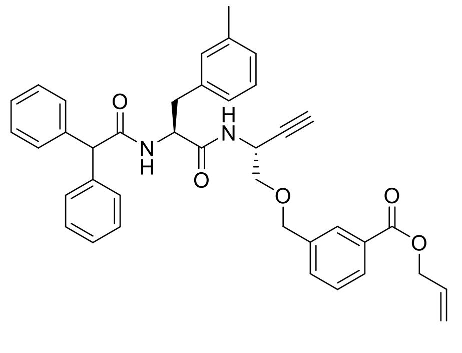

ESI (+):

m/z = 615.15 ([M+H]$^+$)

M ($C_{39}H_{39}N_2O_5^+$, monoisotopic): 615.29

The synthesis was accomplished according to GP III. using 0.05 g **52a** (0.09 mmol, 1 eq.), 0.03 g diphenylacetic acid (0.14 mmol, 1.5 eq.), 32 µL DiPEA (0.18 mmol, 2 eq.), 0.07 g PyBOP (0.14 mmol, 1.5 eq.) and 10 mL THF. *N*-Diphenylacetyl-3-methyl-L-phenylalanyl-*O*-(3-(allyloxycarbonyl)benzyl)-L-serine alkyne (**53c**) was obtained as a yellow oil (0.11 g, quantitative yield, contained 10% undesiered diastereomer and residual impurities).

^{1}H NMR (400 MHz, $CDCl_3$) δ = 8.00 – 7.96 (m, 1H, H-4 aromatic), 7.95 – 7.93 (m, 1H, H-2 aromatic), 7.47 – 7.43 (m, 1H, H-6 aromatic), 7.40 (t, ^3J = 7.6 Hz, 1H, H-5 aromatic), 7.25 – 7.17 (m, 6H, aromatic diphenyl), 7.13 (t, ^3J = 7.6 Hz, 1H, H-5 Phe),

7.10 – 7.00 (m, 5H, aromatic Phe, diphenyl), 6.96 (s, 1H, H-2 Phe), 6.92 (d, ^3J = 7.6 Hz, 1H, aromatic Phe), 6.58 (d, ^3J = 7.6 Hz, 1H, NH), 6.47 (d, ^3J = 8.6 Hz, 1H, NH), 6.10 – 5.98 (m, 1H, C*H*=CH$_2$), 5.45 – 5.37 (m, 1H, CH=CH_{trans}), 5.32 – 5.27 (m, 1H, CH=CH_{cis}), 4.99 – 4.90 (m, 1H, C$_\alpha$ Ser), 4.84 (s, 1H, CH diphenyl), 4.82 (dt, ^3J = 5.7, ^4J = 1.4 Hz, 2H, CH$_2$ allyl), 4.75 – 4.67 (m, 1H, C$_\alpha$ Phe), 4.59 – 4.46 (m, 2H, CH$_2$O), 3.54 – 3.51 (m, 2H, C$_\beta$ Ser), 3.10 – 2.92 (m, 2H, C$_\beta$ Phe), 2.29 (d, ^4J = 2.4 Hz, 1H, C≡CH), 2.27 (s, 3H, CH$_3$).

^{13}C NMR (101 MHz, CDCl$_3$) δ = 172.60, 170.27, 166.54, 139.04, 138.88, 138.43, 138.38, 136.58, 132.24 (*C*H=CH$_2$), 132.13, 130.36, 130.24, 129.06, 128.93, 128.88, 128.85, 128.82, 128.75, 128.69, 127.84, 127.40, 127.31, 126.48, 118.64 (CH=*C*H$_2$), 80.49, 72.41 (CH$_2$O), 72.07, 71.69 (C$_\beta$ Ser), 65.92 (CH$_2$ allyl), 58.86 (CH diphenyl), 54.70 (C$_\alpha$ Phe), 41.07 (C$_\alpha$ Ser), 37.20 (C$_\beta$ Phe), 21.54 (CH$_3$).

Due to overlapping two signals were not identified.

***N*-(4-Phenylbenzoyl)-3-methyl-L-phenylalanyl-*O*-(3-(allyloxycarbonyl)benzyl)-L-serine alkyne (53d)**

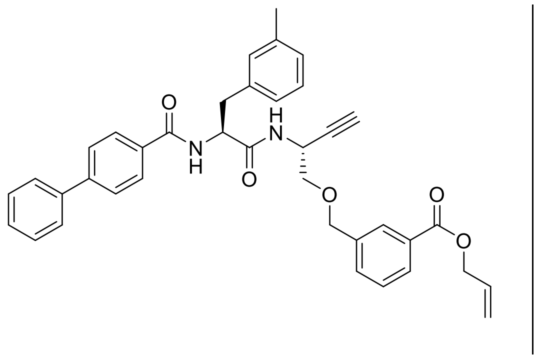

The synthesis was accomplished according to GP IV. using 0.06 g **52a** (0.11 mmol, 1 eq.), 0.02 g 4-phenylbenzoyl chloride (0.10 mmol, 1 eq.), 43 µL TEA (0.31 mmol, 3 eq.) and 10 mL CH$_2$Cl$_2$. *N*-(4-Phenylbenzoyl)-3-methyl-L-phenylalanyl-*O*-(3-(allyloxycarbonyl)benzyl)-L-serine alkyne (**53d**) was obtained as a white solid (0.03 g, 0.05 mmol, 43%, contained 9% undesired diastereomer).

^{1}H NMR (600 MHz, CD$_3$CN) δ = 7.97 (s, 1H, H-2 BnCOO), 7.91 – 7.89 (m, 1H, H-4 BnCOO), 7.76 – 7.74 (m, 2H, H aromatic), 7.67 – 7.64 (m, 4H, H aromatic), 7.56 – 7.53 (m, 1H, H aromatic), 7.50 – 7.46 (m, 2H, H aromatic), 7.42 – 7.39 (m, 2H, H aromatic), 7.25 (d, ^3J = 8.0 Hz, 1H, NH), 7.19 – 7.13 (m, 3H, NH, 2H aromatic Phe), 7.11 – 7.09 (m, 1H, H aromatic Phe), 7.04 – 7.02 (m, 1H, H aromatic Phe), 6.08 – 6.01 (m, 1H, C*H*=CH$_2$), 5.41 – 5.37 (m, 1H, CH=CHH_{trans}), 5.27 – 5.24 (m, 1H, CH=CH_{cis}H), 4.94 – 4.90 (m, 1H, C$_\alpha$H Ser), 4.79 – 4.74 (m, 3H, C$_\alpha$H Phe, CH$_2$ allyl), 4.63 – 4.56 (m, 2H, CH$_2$O), 3.63 (d, ^3J = 5.6 Hz, 2H, C$_\beta$H$_2$ Ser), 3.26 – 3.21 (m, 1H, C$_\beta$H*H* Phe), 3.04 – 2.98 (m, 1H, C$_\beta$*H*H Phe), 2.56 (d, ^4J = 2.4 Hz, 1H, C≡CH), 2.27 (s, 3H, CH$_3$).

^{13}C NMR (151 MHz, CD$_3$CN) δ = 171.49 (CO), 167.59 (CO), 166.79 (CO), 144.89, 140.67, 139.94, 138.91, 138.50, 133.74, 133.60, 133.16, 131.22, 131.07, 129.96,

129.61, 129.42, 129.23, 129.06, 128.69, 128.23, 128.01, 127.88, 127.85, 127.30, 118.36 (solvent, C=CH_2), 82.03, 72.96, 72.72, 72.53, 66.29 (CH_2 allyl), 55.88 ($C_α$ Phe), 41.83 ($C_α$ Ser), 38.00 ($C_β$ Phe), 21.39 (CH_3).

***N*-(4-Iodobenzoyl)-3-methyl-L-phenylalanyl-*O*-(3-(allyloxycarbonyl)benzyl)-L-serine alkyne (53e)**

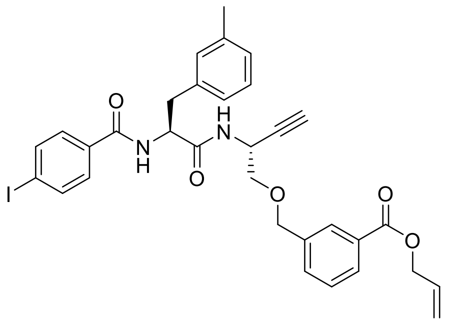

The synthesis was accomplished according to GP III. using 0.05 g **52a** (0.10 mmol, 1 eq.), 0.04 g 4-iodobenzoic acid (0.15 mmol, 1.5 eq.), 70 µL DiPEA (0.40 mmol, 4 eq.), 0.08 g PyBOP (0.15 mmol, 1.5 eq.) and 10 mL THF. *N*-(4-Iodobenzoyl)-3-methyl-L-phenylalanyl-*O*-(3-(allyloxycarbonyl)benzyl)-L-serine alkyne (**53e**) was obtained as a white solid (0.22 mg, 0.09 mmol, 89%, contained 9% undesired diastereomer and residual educt).

^{1}H NMR (600 MHz, DMSO-d_6) δ = 8.66 (d, ^3J = 8.3 Hz, 1H, NH), 8.60 (d, ^3J = 8.5 Hz, 1H, NH), 7.97 – 7.94 (m, 1H, H-2 BnCOO), 7.91 – 7.87 (m, 1H, H-4 BnCOO), 7.84 – 7.80 (m, 2H, H-3,5 IBz), 7.65 – 7.61 (m, 1H, H-6 BnCOO), 7.58 – 7.54 (m, 2H, H-2,6 IBz), 7.48 (t, ^3J = 7.7 Hz, 1H, H-5 BnCOO), 7.15 (s, 1H, H aromatic Phe), 7.12 – 7.09 (m, 2H, H aromatic Phe), 6.98 – 6.93 (m, 1H, H aromatic Phe), 6.07 – 6.00 (m, 1H, C*H*=CH_2), 5.42 – 5.37 (m, 1H, CH=CHH_{trans}), 5.29 – 5.25 (m, 1H, CH=CH_{cis}H), 4.85 – 4.78 (m, 3H, $C_α$H Phe, CH_2 allyl), 4.71 – 4.65 (m, 1H, $C_α$H Ser), 4.63 (s, 2H, CH_2O), 3.58 (d, ^3J = 6.2 Hz, 2H, $C_βH_2$ Ser), 3.24 (d, ^4J = 2.4 Hz, 1H, C≡CH), 3.04 – 2.99 (m, 1H, $C_β$H*H* Phe), 2.96 – 2.90 (m, 1H, $C_β$*H*H Phe), 2.23 (s, 3H, CH_3).

^{13}C NMR (151 MHz, DMSO-d_6) δ = 170.80 (CO), 165.47 (CO), 165.25 (CO), 138.92, 138.00, 137.02, 133.38, 132.58, 132.41, 129.81, 129.56, 129.35, 128.78, 128.64, 128.29, 128.05, 127.87, 126.88, 126.25, 117.99 (C=CH_2), 98.91 (CI), 81.83 (*C*≡CH), 73.92, 71.45, 71.22 ($C_β$ Ser), 65.10 (CH_2 allyl), 54.84 ($C_α$ Phe), 40.50 ($C_α$ Ser), 38.07 ($C_β$ Phe), 21.00 (CH_3).

N-(3-Iodobenzoyl)-3-methyl-L-phenylalanyl-O-(3-(allyloxycarbonyl)benzyl)-L-serine alkyne (53f)

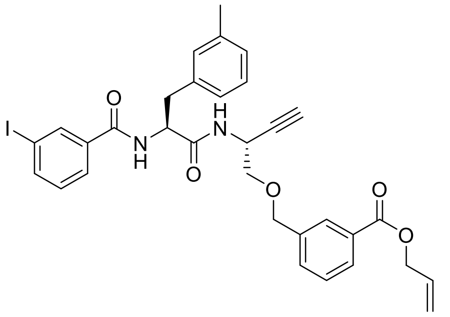

ESI (+):

m/z = 651.00 ([M+H]$^+$)

M ($C_{32}H_{32}IN_2O_5^+$, monoisotopic): 651.14

The synthesis was accomplished according to GP III. using 0.05 g **52a** (0.10 mmol, 1 eq.), 0.04 g 4-iodobenzoic acid (0.15 mmol, 1.5 eq.), 35 µL DiPEA (0.20 mmol, 2 eq.), 0.08 g PyBOP (0.15 mmol, 1.5 eq.) and 10 mL THF. *N-(*3-Iodobenzoyl)-3-methyl-L-phenylalanyl-*O*-(3-(allyloxycarbonyl)benzyl)-L-serine alkyne (**53f**) was obtained as a white solid (0.03 g, 0.04 mmol, 41%, contained 7% undesired diastereomer).

^{1}H NMR (400 MHz, $CDCl_3$) δ = 8.01 – 7.99 (m, 1H, H-1 IBz), 7.95 – 7.93 (m, 2H, H-2,4 aromatic), 7.79 – 7.75 (m, 1H, aromatic IBz), 7.64 – 7.60 (m, 1H, aromatic IBz), 7.45 – 7.41 (m, 1H, H-6 aromatic), 7.40 – 7.34 (m, 1H, H-5 aromatic), 7.22 – 7.16 (m, 2H, H-5 Phe, aromatic IBz), 7.14 – 7.03 (m, 4H, aromatic Phe, NH), 6.37 (d, ^3J = 8.5 Hz, 1H, NH), 6.12 – 5.99 (m, 1H, C*H*=CH_2), 5.46 – 5.39 (m, 1H, CH=CH_{trans}), 5.33 – 5.28 (m, 1H, CH=CH_{cis}), 5.02 – 4.94 (m, 1H, C_αH Ser), 4.87 – 4.84 (m, 2H, CH_2 allyl), 4.81 – 4.74 (m, 1H, C_αH Phe), 4.61 – 4.50 (m, 2H, CH_2O), 3.63 – 3.54 (m, 2H, $C_\beta H_2$ Ser), 3.23 – 3.11 (m, 2H, $C_\beta H_2$ Phe), 2.32 (s, 3H, CH_3), 2.31 (d, ^4J = 2.4 Hz, 1H, C≡CH).

^{13}C NMR (101 MHz, $CDCl_3$) δ = 170.22 (COO), 166.58 (CO), 166.14 (CO), 140.72, 138.56, 138.28, 136.61, 136.33, 136.05, 132.31, 132.07, 130.45, 130.26, 129.10, 128.87, 128.74, 128.68, 128.06, 126.60, 126.40, 118.64 (C=*C*H_2), 94.22 (CI), 80.41 (*C*≡CH), 77.36, 72.55, 72.18 (CH_2O), 71.76 (C_β Ser), 65.97 (CH_2 allyl), 55.18 (C_α Phe), 41.27 (C_α Ser), 38.12 (C_β Phe), 21.55 (CH_3).

N-Benzoyl-3-methyl-L-phenylalanyl-O-(3-(allyloxycarbonyl)benzyl)-L-serine alkyne (53g)

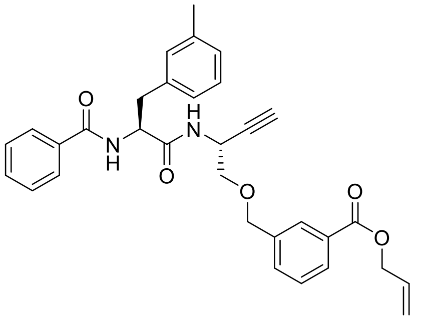

ESI (+):

m/z = 547.12 ([M+Na]$^+$)

M ($C_{32}H_{33}N_2O_5^+$, monoisotopic): 525.24

The synthesis was accomplished according to GP IV. using 0.03 g **52a** (0.06 mmol, 1 eq.), 14 µL benzoyl chloride (0.12 mmol, 2 eq.), 24 µL TEA (0.18 mmol, 3 eq.) and

10 mL CH_2Cl_2. *N*-*B*enzoyl-3-methyl-L-phenylalanyl-*O*-(3-(allyloxycarbonyl)benzyl)-L-serine alkyne (**53g**) was obtained as a clear oil (0.03 g, 0.05 mmol, 97%, contained 10% undesired diastereomer).

^{1}H NMR (400 MHz, CD_3CN) δ = 7.99 – 7.96 (m, 1H, H-2 BnCOO), 7.94 – 7.90 (m, 1H, H-4 BnCOO), 7.70 – 7.66 (m, 2H, H-2,6 Bn), 7.58 – 7.47 (m, 2H, H-4 Bn, H-6 BnCOO), 7.44 – 7.38 (m, 3H, H-5 BnCOO, H-3,5 Bn), 7.22 – 6.99 (m, 6H, aromatic Phe, 2 NH), 6.12 – 6.01 (m, 1H, C*H*=CH2), 5.45 – 5.36 (m, 1H, CH=CH_{trans}), 5.30 – 5.24 (m, 1H, CH=CH_{cis}), 4.94 – 4.87 (m, 1H, C_αH Phe), 4.81 – 4.77 (m, 2H, CH_2 allyl), 4.77 – 4.69 (m, 1H, C_αH Ser), 4.65 – 4.53 (m, 2H, CH_2O), 3.62 (d, ^3J = 5.6 Hz, 2H, $C_\beta H_2$ Ser), 3.24 – 3.18 (m, 1H, C_β*H*H Phe), 3.03 – 2.95 (m, 1H, C_βH*H* Phe), 2.55 (d, ^4J = 2.4 Hz, 1H, C≡CH), 2.26 (s, 3H, CH_3).

^{13}C NMR (101 MHz, CD_3CN) δ = 171.46 (CO), 167.87 (CO), 166.79 (COO), 139.94, 138.90, 138.44, 134.99, 133.62, 133.21, 132.55, 131.26, 131.07, 129.63, 129.46, 129.40, 129.28, 129.23, 128.24, 128.04, 127.31, 118.26 (C=*C*H2), 82.04 (*C*≡CH), 72.98, 72.70, 72.50, 66.29 (CH_2 allyl), 55.86 (C_α Ser), 41.84 (C_α Phe) , 38.07 (C_β Phe), 21.37 (CH_3).

***N*-(3-Fluorobenzoyl)-3-methyl-L-phenylalanyl-*O*-(3-(allyloxycarbonyl)benzyl)-L-serine alkyne (53h)**

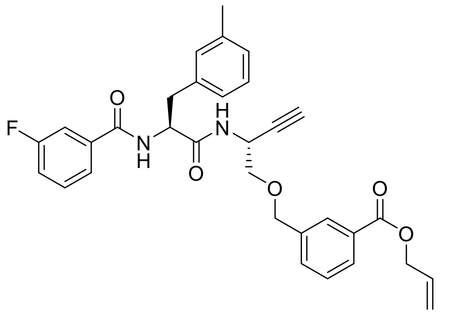

ESI (+):

m/z = 565.11 ([M+Na]$^+$)

M ($C_{32}H_{31}FN_2NaO_5^+$, monoisotopic): 565.21

The synthesis was accomplished according to GP IV. using 0.03 g **52a** (0.06 mmol, 1 eq.), 7 µL 3-fluorobenzoyl chloride (0.06 mmol, 1 eq.), 25 µL TEA (0.18 mmol, 3 eq.) and 10 mL CH_2Cl_2. *N*-(3-Fluorobenzoyl)-3-methyl-L-phenylalanyl-*O*-(3-(allyloxycarbonyl)benzyl)-L-serine alkyne (**53h**) was obtained as a yellow oil (0.03 g, 0.05 mmol, 87%).

^{1}H NMR (400 MHz, $CDCl_3$) δ = 7.97 – 7.91 (m, 2H, H-2,4 BnCOO), 7.46 – 7.30 (m, 5H, H-5,6 BnCOO, FBz), 7.23 – 7.02 (m, 6H, NH, 1H FBz, aromatic Phe), 6.34 (d, ^3J = 8.4 Hz, 1H, NH), 6.11 – 5.99 (m, 1H, C*H*=CH2), 5.46 – 5.38 (m, 1H, CH=CH_{trans}), 5.34 – 5.27 (m, 1H, CH=CH_{cis}), 5.01 – 4.93 (m, 1H, C_αH Ser), 4.85 – 4.81 (m, 2H, CH_2 allyl), 4.81 – 4.76 (m, 1H, C_αH Phe), 4.61 – 4.51 (m, 2H, CH_2O), 3.61 – 3.53 (m, 2H, $C_\beta H_2$ Ser), 3.25 – 3.08 (m, 2H, $C_\beta H_2$ Phe), 2.33 – 2.27 (m, 4H, CH_3, C≡CH).

^{13}C NMR (101 MHz, $CDCl_3$) δ = 170.23 (CO), 166.53 (CO), 166.38 (CO), 162.79 (d, $^1J_{C,F}$ = 247.7 Hz, CF), 138.55, 138.22, 136.52, 136.18 (d, $^3J_{C,F}$ = 7.0 Hz, C-1 FBz), 132.24, 132.13, 130.43, 130.36, 130.30 (d, $^3J_{C,F}$ = 7.9 Hz, C-5 FBz), 129.11, 128.85,

128.76, 128.68, 128.04, 126.58, 122.70 (d, $^4J_{C,F}$ = 3.1 Hz, C-6 FBz), 118.90 (d, $^2J_{C,F}$ = 21.2 Hz, C-4 FBz), 118.65 (C=*C*H2), 114.66 (d, $^2J_{C,F}$ = 22.9 Hz, C-2 FBz), 80.32 (*C*≡CH), 72.53 (CH_2O), 72.20, 71.65 ($C_β$ Ser), 65.93 (CH_2 allyl), 55.17 ($C_α$ Phe), 41.22 ($C_α$ Ser), 38.19 ($C_β$ Phe), 21.50 (CH_3).

N-(3-Bromobenzoyl)-3-methyl-L-phenylalanyl-O-(3-(allyloxycarbonyl)benzyl)-L-serine alkyne (53i)

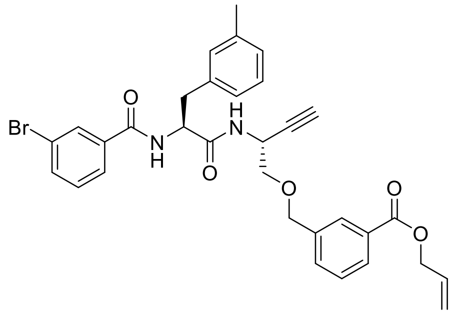

HR-MS ESI (+):

m/z = 603.1487 ($[M+H]^+$)

M ($C_{32}H_{32}BrN_2O_5^+$, monoisotopic): 603.1489

The synthesis was accomplished according to GP IV. using 0.03 g **52a** (0.06 mmol, 1 eq.), 7 µL 3-bromobenzoyl chloride (0.06 mmol, 1 eq.), 23 µL TEA (0.17 mmol, 2.8 eq.) and 10 mL CH_2Cl_2. *N-(*3-Bromobenzoyl)-3-methyl-L-phenylalanyl-*O*-(3-(allyloxycarbonyl)benzyl)-L-serine alkyne (**53i**) was obtained as a yellow, wax-like solid (0.03 g, 0.05 mmol, 88%, contained 7% undesired diastereomer).

^{1}H NMR (400 MHz, CD_3CN) δ = 7.98 – 7.96 (m, 1H, H-2 BnCOO), 7.93 – 7.89 (m, 1H, H-4 BnCOO), 7.82 – 7.80 (m, 1H, H-2 BrBz), 7.67 – 7.61 (m, 2H, H-4,6 BrBz), 7.57 – 7.54 (m, 1H, H-6 BnCOO), 7.42 (t, ^3J = 7.6 Hz, 1H, H-5 BnCOO), 7.35 – 7.27 (m, 2H, NH, H-4 BrBz), 7.18 – 7.00 (m, 5H, NH, aromatic Phe), 6.14 – 6.01 (m, 1H, C*H*=CH_2), 5.44 – 5.37 (m, 1H, CH=CH_{trans}), 5.31 – 5.24 (m, 1H, CH=CH_{cis}), 4.97 – 4.87 (m, 1H, $C_αH$ Ser), 4.82 – 4.77 (m, 2H, CH_2 allyl), 4.76 – 4.68 (m, 1H, $C_αH$ Phe), 4.64 – 4.53 (m, 2H, CH_2O), 3.62 (d, ^3J = 5.7 Hz, 2H, $C_βH_2$ Ser), 3.24 – 3.16 (m, 1H $C_β$H*H* Phe), 3.01 – 2.92 (m, 1H, $C_β$*H*H Phe), 2.55 (d, ^4J = 2.4 Hz, 1H; C≡CH), 2.27 (s, 3H, CH_3).

^{13}C NMR (101 MHz, CD_3CN) δ = 171.28 (CO), 166.82 (CO), 166.53 (CO), 139.94, 138.91, 138.39, 137.16, 135.32, 133.61, 133.16, 131.34, 131.24, 131.13, 131.08, 129.62, 129.45, 129.25, 129.23, 128.26, 127.27, 127.00, 122.89 (CBr), 118.26 (CH_3CN, *C*=CH_2), 81.97 (*C*≡CH), 72.95, 72.74, 72.50, 66.31 (CH_2 allyl), 56.02 ($C_α$ Ser), 41.84 ($C_α$ Phe), 38.01 ($C_β$ Phe), 21.39 (CH_3).

N-(3-(Trifluoromethyl)benzoyl)-3-methyl-L-phenylalanyl-*O*-(3-(allyloxycarbonyl)benzyl)-L-serine alkyne (53j)

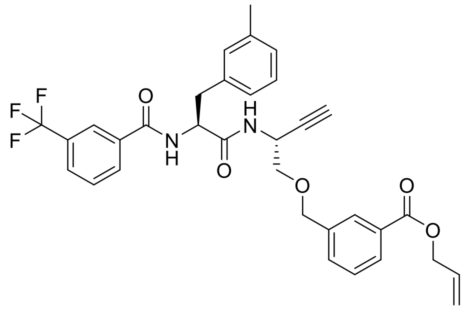

HR-MS ESI (+):

m/z = 593.2255 ([M+H]$^+$)

M ($C_{33}H_{32}F_3N_2O_5^+$, monoisotopic): 593.2258

The synthesis was accomplished according to GP IV. using 0.03 g **52a** (0.06 mmol, 1 eq.), 13 µL 3-(trifluoromethyl)benzoyl chloride (0.08 mmol, 1.5 eq.), 23 µL TEA (0.17 mmol, 3 eq.) and 10 mL CH_2Cl_2. *N*-(3-(Trifluoromethyl)benzoyl)-3-methyl-L-phenylalanyl-*O*-(3-(allyloxycarbonyl)benzyl)-L-serine alkyne (**53j**) was obtained as a yellow oil (0.03 g, 0.05 mmol, 87%, contained 12% undesired diastereomer).

^{1}H NMR (400 MHz, CD_3CN) δ = 8.00 – 7.94 (m, 2H, H-2 BnCOO, H-2 F_3CBz), 7.93 – 7.88 (m, 2H, H-4 BnCOO, H-4 F_3CBz), 7.82 – 7.78 (m, 1H, H-6 F_3CBz), 7.63 – 7.52 (m, 2H, H-5 F_3CBz, H-6 BnCOO), 7.45 – 7.37 (m, 2H, H-5 BnCOO, NH), 7.21 – 7.05 (m, 4H, NH, H-2,4,5 aromatic Phe), 7.02 (d, ^3J = 7.4 Hz, 1H, H-6 aromatic Phe), 6.11 – 5.99 (m, 1H, C*H*=CH$_2$), 5.44 – 5.35 (m, 1H, CH=CH_{trans}), 5.29 – 5.23 (m, 1H, CH=CH_{cis}), 4.95 – 4.86 (m, 1H, C$_\alpha$H Ser), 4.80 – 4.72 (m, 3H, CH$_2$ allyl, C$_\alpha$H Phe), 4.63 – 4.55 (m, 2H, CH$_2$O), 3.62 (d, ^3J = 5.7 Hz, 2H, C$_\beta$H$_2$ Ser), 3.25 – 3.18 (m, 1H, C$_\beta$H*H* Phe), 3.03 – 2.94 (m, 1H, C$_\beta$*H*H Phe), 2.55 (d, ^4J = 2.4 Hz, 1H, C≡CH), 2.26 (s, 3H, CH$_3$).

^{13}C NMR (101 MHz, CD_3CN) δ = 171.26 (CO), 166.80 (CO), 166.59 (CO), 139.93, 138.91, 138.38, 135.96, 133.59, 133.15, 131.93, 131.23, 131.06, 130.85, 130.45, 129.59, 129.43, 129.24, 129.02 (q, $^3J_{C,F}$ = 3.8 Hz, C-4 CF$_3$Bz), 128.25, 127.28, 125,00 (q, $^1J_{C,F}$ = 271.6 Hz, CF$_3$), 124,96 (q, $^3J_{C,F}$ = 4.0 Hz, C-2 CF$_3$Bz) 118.53 (ACN, C=*C*H$_2$), 81.96 (*C*≡CH), 72.95, 72.74, 72.49, 66.28 (CH$_2$ allyl), 56.08 (C$_\alpha$ Phe), 41.85 (C$_\alpha$ Ser), 38.05 (C$_\beta$ Phe), 21.33 (CH$_3$).

N-(4-Fluorobenzoyl)-3-iodo-L-phenylalanyl-*O*-(3-(allyloxycarbonyl)benzyl)-L-serine alkyne (53k)

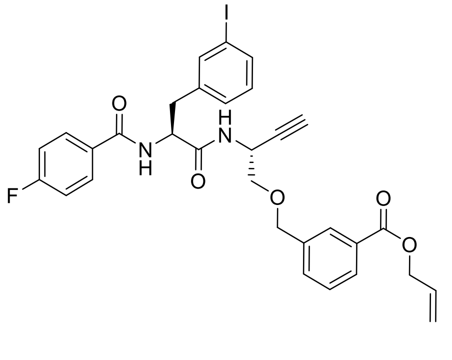

ESI (+):

m/z = 676.95 ([M+Na]$^+$)

M ($C_{31}H_{29}FIN_2O_5^+$, monoisotopic): 655.11

The synthesis was accomplished according to GP IV. using 0.05 g **52b** (0.09 mmol, 1 eq.), 9 µL 4-fluorobenzoyl chloride (0.09 mmol, 1 eq.), 36 µL TEA (0.26 mmol, 2.9 eq.) and 5 mL CH_2Cl_2. *N*-(4-Fluorobenzoyl)-3-iodo-L-phenylalanyl-*O*-(3-(allyloxycarbonyl)benzyl)-L-serine alkyne (**53k**) was obtained as a white solid (0.02 g, 0.03 mmol, 31%, contained 4% undesired diastereomer).

^{1}H NMR (400 MHz, $CDCl_3$) δ = 7.95 – 7.91 (m, 2H, H-2,4 aromatic BnCOO), 7.73 – 7.66 (m, 3H, H-3,5 aromatic FBz, H-2 aromatic Phe), 7.58 – 7.55 (m, 1H, H-4 aromatic Phe), 7.44 – 7.35 (m, 2H, H-5,6 aromatic BnCOO), 7.30 – 7.27 (m, 2H, NH, H aromatic Phe), 7.06 – 6.99 (m, 3H, H-2,6 aromatic FBz, H aromatic Phe), 6.54 (d, ^3J = 8.5 Hz, 1H, NH), 6.12 – 6.00 (m, 1H, C*H*=CH_2), 5.47 – 5.40 (m, 1H, CH=CHH_{trans}), 5.34 – 5.29 (m, 1H, CH=CH_{cis}H), 5.04 – 4.97 (m, 1H, C_αH Ser), 4.89 – 4.85 (m, 2H, CH_2 allyl), 4.84 – 4.78 (m, 1H,C_αH Phe), 4.63 – 4.51 (m, 2H, CH_2O), 3.65 – 3.55 (m, 2H, $C_\beta H_2$ Ser), 3.22 – 3.10 (m, 2H, $C_\beta H_2$ Phe), 2.36 (d, ^4J = 2.4 Hz, 1H, C≡CH).

^{13}C NMR (101 MHz, $CDCl_3$) δ = 169.99 (CO Phe), 166.88 (CO FBz), 166.77 (COO), 165.03 (d, $^1J_{C,F}$ = 252.3 Hz, CF), 139.34 , 138.73, 138.31, 136.29, 132.17, 132.10, 130.61, 130.30, 129.99 (d, $^4J_{C,F}$ = 3.2 Hz, C-1 FBz), 129.65 (d, $^3J_{C,F}$ = 9.1 Hz, C-2/6 FBz), 129.04, 128.77, 128.68, 118.70 (CH=*C*H_2), 115.70 (d, $^2J_{C,F}$ = 21.8 Hz, C-3/5 FBz), 94.75 (C-3 Phe), 80.21 (*C*≡CH), 72.48 (C≡*C*H), 72.36 (CH_2O), 71.70 (C_β Ser), 66.06 (CH_2 allyl), 54.83 (C_α Phe), 41.10 (C_α Ser), 37.46 (C_β Phe).

Due to overlapping one signal was not identified.

Diastereomeric purity > 96%

_N_-Diphenylacetyl-3-iodo-L-phenylalanyl-_O_-(3-(allyloxycarbonyl)benzyl)-L-serine alkyne (53l)

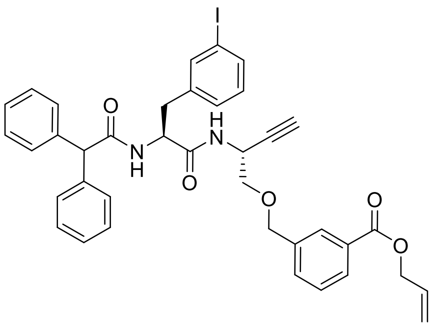

The synthesis was accomplished according to GP III. using 0.02 g **52b** (0.03 mmol, 1 eq.), 0.01 g diphenylacetic acid (0.05 mmol, 1.7 eq.), 24 µL DiPEA (0.14 mmol, 4.7 eq.), 0.03 g PyBOP (0.05 mmol, 1.7 eq.) and 5 mL THF. *N*-Diphenylacetyl-3-iodo-L-phenylalanyl-*O*-(3-(allyloxycarbonyl)benzyl)-L-serine alkyne (**53l**) was obtained as a white solid (0.01 g, 0.02 mmol, 46%) and used without further characterization.

N-(3-Iodobenzoyl)-3-iodo-L-phenylalanyl-O-(3-(allyloxycarbonyl)benzyl)-L-serine alkyne (53m)

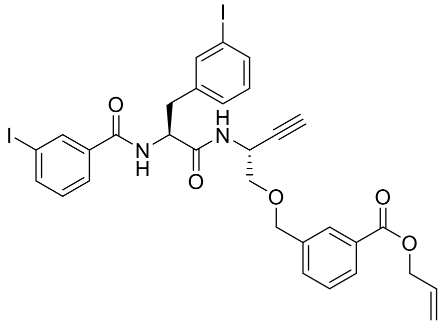

HR-MS ESI (+):

m/z = 763.0159 ([M+H]$^+$)

M ($C_{31}H_{29}I_2N_2O_5^+$, monoisotopic): 763.0161

The synthesis was accomplished according to GP III. using 0.01 g **52b** (0.02 mmol, 1 eq.), 0.06 g 3-iodobenzoic acid (0.02 mmol, 1 eq.), 5 µL DiPEA (0.03 mmol, 1.5 eq.), 0.11 g PyBOP (0.02 mmol, 1 eq.) and 10 mL THF. *N*-(3-Iodobenzoyl)-3-iodo-L-phenylalanyl-*O*-(3-(allyloxycarbonyl)benzyl)-L-serine alkyne (**53m**) was obtained as a white solid (0.01 g, 0.01 mmol, 54%, contained < 5% undesired diastereomer).

^{1}H NMR (400 MHz, $CDCl_3$) δ = 7.99 (t, ^3J = 1.7 Hz, 1H, H-2 IBz), 7.94 – 7.90 (m, 2H, H-2,4 BnCOO), 7.77 – 7.72 (m, 2H, H-4,6 IBz), 7.63 – 7.55 (m, 2H, H-2,4 Phe), 7.48 – 7.33 (m, 3H, NH, H-5,5 BnCOO), 7.30 – 7.26 (m, 1H, H-6 Phe), 7.09 – 7.01 (m, 2H, H-5 Phe, H-5 IBz), 6.64 (d, ^3J = 8.6 Hz, 1H, NH), 6.13 – 6.00 (m, 1H, C*H*=CH_2), 5.48 – 5.40 (m, 1H, CH=CH_{trans}), 5.35 – 5.29 (m, 1H, CH=CH_{cis}), 5.08 – 4.99 (m, 1H, C_αH Ser), 4.89 (dt, ^3J = 5.6, ^4J = 1.4 Hz, 2H, CH_2 allyl), 4.86 – 4.78 (m, 1H, C_αH Phe), 4.63 – 4.51 (m, 2H, CH_2O), 3.66 – 3.52 (m, 2H, $C_\beta H_2$ Ser), 3.25 – 3.09 (m, 2H, $C_\beta H_2$ Phe), 2.36 (d, ^4J = 2.4 Hz, 1H, C≡CH).

^{13}C NMR (101 MHz, CDCl3) δ = 169.99 (CO), 166.94 (COO), 166.64 (CO), 140.81, 139.28, 138.73, 138.29, 136.30, 135.70, 132.16, 132.00, 130.60, 130.24, 129.00, 128.74, 128.67, 128.55, 126.46, 118.74 (C=*C*H_2), 94.78, 94.24, 80.13 (*C*≡CH), 72.54, 72.26, 71.74, 66.20 (CH_2 allyl), 54.90 (C_α Phe), 41.08 (C_α Ser), 37.26 (C_β Phe).

Due to overlapping two signals were not identified.

N-(2,4-Difluorobenzoyl)-3-methyl-L-phenylalanyl-O-(3-carboxybenzyl)serine alkyne (2a)

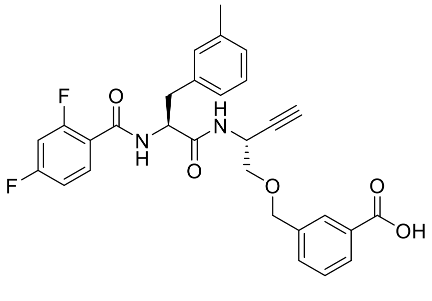

HR-MS ESI (+):

m/z = 543.1702 ([M+Na]$^+$)

M ($C_{29}H_{26}F_2N_2NaO_5^+$, monoisotopic): 543.1702

The synthesis was accomplished according to GP VI. using 0.02 g **53a** (0.03 mmol, 1 eq.), 26 µL morpholine (0.30 mmol, 10 eq.), 0.01 g $Pd(PPh_3)_4$ (0.01 mmol, 0.3 eq.) and 10 mL CH_2Cl_2. The crude product was purified *via* semipreparative HPLC. *N*-(2,4-Difluorobenzoyl)-3-methyl-L-phenylalanyl-*O*-(3-carboxybenzyl)serine alkyne (**2a**) was obtained as a white solid (5.0 mg, 0.01 mmol, 32%).

^{1}H NMR (600 MHz, CD_3CN) δ = 7.95 (s, 1H, H-2 BnCOO), 7.89 (d, ^3J = 7.7 Hz, 1H, H-4 BnCOO), 7.85 – 7.80 (m, 1H, H-6 FBz), 7.55 (d, ^3J = 7.7 Hz, 1H, H-6 BnCOO), 7.42 (t, ^3J = 7.6 Hz, 1H, H-5 BnCOO), 7.26 – 7.22 (m, 1H, NH), 7.15 (t, ^3J = 7.6 Hz, 1H, H-5 Phe), 7.10 – 6.96 (m, 6H, NH, 2H aromatic FBz, 3H aromatic Phe), 4.92 – 4.86 (m, 1H, C_αH Ser), 4.79 – 4.74 (m, 1H, C_αH Phe), 4.64 – 4.55 (m, 2H, CH_2O), 3.61 (d, ^3J = 5.9 Hz, 2H, $C_\beta H_2$ Ser), 3.20 – 3.14 (m, 1H, C_βH*H* Phe), 3.06 – 2.98 (m, 1H, C_β*H*H Phe), 2.57 (d, ^4J = 2.5 Hz, 1H, C≡CH), 2.27 (s, 3H, CH_3).

The carboxyl proton was not detectable in the chosen solvent.

^{13}C NMR (151 MHz, CD_3CN) δ = 170.95 (CO), 167.80 (CO), 165.60 (d, $^1J_{C,F}$ = 252.1 Hz, C-4 F_2Bz), 163.14 (CO), 161.68 (d, $^1J_{C,F}$ = 251.1 Hz, C-2 F_2Bz), 139.79, 138.92, 137.78, 133.96 - 133.75 (m, C-6 F_2Bz), 133.14, 131.18, 129.74, 129.56, 129.52, 129.23, 128.36, 127.44, 119.23 (dd, $^2J_{C,F}$ = 12.9 Hz, $^4J_{C,F}$ = 3.6 Hz, C-1 F_2Bz), 112.99 (dd, $^2J_{C,F}$ = 21.6 Hz, $^4J_{C,F}$ = 3.5 Hz, C-5 F_2Bz) 105.30 (dd, $^2J_{C,F}$ = 28.2 ≈ 26.2 Hz, C-3 F_2Bz), 81.92 (*C*≡CH), 73.01, 72.78, 72.44, 55.76 (C_α Phe), 41.87 (C_α Ser), 38.36 (C_β Phe), 21.35 (CH_3).

Due to overlapping one signal was not identified.

Diastereomeric purity > 99%.

***N*-(4-Fluorobenzoyl)-3-methyl-L-phenylalanyl-*O*-(3-carboxybenzyl)serine alkyne (2b)**

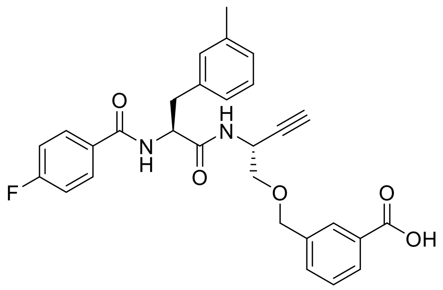

HR-MS ESI (+):

m/z = 503.1976 ([M+H]$^+$)

M ($C_{29}H_{28}FN_2O_5^+$, monoisotopic): 503.1977

The synthesis was accomplished according to GP VI. using 0.09 g **53b** (0.17 mmol 1 eq.), 149 µL morpholine (1.73 mmol, 10 eq.), 0.05 g $Pd(PPh_3)_4$ (0.04 mmol 0.02 eq.) and 15 mL CH_2Cl_2. The crude product was purified *via* semipreparative HPLC. *N*-(4-Fluorobenzoyl)-3-methyl-L-phenylalanyl-*O*-(3-carboxybenzyl)serine alkyne (**2b**) was obtained as a white solid (55 mg, 0.09 mmol, 56%).

^{1}H NMR (400 MHz, DMSO-*d_6*) δ = 8.65 (d, ^3J = 8.3 Hz, 1H, NH Phe), 8.56 (d, ^3J = 8.5 Hz, 1H, NH Ser), 7.93 - 7.90 (m, 1H, aromatic), 7.89 - 7.83 (m, 3H, aromatic), 7.62 -

7.57 (m, 1H, aromatic), 7.44 (t, ^3J = 7.7 Hz, 1H, aromatic), 7.30 - 7.24 (m, 2H, aromatic), 7.18 - 7.09 (m, 3H, aromatic), 6.98 - 6.93 (m, 1H, aromatic), 4.86 - 4.78 (m, 1H, $C_\alpha H$ Phe), 4.72 - 4.65 (m, 1H, $C_\alpha H$ Ser), 4.62 (s, 2H, Ar-CH_2-O), 3.58 (d, ^3J = 6.2 Hz, 2H, $C_\beta H_2$ Ser), 3.24 (d, ^4J = 2.4 Hz, 1H, C≡CH), 3.06 - 2.88 (m, 2H, $C_\beta H_2$ Phe), 2.23 (s, 3H, CH_3).

The carboxyl proton was not detectable in the chosen solvent.

^{13}C NMR (101 MHz, DMSO-*d6*) δ = 171.36 (CO Phe), 167.65 (COOH), 165.58 (CO FBz), 164.33 (d, $^1J_{C,F}$ = 250.5 Hz, CF), 139.07, 138.53, 137.40, 132.38, 131.20, 130.92 (d, $^4J_{C,F}$ = 2.9 Hz, C-1 FBz), 130.51 (d, $^3J_{C,F}$ = 9.0 Hz, C-2/6 FBz), 130.28, 128.99, 128.86, 128.73, 128.33, 127.34, 126.72, 115.51 (d, $^2J_{C,F}$ = 21.7 Hz, C-3/5 FBz), 82.33 (*C*≡CH), 74.36 (C≡*C*H), 72.08, 71.69, 55.35 (C_α Ser), 40.99 (C_α Phe), 37.55, 21.46 (CH_3).

Diastereomeric purity > 94%

_N_-Diphenylacetyl-3-methyl-L-phenylalanyl-_O_-(3-carboxybenzyl)-L-serine alkyne (2c)

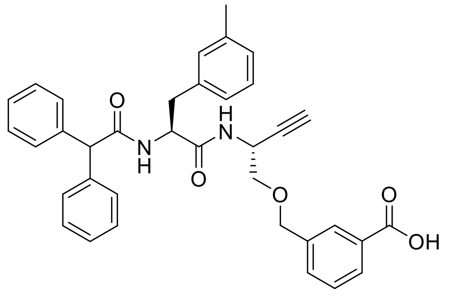

HR-MS ESI (+):

m/z = 575.2538 ($[M+H]^+$)

M ($C_{36}H_{35}N_2O_5^+$, monoisotopic): 575.254

The synthesis was accomplished according to GP VI. using 0.11 g **53c** (0.09 mmol, 1 eq.), 81 µL morpholine (0.92 mmol, 10 eq.), 0.02 g $Pd(PPh_3)_4$ (0.02 mmol, 0.2 eq.) and 10 mL CH_2Cl_2. The crude product was purified *via* semipreparative HPLC. *N*-Diphenylacetyl-3-methyl-L-phenylalanyl-*O*-(3-carboxybenzyl)-L-serine alkyne (**2c**) was obtained as a white solid (16.1 mg, 0.03 mmol, 30%).

^{1}H NMR (400 MHz, CD_3CN) δ = 7.97 – 7.94 (m, 1H, H-2 BnCOO), 7.94 – 7.90 (m, 1H, H-4 BnCOO), 7.58 – 7.53 (m, 1H, H-6 BnCOO), 7.45 (t, ^3J = 7.5 Hz, 1H, H-5 BnCOO), 7.30 – 7.07 (m, 11H, NH, aromatic diphenyl), 7.02 (d, ^3J = 7.6 Hz, 1H, aromatic Phe), 6.97 – 6.90 (m, 3H, aromatic Phe), 6.86 (d, ^3J = 7.9 Hz, 1H, NH), 4.90 (s, 1H, CH diphenyl), 4.87 – 4.79 (m, 1H, $C_\alpha H$ Phe), 4.64 – 4.57 (m, 1H, $C_\alpha H$ Ser), 4.57 – 4.49 (m, 2H, CH_2O), 3.54 (d, ^3J = 5.5 Hz, 2H, $C_\beta H_2$ Ser), 3.09 – 3.01 (m, 1H, $C_\beta H\mathit{H}$ Phe), 2.86 – 2.77 (m, 1H, $C_\beta \mathit{H}H$ Phe), 2.55 (d, ^4J = 2.4 Hz, 1H, C≡CH), 2.24 (s, 3H, CH_3).

The carboxyl proton was not detectable in the chosen solvent.

^{13}C NMR (101 MHz, CD_3CN) δ = 172.47 (CO), 171.11 (CO), 167.77 (CO), 140.80, 140.76, 139.85, 138.84, 137.92, 133.26, 131.09, 131.02, 129.78, 129.67, 129.61, 129.60, 129.58, 129.42, 129.40, 129.21, 128.27, 127.89, 127.80, 127.35, 81.98

(C≡CH), 72.96, 72.72, 72.44, 58.48 (CH diphenyl), 55.29 ($C_α$ Phe), 41.79 ($C_α$ Ser), 38.09 ($C_β$ Phe), 21.43 (CH_3).

Diastereomeric purity > 99%

***N*-(4-Phenylbenzoyl)-3-methyl-L-phenylalanyl-*O*-(3-carboxybenzyl)-L-serine alkyne (2d)**

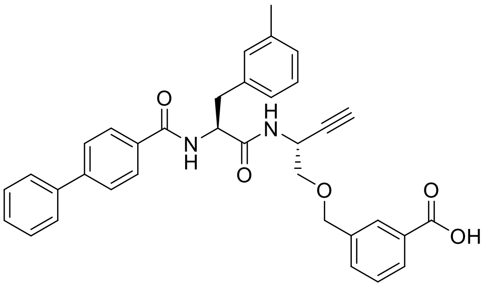

HR-MS ESI (+):

m/z = 583.2204 ($[M+Na]^+$)

M ($C_{35}H_{32}N_2NaO_5$, monoisotopic): 583.2203

The synthesis was accomplished according to GP VI. using 0.13 g **53d** (0.02 mmol, 1 eq.), 19 µL morpholine (0.22 mmol, 10 eq.), 0.01 g $Pd(PPh_3)_4$ (0.004 mmol, 0.2 eq.) and 10 mL CH_2Cl_2. The crude product was purified *via* semipreparative HPLC. *N*-(4-Phenylbenzoyl)-3-methyl-L-phenylalanyl-*O*-(3-carboxybenzyl)-L-serine alkyne (**2d**) was obtained as a white solid (8.0 mg, 0.01 mmol, 66%).

^{1}H NMR (600 MHz, CD_3CN) δ = 7.96 (s, 1H, H-2 BnCOO), 7.89 – 7.86 (m, 1H, H-4 BnCOO), 7.78 – 7.75 (m, 2H, H aromatic), 7.68 – 7.64 (m, 4H, H aromatic), 7.54 (d, ^3J = 7.6 Hz, 1H, H-6 BnCOO), 7.50 – 7.46 (m, 2H, H aromatic), 7.43 – 7.36 (m, 2H, H aromatic), 7.30 (d, ^3J = 8.0 Hz, 1H, NH), 7.20 (d, ^3J = 8.4 Hz, 1H, NH), 7.18 – 7.13 (m, 2H, H aromatic Phe), 7.10 (d, ^3J = 7.3 Hz, 1H, H aromatic Phe), 7.03 (d, ^3J = 7.5 Hz, 1H, H aromatic Phe), 4.95 – 4.89 (m, 1H, $C_αH$ Ser), 4.81 – 4.75 (m, 1H, $C_αH$ Phe), 4.63 – 4.56 (m, 2H, CH_2O), 3.62 (d, ^3J = 5.6 Hz, 2H, $C_βH_2$ Ser), 3.28 – 3.20 (m, 1H, $C_βH$*H* Phe), 3.08 – 2.98 (m, 1H, $C_β$*H*H Phe), 2.55 (d, ^4J = 2.4 Hz, 1H, C≡CH), 2.27 (s, 3H, CH_3).

The carboxyl proton was not detectable in the chosen solvent.

^{13}C NMR (151 MHz, CD_3CN) δ = 171.52 (CO), 167.85 (CO), 167.64 (CO), 144.90, 140.68, 139.83, 138.91, 138.52, 133.71, 133.12, 131.06, 129.95, 129.71, 129.52, 129.50, 129.23, 129.05, 128.72, 128.23, 128.02, 127.86, 127.31, 82.02 (C≡CH), 72.96, 72.72, 72.50, 55.94 ($C_α$ Phe), 41.85 ($C_α$ Ser), 38.02 ($C_β$ Phe), 21.40 (CH_3).

Due to overlapping one signal was not identified.

Diastereomeric purity > 92%

N-(4-Iodobenzoyl)-3-methyl-L-phenylalanyl-O-(3-carboxybenzyl)-L-serine alkyne (2e)

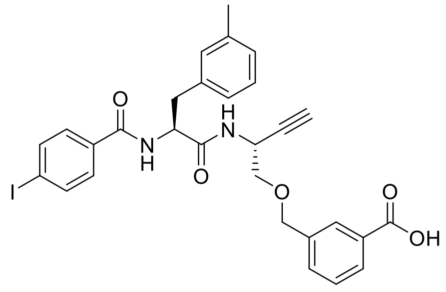

HR-MS ESI (+):

m/z = 633.0853 ($[M+Na]^+$)

M ($C_{29}H_{27}IN_2NaO_5^+$, monoisotopic): 633.0857

The synthesis was accomplished according to GP VI. using 0.04 g **53e** (0.07 mmol, 1 eq.), 57 µL morpholine (0.66 mmol, 10 eq.), 0.02 g $Pd(PPh_3)_4$ (0.01 mmol, 0.1 eq.) and 10 mL CH_2Cl_2. The crude product was purified *via* semipreparative HPLC. *N*-(4-Iodobenzoyl)-3-methyl-L-phenylalanyl-*O*-(3-carboxybenzyl)-L-serine alkyne (**2e**) was obtained as a white solid (11.0 mg, 0.02 mmol, 27%).

^{1}H NMR (600 MHz, CD_3CN) δ = 7.94 (s, 1H, H-2 BnCOO), 7.91 – 7.87 (m, 1H, H-4 BnCOO), 7.79 – 7.76 (m, 2H, H-3,5 IBz), 7.55 – 7.52 (m, 1H, H-6 BnCOO), 7.45 – 7.38 (m, 3H, H-5 2BnCOO, H-2,6 IBz), 7.27 – 7.24 (m, 1H, NH), 7.18 – 7.12 (m, 2H, NH, H aromatic Phe), 7.11 – 7.08 (m, 1H, H aromatic Phe), 7.09 – 7.04 (m, 1H, H aromatic Phe), 7.03 – 7.00 (m, 1H, H aromatic Phe), 4.93 – 4.87 (m, 1H, C_αH Ser), 4.75 – 4.69 (m, 1H, C_αH Phe), 4.62 – 4.54 (m, 2H, CH_2O), 3.61 (d, ^3J = 5.7 Hz, 2H, $C_\beta H_2$ Ser), 3.24 – 3.18 (m, 1H, C_βH*H* Phe), 3.01 – 2.93 (m, 1H, C_β*H*H Phe), 2.55 (d, ^4J = 2.4 Hz, 1H, C≡CH), 2.26 (s, 3H, CH_3).

The carboxyl proton was not detectable in the chosen solvent.

^{13}C NMR (151 MHz, CD_3CN) δ = 173.02 (CO), 169.45 (CO), 168.92 (CO), 141.53, 140.60, 140.26, 140.07, 136.21, 134.84, 132.73, 131.57, 131.41, 131.23, 131.18, 130.92, 129.93, 128.98, 100.62 (CI), 83.65 (*C*≡CH), 74.64, 74.42, 74.17, 57.63 (C_α Phe), 43.54 (C_α Ser), 39.72 (C_β Phe), 23.07 (CH_3).

Due to overlapping one signal was not identified.

Diastereomeric purity > 95%

N-(3-Iodobenzoyl)-3-methyl-L-phenylalanyl-O-(3-carboxybenzyl)-L-serine alkyne (2f)

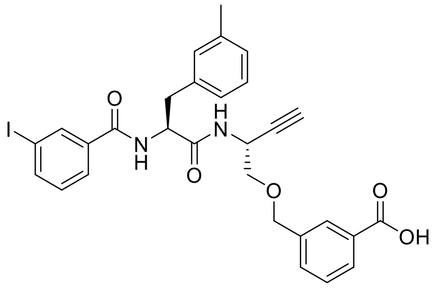

HR-MS ESI (+):

m/z = 633.0855 ([M+Na]+)

M ($C_{29}H_{27}IN_2NaO_5^+$, monoisotopic): 633.0857

The synthesis was accomplished according to GP VI. using 0.03 g **53f** (0.10 mmol, 1 eq.), 88 µL morpholine (1.01 mmol, 10 eq.), 0.02 g $Pd(PPh_3)_4$ (0.02 mmol, 0.2 eq.) and 10 mL CH_2Cl_2. The crude product was purified *via* semipreparative HPLC. *N-(*3-Iodobenzoyl)-3-methyl-L-phenylalanyl-*O*-(3-carboxybenzyl)-L-serine alkyne (**2f**) was obtained as a white solid (20.4 mg, 0.03 mmol, 33%).

^{1}H NMR (400 MHz, CD_3CN) δ = 8.01 (t, ^4J = 1.8 Hz, 1H, H-2 IBz), 7.96 – 7.93 (m, 1H, H-2 BnCOO), 7.90 – 7.82 (m, 2H, H-4,6 IBz), 7.66 (d, ^3J = 7.8 Hz, 1H, H-4 BnCOO), 7.54 (d, ^3J = 7.8 Hz, 1H, H-6 BnCOO), 7.41 (t, ^3J = 7.6 Hz, 1H, H-5 BnCOO), 7.24 (d, ^3J = 8.0 Hz, 1H, NH), 7.21 – 7.01 (m, 6H, NH, H-5 IBz, aromatic Phe), 4.95 – 4.86 (m, 1H, C_αH Phe), 4.76 – 4.68 (m, 1H, C_αH Ser), 4.64 – 4.54 (m, 2H, CH_2O), 3.62 (d, ^3J = 5.7 Hz, 2H, $C_\beta H_2$ Ser), 3.25 – 3.16 (m, 1H, C_βH*H* Phe), 3.01 – 2.92 (m, 1H, C_β*H*H Phe), 2.55 (d, ^4J = 2.4 Hz, 1H, C≡CH), 2.28 (s, 3H, CH_3).

The carboxyl proton was not detectable in the chosen solvent.

^{13}C NMR (101 MHz, CD_3CN) δ = 171.27 (CO), 167.74 (COO), 166.50 (CO), 141.34, 139.86, 138.92, 138.43, 137.04, 133.15, 131.35, 131.08, 129.74, 129.57, 129.49, 129.26, 128.26, 127.56, 127.28, 94.37 (CI), 81.98 (*C*≡CH), 72.96, 72.72, 72.50, 56.00 (C_α Phe), 41.85 (C_α Ser), 37.97 (C_β Phe), 21.43 (CH_3).

Due to overlapping two signals were not identified.

Diastereomeric purity > 95%

N-Benzoyl-3-methyl-L-phenylalanyl-O-(3-carboxybenzyl)-L-serine alkyne (2g)

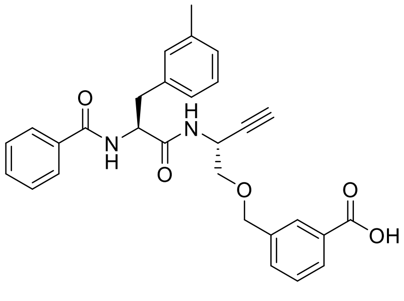

HR-MS ESI (+):

m/z = 507.1888 ([M+Na]+)

M ($C_{29}H_{28}N_2NaO_5^+$, monoisotopic): 507.1890

The synthesis was accomplished according to GP VI. using 0.02 g **53g** (0.04 mmol, 1 eq.), 36 µL morpholine (0.41 mmol, 10 eq.), 0.01 g $Pd(PPh_3)_4$ (0.01 mmol, 0.25 eq.) and 10 mL CH_2Cl_2. The crude product was purified *via* semipreparative HPLC. *N*-Benzoyl-3-methyl-L-phenylalanyl-*O*-(3-carboxybenzyl)-L-serine alkyne (**2g**) was obtained as a white solid (7.2 mg, 0.01 mmol, 36%).

^{1}H NMR (400 MHz, CD_3CN) δ = 7.96 (s, 1H, H-2 BnCOO), 7.89 (d, ^3J = 7.8 Hz, 1H, H-4 BnCOO), 7.72 – 7.67 (m, 2H, H-2,6 Bz), 7.56 – 7.48 (m, 2H, H-4 Bz, H-6 BnCOO), 7.44 – 7.37 (m, 3H, H-5 BnCOO, H-4 Bz), 7.28 – 6.99 (m, 6H, 2 NH, aromatic Phe), 4.96 – 4.86 (m, 1H, C_αH Phe), 4.80 – 4.72 (m, 1H, C_αH Ser), 4.64 – 4.53 (m, 2H, CH_2O), 3.61 (d, ^3J = 5.6 Hz, 2H, $C_\beta H_2$ Ser), 3.25 – 3.17 (m, 1H, $C_\beta H\mathit{H}$ Phe), 3.04 – 2.95 (m, 1H, $C_\beta \mathit{H}$H Phe), 2.55 (d, ^4J = 2.4 Hz, 1H, C≡CH), 2.26 (s, 3H, CH_3).

The carboxyl proton was not detectable in the chosen solvent.

^{13}C NMR (101 MHz, CD_3CN) δ = 171.52 (CO), 167.95 (CO), 167.92 (CO), 139.83, 138.90, 138.43, 134.93, 133.17, 132.57, 131.16, 131.06, 129.73, 129.55, 129.40, 129.22, 128.23, 128.07, 127.31, 82.00 (*C*≡CH), 72.97, 72.71, 72.46, 55.88 (C_α Phe), 41.85 (C_α Ser), 38.09 (C_β Phe), 21.37 (CH_3).

Due to overlapping one signal was not identified.

Diastereomeric purity > 97%

*N-(*3-Fluorobenzoyl)-3-methyl-L-phenylalanyl-*O*-(3-carboxybenzyl)-L-serine alkyne (2h)

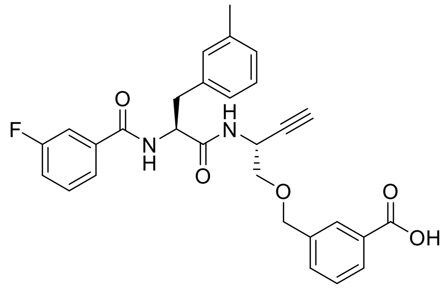

HR-MS ESI (+):

m/z = 503.1976 ($[M+H]^+$)

M ($C_{29}H_{28}FN_2O_5^+$, monoisotopic): 503.1977

The synthesis was accomplished according to GP VI. using 0.02 g **53h** (0.04 mmol, 1 eq.), 36 µL morpholine (0.41 mmol, 10 eq.), 0.01 g $Pd(PPh_3)_4$ (0.01 mmol, 0.25 eq.) and 10 mL CH_2Cl_2. The crude product was purified *via* semipreparative HPLC. *N-(*3-Fluorobenzoyl)-3-methyl-L-phenylalanyl-*O*-(3-carboxybenzyl)-L-serine alkyne (**2h**) was obtained as a white solid (7.7 mg, 0.02 mmol, 37%).

^{1}H NMR (400 MHz, CD_3CN) δ = 7.95 (s, 1H, H-2 BnCOO), 7.89 (d, ^3J = 7.7 Hz, 2H, H-4 BnCOO), 7.56 – 7.46 (m, 2H, H-6 BnCOO, H-6 FBz), 7.46 – 7.36 (m, 3H, H-5 BnCOO, H-2,5 FBz), 7.32 (d, ^3J = 8.1 Hz, 1H, NH), 7.28 – 7.21 (m, 1H, H-4 FBz), 7.20 – 7.05 (m, 4H, NH, H-2,4,5 aromatic Phe), 7.02 (d, ^3J = 7.4 Hz, 1H, H-6 aromatic Phe), 4.94 – 4.84 (m, 1H, C_αH Ser), 4.80 – 4.68 (m, 1H, C_αH Phe), 4.64 – 4.51 (m, 2H,

CH_2O), 3.61 (d, ^3J = 5.7 Hz, 2H, $C_βH_2$ Ser), 3.24 – 3.16 (m, 1H, $C_βHH$ Phe), 3.04 – 2.93 (m, 1H, $C_βHH$ Phe), 2.55 (d, ^4J = 2.4 Hz, 1H, C≡CH), 2.26 (s, 3H, CH_3).

The carboxyl proton was not detectable in the chosen solvent.

^{13}C NMR (101 MHz, CD_3CN) δ = 171.34 (CO), 167.91 (COO), 166.64 (CO), 163.46 (d, $^1J_{C,F}$ = 244.8 Hz, CF), 139.82, 138.91, 138.36, 137.32 (d, $^3J_{C,F}$ = 6.9 Hz, C-1 FBz), 133.12, 131.45, 131.37, 131.19, 131.05, 129.72, 129.52, 129.24, 128.25, 127.30, 123.94 (d, $^4J_{C,F}$ = 2.9 Hz, C-6 FBz), 119.33 ($^2J_{C,F}$ = 21.4 Hz, C-4 FBz), 115.12 (d, $^2J_{C,F}$ = 23.2 Hz, C-2 FBz), 81.96 (*C*≡CH), 72.96, 72.74, 72.46, 56.00 ($C_α$ Phe), 41.86 ($C_α$ Ser), 38.09 ($C_β$ Phe), 21.36 (CH_3).

Diastereomeric purity > 99%

***N*-(3-Bromobenzoyl)-3-methyl-L-phenylalanyl-*O*-(3-carboxybenzyl)-L-serine alkyne (2i)**

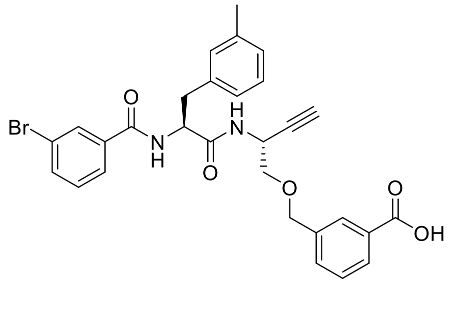

HR-MS ESI (+):

m/z = 585.0994 ([M+Na]$^+$)

M ($C_{29}H_{27}BrN_2NaO_5^+$, monoisotopic): 585.0995

The synthesis was accomplished according to GP VI. using 0.02 g **53i** (0.04 mmol, 1 eq.), 33 µL morpholine (0.38 mmol, 4 eq.), 0.01 g $Pd(PPh_3)_4$ (0.01 mmol, 0.25 eq.) and 10 mL CH_2Cl_2. The crude product was purified *via* semipreparative HPLC. *N*-(3-Bromobenzoyl)-3-methyl-L-phenylalanyl-*O*-(3-carboxybenzyl)-L-serine alkyne (**2i**) was obtained as a white solid (5.6 mg, 0.01 mmol, 26%).

^{1}H NMR (400 MHz, CD_3CN) δ = 7.95 (s, 1H, H-2 aromatic), 7.89 (d, ^3J = 7.7 Hz, 1H, H-4 aromatic), 7.82 (t, ^4J = 1.8 Hz, 1H, H-2 BrBz), 7.67 – 7.62 (m, 2H, H-4,6 BrBz), 7.56 – 7.52 (m, 1H, H-6 aromatic), 7.40 (t, ^3J = 7.7 Hz, 1H, H-5 aromatic), 7.36 – 7.29 (m, 2H, H-5 BrBz, NH), 7.21 – 7.06 (m, 4H, H-2,5,6 Phe), 7.02 (d, ^3J = 7.4 Hz, 1H, H-4 Phe), 4.95 – 4.87 (m, 1H, $C_αH$ Ser), 4.78 – 4.70 (m, 1H, $C_αH$ Phe), 4.64 – 4.53 (m, 2H, CH_2O), 3.62 (d, ^3J = 5.7 Hz, 2H, $C_βH_2$ Ser), 3.25 – 3.17 (m, 1H, $C_βHH$ Phe), 3.01 – 2.93 (m, 1H, $C_βHH$ Phe), 2.55 (d, ^4J = 2.4 Hz, 1H, C≡CH), 2.27 (s, 3H, CH_3).

The carboxyl proton was not detectable in the chosen solvent.

^{13}C NMR (101 MHz, CD_3CN) δ = 171.36 (COOH), 167.89 (CO), 166.57 (CO), 139.83, 138.91, 138.38, 137.10, 135.33, 133.13 (C-6 BnCOO), 131.34, 131.16, 131.08, 129.73, 129.54, 129.50, 129.25, 128.26 (C-4 Phe), 127.29, 127.00, 122.90, 105.88, 81.95, 72.97 (CH_2O), 72.75, 72.48 ($C_β$ Ser), 56.03 ($C_α$ Phe), 41.87 ($C_α$ Ser), 38.04 ($C_β$ Phe), 21.39 (CH_3).

Diastereomeric purity > 95%

N-3-(Trifluoromethyl)benzoyl-3-methyl-L-phenylalanyl-O-(3-carboxybenzyl)-L-serine alkyne (2j)

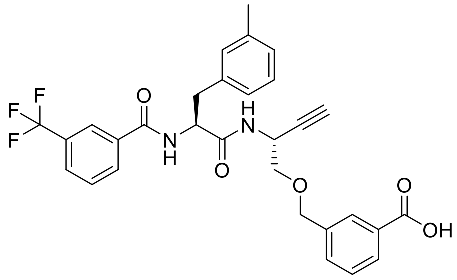

HR-MS ESI (+):

m/z = 575.1763 ($[M+Na]^+$)

M ($C_{30}H_{27}F_3N_2NaO_5^+$, monoisotopic): 575.1764

The synthesis was accomplished according to GP VI. using 0.02 g **53i** (0.04 mmol, 1 eq.), 32 µL morpholine (0.37 mmol, 9.3 eq.), 0.01 g $Pd(PPh_3)_4$ (0.01 mmol, 0.25 eq.) and 10 mL CH_2Cl_2. The crude product was purified *via* semipreparative HPLC. *N*-3-(Trifluoromethyl)benzoyl-3-methyl-L-phenylalanyl-*O*-(3-carboxybenzyl)-L-serine alkyne (**2j**) was obtained as a white solid (8.7 mg, 0.02 mmol, 43%).

^{1}H NMR (400 MHz, CD_3CN) δ = 7.98 – 7.90 (m, 3H, aromatic BnCOO, aromatic F_3CBz), 7.88 (d, ^3J = 7.7 Hz, 1H, aromatic BnCOO), 7.80 (d, ^3J = 7.8 Hz, 1H, aromatic F_3CBz), 7.59 (t, ^3J = 7.8 Hz, 1H, aromatic F_3CBz), 7.54 (d, ^3J = 7.7 Hz, 1H, BnCOO), 7.46 (d, ^3J = 8.2 Hz, 1H, NH Phe), 7.39 (t, ^3J = 7.7 Hz, 1H, BnCOO), 7.20 (d, ^3J = 8.4 Hz, 1H, NH Ser), 7.17 – 7.07 (m, 3H, aromatic Phe), 7.02 (d, ^3J = 7.3 Hz, 1H, aromatic Phe), 4.95 – 4.88 (m, 1H, $C_\alpha H$ Ser), 4.81 – 4.74 (m, 1H, $C_\alpha H$ Phe), 4.63 – 4.54 (m, 2H, CH_2O), 3.62 (d, ^3J = 5.7 Hz, 2H, $C_\beta H_2$ Ser), 3.25 – 3.19 (m, 1H, $C_\beta \mathit{H}H$ Phe), 3.03 – 2.94 (m, 1H, $C_\beta H\mathit{H}$ Phe), 2.56 – 2.54 (m, 1H, C≡CH), 2.26 (s, 3H, CH_3).

The carboxyl proton was not detectable in the chosen solvent.

^{13}C NMR (101 MHz, CD_3CN) δ = 171.33 (CO), 167.89 (CO), 166.65 (CO), 139.83, 138.92, 138.37, 135.90, 133.13, 131.94, 131.17, 131.09, 131.06, 130.45, 129.71, 129.52, 129.49, 129.24, 129.03 (q, $^3J_{C,F}$ = 3.8 Hz, C-4 F_3CBz), 128.26, 127.29, 125.00 (q, $^1J_{C,F}$ = 271.6 Hz, CF_3), 124.98 (q, $^3J_{C,F}$ = 3.9 Hz, C-2 F_3CBz), 81.93 (*C*≡CH), 72.95 (CH_2O), 72.76, 72.47 (C_β Ser), 56.09 (C_α Phe), 41.88 (C_α Ser), 38.07 (C_β Phe), 21.34 (CH_3).

Diastereomeric purity > 94%

_N_-(4-Fluorobenzoyl)-3-iodo-L-phenylalanyl-_O_-(3-(allyloxycarbonyl)benzyl)-L-serine alkyne (2k)

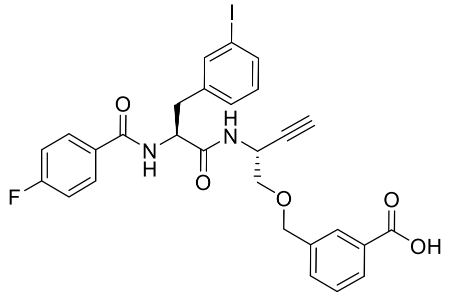

HR-MS ESI (+):

m/z = 637.0601 ([M+Na]$^+$)

M ($C_{28}H_{24}FIN_2NaO_5^+$, monoisotopic): 637.0606

The synthesis was accomplished according to GP VI. using 0.02 g **53k** (0.03 mmol, 1 eq.), 28 µL morpholine (0.32 mmol, 10 eq.), 0.07 g $Pd(PPh_3)_4$ (0.01 mmol, 0.3 eq.) and 10 mL CH_2Cl_2. The crude product was purified *via* semipreparative HPLC. *N*-(4-Fluorobenzoyl)-3-iodo-L-phenylalanyl-*O*-(3-(allyloxycarbonyl)benzyl)-L-serine alkyne (**2k**) was obtained as a white solid (3.9 mg, 0.01 mmol, 36%).

^{1}H NMR (400 MHz, CD_3CN) δ = 7.95 (s, 1H, H-2 aromatic BnCOO), 7.91 – 7.87 (m, 1H, H-4 aromatic BnCOO), 7.77 – 7.71 (m, 2H, H-3,5 aromatic FBz), 7.68 (s, 1H, H-2 aromatic Phe), 7.58 – 7.52 (m, 2H, H-4 aromatic Phe, H-6 aromatic BnCOO), 7.41 (t, ^3J = 7.7 Hz, 1H, H-5 aromatic BnCOO), 7.32 – 7.26 (m, 2H, NH Phe, H-6 aromatic Phe), 7.20 (d, ^3J = 8.3 Hz, 1H, NH Ser), 7.17 – 7.11 (m, 2H, H-2,6 aromatic FBz), 7.04 (t, ^3J = 7.8 Hz, 1H, H-5 aromatic Phe), 4.93 – 4.86 (m, 1H, C_αH Ser), 4.79 – 4.72 (m, 1H, C_αH Phe), 4.63 – 4.53 (m, 2H, CH_2O), 3.61 (d, ^3J = 5.6 Hz, 2H, $C_\beta H_2$ Ser), 3.24 – 3.17 (m, 1H, C_β*H*H Phe), 3.02 – 2.93 (m, 1H, C_βH*H* Phe), 2.56 (d, ^4J = 2.4 Hz, 1H, C≡CH).

The carboxyl proton was not detectable in the chosen solvent.

^{13}C NMR (101 MHz, CD_3CN) δ = 171.11 (CO Phe), 167.89 (COO), 166.94 (CO FBz), 165.62 (d, $^1J_{C,F}$ = 249.5 Hz, CF), 141.30, 139.83, 139.31 (C-2 Phe), 136.57 (C-4 Phe), 133.14 (C-6 BnCOO), 131.29, 131.25, 131.16, 130.81, 130.72, 129.87, 129.72, 129.53 (d, $^4J_{C,F}$ = 2.0 Hz, C-1 FBz), 116.23 (d, $^2J_{C,F}$ = 22.2 Hz, C-3/5 FBz), 94.62 (C-3 aromatic Phe), 81.93 (*C*≡CH), 72.97, 72.81, 72.44, 55.58 (C_α Phe), 41.88 (C_α Ser), 37.64 (C_β Phe).

Diastereomeric purity > 95%

_N_-Diphenylacetyl-3-iodo-L-phenylalanyl-_O_-(3-carboxybenzyl)-L-serine alkyne (2l)

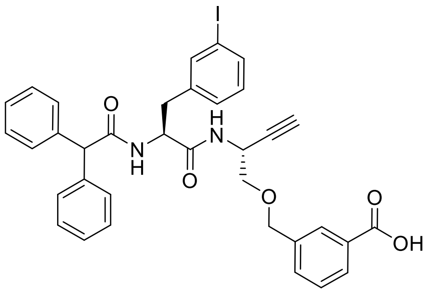

HR-MS ESI (+):

m/z = 687.1352 ([M+H]$^+$)

M ($C_{35}H_{32}IN_2O_5^+$, monoisotopic): 687.1351

The synthesis was accomplished according to GP VI. using 0.01 g **53l** (0.02 mmol, 1 eq.), 19 µL morpholine (0.22 mmol, 10 eq.), 0.001 g $Pd(PPh_3)_4$ (0.001 mmol, 0.05 eq.) and 10 mL CH_2Cl_2. The crude product was purified *via* semipreparative HPLC. *N*-Diphenylacetyl-3-iodo-L-phenylalanyl-*O*-(3-carboxybenzyl)-L-serine alkyne (**2l**) was obtained as a white solid (7.4 mg, 0.01 mmol, 50%).

^{1}H NMR (400 MHz, CD_3CN) δ = 7.96 – 7.90 (m, 2H, H-2,4 aromatic BnCOO), 7.60 – 7.54 (m, 3H, H aromatic), 7.45 (t, ^3J = 7.6 Hz, 1H, H-5 aromatic BnCOO), 7.31 – 7.08 (m, 11H, H aromatic), 7.05 – 6.92 (m, 3H, 2 NH, H aromatic), 4.90 (s, 1H, CH diphenylacetyl), 4.86 – 4.79 (m, 1H, $C_αH$ Ser), 4.69 – 4.61 (m, 1H, $C_αH$ Phe), 4.60 – 4.49 (m, 2H, CH_2O), 3.54 (d, ^3J = 5.5 Hz, 2H, $C_βH_2$ Ser), 3.10 – 3.04 (m, 1H, $C_β$*H*H Phe), 2.86 – 2.78 (m, 1H, $C_β$H*H* Phe), 2.56 (d, ^4J = 2.4 Hz, 1H, C≡CH).

The carboxyl proton was not detectable in the chosen solvent.

^{13}C NMR (101 MHz, CD_3CN) δ = 172.55 (CO), 170.85 (CO), 167.91 (COO), 140.82, 140.73, 140.68, 139.81, 139.18, 136.66, 133.22, 131.23, 131.18, 129.89, 129.78, 129.67, 129.60, 129.57, 129.46, 129.43, 127.90, 94.72 (C-2 aromatic Phe), 81.90 (*C*≡CH), 72.97 (CH_2O), 72.82 ($C_β$ Ser), 72.38 ($C_β$ Ser), 58.43 (CH diphenylacetyl), 54.92 ($C_α$ Phe), 41.83 ($C_α$ Ser), 37.64 ($C_β$ Phe).

Due to overlapping two signals were not identified.

Diastereomeric purity > 96%

_N_-(3-Iodobenzoyl)-3-iodo-L-phenylalanyl-_O_-(3-carboxybenzyl)-L-serine alkyne (2m)

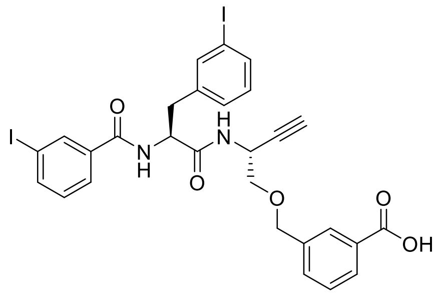

HR-MS ESI (+):

m/z = 744.9662 ([M+Na]$^+$)

M ($C_{28}H_{24}I_2N_2NaO_5^+$, monoisotopic): 744.9667

The synthesis was accomplished according to GP VI. using 0.01 g **53m** (0.01 mmol, 1 eq.), 7 µL morpholine (0.08 mmol, 8 eq.), 0.002 g $Pd(PPh_3)_4$ (0.002 mmol, 0.2 eq.) and 10 mL CH_2Cl_2. The crude product was purified *via* semipreparative HPLC. *N*-(3-Iodobenzoyl)-3-iodo-L-phenylalanyl-*O*-(3-carboxybenzyl)-L-serine alkyne (**2m**) was obtained as a white solid (2.2 mg, 0.003 mmol, 38%).

^{1}H NMR (600 MHz, CD_3CN) δ = 8.03 (t, ^4J = 1.7 Hz, 1H, H-2 IBz), 7.96 – 7.94 (m, 1H, H-2 BnCOO), 7.90 – 7.88 (m, 1H, H-4 BnCOO), 7.87 – 7.84 (m, 1H, H-4 IBz), 7.69 – 7.66 (m, 2H, H-6 IBz, H-2 Phe), 7.58 – 7.55 (m, 1H, H-4 Phe), 7.55 – 7.52 (m, 1H, H-6 BnCOO), 7.40 (t, ^3J = 7.7 Hz, 1H, H-5 BnCOO), 7.34 (d, ^3J = 8.2 Hz, 1H, NH), 7.31 – 7.28 (m, 1H, H-6 Phe), 7.23 – 7.17 (m, 2H, H-5 IBz, NH), 7.05 (t, ^3J = 7.8 Hz, 1H, H-5 Phe), 4.93 – 4.87 (m, 1H, C_αH Ser), 4.76 – 4.70 (m, 1H, C_αH Phe), 4.63 – 4.55 (m, 2H, CH_2O), 3.62 (d, ^3J = 5.7 Hz, 2H, $C_\beta H_2$ Ser), 3.23 – 3.18 (m, 1H, C_βH*H* Phe), 2.99 – 2.93 (m, 1H, C_β*H*H Phe), 2.56 (d, ^4J = 2.4 Hz, 1H, C≡CH).

The carboxyl proton was not detectable in the chosen solvent.

^{13}C NMR (151 MHz, CD_3CN) δ = 170.90 (COO), 167.82 (CO), 166.47 (CO), 141.34, 141.25, 139.76, 139.28, 137.00, 136.87, 136.52, 133.06, 131.31, 131.22, 131.12, 129.81, 129.68, 129.51, 129.44, 127.57, 94.59, 94.39, 81.86 (*C*≡CH), 72.89 (CH_2O), 72.76, 72.36 (C_β Ser), 55.59 (C_α Phe), 41.79 (C_α Ser), 37.48 (C_β Phe).

Diastereomeric purity > 95%

7 Synthesis of Inhibitors 56a – f (Scheme 7, top)

N-(*tert*-Butyloxycarbonyl)-3-methyl-L-phenylalanylglycine (54a)

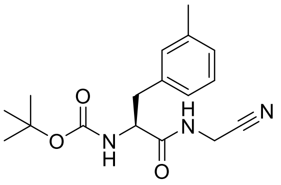

ESI (+):

m/z = 340.17 ([M+Na]$^+$)

M ($C_{17}H_{23}N_3NaO_3^+$, monoisotopic): 340.16

A solution of aminoacetonitrile sulfate (0.11 g, 1.07 mmol, 1 eq.) in water (1 mL) and sodium hydroxide (2 M, 1.07 mL, 2 eq.) was prepared. *N*-Boc-3-methyl-L-phenylalanine (0.30 g, 1.07 mmol, 1 eq.) and NMM (125 µL, 1.07 mmol, 1 eq.) were dissolved in dry THF (10 mL) and cooled to – 25 °C (*iso*-propanol/ liquid nitrogen). iBCF (0.18 mL, 1.07 mmol, 1 eq.) was added dropwise and a white precipitate formed. Then, the aqueous aminoacetonitrile sulfate solution was added. The brown two-face mixture was stirred 10 min at - 25°C and 30 min at room temperature. The reaction progress was monitored *via* thin layer chromatography. The solvent was evaporated and the residue dissolved in ethyl acetate (25 mL). The solution was washed with HCl (2 × 10 mL, 1 M), sat. $NaHCO_3$ (10 mL) and brine (10 mL). The organic layer was dried over Na_2SO_4 and the solvent evaporated. *N*-(*tert*-Butyloxycarbonyl)-3-methyl-L-phenylalanylglycine (**54a**) was obtained as a yellowish solid (0.31 g, 0.98 mmol, 91%).

^{1}H NMR (400 MHz, DMSO-d_6) δ = 7.21 (t, ^{3}J = 7.5 Hz, 1H, H-5 Phe), 7.10 - 7,04 (m, 1H, H-4 Phe), 7.03 - 6.96 (m, 2H, H-2,6 Phe), 6.47 - 6.38 (m, 1H, NH Gly), 5.04 - 4.38 (m, 1H, NH Phe), 4.33 (q, ^{3}J = 7.2 Hz, 1H, $C_{\alpha}H$ Phe), 4.16 - 4.00 (m, 2H, CH_2 Gly), 3.12 - 2.97 (m, 2H, $C_{\beta}H_2$ Phe), 2.33 (s, 3H, CH_3), 1.42 (s, 9H, $C(CH_3)_3$).

^{13}C NMR (101 MHz, DMSO-d_6) δ = 171.85 (CO Phe), 155.75 (CO Boc), 138.87 (C aromatic), 136.08 (C aromatic), 130.12 (C aromatic), 128.98 (C aromatic), 128.22 (C aromatic), 126.36 (C aromatic), 115.55 (C≡N), 77.36 ($\mathit{C}(CH_3)_3$), 56.06 (C_{α} Phe), 38.04 (C_{β} Phe), 28.42 ($C(\mathit{C}H_3)_3$), 27.45 (CH_2 Gly), 21.50 (CH_3).

N-(*tert*-Butyloxycarbonyl)-3-methyl-L-phenylalanylglycine alkyne (54b)

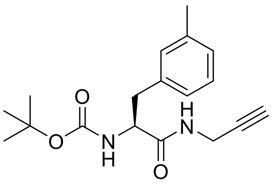

ESI (+):

m/z = 339.22 ($[M+Na]^+$)

M ($C_{18}H_{24}N_2NaO_3^+$, monoisotopic): 339.17

The synthesis was accomplished according to GP VIII. using 0.30 g *N*-Boc-3-methyl-L-phenylalanine (1.07 mmol, 1 eq.), 137 µL propargylamine (2.14 mmol, 2 eq.), 125 µL NMM (1.07 mmol, 1 eq.), 180 µL iBCF (1.07 mmol, 1 eq.) and 10 mL THF. *N*-(*tert*-Butyloxycarbonyl)-3-methyl-L-phenylalanylglycine alkyne (**54b**) was obtained as a white solid (0.30 g, 0.94 mmol, 88%).

^{1}H NMR (400 MHz, $CDCl_3$) δ = 7.19 (t, ^{3}J = 7.5 Hz, 1H, H-5 Phe), 7.10 – 6.94 (m, 3H, H-2,4,6 Phe), 5.98 (s, 1H, NH), 4.99 (s, 1H, NH), 4.30 (d, ^{3}J = 7.6 Hz, 1H, $C_{\alpha}H$ Phe), 4.03 – 3.92 (m, 2H, CH_2 Gly), 3.09 – 2.94 (m, 2H, $C_{\beta}H_2$ Phe), 2.32 (s, 3H, CH_3), 2.18 (t, ^{4}J = 2.6 Hz, 1H, C≡CH), 1.42 (s, 9H, $(CH_3)_3$).

^{13}C NMR (101 MHz, $CDCl_3$) δ = 171.13 (CO Phe), 155.55 (CO Boc), 138.55 (C aromatic), 136.52 (C aromatic), 130.22 (C aromatic), 128.79 (C aromatic), 127.95 (C aromatic), 126.47 (C aromatic), 79.19 ($\mathit{C}(CH_3)_3$), 77.36 (*C*≡CH), 71.77 (C≡*C*H), 55.98 (C_{β} Phe), 38.50 (C_{β} Phe), 29.24 (CH_2 Gly), 28.41 ($(CH_3)_3$), 21.51 (CH_3).

N-(*tert*-Butyloxycarbonyl)-L-leucylglycine alkyne (54c)

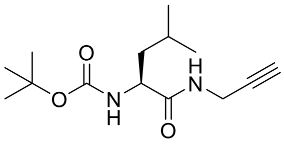

ESI (+):

m/z = 291.27 ($[M+Na]^+$)

M ($C_{14}H_{25}N_2O_3^+$, monoisotopic): 269.19

The synthesis was accomplished according to GP VIII. using 0.20 g *N*-Boc-L-leucine (0.86 mmol, 1 eq.), 111 µL propargylamine (1.73 mmol, 2 eq.), 95 µL NMM (0.86 mmol, 1 eq.), 112 µL iBCF (0.86 mmol, 1 eq.) and 10 mL THF. *N*-(*tert*-

Butyloxycarbonyl)-L-leucylglycine alkyne (**56c**) was obtained as a yellow oil (0.23 g, 0.84 mmol, 98%).

^{1}H NMR (400 MHz, $CDCl_3$) δ = 6.47 (s, 1H, NH Gly), 4.90 (d, ^3J = 8.3 Hz, 1H, NH Leu), 4.18 – 4.07 (m, 1H, $C_\alpha H$), 4.07 – 4.01 (m, 2H, CH_2 Gly), 2.22 (t, ^4J = 2.6 Hz, 1H, C≡CH), 1.73 – 1.58 (m, 2H, C*H*$(CH_3)_2$, C_β*H*H), 1.50 (d, ^3J = 8.9 Hz, 1H, C_βH*H*), 1.45 (s, 9H, $(CH_3)_3$), 0.97 – 0.92 (m, 6H, $(CH_3)_2$).

^{13}C NMR (101 MHz, $CDCl_3$) δ = 172.65 (CO), 156.05 (CO), 79.24 (*C*≡CH, *C*$(CH_3)_3$), 71.92 (C≡*C*H), 53.09 (C_α), 41.06 (C_β), 29.39 (CH_2 Gly), 28.43 ($(CH_3)_3$), 24.87 (*C*H$(CH_3)_2$), 23.08 ($(CH_3)_2$).

***N*-(*tert*-Butyloxycarbonyl)-3-cyclohexyl-L-alanylglycine alkyne (54d)**

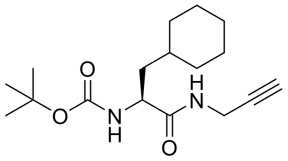

ESI (+):

m/z = 331.31 ([M+Na]$^+$)

M ($C_{17}H_{28}N_2NaO_3^+$, monoisotopic): 331.20

The synthesis was accomplished according to GP VIII. using 0.20 g *N*-Boc-3-(cyclohexyl)alanine (0.44 mmol, 1 eq.), 57 µL propargylamine (0.88 mmol, 2 eq.), 49 µL NMM (0.44 mmol, 1 eq.), 57 µL iBCF (0.44 mmol, 1 eq.) and 10 mL THF. *N*-(*tert*-Butyloxycarbonyl)-3-cyclohexyl-L-alanylglycine alkyne (**54d**) was obtained as a white solid (0.16 g, 0.31 mmol, 70%).

^{1}H NMR (400 MHz, $CDCl_3$) δ = 6.58 (s, 1H, NH Gly), 4.91 (d, ^3J = 8.1 Hz, 1H, NH Cha), 4.15 (s, 1H, $C_\alpha H$), 4.07 – 3.95 (m, 2H, CH_2 Gly), 2.21 (t, ^4J = 2.5 Hz, 1H, C≡CH), 1.81 – 1.58 (m, 7H, C_β*H*H, CH_2 Cha), 1.44 (s, 10H, C_βH*H*, $(CH_3)_3$), 1.39 – 1.29 (m, 1H, CH Cha), 1.29 – 0.81 (m, 4H, CH_2 Cha).

^{13}C NMR (101 MHz, $CDCl_3$) δ = 172.64 (CO Cha), 155.99 (C=), 80.42 (*C*≡CH), 79.42 (*C*$(CH_3)_3$), 71.75 (C≡*C*H), 52.39 (C_α), 39.82 (C_β), 34.19 (CH Cha), 33.79 (CH_2 Cha), 32.72 (CH_2 Cha), 29.27 (CH_2 Gly), 28.45 ($(CH_3)_3$), 26.53 (CH_2 Cha), 26.36 (CH_2 Cha), 26.19 (CH_2 Cha).

3-Methyl-L-phenylalanylglycine nitrile (55a)

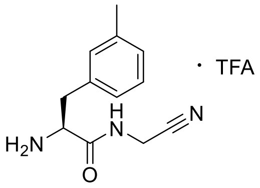

ESI (+):

m/z = 218.20 ([M+H]$^+$)

M ($C_{12}H_{16}N_3O^+$, monoisotopic): 218.13

The synthesis was accomplished according to GP II. using 0.30 g **54a** (0.96 mmol) and 10 mL TFA/CH_2Cl_2 (1:1). 3-Methyl-L-phenylalanylglycine nitrile (**55a**) was obtained as a yellow oil (0.41 g, quantitative yield; contained TFA).

^{1}H NMR (400 MHz, DMSO-*d*$_6$) δ = 9.11 (t, ^3J = 5.6 Hz, 1H, NH), 8.15 (m, 3H, NH_3^+), 7.23 - 7.16, (m, 1H, H-5 aromatic), 7.10 - 6.97 (m, 3H, H-2,3,4 aromatic), 4.20 (d, ^3J = 5.6 Hz, 1H, C*H*H Gly), 4.12 - 3.94 (m, 1H, C_α Phe), 3.79 - 3.62 (m, 1H, CH*H* Gly), 3.09 - 2.84 (m, 2H, $C_\beta H_2$ Phe), 2.27 (s, 3H, CH_3).

^{13}C-NMR (101 MHz, DMSO-*d*$_6$) δ = 169.19 (CO), 138.14, 134.79, 130.48, 128.95, 128.38, 126.94, 117.36 (C≡N), 53.85 (C_αH), 37.18 (C_βH), 27.53 (CH_2 Gly), 21.43 (CH_3).

3-Methyl-L-phenylalanylglycine alkyne (55b)

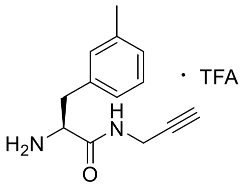

ESI (+):

m/z = 217.16 ([M+H]$^+$)

M ($C_{13}H_{17}N_2O^+$, monoisotopic): 217.13

The synthesis was accomplished according to GP II. using 0.29 g **54b** (0.91 mmol) and 10 mL TFA/CH_2Cl_2 (1:1). 3-Methyl-L-phenylalanylglycine alkyne (**55b**) was obtained as an orange solid (0.32 g, quantitative yield; contained TFA).

^{1}H NMR (400 MHz, DMSO-*d*$_6$) δ = 8.86 (t, ^3J = 5.4 Hz, 1H, NH), 8.17 (s, 3H, NH_3^+), 7.21 (t, ^3J = 7.5 Hz, 1H, H-5 aromatic), 7.06 - 7,12 (m, 1H, H-2 aromatic), 7.05 - 6.99 (m, 2H, H-4,6 aromatic), 3.92 (m, 3H, CH*2* Gly, C_αH Phe), 3.22 (t, ^4J = 2.6 Hz, 1H, C≡CH), 3.05 - 2.87 (m, 2H, $C_\beta H_2$ Phe), 2.29 (s, 3H, CH*3*).

^{13}C NMR (101 MHz, DMSO-*d*$_6$) δ = 167.68 (CO), 137.56 (C-1 aromatic), 134.54 (C-3 aromatic), 130.08 (C-2 aromatic), 128.43 (C-5 aromatic), 127.82 (C-6 aromatic), 126.54 (C-4 aromatic), 80.03 (*C*≡CH), 73.75 (C≡*C*H), 53.44 (C_α Phe), 36.84 (C_β Phe), 28.13 (*C*H_2 Gly), 21.02 (CH_3).

L-Leucylglycine alkyne (55c)

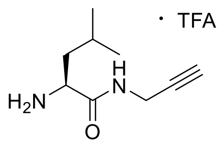

ESI (+):

m/z = 169.13 ([M+H]$^+$)

M ($C_9H_{17}N_2O^+$, monoisotopic): 169.13

The synthesis was accomplished according to GP II. using 0.21 g **54c** (0.79 mmol) and 10 mL TFA/CH_2Cl_2 (1:1). L-Leucylglycine alkyne (**55c**) was obtained as a yellow oil (0.22 g, 1.02 mmol, quantitative yield; contained TFA).

^{1}H NMR (400 MHz, DMSO-*d6*) δ = 8.95 (t, ^{3}J = 5.4 Hz, 1H, NH), 8.15 (s, 3H, NH_3^+), 3.95 (dd, ^{3}J = 5.4 Hz, ^{4}J = 2.6 Hz, 2H, CH_2 Gly), 3.78 – 3.68 (m, 1H, $C_\alpha H$), 3.21 (t, ^{4}J = 2.5 Hz, 1H, C≡CH), 1.70 – 1.58 (m, 1H, C*H*(CH_3)$_2$), 1.57 – 1.49 (m, 2H, $C_\beta H_2$), 0.93 – 0.85 (m, 6H, (CH_3)$_2$).

^{13}C NMR (101 MHz, DMSO-*d6*) δ = 168.74 (CO), 80.20 (*C*≡CH), 73.70 (C≡*C*H), 50.82 (C_α), 39.53 (solvent, C_β), 28.16 (CH_2 Gly), 23.56 (*C*H(CH_3)(CH_3)), 22.50 (CH(*C*H$_3$)(CH_3)), 21.91 (CH(CH_3)(*C*H$_3$)).

3-Cyclohexyl-L-alanylglycine alkyne (55d)

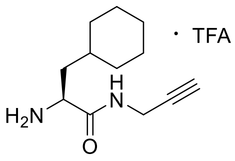

ESI (+):

m/z = 209.27 ([M+H]$^+$)

M ($C_{12}H_{21}N_2O^+$, monoisotopic): 209.16

The synthesis was accomplished according to GP II. using 0.16 g **54d** (0.52 mmol) and 10 mL TFA/CH_2Cl_2 (1:1). 3-Cyclohexyl-L-alanylglycine alkyne (**55d**) was obtained as a yellow oil (0.13 g, quantitative yield; contained TFA).

^{1}H NMR (400 MHz, DMSO-*d6*) δ = 8.93 (t, ^{3}J = 5.4 Hz, 1H, NH), 8.14 (s, 3H, NH_3^+), 4.02 – 3.86 (m, 2H, CH_2 Gly), 3.79 – 3.69 (m, 1H, $C_\alpha H$), 3.21 (t, ^{4}J = 2.5 Hz, 1H, C≡CH), 1.75 – 1.50 (m, 7H, CH_2 Cha), 1.37 – 1.26 (m, 1H, CH Cha), 1.25 – 1.04 (m, 3H, CH_2 Cha), 0.96 – 0.78 (m, 2H, CH_2 Cha).

^{13}C NMR (101 MHz, DMSO-*d6*) δ = 168.81 (CO), 80.21 (*C*≡CH), 73.66 (C≡*C*H), 50.23 (C_α), 38.68, 32.67, 32.56, 32.10, 28.12 (CH_2 Gly), 25.79, 25.58, 25.40.

***N*-(4-Fluorobenzoyl)-3-methyl-L-phenylalanylglycine nitrile (56a)**

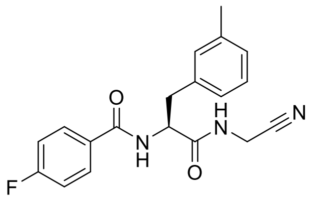

ESI (+):

m/z = 362.1275 ([M+Na]$^+$)

M ($C_{19}H_{18}FN_3NaO_2$, monoisotopic): 362.1275

The synthesis was accomplished according to GP IV. using 0.02 g **55a** (0.06 mmol, 1 eq.), 26 µL TEA (0.19 mmol, 3.2 eq.), 7 µL 4-fluorobenzoyl chloride (0.06 mmol, 1 eq.) and 5 mL CH_2Cl_2. The crude product was purified *via* semipreparative HPLC. *N*-(4-Fluorobenzoyl)-3-methyl-L-phenylalanylglycine nitrile (**56a**) was obtained as a white solid (7.6 mg, 0.02 mmol, 29%).

^{1}H NMR (400 MHz, DMSO-d_6) = 8.78 (t, ^3J = 5.6 Hz, 1H, NH), 8.71 (d, ^3J = 8.2 Hz, 1H, NH), 7.93 - 7.85 (m, 2H, H-3,5 FBz), 7.33 - 7.25 (m, 2H, H-2,6 FBz), 7.16 - 7.08 (m, 3H, H-4,5,6 aromatic Phe), 7.01 - 6.95 (m, 1H, H-2 aromatic Phe), 4.68 - 4.61 (m, 1H, C_αH), 4.16 (d, ^3J = 5.6 Hz, 2H, CH_2 Gly), 3.08 (dd, ^2J = 13.6, ^4J = 4.5 Hz, 1H, C_β*H*H), 2.95 (dd, ^2J = 13.6, ^4J = 10.4 Hz, 1H, C_βH*H*), 2.23 (s, 3H, CH_3).

^{13}C NMR (101 MHz, DMSO-d_6) δ = 171.97 (CO Gly), 165.27 (CO FBz), 163.92 (d, $^1J_{C,F}$ = 248.6 Hz, CF), 137.93, 137.05, 130.31 (d, $^4J_{C,F}$ = 3.0 Hz, C-1 FBz), 130.11 (d, $^3J_{C,F}$ = 8.9 Hz, C-2/6 FBz), 129.74, 127.97, 126.96, 126.14, 117.49 (C≡N), 115.08 (d, $^2J_{C,F}$ = 21.8 Hz, C-3/5 FBz), 54.83 (C_α Phe), 36.76 (C_β Phe), 27.16 (CH_2 Gly), 20.98 (CH_3).

^{19}F NMR (376 MHz, DMSO-d_6) δ = - 109.20 (FBz).

Diastereomeric purity > 99%

***N*-Diphenylacetyl-3-methyl-L-phenylalanylglycine nitrile (56b)**

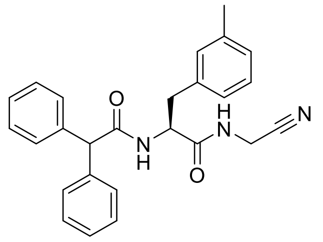

ESI (+):

m/z = 412.2017 ([M+H]$^+$)

M ($C_{26}H_{26}N_3O_2^+$, monoisotopic): 412.202

The synthesis was accomplished according to GP III. using 0.06 g **55a** (0.29 mmol, 1 eq.), 0.09 g diphenylacetic acid (0.44 mmol, 1.5 eq.), 203 µL DiPEA (1.17 mmol, 4 eq.), 0.23 g PyBOP (0.44 mmol, 1.5 eq.) and 5 mL THF. The crude product was purified *via* semipreparative HPLC. *N*-Diphenylacetyl-3-methyl-L-phenylalanylglycine nitrile (**56b**) was obtained as a white solid (0.12 g, 0.29 mmol, 100%).

^{1}H NMR (400 MHz, CD_3CN) δ = 7.32 – 7.19 (m, 8H, H aromatic, NH), 7.18 – 7.08 (m, 4H, H aromatic), 7.03 (d, ^3J = 7.6 Hz, 1H, H aromatic), 6.95 – 6.83 (m, 3H, H aromatic, NH), 4.92 (s, 1H, CH diphenylacetyl), 4.65 – 4.58 (m, 1H, C_αH Phe), 4.05 – 4.00 (m, 2H, CH_2 Gly), 3.06 (dd, ^3J = 14.0, ^2J = 5.4 Hz, 1H, C_βH*H* Phe), 2.83 (dd, ^3J = 14.0, ^2J = 8.6 Hz, 1H, C_β*H*H Phe), 2.24 (s, 3H, CH_3).

^{13}C NMR (101 MHz, CD_3CN) δ = 172.49 (CO), 172.40 (CO), 140.85, 140.81, 138.96, 137.74, 130.91, 129.76, 129.59, 129.44, 129.37, 129.28, 128.34, 127.90, 127.23, 117.62 (CN), 58.44 (CH diphenylacetyl), 55.22 (C_α Phe), 37.97 (C_β Phe), 28.13 (CH_2 Gly), 21.41(CH_3).

Due to overlapping one signal was not identified.

Diastereomeric purity > 99%

N-(4-Fluorobenzoyl)-3-methyl-L-phenylalanylglycine alkyne (56c)

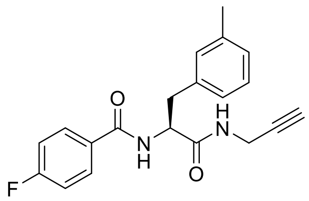

ESI (+):

m/z = 339.1504 ([M+H]$^+$)

M ($C_{20}H_{20}FN_2O_2^+$, monoisotopic): 339.1504

The synthesis was accomplished according to GP III. using 0.30 g **55b** (0.92 mmol, 1 eq.), 390 µL TEA (2.85 mmol, 3 eq.), 105 µL 4-fluorobenzoyl chloride (0.92 mmol, 1 eq.) and 20 mL CH_2Cl_2. The crude product was purified *via* semipreparative HPLC. *N*-(*4*-Fluorobenzoyl)-3-methyl-L-phenylalanylglycine alkyne (**56c**) was obtained as a white solid (0.18 g, 0.54 mmol, 59%).

^{1}H NMR (400 MHz, CD_3CN) δ = 7.80 - 7.73 (m, 2H, 2 NH), 7.20 - 6.96 (m, 8H, 4H FBz, 4H Phe aromatic), 4.73 - 4.67 (m, 1H, C_αH), 3.94 - 3.92 (m, 2H, CH_2 Gly), 3.21 (dd, ^2J = 13.9, ^3J = 5.4 Hz, 1H, C_β*H*H), 2.98 (dd, ^2J = 13.9, ^3J = 8.9 Hz, 1H, C_βH*H*), 2.43 (t, ^4J = 2.5 Hz, 1H, C≡C*H*), 2.27 (s, 3H, CH_3).

^{13}C NMR (101 MHz, CD_3CN) δ = 171.78 (CO), 166.83 (CO), 165.69 (d, $^1J_{C,F}$ = 249.3 Hz, CF), 139.01, 138.55, 131.56 (d, $^4J_{C,F}$ = 3.1 Hz, C-1 FBz), 131.07, 130.77 (d, $^3J_{C,F}$ = 9.1 Hz, C-2/6 FBz), 129.31, 128.31, 127.32, 116.30 (d, $^2J_{C,F}$ = 22.2 Hz, C-3/5 FBz), 81.25 (*C*≡CH), 71.80 (C≡*C*H), 56.03 (C_αH), 38.31 (C_βH), 29.25 (CH_2 Gly), 21.43 (CH_3).

^{19}F NMR (376 MHz, DMSO-*d$_6$*) = - 110.42 (FBz).

Diastereomeric purity > 98%

N-Diphenylacetyl-3-methyl-L-phenylalanylglycine alkyne (56d)

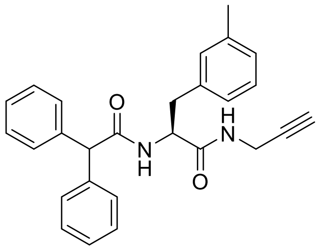

ESI (+):

m/z = 411.2067 ([M+H]$^+$)

M ($C_{27}H_{27}N_2O_2^+$, monoisotopic): 411.2067

The synthesis was accomplished according to GP III. using 0.23 g **55b** (0.72 mmol, 1 eq.), 0.23 g diphenylacetic acid (1.08 mmol, 1.5 eq.), 250 µL DiPEA (1.08 mmol, 1.5 eq.), 0.56 g PyBOP (1.08 mmol, 1.5 eq.) and 10 mL THF. The crude product was purified *via* semipreparative HPLC. *N*-Diphenylacetyl-3-methyl-L-phenylalanylglycine alkyne (**56d**) was obtained as a white solid (0.30 g, 0.72 mmol, 100%).

^{1}H NMR (400 MHz, $CDCl_3$) δ = 7.34 – 7.26 (m, 4H, H aromatic), 7.26 – 7.23 (m, 2H, H aromatic), 7.17 – 7.12 (m, 3H, H aromatic), 7.09 – 7.02 (m, 3H, H aromatic), 6.91 (s, 1H, H aromatic), 6.85 (d, ^3J = 7.5 Hz, 1H, H aromatic), 6.27 (t, ^3J = 5.0 Hz, 1H, NH

Gly), 6.13 (d, ^3J = 7.3 Hz, 1H, NH Phe), 4.89 (s, 1H, CH diphenylacetyl), 4.69 – 4.64 (m, 1H, $C_αH$ Phe), 4.01 – 3.87 (m, 2H, CH_2 Gly), 3.05 – 2.91 (m, 2H, $C_βH_2$ Phe), 2.29 (s, 3H, CH_3), 2.19 (t, ^4J = 2.6 Hz, 1H, C≡CH).

^{13}C NMR (101 MHz, $CDCl_3$) δ = 172.61 (CO diphenylacetyl), 170.53 (CO Phe), 138.89, 138.79, 138.55, 136.30, 130.12, 129.07, 128.94, 128.94, 128.92, 128.84, 128.00, 127.63, 127.50, 126.40, 79.14 (*C*≡CH), 71.74 (C≡*C*H), 59.07 (CH diphenylacetyl), 54.71($C_α$ Phe), 37.51 ($C_β$ Phe), 29.25 (CH_2 Gly), 21.53(CH_3).

Diastereomeric purity > 99%

4-Iodobenzoyl-L-leucylglycine alkyne (56e)

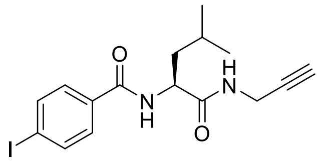

HR-MS ESI (+):

m/z = 421.0386 ([M+Na]$^+$)

M ($C_{16}H_{19}IN_2NaO_2^+$, monoisotopic): 421.0383

The synthesis was accomplished according to GP III. using 0.09 g **55c** (0.79 mmol, 1 eq.), 0.29 g 4-iodobenzoic acid (1.18 mmol, 1.5 eq.), 274 µL DiPEA (1.57 mmol, 2 eq.), 0.61 g PyBOP (1.18 mmol, 1.5 eq.) and 10 mL THF. The crude product was purified *via* semipreparative HPLC. 4-Iodobenzoyl-L-leucylglycine alkyne (**56e**) was obtained as a white solid (0.13 g, 0.13 mmol, 17%).

^{1}H NMR (400 MHz, $CDCl_3$) δ = 7.83 – 7.72 (m, 2H, H-3,5 IBz), 7.53 – 7.45 (m, 2H, H-2,6 IBz), 6.63 (d, ^3J = 8.3 Hz, 1H, NH Leu), 6.51 – 6.43 (m, 1H, NH Gly), 4.71 – 4.60 (m, 1H, $C_αH$ Leu), 4.13 – 3.94 (m, 2H, CH_2 Gly), 2.22 (t, ^4J = 2.6 Hz, 1H, C≡CH), 1.82 – 1.62 (m, 3H, $C_βH_2$, $C_γH_2$), 1.00 – 0.93 (m, 6H, CH_3).

^{13}C NMR (101 MHz, $CDCl_3$) δ = 171.63 (CO), 166.73 (CO), 137.82 (C-3/5 IBz), 132.99, 128.64 (C-2/6 IBz), 98.93, 78.96, 71.82, 51.95 ($C_α$ Leu), 41.17 ($C_β$), 29.25 (CH_2 Gly), 24.87 ($C_γ$), 22.86 (CH_3), 22.25 (CH_3).

Diastereomeric purity > 99%

4-Fluorobenzoyl-3-cyclohexyl-L-alanylglycine alkyne (56f)

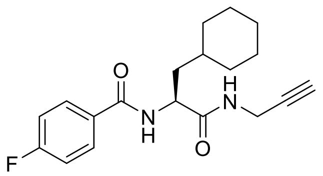

HR-MS ESI (+):

m/z = 331.1817 ([M+H]$^+$)

M ($C_{19}H_{24}FN_2O_2^+$, monoisotopic): 331.1816

The synthesis was accomplished according to GP IV. using 0.11 g **55d** (0.52 mmol, 1 eq.), 231 µL TEA (1.65 mmol, 3.2 eq.), 56 µL 4-fluorobenzoyl chloride (0.52 mmol, 1 eq.) and 20 mL CH_2Cl_2. The crude product was purified *via* semipreparative HPLC.

4-Fluorobenzoyl-3-cyclohexyl-L-alanylglycine alkyne (**56f**) was obtained as a white solid (94.8 mg, 0.29 mmol, 56%).

^{1}H NMR (400 MHz, $CDCl_3$) δ = 7.84 – 7.74 (m, 2H, H-3,5 aromatic FBz), 7.18 – 7.03 (m, 2H, H-2,6 aromatic FBz), 6.69 – 6.51 (m, 2H, NH Cha, NH Gly), 4.75 – 4.62 (m, 1H, C_{α}H), 4.15 – 4.04 (m, 1H, C*H*H Gly), 4.03 – 3.92 (m, 1H, CH*H* Gly), 2.22 (t, ^{4}J = 2.6 Hz, 1H, C≡CH), 1.89 – 1.59 (m, 8H, $C_{\beta}H_2$, CH_2 Cha), 1.45 – 1.33 (m, 1H, CH Cha), 1.31 – 1.08 (m, 2H, CH_2 Cha), 1.07 – 0.85 (m, 2H, CH_2 Cha).

^{13}C NMR (101 MHz, $CDCl_3$) δ = 171.98 (CO), 166.61 (CO), 165.14 (d, $^{1}J_{C,F}$ = 252.7 Hz, CF), 129.95 (d, $^{4}J_{C,F}$ = 3.1 Hz, C-1 FBz), 129.65 (d, $^{3}J_{C,F}$ = 9.0 Hz, C-2/6 FBz), 115.87 (d, $^{2}J_{C,F}$ = 21.9 Hz, C-3/5 FBz), 79.17 (*C*≡CH), 71.96 (C≡*C*H), 51.50 (C_{α}), 39.87 (C_{β}), 34.37, 33.73, 33.06, 29.42, 26.48, 26.29, 26.18.

^{19}F NMR (376 MHz, $CDCl_3$) = - 107.41 (FBz).

Diastereomeric purity > 96%

8 Synthesis of Dipeptide Nitrile 62 with Isobutylsulfonylalanine in P2 (Scheme 7, bottom)

(S)-Isobutyl-L-cysteine (57)

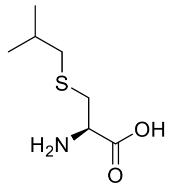

L-Cysteine (0.20 g, 1.14 mmol, 1 eq.), isobutyl bromide (136 µL, 1.25 mmol, 1.1 eq.) and tetrabutylammonium iodide (0.01 g, 0.03 mmol, 0.03 eq.) were dissolved in 3 M NaOH/EtOH (1:1, 5 mL). The solution was stirred 3 days at room temperature. The reaction progress was monitored *via* thin layer chromatography. *(S)*-Isobutyl-L-cysteine (**57**) was used without further purification.

N-(*tert*-Butyloxycarbonyl)-isobutyl-L-cysteine (58)

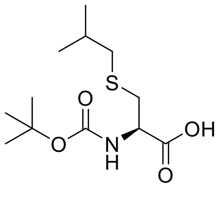

HR-MS ESI (+):

m/z = 300.1240 ($[M+Na]^+$)

M ($C_{12}H_{23}NNaO_4S^+$, monoisotopic): 300.1240

To crude **57** in 3 M NaOH/EtOH (1:1), Boc_2O (0.27 g, 1.25 mmol, 1.1 eq.) was added and the solution stirred 24 h. The prganic solvent was evaporated and the aqueous phase acidified to pH = 1 using HCl. The product was extracted with ethyl acetate (2 × 5 mL) and the combined organic layers washed with aqueous $KHSO_4$ (10%, 1 mL)

and brine (1 mL) and dried over Na_2SO_4. The solvent was evaporated. *N*-(*tert*-Butyloxycarbonyl)isobutyl-L-cysteine (**58**) was obtained as an orange oil (0.13 g, 0.51 mmol, 41% over two steps).

^{1}H NMR (400 MHz, $CDCl_3$) δ = 5.37 (s, 1H, NH), 4.51 (s, 1H, $C_\alpha H$), 2.99 (d, ^3J = 5.2 Hz, 2H, $C_\beta H_2$), 2.45 (d, ^3J = 6.8 Hz, 2H, CH_2S), 1.79 (hept, ^3J = 13.4, 1H, C*H*$(CH_3)_2$), 1.46 (s, 9H, $(CH_3)_3$), 0.98 (d, ^3J = 6.6, 6H, $(CH_3)_2$).

The carboxyl proton was not detectable in the chosen solvent.

^{13}C NMR (101 MHz, $CDCl_3$) δ = 174.63 (COO), 155.77(CO), 77.36 (*C*$(CH_3)_3$), 53.29 (C_α), 42.15 (CH_2S), 34.68 (C_β), 28.76 (*C*H$(CH_3)(CH_3)$), 28.44 ($(CH_3)_3$), 22.04 (CH(*C*H_3)(CH_3)), 22.00 (CH(CH_3)(*C*H_3)).

***N*-(*tert*-Butyloxycarbonyl)-3-(isobutylsulfonyl)-L-alanine (59)**

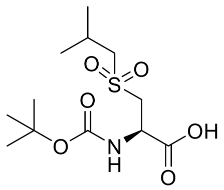

HR-MS ESI (+):

m/z = 332.1141 ($[M+Na]^+$)

M ($C_{12}H_{23}NNaO_6S^+$, monoisotopic): 332.1138

N-(*tert*-Butyloxycarbonyl)isobutyl-L-cysteine (**58**, 0.10 g, 0.36 mmol, 1 q.) was dissolved in acetic acid (710 µL). Potassium permanganate (0.11 g, 0.71 mmol, 2 eq.) in acetic acid (533 µL) was added dropwise and the solution stirred 2,5 h. Then aqueous $KHSO_4$ (sat.) was added until the solution turned colorless. The product was extracted with ethyl acetate (3 × 1 mL). The organic layers were washed with H_2O (10 mL) and brine (10 mL) and dried over Na_2SO_4. The solvent was evaporated. *N*-(*tert*-Butyloxycarbonyl)-3-(isobutylsulfonyl)-L-alanine (**59**) was obtained as a clear oil (0.15 g, quantitative yield).

^{1}H NMR (400 MHz, $CDCl_3$) δ = 5.70 (d, ^3J = 7.2 Hz, 1H, NH), 4.72 – 4.62 (m, 1H, $C_\alpha H$), 3.67 (d, ^3J = 5.0 Hz, 2H, $C_\beta H_2$), 2.95 (d, ^3J = 6.6 Hz, 2H, CH_2SO_2), 2.44 – 2.31 (m, 1H, C*H*$(CH_3)_2$), 1.46 (s, 9H, $(CH_3)_3$), 1.12 (d, ^3J = 6.8 Hz, 6H, $(CH_3)_2$).

The carboxyl proton was not detectable in the chosen solvent.

***N*-(*tert*-Butyloxycarbonyl)-3-(isobutylsulfonyl)-L-alanylglycine alkyne (60)**

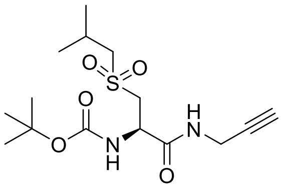

ESI (+):

m/z = 368.86 ($[M+Na]^+$)

M ($C_{15}H_{26}N_2NaO_5S^+$, monoisotopic): 369.15

The synthesis was accomplished according to GP VIII. using 0.13 g **59** (0.43 mmol, 1 eq.), 55 µL propargylamine (0.87 mmol, 2 eq.), 48 µL NMM (0.43 mmol, 1 eq.), 56 µL iBCF (0.43 mmol, 1 eq.) and 10 mL THF. *N*-(*tert*-Butyloxycarbonyl)-3-(isobutylsulfonyl)-L-alanylglycine alkyne (**60**) was obtained as a white solid (0.06 g, 0.16 mmol, 37%).

^{1}H NMR (400 MHz, $CDCl_3$) δ = 7.02 – 6.88 (m, 1H, NH Gly), 5.82 (d, ^3J = 7.8 Hz, 1H, NH Cys), 4.71 – 4.58 (m, 1H, $C_{\alpha}H$), 4.18 – 3.95 (m, 2H, CH_2 Gly), 3.69 (d, ^3J = 15.0 Hz, 1H, C_{β}*H*H), 3.37 (dd, ^3J = 14.9, ^2J = 5.5 Hz, 1H, C_{β}H*H*), 3.20 – 2.94 (m, 2H, CH_2SO_2), 2.50 – 2.29 (m, 1H, C*H*$(CH_3)_2$), 2.25 (td, ^4J = 2.5, ^5J = 0.8 Hz, 1H, C≡CH), 1.46 (s, 9H, $(CH_3)_3$), 1.14 (d, ^3J = 2.5 Hz, 3H, CH(C*H*$_3$)(CH_3)), 1.12 (d, ^3J = 2.5 Hz, 3H, CH(CH_3)(C*H*$_3$)).

^{13}C NMR (101 MHz, $CDCl_3$) δ = 168.93 (CO), 155.46 (CO), 78.80 (*C*$(CH_3)_3$), 77.36 (*C*≡CH), 72.16 (C≡*C*H), 62.15 (CH_2SO_2), 55.65 (C_{β}), 50.36 (C_{α}), 29.73 (CH_2 Gly), 28.38 ($(CH_3)_3$), 23.92 (*C*H(CH_3)(CH_3)), 22.89 (CH(*C*H$_3$)(CH_3)), 22.79 (CH(CH_3)(*C*H$_3$)).

3-(Isobutylsulfonyl)-L-alanylglycine alkyne (61)

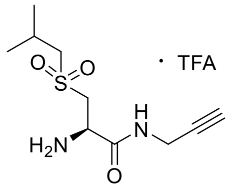

ESI (+):

m/z = 247.19 ($[M+H]^+$)

M ($C_{10}H_{19}N_2O_3S^+$, monoisotopic): 247.11

The synthesis was accomplished according to GP II. using 0.01 g *N*-(*tert*-Butyloxycarbonyl)-3-(isobutylsulfonyl)-L-alanylglycine alkyne (**60**, 0.03 mmol) and 10 mL TFA/CH_2Cl_2 (1:1). 3-(Isobutylsulfonyl)-L-alanylglycine alkyne (**61**) was obtained as a white solid (0.01 g, quantitative yield; contained TFA).

^{1}H NMR (400 MHz, DMSO-*d$_6$*) δ = 9.11 (t, ^3J = 5.3 Hz, 1H, NH),8.44 (broad s, 3H, NH_3^+) 4.31 – 4.25 (m, 1H, $C_{\alpha}H$), 4.02 – 3.88 (m, 2H, CH_2 Gly), 3.75 – 3.39 (m, 2H, $C_{\beta}H_2$), 3.26 (t, ^4J = 2.5 Hz, 1H, C≡CH), 3.23 – 3.11 (m, 2H, CH_2SO_2), 2.34 – 2.16 (m, 1H, C*H*$(CH_3)_2$), 1.05 (d, ^3J = 6.7 Hz, 6H, $(CH_3)_2$).

^{13}C NMR (101 MHz, DMSO-*d$_6$*) δ = 165.67 (CO), 79.73 (*C*≡CH), 74.21 (C≡*C*H), 59.73 (CH_2SO_2), 53.24 (C_{β}), 47.16 (C_{α}), 28.66 (CH_2 Gly), 22.89 (*C*H$(CH_3)_2$), 22.33 (C(*C*H$_3$)(CH_3)), 22.31 (C(CH_3)(*C*H$_3$)).

4-Fluorobenzoyl-3-(isobutylsulfonyl)-L-alanylglycine alkyne (62)

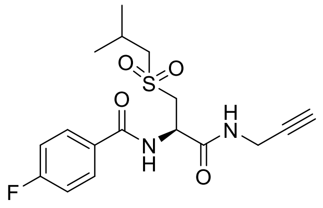

HR-MS ESI (+):

m/z = 391.1099 ([M+Na]$^+$)

M ($C_{17}H_{21}FN_2NaO_4S^+$, monoisotopic): 391.1098

The synthesis was accomplished according to GP IV. using 0.01 g **61** (0.03 mmol, 1 eq.), 4 µL 4-fluorobenzoyl chloride (0.03 mmol, 1 eq.), 15 µL TEA (0.10 mmol, 3 eq.) and 5 mL CH_2Cl_2. The crude product was purified *via* semipreparative HPLC. 4-Fluorobenzoyl-3-(isobutylsulfonyl)-L-alanylglycine alkyne (**62**) was obtained as a white solid (3.8 mg, 0.01 mmol, 30%).

^{1}H NMR (400 MHz, CD_3CN) δ = 7.94 – 7.86 (m, 2H, H-3,5 H aromatic), 7.65 (d, ^3J = 8.0 Hz, 1H, NH), 7.30 (broad s, 1H, NH), 7.27 – 7.19 (m, 2H, H-2,6 aromatic), 5.06 – 4.98 (m, 1H, $C_αH$), 4.01 – 3.88 (m, 2H, CH_2 Gly), 3.70 (dd, ^3J = 14.7, ^2J = 4.0 Hz, 1H, $C_βH\mathit{H}$), 3.54 (dd, ^3J = 14.7, ^2J = 8.6 Hz, 1H, $C_β\mathit{H}H$), 3.01 (d, ^3J = 6.6 Hz, 2H, CH_2SO_2), 2.43 (t, ^4J = 2.6, 1H, C≡CH), 2.28 (p, ^3J = 13.4 Hz, 1H, C*H*(CH_3)$_2$), 1.08 – 1.03 (m, 6H, $(CH_3)_2$).

^{13}C NMR (101 MHz, CD_3CN) δ = 169.59 (CO), 166.97 (CO), 164.83 (d, $^1J_{C,F}$ = 249.9 Hz, CF), 131.05, 131.05 (d, $^4J_{C,F}$ = 3.0 Hz, C-1 FBz), 130.99 (d, $^3J_{C,F}$ = 9.1 Hz, C-2/6 FBz), 116.52 (d, $^2J_{C,F}$ = 22.1 Hz, C-3/5 FBz), 80.93 (*C*≡CH), 71.83 (C≡*C*H), 61.73 (CH_2SO_2), 54.60 ($C_β$), 49.78 ($C_α$), 29.47 (CH_2 Gly), 24.31 (*C*H(CH_3)$_2$), 22.79 (CH_3).

Diastereomeric purity > 99%

9 Methylation of Dipeptide Alkynes 2b and 2f

Synthesis of *N*-nitroso-*N*-methylurea

Half-concentrated sulfuric acid (15 mL, 135 mmol, 1 eq.) was added slowly to an ice-cooled solution of *N*-methylurea (10 g, 135 mmol, 1 eq.) and sodium nitrite (10.25 g, 149 mmol, 1.1 eq.) in H_2O (50 mL) (caution: nitrous gases evolve!). Then the reaction mixture was stored 2 h in the fridge before the precipitate was aspirated, washed with H_2O and dried in the desiccator over P_4O_{10}. *N*-Nitroso-*N*-methylurea was obtained as a white solid (8.72 g, 85 mmol, 63%).

Melting point: 118 °C (decompos.) (lit.: 112-113 °C[21])

^{1}H NMR (400 MHz, $CDCl_3$): δ = 3.19 (s, 3H, CH_3N); 5.66 (broad s, 1H, CON*H*H); 6.91 (broad s, 1H, CONH*H*)

^{13}C NMR (101 MHz, $CDCl_3$): δ = 26.43 (CH_3N); 154.53 ($NCONH_2$)

N-(4-Fluorobenzoyl)-3-methyl-L-phenylalanyl-O-(3-(methoxycarbonyl)benzyl)-L-serine alkyne (63)

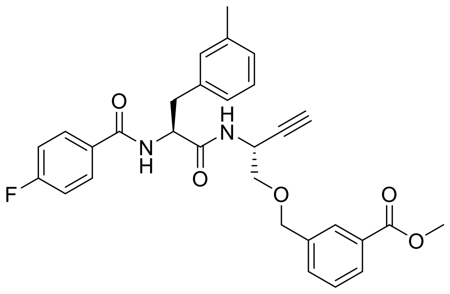

HR-MS ESI (+):

m/z = 517.2132 ([M+H]$^+$)

M ($C_{30}H_{30}FN_2O_5^+$, monoisotopic): 517.2134

N-Nitroso-*N*-methylurea was synthesized as described above. 40% KOH (1 mL) and ether (1 mL) were mixed in a scintillation container and ice-cooled before adding 4 mg *N*-nitroso-*N*-methylure (0,04 mmol, 4 Eq.) as a solid while carefully shaking the container. 10 min after the addition (organic layer turned yellow), the ether phase was separated and added to solid *N*-(4-fluorobenzoyl)-3-methyl-L-phenylalanyl-*O*-(3-carboxybenzyl)serine alkyne (**2b**, 7 mg, 0.01 mmol, 1 eq.). The reaction progress was monitored *via* thin layer chromatography. If the reaction was not completed, the same amount of freshly prepared diazomethane solution was added. *N-(*4-Fluorobenzoyl)-3-methyl-L-phenylalanyl-*O*-(3-(methoxycarbonyl)benzyl)-L-serine alkyne (**63**) was obtained as a white solid (1.8 mg, 0.003 mmol, 35%).

^{1}H NMR (400 MHz, CD_3CN) δ = 7.96 – 7.93 (m, 1H, H-2 BnCOO), 7.91 – 7.87 (m, 1H, H-4 BnCOO), 7.76 – 7.69 (m, 2H, H-2,6 FBz), 7.57 – 7.52 (m, 1H, H-6 BnCOO), 7.41 (t, ^3J = 7.7 Hz, 1H, H-5 BnCOO), 7.22 – 7.00 (m, 8H, H-3,5 FBz, 2 NH, aromatic Phe), 4.95 – 4.86 (m, 1H, C_αH Phe), 4.76 – 4.68 (m, 1H, C_αH Ser), 4.64 – 4.53 (m, 2H, CH_2O), 3.86 (s, 3H, OCH_3), 3.61 (d, ^3J = 5.6 Hz, 2H, $C_\beta H_2$ Ser), 3.20 (dd, ^3J = 13.9, ^2J = 5.5 Hz, 1H, C_βH*H* Phe), 2.98 (dd, ^3J = 13.9, ^2J = 8.9 Hz, 1H, C_β*H*H Phe), 2.55 (d, ^4J = 2.4 Hz, 1H, C≡CH), 2.26 (s, 3H, CH_3).

^{13}C NMR (101 MHz, CD_3CN) δ = 171.41 (CO), 167.64 (CO), 166.84 (CO), 165.60 (d, $^1J_{C,F}$ = 250.5 Hz, CF), 139.90, 138.91, 138.43, 133.07, 131.39 (d, $^4J_{C,F}$ = 3.0 Hz, C-1 FBz), 131.29, 131.05, 130.68 (d, $^3J_{C,F}$ = 9.1 Hz, C-2/6 FBz), 129.59, 129.40, 129.23, 129.20, 128.24, 127.29, 116.21 (d, $^2J_{C,F}$ = 22.2 Hz, C-3/5 FBz), 82.00 (*C*≡CH), 72.98, 72.73, 72.50, 55.93 (C_α Ser), 52.73 (OCH_3), 41.84 (C_α Phe), 38.06 (C_β Phe), 21.36 (CH_3).

Diastereomeric purity > 96%

_N-(_3-Iodobenzoyl)-3-methyl-L-phenylalanyl-_O_-(3-(methoxycarbonyl)benzyl)-L-serine alkyne (64)

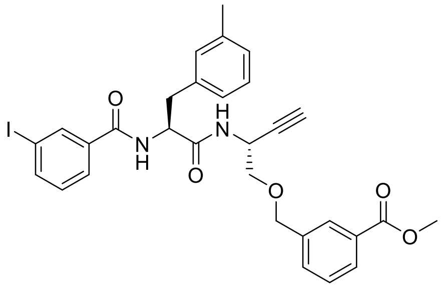

HR-MS ESI (+):

m/z = 625.1192 ($[M+H]^+$)

M ($C_{30}H_{30}IN_2O_5^+$, monoisotopic): 625.1194

N-Nitroso-*N*-methylurea was synthesized as described above. 40% KOH (1 mL) and ether (1 mL) were mixed in a scintillation container and ice-cooled before adding 3 mg *N*-nitroso-*N*-methylure (0,032 mmol, 4 Eq.) as a solid while carefully shaking the container. 10 min after the addition (organic layer turned yellow), the ether phase was separated and added to solid *N-(*3-iodobenzoyl)-3-methyl-L-phenylalanyl-*O*-(3-carboxybenzyl)-L-serine alkyne (**2f**, 5 mg, 0.008 mmol, 1 eq.). The reaction progress was monitored *via* thin layer chromatography. If the reaction was not completed, the same amount of freshly prepared diazomethane solution was added. *N-(*3-Iodobenzoyl)-3-methyl-L-phenylalanyl-*O*-(3-(methoxycarbonyl)benzyl)-L-serine alkyne (**64**) was obtained as a white solid (0.7 mg, 0.001 mmol, 14%).

^{1}H NMR (400 MHz, CD_3CN) δ = 8.00 (t, ^4J = 1.7 Hz, 1H, H-2 IBz), 7.96 – 7.92 (m, 1H, H-2 BnCOO), 7.91 – 7.82 (m, 2H, H-4 BnCOO, H-4 IBz), 7.68 – 7.64 (m, 1H, H-6 IBz), 7.54 (d, ^3J = 7.8 Hz, 1H, H-6 BnCOO), 7.41 (t, ^3J = 7.7 Hz, 1H, H-5 BnCOO), 7.25 (d, ^3J = 8.0 Hz, 1H, NH), 7.21 – 7.00 (m, 6H, NH, H-5 IBz, aromatic Phe), 4.95 – 4.86 (m, 1H, $C_\alpha H$ Phe), 4.75 – 4.67 (m, 1H, $C_\alpha H$ Ser), 4.63 – 4.54 (m, 2H, CH_2O), 3.86 (s, 3H, OCH_3), 3.62 (d, ^3J = 5.7 Hz, 2H, $C_\beta H_2$ Ser), 3.24 – 3.17 (m, 1H, $C_\beta H\mathit{H}$ Phe), 3.00 – 2.93 (m, 1H, $C_\beta \mathit{H}H$ Phe), 2.55 (d, ^4J = 2.4 Hz, 1H, C≡CH), 2.28 (s, 3H, CH_3).

^{13}C NMR (101 MHz, CD_3CN) δ = 171.27 (CO), 168.51 (COO), 167.81 (CO), 141.32, 139.90, 138.91, 138.42, 137.09, 137.02, 133.03, 131.34, 131.08, 129.59, 129.40, 129.25, 129.17, 128.26, 127.56, 127.26, 127.10, 105.87 (CI), 81.97 (*C*≡CH), 72.95 (C≡*C*H), 72.74 (CH_2O), 72.51 (C_β Ser), 56.00 (C_α Phe), 52.77 (OCH_3), 41.82 (C_α Ser), 37.96 (C_β Phe), 21.42 (CH_3).

Diastereomeric purity > 84%

NMR spectra of inhibitor compounds

1 Dipeptide nitriles 1a – e

*N-(*4-Fluorobenzoyl)-3-methyl-L-phenylalanyl-*O*-(3-carboxybenzyl)serine nitrile (1a)

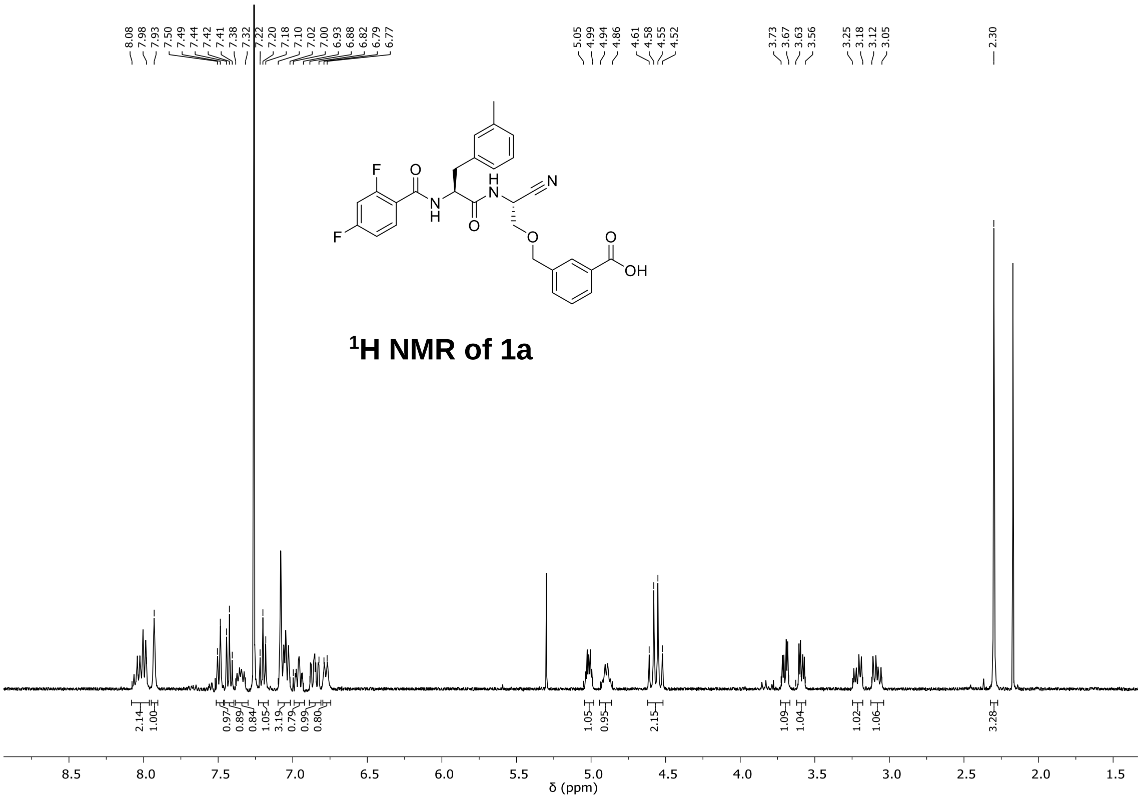

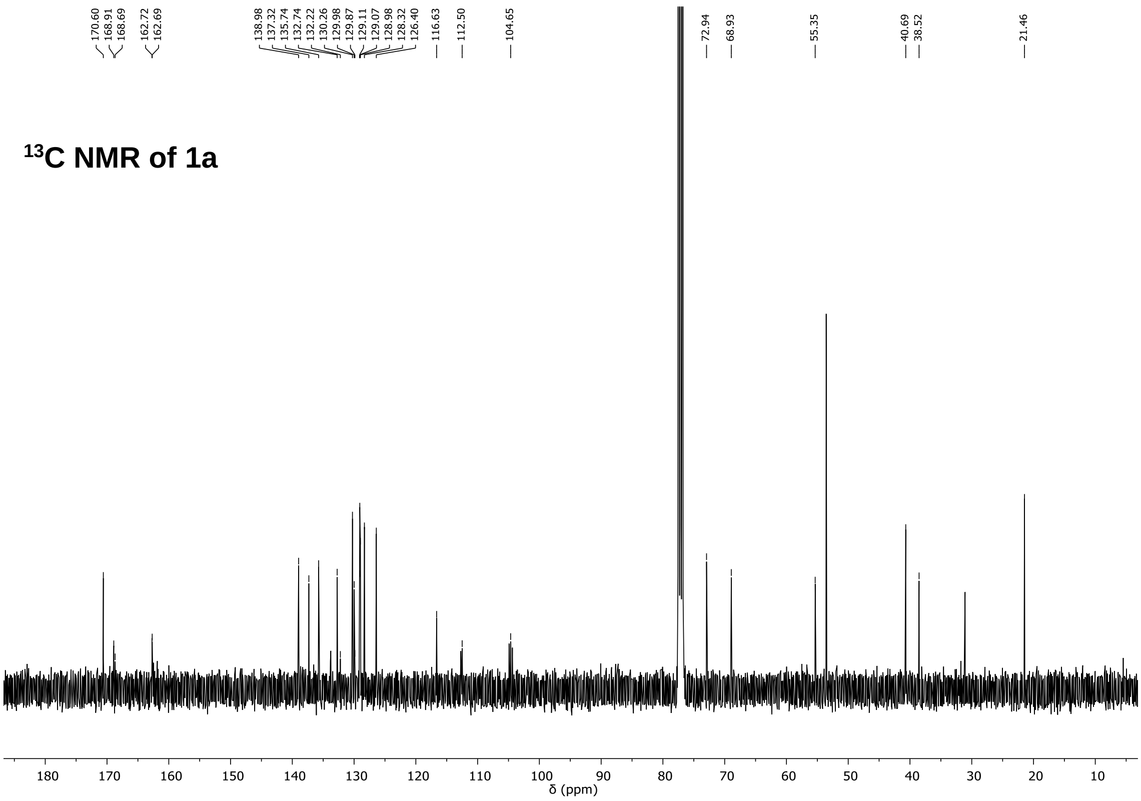

***N*-(4-Fluorobenzoyl)-3-methyl-L-phenylalanyl-*O*-(3-carboxybenzyl)serine nitrile (1b)**

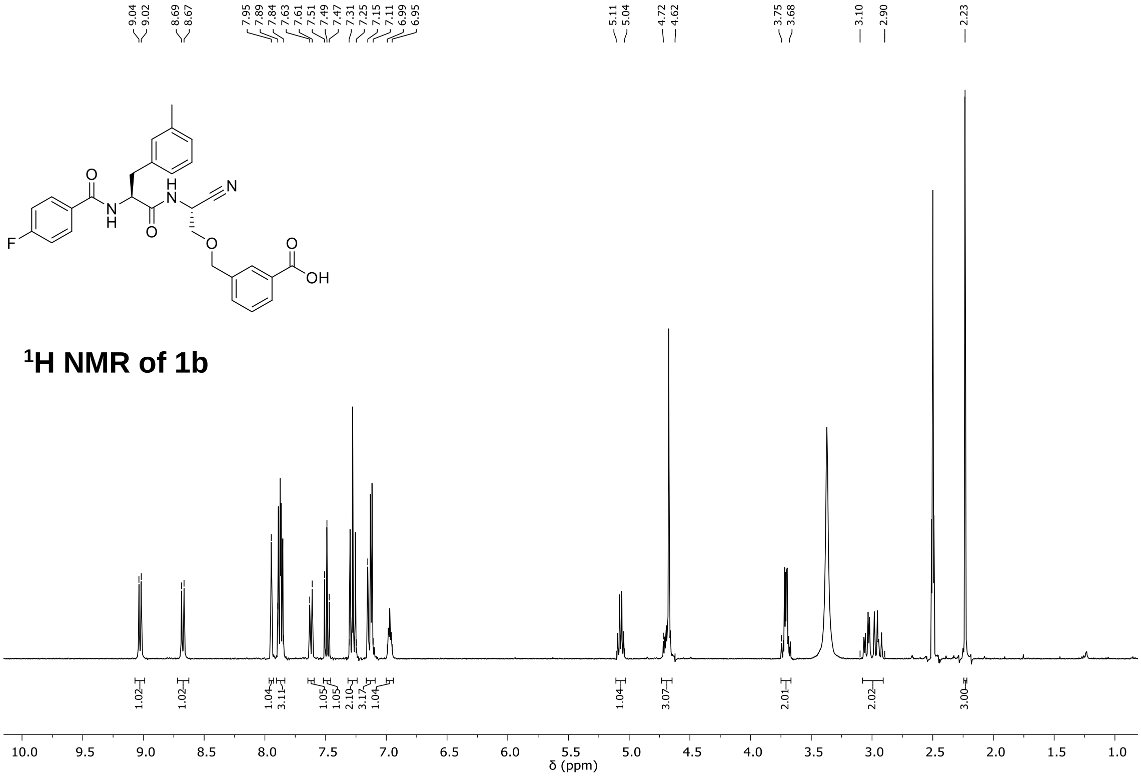

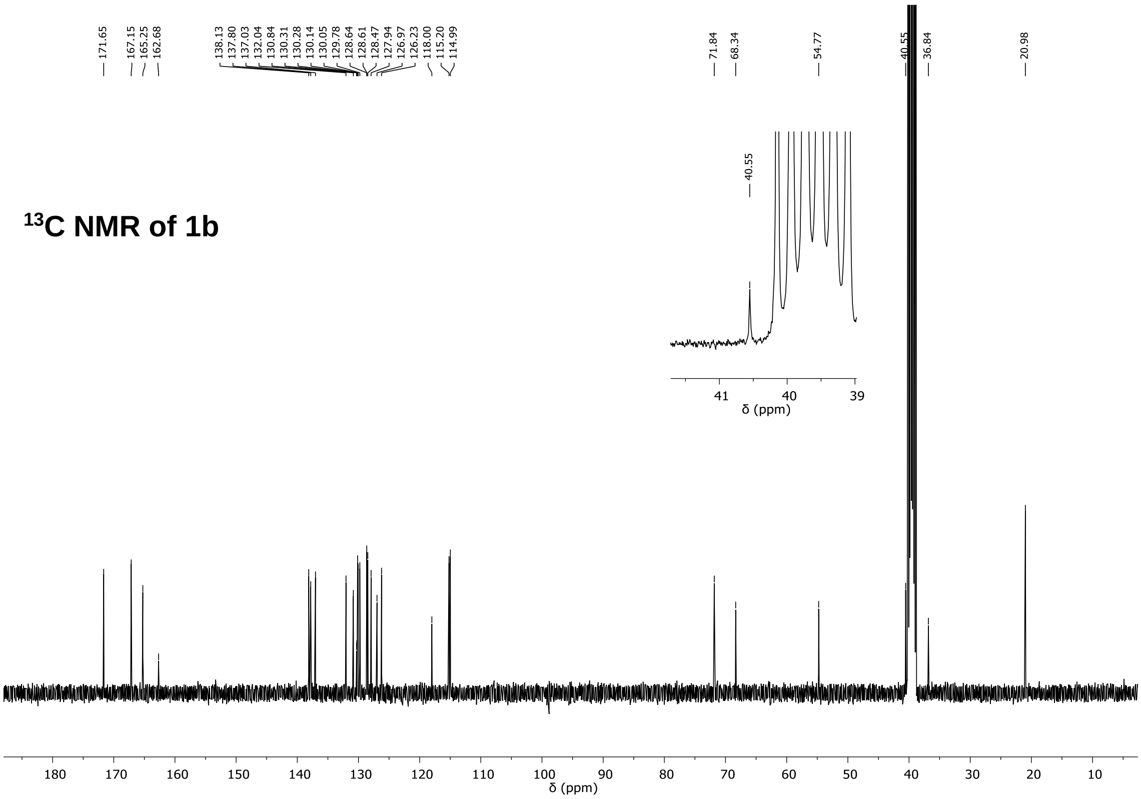

N-Diphenylacetyl-3-methyl-L-phenylalanyl-*O*-(3-carboxybenzyl)-L-serine nitrile (1c)

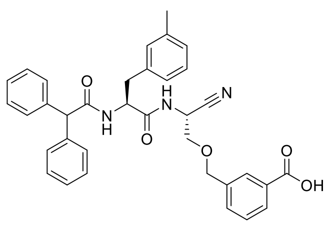

^{1}H NMR of 1c

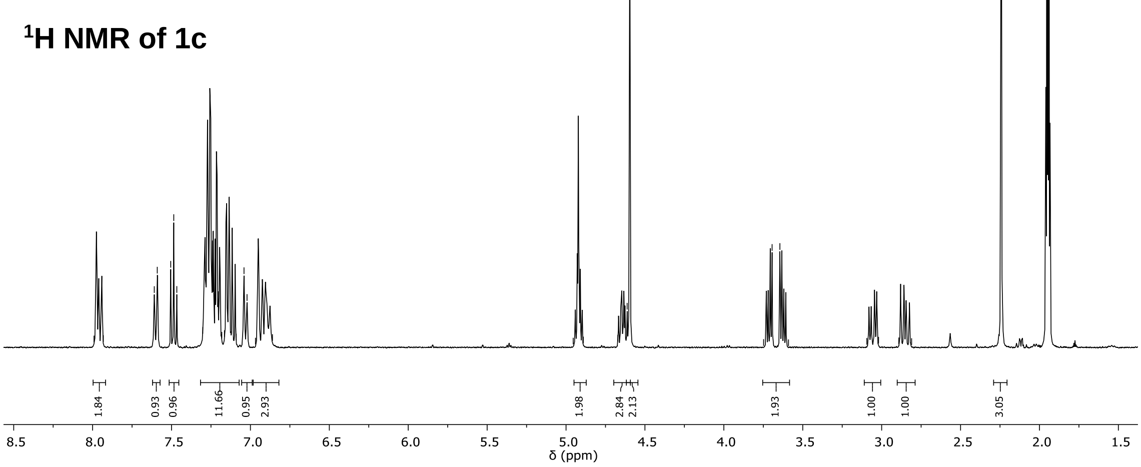

^{13}C NMR of 1c

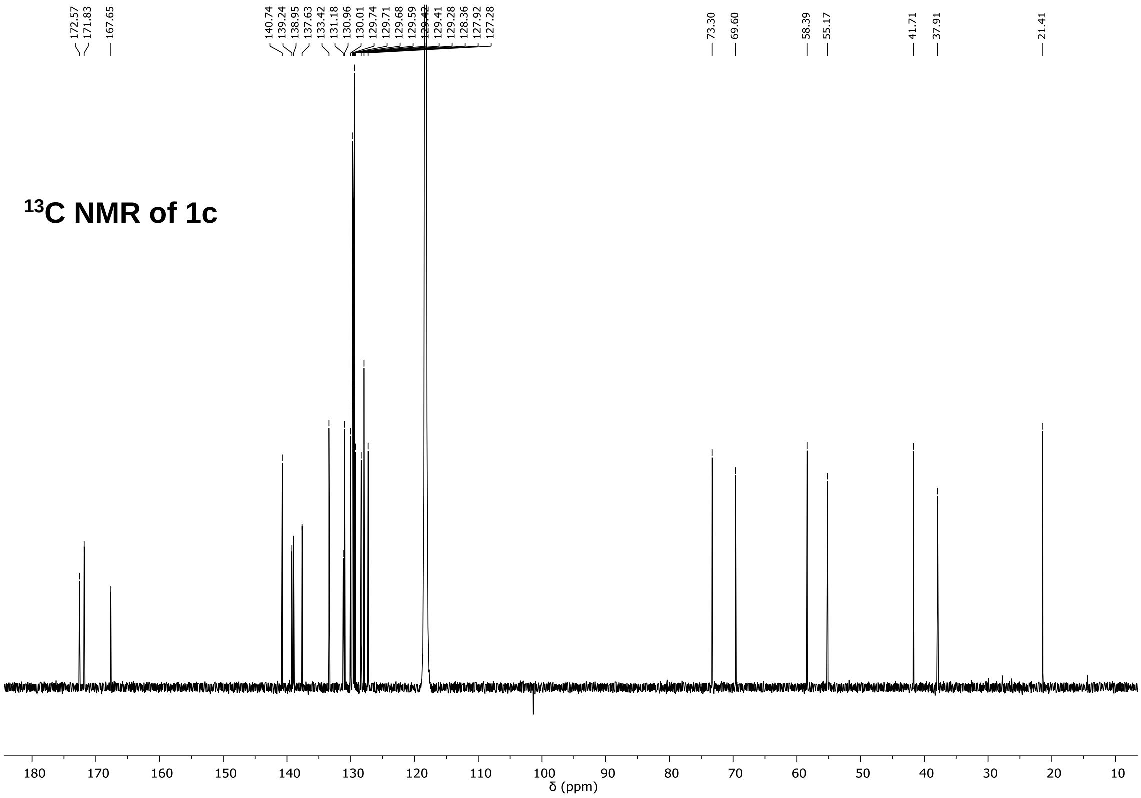

N-(4-Fluorobenzoyl)-3-iodo-L-phenylalanyl-*O*-(3-carboxybenzyl)-L-serine nitrile (1d)

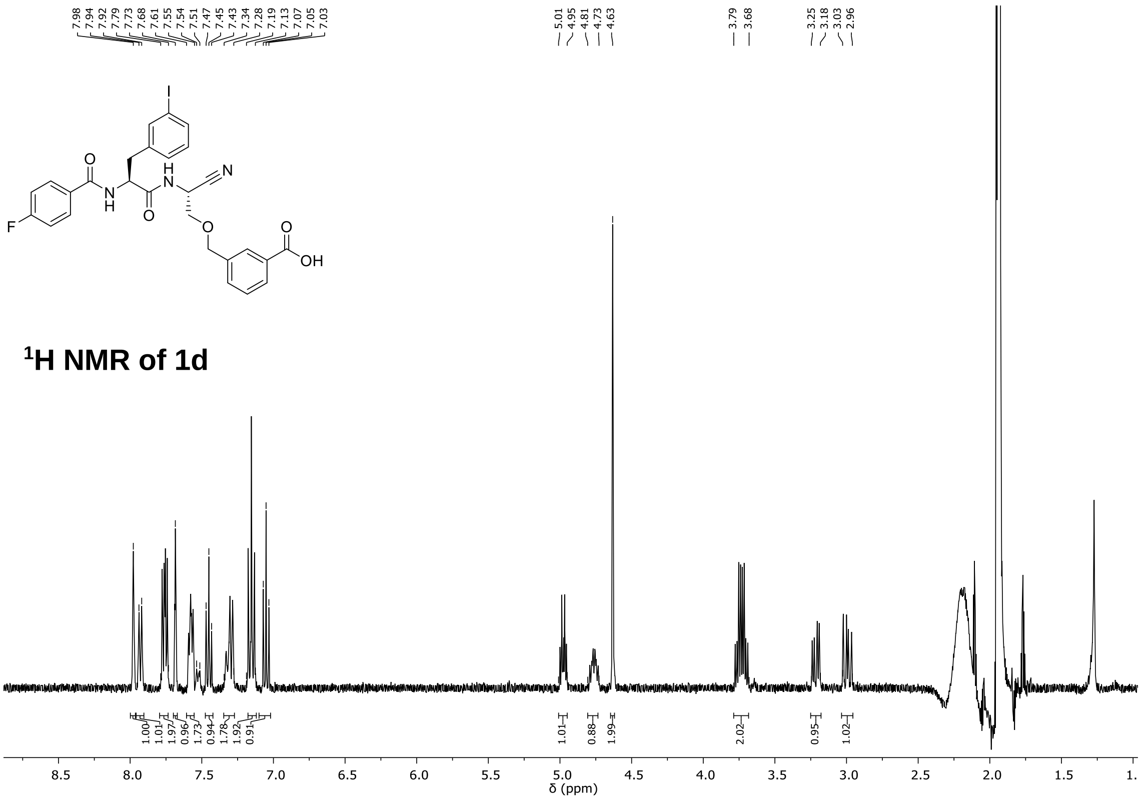

^{1}H NMR of 1d

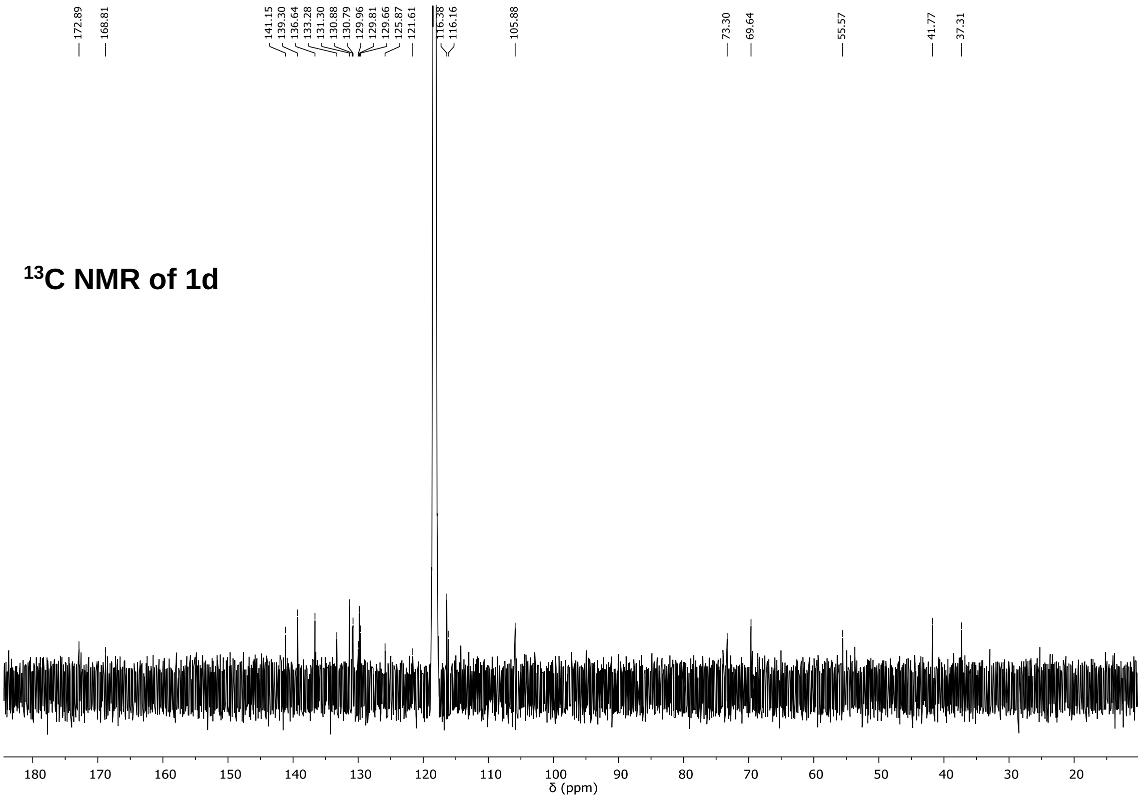

^{13}C NMR of 1d

_N_-Diphenylacetyl-3-iodo-L-phenylalanyl-_O_-(3-carboxybenzyl)-L-serine nitrile (1e)

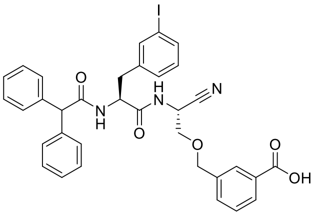

^{1}H NMR of 1e

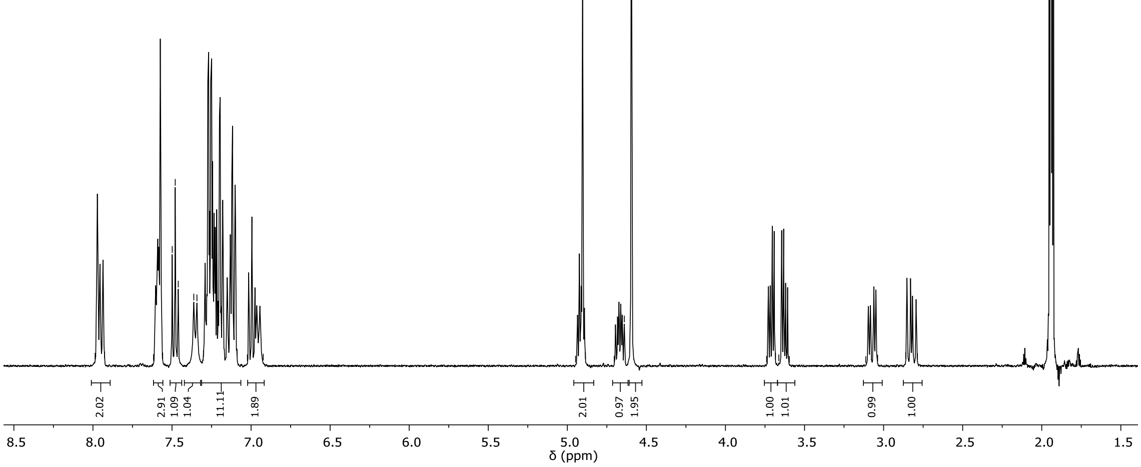

^{13}C NMR of 1e

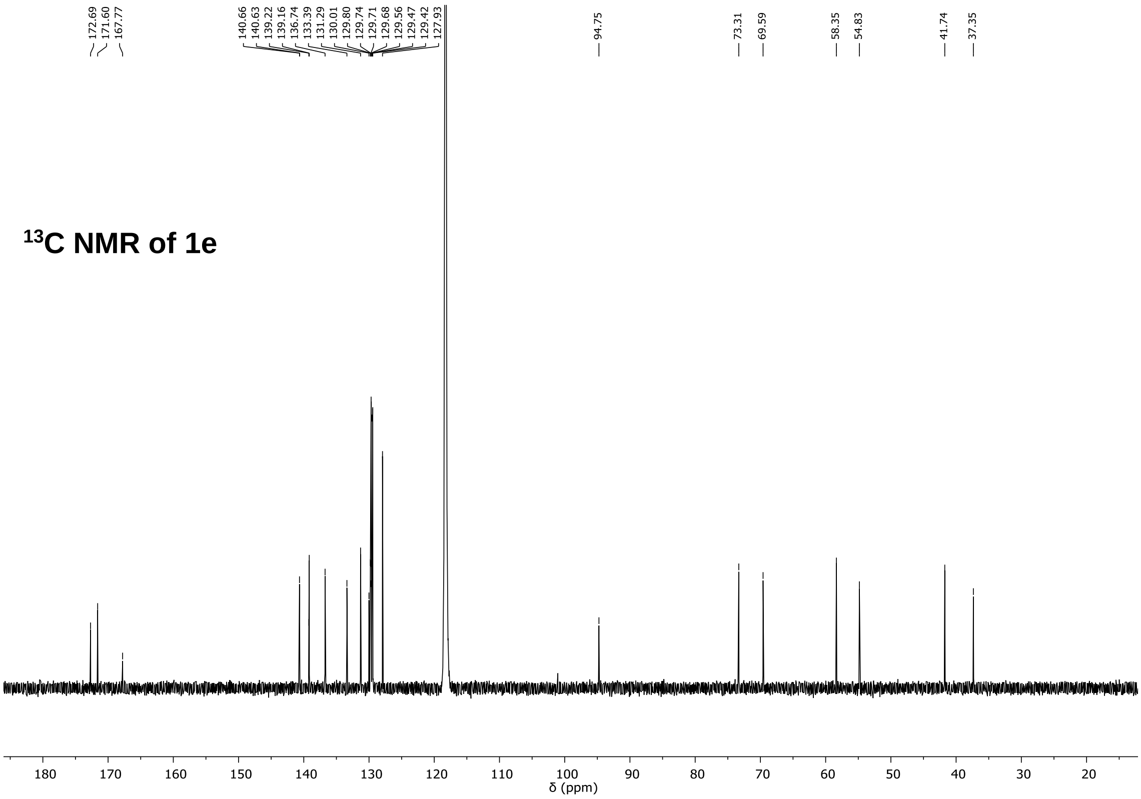

2 Dipeptide alkyne 18 (mixture of 2a and epi-2a)

N-(2,4-Difluorobenzoyl)-3-methyl-L-phenylalanyl-*O*-(3-carboxybenzyl)serine alkyne (18)

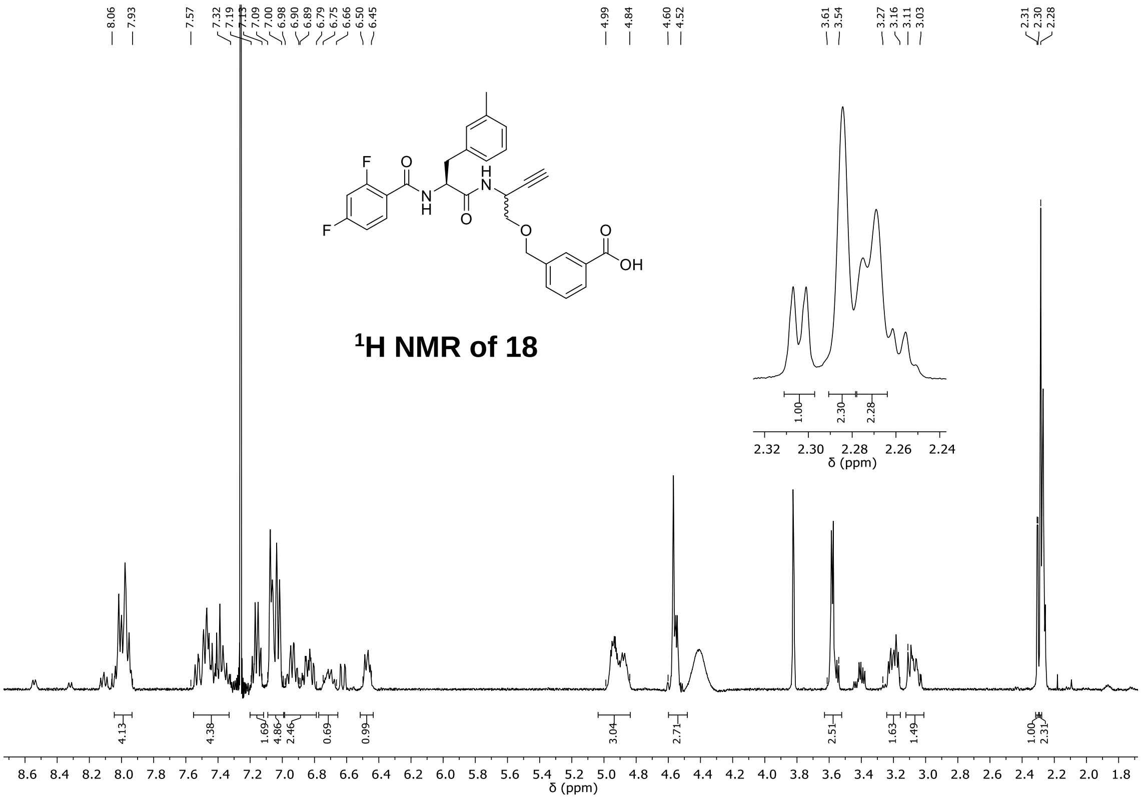

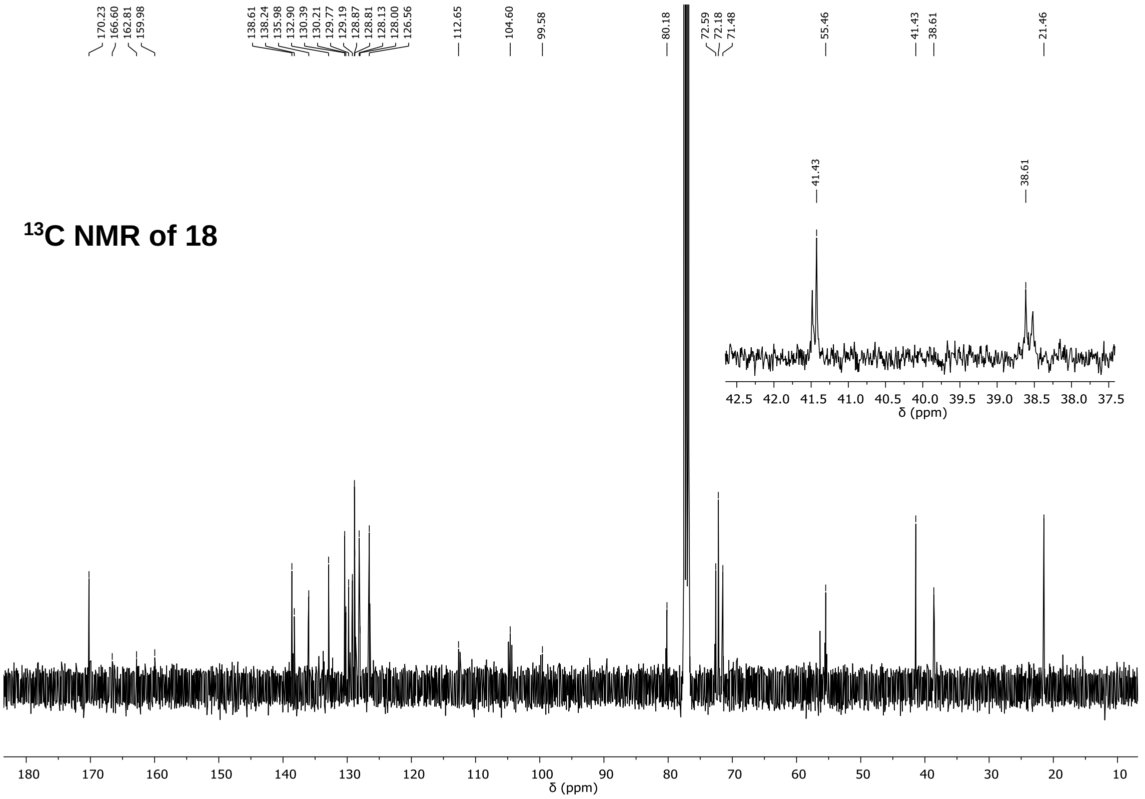

3 Dipeptide alkyne 28 with Carboxy-functionalised 1,2,3-triazole ring in P2

N-(4-Fluorobenzoyl)-3-methyl-L-phenylalanyl-(4-carboxy-1*H*-1,2,3-triazole-1-yl)-L-norvaline alkyne (28)

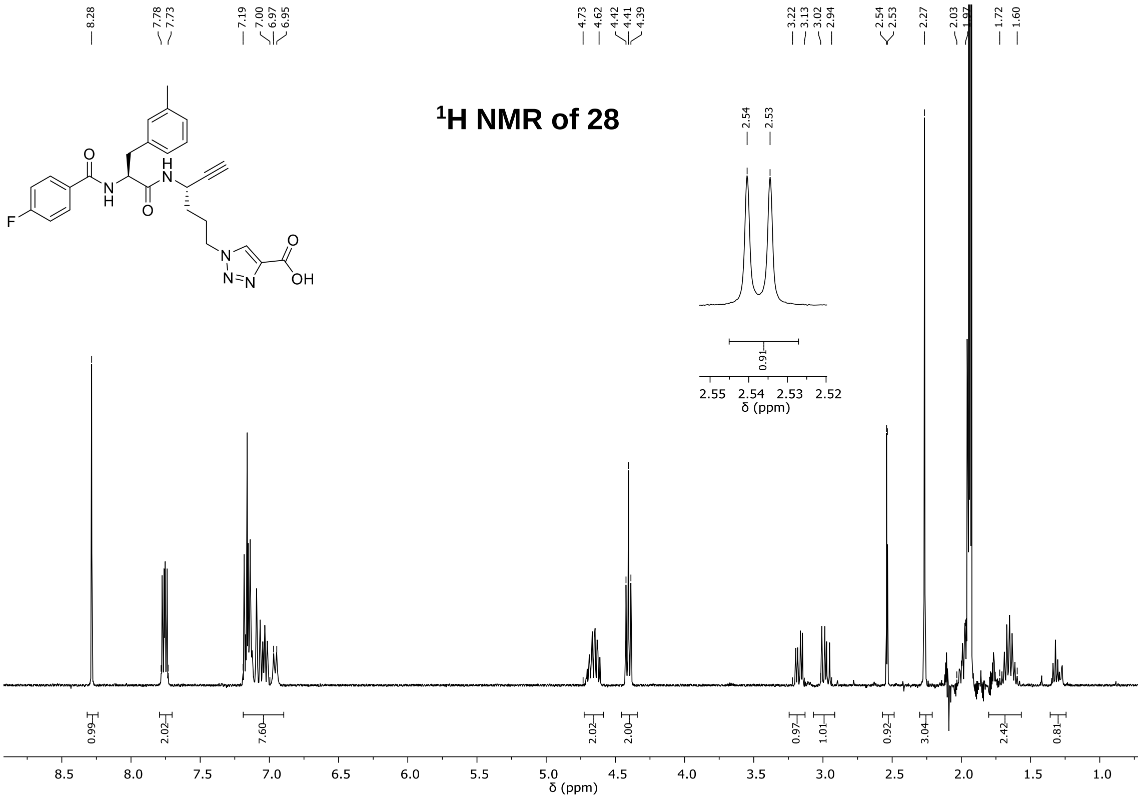

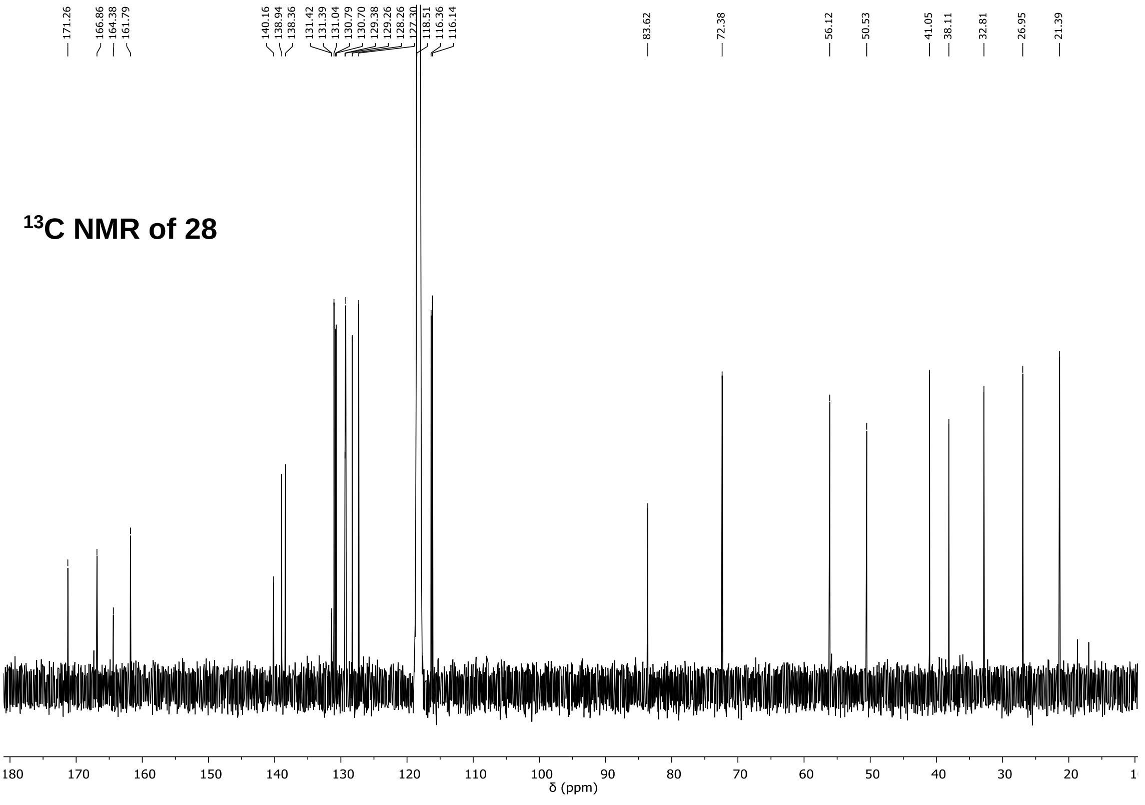

4 Dipeptide nitriles 35a – c mit carboxy-functionalized 1,2,3-triazole ring in P2

N-(4-Fluorobenzoyl)-3-methyl-L-phenylalanyl-(4-carboxy)-1*H*-1,2,3-triazole-1-yl)-L-norvaline nitrile (35a)

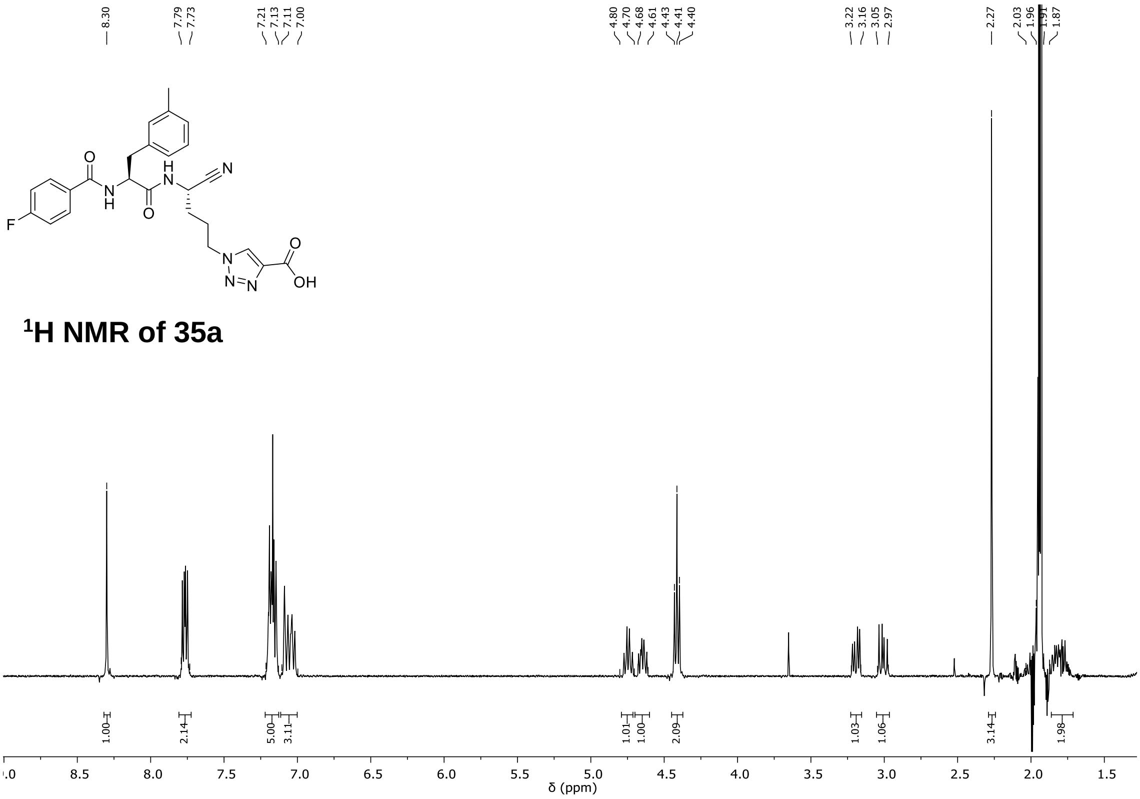

^{1}H NMR of 35a

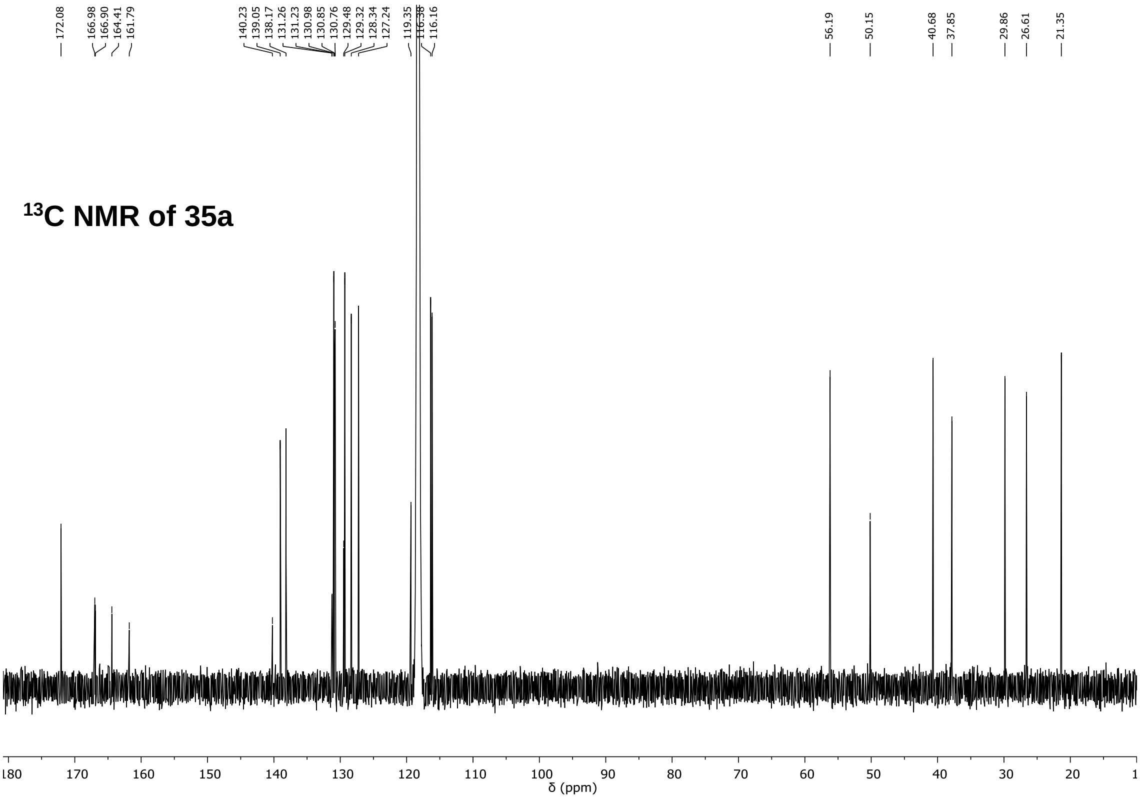

^{13}C NMR of 35a

N-(4-Fluorobenzoyl)-3-methyl-L-phenylalanyl-(4-carboxyethyl)-1*H*-1,2,3-triazole-1-yl)-L-norvaline nitrile (35b)

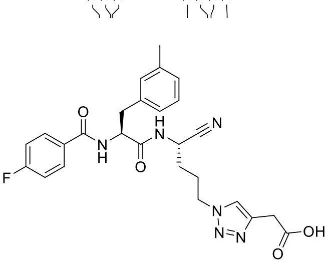

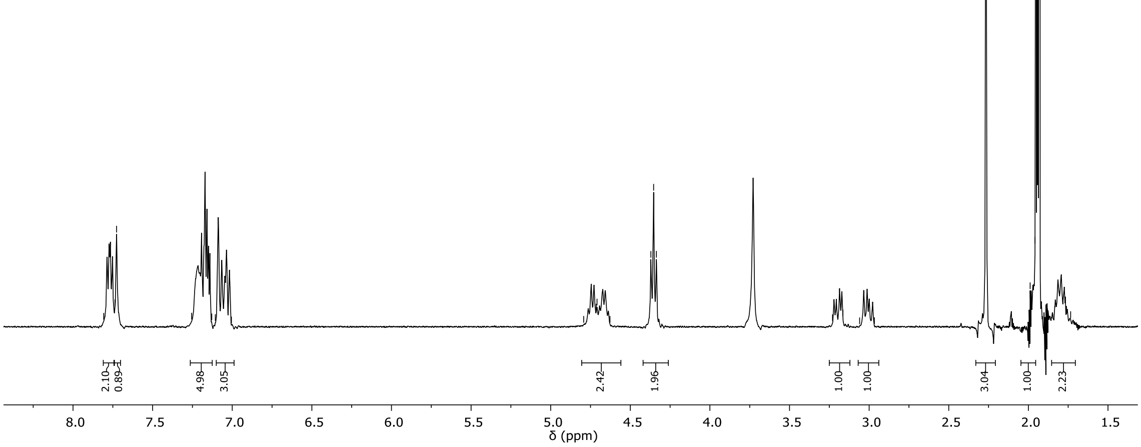

^{1}H NMR of 35b

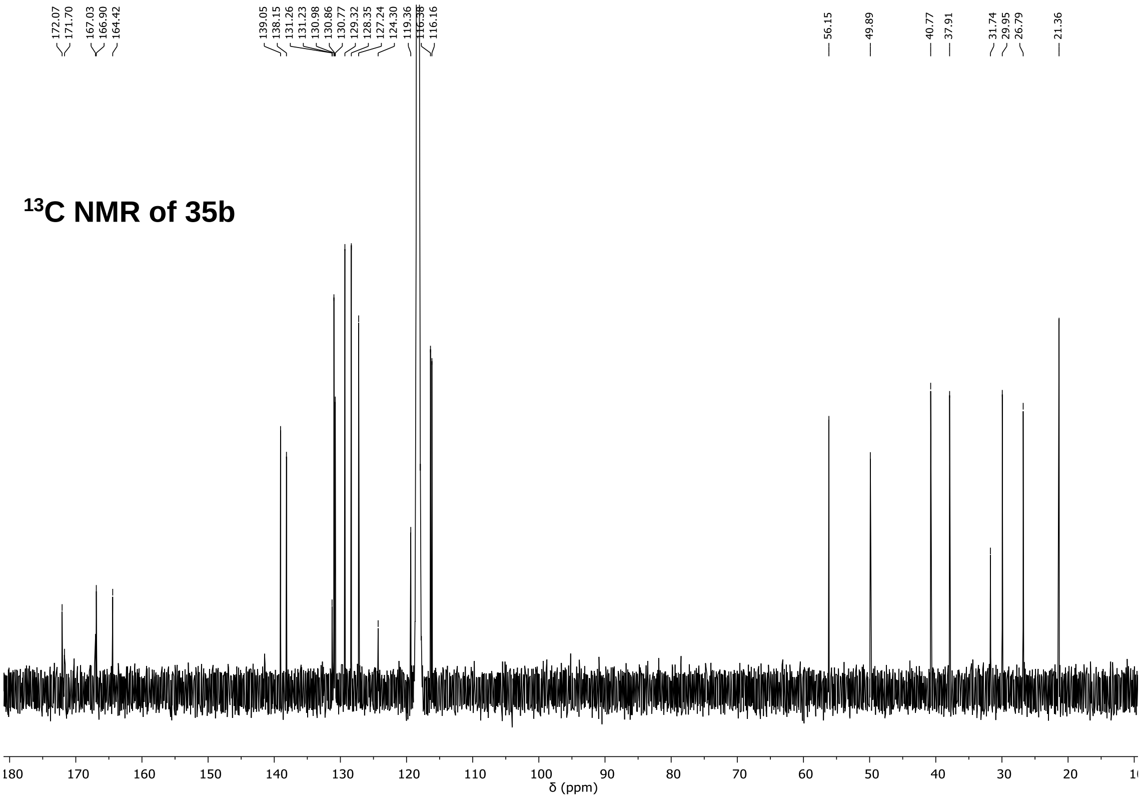

^{13}C NMR of 35b

N-(4-Fluorobenzoyl)-3-methyl-L-phenylalanyl-(4-carboxy-1*H*-1,2,3-triazole-1-yl)-L-norleucine alkyne (35c)

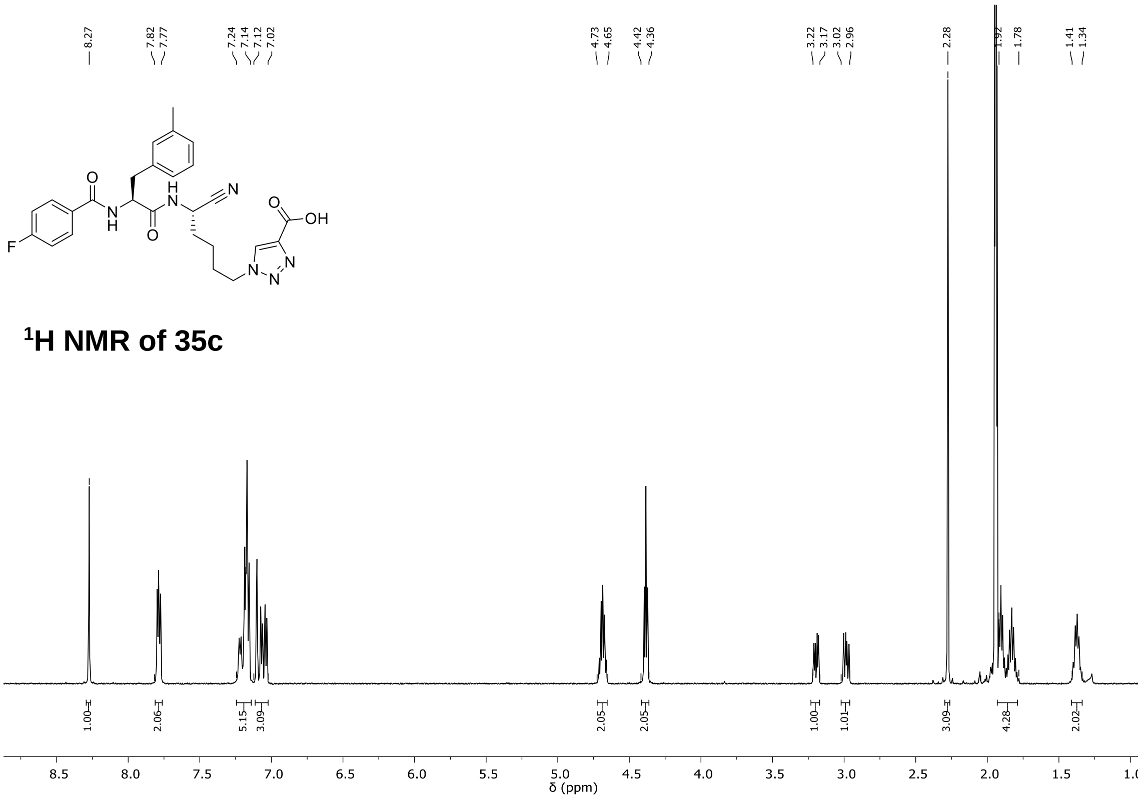

^{1}H NMR of 35c

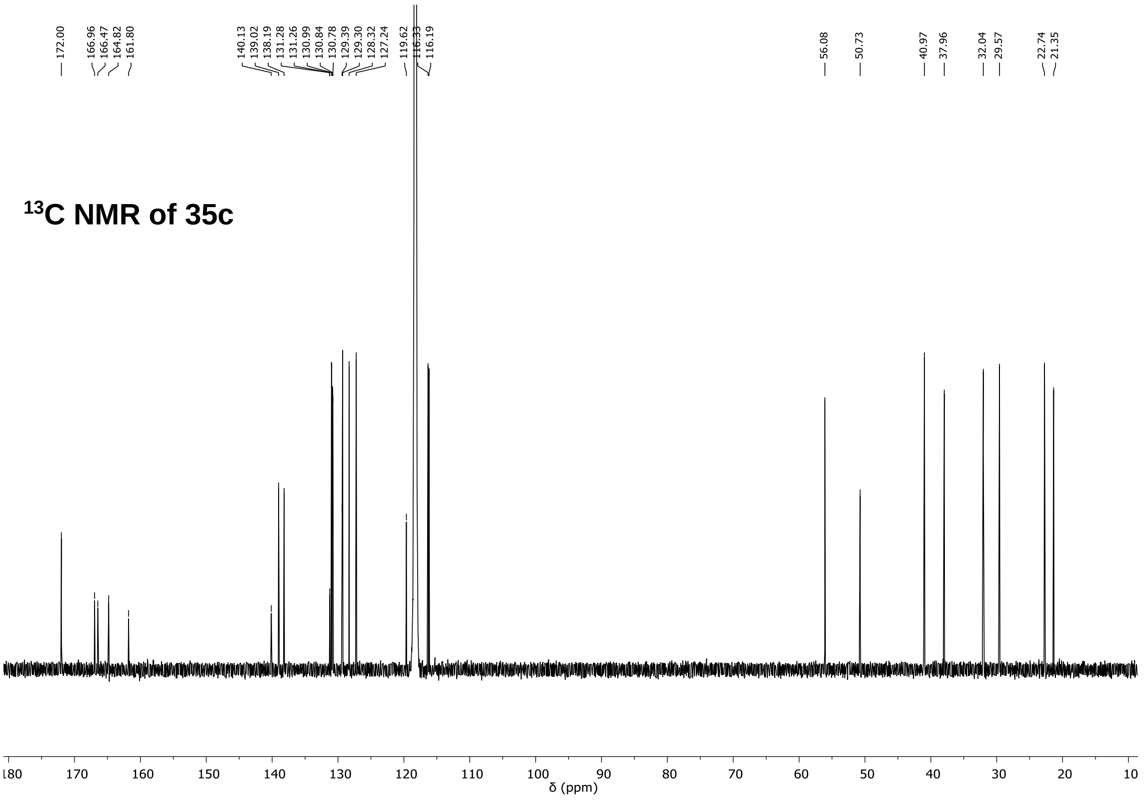

^{13}C NMR of 35c

5 Serine-based dipeptide nitriles 42 und 43 mit carboxy-functionalized 1,2,3-triazole ring in P2

*N-(*4-Fluorobenzoyl)-3-methyl-L-phenylalanyl-*O*-((1-(methylcarboxymethyl)-1*H*-1,2,3-triazol-4-yl)-methyl)-L-serine nitrile (42)

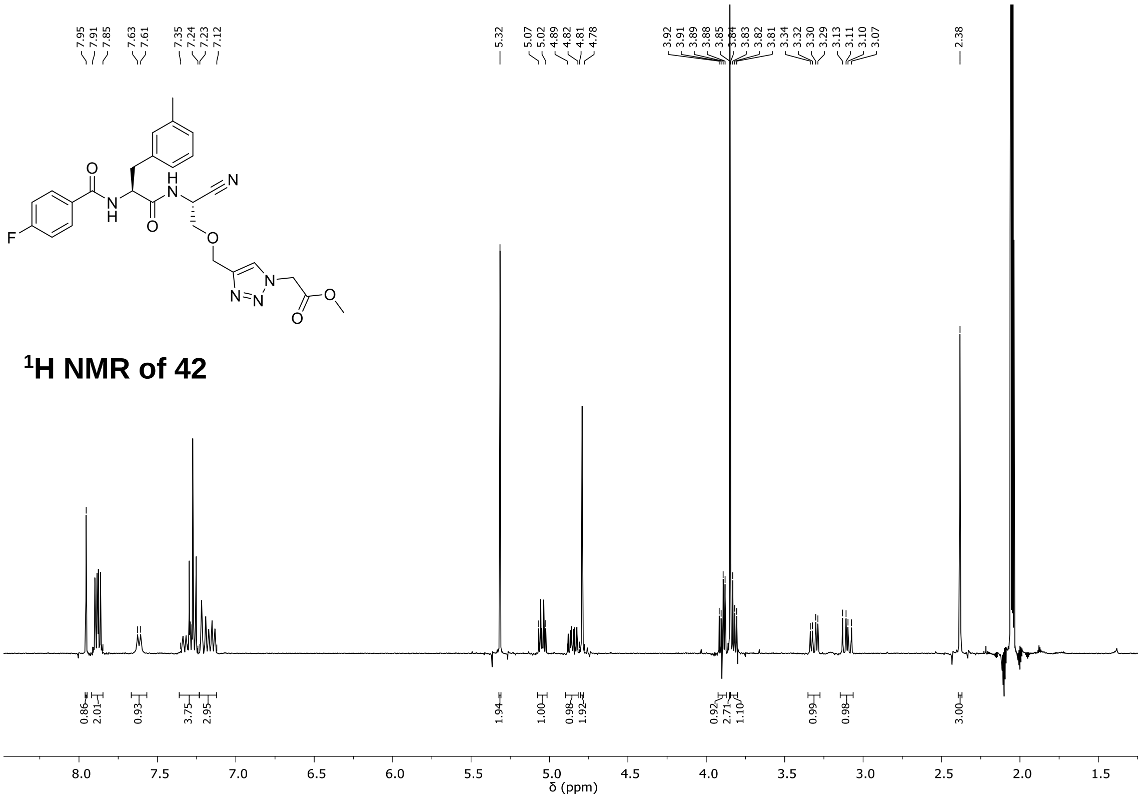

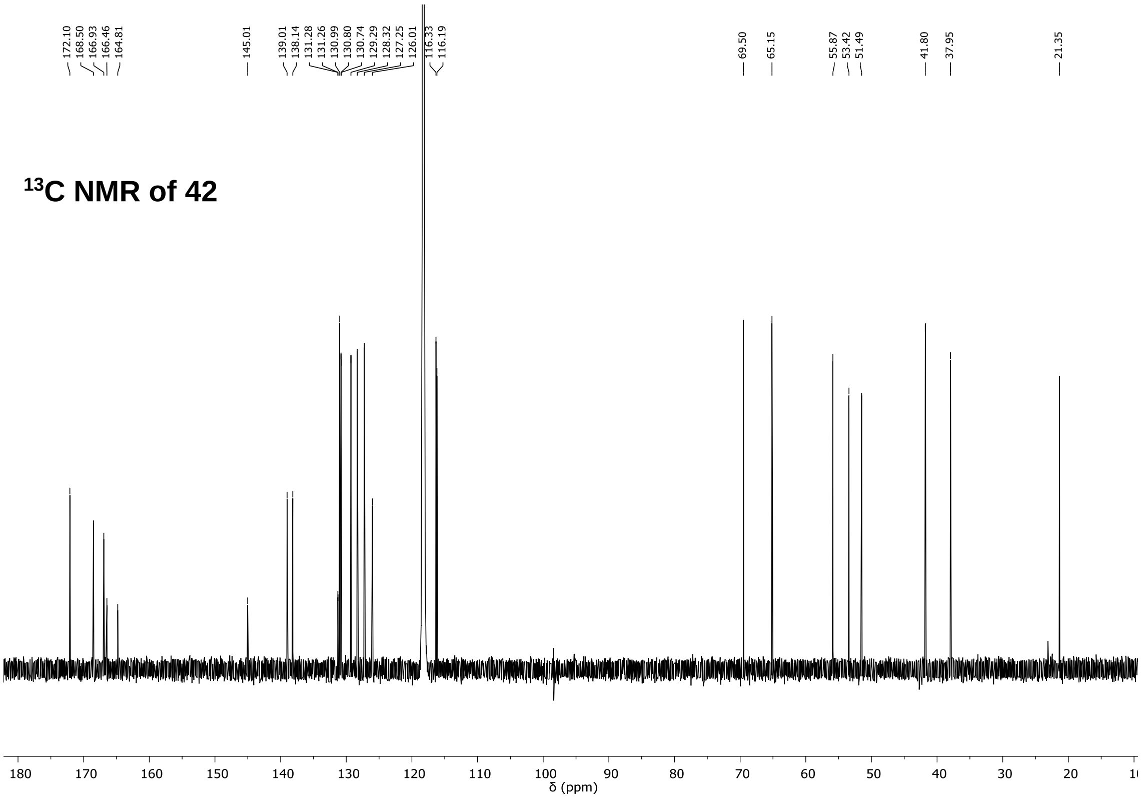

N-(4-Fluorobenzoyl)-3-methyl-L-phenylalanyl-O-((1-(carboxymethyl)-1H-1,2,3-triazole-4-yl)-methyl)-L-serine nitrile (43)

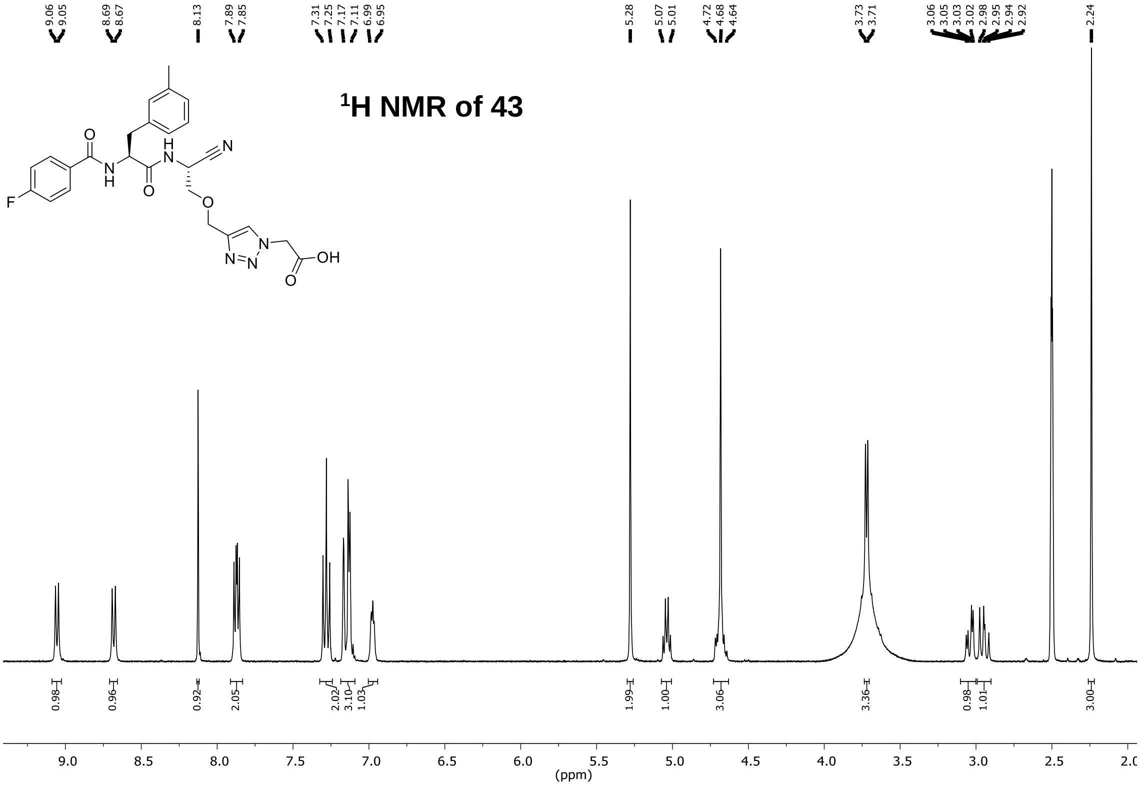

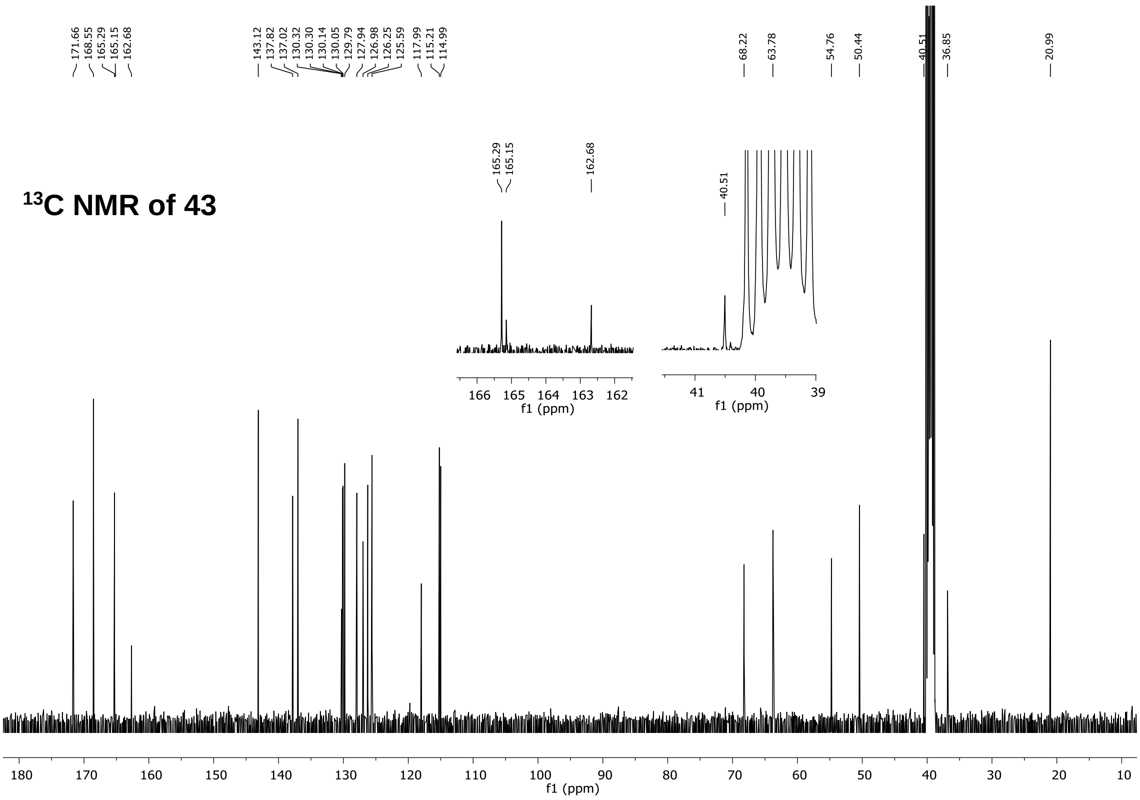

6 Dipeptide alkynes 2a – m

N-(2,4-Difluorobenzoyl)-3-methyl-L-phenylalanyl-*O*-(3-carboxybenzyl)serine alkyne (2a)

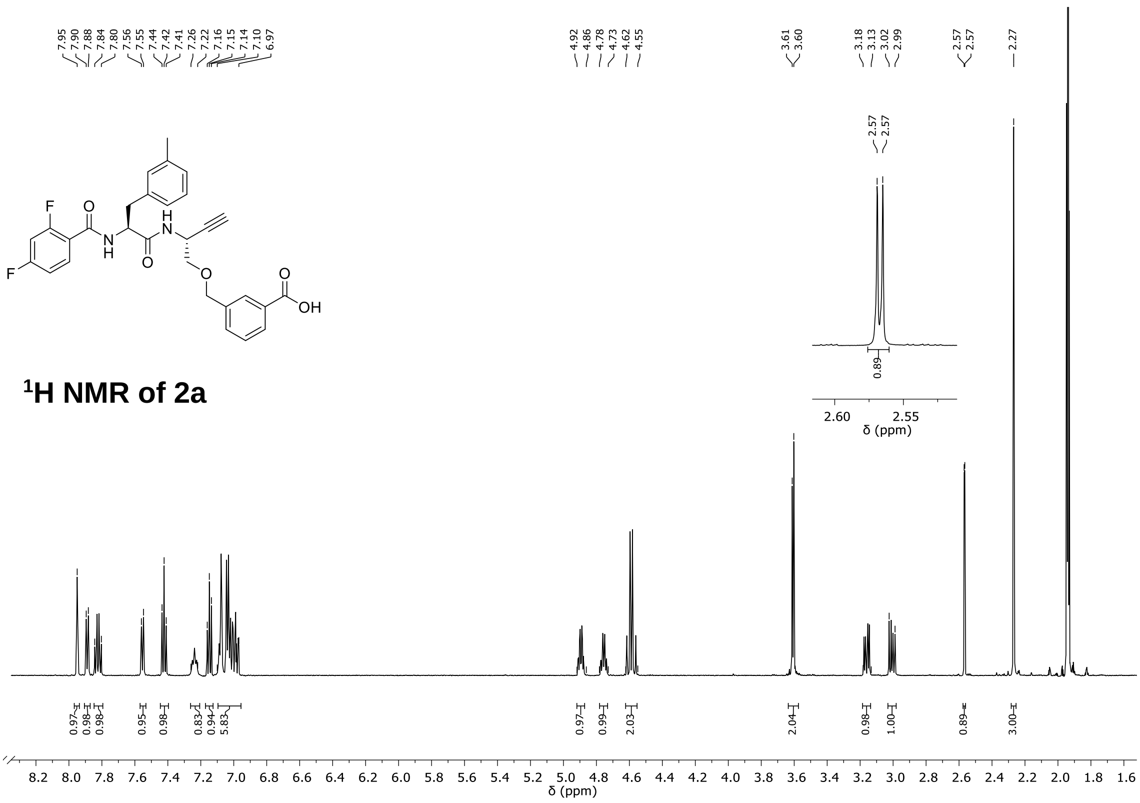

^{1}H NMR of 2a

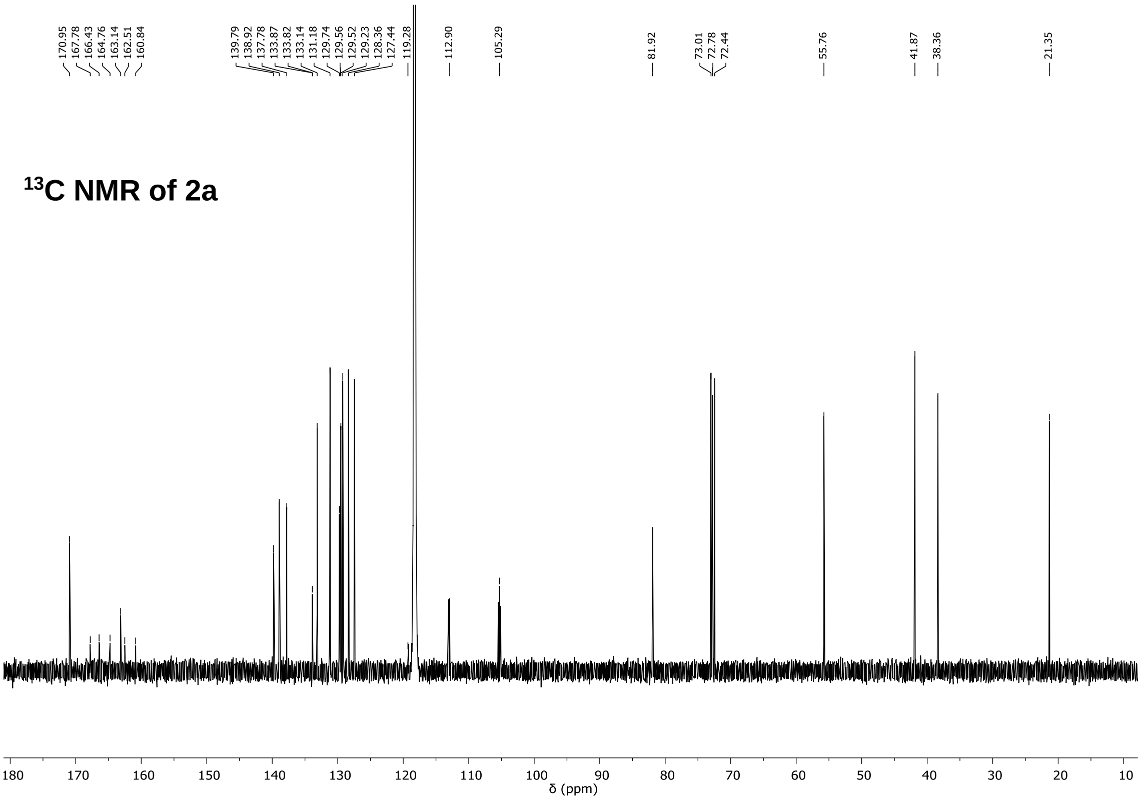

^{13}C NMR of 2a

N-(4-Fluorobenzoyl)-3-methyl-L-phenylalanyl-*O*-(3-carboxybenzyl)serine alkyne (2b)

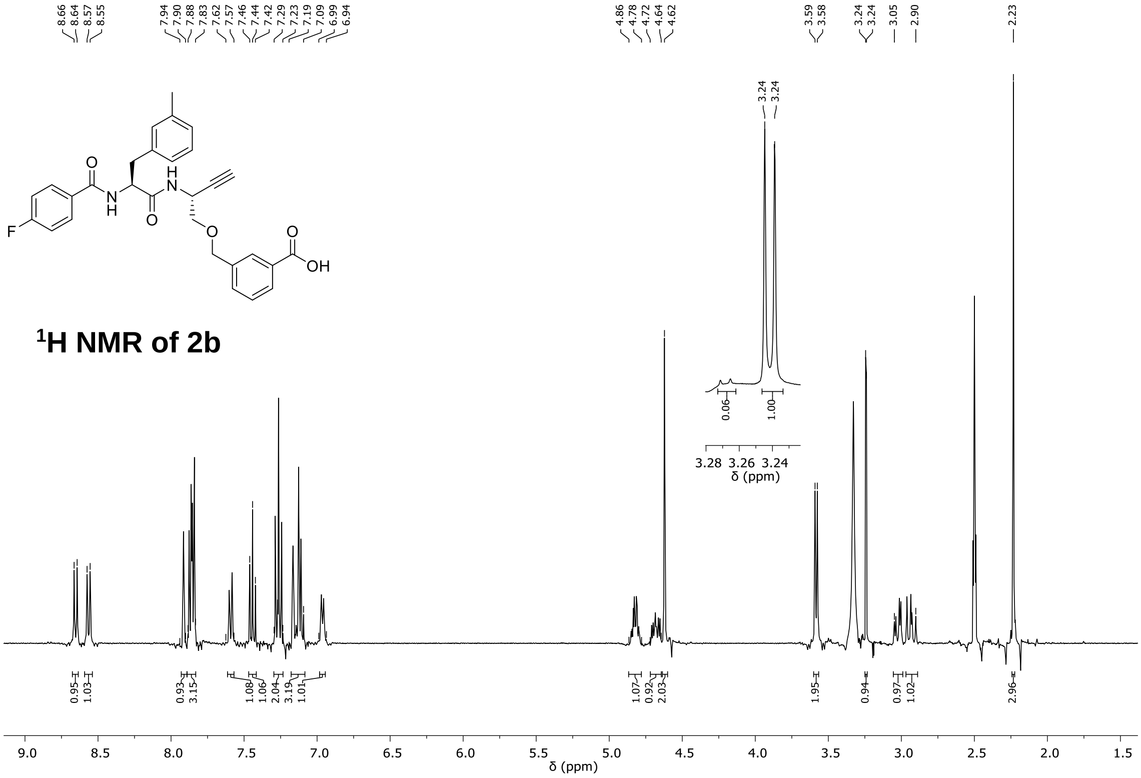

^{1}H NMR of 2b

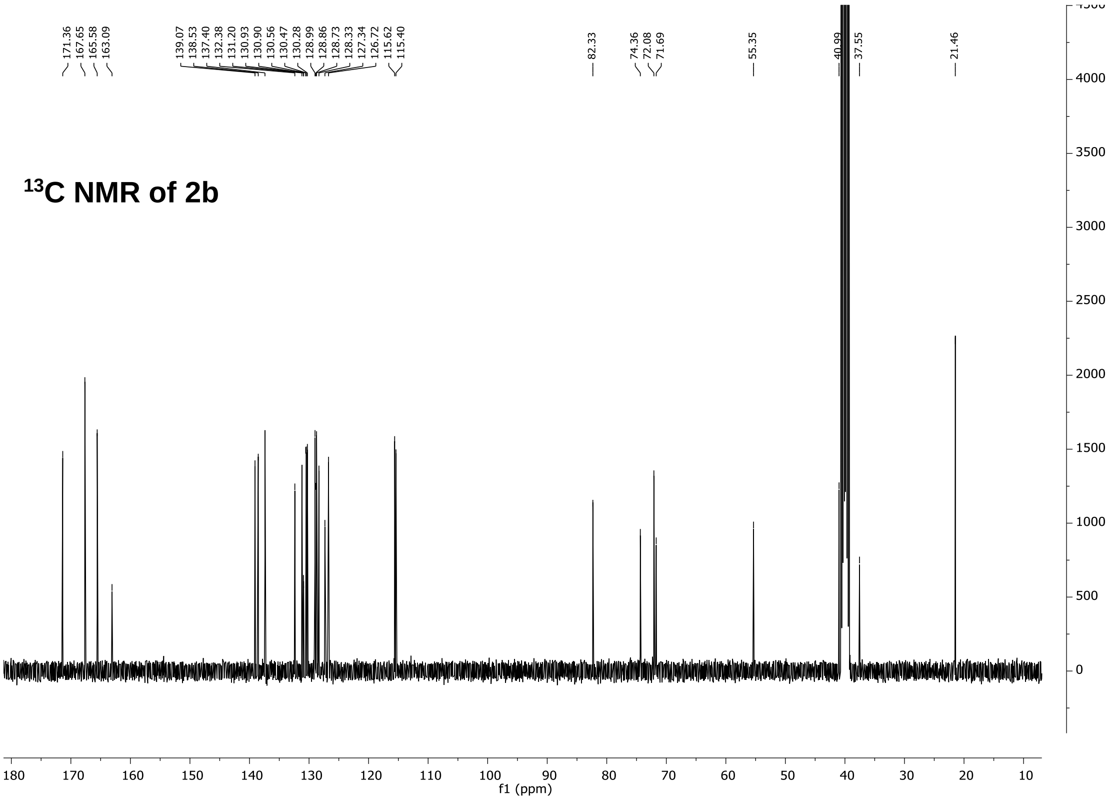

^{13}C NMR of 2b

N-Diphenylacetyl-3-methyl-L-phenylalanyl-*O*-(3-carboxybenzyl)-L-serine alkyne (2c)

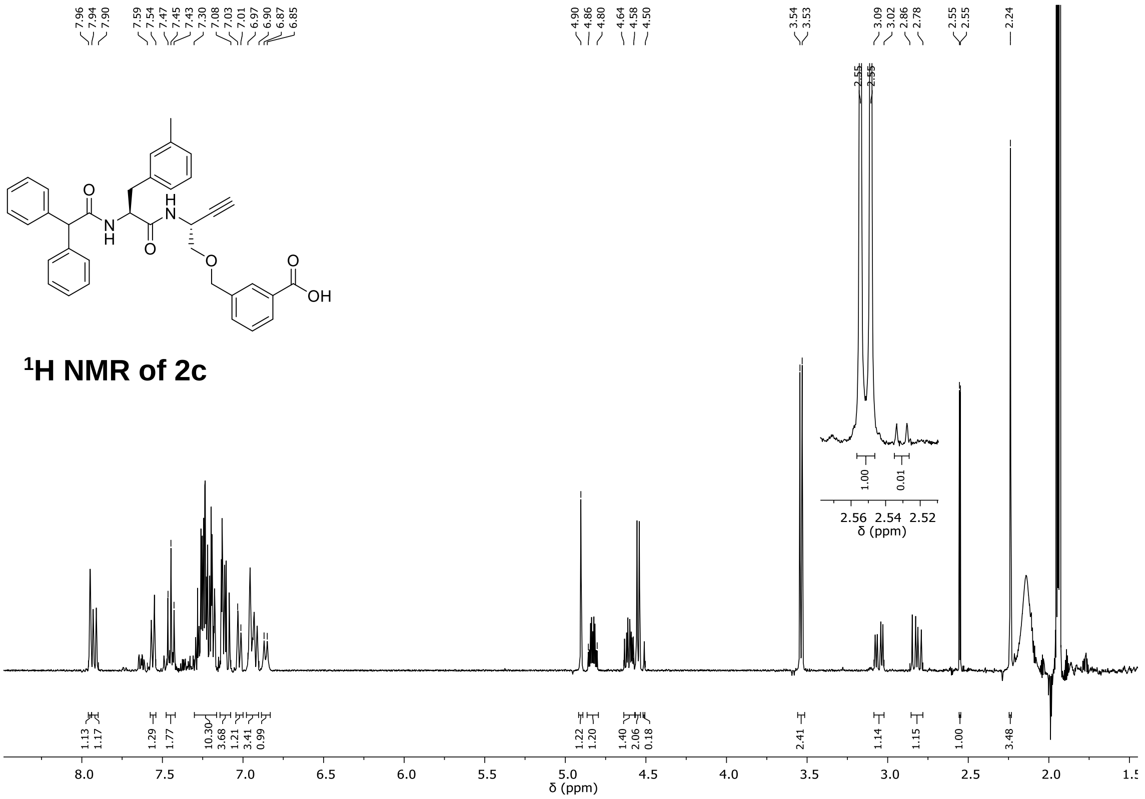

^{1}H NMR of 2c

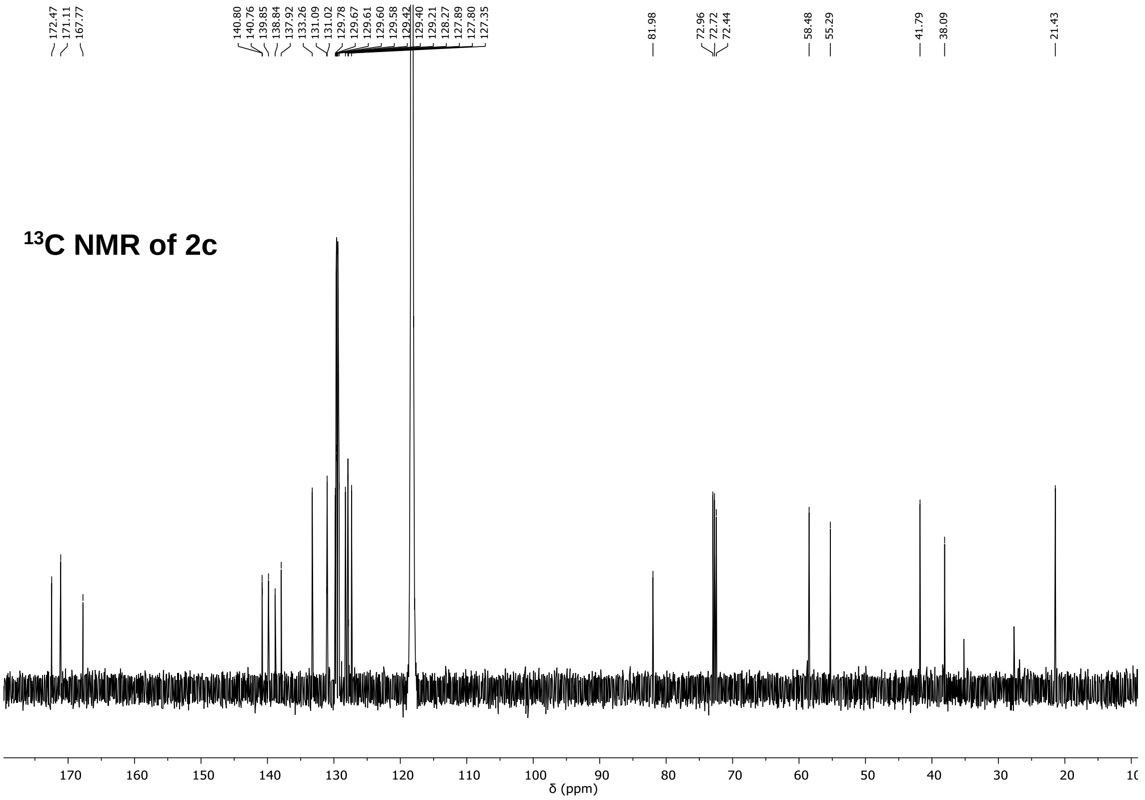

^{13}C NMR of 2c

N-(4-Phenylbenzoyl)-3-methyl-L-phenylalanyl-*O*-(3-carboxybenzyl)-L-serine alkyne (2d)

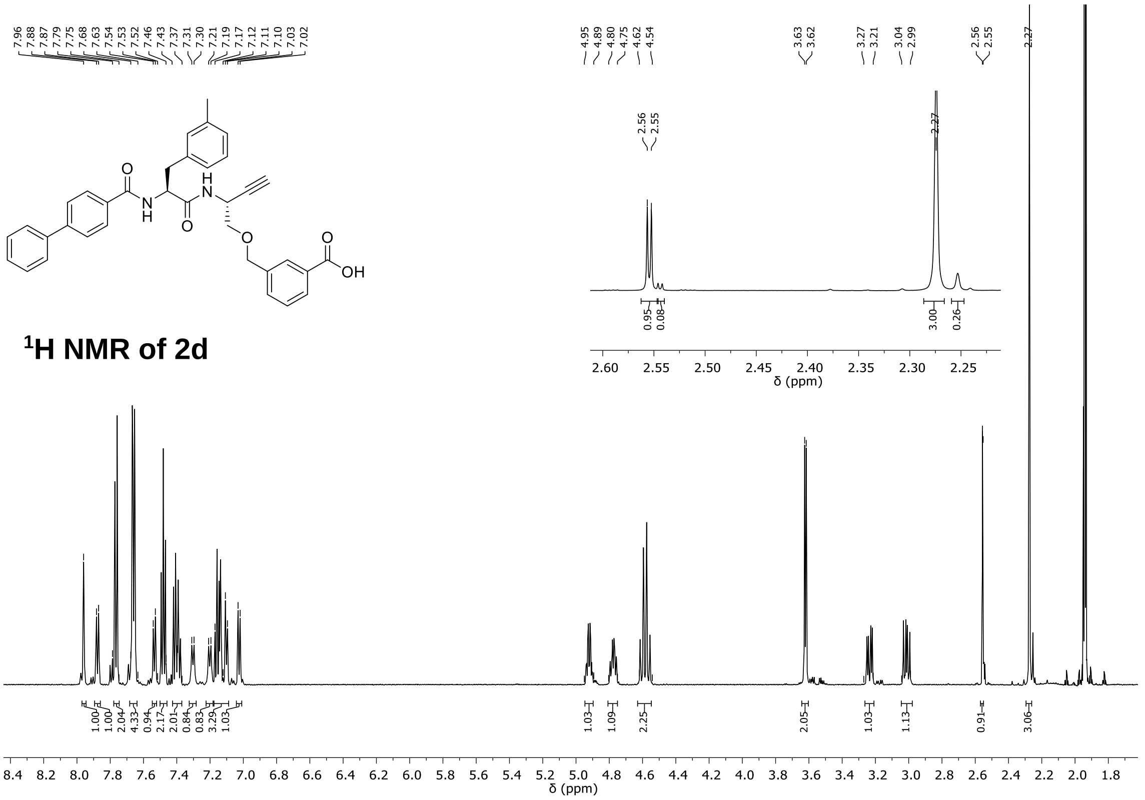

^{1}H NMR of 2d

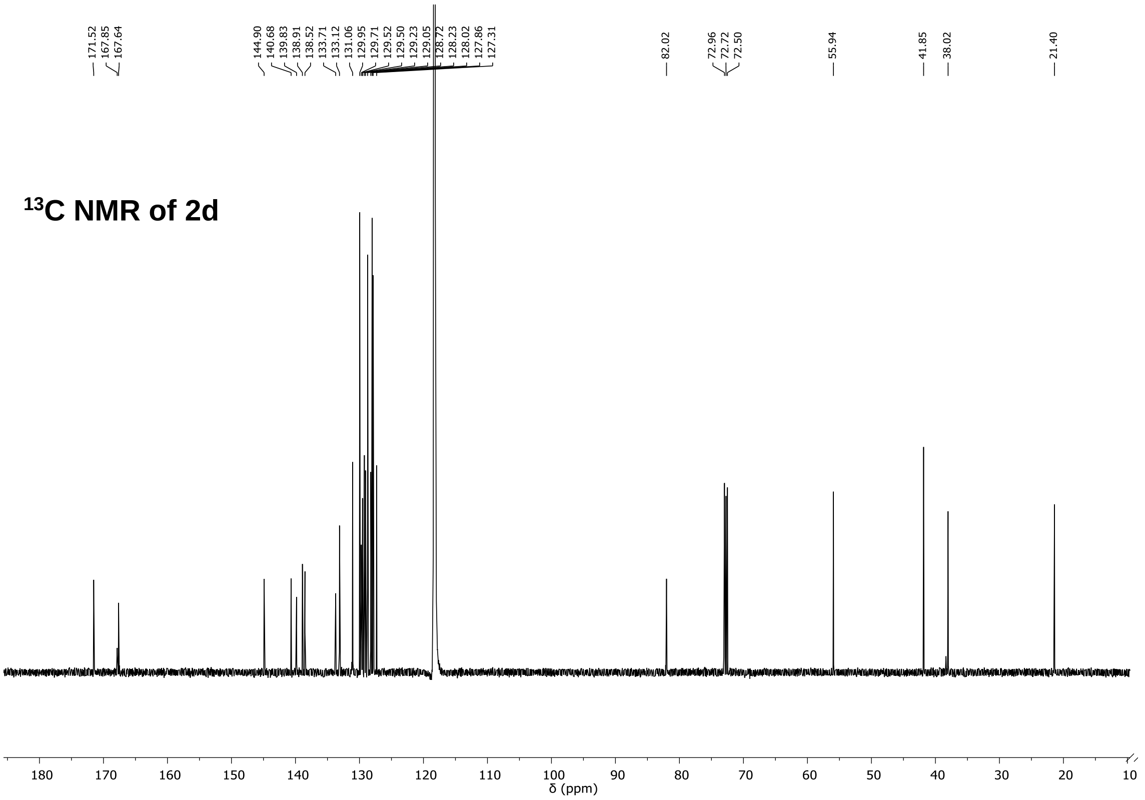

^{13}C NMR of 2d

N-(4-Iodobenzoyl)-3-methyl-L-phenylalanyl-*O*-(3-carboxybenzyl)-L-serine alkyne (2e)

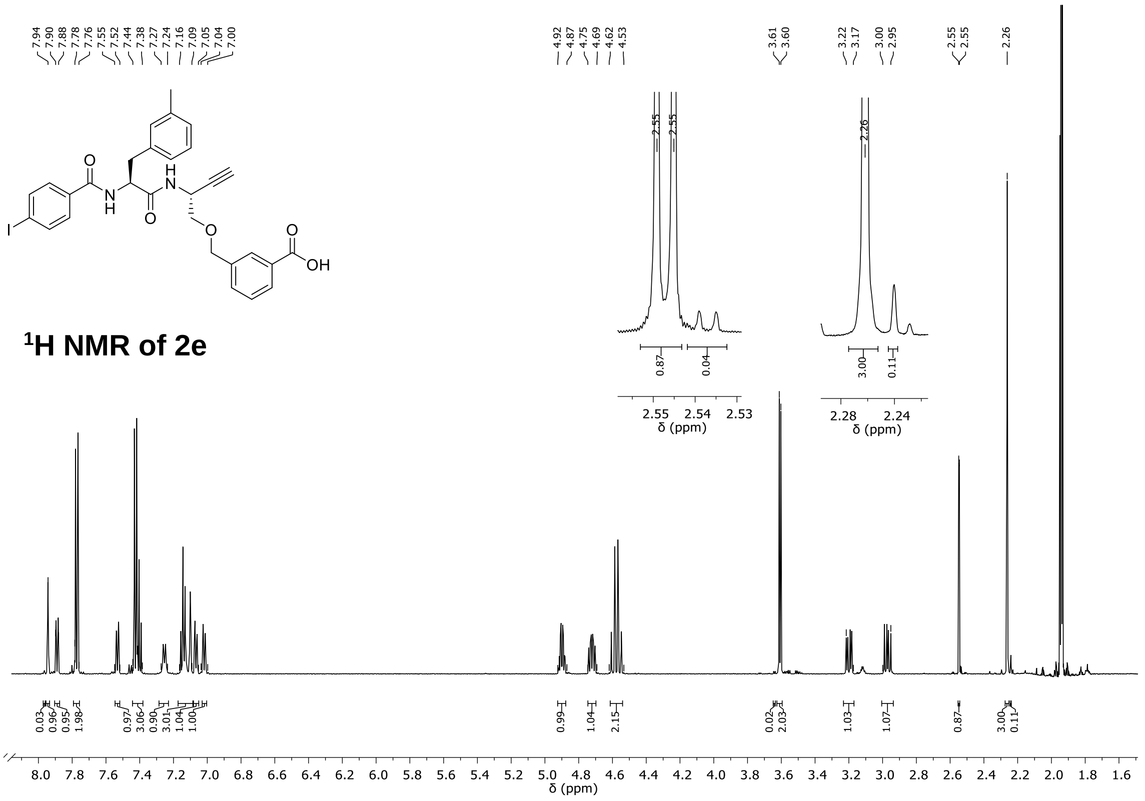

^{1}H NMR of 2e

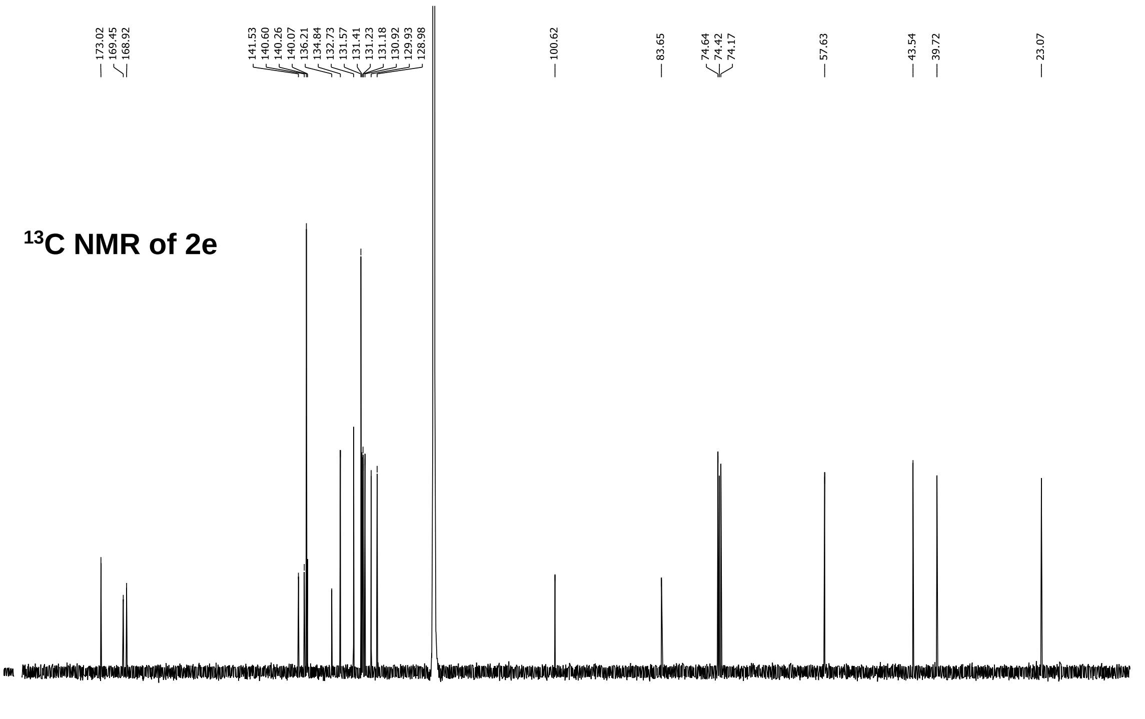

^{13}C NMR of 2e

N-(3-Iodobenzoyl)-3-methyl-L-phenylalanyl-*O*-(3-carboxybenzyl)-L-serine alkyne (2f)

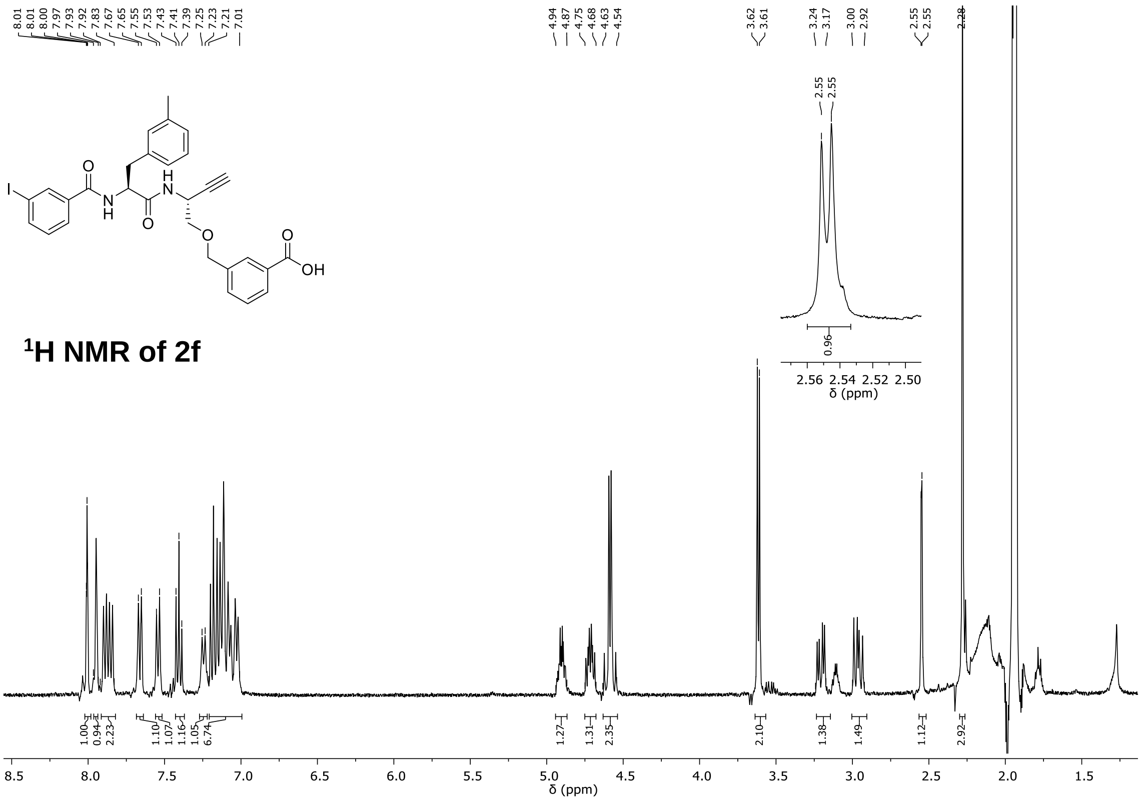

^{1}H NMR of 2f

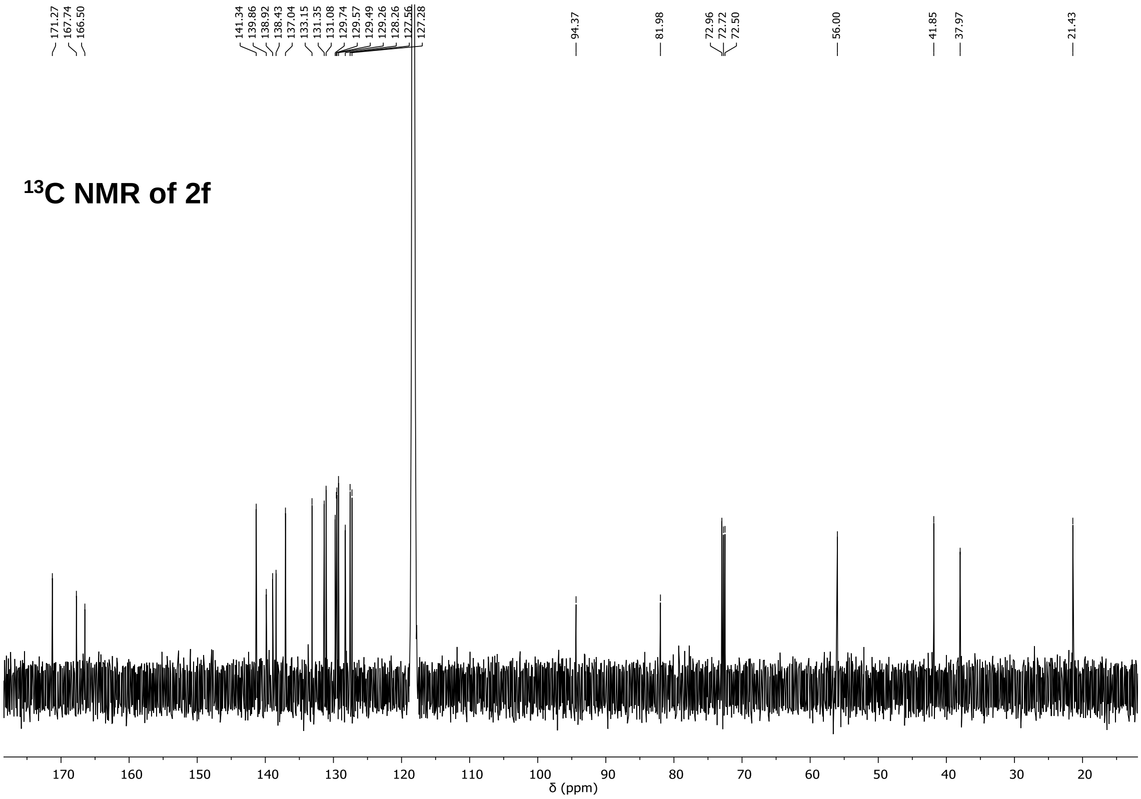

^{13}C NMR of 2f

N-Benzoyl-3-methyl-L-phenylalanyl-*O*-(3-carboxybenzyl)-L-serine alkyne (2g)

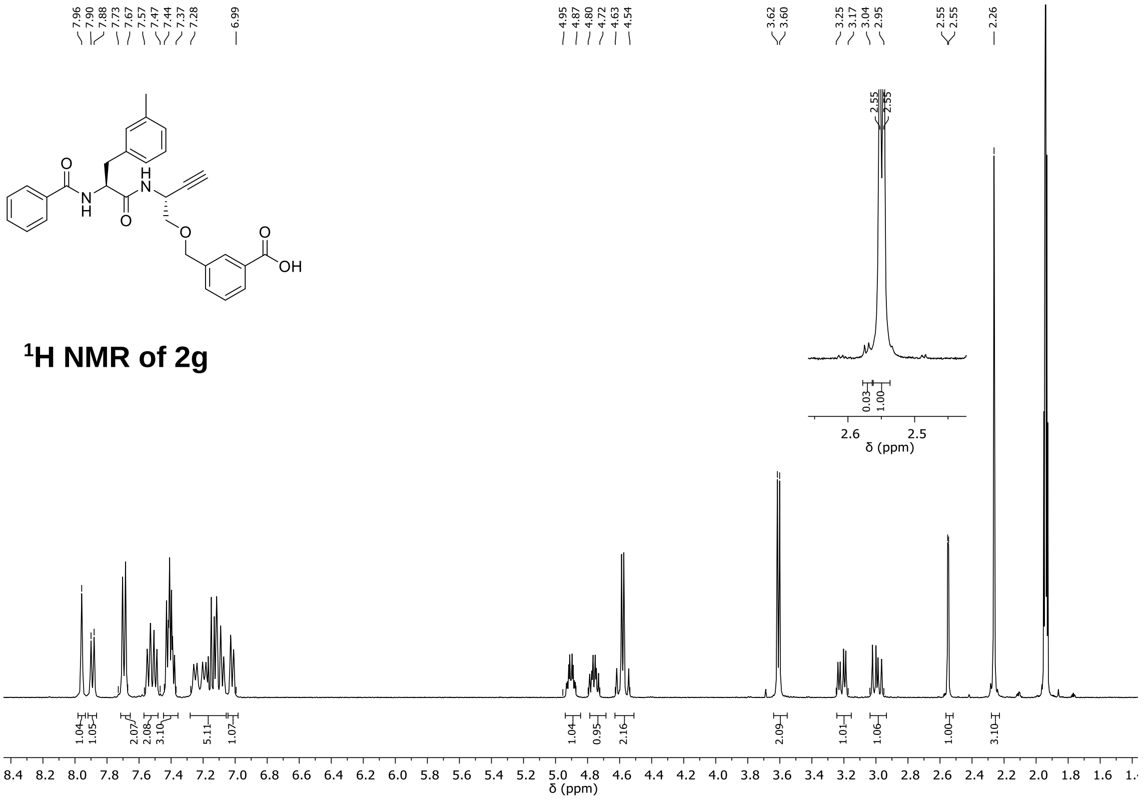

^{1}H NMR of 2g

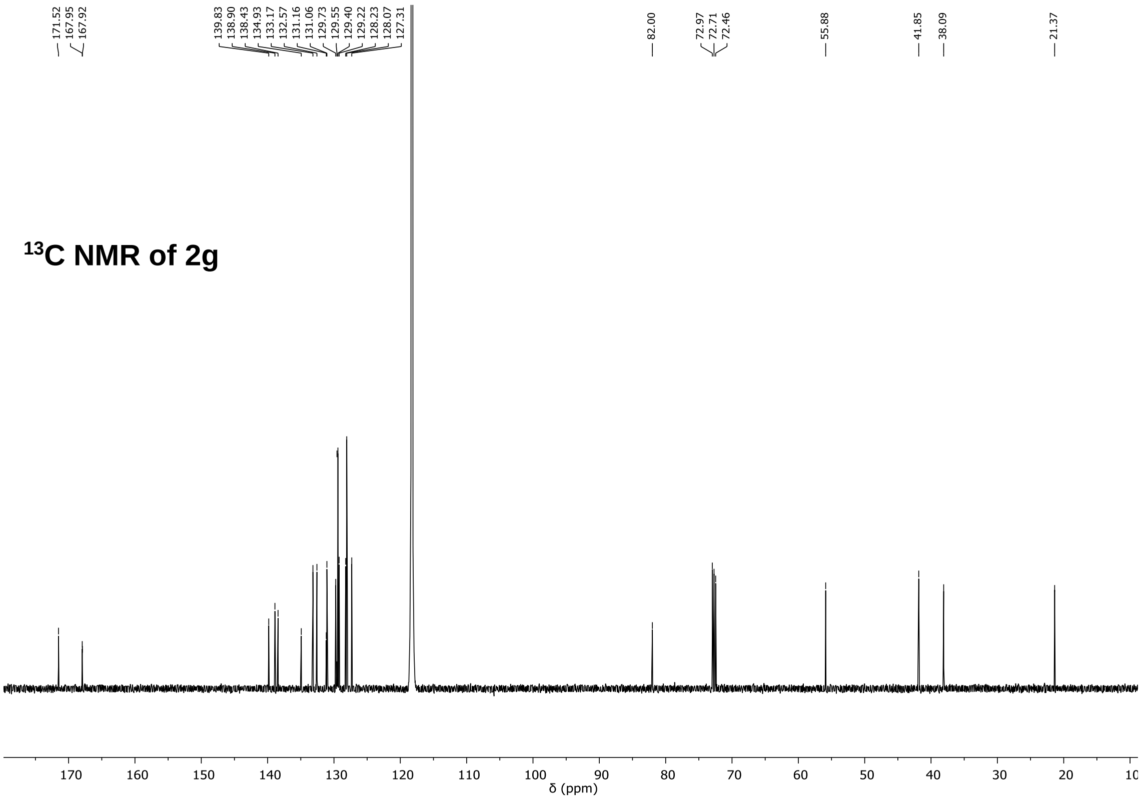

^{13}C NMR of 2g

N-(3-Fluorobenzoyl)-3-methyl-L-phenylalanyl-*O*-(3-carboxybenzyl)-L-serine alkyne (2h)

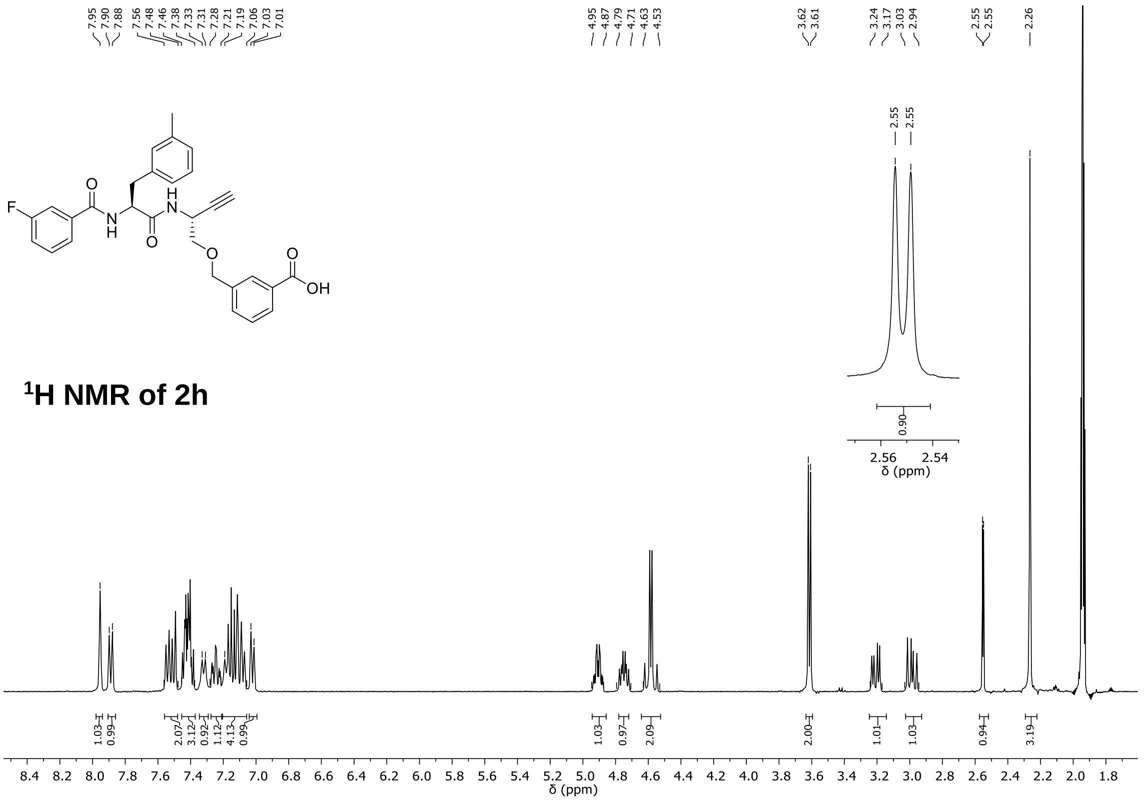

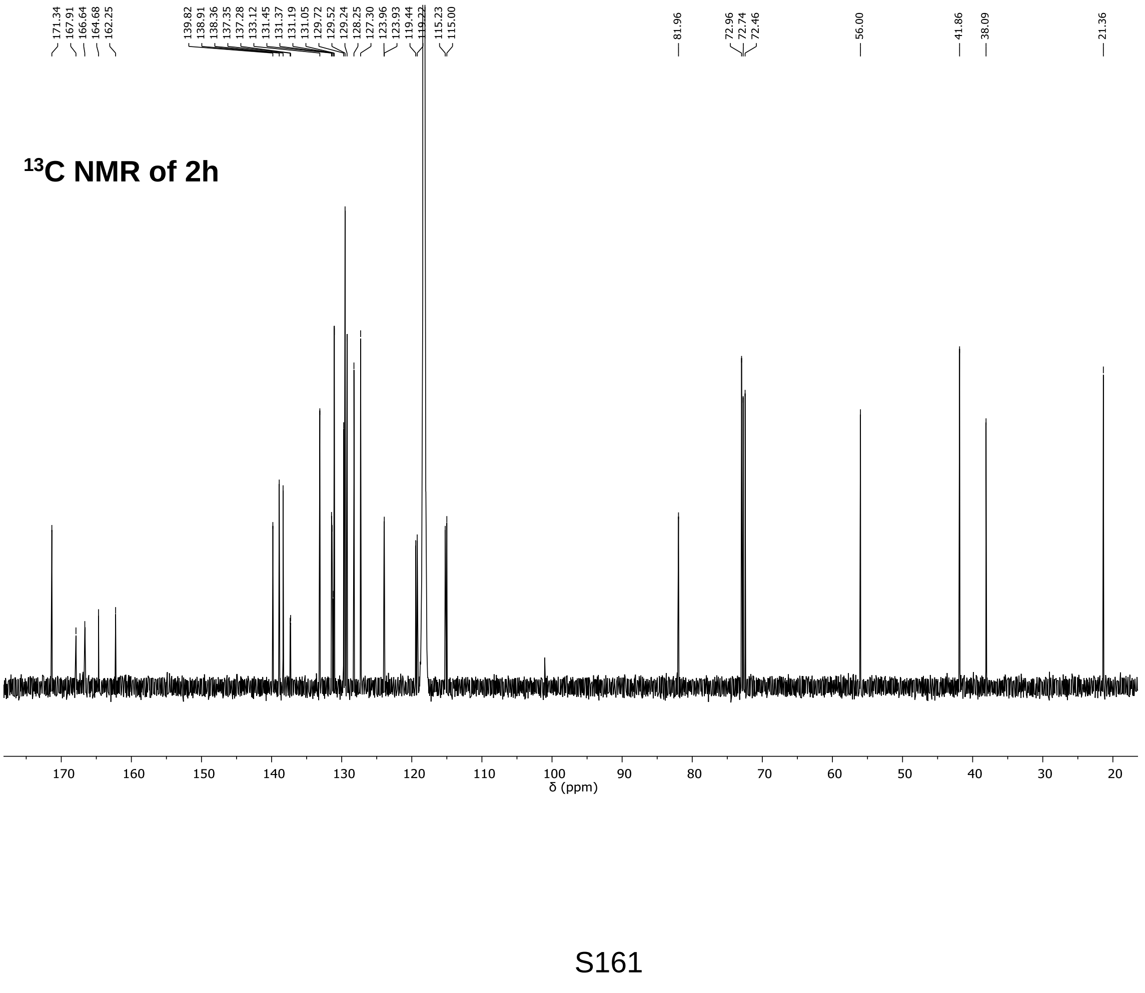

N-(3-Bromobenzoyl)-3-methyl-L-phenylalanyl-O-(3-carboxybenzyl)-L-serine alkyne (2i)

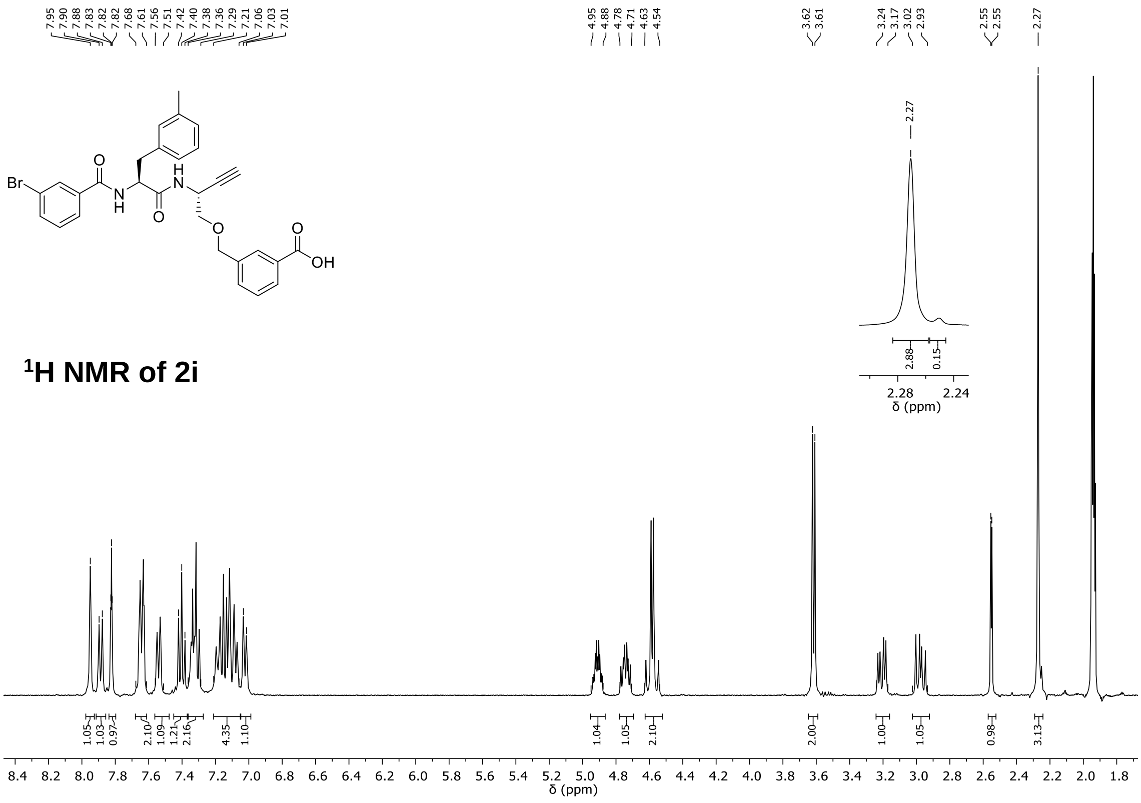

¹H NMR of 2i

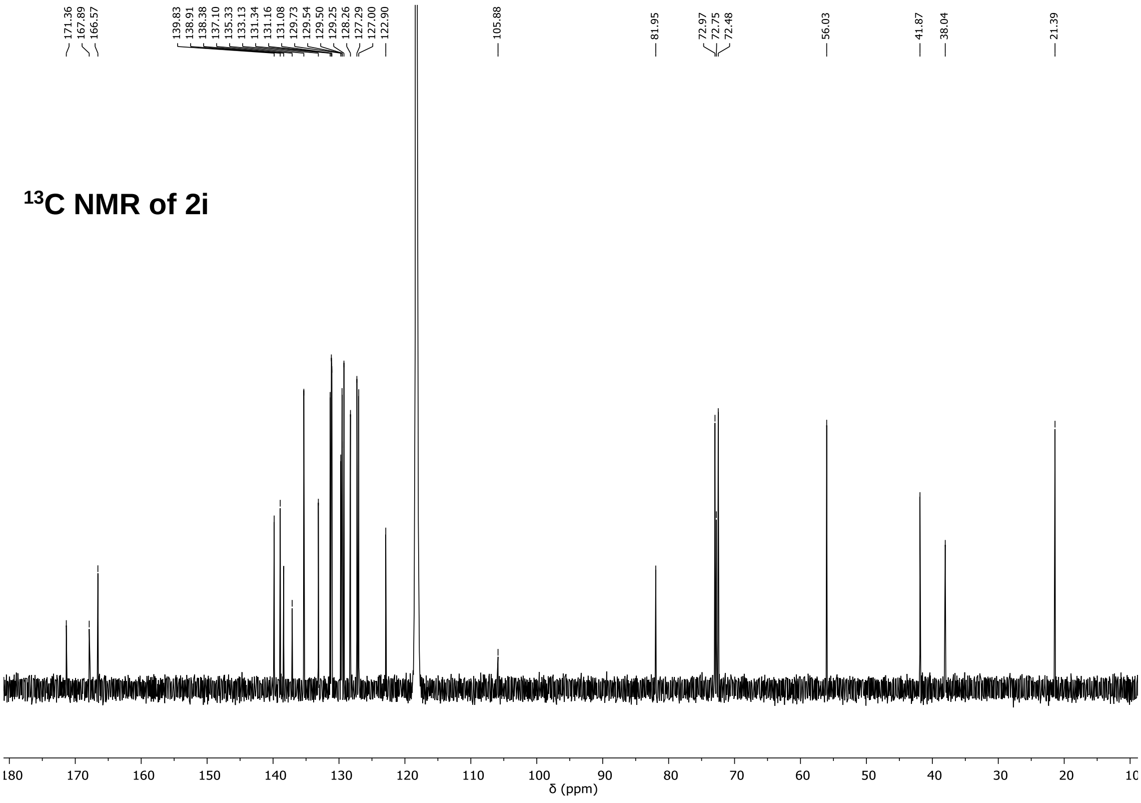

¹³C NMR of 2i

_N_-3-(Trifluoromethyl)benzoyl-3-methyl-L-phenylalanyl-_O_-(3-carboxybenzyl)-L-serine alkyne (2j)

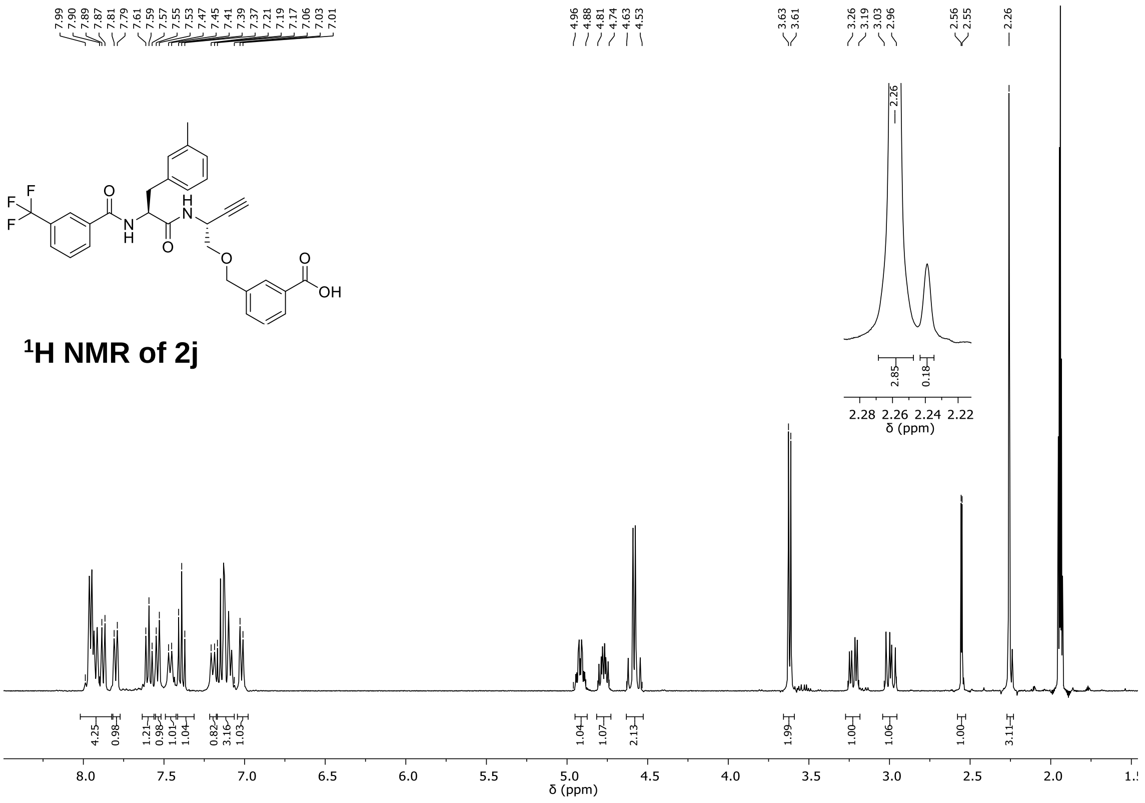

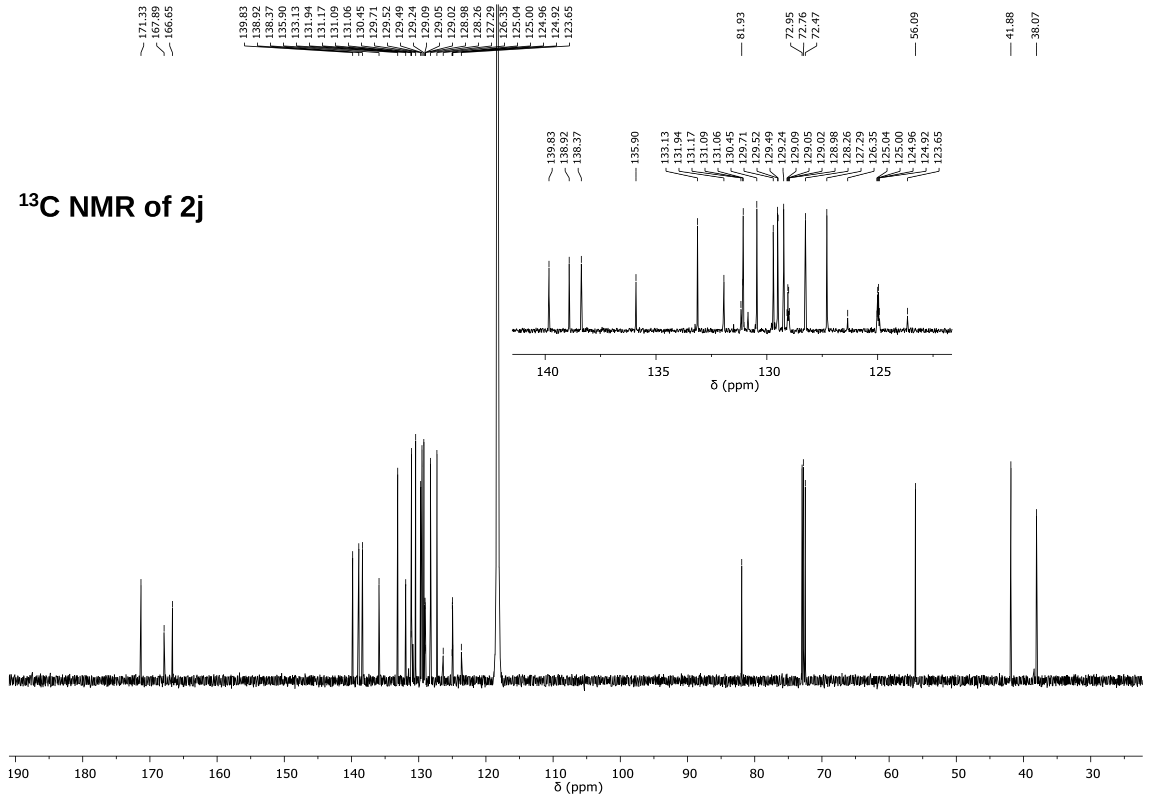

***N*-(4-Fluorobenzoyl)-3-iodo-L-phenylalanyl-*O*-(3-(allyloxycarbonyl)benzyl)-L-serine alkyne (2k)**

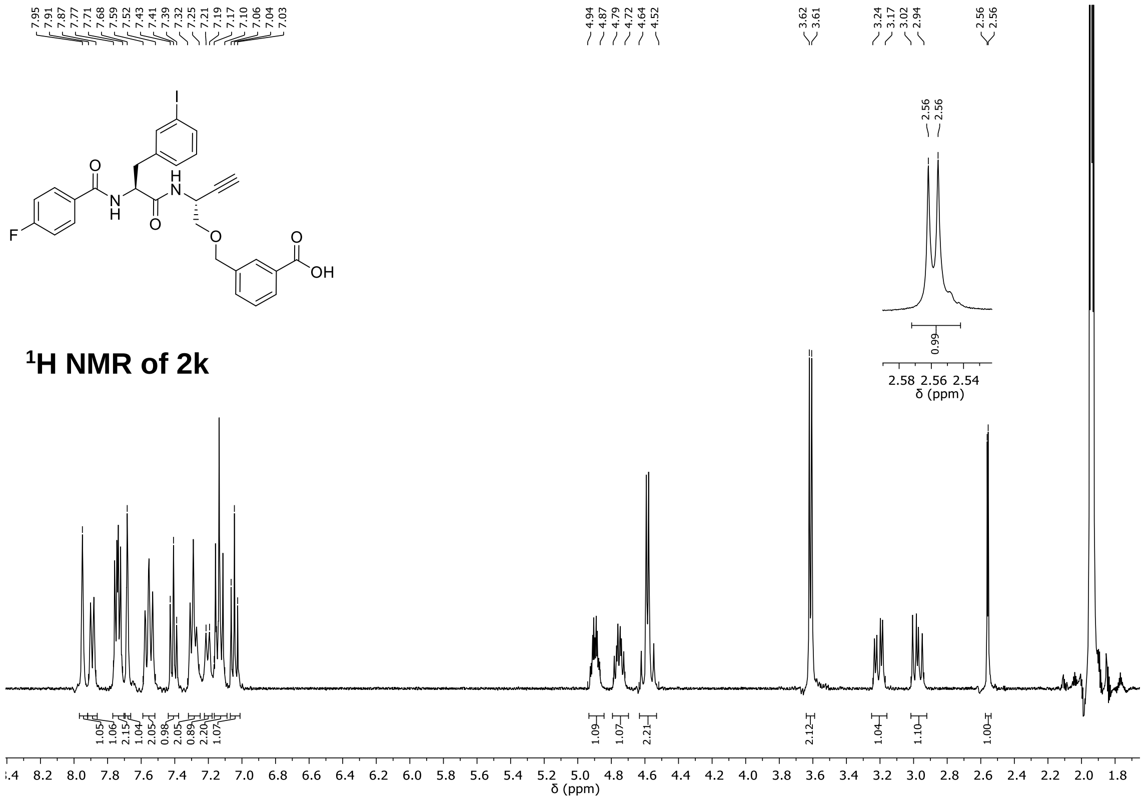

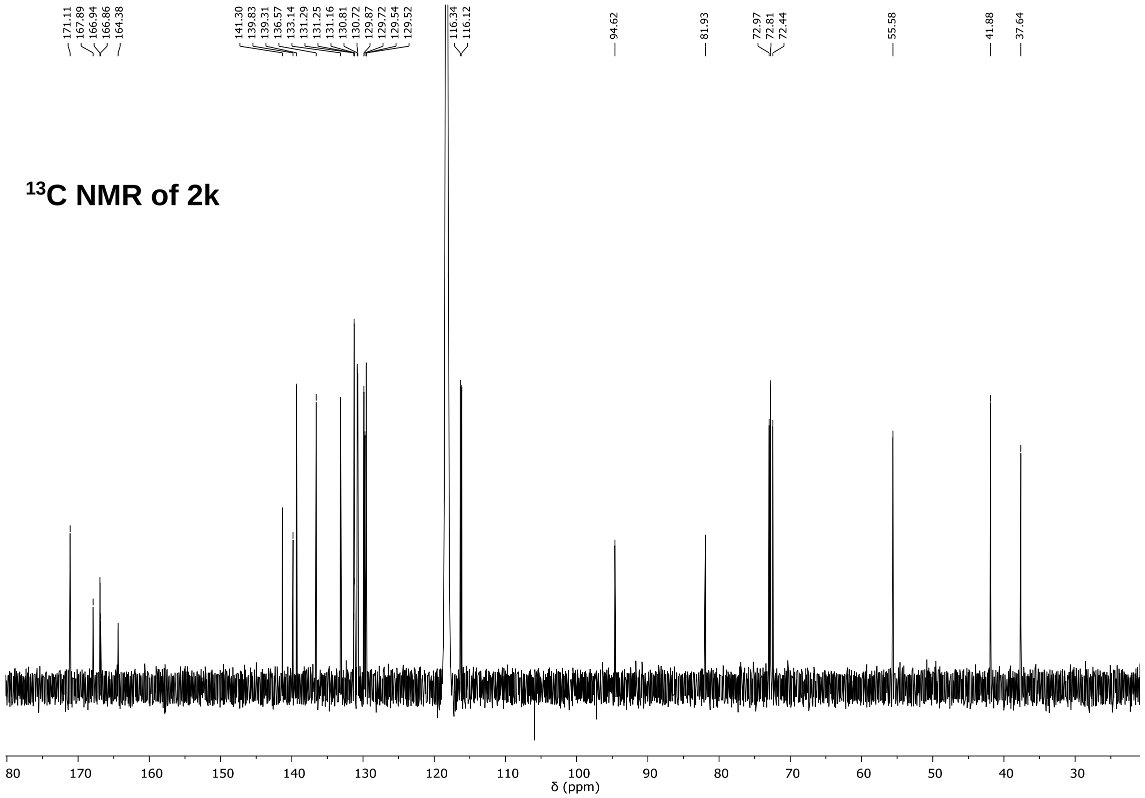

N-Diphenylacetyl-3-iodo-L-phenylalanyl-*O*-(3-carboxybenzyl)-L-serine alkyne (2l)

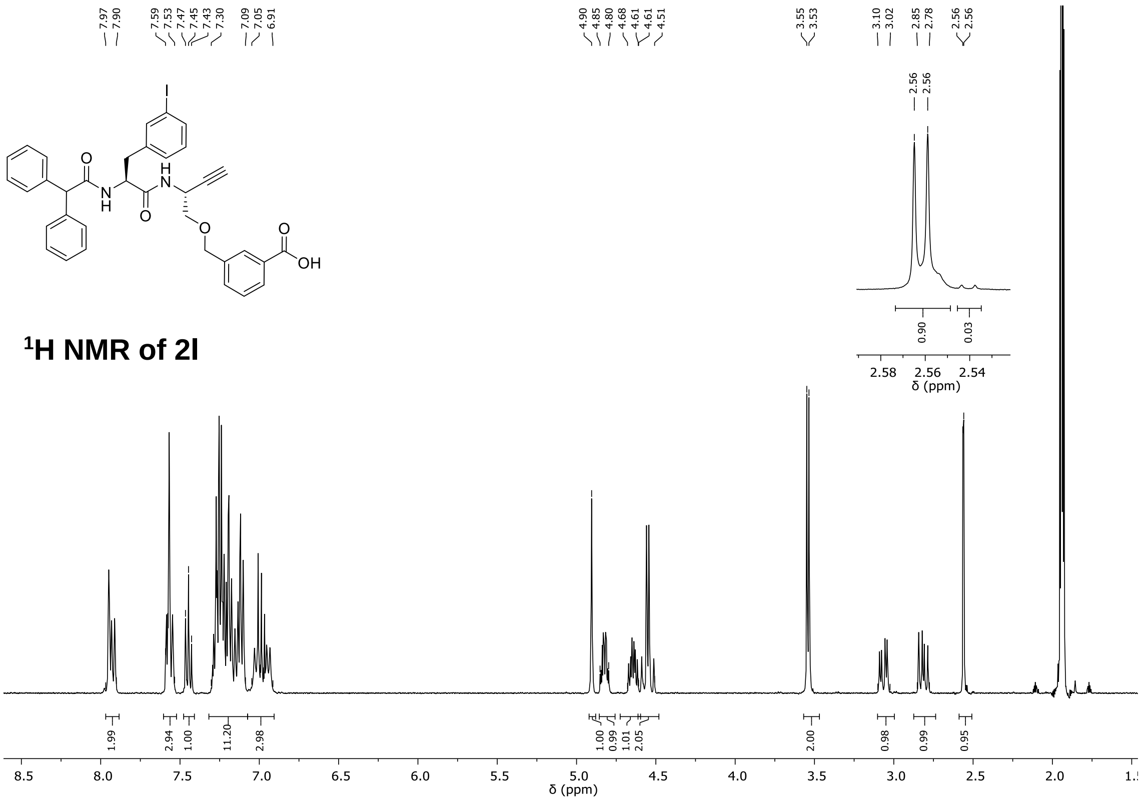

^{1}H NMR of 2l

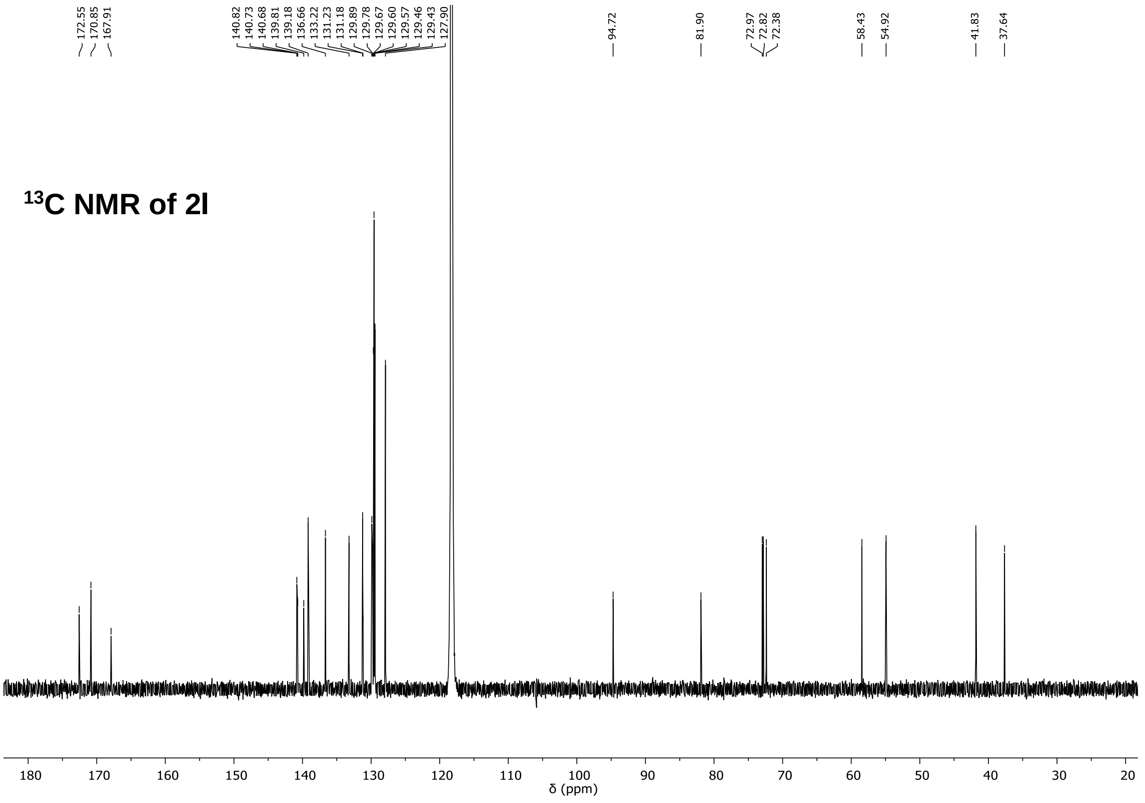

^{13}C NMR of 2l

N-(3-Iodobenzoyl)-3-iodo-L-phenylalanyl-*O*-(3-carboxybenzyl)-L-serine alkyne (2m)

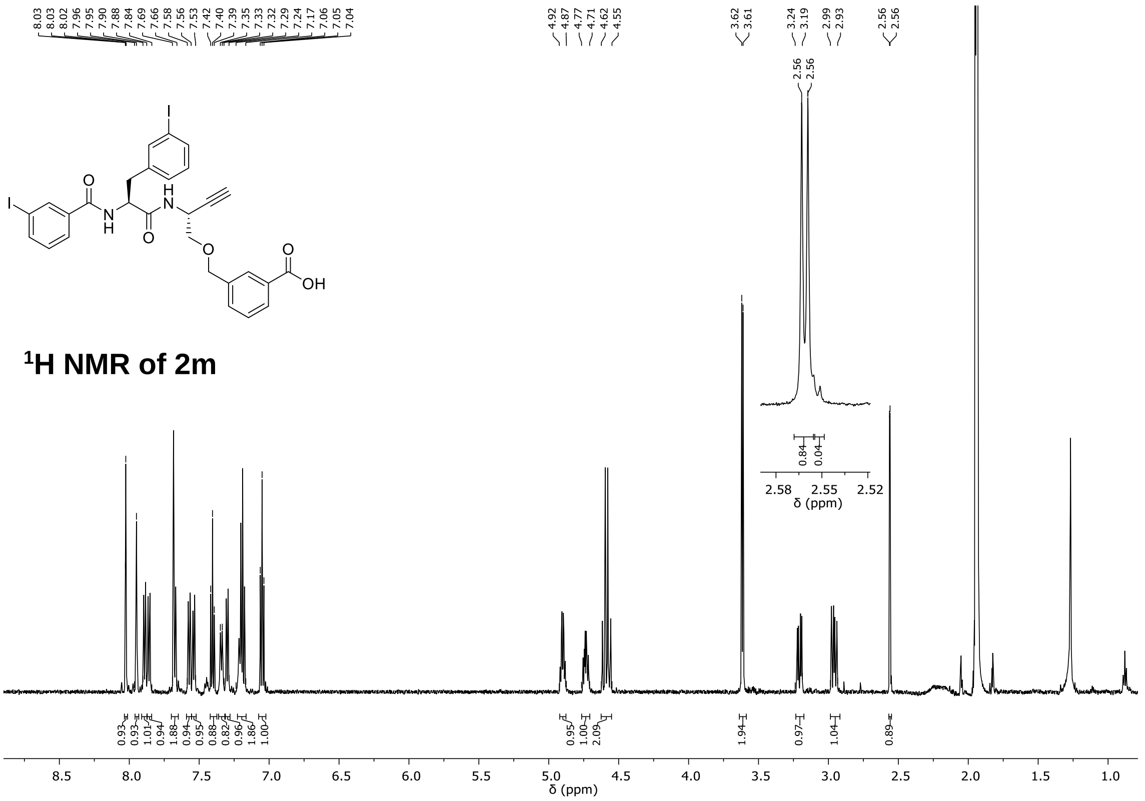

^{1}H NMR of 2m

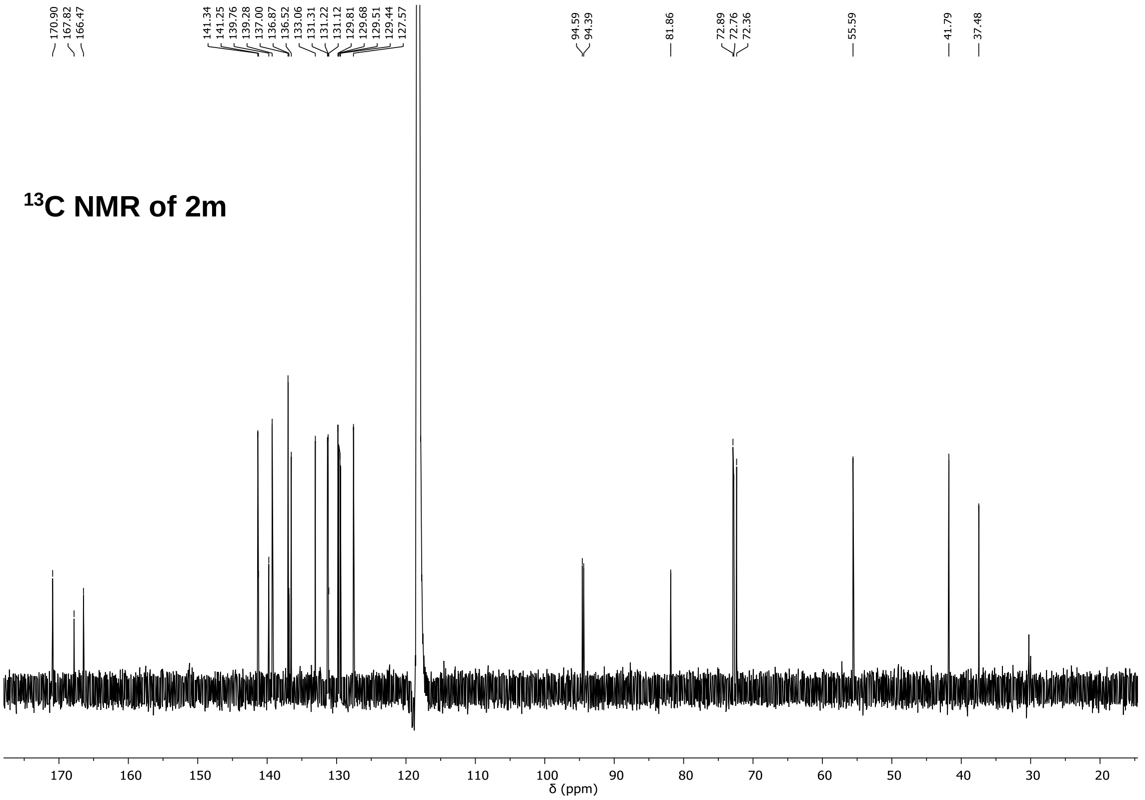

^{13}C NMR of 2m

7 Dipeptide nitriles and alkynes containing Gly in P1 (56a – f, 62)

N-(4-Fluorobenzoyl)-3-methyl-L-phenylalanylglycine nitrile (56a)

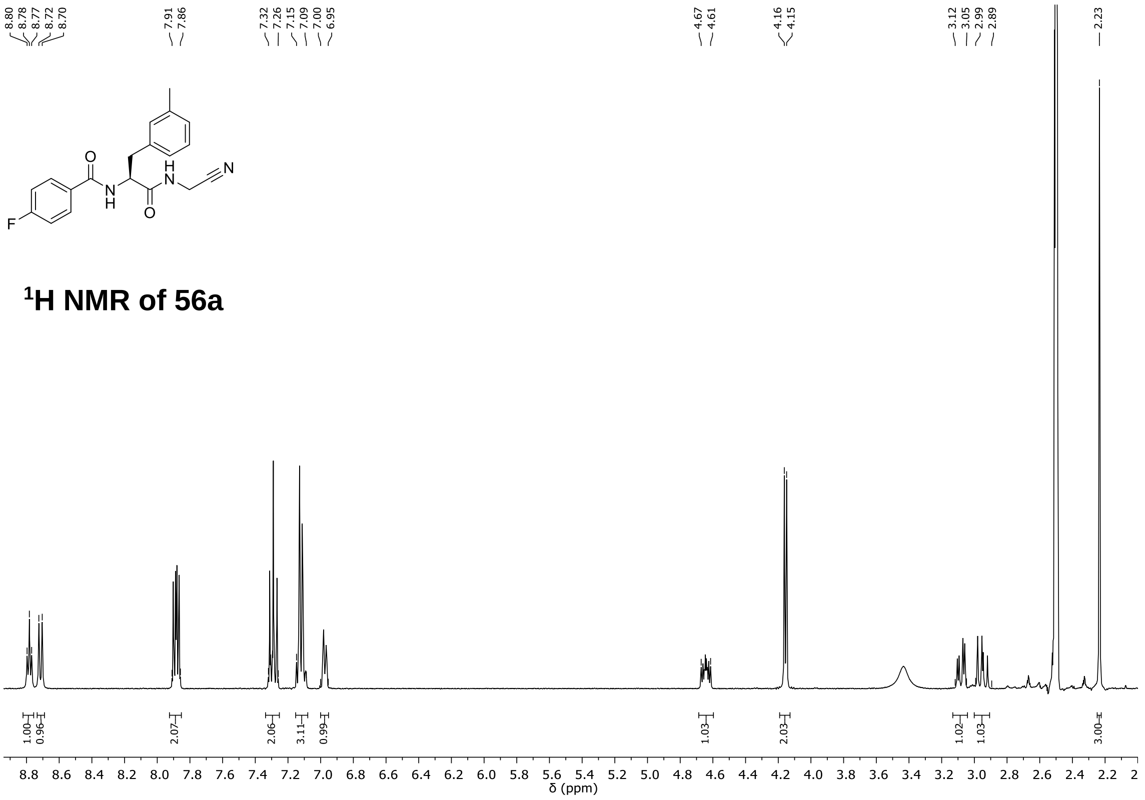

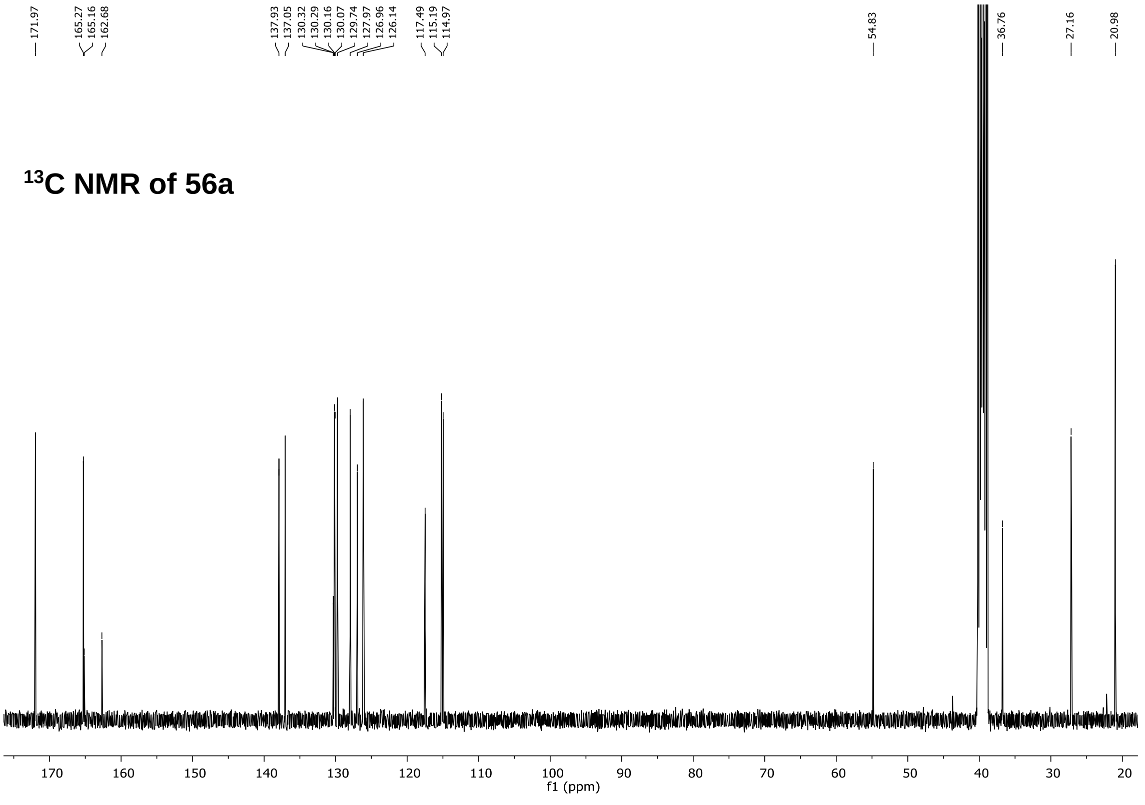

N-Diphenylacetyl-3-methyl-L-phenylalanylglycine nitrile (56b)

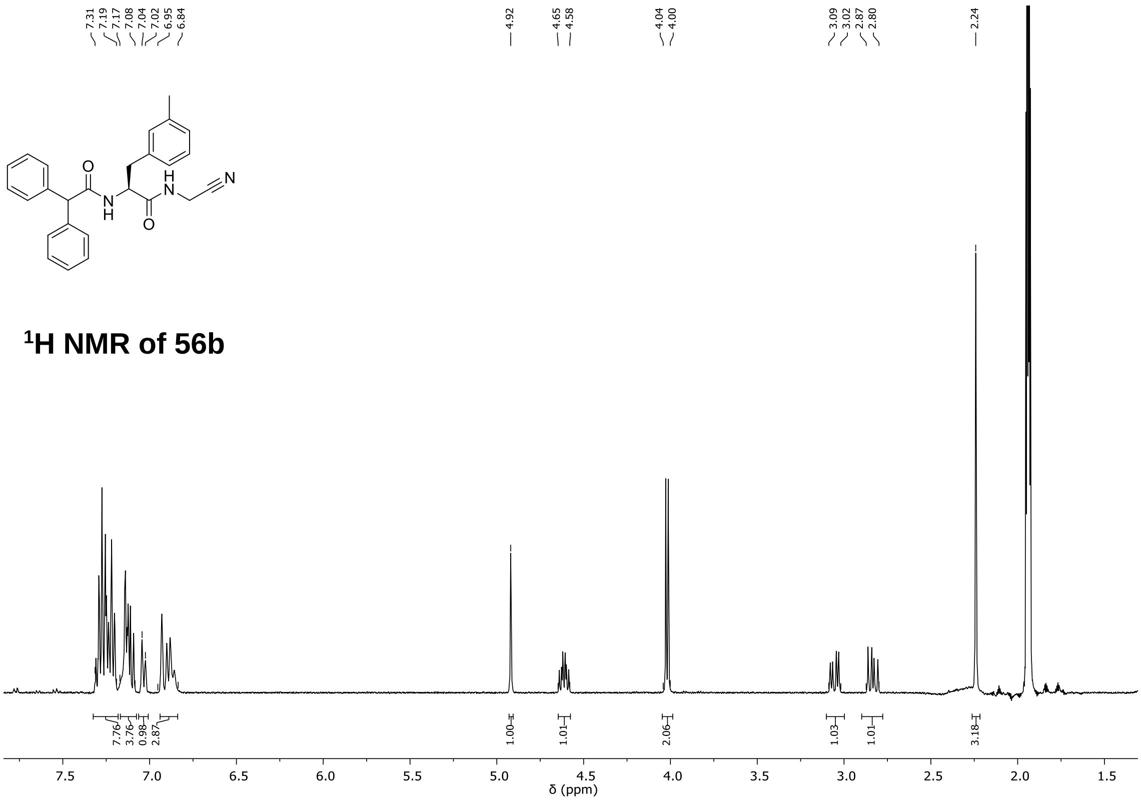

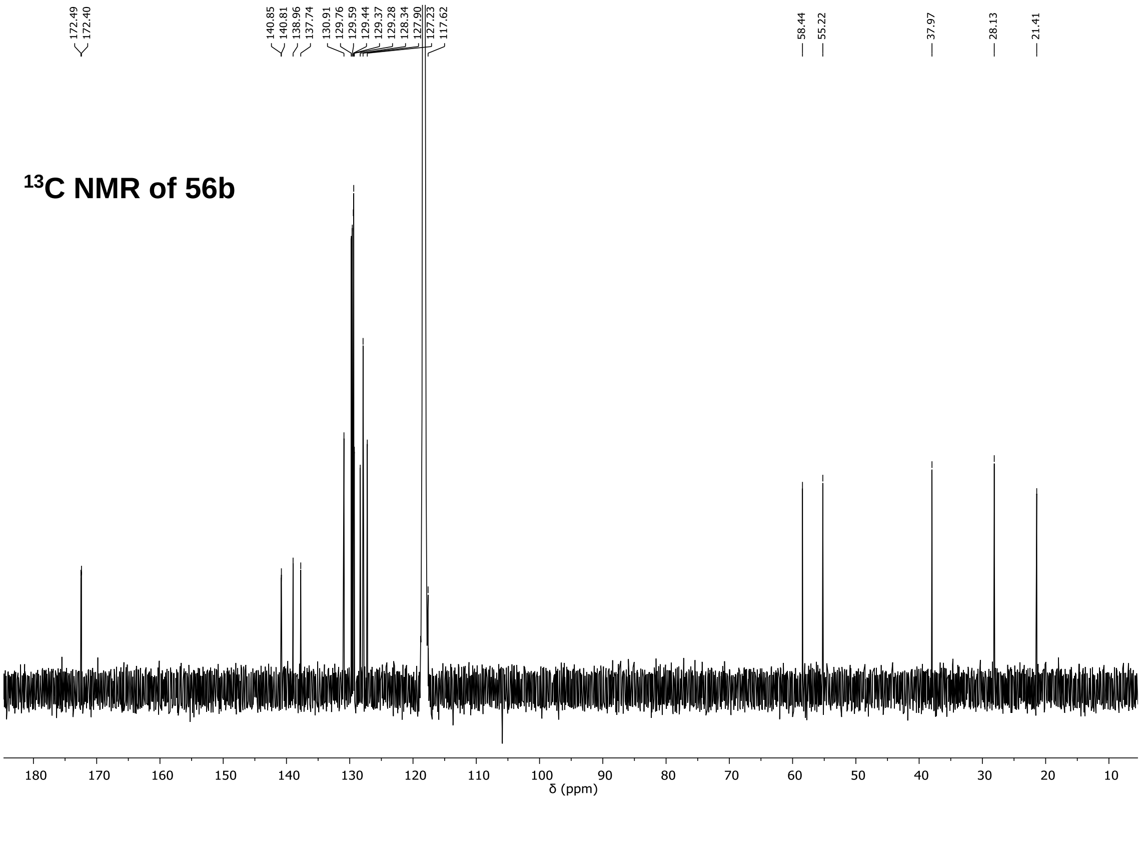

N-(4-Fluorobenzoyl)-3-methyl-L-phenylalanylglycine alkyne (56c)

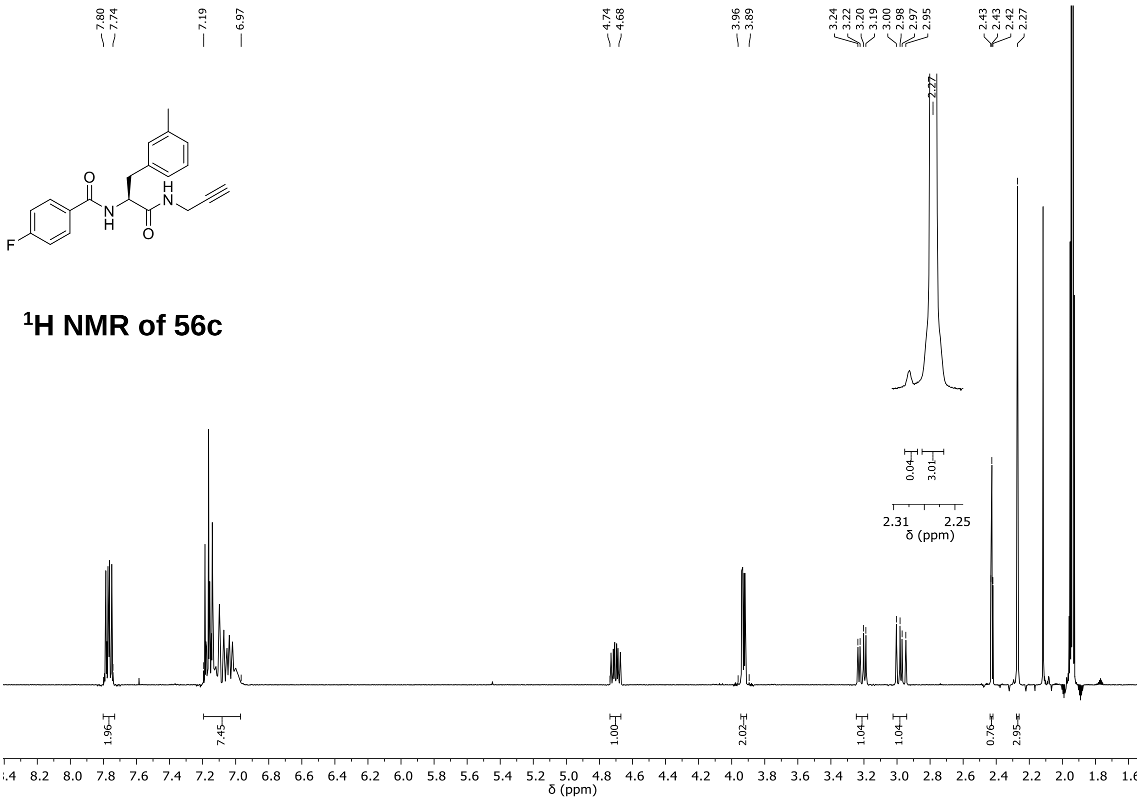

^{1}H NMR of 56c

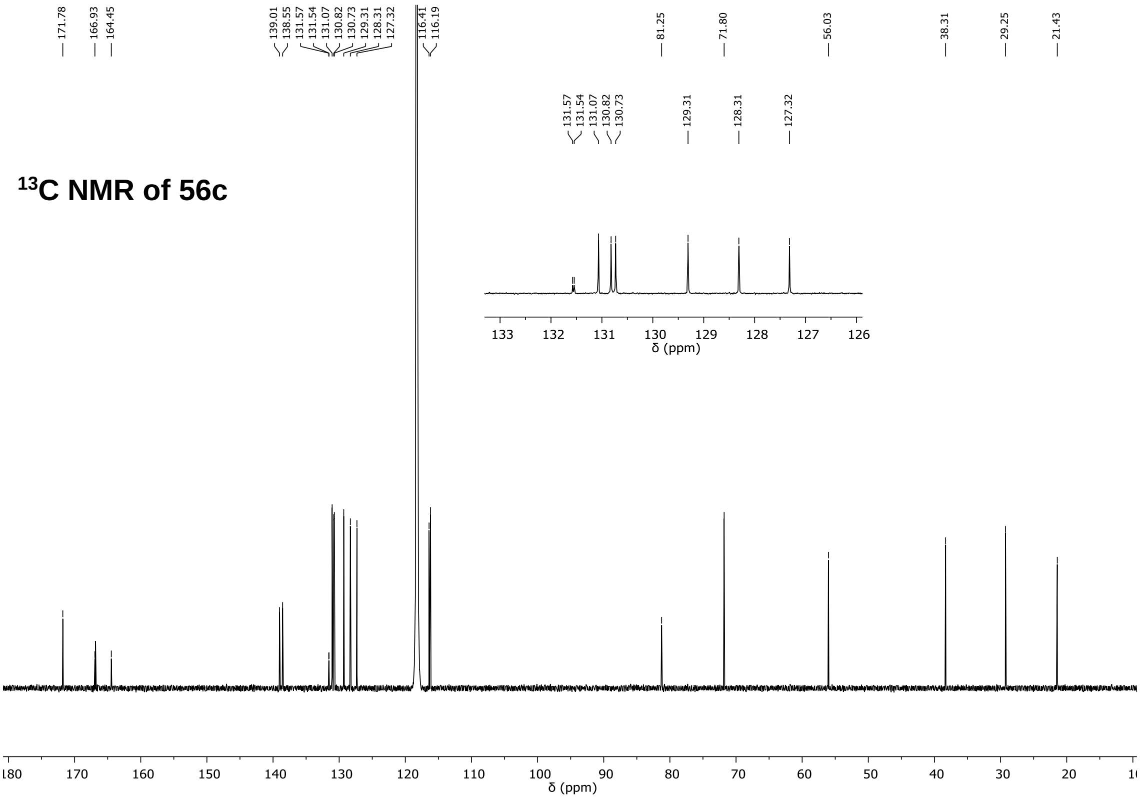

^{13}C NMR of 56c

N-Diphenylacetyl-3-methyl-L-phenylalanylglycine alkyne (56d)

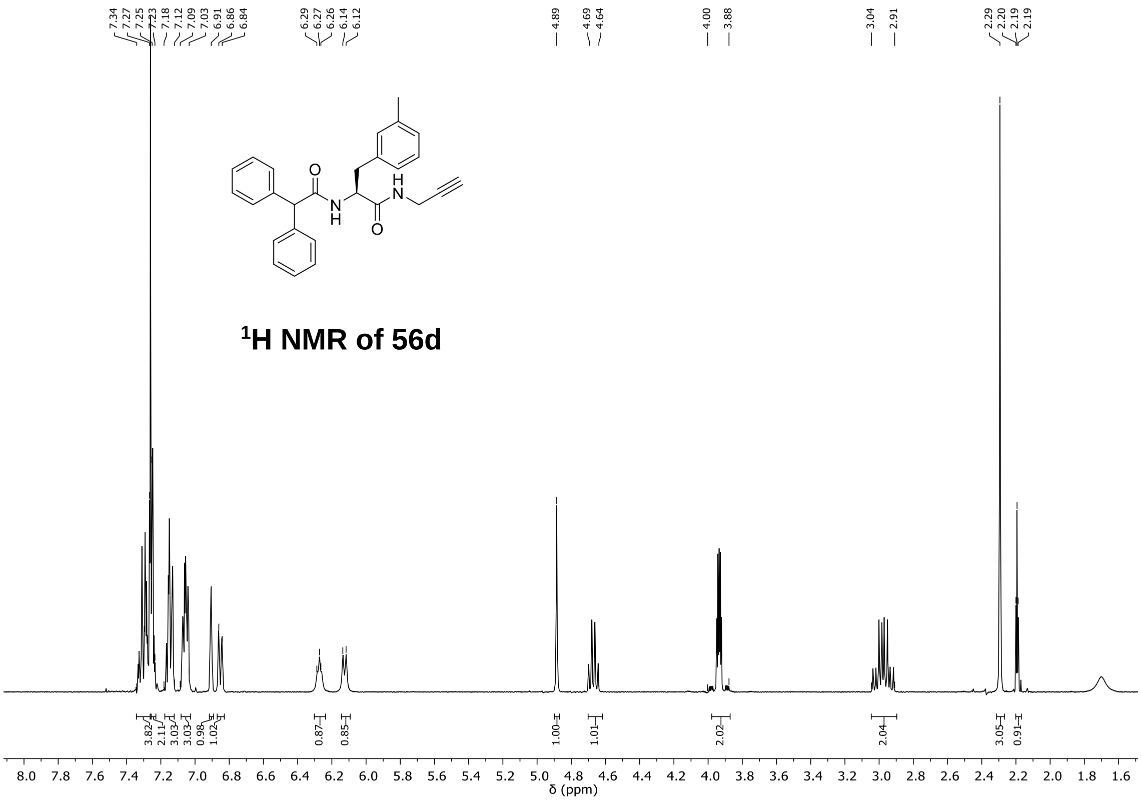

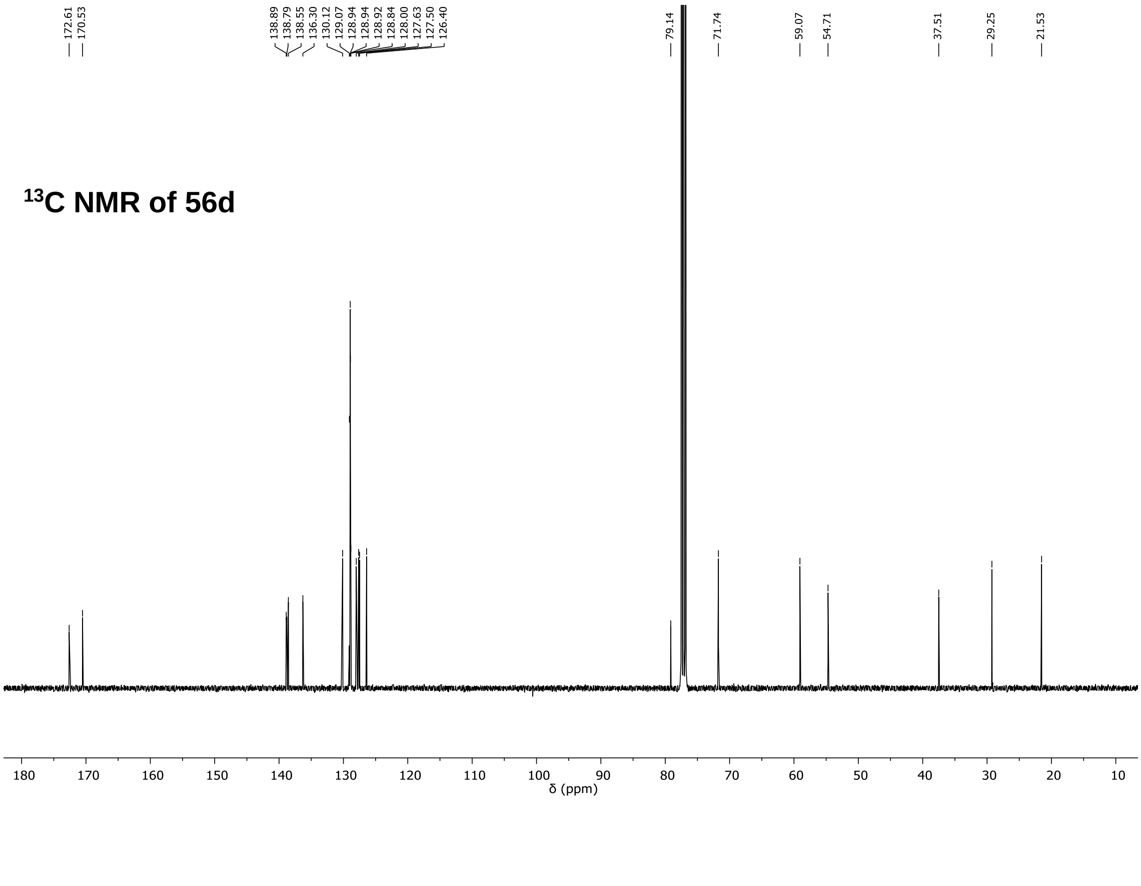

4-Iodobenzoyl-L-leucylglycine alkyne (56e)

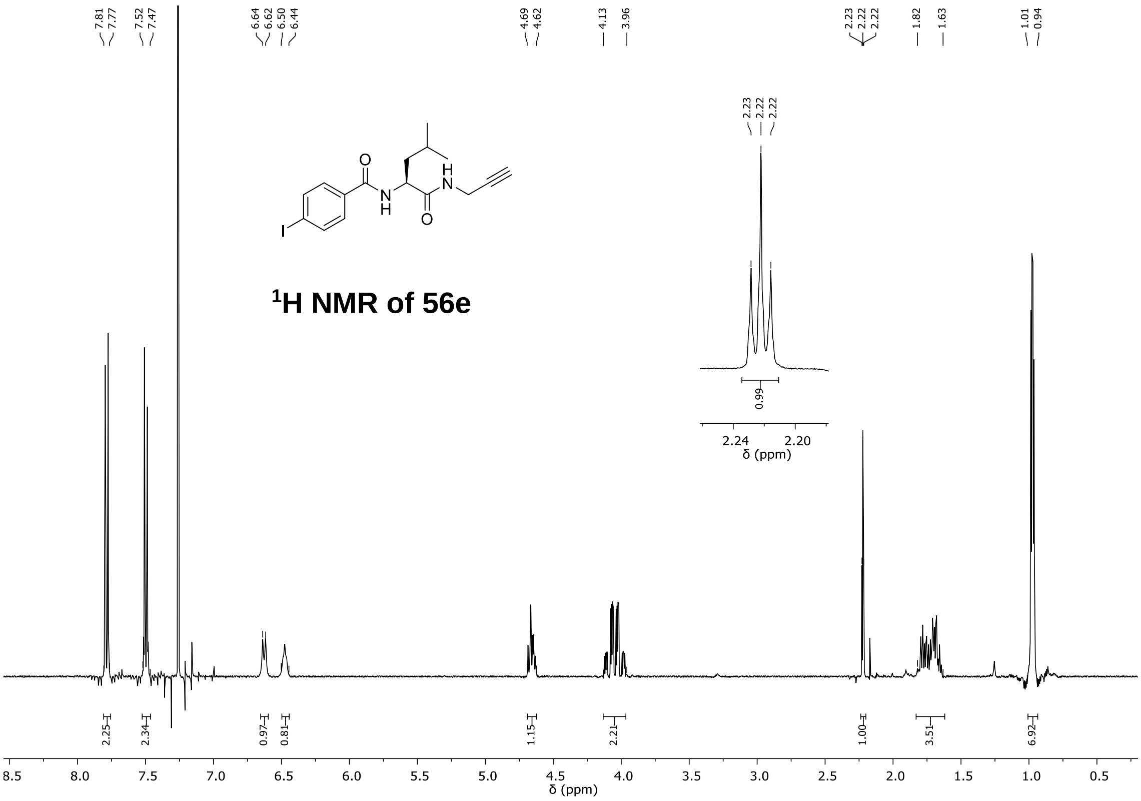

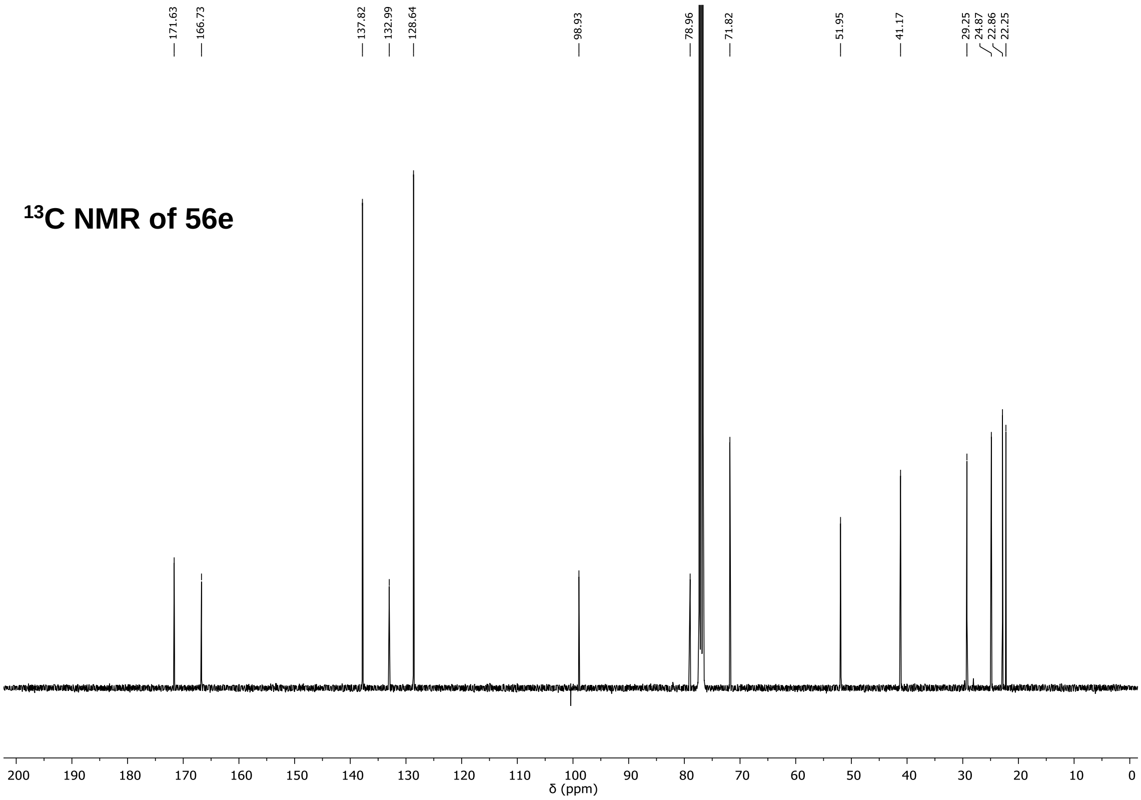

4-Fluorobenzoyl-3-cyclohexyl-L-alanylglycine alkyne (56f)

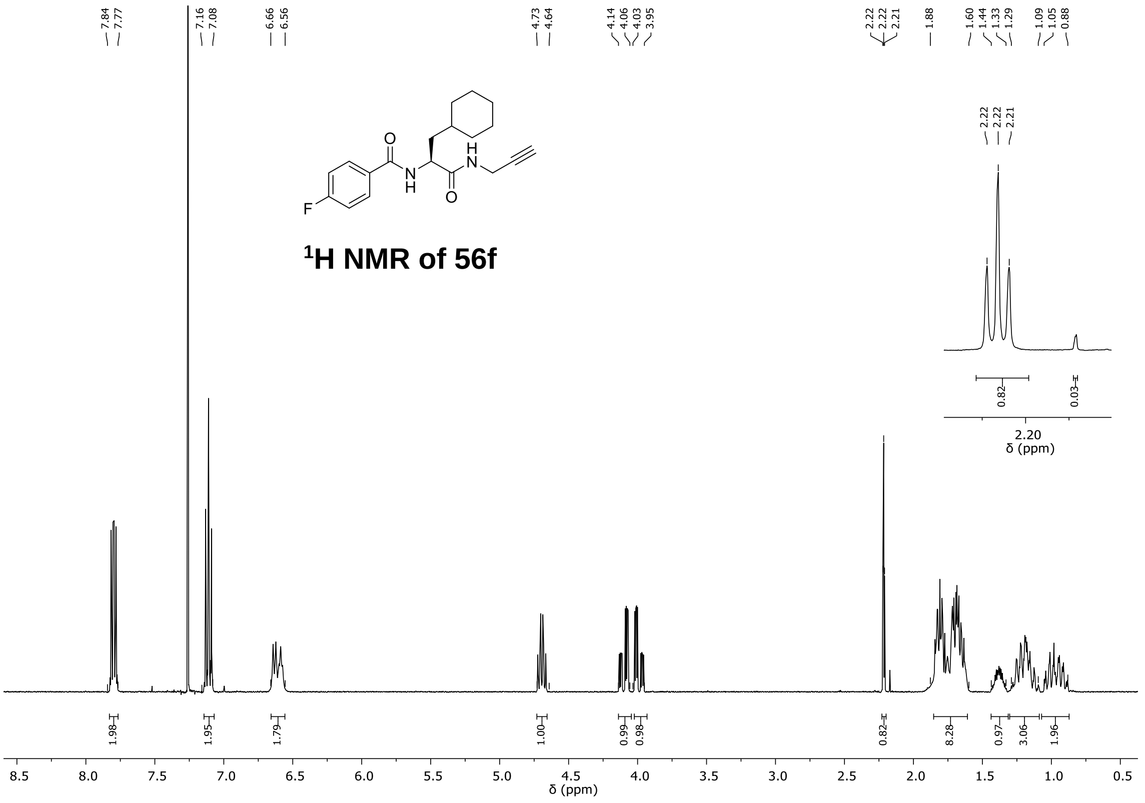

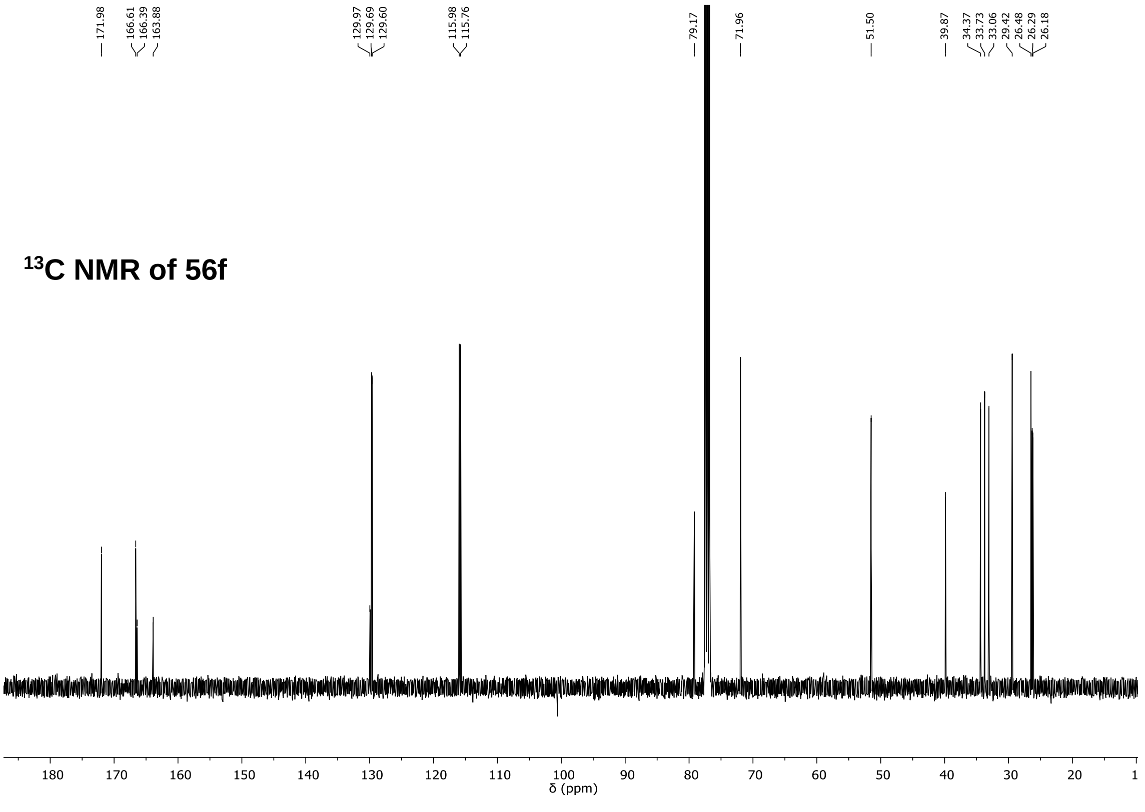

8 4-Fluorobenzoyl-3-(isobutylsulfonyl)-L-alanyl-glycine alkyne (62)

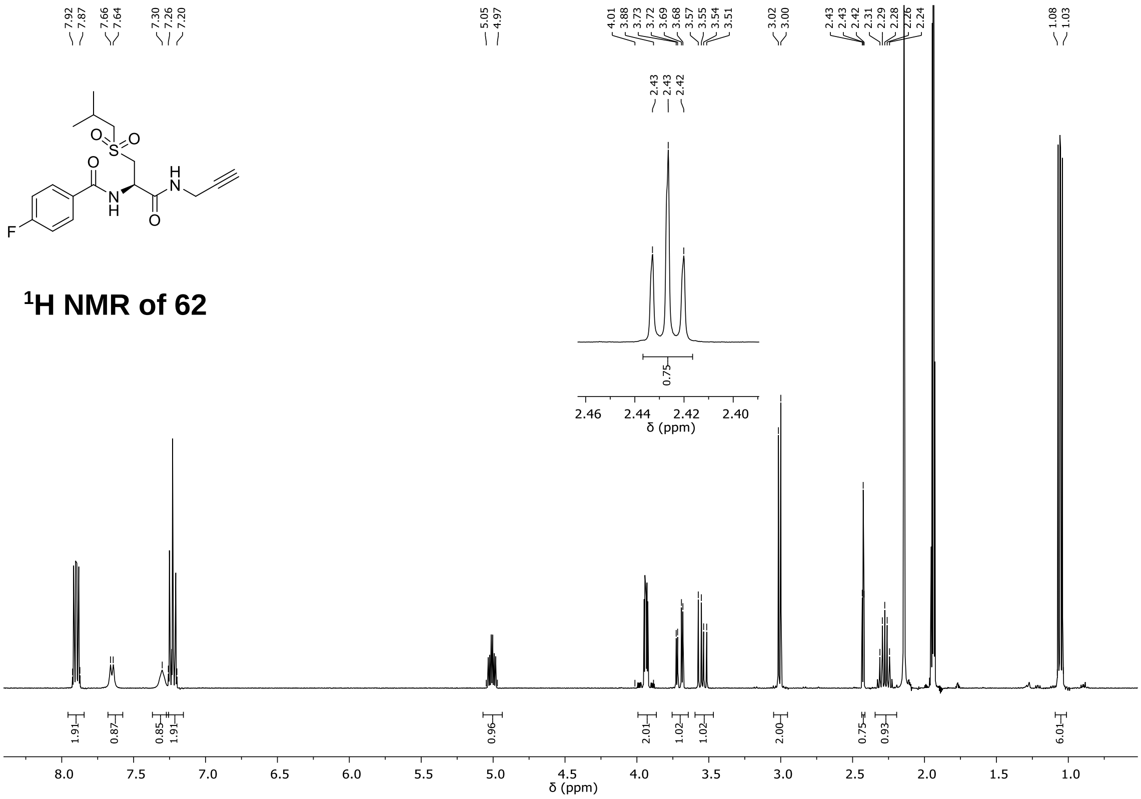

^{1}H NMR of 62

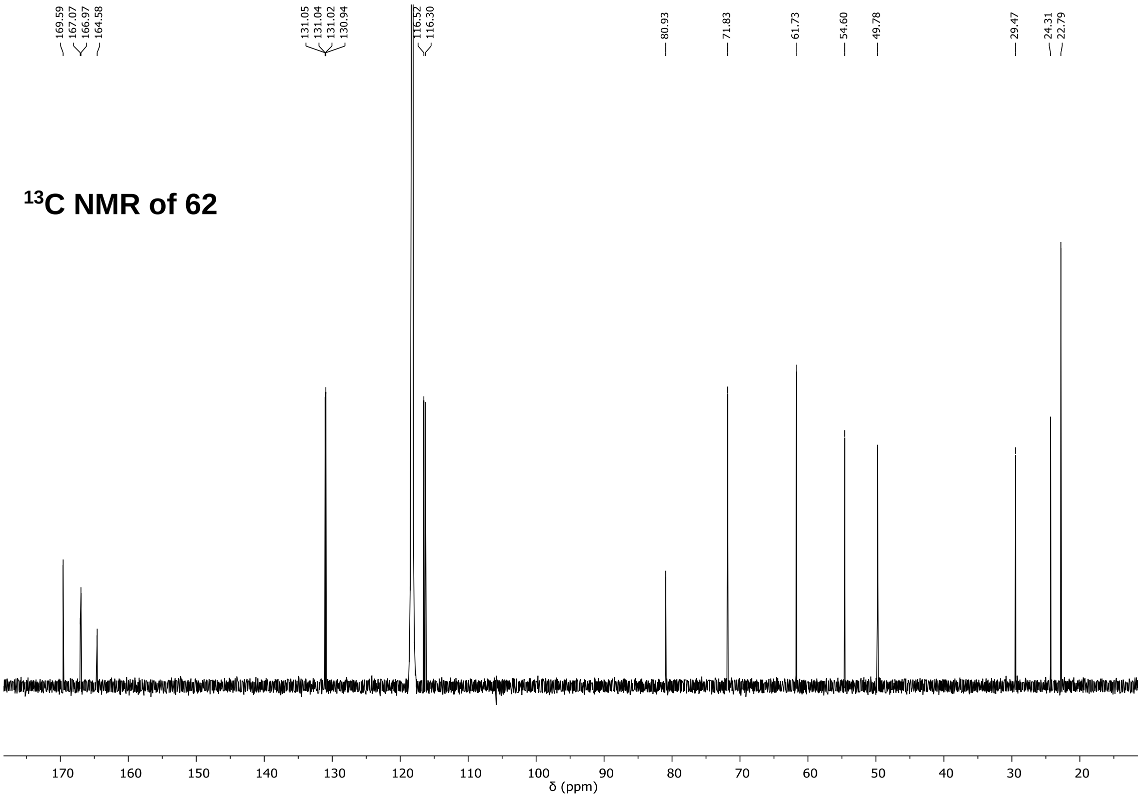

^{13}C NMR of 62

9 Dipeptide alkynes 53k, 63 and 64 with esterified carboxylic group

N-(4-Fluorobenzoyl)-3-iodo-L-phenylalanyl-*O*-(3-(allyloxycarbonyl)benzyl)-L-serine alkyne (53k)

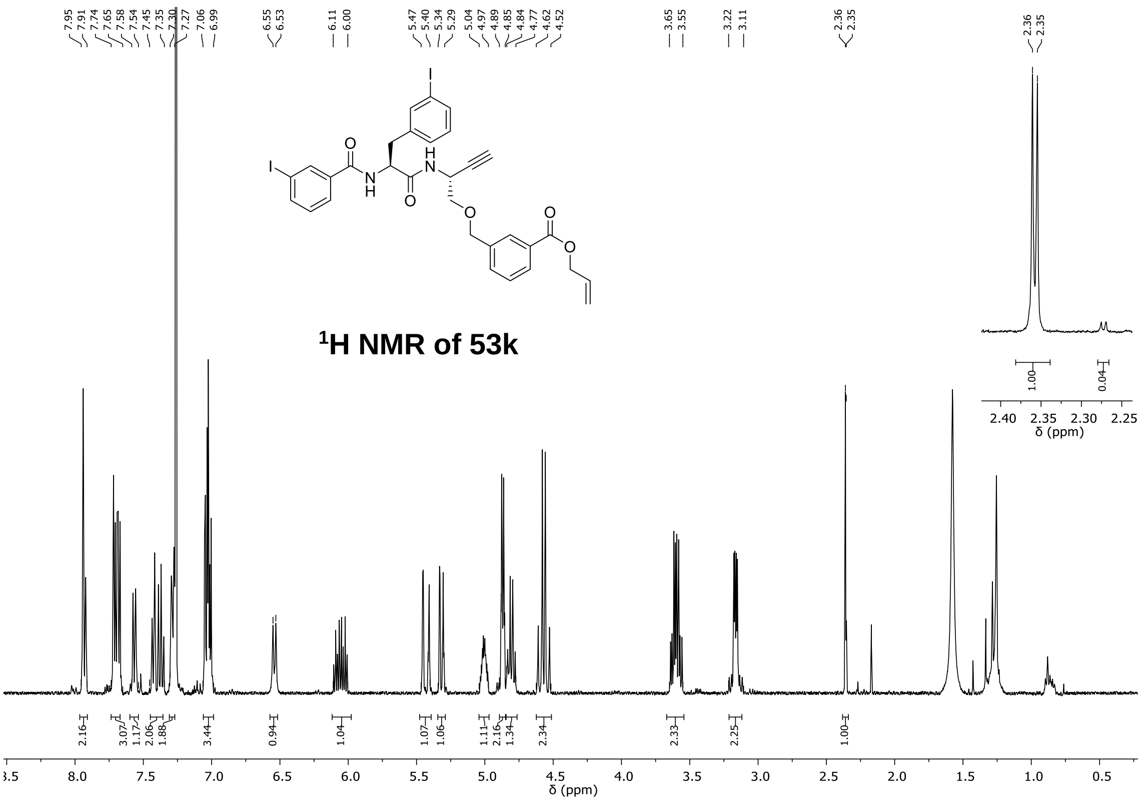

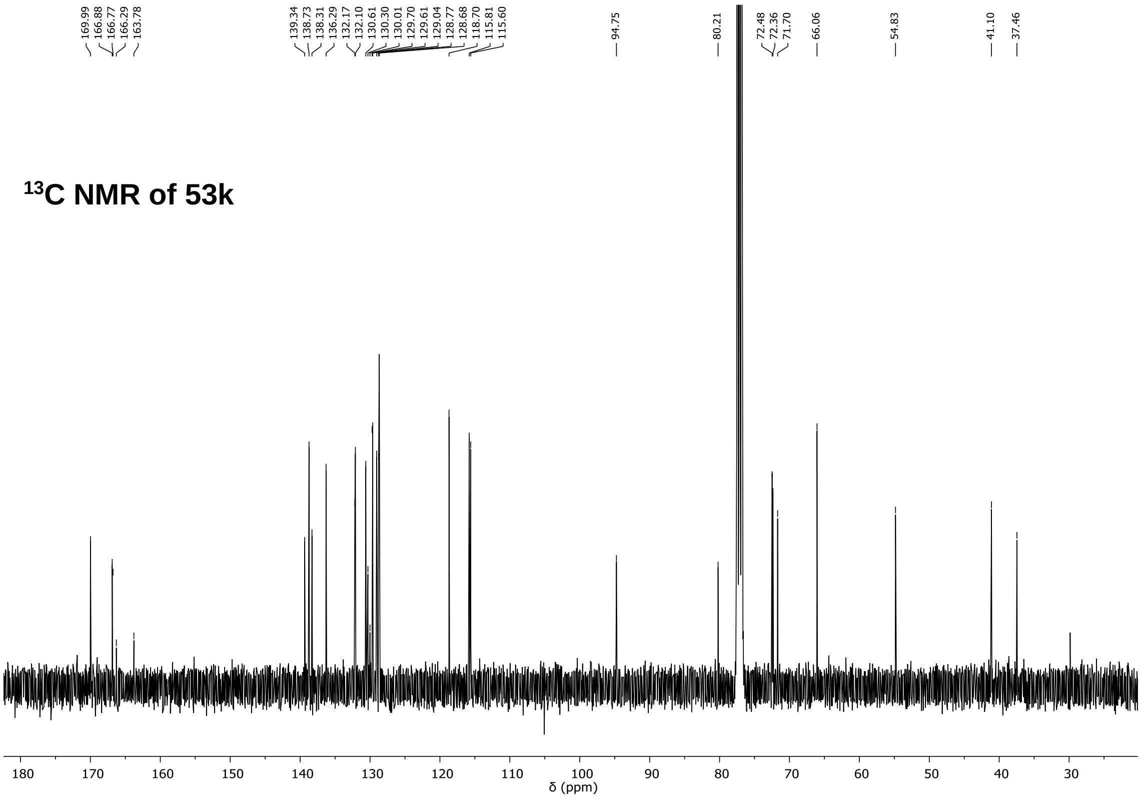

N-(4-Fluorobenzoyl)-3-methyl-L-phenylalanyl-O-(3-(methoxycarbonyl)benzyl)-L-serine alkyne (63)

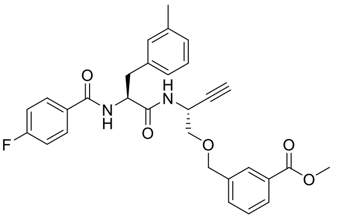

^{1}H NMR of 63

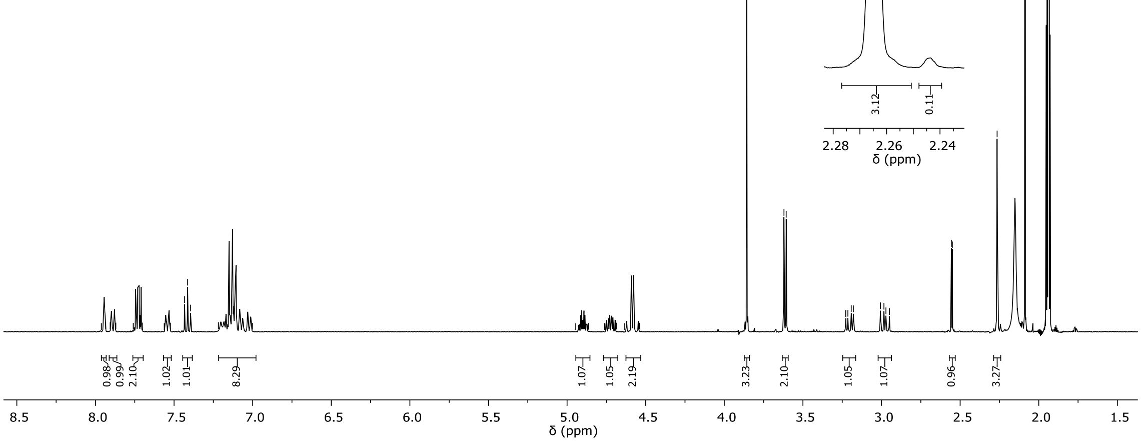

^{13}C NMR of 63

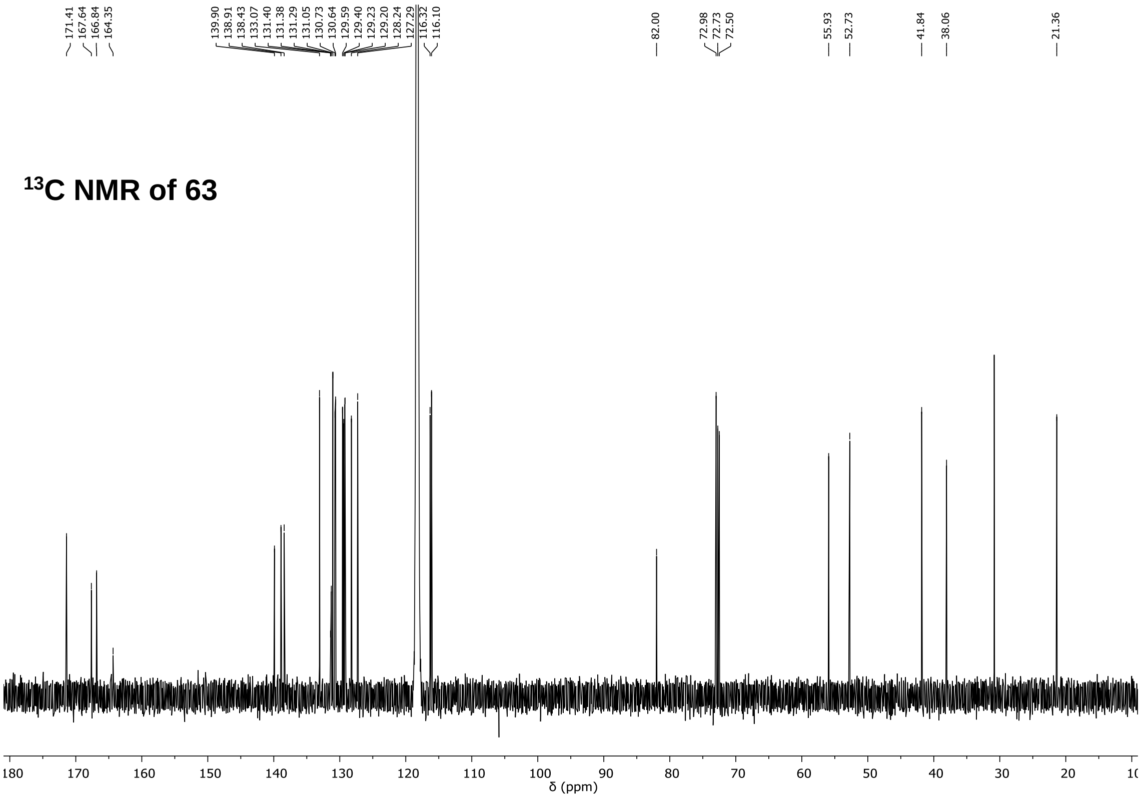

N-(3-Iodobenzoyl)-3-methyl-L-phenylalanyl-O-(3-(methoxycarbonyl)benzyl)-L-serine alkyne (64)

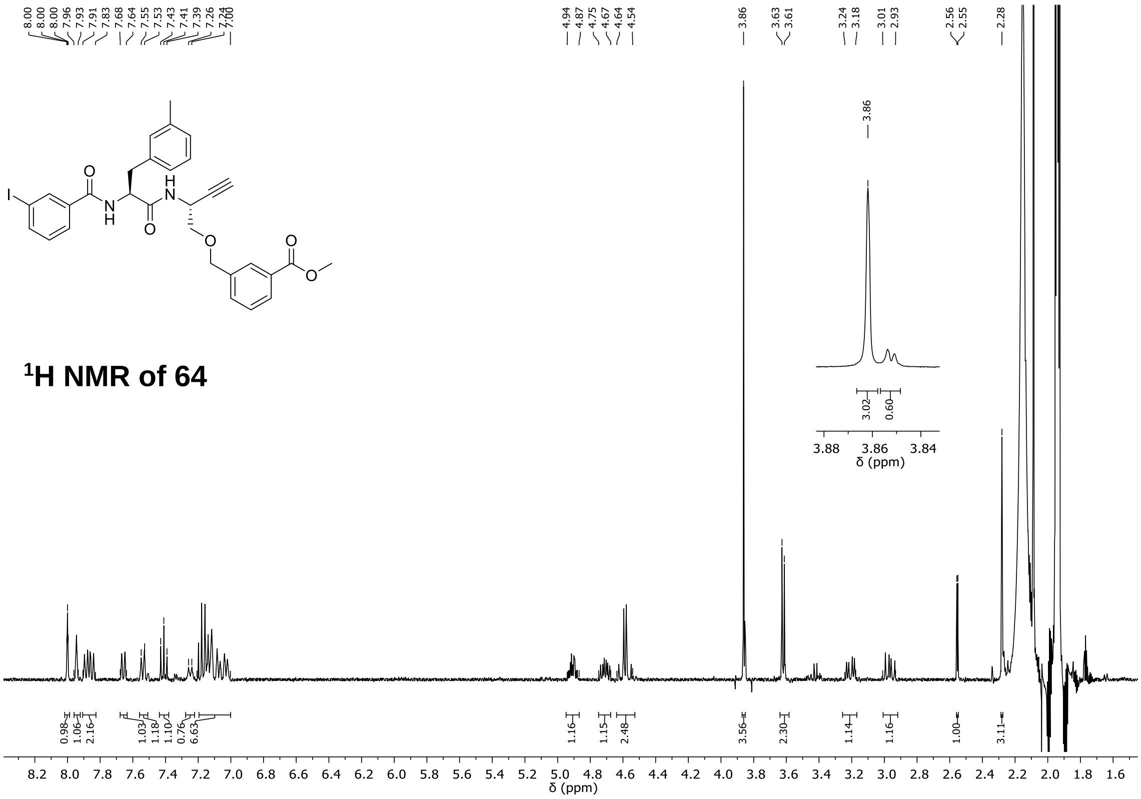

^{1}H NMR of 64

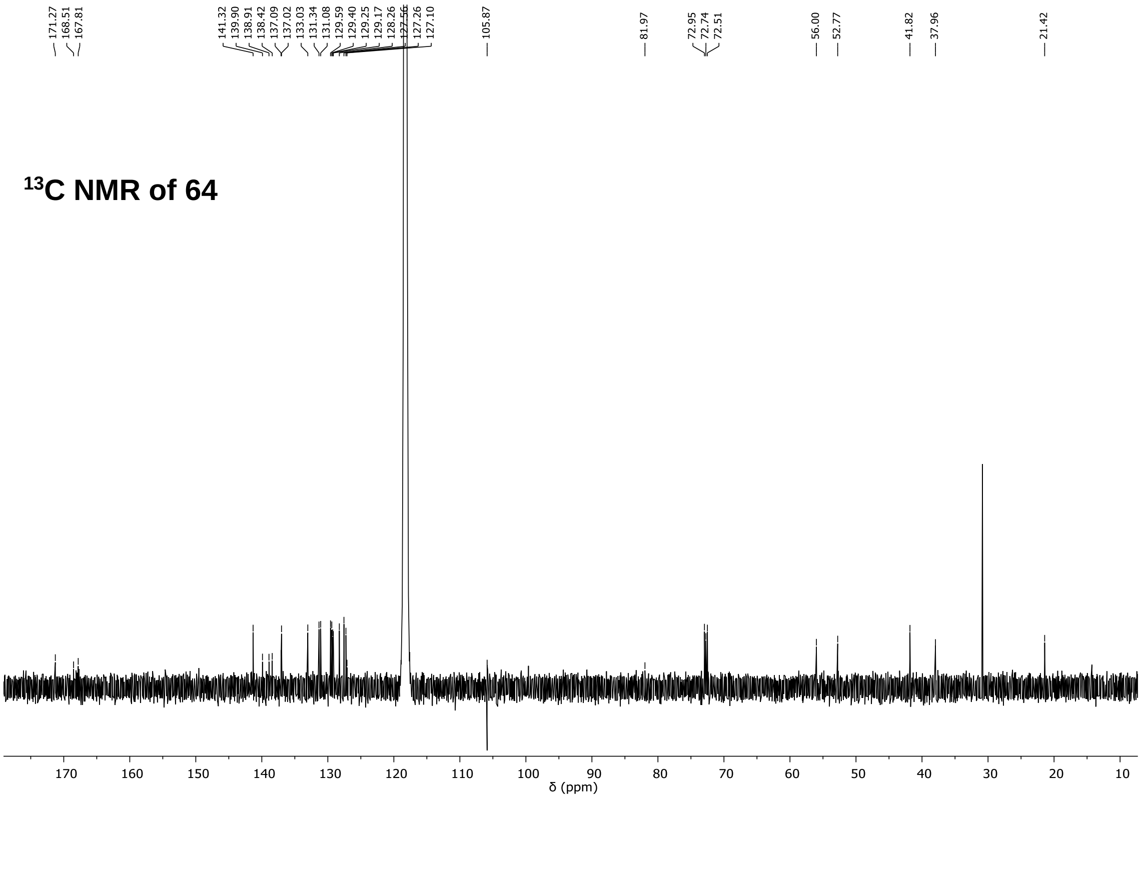

^{13}C NMR of 64

Purities and chromatograms of inhibitor compounds

Table S10: Purities of inhibitor compounds as determined by HPLC

compound	purity (%)*	system
**1a**	96.7	A
**1b**	98.3	A
**1c**	97.7	B
**1d**	96.5	C
**1e**	99.0	B
**2a**	98.4	A
**2b**	96.7	A
**2c**	99.0	B
**2d**	100	A
**2e**	100	A
**2f**	99.1	B
**2g**	98.3	C
**2h**	100	C
**2i**	98.6	B
**2j**	n.d.**	-
**2k**	96.8	C
**2l**	97.7	B
**2m**	98.3	B
**18**	99.7	A
**28**	100	C
**35a**	97.0	C
**35b**	94.9	C
**35c**	99.4	C
**43**	100	C
**56a**	100	C
**56b**	n.d	-
**56c**	99.4	C
**56d**	97.6	A
**56e**	100	A
**56f**	100	A
**62**	100	C
**63**	100	C
**64**	n.d.	-

*Wavelength for detection: 254 nm
**n.d. - not determined

Compound **1a**

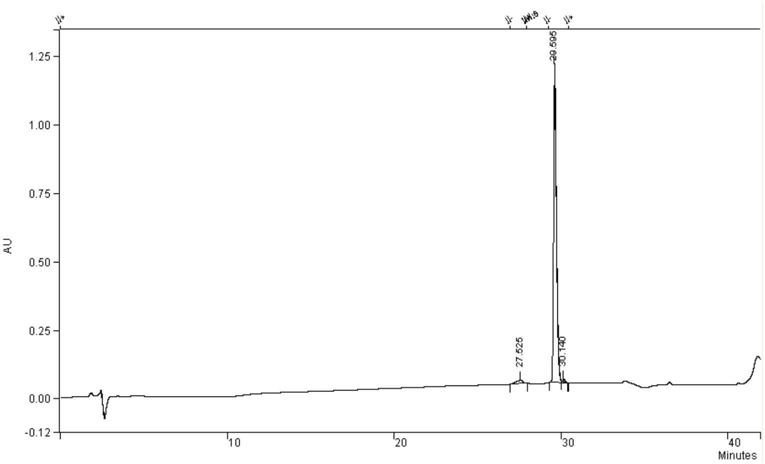

Peak No.	Peak Name	Result ()	Ret. Time (min)	Time Offset (min)	Area (counts)	Sep. Code	Width 1/2 (sec)	Status Codes
1		2.5630	27.525	0.000	3574296	BB	27.2	
2		96.6980	29.595	0.000	134854752	BB	10.3	
3		0.7390	30.140	0.000	1030666	TS	0.0	
	Totals:	100.0000		0.000	139459714			

Compound **1b**

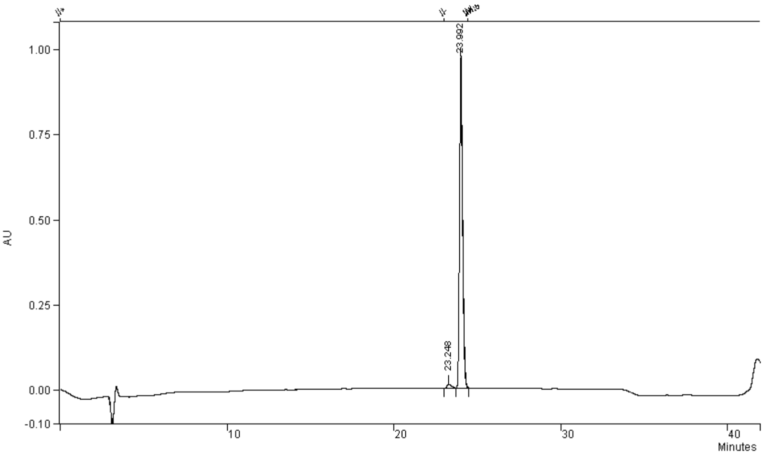

Calculation Type: Percent

Peak No.	Peak Name	Result ()	Ret. Time (min)	Time Offset (min)	Area (counts)	Sep. Code	Width 1/2 (sec)	Status Codes
1		1.6739	23.248	0.000	2052819	BV	15.5	
2		98.3261	23.992	0.000	120587296	VB	11.3	
	Totals:	100.0000		0.000	122640115			

Compound **1c**

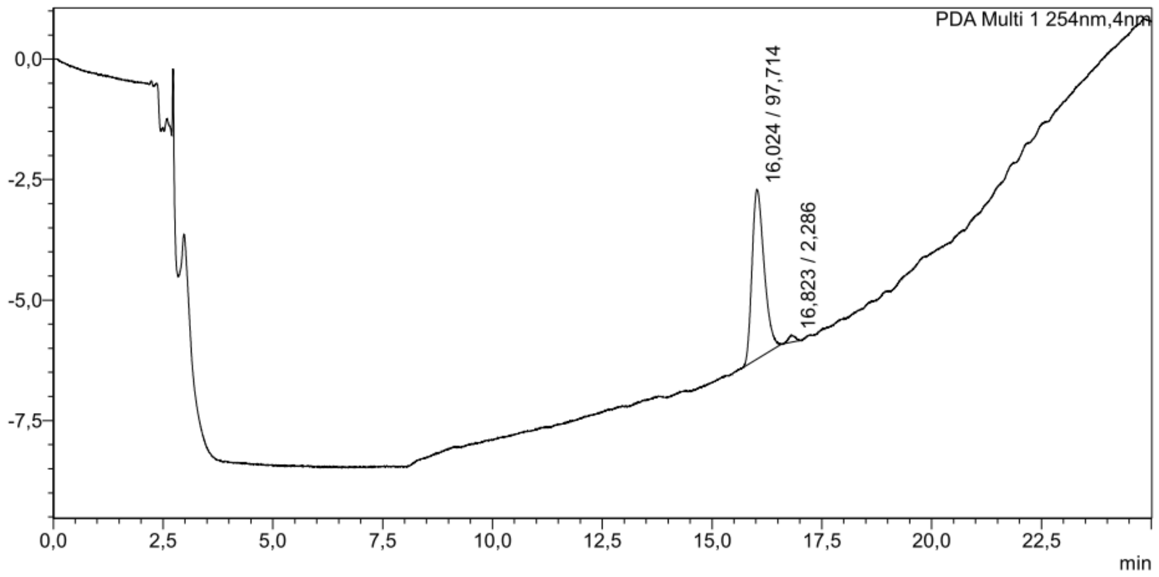

PDA Ch1 254nm						
Peak#	Ret. Time	Area	Height	Conc.	Unit	Area%
1	16,024	67541	3523	0,000		97,714
2	16,823	1580	139	0,000		2,286
Total		69121	3663			100,000

Compound **1d**

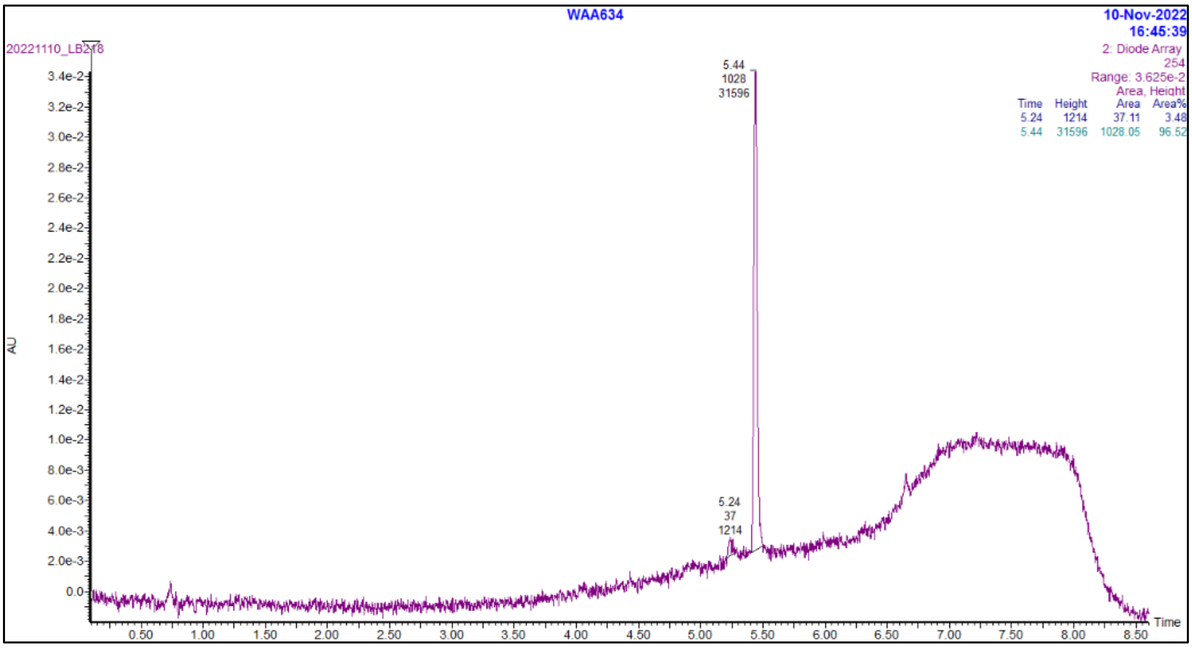

Compound **1e**

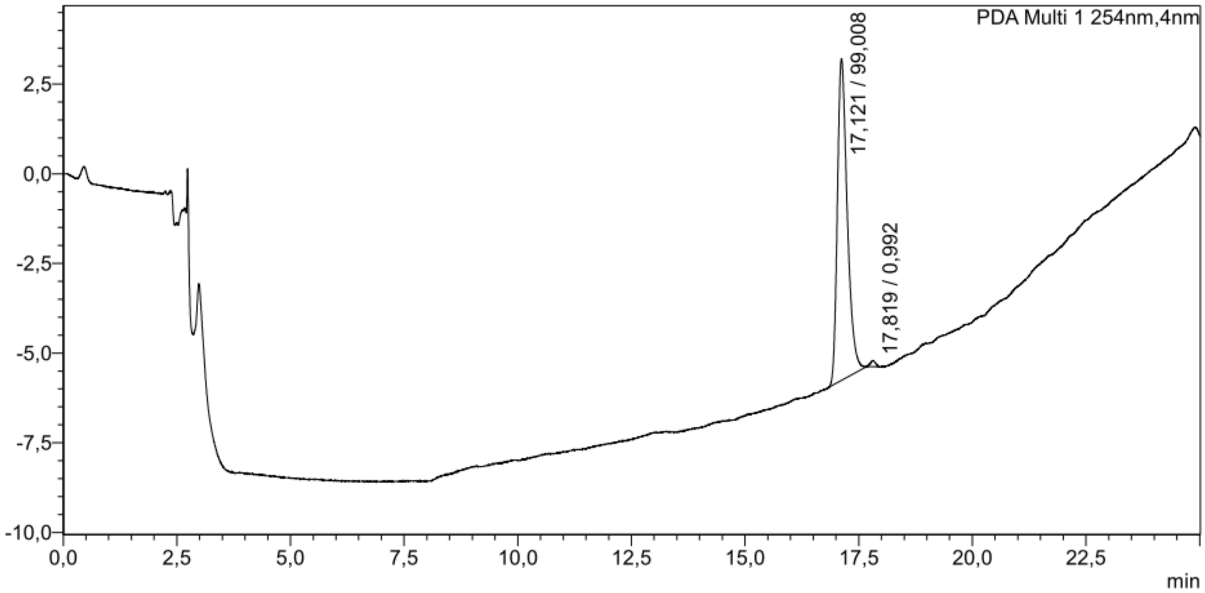

PDA Ch1 254nm						
Peak#	Ret. Time	Area	Height	Conc.	Unit	Area%
1	17,121	135696	8958	0,000		99,008
2	17,819	1359	169	0,000		0,992
Total		137055	9127			100,000

Compound **2a**

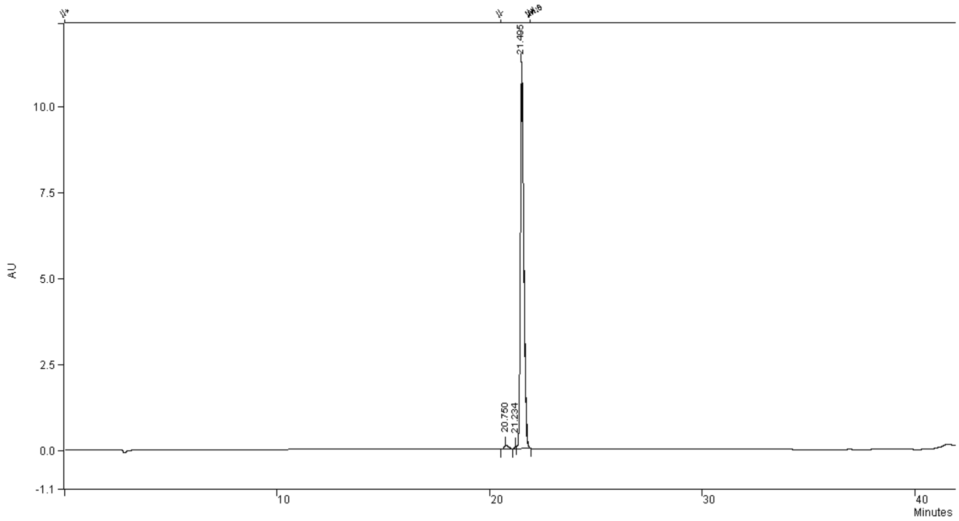

Peak No.	Peak Name	Result ()	Ret. Time (min)	Time Offset (min)	Area (counts)	Sep. Code	Width 1/2 (sec)	Status Codes
1		1.2371	20.750	0.000	15382619	BP	13.5	
2		0.3378	21.234	0.000	4200355	PV	9.2	
3		98.4251	21.495	0.000	1223844352	VB	9.9	
	Totals:	100.0000		0.000	1243427326			

Compound **2b**

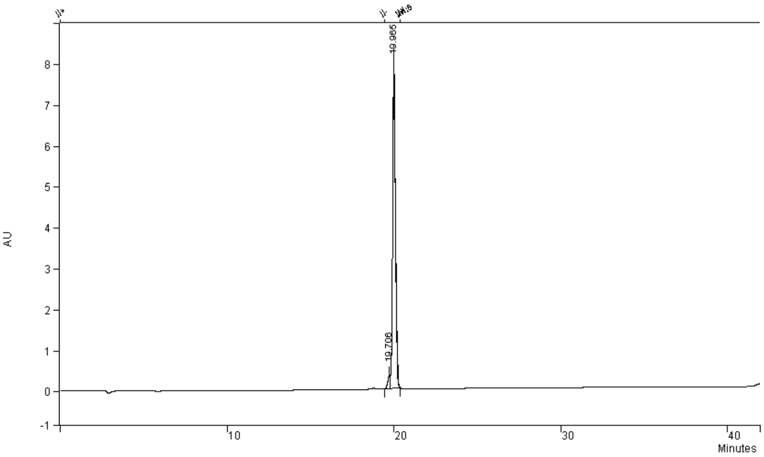

Peak No.	Peak Name	Result ()	Ret. Time (min)	Time Offset (min)	Area (counts)	Sep. Code	Width 1/2 (sec)	Status Codes
1		3.2999	19.706	0.000	31458052	BV	13.7	
2		96.7001	19.965	0.000	921858112	VB	10.3	
	Totals:	100.0000		0.000	953316164			

Compound **2c**

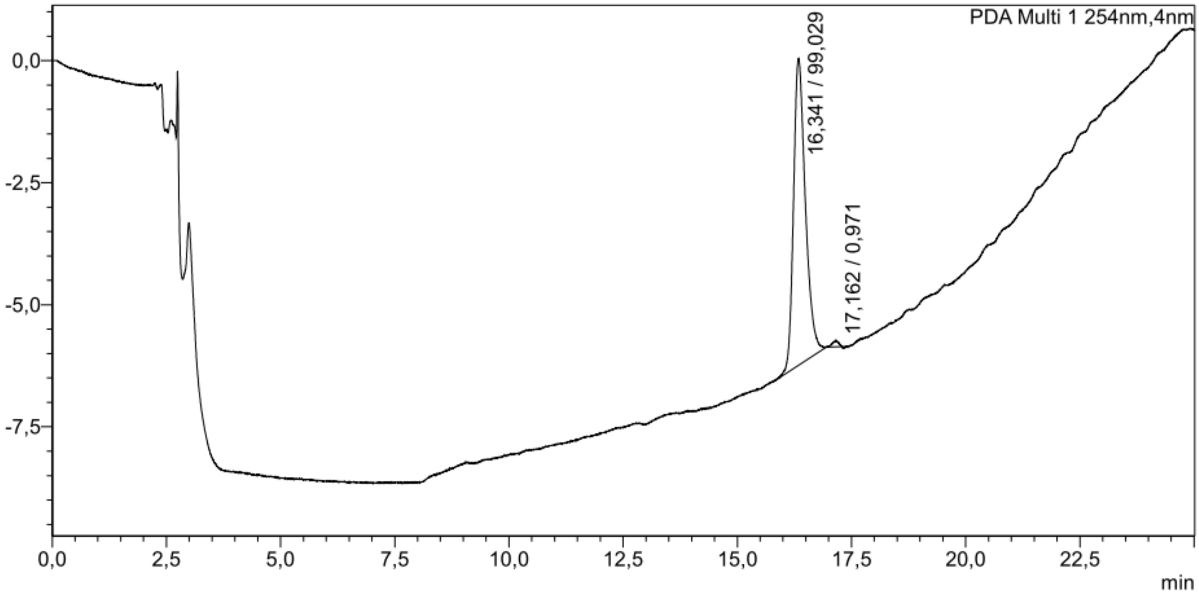

PDA Ch1 254nm

Peak#	Ret. Time	Area	Height	Conc.	Unit	Area%
1	16,341	113685	6284	0,000		99,029
2	17,162	1115	126	0,000		0,971
Total		114800	6410			100,000

Compound **2d**

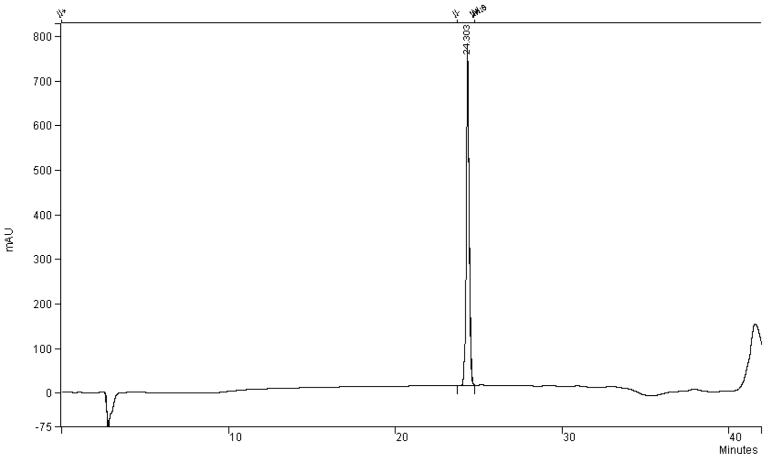

Peak No.	Peak Name	Result ()	Ret. Time (min)	Time Offset (min)	Area (counts)	Sep. Code	Width 1/2 (sec)	Status Codes
1		100.0000	24.303	0.000	86998960	BB	10.4	
	Totals:	100.0000		0.000	86998960			

Compound **2e**

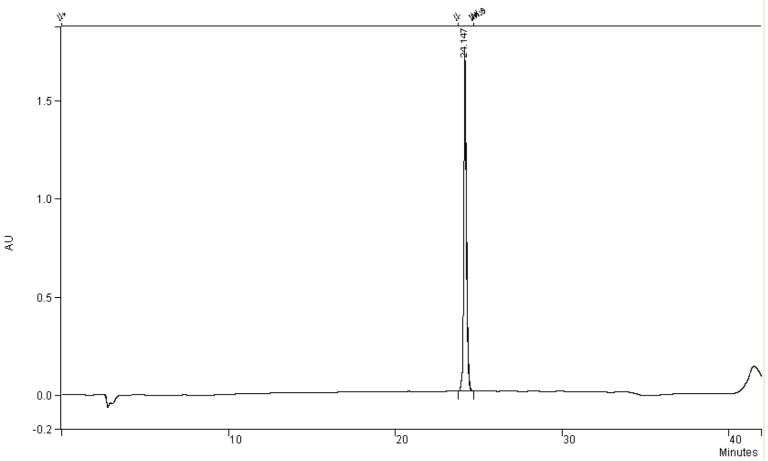

Peak No.	Peak Name	Result ()	Ret. Time (min)	Time Offset (min)	Area (counts)	Sep. Code	Width 1/2 (sec)	Status Codes
1		1.2371	20.750	0.000	15382619	BP	13.5	
2		0.3378	21.234	0.000	4200355	PV	9.2	
3		98.4251	21.495	0.000	1223844352	VB	9.9	
	Totals:	100.0000		0.000	1243427326			

Compound **2f**

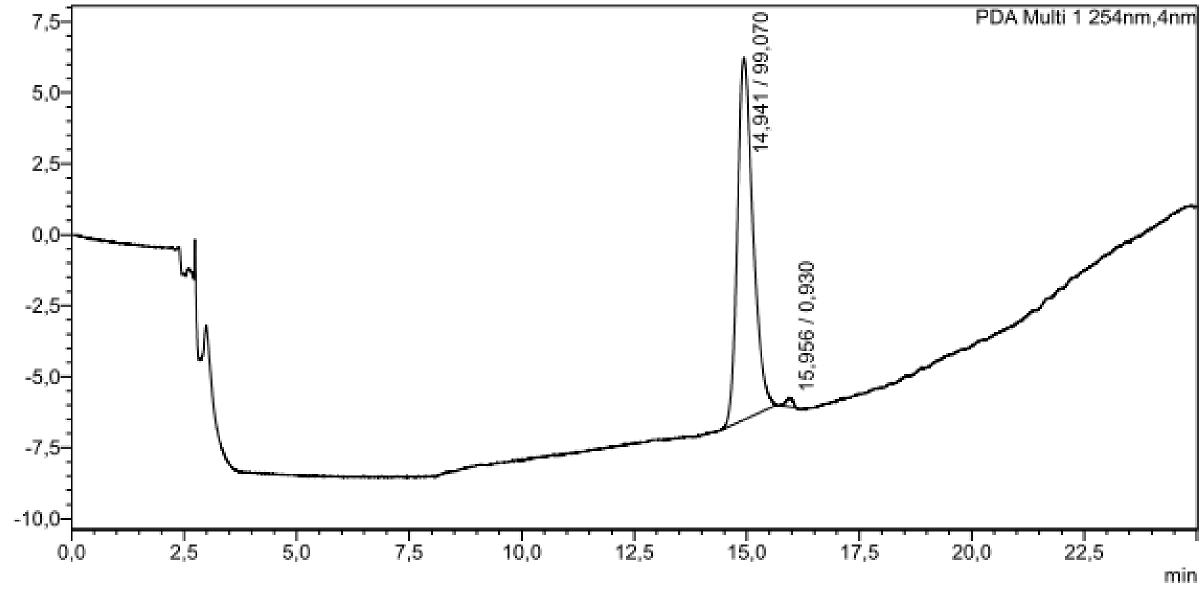

PDA Ch1 254nm						
Peak#	Ret. Time	Area	Height	Conc.	Unit	Area%
1	14,941	306473	12719	0,000		99,070
2	15,956	2877	309	0,000		0,930
Total		309350	13028			100,000

Compound **2g**

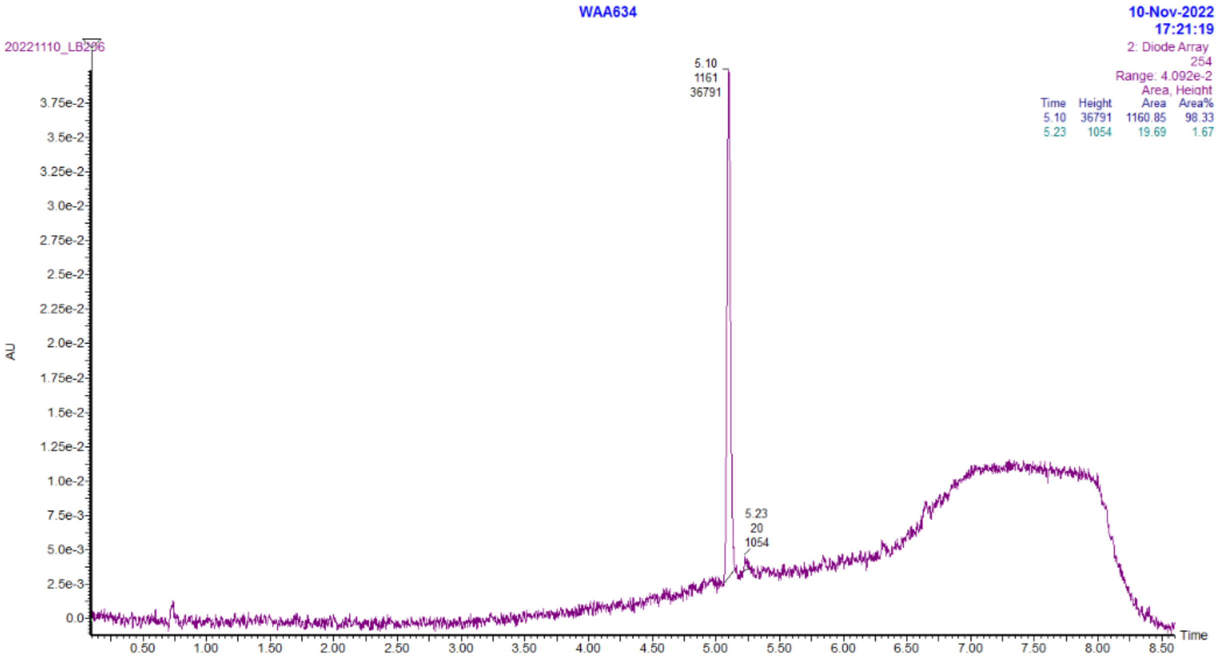

Compound **2h**

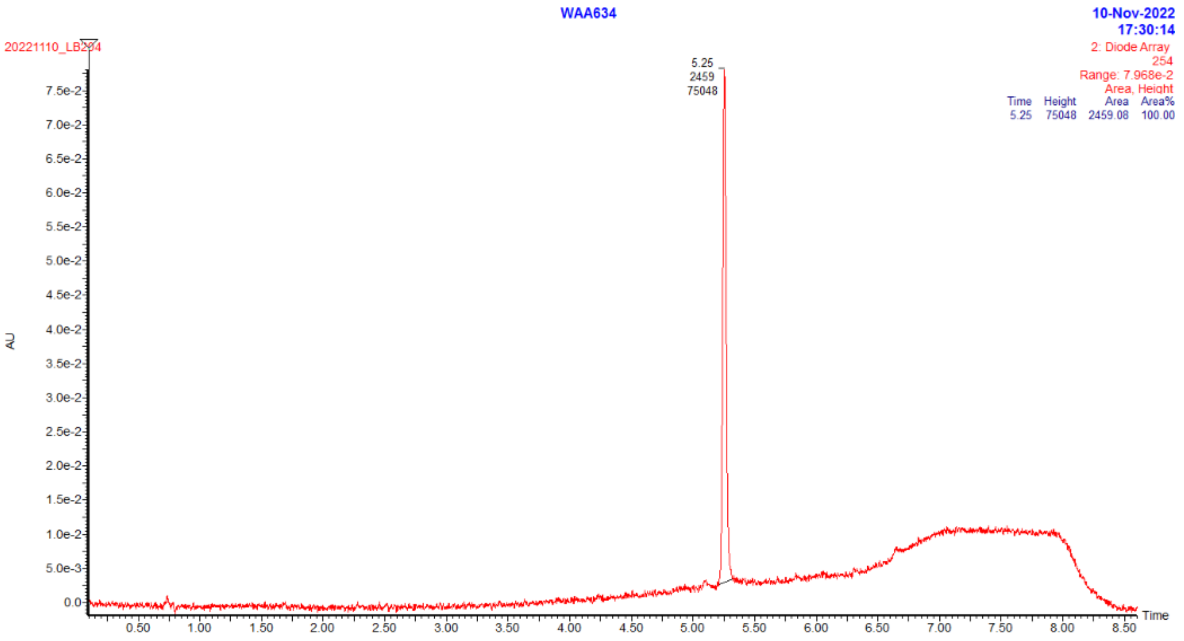

Compound 2i

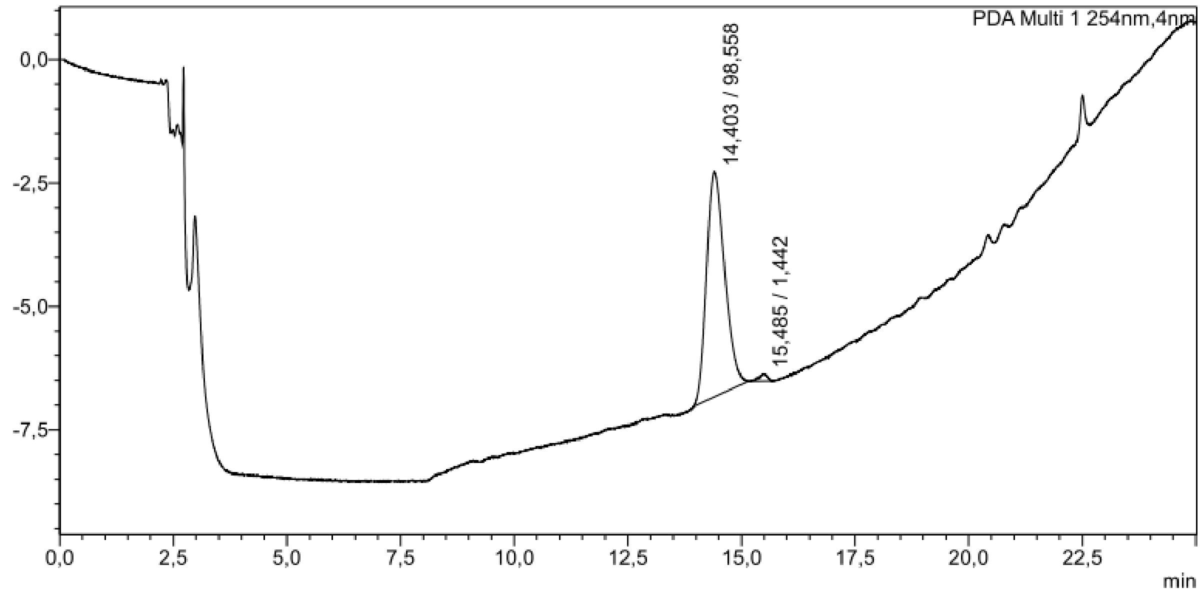

PDA Ch1 254nm						
Peak#	Ret. Time	Area	Height	Conc.	Unit	Area%
1	14,403	126804	4547	0,000		98,558
2	15,485	1855	144	0,000		1,442
Total		128659	4691			100,000

Compound **2k**

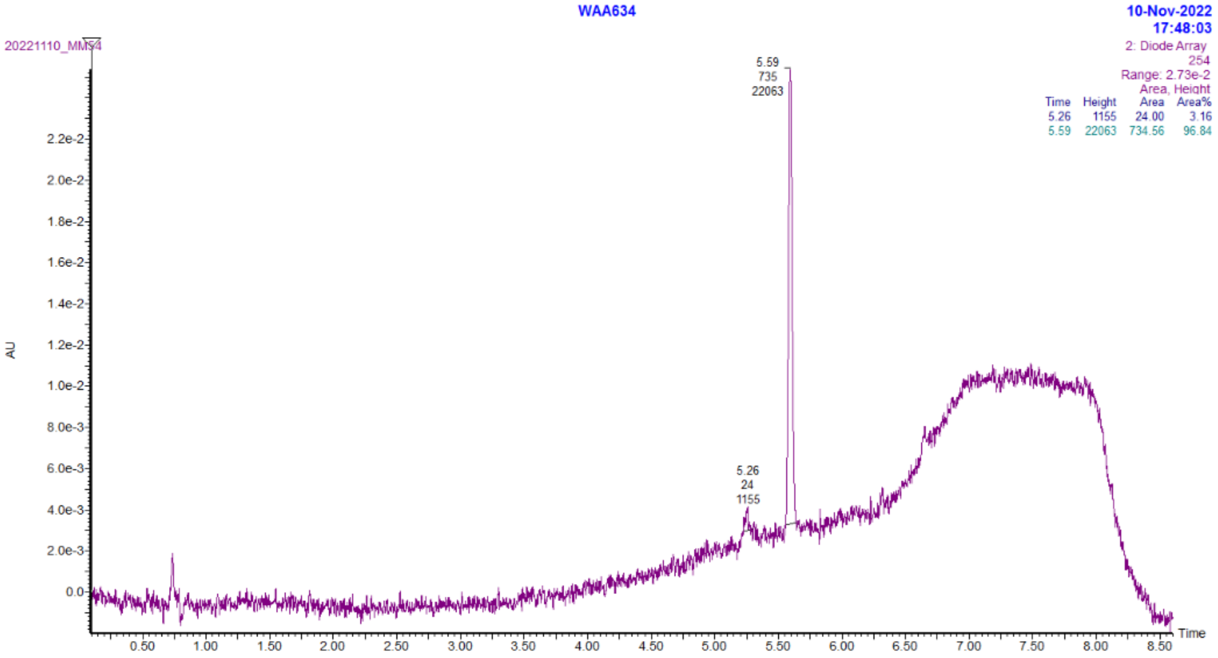

Compound **2l**

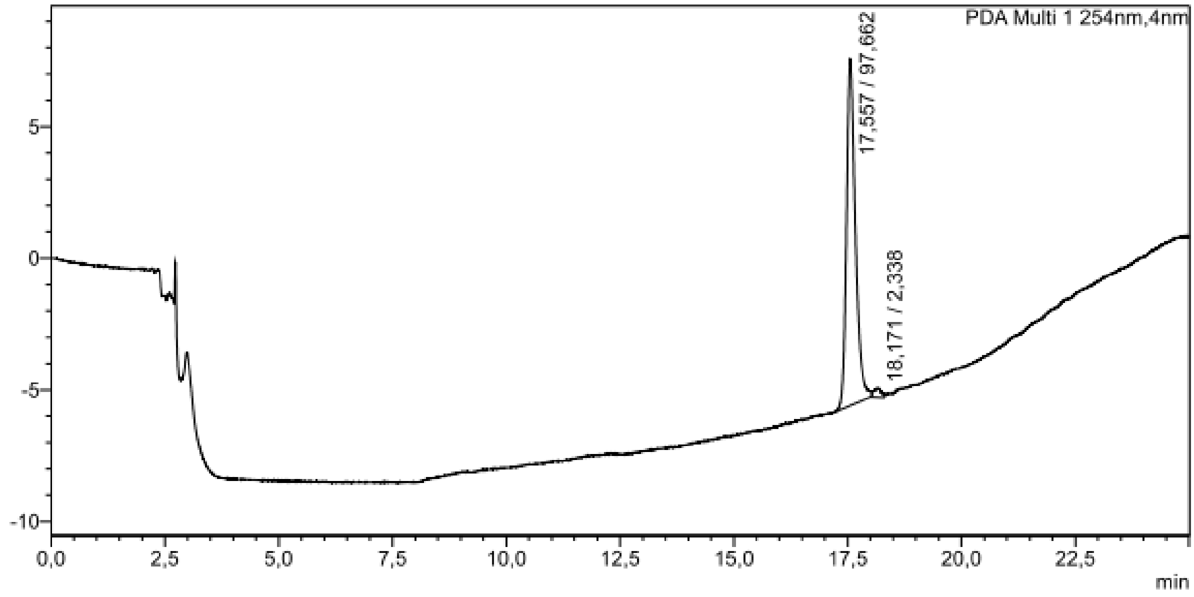

PDA Ch1 254nm

Peak#	Ret. Time	Area	Height	Conc.	Unit	Area%
1	17,557	181508	13163	0,000		97,662
2	18,171	4345	358	0,000		2,338
Total		185853	13521			100,000

Compound **2m**

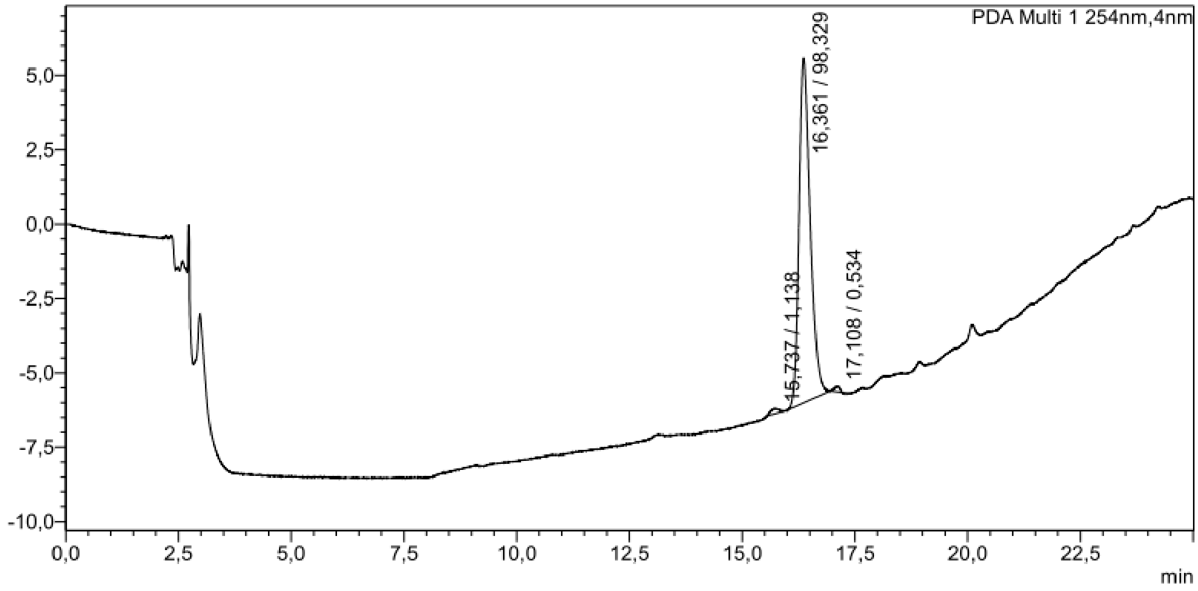

PDA Ch1 254nm						
Peak#	Ret. Time	Area	Height	Conc.	Unit	Area%
1	15,737	2338	169	0,000		1,138
2	16,361	202076	11576	0,000		98,329
3	17,108	1097	188	0,000		0,534
Total		205511	11933			100,000

Compound **18**

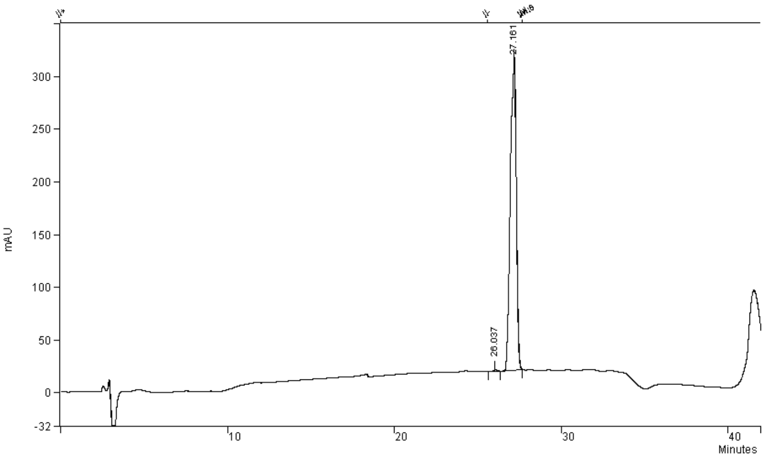

Peak No.	Peak Name	Result ()	Ret. Time (min)	Time Offset (min)	Area (counts)	Sep. Code	Width 1/2 (sec)	Status Codes
1		0.2532	26.037	0.000	186541	BP	15.1	
2		99.7468	27.161	0.000	73477808	PB	26.3	
	Totals:	100.0000		0.000	73664349			

Compound **28**

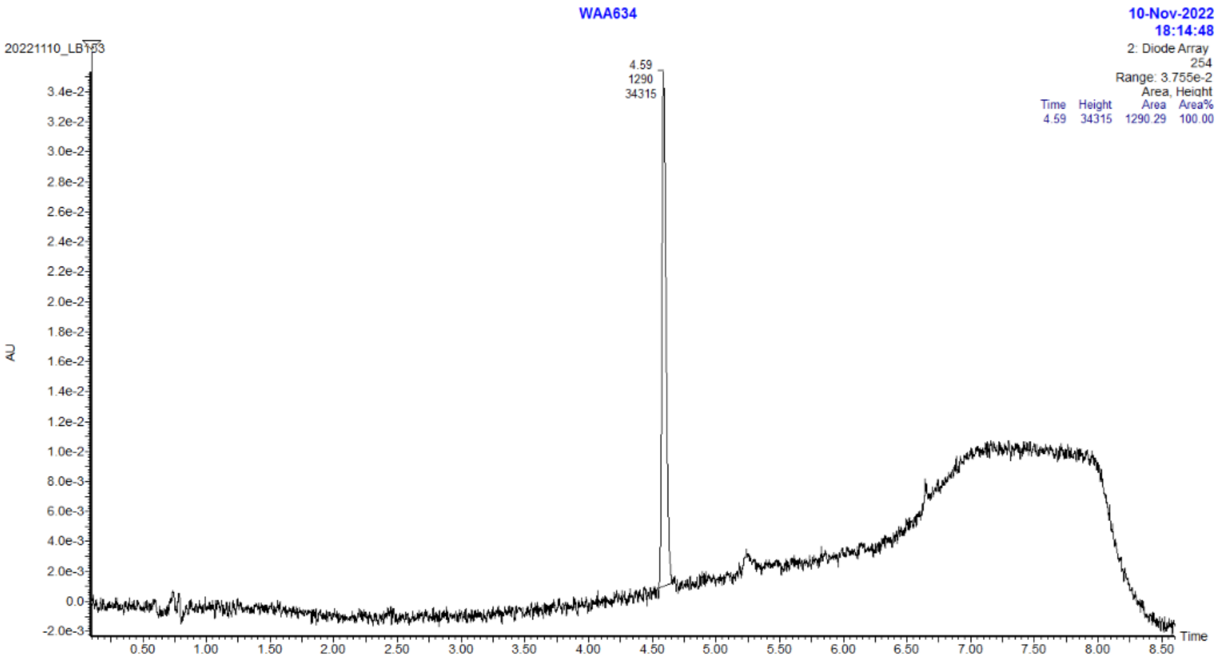

Compound **35a**

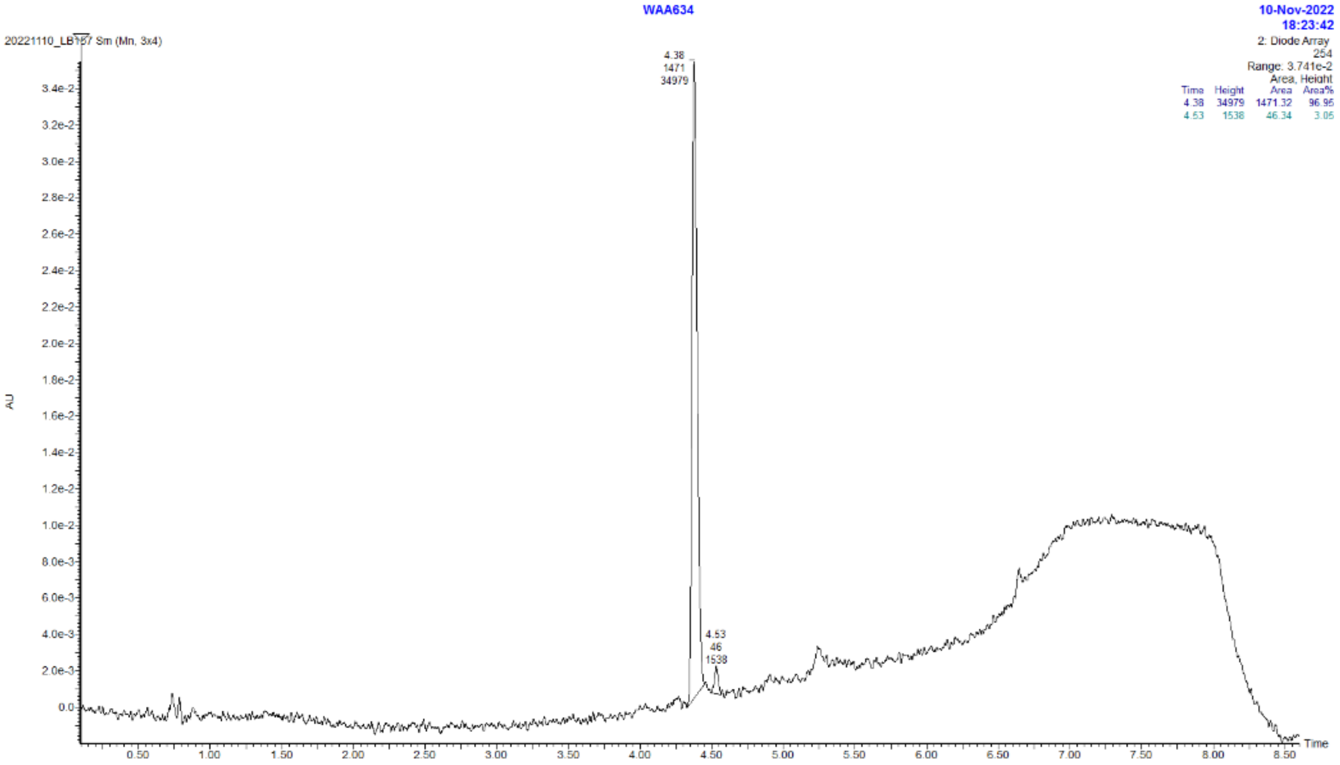

Compound **35b**

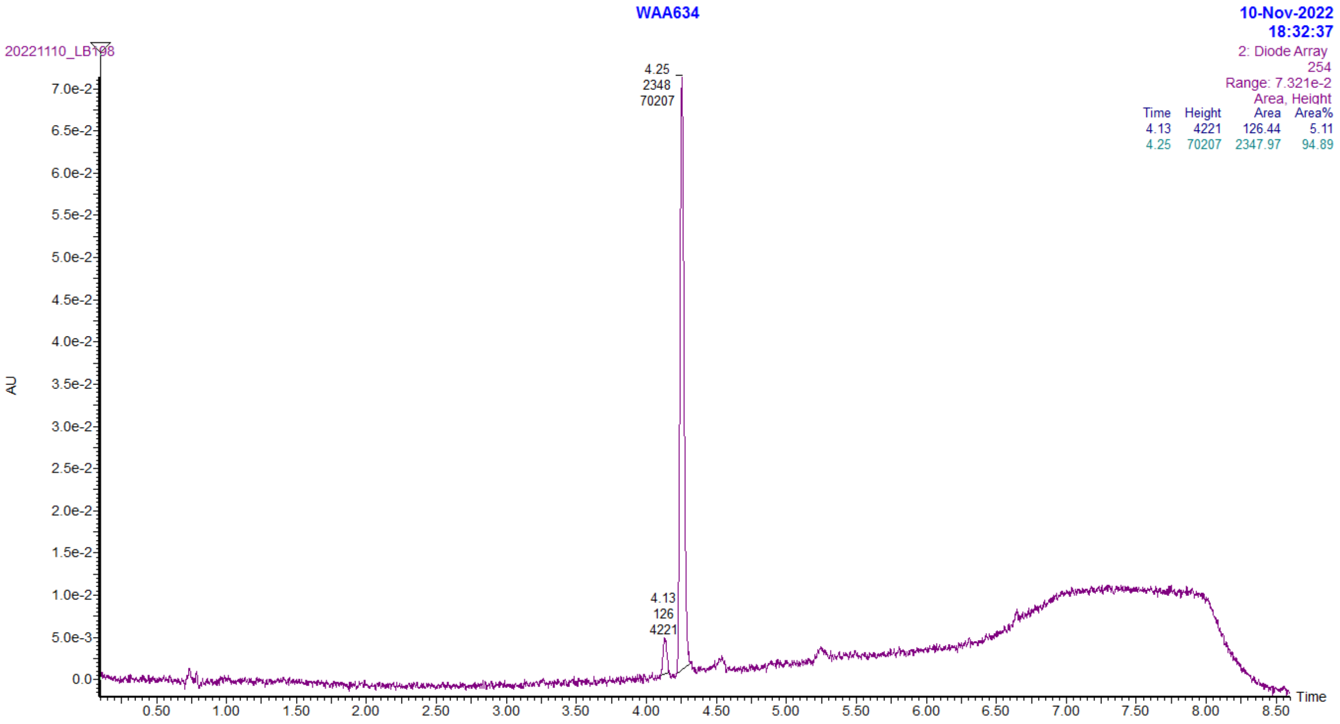

Compound **35c**

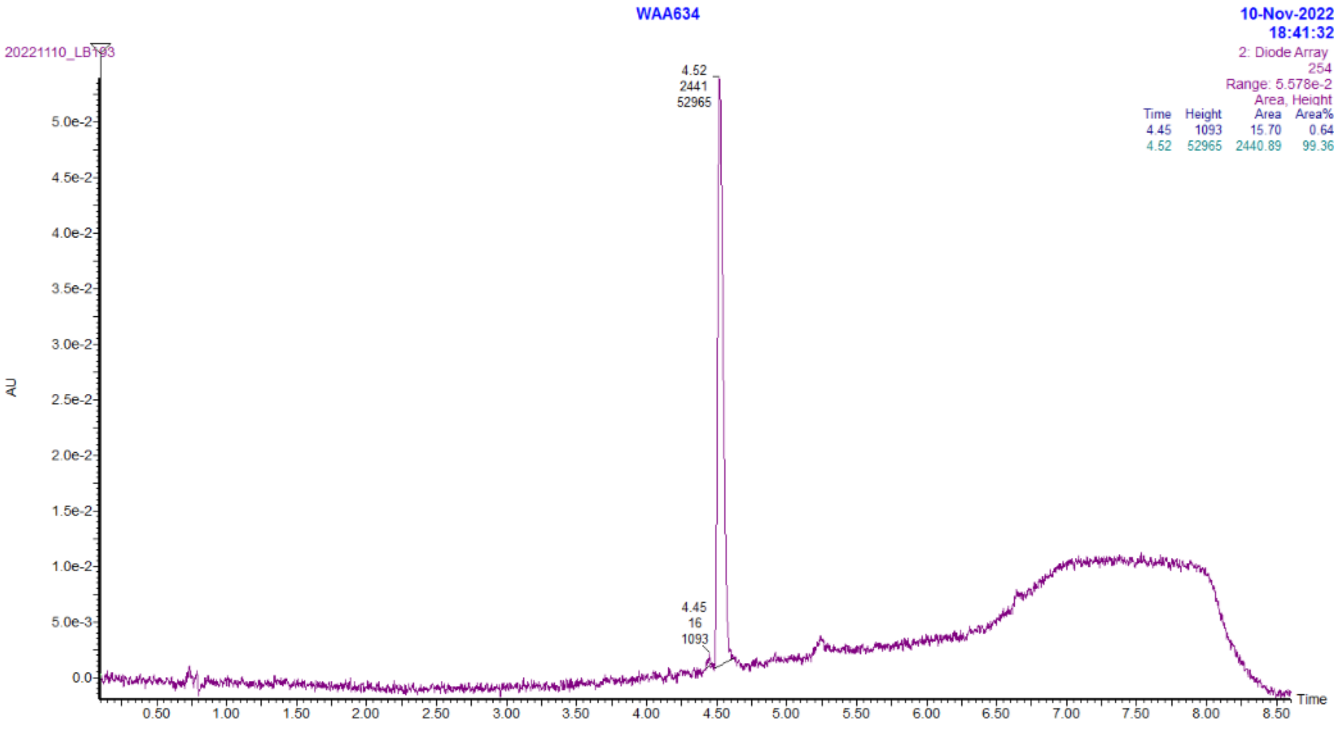

Compound **43**

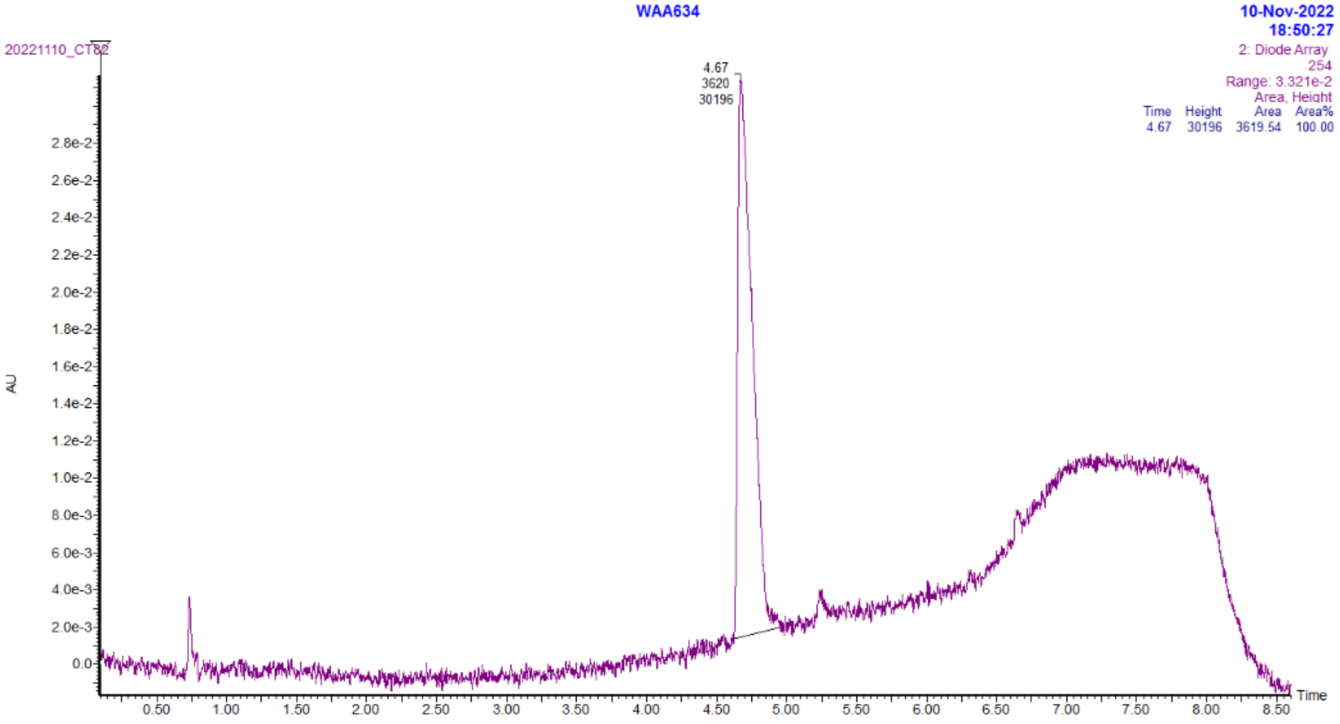

Compound **56a**

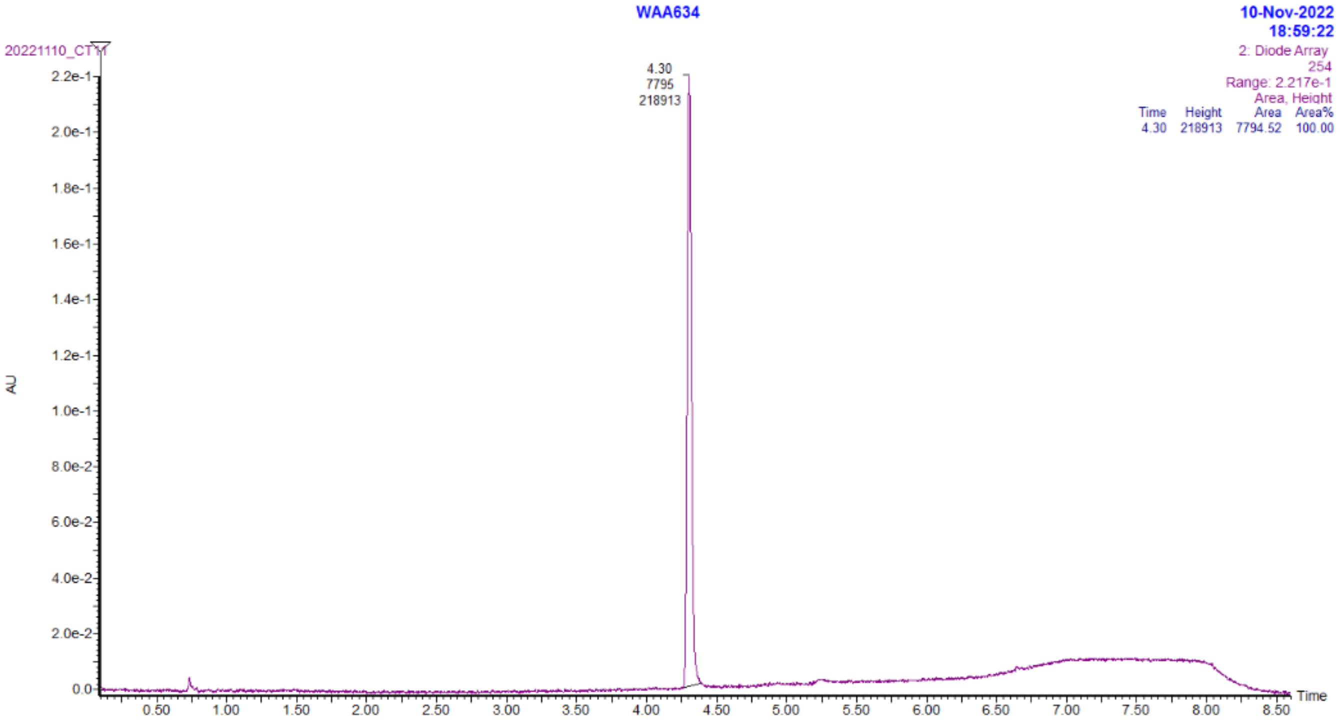

Compound **56c**

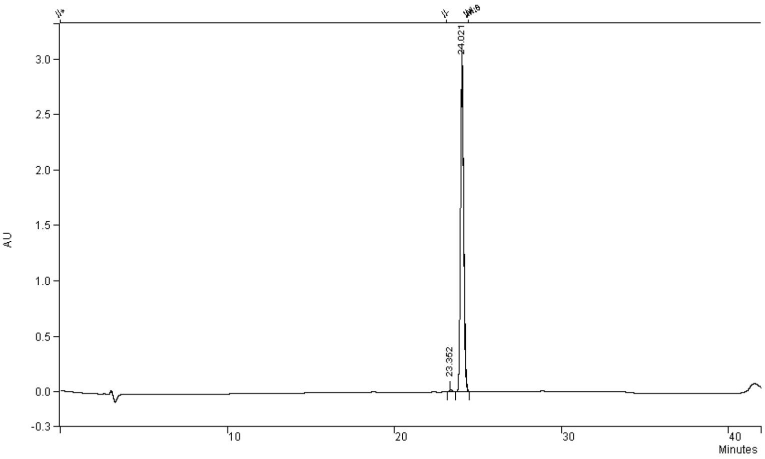

Peak No.	Peak Name	Result ()	Ret. Time (min)	Time Offset (min)	Area (counts)	Sep. Code	Width 1/2 (sec)	Status Codes
1		0.6199	23.352	0.000	2444970	BP	12.1	
2		99.3801	24.021	0.000	391965088	PB	12.2	
	Totals:	100.0000		0.000	394410058			

Compound **56d**

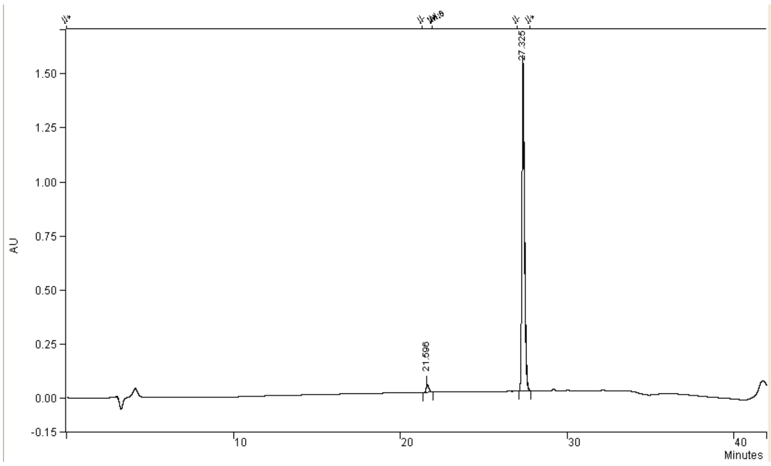

Peak No.	Peak Name	Result ()	Ret. Time (min)	Time Offset (min)	Area (counts)	Sep. Code	Width 1/2 (sec)	Status Codes
1		2.4080	21.596	0.000	3967278	BB	10.8	
2		97.5920	27.325	0.000	160785680	BB	9.3	
	Totals:	100.0000		0.000	164752958			

Compound **56e**

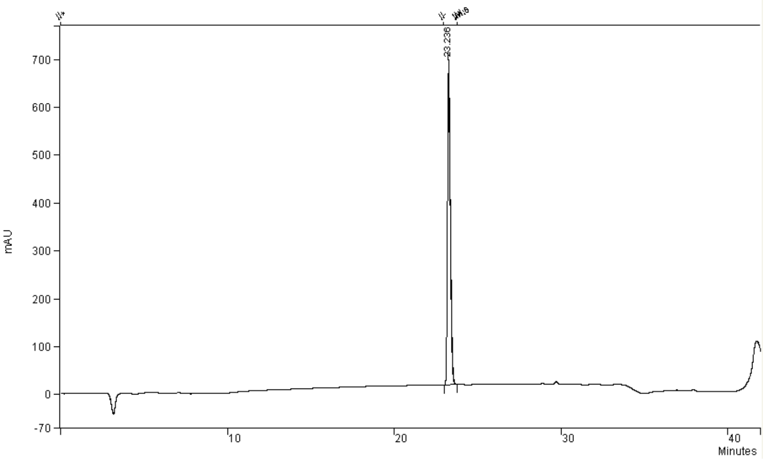

Peak No.	Peak Name	Result ()	Ret. Time (min)	Time Offset (min)	Area (counts)	Sep. Code	Width 1/2 (sec)	Status Codes
1		100.0000	23.236	0.000	78922848	BB	10.8	
	Totals:	100.0000		0.000	78922848			

Compound **56f**

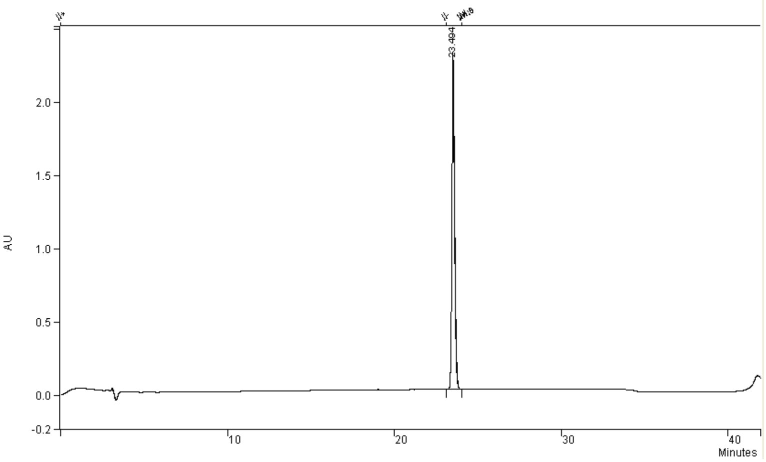

Peak No.	Peak Name	Result ()	Ret. Time (min)	Time Offset (min)	Area (counts)	Sep. Code	Width 1/2 (sec)	Status Codes
1		100.0000	23.494	0.000	248322720	BB	10.1	
	Totals:	100.0000		0.000	248322720			

Compound **62**

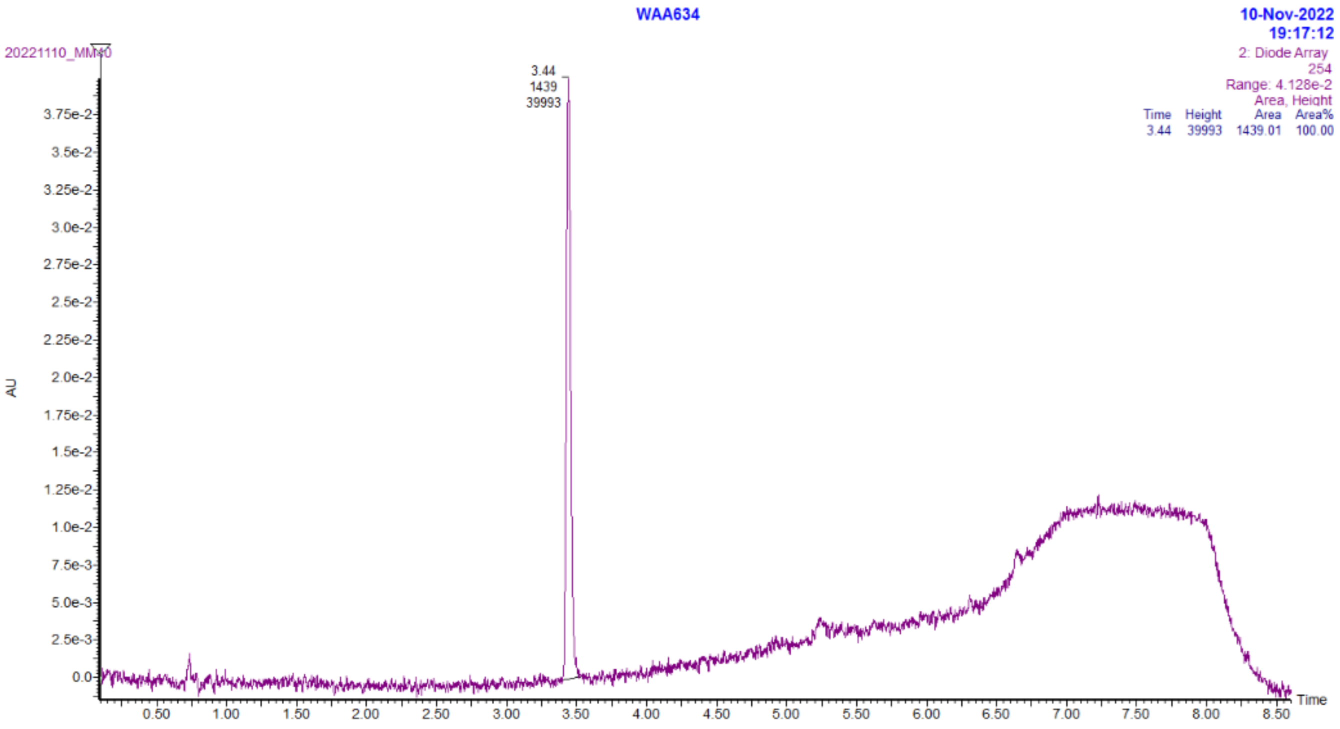

Compound **63**

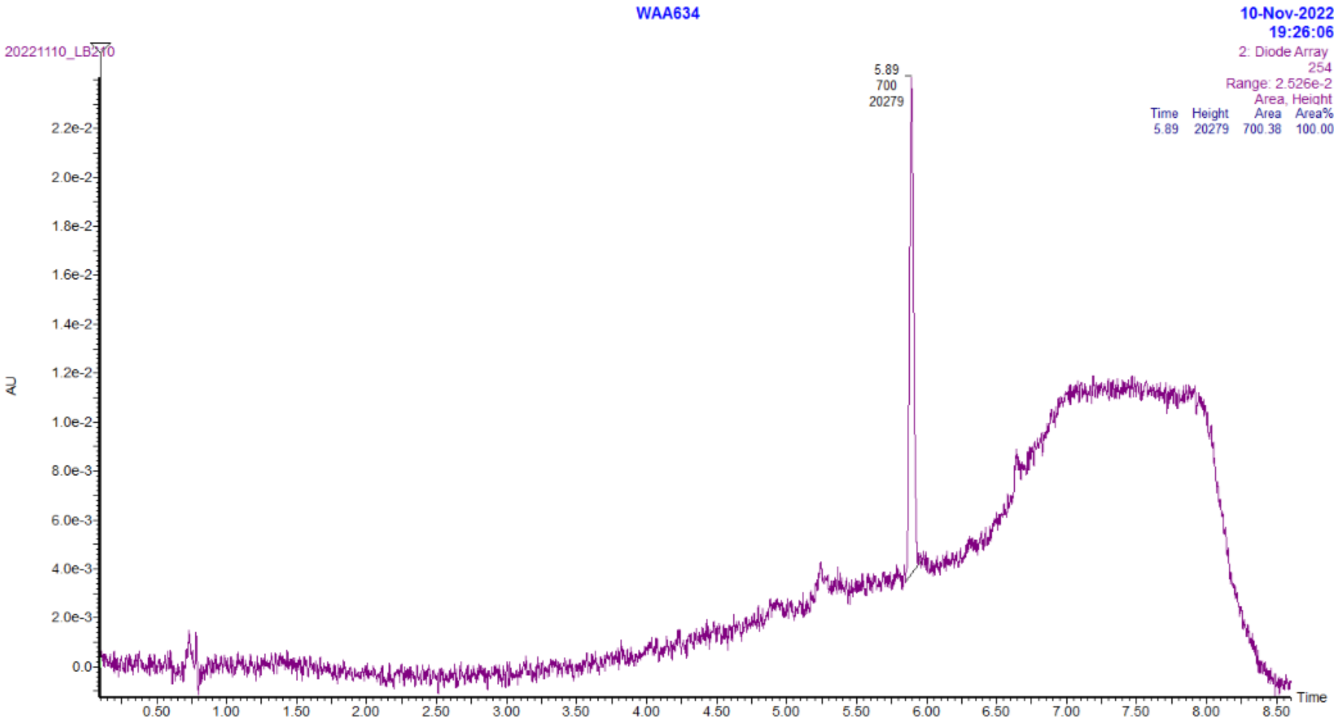

References for Supporting Information

1. Baici, A.; Novinec, M.; Lenarčič, B., Kinetics of the interaction of peptidases with substrates and modifiers. In *Proteases: Structure and Function*, Springer-Verlag: Wien, 2013; pp 37-84.
2. Schmitz, J.; Li, T.; Bartz, U.; Gütschow, M., Cathepsin B Inhibitors: Combining Dipeptide Nitriles with an Occluding Loop Recognition Element by Click Chemistry. *ACS Med. Chem. Lett.* **2016**, *7* (3), 211-216.
3. Cheng, Y.-C.; Prusoff, W. H., Relationship between the inhibition constant (KI) and the concentration of inhibitor which causes 50 percent inhibition (I50) of an enzymatic reaction. *Biochem. Pharmacol.* **1973**, *22* (23), 3099-3108.
4. Copeland, R. A., *Enzymes: A practical introduction to structure, mechanism, and data analysis*. 2nd ed.; Wiley-VCH, Inc.: 2000.
5. Lineweaver, H.; Burk, D., The determination of enzyme dissociation constants. *J. Am. Chem. Soc.* **1934**, *56* (3), 658-666.
6. Dixon, M., The determination of enzyme inhibitor constants. *Biochem. J.* **1953**, *55* (1), 170-171.
7. Segel, I. H., *Enzyme kinetics. Behaviour and analysis of rapid equilibrium and steady-state enzyme systems*. John Wiley & Sons Inc.: 1993.
8. Gobbi, L.; Mercier, J.; Bang-Andersen, B.; Nicolas, J.-M.; Reilly, J.; Wagner, B.; Whitehead, D.; Briard, E.; Maguire, R. P.; Borroni, E.; Auberson, Y. P., A Comparative Study of in vitro Assays for Predicting the Nonspecific Binding of PET Imaging Agents in vivo. *ChemMedChem* **2020**, *15* (7), 585-592.
9. Auberson, Y. P.; Briard, E.; Sykes, D.; Reilly, J.; Healy, M., Ligand specific efficiency (LSE) index for PET tracer optimization. *ChemMedChem* **2016**, *11* (13), 1415-1427.
10. Smith, P. K.; Krohn, R. I.; Hermanson, G. T.; Mallia, A. K.; Gartner, F. H.; Provenzano, M. D.; Fujimoto, E. K.; Goeke, N. M.; Olson, B. J.; Klenk, D. C., Measurement of protein using bicinchoninic acid. *Anal. Biochem.* **1985**, *150* (1), 76-85.
11. Dale, J. A.; Mosher, H. S., Nuclear magnetic resonance enantiomer regents. Configurational correlations via nuclear magnetic resonance chemical shifts of diastereomeric mandelate, O-methylmandelate, and alpha-methoxy-alpha-trifluoromethylphenylacetate (MTPA) esters. *J. Am. Chem. Soc.* **1973**, *95* (2), 512-519.
12. Hoye, T. R.; Jeffrey, C. S.; Shao, F., Mosher ester analysis for the determination of absolute configuration of stereogenic (chiral) carbinol carbons. *Nat. Protoc.* **2007**, *2* (10), 2451-8.
13. Seco, J. M.; Quiñoá, E.; Riguera, R., The Assignment of Absolute Configuration by NMR. *Chem. Rev.* **2004**, *104* (1), 17-118.
14. Lehane, K. N.; Moynihan, E. J. A.; Brondel, N.; Lawrence, S. E.; Maguire, A. R., Impact of sulfur substituents on the C–H···O interaction of terminal alkynes in crystal engineering. *CrystEngComm* **2007**, *9* (11).
15. Raimundo, B. C.; Oslob, J. D.; Braisted, A. C.; Hyde, J.; McDowell, R. S.; Randal, M.; Waal, N. D.; Wilkinson, J.; Yu, C. H.; Arkin, M. R., Integrating Fragment Assembly and Biophysical Methods in the Chemical Advancement of Small-Molecule Antagonists of IL-2: An Approach for Inhibiting Protein−Protein Interactions. *J. Med. Chem.* **2004**, *47* (12), 3111-30.
16. Greenspan, P. D.; Clark, K. L.; Tommasi, R. A.; Cowen, S. D.; McQuire, L. W.; Farley, D. L.; van Duzer, J. H.; Goldberg, R. L.; Zhou, H. H.; Du, Z. M.; Fitt, J. J.; Coppa, D. E.; Fang, Z.; Macchia, W.; Zhu, L. J.; Capparelli, M. P.; Goldstein, R.; Wigg, A. M.; Doughty, J. R.; Bohacek, R. S.; Knap, A. K., Identification of dipeptidyl nitriles as potent and selective inhibitors of cathepsin B through structure-based drug design. *J. Med. Chem.* **2001**, *44* (26), 4524-4534.
17. Dondoni, A.; Perrone, D.; Gleason, M. M.; Roush, W. R., Synthesis of 1,1-dimethylethyl (S)-4-formyl-2,2-dimethyl-3-oxazolidinecarboxylate by oxidation of the alcohol. *Org. Synth.* **2000**, *77*, 64.
18. Meffre, P.; Boibessot, T.; Bénimèlis, D.; Jean, M.; Benfodda, Z., Synthesis of a novel rhizobitoxine-like triazole-containing amino acid. *Synlett* **2016**, *27* (19), 2685-2688.

19. Usuki, T.; Yamada, H.; Hayashi, T.; Yanuma, H.; Koseki, Y.; Suzuki, N.; Masuyama, Y.; Lin, Y. Y., Total synthesis of COPD biomarker desmosine that crosslinks elastin. *Chem. Commun. (Camb.)* **2012**, *48*, 3233-3235.
20. Meffre, P.; Gauzy, L.; Branquet, E.; Durand, P.; Goffic, F. L., Synthesis of optically active β,γ-alkynylglycine derivatives. *Tetrahedron* **1996**, *52*, 11215-11238.
21. Snyder, J. K.; Stock, L. M., Conformational preferences in alkylnitrosoureas. *J. Org. Chem.* **1980**, *45* (5), 886-891.
